# Whole genome analysis for plant growth promotion profiling of *Pantoea agglomerans* CPHN2, a non-rhizobial nodule endophyte

**DOI:** 10.3389/fmicb.2022.998821

**Published:** 2022-11-07

**Authors:** Pradeep Kumar, Simran Rani, Priyanka Dahiya, Ajit Kumar, Amita Suneja Dang, Pooja Suneja

**Affiliations:** ^1^Department of Microbiology, Maharshi Dayanand University, Rohtak, India; ^2^Centre for Bioinformatics, Maharshi Dayanand University, Rohtak, India; ^3^Centre for Medical Biotechnology, Maharshi Dayanand University, Rohtak, India

**Keywords:** KEGG pathway, next-generation sequencing, *Pantoea agglomerans*, PGPEB, whole genome

## Abstract

Reduced agricultural production as well as issues like nutrient-depleted soils, eutrophication, and groundwater contamination have drawn attention to the use of endophyte-based bioformulations to restore soil fertility. *Pantoea agglomerans* CPHN2, a non-rhizobial nodule endophyte isolated from *Cicer arietinum*, exhibited a variety of plant growth-promoting traits. In this study, we used NextSeq500 technology to analyze whole-genome sequence information of this plant growth-promoting endophytic bacteria. The genome of *P. agglomerans* CPHN2 has a length of 4,839,532 bp and a G + C content of 55.2%. The whole genome comprises three different genomic fractions, comprising one circular chromosome and two circular plasmids. A comparative analysis between *P. agglomerans* CPHN2 and 10 genetically similar strains was performed using a bacterial pan-genome pipeline. All the predicted and annotated gene sequences for plant growth promotions (PGPs), such as phosphate solubilization, siderophore synthesis, nitrogen metabolism, and indole-3-acetic acid (IAA) of *P. agglomerans* CPHN2, were identified. The whole-genome analysis of *P. agglomerans* CPHN2 provides an insight into the mechanisms underlying PGP by endophytes and its potential applications as a biofertilizer.

## Graphical Abstract

Overview of Pantoea agglomerans CPHN2 whole genome study and pathway analysis. 
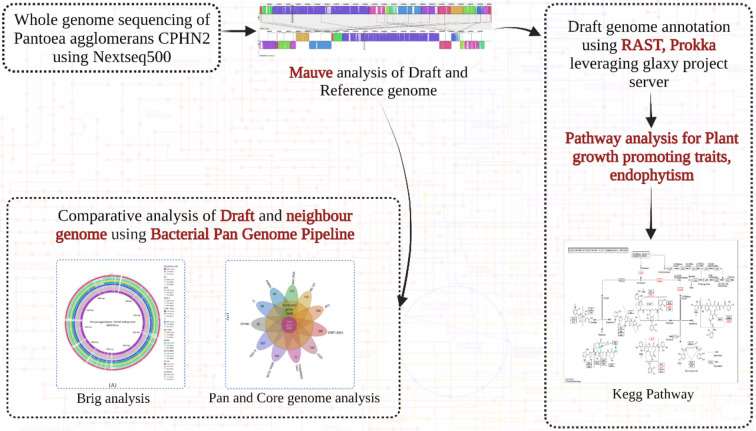

## Introduction

*Pantoea*, a highly versatile member of family Enterobacteriaceae, capable of adapting to diverse range of environment conditions, has more than twenty recognized species assigned to it (Walterson and Stavrinides, 2015). It has also been reported that, in addition to inhabiting a variety of hosts as parasites, *Pantoea* occurs as a mutualistic associate, exhibiting the true versatility of its nature. It has been isolated from different habitats, such as soil, water, plants, humans, and animals (Lee et al., 2010; Walterson and Stavrinides, 2015). *Pantoea* was previously considered an enemy because of its pathogenicity, but it is now recognized as a friend after the establishment of its beneficial effects such as its potential for biocontrol and plant growth promotion (PGP) (Dutkiewicz et al., 2016; Kukreti et al., 2020; Agri et al., 2021, 2022).

*Pantoea agglomerans* CPHN2, non-rhizobial plant growth promoting endophytic bacteria (PGPEB) was isolated from *Cicer arietinum* nodules. This strain possessed the ability to produce ammonia, phytohormone indole-3-acetic acid (IAA), and also able to solubilise phosphate (Maheshwari et al., 2019; Rani et al., 2021). Indole acetic acid (IAA), an important phytohormone, plays a crucial role in plant physiology and development. Under optimized conditions, this strain was also able to produce high amount of IAA (unpublished). Multiple IAA biosynthetic pathways have been reported in both PGPEBs and plants. These pathways can be characterized by metabolic intermediates, enzymes, and mutants or by genomic studies (Duca et al., 2014; Navarro-Torre et al., 2017; Chi et al., 2018; Duca and Glick, 2020; Dudeja et al., 2021).

Whole-genome shotgun (WGS) sequencing provides insights into complete data of chromosomes and helps in the identification of significant target genes in terms of PGP activity in addition to yielding information on their interactions with plants. Recently, a large number of whole-genome sequences of PGPEBs, such as *Bacillus, Pseudomonas, Pantoea*, and *Enterobacter*, have been studied (Verheggen et al., 2016). In the present study, whole-genome sequencing of *P. agglomerans* CPHN2 was carried out with the aim of unraveling the underlying molecular mechanisms, functional potential, and taxonomy. Comparative genomics was carried out by evaluating its proximity to related strains and by comparing its whole genome to 10 closely related strains in terms of the core and pan-genomes.

## Materials and methods

### Bacterial sample

*Pantoea agglomerans* CPHN2 for this study was taken from the Plant Microbe Interaction Laboratory, Department of Microbiology, Maharshi Dayanand University, Rohtak. The isolate was initially grown at 30°C ± 2°C on tryptone soya agar (TSA) medium and then purified.

### Genome sequencing and annotations

The colonies were sent for whole genome sequencing to Eurofins Genomics India Pvt. Ltd. Bacterial deoxyribonucleic acid (DNA) was isolated from pure culture using the Quick-DNA Miniprep Plus kit (Zymo Research), according to the kit procedure. The quality and quantity of the extracted genomic DNA (gDNA) were tested using the NanoDrop Spectrophotometer.

The whole genome was sequenced using the Illumina NextSeq500 platform. The paired-end (PE) sequencing library was constructed from 1 mg of genomic DNA samples using the illuminaTruSeq Nano DNA Library Prep kit as per the manufacturer's protocol. The quality of raw reads was checked with FastQC v0.11.8 (Andrews, 2010) and filtered and trimmed with fastp v0.201 (Chen et al., 2018), and *de novo* genome assemblies were performed with Shovill v1.0.4 (Seemann, 2017). Genome identification and annotations were performed with Galaxy (https://usegalaxy.org/) as well as with RAST (https://rast.nmpdr.org/). Several general features of genomes such as transfer ribonucleic acid (tRNA) and ribosomal RNA (rRNA) were filtered and reported using the in-house script provided by the annotation tools. The annotated genes were then analyzed to determine their role in IAA production and in other plant growth-promoting pathways.

### Whole-genome and pan-genome analysis

The genome of *P. agglomerans* CPHN2 was compared to 10 closely related genomes available in NCBI databases using 16s rRNA with WGS contigs. Several genomic features, such as genome size, gene number, GC content, and the number of tRNAs and rRNA, were compared. BRIG 0.95 was used to construct a pairwise genomic alignment of all the 11 strains and an out-group (Alikhan et al., 2011).

To study the rearrangement and alignment of genomes, *P. agglomerans* CPHN2 (draft genome) was compared with *P. agglomerans* FDAARGOS1447 (GenBank accession no. CP077366.1) using the Mauve program in Mauve v 2.3.1 (Darling et al., 2004). By taking the genome of *P. agglomerans* CPHN2 and its 10 neighbor strains, BPGA was used to perform pan-genome analysis (Chaudhari et al., 2016) to identify strain-specific and core genes (Shariati et al., 2017; Pinski et al., 2020).

### Pathway analysis

To determine the presence of specific pathways for plant growth-promoting traits, annotated and predicted gene sequences were evaluated. The ascribed gene functions were manually analyzed from previously reported studies (Glick, 2012; Ahemad and Kibret, 2014; Ke et al., 2016; Liu et al., 2016; Dudeja et al., 2021) and compared with the relevant pathways available in the Kyoto Encyclopedia of Genes and Genomes (KEGG) pathway databases (https://www.genome.jp/kegg/pathway.html).

## Results

### *P. agglomerans* CPHN2 sequencing statistics

Illumina NextSeq500 technology was used to sequence the whole genome of *P. agglomerans* CPHN2, which resulted in 100-fold coverage. In total, 9,731,611 PE reads with a minimum length of 100 bp were filtered for low-quality sequences (reads with more than 10% quality threshold (QV) 20 Phred score) and ambiguous reads (reads with unknown nucleotides “N” >5%) (Supplementary Table 1). Finally, in the downstream analysis, a total of 2,898,434,450 bp were used. The reads were assembled, and the total length of 4,839,532 bp was generated. In the final assembly, a total of 32 contigs were obtained, each with a total of 4,424 coding sequences (CDS), including N50 of size 558,390 bp (Supplementary Table 2).

### Genomic characteristics of *P. agglomerans* CPHN2 and comparative genomic analysis

**Figure 1:**
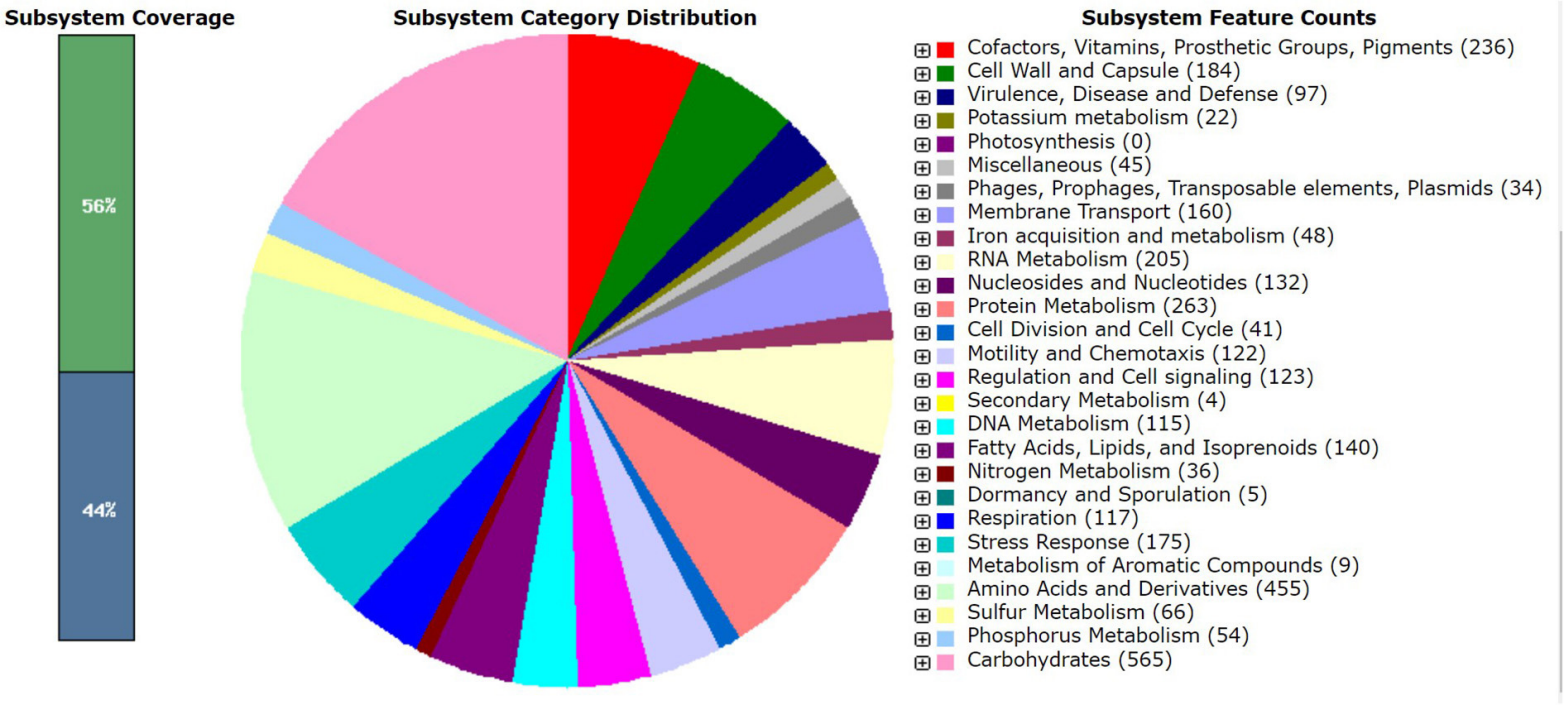
RAST annotations of *Pantoea agglomerans* CPHN2 whole genome.

**Figure 2:**
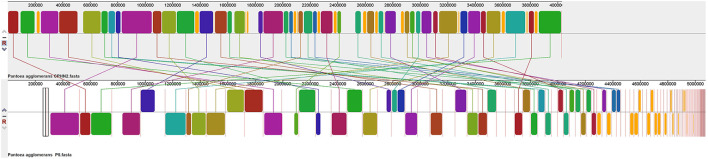
FDAARGOS 1447 and *P. agglomerans* CPHN2 genomes are compared using Mauve 2.3.1 software.

**Figure 3:**
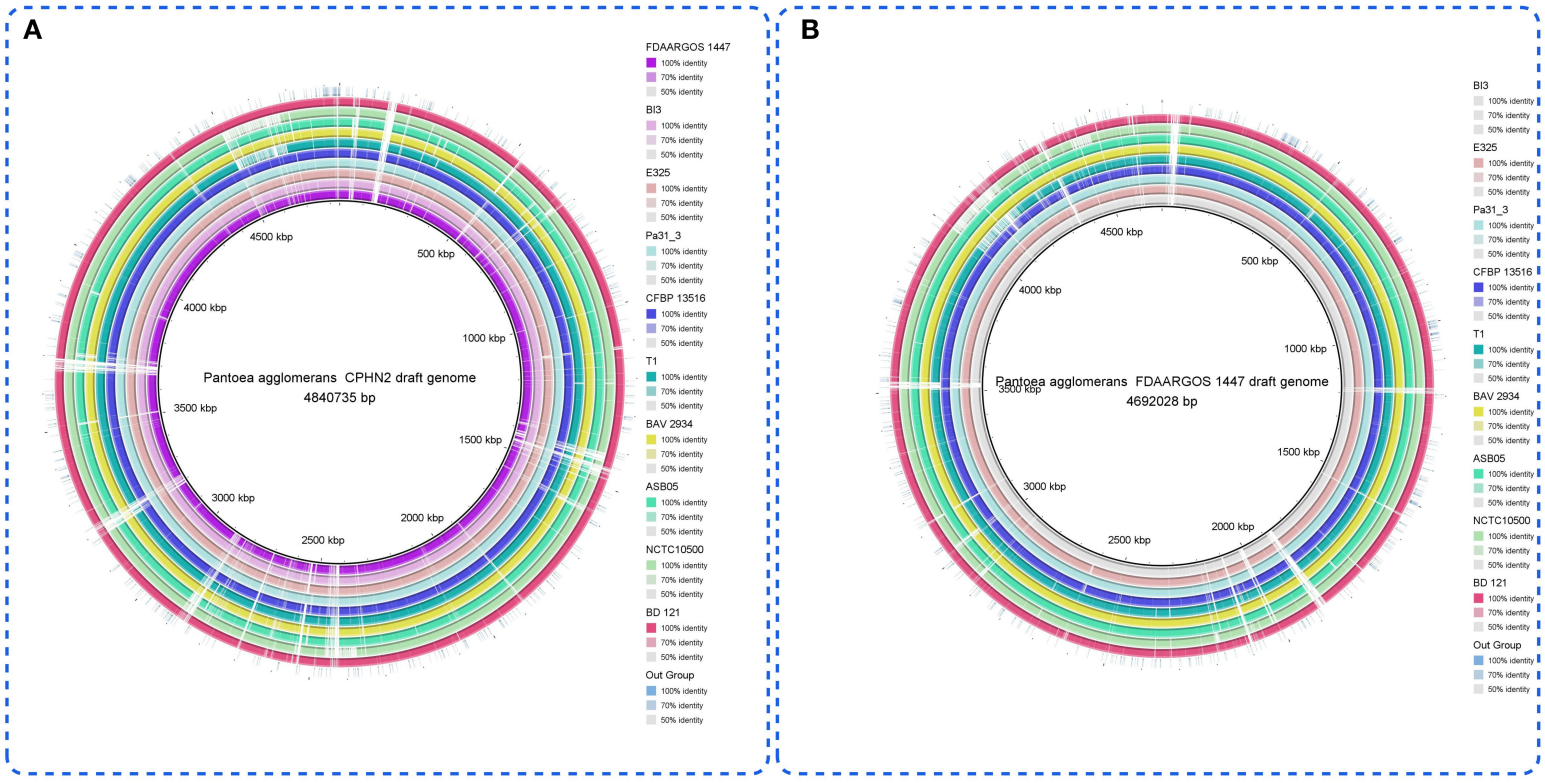
**(A)** Genome comparisons of different *P. agglomerans* strains to the drafted CPHN2 genome created by BRIG 0.95. The inner cycle (back) reflects the whole genome of the reference strain CPHN2, and the shade of each color indicates the similarities between all strains with the strain CPHN2. **(B)** With genome comparisons of other *P. agglomerans* strains except for CPHN2 vs. the reference genome (FDAARGOS 1447), the circular map depicts the full-genome comparison of the strain FDAARGOS 1447 against the other 10 sequenced *P. agglomerans* and an out group.

**Figure 4:**
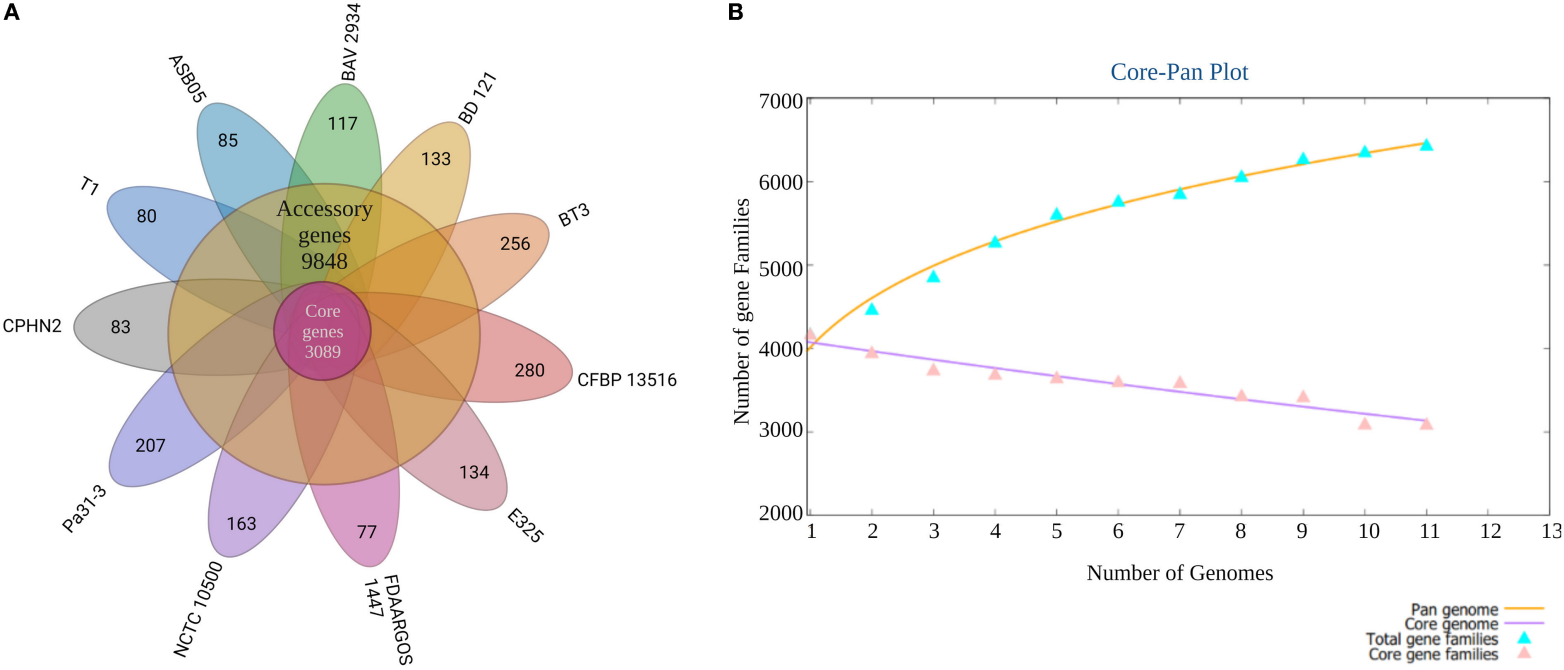
A plot of the core/pan-genome of *P. agglomerans* strains and a Venn diagram of the core-genome and strain-specific coding sequences (CDS). **(A)** The number of distinct CDS for each strain of the pan-genome of *P. agglomerans*. **(B)** Genome comparisons using *n* = 11 genomes were performed to identify the core-genome and strain-specific CDS for the 11 *P. agglomerans* strains sequenced.

*Pantoea agglomerans* CPHN2 has a single circular chromosome approximately 4.8 Mbp long, with a GC content of 55.2% and two plasmids. RAST (https://rast.nmpdr.org/) was used to predict a total of 4,468 putative CDS (Figure 1). The chromosomal sequence predicted 71 tRNA-coding genes, one rRNA gene, and one CRISPR. The closely related genome of *P. agglomerans* FDAARGOS 1447 was used as the reference genome for preliminary comparative analysis based on 16S rRNA sequences. The ordered genome assembly of CPHN2 and strain FDAARGOS 1447 (GenBank accession no. CP077366.1) were compared with progressive Mauve from Mauvev 2.3.1 software. The genomic alignment identified 12 collinear blocks, as well as many inversion and rearrangement sites (Figure 2). By mapping most of the portions of the two genomes, large areas of high similarity were observed, indicating that the chromosome alignments of both strains are approximately identical. However, a region in the chromosome scaffolding between contigs 4–9 and 11 has an inverted orientation, indicating differences in their synteny relationships. In addition, the genomic features of *P. agglomerans* CPHN2 and 10 closely related genomes were analyzed based on genome size, the number of genes, the predicted CDS, GC content distribution, and the number of tRNA and rRNA genes (Figures 3A,B, Table 1). The genome size of CPHN2 was lower than that of the strains BI3, Pa31 3, and CFBP13516 with a genome size of more than 5 Mb. Of these, Pa31_3 has approximately the genome size of 5.0964 Mb, which is relatively large among all genomes. The draft genome of CPHN2 has 83 unique CDS (Figures 4A,B).

**Table 1:** *Pantoea agglomerans* strains genome used for comparative study.

**Strain**	**CPHN2**	**ASD05**	**BAV 2934**	**BD 1212**	**BI3**	**CFBP13516**	**E325**	**FDAARGOS1447**	**NCTC10500**	**Pa31_3**	**T1**
Taxonomy	Bacteria; *Proteobacteria; Gammaproteobacteria; Enterobacterales; Erwiniaceae; Pantoea; Pantoea agglomerans group; Pantoea agglomerans*										
Taxonomy ID	549
Size (mb)	4,839,757	4.8586	4.9449	4.8754	5.0477	5.0262	4.8038	4.692	4.6555	5.0964	4.5307
GC content (%)	55.2	55	54.9	55.1	55.2	54.8	55.2	55.1	55.2	55	55.4
N50	558,390	4,022,781	4,003,977	125,314	418,672	186,387	337,568	3,999,686	460,684	294,867	22,549,703
L50	2	1	1	13	3	8	4	1	1	6	1
Number of contigs (with PEGs)	32	4	5	103	24	54	96	3	4	105	17
Number of subsystems	529	340	337	345	349	354	341	337	340	345	334
Number of coding sequences	4,424	4,655	4,754	4,745	4,917	4,974	4,633	4,461	4,464	5,049	4,258
Number of RNAs	84	98	97	56	83	76	92	98	98	85	74

### Intra-species phylogenetic tree analysis

**Figure 5:**
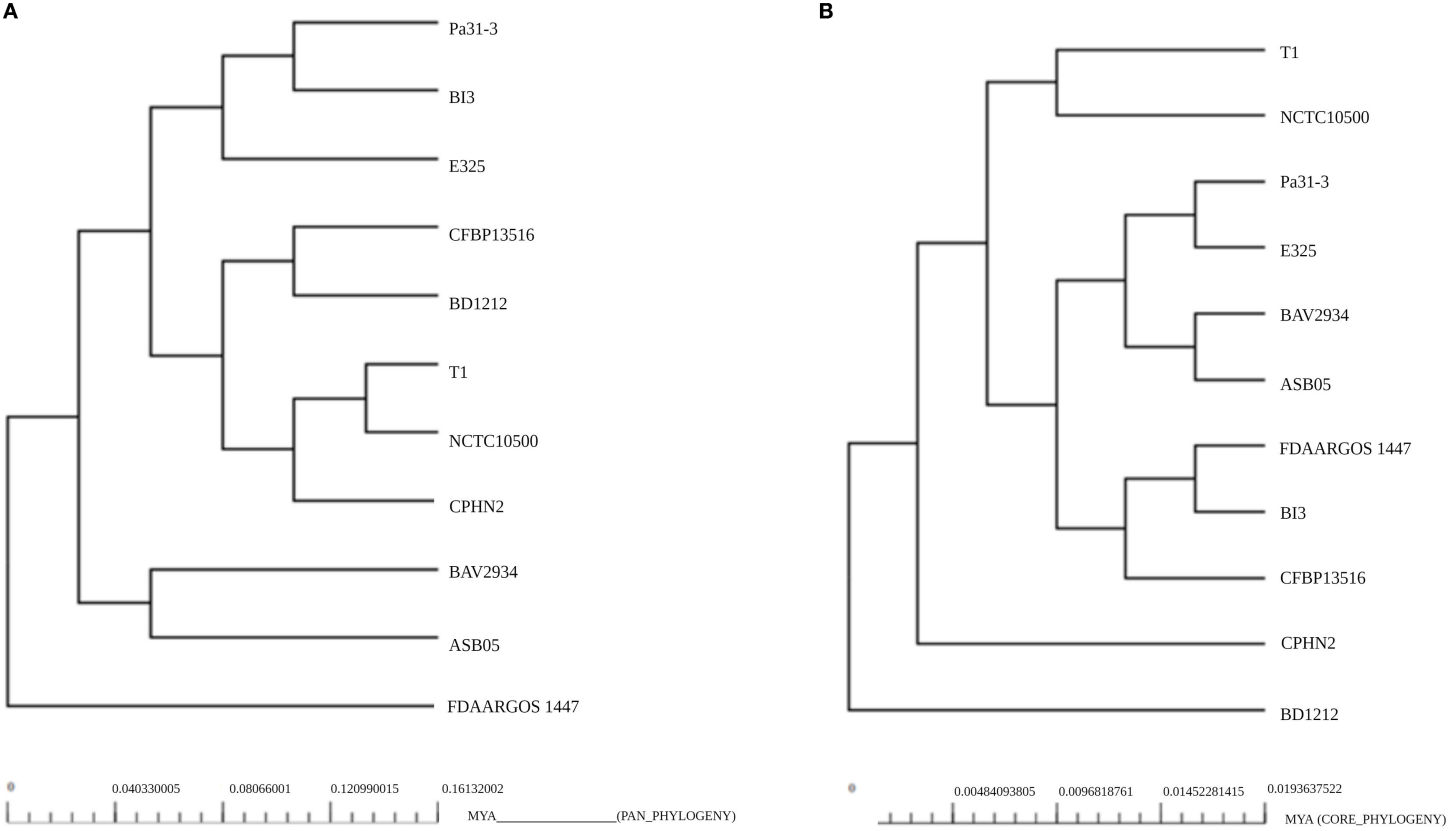
Phylogeny of the *P. agglomerans* strains based on the analysis of **(A)** pan-genomes and **(B)** core genomes.

The phylogenetic relationships of *P. agglomerans* CPHN2 and 10 other *P. agglomerans* strains were shown separately based on the pan genome (Figure 5A) and the core genome (Figure 5B). This analysis depicted *P. agglomerans strain* CPHN2, genetically close to the NCTC10500 and T1 genomes, clustered in the same clade based on the pan-genome. The analysis also revealed a distant phylogenetic relationship between the strain FDAARGOS 1447 and other strains. In a core-genome-based phylogenetic study, CPHN2 did not cluster in any clade, while FDAARGOS 1447 clustered along with BI3 and CFBP13516 (Figure 5).

### Carbohydrate metabolism

The CPHN2 genome comprises a number of carbohydrate metabolism subsystems that encompass around 12.7% of the coding area. The genome has a number of metabolic pathways for carbohydrate metabolisms such as glycolysis, gluconeogenesis, Entner–Doudoroff, the tricarboxylic acid (TCA) cycle, the pentose phosphate pathway, and acetyl CoA fermentation. It also has genes that metabolize the amount of other sugars and their derivatives (Supplementary Table 3).

### Genes involved in plant growth stimulation

Genes involved in the solubilization of phosphates and in the production of organic acids, siderophores, ammonia, and indole acetic acid (IAA) have been annotated and identified in the *P. agglomerans* CPHN2 genome (Glick, 2012; Ahemad and Kibret, 2014; Liu et al., 2016).

#### Phosphate and organic acids

It is well-known that, after being applied as a fertilizer, a significant portion of inorganic phosphates is immobilized, leaving the phosphate unavailable to plants (Xie et al., 2016). As a consequence, it is crucial for some bacterial species to produce acid phosphatases and organic acids, notably gluconic acid (GA), and to solubilize insoluble or poorly soluble mineral phosphates (Rodríguez et al., 2007). GA is a kind of organic acid that aids in converting the immobilized mineral phosphates that contain phosphorus into a biologically available form. Glucose-1-dehydrogenase (*gcd*) along with its cofactor, pyrrolo-quinolone quinine (*pqq*), catalyzes the synthesis of GA. Gluconate dehydrogenase is another enzyme that also helps in the production of GA and its conversion to 2-ketogluconate (Ramachandran et al., 2006; Eastman et al., 2014). CPHN2 genomic annotation showed the existence of a number of GA and cofactor genes, including *pqq*ABCDE (Eastman et al., 2014).

**Figure 6:**
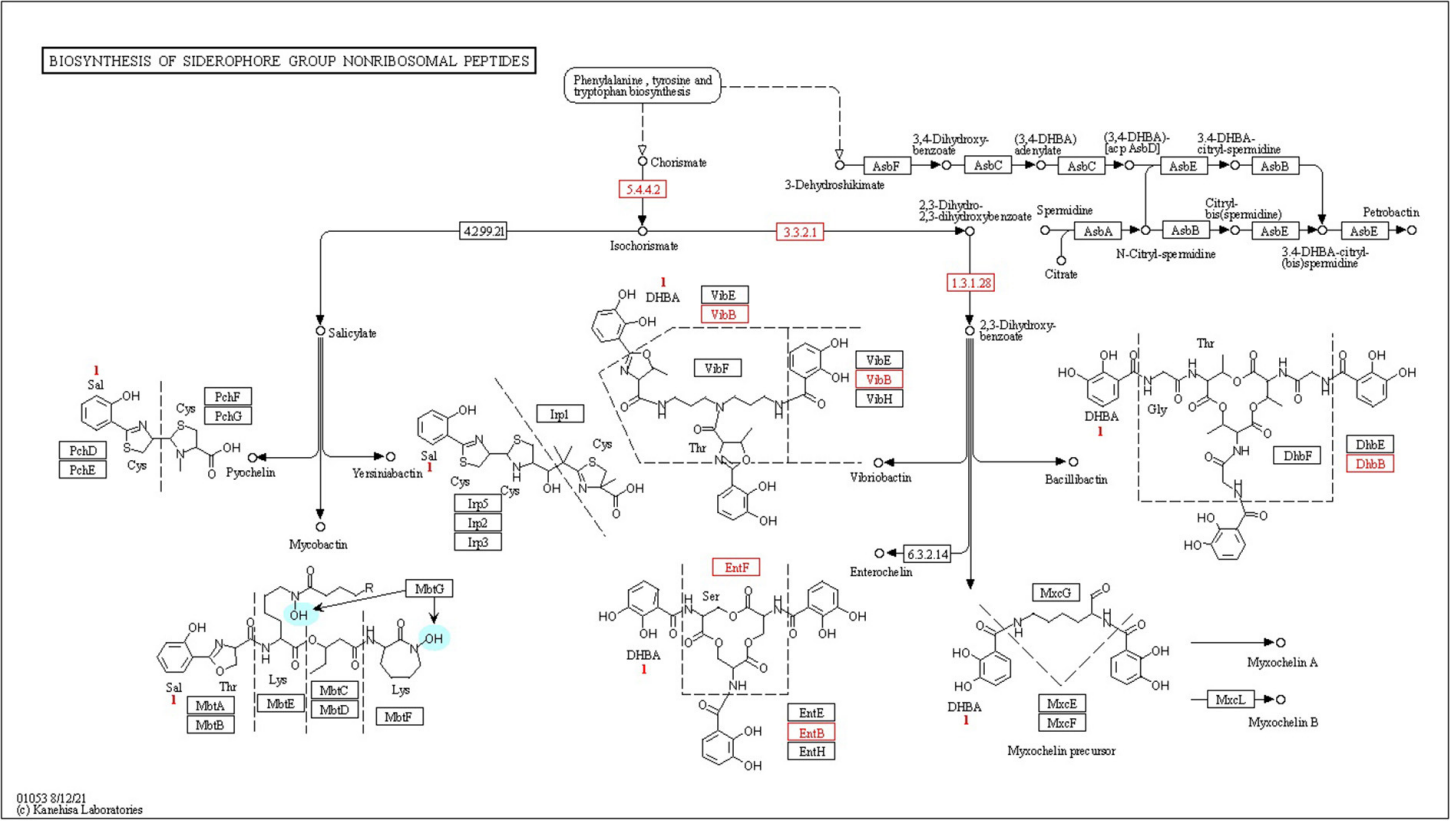
Kyoto Encyclopedia of Genes and Genomes (KEGG) pathway gene mapping of the strain CPHN2 for siderophore synthesis.

Phosphonates, which are organophosphorus molecules, have a direct carbon–phosphorus (C–P) bond instead of a typical C–O–P linkage that is often observed in phosphate esters (C–O–P) (Parker et al., 1999). Bacterial breakdown of phosphonates results in the release of physiologically available phosphates, which are controlled by the phosphonate-related *phn* gene cluster. CPHN2 has numerous *phn* genes, including *phn*CDFGHIJKLMNOPRV, which catalyze the hydrolysis of phosphonate into phosphate and an alkane, according to genomic analysis. Phosphorus compounds must be transported across the plasma membrane before they may be used. Two high-affinity phosphate transport systems, *pst*BAC (phosphate transporter) and *phn*DC (phosphate dehydrogenase), have also been observed in the CPHN2 genome (phosphonate transporter) (Figure 6, Table 2, Supplementary Table 4).

**Table 2:** *P. agglomerans* strain CPHN2 genomic annotation for phosphate solubilization.

**Gene name**	**Gene product**
*gcd*	Quinoprotein glucose dehydrogenase
*pqq*B	Coenzyme PQQ synthesis protein B
*pqq*C	Pyrroloquinoline-quinone synthase
*pqq*D	*pqq*A binding protein
*pqq*E	*pqq*A peptide cyclase
*pst*A	Phosphate transport system permease protein *pst*A
*pst*B	Phosphate-import ATP-binding protein *pst*B
*pst*C	Phosphate transport system permease protein *pst*C
*pst*S	Phosphate-binding protein *pst*S
*phn*C	Phosphate-import ATP-binding protein
*phn*D	Phosphate-import
*phn*F	protein for transcriptional regulator
*phn*GHI	Alpha-D-ribose 1-methylphosphonate 5-triphosphate synthase
*phn*J	Alpha-D-ribose 1-methylphosphonate 5-phosphate C-P lyase
*phn*K	Putative phosphonates utilization ATP-binding protein
*phn*L	Alpha-D-ribose 1-methylphosphonate 5-triphosphate diphosphatase
*phn*M	Alpha-D-ribose 1-methylphosphonate 5-triphosphate diphosphatase
*phn*N	Ribose 1,5-bisphosphate phosphokinase
*phn*O	N-acetyltransferase
*phn*P	Phosphoribosyl 1,2-cyclic phosphate phosphodiesterase
*phn*RV	transcriptional regulator of degradation operons for 2-aminoethylphosphonate
*phn*V	transport system permease protein responsible for 2-aminoethylphosphonate

Hydrogen sulfide (H_2_S) has emerged as an essential chemical for phosphate solubilization. H_2_S interacts with ferric phosphate to produce ferrous sulfate and release phosphate. The CPHN2 genome encodes a collection of genes involved in H_2_S production (Table 3), including a cluster of *cys* genes involved in reduction, transportation, protein binding, and acetyl and adenyl group transfer. The CPHN2 genome also has genes related to sulfur metabolism: mineralization and transport of sulfite, sulfate, and phosphoadenosine phosphosulfate.

**Table 3:** *P. agglomerans* strain CPHN2 genomic annotation for sulfate metabolism.

**Gene name**	**Gene product**
*cys*E	Serine acetyltransferase
*cys*J	Sulfite reductase [NADPH] flavoprotein alpha-component
*cys*Q	3′(2′),5′-bisphosphate nucleotidase CysQ
*cys*S	Cysteine–tRNA ligase
*cys*B	HTH-type transcriptional regulator CysB
*cys*G	Siroheme synthase
*cys*L	HTH-type transcriptional regulator CysL
*cys*Z	Sulfate transporter CysZ
*cys*M	Cysteine synthase B
*cys*A	Sulfate/thiosulfate import ATP-binding protein CysA
*cys*W	Sulfate transport system permease protein CysW
*cys*T	Sulfate transport system permease protein CysT
*cys*P	Thiosulfate-binding protein
*cys*C	Adenylyl-sulfate kinase
*cys*N	Sulfate adenylyl transferase subunit 1
*cys*D	Sulfate adenylyl transferase subunit 2
*cys*H	Phosphoadenosine phosphosulfate reductase
*cys*I	Sulfite reductase [NADPH] hemoprotein beta-component
cysJ	Sulfite reductase [NADPH] flavoprotein alpha-component

#### Siderophores

High-affinity iron chelating compounds have been shown to be produced mostly by plant-associated bacterial strains (de Souza et al., 2015) and may assist them by collecting iron from the environment (Niazi et al., 2014). CPHN2 has the ability to synthesize an enterobactin siderophore, and the *ent*ABCEF genes for the same have been annotated. This siderophore is responsible for the recovery of iron through complex formation once it is exported from the cell by *ent*S. *P. agglomerans*. CPHN2 also has many receptors for siderophores, *mbt*H, an efflux pump protein, which helps in transport, and *fhu*ABCDEF, which helps in transport and binding (Figure 6, Table 4, Supplementary Table 4).

**Table 4:** *P. agglomerans* strain CPHN2 genomic annotation for siderophore production.

**Gene name**	**Gene product**
*fhu*A	Ferrichrome outer-membrane transporter/phage receptor
*fhu*B	Iron (3+)-hydroxamate import system permease protein
*fhu*C	Iron (3+)-hydroxamate import ATP-binding protein
*fhu*D	Iron (3+)-hydroxamate-binding protein
*fhu*E	Receptor
*fhu*F	Ferric iron reductase protein
*ent*A	2,3-dihydro-2,3-dihydroxybenzoate dehydrogenase
*ent*BCE	Enterobactin synthase
*ent*S	Exporter
*mbt*H	efflux pump protein that helps in transport

#### Nitrogen metabolism

**Figure 7:**
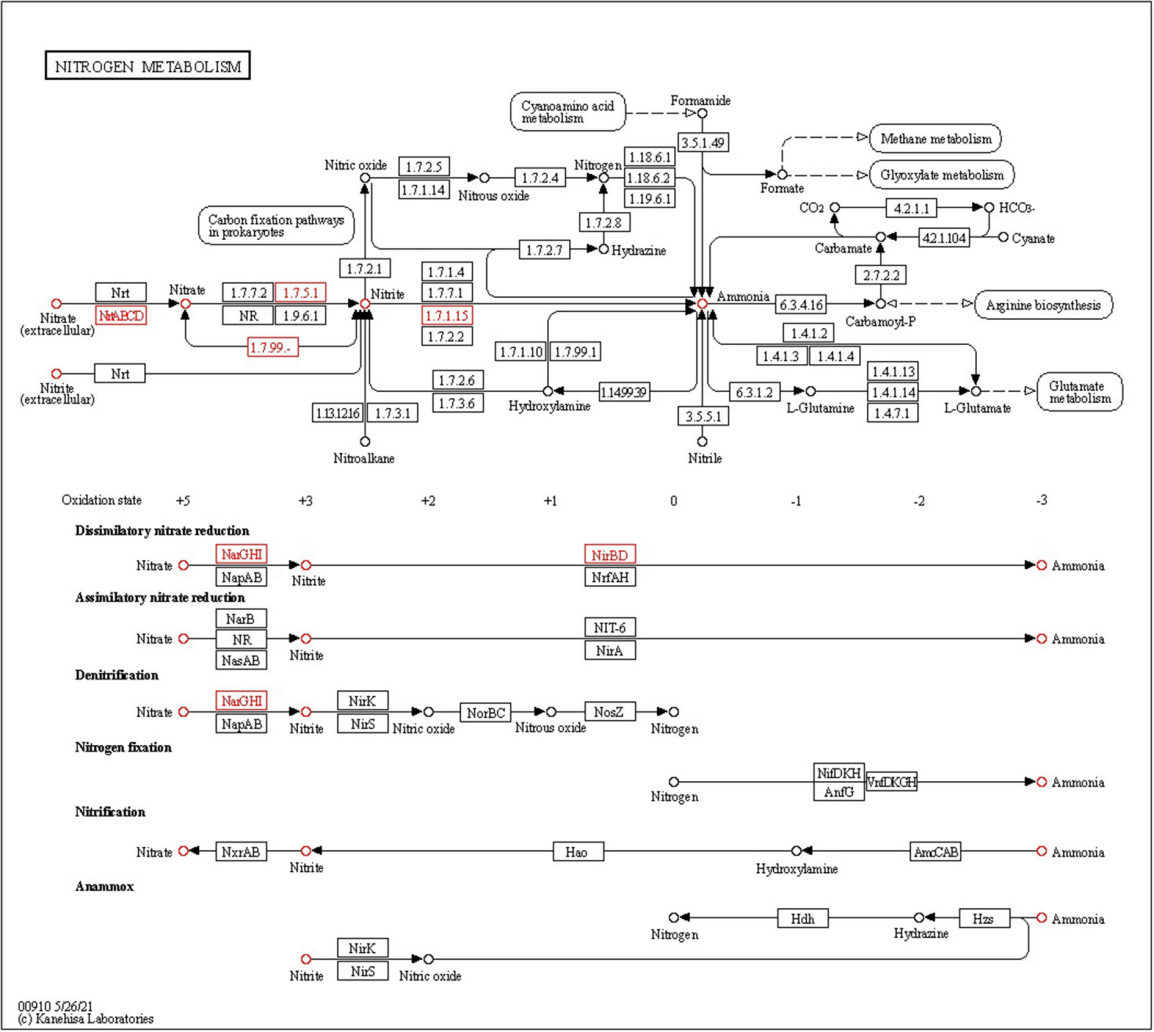
KEGG pathway gene mapping of the strain CPHN2 for nitrogen metabolism.

Ammonia production, another important trait of PGPEB, indirectly promotes plant growth and biomass accumulation. Our genomic study has shown that CPHN2 includes *nar*P, *nar*J, and *nar*L, which converts atmospheric nitrogen to nitrite, as well as *nir*B and *nir*D, nitrite to nitrate, and *nrt*A is responsible for nitrate transportation (Figure 7, Table 5, Supplementary Table 4).

**Table 5:** *P. agglomerans* strain CPHN2 genomic annotation for dissimilatory nitrate reduction.

**Gene name**	**Gene product**
*nar*Q	Nitrate/nitrite sensor protein *nar*Q
*nar*L	Nitrate/nitrite response regulator protein *nar*L
*nar*I	Respiratory nitrate reductase 1 gamma chain
*nar*J	Nitrate reductase molybdenum cofactor assembly chaperone *nar*J
*nar*H	Respiratory nitrate reductase 1 beta chain
*nar*G	Respiratory nitrate reductase 1 alpha chain
*nar*K	Nitrate/nitrite transporter *nark*
*nir*D	Nitrite reductase (NADH) small subunit

#### Indole-3-acetic acid

**Figure 8:**
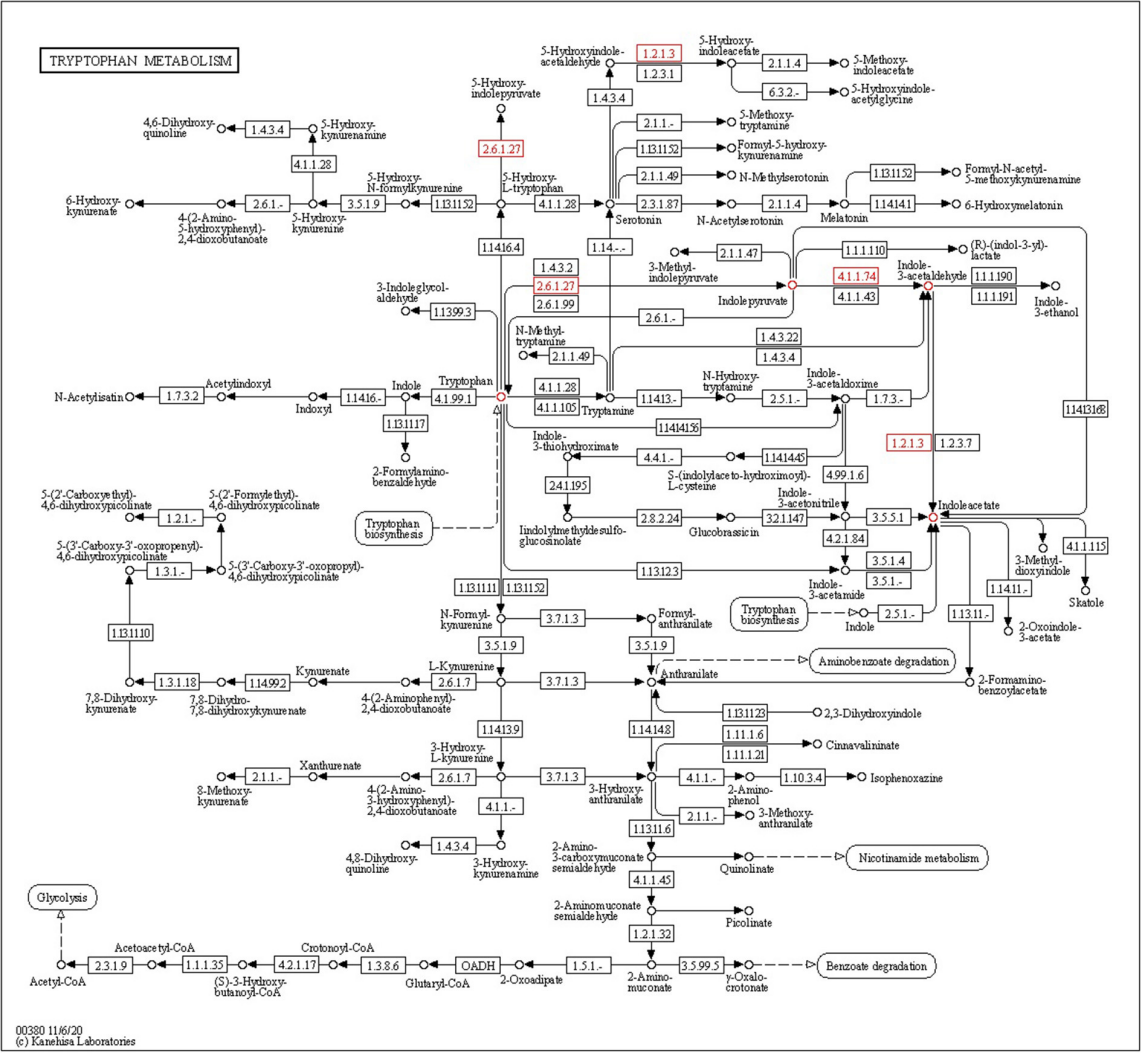
KEGG pathway gene mapping of the strain CPHN2 for indole-3-acetic acid (IAA) synthesis.

Endophytic bacteria produce indole-3-acetic acid (IAA), which is necessary for plant development processes and play an important role in plant–microbe interactions. Four tryptophan-dependent pathways, namely, indole-3-acetamide (IAM), indole-3-pyruvic acid (IPA), tryptamine (TAM), and indole-3-acetaldoxime (IAOx), are involved in the biosynthesis of IAA present in bacteria. The indole-3-pyruvate decarboxylase (*ipd*C) gene is present in CPHN2 and is involved in the conversion of indole-3-pyruvate to IAA in the IPA pathway. The *iaa*M and *iaa*H genes were involved in the synthesis of IAA using the IAM pathway. Of these, only the *iaa*H gene is present in the CPHN2 genome. These data show that IPA is the sole route for the synthesis of IAA using this strain (Figure 8, Supplementary Table 4).

#### Chemotaxis, motility, and attachment of genes

In the whole genome of CPHN2, genes, such as the cluster of *fli* gene (*fli*AEFGHIJMNOPQRSTYZ), *sec* gene (*sec*ABDEFGMY), *mgl* gene (*mgl*ABC), *rbs* gene (*rbs*ABCDKR), *che* gene (*che*ABRVWYZ), *lap*AB, *mot*AB, *lap*AB, *pil*Q, *rfb*X, *opr*BM, *pal, eps*F, and *sct*C, responsible for endophytic behavior, like chemotaxis movement and attachment to the host, have been annotated (Table 6, Supplementary Table 4).

**Table 6:** *P. agglomerans* strain CPHN2 genomic annotation for chemotaxis, motility, and attachment.

**Gene name**	**Gene product**
*fli*A	RNA polymerase sigma factor
*fli*E	Flagellar hook-basal body complex protein
*fli*F	Flagellar M-ring protein
*fli*G	Flagellar motor switch protein
*fli*H	Flagellar assembly protein
*fli*I	Flagellum-specific ATP synthase
*fli*J	Flagellar FliJ protein
*fli*M	Flagellar motor switch protein
*fli*N	Flagellar motor switch protein
*fli*O	Flagellar protein
*fli*P	Flagellar biosynthetic protein
*fli*Q	Flagellar biosynthetic protein
*fli*R	Flagellar biosynthetic protein
*fli*S	Flagellar secretion chaperone
*fli*T	Flagellar protein
*fli*Y	L-cystine-binding protein
*fli*Z	Regulator of sigma S factor
*mgl*A	Galactose/methyl galactoside import ATP-binding protein
*mgl*B	D-galactose-binding periplasmic protein
*mgl*C	Galactoside transport system permease protein
*pil*Q	Type IV pilus biogenesis and competence protein
*rbs*A	Ribose import ATP-binding protein
*rbs*B	Ribose import binding protein
*rbs*C	Ribose import permease protein
*rbs*D	D-ribose pyranase
*rbs*K	Ribokinase
*rbs*R	Ribose operon repressor
*che*A	Chemotaxis protein
*che*B	Protein-glutamate methylesterase/protein-glutamine glutaminase
*che*R	Chemotaxis protein methyltransferase
*che*V	Chemotaxis protein
*che*W	Chemotaxis protein
*che*Y	Chemotaxis protein
*che*Z	Protein phosphatase
*lap*A	Lipopolysaccharide assembly protein A
*lap*B	Lipopolysaccharide assembly protein B
*mot*A	Motility protein A
*mot*B	Motility protein B
*lap*A	Lipopolysaccharide assembly protein A
*lap*B	Lipopolysaccharide assembly protein B
*pil*Q	Type IV pilus biogenesis and competence protein
*rfb*X	Putative O-antigen transporter
*cel*Y	Minor endoglucanase Y
*opr*B	Porin B
*opr*M	Outer membrane protein *opr*M
*sec*A	Protein translocase subunit *sec*A
*sec*B	Protein-export protein s*ec*B
*sec*D	Protein translocase subunit *sec*D
*sec*E	Protein translocase subunit s*ec*E
*sec*F	Protein translocase subunit *sec*F
*sec*G	Protein-export membrane protein *sec*G
*sec*M	Secretion monitor
*sec*Y	Protein translocase subunit s*ec*Y
*sct*C	Type 3 secretion system secretin
*pal*	Peptidoglycan-associated lipoprotein
*eps*F	Type II secretion system protein F

## Discussion

Plant-associated endophytic microbes have evolved unique biosynthetic pathways to aid in their interactions with their host. These different metabolites of endophytic origin promote plant growth; therefore, their genomic studies recently gained substantial interest (Patel et al., 2016; Kandel et al., 2022). In our previous study, we isolated non-rhizobial endophytic bacteria *P. agglomerans* CPHN2, which possessed multiple plant growth-promoting traits such as the production of ammonia, siderophores, and IAA, and phosphate solubilization (Maheshwari et al., 2019) and produced a high amount of IAA under optimized conditions (Unpublished). Genomic mining of their genes might help us develop commercial bioinoculants so as to reduce the use of chemical fertilizers. The presence of these positive traits in *P. agglomerans* CPHN2 prompted whole-genome sequencing and genomic annotations. The genome of *P. agglomerans* CPHN2 was also compared with the already reported genomic sequences of 10 strains to unravel the similarities between the two. A phylogenetic tree constructed based on the pan-genome showed that *P. agglomerans* CPHN2, NCTC10500, and T1 belonged to the same clade (Figure 4). This indicates that these isolates share a large set of similar genes. Core-genome-based phylogenetic tree shows that these two strains NCTC10500 and T1 belong to the same clade but CPHN2 has a separate niche. The closely related FDAARGOS 1447 was present in a separate niche upon the pan-genome analysis and in a clade upon the core-genome analysis.

Genomic mining of CPHN2 supported a previous study on its ability to possess multiple PGP traits and promote plant growth (Maheshwari et al., 2019). Analysis of CPHN2 provided clues to the presence of specific genes for phosphate solubilization, nitrogen metabolism, and the production of siderophores and IAA. PGPR promotes plant growth by solubilizing the phosphate mineral and making it accessible to plants. They employed different solubilization mechanisms, either through the production of extracellular enzymes or by the release of organic acids, protons, and hydroxyl ions. Phosphate-solubilizing bacteria such as *Bacillus, Pseudomonas*, and *Pantoea* are reported to release GA most commonly (Rodríguez et al., 2007). It has also been reported that *Enterobacter asburiae* mutants, which lack glucose dehydrogenase (GDH) activity, were unable to release phosphate (Gyaneshwar et al., 1999). Analysis of the CPHN2 genome revealed the presence of the genes *gcd* and *pqq* responsible for the synthesis of GA and the solubilization of inorganic mineral phosphates.

Microorganisms have also evolved mechanisms to use other sources of phosphorous, such as phosphonates, during phosphate starvation. Enzymes involved in phosphonate catabolic pathways are conserved and encoded by ortholog genes in bacteria. The strain CPHN2 has *phn* genes like *phn*CDFGHIJKLMNOPRV responsible for phosphate solubilization. *phn*CD is a gene for phosphate-import protein, *phn*F is a gene for transcriptional regulation, *phn*GHILMNOP is a gene for the involvement of enzymes in the phosphonate degradation pathway, and *phn*RV is a gene for transcriptional regulation of degrading operons, and *phn*V is a gene for permease protein. The strain CPHN2 also has *pst*ABCS genes, which are responsible for phosphate binding, import, and transportation. Such similar clusters have also been reported for phosphonate binding, transport, and degradation in *Escherichia coli* and *P. agglomerans* strain P5 (Metcalf and Wanner, 1993; Shariati et al., 2017).

Siderophores are low molecular weight compounds, which are produced by a large number of bacterial endophytes to scavenge iron under iron-destitute state. Siderophore production is an important plant growth-promoting trait as it helps the microbes to obtain iron, which in turn is directly transferred to plants and indirectly suppresses the growth of phytopathogens. In addition to this, genes associated with the transport and production of siderophores are also present. *fhu*ABCDEF genes are responsible for binding and transport, *ent*A gene is for 2,3-dihydro-2,3-dihydroxybenzoate dehydrogenase, *ent*BCE gene is for Enterobactin synthase, *ent*S gene is an exporter, and *mbt*H gene is an efflux pump protein that helps in transport. Along with this, we were also able to find out the cytoplasmic membrane and periplasmic proteins involved in the uptake of siderophores (Stevens et al., 1999) and *ton*B-dependent outer-membrane receptors are coded for by the *fhu*DCB operon in Rhizobium (Shariati et al., 2017). In addition to this, CPHN2 is also capable of carrying out dissimilatory nitrate reduction. It is a two-step process in which, during anoxic conditions, nitrate acts as an electron acceptor and is converted to ammonia. The main enzymes involved are nitrate reductase (encoded by *nar*GHIJ) and nitrite reductase (*nir*D).

Several endophytic bacterial genera are known to produce phytohormones, the most common of which is IAA. Multiple pathways have been used by both plants as well as bacteria for the biosynthesis of IAA (Spaepen et al., 2007). There are several pathways in which the amino acid tryptophan is a direct precursor of IAA and modulates the level of IAA synthesis (Zaidi et al., 2009). Among all these Trp-dependent pathways, IAM and IPA pathways are widespread among bacteria (Mano and Nemoto, 2012). The IAM pathway is common among phytopathogens such as *Pseudomonas syringae, Agrobacterium tumefaciens*, and *Agrobacterium rhizogenes* (Szkop and Bielawski, 2013), while most PGPRs such as *Pseudomonas putida, Enterobacter cloacae*, and *Azospirillum brasilense* use the IPA pathway for the biosynthesis of IAA. The IPA pathway has been well-characterized in bacteria, and the conversion of indole-3-pyruvate to indole-3-acetaldehyde by *ipd*C is the rate-limiting step (Zhao, 2010). The expression of the key gene *ipd*C has been found to increase in *P. agglomerans* while growing on the surfaces of plants, depicting an interesting case of interaction between plants and microbes (Jasim et al., 2014). This study also aimed to identify the complete IAA biosynthetic pathways in CPHN2. Genomic mining revealed the presence of all the genes, including the involvement of the key gene *ipd*C in the IPA pathway. A previous study on the genomic study of *P. agglomerans* strain P5 revealed that IAM was the only pathway for the production of IAA (Shariati et al., 2017). Meanwhile, our CPHN2 has an incomplete IAM pathway in which the *iaa*H gene is present.

Endophytic colonization is a conglomerate of processes such as entry to the host plant, growth on to the plant, and subsequent multiplication there. Root exudates are various chemicals, ranging from organic acids and amino acids to sugars, which help in the recruitment of bacterial endophytes from a pool of rhizospheric bacteria. According to our annotation, CPHN2 contains the cluster of FLI proteins, which are responsible for the production of the flagellar protein and motor system. It also has a cluster of *che, mgl*, and *rbs* genes, responsible for galactose and ribose binding, transport, and chemotaxis, as previously reported (Harayama et al., 1983; Barroga et al., 1996). In addition, the Sec gene cluster responsible for protein translocation, export, and monitoring was also found to be present.

The *lip, pil, rfb, cel, opr, sct*, and *pal* genes were also annotated in CPHN2. Previous studies reported the role of these genes in chemotaxis, motility, adhesion, pilli formation, dehydratase synthesis, and porin creation in the outer membrane and in the formation of exopolysaccharides (EPSs). Recent studies demonstrated that endophytic bacteria frequently secrete EPSs for their adherence to the root surface (Dudeja et al., 2021). EPS also decreases the concentration of free radicals in the plant cell wall and protects cells against oxidative stress. CPHN2 also carries the gene for the production of EPS.

As members of the genus *Pantoea* were previously recognized as phytopathogens causing wilting and necrosis, we also analyzed the presence of the type III secretion system (T3SSs) coded by the *hrc/hrp* gene cluster responsible for pathogenicity. A large number of Gram-negative bacteria use this secretion system to convey effector molecules directly to the cytosol of their host. Several studies reported the role of the whole *hrc/hrp* gene cluster in pathogenicity and hypersensitive response in plants (Buonaurio et al., 2015; Shariati et al., 2017). However, CPHN2 does not contain the complete gene cluster and lacks pathogenicity. The study also revealed the occurrence of only one pathway, i.e., IPA pathway, associated with useful bacteria for the production of IAA, out of the five pathways reported in bacteria. Therefore, this study provides in-depth information about genomic pathways and helps in understanding the mechanisms of PGP by endophytic bacteria.

## Conclusion

Genomic analysis of *P. agglomerans* CPHN2 revealed the presence of several genes engaged in PGPs, such as phosphate solubilization and nitrate reduction, and in the production of siderophores and IAA. Moreover, it has genes related to chemotaxis, motility, adhesion, and pilli formation, which are required for effective colonization. It lacks the complete pathogenic gene cluster. This research provides insights into the plant growth-promoting ability of CPHN2 and highlights its potential as a biofertilizer. Therefore, this strain is deemed safe and can be further explored as a bioinoculant. However, more research is needed to provide a detailed interpretation of the functions and the regulation of these genes.

## Data availability statement

The whole genome of *P. agglomerans* CPHN2 has been deposited in GenBank under Accession nos. CP098412, CP098413 for plasmids, and CP098414 for genome and is annotated by a prokaryotic genomic annotation pipeline (PGAP) (Kumar et al., 2022) (Tatusova et al., 2016; Kumar et al., 2022). Bio Project accession number PRJNA811747 (Sequence Read Archive Accession No. SRR18189611) (https://www.ncbi.nlm.nih.gov/sra/SRR18189611).

## Author contributions

PK: data curation, investigation, methodology, software, validation, visualization, and writing–original draft. SR and PD: formal analysis, writing, and software analysis. AK, AD, and PS: conceptualization, funding acquisition, supervision, validation, and writing–review and editing. All authors contributed to the article and approved the submitted version.

## Conflict of interest

The authors declare that the research was conducted in the absence of any commercial or financial relationships that could be construed as a potential conflict of interest.

## Publisher's note

All claims expressed in this article are solely those of the authors and do not necessarily represent those of their affiliated organizations, or those of the publisher, the editors and the reviewers. Any product that may be evaluated in this article, or claim that may be made by its manufacturer, is not guaranteed or endorsed by the publisher.

## References

[B1] Agri U. Chaudhary P. Sharma A. (2021). *In vitro* compatibility evaluation of agriusable nanochitosan on beneficial plant growth-promoting rhizobacteria and maize plant. Natl. Acad. Sci. Lett. 44, 555–559. 10.1007/s40009-021-01047-w

[B2] Agri U. Chaudhary P. Sharma A. Kukreti B. (2022). Physiological response of maize plants and its rhizospheric microbiome under the influence of potential bioinoculants and nanochitosan. Plant Soil 474, 451–468. 10.1007/s11104-022-05351-2

[B3] Ahemad M. Kibret M. (2014). Mechanisms and applications of plant growth promoting rhizobacteria: current perspective. J. King Saud Univ. Sci. 26, 1–20. 10.1016/j.jksus.2013.05.001

[B4] Alikhan N. F. Petty N. K. Ben Zakour N. L. Beatson S. A. (2011). BLAST ring image generator (BRIG): simple prokaryote genome comparisons. BMC Genomics 12, 1–10. 10.1186/1471-2164-12-402 21824423 PMC3163573

[B5] Andrews S. (2010). Data from: FastQC: A Quality Control Tool for High Throughput Sequence Data. Available online at: https://scholar.google.com/scholar?hl=en&as_sdt=0%2C5&q=17.+Andrews+S.+2010.+FastQC%3A+a+quality+control+tool+for+high+throughput+sequence+data.+http%3A%2F%2F (accessed April 4, 2022).

[B6] Barroga C. F. Zhang H. Wajih N. Bouyer J. H. Hermodson M. A. (1996). The proteins encoded by the rbs operon of Escherichia coli: I. Overproduction, purification, characterization, and functional analysis of RbsA. Protein Sci. 5, 1093–1099. 10.1002/PRO.5560050611 8762140 PMC2143435

[B7] Buonaurio R. Moretti C. Da Silva D. P. Cortese C. Ramos C. Venturi V. (2015). The olive knot disease as a model to study the role of interspecies bacterial communities in plant disease. Front. Plant Sci. 6, 434. 10.3389/fpls.2015.00434 26113855 PMC4461811

[B8] Chaudhari N. M. Gupta V. K. Dutta C. (2016). BPGA- an ultra-fast pan-genome analysis pipeline. Sci. Rep. 6, 1–10. 10.1038/srep24373 27071527 PMC4829868

[B9] Chen S. Zhou Y. Chen Y. Gu J. (2018). fastp: an ultra-fast all-in-one FASTQ preprocessor. Bioinformatics 34, i884–i890. 10.1093/bioinformatics/bty560 30423086 PMC6129281

[B10] Chi H. Liu C. Yang H. Zeng W. F. Wu L. Zhou W. J.. (2018). Comprehensive identification of peptides in tandem mass spectra using an efficient open search engine. Nat. Biotechnol. 36, 1059–1061. 10.1038/nbt.4236 30295672

[B11] Darling A. C. E. Mau B. Blattner F. R. Perna N. T. (2004). Mauve: multiple alignment of conserved genomic sequence with rearrangements. Genome Res. 14, 1394. 10.1101/gr.2289704 15231754 PMC442156

[B12] de Souza R. Ambrosini A. Passaglia L. M. P. (2015). Plant growth-promoting bacteria as inoculants in agricultural soils. Genet. Mol. Biol. 38, 401–419. 10.1590/S1415-475738420150053 26537605 PMC4763327

[B13] Duca D. Lorv J. Patten C. L. Rose D. Glick B. R. (2014). Indole-3-acetic acid in plant-microbe interactions. Antonie Van Leeuwenhoek. 106, 85–125. 10.1007/s10482-013-0095-y 24445491

[B14] Duca D. R. Glick B. R. (2020). Indole-3-acetic acid biosynthesis and its regulation in plant-associated bacteria. Appl. Microbiol. Biotechnol. 104, 8607–8619. 10.1007/s00253-020-10869-5 32875364

[B15] Dudeja S. S. Suneja-Madan P. Paul M. Maheswari R. Kothe E. (2021). Bacterial endophytes: molecular interactions with their hosts. J. Basic Microbiol. 61, 475–505. 10.1002/jobm.202000657 33834549

[B16] Dutkiewicz J. Mackiewicz B. Lemieszek M. K. Golec M. Milanowski J. (2016). Pantoea agglomerans: a mysterious bacterium of evil and good. Part IV. Beneficial effects. Ann. Agric. Environ. Med. 23, 206–222. 10.5604/12321966.1203879 27294621

[B17] Eastman A. W. Heinrichs D. E. Yuan Z. C. (2014). Comparative and genetic analysis of the four sequenced *Paenibacillus polymyxa* genomes reveals a diverse metabolism and conservation of genes relevant to plant-growth promotion and competitiveness. BMC Genomics 15, 1–22. 10.1186/1471-2164-15-851 25280501 PMC4209062

[B18] Glick B. R. (2012). Plant growth-promoting bacteria: mechanisms and applications. Scientifica 2012, 1–15. 10.6064/2012/963401 24278762 PMC3820493

[B19] Gyaneshwar P. Parekh L. J. Archana G. Poole P. S. Collins M. D. Hutson R. A.. (1999). Involvement of a phosphate starvation inducible glucose dehydrogenase in soil phosphate solubilization by *Enterobacter asburiae*. FEMS Microbiol. Lett. 171, 223–229. 10.1111/j.1574-6968.1999.tb13436.x

[B20] Harayama S. Bollinger J. Iino T. Hazelbauer G. L. (1983). Characterization of the mgl operon of *Escherichia coli* by transposon mutagenesis and molecular cloning. J. Bacteriol. 153, 408–415. 10.1128/jb.153.1.408-415.1983 6294056 PMC217387

[B21] Jasim B. Jimtha John C. Shimil V. Jyothis M. Radhakrishnan E. K. (2014). Studies on the factors modulating indole-3-acetic acid production in endophytic bacterial isolates from Piper nigrum and molecular analysis of ipdc gene. J. Appl. Microbiol. 117, 786–799. 10.1111/jam.12569 24916921

[B22] Kandel P. P. Naumova M. Fautt C. Patel R. R. Triplett L. R. Hockett K. L. (2022). Genome mining shows ubiquitous presence and extensive diversity of toxin-antitoxin systems in pseudomonas syringae. Front. Microbiol. 12, 815911. 10.3389/fmicb.2021.815911 35095819 PMC8790059

[B23] Ke M. Shen H. Wang L. Luo S. Lin L. Yang J.. (2016). Identification, quantification, and site localization of protein posttranslational modifications via mass spectrometry-based proteomics. Adv. Exp. Med. Biol. 919, 345–382. 10.1007/978-3-319-41448-5_17 27975226

[B24] Kukreti B. Sharma A. Chaudhary P. Agri U. Maithani D. (2020). Influence of nanosilicon dioxide along with bioinoculants on Zea mays and its rhizospheric soil. 3 Biotech 10, 345. 10.1007/s13205-020-02329-8 32728512 PMC7374527

[B25] Kumar P. Chauhan V. Dang A. S. Kumar A. Suneja P. (2022). Draft genome sequence of pantoea agglomerans CPHN2, a potential plant-growth-promoting endophyte. Microbiol. Resour. Announc. 11, e0019222. 10.1128/mra.00192-22 35861538 PMC9387234

[B26] Lee J. E. Lee B. J. Chung J. O. Hwang J. A. Lee S. J. Lee C. H.. (2010). Geographical and climatic dependencies of green tea (*Camellia sinensis*) metabolites: A 1H NMR-based metabolomics study. J. Agric. Food Chem. 58, 10582–10589. 10.1021/jf102415m 20828156

[B27] Liu W. Wang Q. Hou J. Tu C. Luo Y. Christie P. (2016). Whole genome analysis of halotolerant and alkalotolerant plant growth-promoting rhizobacterium Klebsiella sp. D5A. Sci. Rep. 6, 26710. 10.1038/srep26710 27216548 PMC4877636

[B28] Maheshwari R. Bhutani N. Bhardwaj A. Suneja P. (2019). Functional diversity of cultivable endophytes from *Cicer arietinum* and *Pisum sativum*: bioprospecting their plant growth potential. Biocatal. Agric. Biotechnol. 20, 101229. 10.1016/j.bcab.2019.101229

[B29] Mano Y. Nemoto K. (2012). The pathway of auxin biosynthesis in plants. J. Exp. Bot. 63, 2853–2872. 10.1093/jxb/ers091 22447967

[B30] Metcalf W. W. Wanner B. L. (1993). Evidence for a fourteen-gene, phnC to phnP locus for phosphonate metabolism in *Escherichia coli*. Gene 129, 27–32. 10.1016/0378-1119(93)90692-V 8335257

[B31] Navarro-Torre S. Barcia-Piedras J. M. Mateos-Naranjo E. Redondo-Gómez S. Camacho M. Caviedes M. A.. (2017). Assessing the role of endophytic bacteria in the halophyte Arthrocnemum macrostachyum salt tolerance. Plant Biol. 19, 249–256. 10.1111/plb.12521 27770586

[B32] Niazi A. Manzoor S. Asari S. Bejai S. Meijer J. Bongcam-Rudloff E. (2014). Genome analysis of Bacillus amyloliquefaciens Subsp. plantarum UCMB5113: a rhizobacterium that improves plant growth and stress management. PLoS ONE 9, e104651. 10.1371/journal.pone.0104651 25119988 PMC4138018

[B33] Parker G. F. Higgins T. P. Hawkes T. Robson R. L. (1999). Rhizobium (Sinorhizobium) meliloti phn genes: characterization and identification of their protein products. J. Bacteriol. 181, 389–395. 10.1128/JB.181.2.389-395.1999 9882650 PMC93390

[B34] Patel R. R. Thakkar V. R. Subramanian R. B. (2016). Simultaneous detection and quantification of phytohormones by a sensitive method of separation in culture of *Pseudomonas sp*. Curr. Microbiol. 72, 744–751. 10.1007/s00284-016-1012-1 26905268

[B35] Pinski A. Zur J. Hasterok R. Hupert-Kocurek K. (2020). Comparative genomics of *Stenotrophomonas maltophili*a and *Stenotrophomonas rhizophila* revealed characteristic features of both species. Int. J. Mol. Sci. 21, 4922. 10.3390/ijms21144922 32664682 PMC7404187

[B36] Ramachandran S. Fontanille P. Pandey A. Larroche C. (2006). Gluconic Acid: Properties, Applications and Microbial Production. *Food Technol. Biotechnol*. 44. Available online at: https://www.researchgate.net/publication/228342155_Gluconic_Acid_Properties_Applications_and_Microbial_Production (accessed April 5, 2022).

[B37] Rani S. Kumar P. Suneja P. (2021). Biotechnological interventions for inducing abiotic stress tolerance in crops. Plant Gene 27, 100315. 10.1016/j.plgene.2021.100315

[B38] Rodríguez H. Fraga R. Gonzalez T. Bashan Y. (2007). First International Meeting on Microbial Phosphate Solubilization. Available online at: https://link.springer.com/book/10.1007/978-1-4020-5765-6 (accessed April 4, 2022).

[B39] Seemann T. (2017). Shovill: faster SPAdes assembly of Illumina reads (v0. 9.0). Available online at: https://github.com/tseemann/shovill

[B40] Shariati V. J. Malboobi M. A. Tabrizi Z. Tavakol E. Owilia P. Safari M. (2017). Comprehensive genomic analysis of a plant growth-promoting rhizobacterium Pantoea agglomerans strain P5. Sci. Rep. 7, 15610. 10.1038/s41598-017-15820-9 29142289 PMC5688152

[B41] Spaepen S. Vanderleyden J. Remans R. (2007). Indole-3-acetic acid in microbial and microorganism-plant signaling. FEMS Microbiol. Rev. 31, 425–448. 10.1111/j.1574-6976.2007.00072.x 17509086

[B42] Stevens J. B. Carter R. A. Hussain H. Carson K. C. Dilworth M. J. Johnston A. W. B. (1999). The fhu genes of Rhizobium leguminosarum, specifying siderophore uptake proteins: FhuDCB are adjacent to a pseudogene version of fhuA. Microbiology 145, 593–601. 10.1099/13500872-145-3-593 10217493

[B43] Szkop M. Bielawski W. (2013). TyrB-2 and phhC genes of *Pseudomonas putida* encode aromatic amino acid aminotransferase isozymes: evidence at the protein level. Amino Acids 45, 351–358. 10.1007/s00726-013-1508-y 23685963 PMC3714555

[B44] Tatusova T. Dicuccio M. Badretdin A. Chetvernin V. Nawrocki E. P. Zaslavsky L.. (2016). NCBI prokaryotic genome annotation pipeline. Nucleic Acids Res. 44, 6614–6624. 10.1093/nar/gkw569 27342282 PMC5001611

[B45] Verheggen K. Martens L. Berven F. S. Barsnes H. Vaudel M. (2016). Database search engines: paradigms, challenges and solutions. Adv. Exp. Med. Biol. 919, 147–156. 10.1007/978-3-319-41448-5_6 27975215

[B46] Walterson A. M. Stavrinides J. (2015). Pantoea: insights into a highly versatile and diverse genus within the Enterobacteriaceae. FEMS Microbiol. Rev. 39, 968–984. 10.1093/femsre/fuv027 26109597

[B47] Xie J. Shi H. Du Z. Wang T. Liu X. Chen S. (2016). Comparative genomic and functional analysis reveal conservation of plant growth promoting traits in *Paenibacillus polymyxa* and its closely related species. Sci. Rep. 6, 21329. 10.1038/srep21329 26856413 PMC4746698

[B48] Zaidi A. Khan M. S. Ahemad M. Oves M. (2009). Plant growth promotion by phosphate solubilizing bacteria. Acta Microbiol. Immunol. Hung. 56, 263–284. 10.1556/AMicr.56.2009.3.6 19789141

[B49] Zhao Y. (2010). Auxin biosynthesis and its role in plant development. Annu. Rev. Plant Biol. 61, 49–64. 10.1146/annurev-arplant-042809-112308 20192736 PMC3070418

