# Supplementary material for: Whole genome analysis for plant growth promotion profiling of Pantoea agglomerans CPHN2, a non-rhizobial nodule endophyte
Source: Front Microbiol. 2022 Nov 7;13:998821. doi: 10.3389/fmicb.2022.998821 (PMC9676466; doi:10.3389/fmicb.2022.998821)
Supplement: Supplementary file 1 [file Data_Sheet_1.DOCX]

Table S1: Details of genome data of *P. agglomerans* strain CPHN 2

| **Sample Name** | **PE Reads** | **Total No. of Bases** | **Data in Gb** |
| --- | --- | --- | --- |
| **CPHN2** | 9,731,611 | 2,898,434,450 bp | 7 |

Table S2: Rapid Annotation using Subsystem Technology report of *P. agglomerans* CPHN2

| **Genome** | *Pantoea agglomerans* CPHN2 (Taxonomy ID: [549](http://www.ncbi.nlm.nih.gov/Taxonomy/Browser/wwwtax.cgi?mode=Info&id=549&lvl=3&lin=f&keep=1&srchmode=1&unlock)) |
| --- | --- |
| **Domain** | Bacteria |
| **Taxonomy** | Bacteria; *Proteobacteria; Gammaproteobacteria; Enterobacterales; Erwiniaceae; Pantoea; Pantoea agglomerans group; Pantoea agglomerans*CPHN2 |
| **Size (bp)** | 4,839,757 |
| **GC Content (%)** | 55.2 |
| **N50** | 558390 |
| **L50** | 2 |
| **Number of Contigs (with PEGs)** | 32 |
| **Number of Subsystems** | 529 |
| **Number of Coding Sequences** | 4424 |
| **Number of RNAs** | 84 |

Table S3: *P. agglomerans* CPHN2 strain-specific genes

| Cofactors, Vitamins, Prosthetic Groups, Pigments | Biotin | [Biotin synthesis cluster](https://rast.nmpdr.org/seedviewer.cgi?page=Subsystems&subsystem=Biotin_synthesis_cluster&organism=549.408) | [Competence protein F homolog, phosphoribosyltransferase domain](https://rast.nmpdr.org/seedviewer.cgi?page=FunctionalRole&role=Competence%20protein%20F%20homolog,%20phosphoribosyltransferase%20domain&subsystem_name=Biotin_synthesis_cluster) |
| --- | --- | --- | --- |
| Cofactors, Vitamins, Prosthetic Groups, Pigments | Biotin | [Biotin synthesis cluster](https://rast.nmpdr.org/seedviewer.cgi?page=Subsystems&subsystem=Biotin_synthesis_cluster&organism=549.408) | [Biotin operon repressor](https://rast.nmpdr.org/seedviewer.cgi?page=FunctionalRole&role=Biotin%20operon%20repressor&subsystem_name=Biotin_synthesis_cluster) |
| Cofactors, Vitamins, Prosthetic Groups, Pigments | Biotin | [Biotin synthesis cluster](https://rast.nmpdr.org/seedviewer.cgi?page=Subsystems&subsystem=Biotin_synthesis_cluster&organism=549.408) | [Adenosylmethionine-8-amino-7-oxononanoate aminotransferase (EC 2.6.1.62)](https://rast.nmpdr.org/seedviewer.cgi?page=FunctionalRole&role=Adenosylmethionine-8-amino-7-oxononanoate%20aminotransferase%20(EC%202.6.1.62)&subsystem_name=Biotin_synthesis_cluster) |
| Cofactors, Vitamins, Prosthetic Groups, Pigments | Biotin | [Biotin synthesis cluster](https://rast.nmpdr.org/seedviewer.cgi?page=Subsystems&subsystem=Biotin_synthesis_cluster&organism=549.408) | [8-amino-7-oxononanoate synthase (EC 2.3.1.47)](https://rast.nmpdr.org/seedviewer.cgi?page=FunctionalRole&role=8-amino-7-oxononanoate%20synthase%20(EC%202.3.1.47)&subsystem_name=Biotin_synthesis_cluster) |
| Cofactors, Vitamins, Prosthetic Groups, Pigments | Biotin | [Biotin synthesis cluster](https://rast.nmpdr.org/seedviewer.cgi?page=Subsystems&subsystem=Biotin_synthesis_cluster&organism=549.408) | [Dethiobiotin synthetase (EC 6.3.3.3)](https://rast.nmpdr.org/seedviewer.cgi?page=FunctionalRole&role=Dethiobiotin%20synthetase%20(EC%206.3.3.3)&subsystem_name=Biotin_synthesis_cluster) |
| Cofactors, Vitamins, Prosthetic Groups, Pigments | Biotin | [Biotin synthesis cluster](https://rast.nmpdr.org/seedviewer.cgi?page=Subsystems&subsystem=Biotin_synthesis_cluster&organism=549.408) | [Biotin synthesis protein BioH](https://rast.nmpdr.org/seedviewer.cgi?page=FunctionalRole&role=Biotin%20synthesis%20protein%20BioH&subsystem_name=Biotin_synthesis_cluster) |
| Cofactors, Vitamins, Prosthetic Groups, Pigments | Biotin | [Biotin synthesis cluster](https://rast.nmpdr.org/seedviewer.cgi?page=Subsystems&subsystem=Biotin_synthesis_cluster&organism=549.408) | [Long-chain-fatty-acid--CoA ligase (EC 6.2.1.3)](https://rast.nmpdr.org/seedviewer.cgi?page=FunctionalRole&role=Long-chain-fatty-acid--CoA%20ligase%20(EC%206.2.1.3)&subsystem_name=Biotin_synthesis_cluster) |
| Cofactors, Vitamins, Prosthetic Groups, Pigments | Biotin | [Biotin synthesis cluster](https://rast.nmpdr.org/seedviewer.cgi?page=Subsystems&subsystem=Biotin_synthesis_cluster&organism=549.408) | [Biotin synthase (EC 2.8.1.6)](https://rast.nmpdr.org/seedviewer.cgi?page=FunctionalRole&role=Biotin%20synthase%20(EC%202.8.1.6)&subsystem_name=Biotin_synthesis_cluster) |
| Cofactors, Vitamins, Prosthetic Groups, Pigments | Biotin | [Biotin synthesis cluster](https://rast.nmpdr.org/seedviewer.cgi?page=Subsystems&subsystem=Biotin_synthesis_cluster&organism=549.408) | [Biotin synthesis protein BioC](https://rast.nmpdr.org/seedviewer.cgi?page=FunctionalRole&role=Biotin%20synthesis%20protein%20BioC&subsystem_name=Biotin_synthesis_cluster) |
| Cofactors, Vitamins, Prosthetic Groups, Pigments | Biotin | [Biotin synthesis cluster](https://rast.nmpdr.org/seedviewer.cgi?page=Subsystems&subsystem=Biotin_synthesis_cluster&organism=549.408) | [3-ketoacyl-CoA thiolase (EC 2.3.1.16)](https://rast.nmpdr.org/seedviewer.cgi?page=FunctionalRole&role=3-ketoacyl-CoA%20thiolase%20(EC%202.3.1.16)&subsystem_name=Biotin_synthesis_cluster) |
| Cofactors, Vitamins, Prosthetic Groups, Pigments | Biotin | [Biotin synthesis cluster](https://rast.nmpdr.org/seedviewer.cgi?page=Subsystems&subsystem=Biotin_synthesis_cluster&organism=549.408) | [tRNA (cytidine(34)-2'-O)-methyltransferase (EC 2.1.1.207)](https://rast.nmpdr.org/seedviewer.cgi?page=FunctionalRole&role=tRNA%20(cytidine(34)-2) |
| Cofactors, Vitamins, Prosthetic Groups, Pigments | Biotin | [Biotin biosynthesis](https://rast.nmpdr.org/seedviewer.cgi?page=Subsystems&subsystem=Biotin_biosynthesis&organism=549.408) | [Biotin operon repressor](https://rast.nmpdr.org/seedviewer.cgi?page=FunctionalRole&role=Biotin%20operon%20repressor&subsystem_name=Biotin_biosynthesis) |
| Cofactors, Vitamins, Prosthetic Groups, Pigments | Biotin | [Biotin biosynthesis](https://rast.nmpdr.org/seedviewer.cgi?page=Subsystems&subsystem=Biotin_biosynthesis&organism=549.408) | [Adenosylmethionine-8-amino-7-oxononanoate aminotransferase (EC 2.6.1.62)](https://rast.nmpdr.org/seedviewer.cgi?page=FunctionalRole&role=Adenosylmethionine-8-amino-7-oxononanoate%20aminotransferase%20(EC%202.6.1.62)&subsystem_name=Biotin_biosynthesis) |
| Cofactors, Vitamins, Prosthetic Groups, Pigments | Biotin | [Biotin biosynthesis](https://rast.nmpdr.org/seedviewer.cgi?page=Subsystems&subsystem=Biotin_biosynthesis&organism=549.408) | [8-amino-7-oxononanoate synthase (EC 2.3.1.47)](https://rast.nmpdr.org/seedviewer.cgi?page=FunctionalRole&role=8-amino-7-oxononanoate%20synthase%20(EC%202.3.1.47)&subsystem_name=Biotin_biosynthesis) |
| Cofactors, Vitamins, Prosthetic Groups, Pigments | Biotin | [Biotin biosynthesis](https://rast.nmpdr.org/seedviewer.cgi?page=Subsystems&subsystem=Biotin_biosynthesis&organism=549.408) | [Biotin synthesis protein BioH](https://rast.nmpdr.org/seedviewer.cgi?page=FunctionalRole&role=Biotin%20synthesis%20protein%20BioH&subsystem_name=Biotin_biosynthesis) |
| Cofactors, Vitamins, Prosthetic Groups, Pigments | Biotin | [Biotin biosynthesis](https://rast.nmpdr.org/seedviewer.cgi?page=Subsystems&subsystem=Biotin_biosynthesis&organism=549.408) | [Dethiobiotin synthetase (EC 6.3.3.3)](https://rast.nmpdr.org/seedviewer.cgi?page=FunctionalRole&role=Dethiobiotin%20synthetase%20(EC%206.3.3.3)&subsystem_name=Biotin_biosynthesis) |
| Cofactors, Vitamins, Prosthetic Groups, Pigments | Biotin | [Biotin biosynthesis](https://rast.nmpdr.org/seedviewer.cgi?page=Subsystems&subsystem=Biotin_biosynthesis&organism=549.408) | [Long-chain-fatty-acid--CoA ligase (EC 6.2.1.3)](https://rast.nmpdr.org/seedviewer.cgi?page=FunctionalRole&role=Long-chain-fatty-acid--CoA%20ligase%20(EC%206.2.1.3)&subsystem_name=Biotin_biosynthesis) |
| Cofactors, Vitamins, Prosthetic Groups, Pigments | Biotin | [Biotin biosynthesis](https://rast.nmpdr.org/seedviewer.cgi?page=Subsystems&subsystem=Biotin_biosynthesis&organism=549.408) | [Biotin synthase (EC 2.8.1.6)](https://rast.nmpdr.org/seedviewer.cgi?page=FunctionalRole&role=Biotin%20synthase%20(EC%202.8.1.6)&subsystem_name=Biotin_biosynthesis) |
| Cofactors, Vitamins, Prosthetic Groups, Pigments | Biotin | [Biotin biosynthesis](https://rast.nmpdr.org/seedviewer.cgi?page=Subsystems&subsystem=Biotin_biosynthesis&organism=549.408) | [Biotin synthesis protein BioC](https://rast.nmpdr.org/seedviewer.cgi?page=FunctionalRole&role=Biotin%20synthesis%20protein%20BioC&subsystem_name=Biotin_biosynthesis) |
| Cofactors, Vitamins, Prosthetic Groups, Pigments | Biotin | [Biotin biosynthesis](https://rast.nmpdr.org/seedviewer.cgi?page=Subsystems&subsystem=Biotin_biosynthesis&organism=549.408) | [3-ketoacyl-CoA thiolase (EC 2.3.1.16)](https://rast.nmpdr.org/seedviewer.cgi?page=FunctionalRole&role=3-ketoacyl-CoA%20thiolase%20(EC%202.3.1.16)&subsystem_name=Biotin_biosynthesis) |
| Cofactors, Vitamins, Prosthetic Groups, Pigments | Biotin | [Biotin biosynthesis Experimental](https://rast.nmpdr.org/seedviewer.cgi?page=Subsystems&subsystem=Biotin_biosynthesis_Experimental&organism=549.408) | [Competence protein F homolog, phosphoribosyltransferase domain](https://rast.nmpdr.org/seedviewer.cgi?page=FunctionalRole&role=Competence%20protein%20F%20homolog,%20phosphoribosyltransferase%20domain&subsystem_name=Biotin_biosynthesis_Experimental) |
| Cofactors, Vitamins, Prosthetic Groups, Pigments | Biotin | [Biotin biosynthesis Experimental](https://rast.nmpdr.org/seedviewer.cgi?page=Subsystems&subsystem=Biotin_biosynthesis_Experimental&organism=549.408) | [NfuA Fe-S protein maturation](https://rast.nmpdr.org/seedviewer.cgi?page=FunctionalRole&role=NfuA%20Fe-S%20protein%20maturation&subsystem_name=Biotin_biosynthesis_Experimental) |
| Cofactors, Vitamins, Prosthetic Groups, Pigments | Biotin | [Biotin biosynthesis Experimental](https://rast.nmpdr.org/seedviewer.cgi?page=Subsystems&subsystem=Biotin_biosynthesis_Experimental&organism=549.408) | [Adenosylmethionine-8-amino-7-oxononanoate aminotransferase (EC 2.6.1.62)](https://rast.nmpdr.org/seedviewer.cgi?page=FunctionalRole&role=Adenosylmethionine-8-amino-7-oxononanoate%20aminotransferase%20(EC%202.6.1.62)&subsystem_name=Biotin_biosynthesis_Experimental) |
| Cofactors, Vitamins, Prosthetic Groups, Pigments | Biotin | [Biotin biosynthesis Experimental](https://rast.nmpdr.org/seedviewer.cgi?page=Subsystems&subsystem=Biotin_biosynthesis_Experimental&organism=549.408) | [8-amino-7-oxononanoate synthase (EC 2.3.1.47)](https://rast.nmpdr.org/seedviewer.cgi?page=FunctionalRole&role=8-amino-7-oxononanoate%20synthase%20(EC%202.3.1.47)&subsystem_name=Biotin_biosynthesis_Experimental) |
| Cofactors, Vitamins, Prosthetic Groups, Pigments | Biotin | [Biotin biosynthesis Experimental](https://rast.nmpdr.org/seedviewer.cgi?page=Subsystems&subsystem=Biotin_biosynthesis_Experimental&organism=549.408) | [Biotin synthesis protein BioH](https://rast.nmpdr.org/seedviewer.cgi?page=FunctionalRole&role=Biotin%20synthesis%20protein%20BioH&subsystem_name=Biotin_biosynthesis_Experimental) |
| Cofactors, Vitamins, Prosthetic Groups, Pigments | Biotin | [Biotin biosynthesis Experimental](https://rast.nmpdr.org/seedviewer.cgi?page=Subsystems&subsystem=Biotin_biosynthesis_Experimental&organism=549.408) | [Dethiobiotin synthetase (EC 6.3.3.3)](https://rast.nmpdr.org/seedviewer.cgi?page=FunctionalRole&role=Dethiobiotin%20synthetase%20(EC%206.3.3.3)&subsystem_name=Biotin_biosynthesis_Experimental) |
| Cofactors, Vitamins, Prosthetic Groups, Pigments | Biotin | [Biotin biosynthesis Experimental](https://rast.nmpdr.org/seedviewer.cgi?page=Subsystems&subsystem=Biotin_biosynthesis_Experimental&organism=549.408) | [Biotin synthase (EC 2.8.1.6)](https://rast.nmpdr.org/seedviewer.cgi?page=FunctionalRole&role=Biotin%20synthase%20(EC%202.8.1.6)&subsystem_name=Biotin_biosynthesis_Experimental) |
| Cofactors, Vitamins, Prosthetic Groups, Pigments | Biotin | [Biotin biosynthesis Experimental](https://rast.nmpdr.org/seedviewer.cgi?page=Subsystems&subsystem=Biotin_biosynthesis_Experimental&organism=549.408) | [Biotin synthesis protein BioC](https://rast.nmpdr.org/seedviewer.cgi?page=FunctionalRole&role=Biotin%20synthesis%20protein%20BioC&subsystem_name=Biotin_biosynthesis_Experimental) |
| Cofactors, Vitamins, Prosthetic Groups, Pigments | Cofactors, Vitamins, Prosthetic Groups, Pigments - no subcategory | [Thiamin biosynthesis](https://rast.nmpdr.org/seedviewer.cgi?page=Subsystems&subsystem=Thiamin_biosynthesis&organism=549.408) | [Hydroxymethylpyrimidine ABC transporter, substrate-binding component](https://rast.nmpdr.org/seedviewer.cgi?page=FunctionalRole&role=Hydroxymethylpyrimidine%20ABC%20transporter,%20substrate-binding%20component&subsystem_name=Thiamin_biosynthesis) |
| Cofactors, Vitamins, Prosthetic Groups, Pigments | Cofactors, Vitamins, Prosthetic Groups, Pigments - no subcategory | [Thiamin biosynthesis](https://rast.nmpdr.org/seedviewer.cgi?page=Subsystems&subsystem=Thiamin_biosynthesis&organism=549.408) | [Hydroxymethylpyrimidine ABC transporter, ATPase component](https://rast.nmpdr.org/seedviewer.cgi?page=FunctionalRole&role=Hydroxymethylpyrimidine%20ABC%20transporter,%20ATPase%20component&subsystem_name=Thiamin_biosynthesis) |
| Cofactors, Vitamins, Prosthetic Groups, Pigments | Cofactors, Vitamins, Prosthetic Groups, Pigments - no subcategory | [Thiamin biosynthesis](https://rast.nmpdr.org/seedviewer.cgi?page=Subsystems&subsystem=Thiamin_biosynthesis&organism=549.408) | [Thiamin ABC transporter, transmembrane component](https://rast.nmpdr.org/seedviewer.cgi?page=FunctionalRole&role=Thiamin%20ABC%20transporter,%20transmembrane%20component&subsystem_name=Thiamin_biosynthesis) |
| Cofactors, Vitamins, Prosthetic Groups, Pigments | Cofactors, Vitamins, Prosthetic Groups, Pigments - no subcategory | [Thiamin biosynthesis](https://rast.nmpdr.org/seedviewer.cgi?page=Subsystems&subsystem=Thiamin_biosynthesis&organism=549.408) | [Hydroxyethylthiazole kinase (EC 2.7.1.50)](https://rast.nmpdr.org/seedviewer.cgi?page=FunctionalRole&role=Hydroxyethylthiazole%20kinase%20(EC%202.7.1.50)&subsystem_name=Thiamin_biosynthesis) |
| Cofactors, Vitamins, Prosthetic Groups, Pigments | Cofactors, Vitamins, Prosthetic Groups, Pigments - no subcategory | [Thiamin biosynthesis](https://rast.nmpdr.org/seedviewer.cgi?page=Subsystems&subsystem=Thiamin_biosynthesis&organism=549.408) | [Sulfur carrier protein adenylyltransferase ThiF](https://rast.nmpdr.org/seedviewer.cgi?page=FunctionalRole&role=Sulfur%20carrier%20protein%20adenylyltransferase%20ThiF&subsystem_name=Thiamin_biosynthesis) |
| Cofactors, Vitamins, Prosthetic Groups, Pigments | Cofactors, Vitamins, Prosthetic Groups, Pigments - no subcategory | [Thiamin biosynthesis](https://rast.nmpdr.org/seedviewer.cgi?page=Subsystems&subsystem=Thiamin_biosynthesis&organism=549.408) | [Glycine oxidase ThiO (EC 1.4.3.19)](https://rast.nmpdr.org/seedviewer.cgi?page=FunctionalRole&role=Glycine%20oxidase%20ThiO%20(EC%201.4.3.19)&subsystem_name=Thiamin_biosynthesis) |
| Cofactors, Vitamins, Prosthetic Groups, Pigments | Cofactors, Vitamins, Prosthetic Groups, Pigments - no subcategory | [Thiamin biosynthesis](https://rast.nmpdr.org/seedviewer.cgi?page=Subsystems&subsystem=Thiamin_biosynthesis&organism=549.408) | [Thiamin ABC transporter, ATPase component](https://rast.nmpdr.org/seedviewer.cgi?page=FunctionalRole&role=Thiamin%20ABC%20transporter,%20ATPase%20component&subsystem_name=Thiamin_biosynthesis) |
| Cofactors, Vitamins, Prosthetic Groups, Pigments | Cofactors, Vitamins, Prosthetic Groups, Pigments - no subcategory | [Thiamin biosynthesis](https://rast.nmpdr.org/seedviewer.cgi?page=Subsystems&subsystem=Thiamin_biosynthesis&organism=549.408) | [Thiazole biosynthesis protein ThiG](https://rast.nmpdr.org/seedviewer.cgi?page=FunctionalRole&role=Thiazole%20biosynthesis%20protein%20ThiG&subsystem_name=Thiamin_biosynthesis) |
| Cofactors, Vitamins, Prosthetic Groups, Pigments | Cofactors, Vitamins, Prosthetic Groups, Pigments - no subcategory | [Thiamin biosynthesis](https://rast.nmpdr.org/seedviewer.cgi?page=Subsystems&subsystem=Thiamin_biosynthesis&organism=549.408) | [Thiamine-monophosphate kinase (EC 2.7.4.16)](https://rast.nmpdr.org/seedviewer.cgi?page=FunctionalRole&role=Thiamine-monophosphate%20kinase%20(EC%202.7.4.16)&subsystem_name=Thiamin_biosynthesis) |
| Cofactors, Vitamins, Prosthetic Groups, Pigments | Cofactors, Vitamins, Prosthetic Groups, Pigments - no subcategory | [Thiamin biosynthesis](https://rast.nmpdr.org/seedviewer.cgi?page=Subsystems&subsystem=Thiamin_biosynthesis&organism=549.408) | [1-deoxy-D-xylulose 5-phosphate synthase (EC 2.2.1.7)](https://rast.nmpdr.org/seedviewer.cgi?page=FunctionalRole&role=1-deoxy-D-xylulose%205-phosphate%20synthase%20(EC%202.2.1.7)&subsystem_name=Thiamin_biosynthesis) |
| Cofactors, Vitamins, Prosthetic Groups, Pigments | Cofactors, Vitamins, Prosthetic Groups, Pigments - no subcategory | [Thiamin biosynthesis](https://rast.nmpdr.org/seedviewer.cgi?page=Subsystems&subsystem=Thiamin_biosynthesis&organism=549.408) | [Thiamin ABC transporter, substrate-binding component](https://rast.nmpdr.org/seedviewer.cgi?page=FunctionalRole&role=Thiamin%20ABC%20transporter,%20substrate-binding%20component&subsystem_name=Thiamin_biosynthesis) |
| Cofactors, Vitamins, Prosthetic Groups, Pigments | Cofactors, Vitamins, Prosthetic Groups, Pigments - no subcategory | [Thiamin biosynthesis](https://rast.nmpdr.org/seedviewer.cgi?page=Subsystems&subsystem=Thiamin_biosynthesis&organism=549.408) | [Hydroxymethylpyrimidine ABC transporter, transmembrane component](https://rast.nmpdr.org/seedviewer.cgi?page=FunctionalRole&role=Hydroxymethylpyrimidine%20ABC%20transporter,%20transmembrane%20component&subsystem_name=Thiamin_biosynthesis) |
| Cofactors, Vitamins, Prosthetic Groups, Pigments | Cofactors, Vitamins, Prosthetic Groups, Pigments - no subcategory | [Thiamin biosynthesis](https://rast.nmpdr.org/seedviewer.cgi?page=Subsystems&subsystem=Thiamin_biosynthesis&organism=549.408) | [Cysteine desulfurase (EC 2.8.1.7), IscS subfamily](https://rast.nmpdr.org/seedviewer.cgi?page=FunctionalRole&role=Cysteine%20desulfurase%20(EC%202.8.1.7),%20IscS%20subfamily&subsystem_name=Thiamin_biosynthesis) |
| Cofactors, Vitamins, Prosthetic Groups, Pigments | Cofactors, Vitamins, Prosthetic Groups, Pigments - no subcategory | [Thiamin biosynthesis](https://rast.nmpdr.org/seedviewer.cgi?page=Subsystems&subsystem=Thiamin_biosynthesis&organism=549.408) | [Thiamine kinase (EC 2.7.1.89)](https://rast.nmpdr.org/seedviewer.cgi?page=FunctionalRole&role=Thiamine%20kinase%20(EC%202.7.1.89)&subsystem_name=Thiamin_biosynthesis) |
| Cofactors, Vitamins, Prosthetic Groups, Pigments | Cofactors, Vitamins, Prosthetic Groups, Pigments - no subcategory | [Thiamin biosynthesis](https://rast.nmpdr.org/seedviewer.cgi?page=Subsystems&subsystem=Thiamin_biosynthesis&organism=549.408) | [Thiamin-phosphate pyrophosphorylase (EC 2.5.1.3)](https://rast.nmpdr.org/seedviewer.cgi?page=FunctionalRole&role=Thiamin-phosphate%20pyrophosphorylase%20(EC%202.5.1.3)&subsystem_name=Thiamin_biosynthesis) |
| Cofactors, Vitamins, Prosthetic Groups, Pigments | Cofactors, Vitamins, Prosthetic Groups, Pigments - no subcategory | [Thiamin biosynthesis](https://rast.nmpdr.org/seedviewer.cgi?page=Subsystems&subsystem=Thiamin_biosynthesis&organism=549.408) | [Sulfur carrier protein ThiS](https://rast.nmpdr.org/seedviewer.cgi?page=FunctionalRole&role=Sulfur%20carrier%20protein%20ThiS&subsystem_name=Thiamin_biosynthesis) |
| Cofactors, Vitamins, Prosthetic Groups, Pigments | Quinone cofactors | [Menaquinone and Phylloquinone Biosynthesis](https://rast.nmpdr.org/seedviewer.cgi?page=Subsystems&subsystem=Menaquinone_and_Phylloquinone_Biosynthesis&organism=549.408) | [Ubiquinone/menaquinone biosynthesis methyltransferase UbiE (EC 2.1.1.-)](https://rast.nmpdr.org/seedviewer.cgi?page=FunctionalRole&role=Ubiquinone/menaquinone%20biosynthesis%20methyltransferase%20UbiE%20(EC%202.1.1.-)&subsystem_name=Menaquinone_and_Phylloquinone_Biosynthesis) |
| Cofactors, Vitamins, Prosthetic Groups, Pigments | Quinone cofactors | [Ubiquinone Biosynthesis](https://rast.nmpdr.org/seedviewer.cgi?page=Subsystems&subsystem=Ubiquinone_Biosynthesis&organism=549.408) | [2-octaprenyl-6-methoxyphenol hydroxylase (EC 1.14.13.-)](https://rast.nmpdr.org/seedviewer.cgi?page=FunctionalRole&role=2-octaprenyl-6-methoxyphenol%20hydroxylase%20(EC%201.14.13.-)&subsystem_name=Ubiquinone_Biosynthesis) |
| Cofactors, Vitamins, Prosthetic Groups, Pigments | Quinone cofactors | [Ubiquinone Biosynthesis](https://rast.nmpdr.org/seedviewer.cgi?page=Subsystems&subsystem=Ubiquinone_Biosynthesis&organism=549.408) | [Ubiquinone biosynthesis monooxygenase UbiB](https://rast.nmpdr.org/seedviewer.cgi?page=FunctionalRole&role=Ubiquinone%20biosynthesis%20monooxygenase%20UbiB&subsystem_name=Ubiquinone_Biosynthesis) |
| Cofactors, Vitamins, Prosthetic Groups, Pigments | Quinone cofactors | [Ubiquinone Biosynthesis](https://rast.nmpdr.org/seedviewer.cgi?page=Subsystems&subsystem=Ubiquinone_Biosynthesis&organism=549.408) | [Ubiquinone/menaquinone biosynthesis methyltransferase UbiE (EC 2.1.1.-)](https://rast.nmpdr.org/seedviewer.cgi?page=FunctionalRole&role=Ubiquinone/menaquinone%20biosynthesis%20methyltransferase%20UbiE%20(EC%202.1.1.-)&subsystem_name=Ubiquinone_Biosynthesis) |
| Cofactors, Vitamins, Prosthetic Groups, Pigments | Quinone cofactors | [Ubiquinone Biosynthesis](https://rast.nmpdr.org/seedviewer.cgi?page=Subsystems&subsystem=Ubiquinone_Biosynthesis&organism=549.408) | [4-hydroxybenzoate polyprenyltransferase (EC 2.5.1.39)](https://rast.nmpdr.org/seedviewer.cgi?page=FunctionalRole&role=4-hydroxybenzoate%20polyprenyltransferase%20(EC%202.5.1.39)&subsystem_name=Ubiquinone_Biosynthesis) |
| Cofactors, Vitamins, Prosthetic Groups, Pigments | Quinone cofactors | [Ubiquinone Biosynthesis](https://rast.nmpdr.org/seedviewer.cgi?page=Subsystems&subsystem=Ubiquinone_Biosynthesis&organism=549.408) | [3-polyprenyl-4-hydroxybenzoate carboxy-lyase UbiX (EC 4.1.1.-)](https://rast.nmpdr.org/seedviewer.cgi?page=FunctionalRole&role=3-polyprenyl-4-hydroxybenzoate%20carboxy-lyase%20UbiX%20(EC%204.1.1.-)&subsystem_name=Ubiquinone_Biosynthesis) |
| Cofactors, Vitamins, Prosthetic Groups, Pigments | Quinone cofactors | [Ubiquinone Biosynthesis](https://rast.nmpdr.org/seedviewer.cgi?page=Subsystems&subsystem=Ubiquinone_Biosynthesis&organism=549.408) | [Chorismate--pyruvate lyase (EC 4.1.3.40)](https://rast.nmpdr.org/seedviewer.cgi?page=FunctionalRole&role=Chorismate--pyruvate%20lyase%20(EC%204.1.3.40)&subsystem_name=Ubiquinone_Biosynthesis) |
| Cofactors, Vitamins, Prosthetic Groups, Pigments | Quinone cofactors | [Ubiquinone Biosynthesis](https://rast.nmpdr.org/seedviewer.cgi?page=Subsystems&subsystem=Ubiquinone_Biosynthesis&organism=549.408) | [2-octaprenyl-3-methyl-6-methoxy-1,4-benzoquinol hydroxylase (EC 1.14.13.-)](https://rast.nmpdr.org/seedviewer.cgi?page=FunctionalRole&role=2-octaprenyl-3-methyl-6-methoxy-1,4-benzoquinol%20hydroxylase%20(EC%201.14.13.-)&subsystem_name=Ubiquinone_Biosynthesis) |
| Cofactors, Vitamins, Prosthetic Groups, Pigments | Quinone cofactors | [Ubiquinone Biosynthesis](https://rast.nmpdr.org/seedviewer.cgi?page=Subsystems&subsystem=Ubiquinone_Biosynthesis&organism=549.408) | [NAD(P)H-flavin reductase (EC 1.5.1.29) (EC 1.16.1.3)](https://rast.nmpdr.org/seedviewer.cgi?page=FunctionalRole&role=NAD(P)H-flavin%20reductase%20(EC%201.5.1.29)%20(EC%201.16.1.3)&subsystem_name=Ubiquinone_Biosynthesis) |
| Cofactors, Vitamins, Prosthetic Groups, Pigments | Quinone cofactors | [Ubiquinone Biosynthesis](https://rast.nmpdr.org/seedviewer.cgi?page=Subsystems&subsystem=Ubiquinone_Biosynthesis&organism=549.408) | [Protein YigP (COG3165) clustered with ubiquinone biosynthetic genes](https://rast.nmpdr.org/seedviewer.cgi?page=FunctionalRole&role=Protein%20YigP%20(COG3165)%20clustered%20with%20ubiquinone%20biosynthetic%20genes&subsystem_name=Ubiquinone_Biosynthesis) |
| Cofactors, Vitamins, Prosthetic Groups, Pigments | Quinone cofactors | [Ubiquinone Biosynthesis](https://rast.nmpdr.org/seedviewer.cgi?page=Subsystems&subsystem=Ubiquinone_Biosynthesis&organism=549.408) | [3-polyprenyl-4-hydroxybenzoate carboxy-lyase (EC 4.1.1.-)](https://rast.nmpdr.org/seedviewer.cgi?page=FunctionalRole&role=3-polyprenyl-4-hydroxybenzoate%20carboxy-lyase%20(EC%204.1.1.-)&subsystem_name=Ubiquinone_Biosynthesis) |
| Cofactors, Vitamins, Prosthetic Groups, Pigments | Quinone cofactors | [Ubiquinone Biosynthesis - gjo](https://rast.nmpdr.org/seedviewer.cgi?page=Subsystems&subsystem=Ubiquinone_Biosynthesis_-_gjo&organism=549.408) | [2-octaprenyl-6-methoxyphenol hydroxylase (EC 1.14.13.-)](https://rast.nmpdr.org/seedviewer.cgi?page=FunctionalRole&role=2-octaprenyl-6-methoxyphenol%20hydroxylase%20(EC%201.14.13.-)&subsystem_name=Ubiquinone_Biosynthesis_-_gjo) |
| Cofactors, Vitamins, Prosthetic Groups, Pigments | Quinone cofactors | [Ubiquinone Biosynthesis - gjo](https://rast.nmpdr.org/seedviewer.cgi?page=Subsystems&subsystem=Ubiquinone_Biosynthesis_-_gjo&organism=549.408) | [Ubiquinone biosynthesis monooxygenase UbiB](https://rast.nmpdr.org/seedviewer.cgi?page=FunctionalRole&role=Ubiquinone%20biosynthesis%20monooxygenase%20UbiB&subsystem_name=Ubiquinone_Biosynthesis_-_gjo) |
| Cofactors, Vitamins, Prosthetic Groups, Pigments | Quinone cofactors | [Ubiquinone Biosynthesis - gjo](https://rast.nmpdr.org/seedviewer.cgi?page=Subsystems&subsystem=Ubiquinone_Biosynthesis_-_gjo&organism=549.408) | [Ubiquinone/menaquinone biosynthesis methyltransferase UbiE (EC 2.1.1.-)](https://rast.nmpdr.org/seedviewer.cgi?page=FunctionalRole&role=Ubiquinone/menaquinone%20biosynthesis%20methyltransferase%20UbiE%20(EC%202.1.1.-)&subsystem_name=Ubiquinone_Biosynthesis_-_gjo) |
| Cofactors, Vitamins, Prosthetic Groups, Pigments | Quinone cofactors | [Ubiquinone Biosynthesis - gjo](https://rast.nmpdr.org/seedviewer.cgi?page=Subsystems&subsystem=Ubiquinone_Biosynthesis_-_gjo&organism=549.408) | [4-hydroxybenzoate polyprenyltransferase (EC 2.5.1.39)](https://rast.nmpdr.org/seedviewer.cgi?page=FunctionalRole&role=4-hydroxybenzoate%20polyprenyltransferase%20(EC%202.5.1.39)&subsystem_name=Ubiquinone_Biosynthesis_-_gjo) |
| Cofactors, Vitamins, Prosthetic Groups, Pigments | Quinone cofactors | [Ubiquinone Biosynthesis - gjo](https://rast.nmpdr.org/seedviewer.cgi?page=Subsystems&subsystem=Ubiquinone_Biosynthesis_-_gjo&organism=549.408) | [3-polyprenyl-4-hydroxybenzoate carboxy-lyase UbiX (EC 4.1.1.-)](https://rast.nmpdr.org/seedviewer.cgi?page=FunctionalRole&role=3-polyprenyl-4-hydroxybenzoate%20carboxy-lyase%20UbiX%20(EC%204.1.1.-)&subsystem_name=Ubiquinone_Biosynthesis_-_gjo) |
| Cofactors, Vitamins, Prosthetic Groups, Pigments | Quinone cofactors | [Ubiquinone Biosynthesis - gjo](https://rast.nmpdr.org/seedviewer.cgi?page=Subsystems&subsystem=Ubiquinone_Biosynthesis_-_gjo&organism=549.408) | [Chorismate--pyruvate lyase (EC 4.1.3.40)](https://rast.nmpdr.org/seedviewer.cgi?page=FunctionalRole&role=Chorismate--pyruvate%20lyase%20(EC%204.1.3.40)&subsystem_name=Ubiquinone_Biosynthesis_-_gjo) |
| Cofactors, Vitamins, Prosthetic Groups, Pigments | Quinone cofactors | [Ubiquinone Biosynthesis - gjo](https://rast.nmpdr.org/seedviewer.cgi?page=Subsystems&subsystem=Ubiquinone_Biosynthesis_-_gjo&organism=549.408) | [2-octaprenyl-3-methyl-6-methoxy-1,4-benzoquinol hydroxylase (EC 1.14.13.-)](https://rast.nmpdr.org/seedviewer.cgi?page=FunctionalRole&role=2-octaprenyl-3-methyl-6-methoxy-1,4-benzoquinol%20hydroxylase%20(EC%201.14.13.-)&subsystem_name=Ubiquinone_Biosynthesis_-_gjo) |
| Cofactors, Vitamins, Prosthetic Groups, Pigments | Quinone cofactors | [Ubiquinone Biosynthesis - gjo](https://rast.nmpdr.org/seedviewer.cgi?page=Subsystems&subsystem=Ubiquinone_Biosynthesis_-_gjo&organism=549.408) | [NAD(P)H-flavin reductase (EC 1.5.1.29) (EC 1.16.1.3)](https://rast.nmpdr.org/seedviewer.cgi?page=FunctionalRole&role=NAD(P)H-flavin%20reductase%20(EC%201.5.1.29)%20(EC%201.16.1.3)&subsystem_name=Ubiquinone_Biosynthesis_-_gjo) |
| Cofactors, Vitamins, Prosthetic Groups, Pigments | Quinone cofactors | [Ubiquinone Biosynthesis - gjo](https://rast.nmpdr.org/seedviewer.cgi?page=Subsystems&subsystem=Ubiquinone_Biosynthesis_-_gjo&organism=549.408) | [Protein YigP (COG3165) clustered with ubiquinone biosynthetic genes](https://rast.nmpdr.org/seedviewer.cgi?page=FunctionalRole&role=Protein%20YigP%20(COG3165)%20clustered%20with%20ubiquinone%20biosynthetic%20genes&subsystem_name=Ubiquinone_Biosynthesis_-_gjo) |
| Cofactors, Vitamins, Prosthetic Groups, Pigments | Quinone cofactors | [Ubiquinone Biosynthesis - gjo](https://rast.nmpdr.org/seedviewer.cgi?page=Subsystems&subsystem=Ubiquinone_Biosynthesis_-_gjo&organism=549.408) | [3-polyprenyl-4-hydroxybenzoate carboxy-lyase (EC 4.1.1.-)](https://rast.nmpdr.org/seedviewer.cgi?page=FunctionalRole&role=3-polyprenyl-4-hydroxybenzoate%20carboxy-lyase%20(EC%204.1.1.-)&subsystem_name=Ubiquinone_Biosynthesis_-_gjo) |
| Cofactors, Vitamins, Prosthetic Groups, Pigments | Tetrapyrroles | [Heme and Siroheme Biosynthesis](https://rast.nmpdr.org/seedviewer.cgi?page=Subsystems&subsystem=Heme_and_Siroheme_Biosynthesis&organism=549.408) | [Glutamyl-tRNA synthetase (EC 6.1.1.17)](https://rast.nmpdr.org/seedviewer.cgi?page=FunctionalRole&role=Glutamyl-tRNA%20synthetase%20(EC%206.1.1.17)&subsystem_name=Heme_and_Siroheme_Biosynthesis) |
| Cofactors, Vitamins, Prosthetic Groups, Pigments | Tetrapyrroles | [Heme and Siroheme Biosynthesis](https://rast.nmpdr.org/seedviewer.cgi?page=Subsystems&subsystem=Heme_and_Siroheme_Biosynthesis&organism=549.408) | [Coproporphyrinogen III oxidase, aerobic (EC 1.3.3.3)](https://rast.nmpdr.org/seedviewer.cgi?page=FunctionalRole&role=Coproporphyrinogen%20III%20oxidase,%20aerobic%20(EC%201.3.3.3)&subsystem_name=Heme_and_Siroheme_Biosynthesis) |
| Cofactors, Vitamins, Prosthetic Groups, Pigments | Tetrapyrroles | [Heme and Siroheme Biosynthesis](https://rast.nmpdr.org/seedviewer.cgi?page=Subsystems&subsystem=Heme_and_Siroheme_Biosynthesis&organism=549.408) | [Uroporphyrinogen-III synthase (EC 4.2.1.75)](https://rast.nmpdr.org/seedviewer.cgi?page=FunctionalRole&role=Uroporphyrinogen-III%20synthase%20(EC%204.2.1.75)&subsystem_name=Heme_and_Siroheme_Biosynthesis) |
| Cofactors, Vitamins, Prosthetic Groups, Pigments | Tetrapyrroles | [Heme and Siroheme Biosynthesis](https://rast.nmpdr.org/seedviewer.cgi?page=Subsystems&subsystem=Heme_and_Siroheme_Biosynthesis&organism=549.408) | [Glutamate-1-semialdehyde aminotransferase (EC 5.4.3.8)](https://rast.nmpdr.org/seedviewer.cgi?page=FunctionalRole&role=Glutamate-1-semialdehyde%20aminotransferase%20(EC%205.4.3.8)&subsystem_name=Heme_and_Siroheme_Biosynthesis) |
| Cofactors, Vitamins, Prosthetic Groups, Pigments | Tetrapyrroles | [Heme and Siroheme Biosynthesis](https://rast.nmpdr.org/seedviewer.cgi?page=Subsystems&subsystem=Heme_and_Siroheme_Biosynthesis&organism=549.408) | [Precorrin-2 oxidase (EC 1.3.1.76)](https://rast.nmpdr.org/seedviewer.cgi?page=FunctionalRole&role=Precorrin-2%20oxidase%20(EC%201.3.1.76)&subsystem_name=Heme_and_Siroheme_Biosynthesis) |
| Cofactors, Vitamins, Prosthetic Groups, Pigments | Tetrapyrroles | [Heme and Siroheme Biosynthesis](https://rast.nmpdr.org/seedviewer.cgi?page=Subsystems&subsystem=Heme_and_Siroheme_Biosynthesis&organism=549.408) | [Porphobilinogen synthase (EC 4.2.1.24)](https://rast.nmpdr.org/seedviewer.cgi?page=FunctionalRole&role=Porphobilinogen%20synthase%20(EC%204.2.1.24)&subsystem_name=Heme_and_Siroheme_Biosynthesis) |
| Cofactors, Vitamins, Prosthetic Groups, Pigments | Tetrapyrroles | [Heme and Siroheme Biosynthesis](https://rast.nmpdr.org/seedviewer.cgi?page=Subsystems&subsystem=Heme_and_Siroheme_Biosynthesis&organism=549.408) | [Radical SAM family enzyme, similar to coproporphyrinogen III oxidase, oxygen-independent, clustered with nucleoside-triphosphatase RdgB](https://rast.nmpdr.org/seedviewer.cgi?page=FunctionalRole&role=Radical%20SAM%20family%20enzyme,%20similar%20to%20coproporphyrinogen%20III%20oxidase,%20oxygen-independent,%20clustered%20with%20nucleoside-triphosphatase%20RdgB&subsystem_name=Heme_and_Siroheme_Biosynthesis) |
| Cofactors, Vitamins, Prosthetic Groups, Pigments | Tetrapyrroles | [Heme and Siroheme Biosynthesis](https://rast.nmpdr.org/seedviewer.cgi?page=Subsystems&subsystem=Heme_and_Siroheme_Biosynthesis&organism=549.408) | [Protoporphyrinogen IX oxidase, oxygen-independent, HemG (EC 1.3.-.-)](https://rast.nmpdr.org/seedviewer.cgi?page=FunctionalRole&role=Protoporphyrinogen%20IX%20oxidase,%20oxygen-independent,%20HemG%20(EC%201.3.-.-)&subsystem_name=Heme_and_Siroheme_Biosynthesis) |
| Cofactors, Vitamins, Prosthetic Groups, Pigments | Tetrapyrroles | [Heme and Siroheme Biosynthesis](https://rast.nmpdr.org/seedviewer.cgi?page=Subsystems&subsystem=Heme_and_Siroheme_Biosynthesis&organism=549.408) | [Coproporphyrinogen III oxidase, oxygen-independent (EC 1.3.99.22)](https://rast.nmpdr.org/seedviewer.cgi?page=FunctionalRole&role=Coproporphyrinogen%20III%20oxidase,%20oxygen-independent%20(EC%201.3.99.22)&subsystem_name=Heme_and_Siroheme_Biosynthesis) |
| Cofactors, Vitamins, Prosthetic Groups, Pigments | Tetrapyrroles | [Heme and Siroheme Biosynthesis](https://rast.nmpdr.org/seedviewer.cgi?page=Subsystems&subsystem=Heme_and_Siroheme_Biosynthesis&organism=549.408) | [Glutamyl-tRNA reductase (EC 1.2.1.70)](https://rast.nmpdr.org/seedviewer.cgi?page=FunctionalRole&role=Glutamyl-tRNA%20reductase%20(EC%201.2.1.70)&subsystem_name=Heme_and_Siroheme_Biosynthesis) |
| Cofactors, Vitamins, Prosthetic Groups, Pigments | Tetrapyrroles | [Heme and Siroheme Biosynthesis](https://rast.nmpdr.org/seedviewer.cgi?page=Subsystems&subsystem=Heme_and_Siroheme_Biosynthesis&organism=549.408) | [Porphobilinogen deaminase (EC 2.5.1.61)](https://rast.nmpdr.org/seedviewer.cgi?page=FunctionalRole&role=Porphobilinogen%20deaminase%20(EC%202.5.1.61)&subsystem_name=Heme_and_Siroheme_Biosynthesis) |
| Cofactors, Vitamins, Prosthetic Groups, Pigments | Tetrapyrroles | [Heme and Siroheme Biosynthesis](https://rast.nmpdr.org/seedviewer.cgi?page=Subsystems&subsystem=Heme_and_Siroheme_Biosynthesis&organism=549.408) | [Uroporphyrinogen-III methyltransferase (EC 2.1.1.107)](https://rast.nmpdr.org/seedviewer.cgi?page=FunctionalRole&role=Uroporphyrinogen-III%20methyltransferase%20(EC%202.1.1.107)&subsystem_name=Heme_and_Siroheme_Biosynthesis) |
| Cofactors, Vitamins, Prosthetic Groups, Pigments | Tetrapyrroles | [Heme and Siroheme Biosynthesis](https://rast.nmpdr.org/seedviewer.cgi?page=Subsystems&subsystem=Heme_and_Siroheme_Biosynthesis&organism=549.408) | [Ferrochelatase, protoheme ferro-lyase (EC 4.99.1.1)](https://rast.nmpdr.org/seedviewer.cgi?page=FunctionalRole&role=Ferrochelatase,%20protoheme%20ferro-lyase%20(EC%204.99.1.1)&subsystem_name=Heme_and_Siroheme_Biosynthesis) |
| Cofactors, Vitamins, Prosthetic Groups, Pigments | Tetrapyrroles | [Heme and Siroheme Biosynthesis](https://rast.nmpdr.org/seedviewer.cgi?page=Subsystems&subsystem=Heme_and_Siroheme_Biosynthesis&organism=549.408) | [Uroporphyrinogen III decarboxylase (EC 4.1.1.37)](https://rast.nmpdr.org/seedviewer.cgi?page=FunctionalRole&role=Uroporphyrinogen%20III%20decarboxylase%20(EC%204.1.1.37)&subsystem_name=Heme_and_Siroheme_Biosynthesis) |
| Cofactors, Vitamins, Prosthetic Groups, Pigments | Tetrapyrroles | [Heme and Siroheme Biosynthesis](https://rast.nmpdr.org/seedviewer.cgi?page=Subsystems&subsystem=Heme_and_Siroheme_Biosynthesis&organism=549.408) | [Sirohydrochlorin ferrochelatase (EC 4.99.1.4)](https://rast.nmpdr.org/seedviewer.cgi?page=FunctionalRole&role=Sirohydrochlorin%20ferrochelatase%20(EC%204.99.1.4)&subsystem_name=Heme_and_Siroheme_Biosynthesis) |
| Cofactors, Vitamins, Prosthetic Groups, Pigments | Riboflavin, FMN, FAD | [riboflavin to FAD](https://rast.nmpdr.org/seedviewer.cgi?page=Subsystems&subsystem=riboflavin_to_FAD&organism=549.408) | [FMN adenylyltransferase (EC 2.7.7.2)](https://rast.nmpdr.org/seedviewer.cgi?page=FunctionalRole&role=FMN%20adenylyltransferase%20(EC%202.7.7.2)&subsystem_name=riboflavin_to_FAD) |
| Cofactors, Vitamins, Prosthetic Groups, Pigments | Riboflavin, FMN, FAD | [riboflavin to FAD](https://rast.nmpdr.org/seedviewer.cgi?page=Subsystems&subsystem=riboflavin_to_FAD&organism=549.408) | [Riboflavin synthase eubacterial/eukaryotic (EC 2.5.1.9)](https://rast.nmpdr.org/seedviewer.cgi?page=FunctionalRole&role=Riboflavin%20synthase%20eubacterial/eukaryotic%20(EC%202.5.1.9)&subsystem_name=riboflavin_to_FAD) |
| Cofactors, Vitamins, Prosthetic Groups, Pigments | Riboflavin, FMN, FAD | [riboflavin to FAD](https://rast.nmpdr.org/seedviewer.cgi?page=Subsystems&subsystem=riboflavin_to_FAD&organism=549.408) | [Riboflavin kinase (EC 2.7.1.26)](https://rast.nmpdr.org/seedviewer.cgi?page=FunctionalRole&role=Riboflavin%20kinase%20(EC%202.7.1.26)&subsystem_name=riboflavin_to_FAD) |
| Cofactors, Vitamins, Prosthetic Groups, Pigments | Riboflavin, FMN, FAD | [riboflavin to FAD](https://rast.nmpdr.org/seedviewer.cgi?page=Subsystems&subsystem=riboflavin_to_FAD&organism=549.408) | [3,4-dihydroxy-2-butanone 4-phosphate synthase (EC 4.1.99.12)](https://rast.nmpdr.org/seedviewer.cgi?page=FunctionalRole&role=3,4-dihydroxy-2-butanone%204-phosphate%20synthase%20(EC%204.1.99.12)&subsystem_name=riboflavin_to_FAD) |
| Cofactors, Vitamins, Prosthetic Groups, Pigments | Riboflavin, FMN, FAD | [Flavodoxin](https://rast.nmpdr.org/seedviewer.cgi?page=Subsystems&subsystem=Flavodoxin&organism=549.408) | [Hypothetical flavoprotein YqcA (clustered with tRNA pseudouridine synthase C)](https://rast.nmpdr.org/seedviewer.cgi?page=FunctionalRole&role=Hypothetical%20flavoprotein%20YqcA%20(clustered%20with%20tRNA%20pseudouridine%20synthase%20C)&subsystem_name=Flavodoxin) |
| Cofactors, Vitamins, Prosthetic Groups, Pigments | Riboflavin, FMN, FAD | [Flavodoxin](https://rast.nmpdr.org/seedviewer.cgi?page=Subsystems&subsystem=Flavodoxin&organism=549.408) | [NAD(P)H oxidoreductase YRKL (EC 1.6.99.-)](https://rast.nmpdr.org/seedviewer.cgi?page=FunctionalRole&role=NAD(P)H%20oxidoreductase%20YRKL%20(EC%201.6.99.-)&subsystem_name=Flavodoxin) |
| Cofactors, Vitamins, Prosthetic Groups, Pigments | Riboflavin, FMN, FAD | [Flavodoxin](https://rast.nmpdr.org/seedviewer.cgi?page=Subsystems&subsystem=Flavodoxin&organism=549.408) | [Flavodoxin 1](https://rast.nmpdr.org/seedviewer.cgi?page=FunctionalRole&role=Flavodoxin%201&subsystem_name=Flavodoxin) |
| Cofactors, Vitamins, Prosthetic Groups, Pigments | Riboflavin, FMN, FAD | [Flavodoxin](https://rast.nmpdr.org/seedviewer.cgi?page=Subsystems&subsystem=Flavodoxin&organism=549.408) | [Flavodoxin 2](https://rast.nmpdr.org/seedviewer.cgi?page=FunctionalRole&role=Flavodoxin%202&subsystem_name=Flavodoxin) |
| Cofactors, Vitamins, Prosthetic Groups, Pigments | Riboflavin, FMN, FAD | [Flavodoxin](https://rast.nmpdr.org/seedviewer.cgi?page=Subsystems&subsystem=Flavodoxin&organism=549.408) | [Flavoprotein MioC](https://rast.nmpdr.org/seedviewer.cgi?page=FunctionalRole&role=Flavoprotein%20MioC&subsystem_name=Flavodoxin) |
| Cofactors, Vitamins, Prosthetic Groups, Pigments | Riboflavin, FMN, FAD | [Riboflavin, FMN and FAD metabolism](https://rast.nmpdr.org/seedviewer.cgi?page=Subsystems&subsystem=Riboflavin,_FMN_and_FAD_metabolism&organism=549.408) | [FMN adenylyltransferase (EC 2.7.7.2)](https://rast.nmpdr.org/seedviewer.cgi?page=FunctionalRole&role=FMN%20adenylyltransferase%20(EC%202.7.7.2)&subsystem_name=Riboflavin,_FMN_and_FAD_metabolism) |
| Cofactors, Vitamins, Prosthetic Groups, Pigments | Riboflavin, FMN, FAD | [Riboflavin, FMN and FAD metabolism](https://rast.nmpdr.org/seedviewer.cgi?page=Subsystems&subsystem=Riboflavin,_FMN_and_FAD_metabolism&organism=549.408) | [6,7-dimethyl-8-ribityllumazine synthase (EC 2.5.1.78)](https://rast.nmpdr.org/seedviewer.cgi?page=FunctionalRole&role=6,7-dimethyl-8-ribityllumazine%20synthase%20(EC%202.5.1.78)&subsystem_name=Riboflavin,_FMN_and_FAD_metabolism) |
| Cofactors, Vitamins, Prosthetic Groups, Pigments | Riboflavin, FMN, FAD | [Riboflavin, FMN and FAD metabolism](https://rast.nmpdr.org/seedviewer.cgi?page=Subsystems&subsystem=Riboflavin,_FMN_and_FAD_metabolism&organism=549.408) | [5-Amino-6-(5'-phosphoribitylamino)uracil phosphatase](https://rast.nmpdr.org/seedviewer.cgi?page=FunctionalRole&role=5-Amino-6-(5) |
| Cofactors, Vitamins, Prosthetic Groups, Pigments | Riboflavin, FMN, FAD | [Riboflavin, FMN and FAD metabolism](https://rast.nmpdr.org/seedviewer.cgi?page=Subsystems&subsystem=Riboflavin,_FMN_and_FAD_metabolism&organism=549.408) | [5-amino-6-(5-phosphoribosylamino)uracil reductase (EC 1.1.1.193)](https://rast.nmpdr.org/seedviewer.cgi?page=FunctionalRole&role=5-amino-6-(5-phosphoribosylamino)uracil%20reductase%20(EC%201.1.1.193)&subsystem_name=Riboflavin,_FMN_and_FAD_metabolism) |
| Cofactors, Vitamins, Prosthetic Groups, Pigments | Riboflavin, FMN, FAD | [Riboflavin, FMN and FAD metabolism](https://rast.nmpdr.org/seedviewer.cgi?page=Subsystems&subsystem=Riboflavin,_FMN_and_FAD_metabolism&organism=549.408) | [Riboflavin kinase (EC 2.7.1.26)](https://rast.nmpdr.org/seedviewer.cgi?page=FunctionalRole&role=Riboflavin%20kinase%20(EC%202.7.1.26)&subsystem_name=Riboflavin,_FMN_and_FAD_metabolism) |
| Cofactors, Vitamins, Prosthetic Groups, Pigments | Riboflavin, FMN, FAD | [Riboflavin, FMN and FAD metabolism](https://rast.nmpdr.org/seedviewer.cgi?page=Subsystems&subsystem=Riboflavin,_FMN_and_FAD_metabolism&organism=549.408) | [GTP cyclohydrolase II (EC 3.5.4.25)](https://rast.nmpdr.org/seedviewer.cgi?page=FunctionalRole&role=GTP%20cyclohydrolase%20II%20(EC%203.5.4.25)&subsystem_name=Riboflavin,_FMN_and_FAD_metabolism) |
| Cofactors, Vitamins, Prosthetic Groups, Pigments | Riboflavin, FMN, FAD | [Riboflavin, FMN and FAD metabolism](https://rast.nmpdr.org/seedviewer.cgi?page=Subsystems&subsystem=Riboflavin,_FMN_and_FAD_metabolism&organism=549.408) | [Riboflavin synthase eubacterial/eukaryotic (EC 2.5.1.9)](https://rast.nmpdr.org/seedviewer.cgi?page=FunctionalRole&role=Riboflavin%20synthase%20eubacterial/eukaryotic%20(EC%202.5.1.9)&subsystem_name=Riboflavin,_FMN_and_FAD_metabolism) |
| Cofactors, Vitamins, Prosthetic Groups, Pigments | Riboflavin, FMN, FAD | [Riboflavin, FMN and FAD metabolism](https://rast.nmpdr.org/seedviewer.cgi?page=Subsystems&subsystem=Riboflavin,_FMN_and_FAD_metabolism&organism=549.408) | [Diaminohydroxyphosphoribosylaminopyrimidine deaminase (EC 3.5.4.26)](https://rast.nmpdr.org/seedviewer.cgi?page=FunctionalRole&role=Diaminohydroxyphosphoribosylaminopyrimidine%20deaminase%20(EC%203.5.4.26)&subsystem_name=Riboflavin,_FMN_and_FAD_metabolism) |
| Cofactors, Vitamins, Prosthetic Groups, Pigments | Riboflavin, FMN, FAD | [Riboflavin, FMN and FAD metabolism](https://rast.nmpdr.org/seedviewer.cgi?page=Subsystems&subsystem=Riboflavin,_FMN_and_FAD_metabolism&organism=549.408) | [3,4-dihydroxy-2-butanone 4-phosphate synthase (EC 4.1.99.12)](https://rast.nmpdr.org/seedviewer.cgi?page=FunctionalRole&role=3,4-dihydroxy-2-butanone%204-phosphate%20synthase%20(EC%204.1.99.12)&subsystem_name=Riboflavin,_FMN_and_FAD_metabolism) |
| Cofactors, Vitamins, Prosthetic Groups, Pigments | Pyridoxine | [Pyridoxin (Vitamin B6) Biosynthesis](https://rast.nmpdr.org/seedviewer.cgi?page=Subsystems&subsystem=Pyridoxin_(Vitamin_B6)_Biosynthesis&organism=549.408) | [D-3-phosphoglycerate dehydrogenase (EC 1.1.1.95)](https://rast.nmpdr.org/seedviewer.cgi?page=FunctionalRole&role=D-3-phosphoglycerate%20dehydrogenase%20(EC%201.1.1.95)&subsystem_name=Pyridoxin_(Vitamin_B6)_Biosynthesis) |
| Cofactors, Vitamins, Prosthetic Groups, Pigments | Pyridoxine | [Pyridoxin (Vitamin B6) Biosynthesis](https://rast.nmpdr.org/seedviewer.cgi?page=Subsystems&subsystem=Pyridoxin_(Vitamin_B6)_Biosynthesis&organism=549.408) | [4-hydroxythreonine-4-phosphate dehydrogenase (EC 1.1.1.262)](https://rast.nmpdr.org/seedviewer.cgi?page=FunctionalRole&role=4-hydroxythreonine-4-phosphate%20dehydrogenase%20(EC%201.1.1.262)&subsystem_name=Pyridoxin_(Vitamin_B6)_Biosynthesis) |
| Cofactors, Vitamins, Prosthetic Groups, Pigments | Pyridoxine | [Pyridoxin (Vitamin B6) Biosynthesis](https://rast.nmpdr.org/seedviewer.cgi?page=Subsystems&subsystem=Pyridoxin_(Vitamin_B6)_Biosynthesis&organism=549.408) | [Pyridoxamine 5'-phosphate oxidase (EC 1.4.3.5)](https://rast.nmpdr.org/seedviewer.cgi?page=FunctionalRole&role=Pyridoxamine%205) |
| Cofactors, Vitamins, Prosthetic Groups, Pigments | Pyridoxine | [Pyridoxin (Vitamin B6) Biosynthesis](https://rast.nmpdr.org/seedviewer.cgi?page=Subsystems&subsystem=Pyridoxin_(Vitamin_B6)_Biosynthesis&organism=549.408) | [Erythronate-4-phosphate dehydrogenase (EC 1.1.1.290)](https://rast.nmpdr.org/seedviewer.cgi?page=FunctionalRole&role=Erythronate-4-phosphate%20dehydrogenase%20(EC%201.1.1.290)&subsystem_name=Pyridoxin_(Vitamin_B6)_Biosynthesis) |
| Cofactors, Vitamins, Prosthetic Groups, Pigments | Pyridoxine | [Pyridoxin (Vitamin B6) Biosynthesis](https://rast.nmpdr.org/seedviewer.cgi?page=Subsystems&subsystem=Pyridoxin_(Vitamin_B6)_Biosynthesis&organism=549.408) | [Pyridoxine 5'-phosphate synthase (EC 2.6.99.2)](https://rast.nmpdr.org/seedviewer.cgi?page=FunctionalRole&role=Pyridoxine%205) |
| Cofactors, Vitamins, Prosthetic Groups, Pigments | Pyridoxine | [Pyridoxin (Vitamin B6) Biosynthesis](https://rast.nmpdr.org/seedviewer.cgi?page=Subsystems&subsystem=Pyridoxin_(Vitamin_B6)_Biosynthesis&organism=549.408) | [Pyridoxal kinase (EC 2.7.1.35)](https://rast.nmpdr.org/seedviewer.cgi?page=FunctionalRole&role=Pyridoxal%20kinase%20(EC%202.7.1.35)&subsystem_name=Pyridoxin_(Vitamin_B6)_Biosynthesis) |
| Cofactors, Vitamins, Prosthetic Groups, Pigments | Pyridoxine | [Pyridoxin (Vitamin B6) Biosynthesis](https://rast.nmpdr.org/seedviewer.cgi?page=Subsystems&subsystem=Pyridoxin_(Vitamin_B6)_Biosynthesis&organism=549.408) | [D-erythrose-4-phosphate dehydrogenase (EC 1.2.1.72)](https://rast.nmpdr.org/seedviewer.cgi?page=FunctionalRole&role=D-erythrose-4-phosphate%20dehydrogenase%20(EC%201.2.1.72)&subsystem_name=Pyridoxin_(Vitamin_B6)_Biosynthesis) |
| Cofactors, Vitamins, Prosthetic Groups, Pigments | Pyridoxine | [Pyridoxin (Vitamin B6) Biosynthesis](https://rast.nmpdr.org/seedviewer.cgi?page=Subsystems&subsystem=Pyridoxin_(Vitamin_B6)_Biosynthesis&organism=549.408) | [Phosphoserine aminotransferase (EC 2.6.1.52)](https://rast.nmpdr.org/seedviewer.cgi?page=FunctionalRole&role=Phosphoserine%20aminotransferase%20(EC%202.6.1.52)&subsystem_name=Pyridoxin_(Vitamin_B6)_Biosynthesis) |
| Cofactors, Vitamins, Prosthetic Groups, Pigments | Pyridoxine | [Pyridoxin (Vitamin B6) Biosynthesis](https://rast.nmpdr.org/seedviewer.cgi?page=Subsystems&subsystem=Pyridoxin_(Vitamin_B6)_Biosynthesis&organism=549.408) | [1-deoxy-D-xylulose 5-phosphate synthase (EC 2.2.1.7)](https://rast.nmpdr.org/seedviewer.cgi?page=FunctionalRole&role=1-deoxy-D-xylulose%205-phosphate%20synthase%20(EC%202.2.1.7)&subsystem_name=Pyridoxin_(Vitamin_B6)_Biosynthesis) |
| Cofactors, Vitamins, Prosthetic Groups, Pigments | Pyridoxine | [Pyridoxin (Vitamin B6) Biosynthesis](https://rast.nmpdr.org/seedviewer.cgi?page=Subsystems&subsystem=Pyridoxin_(Vitamin_B6)_Biosynthesis&organism=549.408) | [NAD-dependent glyceraldehyde-3-phosphate dehydrogenase (EC 1.2.1.12)](https://rast.nmpdr.org/seedviewer.cgi?page=FunctionalRole&role=NAD-dependent%20glyceraldehyde-3-phosphate%20dehydrogenase%20(EC%201.2.1.12)&subsystem_name=Pyridoxin_(Vitamin_B6)_Biosynthesis) |
| Cofactors, Vitamins, Prosthetic Groups, Pigments | NAD and NADP | [NAD and NADP cofactor biosynthesis global](https://rast.nmpdr.org/seedviewer.cgi?page=Subsystems&subsystem=NAD_and_NADP_cofactor_biosynthesis_global&organism=549.408) | [Quinolinate synthetase (EC 2.5.1.72)](https://rast.nmpdr.org/seedviewer.cgi?page=FunctionalRole&role=Quinolinate%20synthetase%20(EC%202.5.1.72)&subsystem_name=NAD_and_NADP_cofactor_biosynthesis_global) |
| Cofactors, Vitamins, Prosthetic Groups, Pigments | NAD and NADP | [NAD and NADP cofactor biosynthesis global](https://rast.nmpdr.org/seedviewer.cgi?page=Subsystems&subsystem=NAD_and_NADP_cofactor_biosynthesis_global&organism=549.408) | [Nicotinate phosphoribosyltransferase (EC 2.4.2.11)](https://rast.nmpdr.org/seedviewer.cgi?page=FunctionalRole&role=Nicotinate%20phosphoribosyltransferase%20(EC%202.4.2.11)&subsystem_name=NAD_and_NADP_cofactor_biosynthesis_global) |
| Cofactors, Vitamins, Prosthetic Groups, Pigments | NAD and NADP | [NAD and NADP cofactor biosynthesis global](https://rast.nmpdr.org/seedviewer.cgi?page=Subsystems&subsystem=NAD_and_NADP_cofactor_biosynthesis_global&organism=549.408) | [Nicotinamidase/isochorismatase family protein](https://rast.nmpdr.org/seedviewer.cgi?page=FunctionalRole&role=Nicotinamidase/isochorismatase%20family%20protein&subsystem_name=NAD_and_NADP_cofactor_biosynthesis_global) |
| Cofactors, Vitamins, Prosthetic Groups, Pigments | NAD and NADP | [NAD and NADP cofactor biosynthesis global](https://rast.nmpdr.org/seedviewer.cgi?page=Subsystems&subsystem=NAD_and_NADP_cofactor_biosynthesis_global&organism=549.408) | [Ribosylnicotinamide kinase (EC 2.7.1.22)](https://rast.nmpdr.org/seedviewer.cgi?page=FunctionalRole&role=Ribosylnicotinamide%20kinase%20(EC%202.7.1.22)&subsystem_name=NAD_and_NADP_cofactor_biosynthesis_global) |
| Cofactors, Vitamins, Prosthetic Groups, Pigments | NAD and NADP | [NAD and NADP cofactor biosynthesis global](https://rast.nmpdr.org/seedviewer.cgi?page=Subsystems&subsystem=NAD_and_NADP_cofactor_biosynthesis_global&organism=549.408) | [Quinolinate phosphoribosyltransferase [decarboxylating] (EC 2.4.2.19)](https://rast.nmpdr.org/seedviewer.cgi?page=FunctionalRole&role=Quinolinate%20phosphoribosyltransferase%20%5bdecarboxylating%5d%20(EC%202.4.2.19)&subsystem_name=NAD_and_NADP_cofactor_biosynthesis_global) |
| Cofactors, Vitamins, Prosthetic Groups, Pigments | NAD and NADP | [NAD and NADP cofactor biosynthesis global](https://rast.nmpdr.org/seedviewer.cgi?page=Subsystems&subsystem=NAD_and_NADP_cofactor_biosynthesis_global&organism=549.408) | [NAD kinase (EC 2.7.1.23)](https://rast.nmpdr.org/seedviewer.cgi?page=FunctionalRole&role=NAD%20kinase%20(EC%202.7.1.23)&subsystem_name=NAD_and_NADP_cofactor_biosynthesis_global) |
| Cofactors, Vitamins, Prosthetic Groups, Pigments | NAD and NADP | [NAD and NADP cofactor biosynthesis global](https://rast.nmpdr.org/seedviewer.cgi?page=Subsystems&subsystem=NAD_and_NADP_cofactor_biosynthesis_global&organism=549.408) | [ADP-ribose pyrophosphatase (EC 3.6.1.13)](https://rast.nmpdr.org/seedviewer.cgi?page=FunctionalRole&role=ADP-ribose%20pyrophosphatase%20(EC%203.6.1.13)&subsystem_name=NAD_and_NADP_cofactor_biosynthesis_global) |
| Cofactors, Vitamins, Prosthetic Groups, Pigments | NAD and NADP | [NAD and NADP cofactor biosynthesis global](https://rast.nmpdr.org/seedviewer.cgi?page=Subsystems&subsystem=NAD_and_NADP_cofactor_biosynthesis_global&organism=549.408) | [NMN 5'-nucleotidase, extracellular (EC 3.1.3.5)](https://rast.nmpdr.org/seedviewer.cgi?page=FunctionalRole&role=NMN%205) |
| Cofactors, Vitamins, Prosthetic Groups, Pigments | NAD and NADP | [NAD and NADP cofactor biosynthesis global](https://rast.nmpdr.org/seedviewer.cgi?page=Subsystems&subsystem=NAD_and_NADP_cofactor_biosynthesis_global&organism=549.408) | [NadR transcriptional regulator](https://rast.nmpdr.org/seedviewer.cgi?page=FunctionalRole&role=NadR%20transcriptional%20regulator&subsystem_name=NAD_and_NADP_cofactor_biosynthesis_global) |
| Cofactors, Vitamins, Prosthetic Groups, Pigments | NAD and NADP | [NAD and NADP cofactor biosynthesis global](https://rast.nmpdr.org/seedviewer.cgi?page=Subsystems&subsystem=NAD_and_NADP_cofactor_biosynthesis_global&organism=549.408) | [NAD synthetase (EC 6.3.1.5)](https://rast.nmpdr.org/seedviewer.cgi?page=FunctionalRole&role=NAD%20synthetase%20(EC%206.3.1.5)&subsystem_name=NAD_and_NADP_cofactor_biosynthesis_global) |
| Cofactors, Vitamins, Prosthetic Groups, Pigments | NAD and NADP | [NAD and NADP cofactor biosynthesis global](https://rast.nmpdr.org/seedviewer.cgi?page=Subsystems&subsystem=NAD_and_NADP_cofactor_biosynthesis_global&organism=549.408) | [L-aspartate oxidase (EC 1.4.3.16)](https://rast.nmpdr.org/seedviewer.cgi?page=FunctionalRole&role=L-aspartate%20oxidase%20(EC%201.4.3.16)&subsystem_name=NAD_and_NADP_cofactor_biosynthesis_global) |
| Cofactors, Vitamins, Prosthetic Groups, Pigments | NAD and NADP | [NAD and NADP cofactor biosynthesis global](https://rast.nmpdr.org/seedviewer.cgi?page=Subsystems&subsystem=NAD_and_NADP_cofactor_biosynthesis_global&organism=549.408) | [Ribosyl nicotinamide transporter, PnuC-like](https://rast.nmpdr.org/seedviewer.cgi?page=FunctionalRole&role=Ribosyl%20nicotinamide%20transporter,%20PnuC-like&subsystem_name=NAD_and_NADP_cofactor_biosynthesis_global) |
| Cofactors, Vitamins, Prosthetic Groups, Pigments | NAD and NADP | [NAD and NADP cofactor biosynthesis global](https://rast.nmpdr.org/seedviewer.cgi?page=Subsystems&subsystem=NAD_and_NADP_cofactor_biosynthesis_global&organism=549.408) | [Nicotinate-nucleotide adenylyltransferase (EC 2.7.7.18)](https://rast.nmpdr.org/seedviewer.cgi?page=FunctionalRole&role=Nicotinate-nucleotide%20adenylyltransferase%20(EC%202.7.7.18)&subsystem_name=NAD_and_NADP_cofactor_biosynthesis_global) |
| Cofactors, Vitamins, Prosthetic Groups, Pigments | NAD and NADP | [NAD and NADP cofactor biosynthesis global](https://rast.nmpdr.org/seedviewer.cgi?page=Subsystems&subsystem=NAD_and_NADP_cofactor_biosynthesis_global&organism=549.408) | [Nicotinamidase (EC 3.5.1.19)](https://rast.nmpdr.org/seedviewer.cgi?page=FunctionalRole&role=Nicotinamidase%20(EC%203.5.1.19)&subsystem_name=NAD_and_NADP_cofactor_biosynthesis_global) |
| Cofactors, Vitamins, Prosthetic Groups, Pigments | NAD and NADP | [NAD and NADP cofactor biosynthesis global](https://rast.nmpdr.org/seedviewer.cgi?page=Subsystems&subsystem=NAD_and_NADP_cofactor_biosynthesis_global&organism=549.408) | [Nicotinamide-nucleotide adenylyltransferase, NadR family (EC 2.7.7.1)](https://rast.nmpdr.org/seedviewer.cgi?page=FunctionalRole&role=Nicotinamide-nucleotide%20adenylyltransferase,%20NadR%20family%20(EC%202.7.7.1)&subsystem_name=NAD_and_NADP_cofactor_biosynthesis_global) |
| Cofactors, Vitamins, Prosthetic Groups, Pigments | Folate and pterines | [Folate biosynthesis cluster](https://rast.nmpdr.org/seedviewer.cgi?page=Subsystems&subsystem=Folate_biosynthesis_cluster&organism=549.408) | [Dihydropteroate synthase (EC 2.5.1.15)](https://rast.nmpdr.org/seedviewer.cgi?page=FunctionalRole&role=Dihydropteroate%20synthase%20(EC%202.5.1.15)&subsystem_name=Folate_biosynthesis_cluster) |
| Cofactors, Vitamins, Prosthetic Groups, Pigments | Folate and pterines | [Folate biosynthesis cluster](https://rast.nmpdr.org/seedviewer.cgi?page=Subsystems&subsystem=Folate_biosynthesis_cluster&organism=549.408) | [tRNA(Ile)-lysidine synthetase (EC 6.3.4.19)](https://rast.nmpdr.org/seedviewer.cgi?page=FunctionalRole&role=tRNA(Ile)-lysidine%20synthetase%20(EC%206.3.4.19)&subsystem_name=Folate_biosynthesis_cluster) |
| Cofactors, Vitamins, Prosthetic Groups, Pigments | Folate and pterines | [Folate biosynthesis cluster](https://rast.nmpdr.org/seedviewer.cgi?page=Subsystems&subsystem=Folate_biosynthesis_cluster&organism=549.408) | [Hypoxanthine-guanine phosphoribosyltransferase (EC 2.4.2.8)](https://rast.nmpdr.org/seedviewer.cgi?page=FunctionalRole&role=Hypoxanthine-guanine%20phosphoribosyltransferase%20(EC%202.4.2.8)&subsystem_name=Folate_biosynthesis_cluster) |
| Cofactors, Vitamins, Prosthetic Groups, Pigments | Folate and pterines | [Folate biosynthesis cluster](https://rast.nmpdr.org/seedviewer.cgi?page=Subsystems&subsystem=Folate_biosynthesis_cluster&organism=549.408) | [Aspartate 1-decarboxylase (EC 4.1.1.11)](https://rast.nmpdr.org/seedviewer.cgi?page=FunctionalRole&role=Aspartate%201-decarboxylase%20(EC%204.1.1.11)&subsystem_name=Folate_biosynthesis_cluster) |
| Cofactors, Vitamins, Prosthetic Groups, Pigments | Folate and pterines | [Folate biosynthesis cluster](https://rast.nmpdr.org/seedviewer.cgi?page=Subsystems&subsystem=Folate_biosynthesis_cluster&organism=549.408) | [Cell division protein FtsH (EC 3.4.24.-)](https://rast.nmpdr.org/seedviewer.cgi?page=FunctionalRole&role=Cell%20division%20protein%20FtsH%20(EC%203.4.24.-)&subsystem_name=Folate_biosynthesis_cluster) |
| Cofactors, Vitamins, Prosthetic Groups, Pigments | Folate and pterines | [Folate biosynthesis cluster](https://rast.nmpdr.org/seedviewer.cgi?page=Subsystems&subsystem=Folate_biosynthesis_cluster&organism=549.408) | [GTP cyclohydrolase I (EC 3.5.4.16) type 1](https://rast.nmpdr.org/seedviewer.cgi?page=FunctionalRole&role=GTP%20cyclohydrolase%20I%20(EC%203.5.4.16)%20type%201&subsystem_name=Folate_biosynthesis_cluster) |
| Cofactors, Vitamins, Prosthetic Groups, Pigments | Folate and pterines | [Folate biosynthesis cluster](https://rast.nmpdr.org/seedviewer.cgi?page=Subsystems&subsystem=Folate_biosynthesis_cluster&organism=549.408) | [Pantoate--beta-alanine ligase (EC 6.3.2.1)](https://rast.nmpdr.org/seedviewer.cgi?page=FunctionalRole&role=Pantoate--beta-alanine%20ligase%20(EC%206.3.2.1)&subsystem_name=Folate_biosynthesis_cluster) |
| Cofactors, Vitamins, Prosthetic Groups, Pigments | Folate and pterines | [Folate biosynthesis cluster](https://rast.nmpdr.org/seedviewer.cgi?page=Subsystems&subsystem=Folate_biosynthesis_cluster&organism=549.408) | [2-amino-4-hydroxy-6-hydroxymethyldihydropteridine pyrophosphokinase (EC 2.7.6.3)](https://rast.nmpdr.org/seedviewer.cgi?page=FunctionalRole&role=2-amino-4-hydroxy-6-hydroxymethyldihydropteridine%20pyrophosphokinase%20(EC%202.7.6.3)&subsystem_name=Folate_biosynthesis_cluster) |
| Cofactors, Vitamins, Prosthetic Groups, Pigments | Folate and pterines | [Folate biosynthesis cluster](https://rast.nmpdr.org/seedviewer.cgi?page=Subsystems&subsystem=Folate_biosynthesis_cluster&organism=549.408) | [Dihydroneopterin aldolase (EC 4.1.2.25)](https://rast.nmpdr.org/seedviewer.cgi?page=FunctionalRole&role=Dihydroneopterin%20aldolase%20(EC%204.1.2.25)&subsystem_name=Folate_biosynthesis_cluster) |
| Cofactors, Vitamins, Prosthetic Groups, Pigments | Folate and pterines | [p-Aminobenzoyl-Glutamate Utilization](https://rast.nmpdr.org/seedviewer.cgi?page=Subsystems&subsystem=p-Aminobenzoyl-Glutamate_Utilization&organism=549.408) | [Catalyzes the cleavage of p-aminobenzoyl-glutamate to p-aminobenzoate and glutamate, subunit A](https://rast.nmpdr.org/seedviewer.cgi?page=FunctionalRole&role=Catalyzes%20the%20cleavage%20of%20p-aminobenzoyl-glutamate%20to%20p-aminobenzoate%20and%20glutamate,%20subunit%20A&subsystem_name=p-Aminobenzoyl-Glutamate_Utilization) |
| Cofactors, Vitamins, Prosthetic Groups, Pigments | Folate and pterines | [p-Aminobenzoyl-Glutamate Utilization](https://rast.nmpdr.org/seedviewer.cgi?page=Subsystems&subsystem=p-Aminobenzoyl-Glutamate_Utilization&organism=549.408) | [Regulatory protein (induces abgABT, used to catabolize p-aminobenzoyl-glutamate)](https://rast.nmpdr.org/seedviewer.cgi?page=FunctionalRole&role=Regulatory%20protein%20(induces%20abgABT,%20used%20to%20catabolize%20p-aminobenzoyl-glutamate)&subsystem_name=p-Aminobenzoyl-Glutamate_Utilization) |
| Cofactors, Vitamins, Prosthetic Groups, Pigments | Folate and pterines | [p-Aminobenzoyl-Glutamate Utilization](https://rast.nmpdr.org/seedviewer.cgi?page=Subsystems&subsystem=p-Aminobenzoyl-Glutamate_Utilization&organism=549.408) | [Aminobenzoyl-glutamate transport protein](https://rast.nmpdr.org/seedviewer.cgi?page=FunctionalRole&role=Aminobenzoyl-glutamate%20transport%20protein&subsystem_name=p-Aminobenzoyl-Glutamate_Utilization) |
| Cofactors, Vitamins, Prosthetic Groups, Pigments | Folate and pterines | [p-Aminobenzoyl-Glutamate Utilization](https://rast.nmpdr.org/seedviewer.cgi?page=Subsystems&subsystem=p-Aminobenzoyl-Glutamate_Utilization&organism=549.408) | [Catalyzes the cleavage of p-aminobenzoyl-glutamate to p-aminobenzoate and glutamate, subunit B](https://rast.nmpdr.org/seedviewer.cgi?page=FunctionalRole&role=Catalyzes%20the%20cleavage%20of%20p-aminobenzoyl-glutamate%20to%20p-aminobenzoate%20and%20glutamate,%20subunit%20B&subsystem_name=p-Aminobenzoyl-Glutamate_Utilization) |
| Cofactors, Vitamins, Prosthetic Groups, Pigments | Folate and pterines | [Molybdenum cofactor biosynthesis](https://rast.nmpdr.org/seedviewer.cgi?page=Subsystems&subsystem=Molybdenum_cofactor_biosynthesis&organism=549.408) | [Molybdenum transport ATP-binding protein ModC (TC 3.A.1.8.1)](https://rast.nmpdr.org/seedviewer.cgi?page=FunctionalRole&role=Molybdenum%20transport%20ATP-binding%20protein%20ModC%20(TC%203.A.1.8.1)&subsystem_name=Molybdenum_cofactor_biosynthesis) |
| Cofactors, Vitamins, Prosthetic Groups, Pigments | Folate and pterines | [Molybdenum cofactor biosynthesis](https://rast.nmpdr.org/seedviewer.cgi?page=Subsystems&subsystem=Molybdenum_cofactor_biosynthesis&organism=549.408) | [Molybdenum cofactor biosynthesis protein MoaC](https://rast.nmpdr.org/seedviewer.cgi?page=FunctionalRole&role=Molybdenum%20cofactor%20biosynthesis%20protein%20MoaC&subsystem_name=Molybdenum_cofactor_biosynthesis) |
| Cofactors, Vitamins, Prosthetic Groups, Pigments | Folate and pterines | [Molybdenum cofactor biosynthesis](https://rast.nmpdr.org/seedviewer.cgi?page=Subsystems&subsystem=Molybdenum_cofactor_biosynthesis&organism=549.408) | [Molybdenum cofactor biosynthesis protein MoaE](https://rast.nmpdr.org/seedviewer.cgi?page=FunctionalRole&role=Molybdenum%20cofactor%20biosynthesis%20protein%20MoaE&subsystem_name=Molybdenum_cofactor_biosynthesis) |
| Cofactors, Vitamins, Prosthetic Groups, Pigments | Folate and pterines | [Molybdenum cofactor biosynthesis](https://rast.nmpdr.org/seedviewer.cgi?page=Subsystems&subsystem=Molybdenum_cofactor_biosynthesis&organism=549.408) | [Molybdopterin-guanine dinucleotide biosynthesis protein MobA](https://rast.nmpdr.org/seedviewer.cgi?page=FunctionalRole&role=Molybdopterin-guanine%20dinucleotide%20biosynthesis%20protein%20MobA&subsystem_name=Molybdenum_cofactor_biosynthesis) |
| Cofactors, Vitamins, Prosthetic Groups, Pigments | Folate and pterines | [Molybdenum cofactor biosynthesis](https://rast.nmpdr.org/seedviewer.cgi?page=Subsystems&subsystem=Molybdenum_cofactor_biosynthesis&organism=549.408) | [Molybdenum cofactor biosynthesis protein MoaD](https://rast.nmpdr.org/seedviewer.cgi?page=FunctionalRole&role=Molybdenum%20cofactor%20biosynthesis%20protein%20MoaD&subsystem_name=Molybdenum_cofactor_biosynthesis) |
| Cofactors, Vitamins, Prosthetic Groups, Pigments | Folate and pterines | [Molybdenum cofactor biosynthesis](https://rast.nmpdr.org/seedviewer.cgi?page=Subsystems&subsystem=Molybdenum_cofactor_biosynthesis&organism=549.408) | [GTP cyclohydrolase I (EC 3.5.4.16) type 1](https://rast.nmpdr.org/seedviewer.cgi?page=FunctionalRole&role=GTP%20cyclohydrolase%20I%20(EC%203.5.4.16)%20type%201&subsystem_name=Molybdenum_cofactor_biosynthesis) |
| Cofactors, Vitamins, Prosthetic Groups, Pigments | Folate and pterines | [Molybdenum cofactor biosynthesis](https://rast.nmpdr.org/seedviewer.cgi?page=Subsystems&subsystem=Molybdenum_cofactor_biosynthesis&organism=549.408) | [Molybdenum cofactor biosynthesis protein MoaA](https://rast.nmpdr.org/seedviewer.cgi?page=FunctionalRole&role=Molybdenum%20cofactor%20biosynthesis%20protein%20MoaA&subsystem_name=Molybdenum_cofactor_biosynthesis) |
| Cofactors, Vitamins, Prosthetic Groups, Pigments | Folate and pterines | [Molybdenum cofactor biosynthesis](https://rast.nmpdr.org/seedviewer.cgi?page=Subsystems&subsystem=Molybdenum_cofactor_biosynthesis&organism=549.408) | [Molybdopterin-guanine dinucleotide biosynthesis protein MobB](https://rast.nmpdr.org/seedviewer.cgi?page=FunctionalRole&role=Molybdopterin-guanine%20dinucleotide%20biosynthesis%20protein%20MobB&subsystem_name=Molybdenum_cofactor_biosynthesis) |
| Cofactors, Vitamins, Prosthetic Groups, Pigments | Folate and pterines | [Molybdenum cofactor biosynthesis](https://rast.nmpdr.org/seedviewer.cgi?page=Subsystems&subsystem=Molybdenum_cofactor_biosynthesis&organism=549.408) | [Molybdopterin biosynthesis protein MoeB](https://rast.nmpdr.org/seedviewer.cgi?page=FunctionalRole&role=Molybdopterin%20biosynthesis%20protein%20MoeB&subsystem_name=Molybdenum_cofactor_biosynthesis) |
| Cofactors, Vitamins, Prosthetic Groups, Pigments | Folate and pterines | [Molybdenum cofactor biosynthesis](https://rast.nmpdr.org/seedviewer.cgi?page=Subsystems&subsystem=Molybdenum_cofactor_biosynthesis&organism=549.408) | [Molybdenum transport system permease protein ModB (TC 3.A.1.8.1)](https://rast.nmpdr.org/seedviewer.cgi?page=FunctionalRole&role=Molybdenum%20transport%20system%20permease%20protein%20ModB%20(TC%203.A.1.8.1)&subsystem_name=Molybdenum_cofactor_biosynthesis) |
| Cofactors, Vitamins, Prosthetic Groups, Pigments | Folate and pterines | [Molybdenum cofactor biosynthesis](https://rast.nmpdr.org/seedviewer.cgi?page=Subsystems&subsystem=Molybdenum_cofactor_biosynthesis&organism=549.408) | [DNA-binding domain of ModE](https://rast.nmpdr.org/seedviewer.cgi?page=FunctionalRole&role=DNA-binding%20domain%20of%20ModE&subsystem_name=Molybdenum_cofactor_biosynthesis) |
| Cofactors, Vitamins, Prosthetic Groups, Pigments | Folate and pterines | [Molybdenum cofactor biosynthesis](https://rast.nmpdr.org/seedviewer.cgi?page=Subsystems&subsystem=Molybdenum_cofactor_biosynthesis&organism=549.408) | [Molybdopterin biosynthesis protein MoeA](https://rast.nmpdr.org/seedviewer.cgi?page=FunctionalRole&role=Molybdopterin%20biosynthesis%20protein%20MoeA&subsystem_name=Molybdenum_cofactor_biosynthesis) |
| Cofactors, Vitamins, Prosthetic Groups, Pigments | Folate and pterines | [Molybdenum cofactor biosynthesis](https://rast.nmpdr.org/seedviewer.cgi?page=Subsystems&subsystem=Molybdenum_cofactor_biosynthesis&organism=549.408) | [GTP cyclohydrolase II (EC 3.5.4.25)](https://rast.nmpdr.org/seedviewer.cgi?page=FunctionalRole&role=GTP%20cyclohydrolase%20II%20(EC%203.5.4.25)&subsystem_name=Molybdenum_cofactor_biosynthesis) |
| Cofactors, Vitamins, Prosthetic Groups, Pigments | Folate and pterines | [Molybdenum cofactor biosynthesis](https://rast.nmpdr.org/seedviewer.cgi?page=Subsystems&subsystem=Molybdenum_cofactor_biosynthesis&organism=549.408) | [Xanthine and CO dehydrogenases maturation factor, XdhC/CoxF family](https://rast.nmpdr.org/seedviewer.cgi?page=FunctionalRole&role=Xanthine%20and%20CO%20dehydrogenases%20maturation%20factor,%20XdhC/CoxF%20family&subsystem_name=Molybdenum_cofactor_biosynthesis) |
| Cofactors, Vitamins, Prosthetic Groups, Pigments | Folate and pterines | [Molybdenum cofactor biosynthesis](https://rast.nmpdr.org/seedviewer.cgi?page=Subsystems&subsystem=Molybdenum_cofactor_biosynthesis&organism=549.408) | [Molybdenum ABC transporter, periplasmic molybdenum-binding protein ModA (TC 3.A.1.8.1)](https://rast.nmpdr.org/seedviewer.cgi?page=FunctionalRole&role=Molybdenum%20ABC%20transporter,%20periplasmic%20molybdenum-binding%20protein%20ModA%20(TC%203.A.1.8.1)&subsystem_name=Molybdenum_cofactor_biosynthesis) |
| Cofactors, Vitamins, Prosthetic Groups, Pigments | Folate and pterines | [Molybdenum cofactor biosynthesis](https://rast.nmpdr.org/seedviewer.cgi?page=Subsystems&subsystem=Molybdenum_cofactor_biosynthesis&organism=549.408) | [Molybdenum cofactor biosynthesis protein MoaB](https://rast.nmpdr.org/seedviewer.cgi?page=FunctionalRole&role=Molybdenum%20cofactor%20biosynthesis%20protein%20MoaB&subsystem_name=Molybdenum_cofactor_biosynthesis) |
| Cofactors, Vitamins, Prosthetic Groups, Pigments | Folate and pterines | [Molybdenum cofactor biosynthesis](https://rast.nmpdr.org/seedviewer.cgi?page=Subsystems&subsystem=Molybdenum_cofactor_biosynthesis&organism=549.408) | [Molybdate-binding domain of ModE](https://rast.nmpdr.org/seedviewer.cgi?page=FunctionalRole&role=Molybdate-binding%20domain%20of%20ModE&subsystem_name=Molybdenum_cofactor_biosynthesis) |
| Cofactors, Vitamins, Prosthetic Groups, Pigments | Folate and pterines | [Molybdenum cofactor biosynthesis](https://rast.nmpdr.org/seedviewer.cgi?page=Subsystems&subsystem=Molybdenum_cofactor_biosynthesis&organism=549.408) | [Molybdopterin biosynthesis molybdochelatase MogA](https://rast.nmpdr.org/seedviewer.cgi?page=FunctionalRole&role=Molybdopterin%20biosynthesis%20molybdochelatase%20MogA&subsystem_name=Molybdenum_cofactor_biosynthesis) |
| Cofactors, Vitamins, Prosthetic Groups, Pigments | Folate and pterines | [Folate Biosynthesis](https://rast.nmpdr.org/seedviewer.cgi?page=Subsystems&subsystem=Folate_Biosynthesis&organism=549.408) | [Aminodeoxychorismate lyase (EC 4.1.3.38)](https://rast.nmpdr.org/seedviewer.cgi?page=FunctionalRole&role=Aminodeoxychorismate%20lyase%20(EC%204.1.3.38)&subsystem_name=Folate_Biosynthesis) |
| Cofactors, Vitamins, Prosthetic Groups, Pigments | Folate and pterines | [Folate Biosynthesis](https://rast.nmpdr.org/seedviewer.cgi?page=Subsystems&subsystem=Folate_Biosynthesis&organism=549.408) | [Folylpolyglutamate synthase (EC 6.3.2.17)](https://rast.nmpdr.org/seedviewer.cgi?page=FunctionalRole&role=Folylpolyglutamate%20synthase%20(EC%206.3.2.17)&subsystem_name=Folate_Biosynthesis) |
| Cofactors, Vitamins, Prosthetic Groups, Pigments | Folate and pterines | [Folate Biosynthesis](https://rast.nmpdr.org/seedviewer.cgi?page=Subsystems&subsystem=Folate_Biosynthesis&organism=549.408) | [Dihydropteroate synthase (EC 2.5.1.15)](https://rast.nmpdr.org/seedviewer.cgi?page=FunctionalRole&role=Dihydropteroate%20synthase%20(EC%202.5.1.15)&subsystem_name=Folate_Biosynthesis) |
| Cofactors, Vitamins, Prosthetic Groups, Pigments | Folate and pterines | [Folate Biosynthesis](https://rast.nmpdr.org/seedviewer.cgi?page=Subsystems&subsystem=Folate_Biosynthesis&organism=549.408) | [5-formyltetrahydrofolate cyclo-ligase (EC 6.3.3.2)](https://rast.nmpdr.org/seedviewer.cgi?page=FunctionalRole&role=5-formyltetrahydrofolate%20cyclo-ligase%20(EC%206.3.3.2)&subsystem_name=Folate_Biosynthesis) |
| Cofactors, Vitamins, Prosthetic Groups, Pigments | Folate and pterines | [Folate Biosynthesis](https://rast.nmpdr.org/seedviewer.cgi?page=Subsystems&subsystem=Folate_Biosynthesis&organism=549.408) | [Dihydrofolate reductase (EC 1.5.1.3)](https://rast.nmpdr.org/seedviewer.cgi?page=FunctionalRole&role=Dihydrofolate%20reductase%20(EC%201.5.1.3)&subsystem_name=Folate_Biosynthesis) |
| Cofactors, Vitamins, Prosthetic Groups, Pigments | Folate and pterines | [Folate Biosynthesis](https://rast.nmpdr.org/seedviewer.cgi?page=Subsystems&subsystem=Folate_Biosynthesis&organism=549.408) | [Dihydrofolate synthase (EC 6.3.2.12)](https://rast.nmpdr.org/seedviewer.cgi?page=FunctionalRole&role=Dihydrofolate%20synthase%20(EC%206.3.2.12)&subsystem_name=Folate_Biosynthesis) |
| Cofactors, Vitamins, Prosthetic Groups, Pigments | Folate and pterines | [Folate Biosynthesis](https://rast.nmpdr.org/seedviewer.cgi?page=Subsystems&subsystem=Folate_Biosynthesis&organism=549.408) | [GTP cyclohydrolase I (EC 3.5.4.16) type 1](https://rast.nmpdr.org/seedviewer.cgi?page=FunctionalRole&role=GTP%20cyclohydrolase%20I%20(EC%203.5.4.16)%20type%201&subsystem_name=Folate_Biosynthesis) |
| Cofactors, Vitamins, Prosthetic Groups, Pigments | Folate and pterines | [Folate Biosynthesis](https://rast.nmpdr.org/seedviewer.cgi?page=Subsystems&subsystem=Folate_Biosynthesis&organism=549.408) | [Para-aminobenzoate synthase, aminase component (EC 2.6.1.85)](https://rast.nmpdr.org/seedviewer.cgi?page=FunctionalRole&role=Para-aminobenzoate%20synthase,%20aminase%20component%20(EC%202.6.1.85)&subsystem_name=Folate_Biosynthesis) |
| Cofactors, Vitamins, Prosthetic Groups, Pigments | Folate and pterines | [Folate Biosynthesis](https://rast.nmpdr.org/seedviewer.cgi?page=Subsystems&subsystem=Folate_Biosynthesis&organism=549.408) | [Thymidylate synthase (EC 2.1.1.45)](https://rast.nmpdr.org/seedviewer.cgi?page=FunctionalRole&role=Thymidylate%20synthase%20(EC%202.1.1.45)&subsystem_name=Folate_Biosynthesis) |
| Cofactors, Vitamins, Prosthetic Groups, Pigments | Folate and pterines | [Folate Biosynthesis](https://rast.nmpdr.org/seedviewer.cgi?page=Subsystems&subsystem=Folate_Biosynthesis&organism=549.408) | [2-amino-4-hydroxy-6-hydroxymethyldihydropteridine pyrophosphokinase (EC 2.7.6.3)](https://rast.nmpdr.org/seedviewer.cgi?page=FunctionalRole&role=2-amino-4-hydroxy-6-hydroxymethyldihydropteridine%20pyrophosphokinase%20(EC%202.7.6.3)&subsystem_name=Folate_Biosynthesis) |
| Cofactors, Vitamins, Prosthetic Groups, Pigments | Folate and pterines | [Folate Biosynthesis](https://rast.nmpdr.org/seedviewer.cgi?page=Subsystems&subsystem=Folate_Biosynthesis&organism=549.408) | [Dihydroneopterin aldolase (EC 4.1.2.25)](https://rast.nmpdr.org/seedviewer.cgi?page=FunctionalRole&role=Dihydroneopterin%20aldolase%20(EC%204.1.2.25)&subsystem_name=Folate_Biosynthesis) |
| Cofactors, Vitamins, Prosthetic Groups, Pigments | Folate and pterines | [Folate Biosynthesis](https://rast.nmpdr.org/seedviewer.cgi?page=Subsystems&subsystem=Folate_Biosynthesis&organism=549.408) | [Para-aminobenzoate synthase, amidotransferase component (EC 2.6.1.85)](https://rast.nmpdr.org/seedviewer.cgi?page=FunctionalRole&role=Para-aminobenzoate%20synthase,%20amidotransferase%20component%20(EC%202.6.1.85)&subsystem_name=Folate_Biosynthesis) |
| Cofactors, Vitamins, Prosthetic Groups, Pigments | Folate and pterines | [5-FCL-like protein](https://rast.nmpdr.org/seedviewer.cgi?page=Subsystems&subsystem=5-FCL-like_protein&organism=549.408) | [Alcohol dehydrogenase (EC 1.1.1.1)](https://rast.nmpdr.org/seedviewer.cgi?page=FunctionalRole&role=Alcohol%20dehydrogenase%20(EC%201.1.1.1)&subsystem_name=5-FCL-like_protein) |
| Cofactors, Vitamins, Prosthetic Groups, Pigments | Folate and pterines | [5-FCL-like protein](https://rast.nmpdr.org/seedviewer.cgi?page=Subsystems&subsystem=5-FCL-like_protein&organism=549.408) | [Dihydrofolate reductase (EC 1.5.1.3)](https://rast.nmpdr.org/seedviewer.cgi?page=FunctionalRole&role=Dihydrofolate%20reductase%20(EC%201.5.1.3)&subsystem_name=5-FCL-like_protein) |
| Cofactors, Vitamins, Prosthetic Groups, Pigments | Folate and pterines | [5-FCL-like protein](https://rast.nmpdr.org/seedviewer.cgi?page=Subsystems&subsystem=5-FCL-like_protein&organism=549.408) | [Serine hydroxymethyltransferase (EC 2.1.2.1)](https://rast.nmpdr.org/seedviewer.cgi?page=FunctionalRole&role=Serine%20hydroxymethyltransferase%20(EC%202.1.2.1)&subsystem_name=5-FCL-like_protein) |
| Cofactors, Vitamins, Prosthetic Groups, Pigments | Folate and pterines | [5-FCL-like protein](https://rast.nmpdr.org/seedviewer.cgi?page=Subsystems&subsystem=5-FCL-like_protein&organism=549.408) | [Phosphoribosylglycinamide formyltransferase (EC 2.1.2.2)](https://rast.nmpdr.org/seedviewer.cgi?page=FunctionalRole&role=Phosphoribosylglycinamide%20formyltransferase%20(EC%202.1.2.2)&subsystem_name=5-FCL-like_protein) |
| Cofactors, Vitamins, Prosthetic Groups, Pigments | Folate and pterines | [5-FCL-like protein](https://rast.nmpdr.org/seedviewer.cgi?page=Subsystems&subsystem=5-FCL-like_protein&organism=549.408) | [Succinate dehydrogenase iron-sulfur protein (EC 1.3.99.1)](https://rast.nmpdr.org/seedviewer.cgi?page=FunctionalRole&role=Succinate%20dehydrogenase%20iron-sulfur%20protein%20(EC%201.3.99.1)&subsystem_name=5-FCL-like_protein) |
| Cofactors, Vitamins, Prosthetic Groups, Pigments | Folate and pterines | [5-FCL-like protein](https://rast.nmpdr.org/seedviewer.cgi?page=Subsystems&subsystem=5-FCL-like_protein&organism=549.408) | [5,10-methylenetetrahydrofolate reductase (EC 1.5.1.20)](https://rast.nmpdr.org/seedviewer.cgi?page=FunctionalRole&role=5,10-methylenetetrahydrofolate%20reductase%20(EC%201.5.1.20)&subsystem_name=5-FCL-like_protein) |
| Cofactors, Vitamins, Prosthetic Groups, Pigments | Folate and pterines | [5-FCL-like protein](https://rast.nmpdr.org/seedviewer.cgi?page=Subsystems&subsystem=5-FCL-like_protein&organism=549.408) | [Dihydrolipoamide dehydrogenase (EC 1.8.1.4)](https://rast.nmpdr.org/seedviewer.cgi?page=FunctionalRole&role=Dihydrolipoamide%20dehydrogenase%20(EC%201.8.1.4)&subsystem_name=5-FCL-like_protein) |
| Cofactors, Vitamins, Prosthetic Groups, Pigments | Folate and pterines | [5-FCL-like protein](https://rast.nmpdr.org/seedviewer.cgi?page=Subsystems&subsystem=5-FCL-like_protein&organism=549.408) | [Hydroxyethylthiazole kinase (EC 2.7.1.50)](https://rast.nmpdr.org/seedviewer.cgi?page=FunctionalRole&role=Hydroxyethylthiazole%20kinase%20(EC%202.7.1.50)&subsystem_name=5-FCL-like_protein) |
| Cofactors, Vitamins, Prosthetic Groups, Pigments | Folate and pterines | [5-FCL-like protein](https://rast.nmpdr.org/seedviewer.cgi?page=Subsystems&subsystem=5-FCL-like_protein&organism=549.408) | [Formyltetrahydrofolate deformylase (EC 3.5.1.10)](https://rast.nmpdr.org/seedviewer.cgi?page=FunctionalRole&role=Formyltetrahydrofolate%20deformylase%20(EC%203.5.1.10)&subsystem_name=5-FCL-like_protein) |
| Cofactors, Vitamins, Prosthetic Groups, Pigments | Folate and pterines | [5-FCL-like protein](https://rast.nmpdr.org/seedviewer.cgi?page=Subsystems&subsystem=5-FCL-like_protein&organism=549.408) | [Isocitrate dehydrogenase [NADP] (EC 1.1.1.42)](https://rast.nmpdr.org/seedviewer.cgi?page=FunctionalRole&role=Isocitrate%20dehydrogenase%20%5bNADP%5d%20(EC%201.1.1.42)&subsystem_name=5-FCL-like_protein) |
| Cofactors, Vitamins, Prosthetic Groups, Pigments | Folate and pterines | [5-FCL-like protein](https://rast.nmpdr.org/seedviewer.cgi?page=Subsystems&subsystem=5-FCL-like_protein&organism=549.408) | [5-formyltetrahydrofolate cyclo-ligase (EC 6.3.3.2)](https://rast.nmpdr.org/seedviewer.cgi?page=FunctionalRole&role=5-formyltetrahydrofolate%20cyclo-ligase%20(EC%206.3.3.2)&subsystem_name=5-FCL-like_protein) |
| Cofactors, Vitamins, Prosthetic Groups, Pigments | Folate and pterines | [5-FCL-like protein](https://rast.nmpdr.org/seedviewer.cgi?page=Subsystems&subsystem=5-FCL-like_protein&organism=549.408) | [Pyruvate dehydrogenase E1 component (EC 1.2.4.1)](https://rast.nmpdr.org/seedviewer.cgi?page=FunctionalRole&role=Pyruvate%20dehydrogenase%20E1%20component%20(EC%201.2.4.1)&subsystem_name=5-FCL-like_protein) |
| Cofactors, Vitamins, Prosthetic Groups, Pigments | Folate and pterines | [5-FCL-like protein](https://rast.nmpdr.org/seedviewer.cgi?page=Subsystems&subsystem=5-FCL-like_protein&organism=549.408) | [Thiamine-monophosphate kinase (EC 2.7.4.16)](https://rast.nmpdr.org/seedviewer.cgi?page=FunctionalRole&role=Thiamine-monophosphate%20kinase%20(EC%202.7.4.16)&subsystem_name=5-FCL-like_protein) |
| Cofactors, Vitamins, Prosthetic Groups, Pigments | Folate and pterines | [5-FCL-like protein](https://rast.nmpdr.org/seedviewer.cgi?page=Subsystems&subsystem=5-FCL-like_protein&organism=549.408) | [Methylenetetrahydrofolate dehydrogenase (NADP+) (EC 1.5.1.5)](https://rast.nmpdr.org/seedviewer.cgi?page=FunctionalRole&role=Methylenetetrahydrofolate%20dehydrogenase%20(NADP+)%20(EC%201.5.1.5)&subsystem_name=5-FCL-like_protein) |
| Cofactors, Vitamins, Prosthetic Groups, Pigments | Folate and pterines | [5-FCL-like protein](https://rast.nmpdr.org/seedviewer.cgi?page=Subsystems&subsystem=5-FCL-like_protein&organism=549.408) | [Phosphoribosylaminoimidazolecarboxamide formyltransferase (EC 2.1.2.3)](https://rast.nmpdr.org/seedviewer.cgi?page=FunctionalRole&role=Phosphoribosylaminoimidazolecarboxamide%20formyltransferase%20(EC%202.1.2.3)&subsystem_name=5-FCL-like_protein) |
| Cofactors, Vitamins, Prosthetic Groups, Pigments | Folate and pterines | [5-FCL-like protein](https://rast.nmpdr.org/seedviewer.cgi?page=Subsystems&subsystem=5-FCL-like_protein&organism=549.408) | [Thiamine kinase (EC 2.7.1.89)](https://rast.nmpdr.org/seedviewer.cgi?page=FunctionalRole&role=Thiamine%20kinase%20(EC%202.7.1.89)&subsystem_name=5-FCL-like_protein) |
| Cofactors, Vitamins, Prosthetic Groups, Pigments | Folate and pterines | [5-FCL-like protein](https://rast.nmpdr.org/seedviewer.cgi?page=Subsystems&subsystem=5-FCL-like_protein&organism=549.408) | [Dihydrolipoamide acetyltransferase component of pyruvate dehydrogenase complex (EC 2.3.1.12)](https://rast.nmpdr.org/seedviewer.cgi?page=FunctionalRole&role=Dihydrolipoamide%20acetyltransferase%20component%20of%20pyruvate%20dehydrogenase%20complex%20(EC%202.3.1.12)&subsystem_name=5-FCL-like_protein) |
| Cofactors, Vitamins, Prosthetic Groups, Pigments | Folate and pterines | [5-FCL-like protein](https://rast.nmpdr.org/seedviewer.cgi?page=Subsystems&subsystem=5-FCL-like_protein&organism=549.408) | [Thiamin-phosphate pyrophosphorylase (EC 2.5.1.3)](https://rast.nmpdr.org/seedviewer.cgi?page=FunctionalRole&role=Thiamin-phosphate%20pyrophosphorylase%20(EC%202.5.1.3)&subsystem_name=5-FCL-like_protein) |
| Cofactors, Vitamins, Prosthetic Groups, Pigments | Lipoic acid | [Lipoic acid metabolism](https://rast.nmpdr.org/seedviewer.cgi?page=Subsystems&subsystem=Lipoic_acid_metabolism&organism=549.408) | [Proposed lipoate regulatory protein YbeD](https://rast.nmpdr.org/seedviewer.cgi?page=FunctionalRole&role=Proposed%20lipoate%20regulatory%20protein%20YbeD&subsystem_name=Lipoic_acid_metabolism) |
| Cofactors, Vitamins, Prosthetic Groups, Pigments | Lipoic acid | [Lipoic acid metabolism](https://rast.nmpdr.org/seedviewer.cgi?page=Subsystems&subsystem=Lipoic_acid_metabolism&organism=549.408) | [Octanoate-[acyl-carrier-protein]-protein-N-octanoyltransferase](https://rast.nmpdr.org/seedviewer.cgi?page=FunctionalRole&role=Octanoate-%5bacyl-carrier-protein%5d-protein-N-octanoyltransferase&subsystem_name=Lipoic_acid_metabolism) |
| Cofactors, Vitamins, Prosthetic Groups, Pigments | Lipoic acid | [Lipoic acid metabolism](https://rast.nmpdr.org/seedviewer.cgi?page=Subsystems&subsystem=Lipoic_acid_metabolism&organism=549.408) | [Lipoate-protein ligase A](https://rast.nmpdr.org/seedviewer.cgi?page=FunctionalRole&role=Lipoate-protein%20ligase%20A&subsystem_name=Lipoic_acid_metabolism) |
| Cofactors, Vitamins, Prosthetic Groups, Pigments | Lipoic acid | [Lipoic acid metabolism](https://rast.nmpdr.org/seedviewer.cgi?page=Subsystems&subsystem=Lipoic_acid_metabolism&organism=549.408) | [Lipoate synthase](https://rast.nmpdr.org/seedviewer.cgi?page=FunctionalRole&role=Lipoate%20synthase&subsystem_name=Lipoic_acid_metabolism) |
| Cofactors, Vitamins, Prosthetic Groups, Pigments | Coenzyme A | [Coenzyme A Biosynthesis cluster](https://rast.nmpdr.org/seedviewer.cgi?page=Subsystems&subsystem=Coenzyme_A_Biosynthesis_cluster&organism=549.408) | [3-methyl-2-oxobutanoate hydroxymethyltransferase (EC 2.1.2.11)](https://rast.nmpdr.org/seedviewer.cgi?page=FunctionalRole&role=3-methyl-2-oxobutanoate%20hydroxymethyltransferase%20(EC%202.1.2.11)&subsystem_name=Coenzyme_A_Biosynthesis_cluster) |
| Cofactors, Vitamins, Prosthetic Groups, Pigments | Coenzyme A | [Coenzyme A Biosynthesis cluster](https://rast.nmpdr.org/seedviewer.cgi?page=Subsystems&subsystem=Coenzyme_A_Biosynthesis_cluster&organism=549.408) | [Pantoate--beta-alanine ligase (EC 6.3.2.1)](https://rast.nmpdr.org/seedviewer.cgi?page=FunctionalRole&role=Pantoate--beta-alanine%20ligase%20(EC%206.3.2.1)&subsystem_name=Coenzyme_A_Biosynthesis_cluster) |
| Cofactors, Vitamins, Prosthetic Groups, Pigments | Coenzyme A | [Coenzyme A Biosynthesis cluster](https://rast.nmpdr.org/seedviewer.cgi?page=Subsystems&subsystem=Coenzyme_A_Biosynthesis_cluster&organism=549.408) | [Aspartate 1-decarboxylase (EC 4.1.1.11)](https://rast.nmpdr.org/seedviewer.cgi?page=FunctionalRole&role=Aspartate%201-decarboxylase%20(EC%204.1.1.11)&subsystem_name=Coenzyme_A_Biosynthesis_cluster) |
| Cofactors, Vitamins, Prosthetic Groups, Pigments | Coenzyme A | [Coenzyme A Biosynthesis](https://rast.nmpdr.org/seedviewer.cgi?page=Subsystems&subsystem=Coenzyme_A_Biosynthesis&organism=549.408) | [Pantothenate:Na+ symporter (TC 2.A.21.1.1)](https://rast.nmpdr.org/seedviewer.cgi?page=FunctionalRole&role=Pantothenate:Na+%20symporter%20(TC%202.A.21.1.1)&subsystem_name=Coenzyme_A_Biosynthesis) |
| Cofactors, Vitamins, Prosthetic Groups, Pigments | Coenzyme A | [Coenzyme A Biosynthesis](https://rast.nmpdr.org/seedviewer.cgi?page=Subsystems&subsystem=Coenzyme_A_Biosynthesis&organism=549.408) | [3-methyl-2-oxobutanoate hydroxymethyltransferase (EC 2.1.2.11)](https://rast.nmpdr.org/seedviewer.cgi?page=FunctionalRole&role=3-methyl-2-oxobutanoate%20hydroxymethyltransferase%20(EC%202.1.2.11)&subsystem_name=Coenzyme_A_Biosynthesis) |
| Cofactors, Vitamins, Prosthetic Groups, Pigments | Coenzyme A | [Coenzyme A Biosynthesis](https://rast.nmpdr.org/seedviewer.cgi?page=Subsystems&subsystem=Coenzyme_A_Biosynthesis&organism=549.408) | [Pantothenate kinase (EC 2.7.1.33)](https://rast.nmpdr.org/seedviewer.cgi?page=FunctionalRole&role=Pantothenate%20kinase%20(EC%202.7.1.33)&subsystem_name=Coenzyme_A_Biosynthesis) |
| Cofactors, Vitamins, Prosthetic Groups, Pigments | Coenzyme A | [Coenzyme A Biosynthesis](https://rast.nmpdr.org/seedviewer.cgi?page=Subsystems&subsystem=Coenzyme_A_Biosynthesis&organism=549.408) | [Dephospho-CoA kinase (EC 2.7.1.24)](https://rast.nmpdr.org/seedviewer.cgi?page=FunctionalRole&role=Dephospho-CoA%20kinase%20(EC%202.7.1.24)&subsystem_name=Coenzyme_A_Biosynthesis) |
| Cofactors, Vitamins, Prosthetic Groups, Pigments | Coenzyme A | [Coenzyme A Biosynthesis](https://rast.nmpdr.org/seedviewer.cgi?page=Subsystems&subsystem=Coenzyme_A_Biosynthesis&organism=549.408) | [Aspartate 1-decarboxylase (EC 4.1.1.11)](https://rast.nmpdr.org/seedviewer.cgi?page=FunctionalRole&role=Aspartate%201-decarboxylase%20(EC%204.1.1.11)&subsystem_name=Coenzyme_A_Biosynthesis) |
| Cofactors, Vitamins, Prosthetic Groups, Pigments | Coenzyme A | [Coenzyme A Biosynthesis](https://rast.nmpdr.org/seedviewer.cgi?page=Subsystems&subsystem=Coenzyme_A_Biosynthesis&organism=549.408) | [2-dehydropantoate 2-reductase (EC 1.1.1.169)](https://rast.nmpdr.org/seedviewer.cgi?page=FunctionalRole&role=2-dehydropantoate%202-reductase%20(EC%201.1.1.169)&subsystem_name=Coenzyme_A_Biosynthesis) |
| Cofactors, Vitamins, Prosthetic Groups, Pigments | Coenzyme A | [Coenzyme A Biosynthesis](https://rast.nmpdr.org/seedviewer.cgi?page=Subsystems&subsystem=Coenzyme_A_Biosynthesis&organism=549.408) | [Phosphopantetheine adenylyltransferase (EC 2.7.7.3)](https://rast.nmpdr.org/seedviewer.cgi?page=FunctionalRole&role=Phosphopantetheine%20adenylyltransferase%20(EC%202.7.7.3)&subsystem_name=Coenzyme_A_Biosynthesis) |
| Cofactors, Vitamins, Prosthetic Groups, Pigments | Coenzyme A | [Coenzyme A Biosynthesis](https://rast.nmpdr.org/seedviewer.cgi?page=Subsystems&subsystem=Coenzyme_A_Biosynthesis&organism=549.408) | [Ketol-acid reductoisomerase (EC 1.1.1.86)](https://rast.nmpdr.org/seedviewer.cgi?page=FunctionalRole&role=Ketol-acid%20reductoisomerase%20(EC%201.1.1.86)&subsystem_name=Coenzyme_A_Biosynthesis) |
| Cofactors, Vitamins, Prosthetic Groups, Pigments | Coenzyme A | [Coenzyme A Biosynthesis](https://rast.nmpdr.org/seedviewer.cgi?page=Subsystems&subsystem=Coenzyme_A_Biosynthesis&organism=549.408) | [Phosphopantothenoylcysteine synthetase (EC 6.3.2.5)](https://rast.nmpdr.org/seedviewer.cgi?page=FunctionalRole&role=Phosphopantothenoylcysteine%20synthetase%20(EC%206.3.2.5)&subsystem_name=Coenzyme_A_Biosynthesis) |
| Cofactors, Vitamins, Prosthetic Groups, Pigments | Coenzyme A | [Coenzyme A Biosynthesis](https://rast.nmpdr.org/seedviewer.cgi?page=Subsystems&subsystem=Coenzyme_A_Biosynthesis&organism=549.408) | [Phosphopantothenoylcysteine decarboxylase (EC 4.1.1.36)](https://rast.nmpdr.org/seedviewer.cgi?page=FunctionalRole&role=Phosphopantothenoylcysteine%20decarboxylase%20(EC%204.1.1.36)&subsystem_name=Coenzyme_A_Biosynthesis) |
| Cofactors, Vitamins, Prosthetic Groups, Pigments | Coenzyme A | [Coenzyme A Biosynthesis](https://rast.nmpdr.org/seedviewer.cgi?page=Subsystems&subsystem=Coenzyme_A_Biosynthesis&organism=549.408) | [Pantoate--beta-alanine ligase (EC 6.3.2.1)](https://rast.nmpdr.org/seedviewer.cgi?page=FunctionalRole&role=Pantoate--beta-alanine%20ligase%20(EC%206.3.2.1)&subsystem_name=Coenzyme_A_Biosynthesis) |
| Cell Wall and Capsule | Capsular and extracellular polysacchrides | [dTDP-rhamnose synthesis](https://rast.nmpdr.org/seedviewer.cgi?page=Subsystems&subsystem=dTDP-rhamnose_synthesis&organism=549.408) | [dTDP-glucose 4,6-dehydratase (EC 4.2.1.46)](https://rast.nmpdr.org/seedviewer.cgi?page=FunctionalRole&role=dTDP-glucose%204,6-dehydratase%20(EC%204.2.1.46)&subsystem_name=dTDP-rhamnose_synthesis) |
| Cell Wall and Capsule | Capsular and extracellular polysacchrides | [dTDP-rhamnose synthesis](https://rast.nmpdr.org/seedviewer.cgi?page=Subsystems&subsystem=dTDP-rhamnose_synthesis&organism=549.408) | [Glucose-1-phosphate thymidylyltransferase (EC 2.7.7.24)](https://rast.nmpdr.org/seedviewer.cgi?page=FunctionalRole&role=Glucose-1-phosphate%20thymidylyltransferase%20(EC%202.7.7.24)&subsystem_name=dTDP-rhamnose_synthesis) |
| Cell Wall and Capsule | Capsular and extracellular polysacchrides | [Capsular heptose biosynthesis](https://rast.nmpdr.org/seedviewer.cgi?page=Subsystems&subsystem=Capsular_heptose_biosynthesis&organism=549.408) | [Phosphoheptose isomerase (EC 5.3.1.-)](https://rast.nmpdr.org/seedviewer.cgi?page=FunctionalRole&role=Phosphoheptose%20isomerase%20(EC%205.3.1.-)&subsystem_name=Capsular_heptose_biosynthesis) |
| Cell Wall and Capsule | Capsular and extracellular polysacchrides | [Capsular heptose biosynthesis](https://rast.nmpdr.org/seedviewer.cgi?page=Subsystems&subsystem=Capsular_heptose_biosynthesis&organism=549.408) | [Phosphoheptose isomerase 1 (EC 5.3.1.-)](https://rast.nmpdr.org/seedviewer.cgi?page=FunctionalRole&role=Phosphoheptose%20isomerase%201%20(EC%205.3.1.-)&subsystem_name=Capsular_heptose_biosynthesis) |
| Cell Wall and Capsule | Capsular and extracellular polysacchrides | [Capsular heptose biosynthesis](https://rast.nmpdr.org/seedviewer.cgi?page=Subsystems&subsystem=Capsular_heptose_biosynthesis&organism=549.408) | [GDP-mannose 4,6-dehydratase (EC 4.2.1.47)](https://rast.nmpdr.org/seedviewer.cgi?page=FunctionalRole&role=GDP-mannose%204,6-dehydratase%20(EC%204.2.1.47)&subsystem_name=Capsular_heptose_biosynthesis) |
| Cell Wall and Capsule | Capsular and extracellular polysacchrides | [Capsular heptose biosynthesis](https://rast.nmpdr.org/seedviewer.cgi?page=Subsystems&subsystem=Capsular_heptose_biosynthesis&organism=549.408) | [D-glycero-D-manno-heptose 1,7-bisphosphate phosphatase (EC 3.1.1.-)](https://rast.nmpdr.org/seedviewer.cgi?page=FunctionalRole&role=D-glycero-D-manno-heptose%201,7-bisphosphate%20phosphatase%20(EC%203.1.1.-)&subsystem_name=Capsular_heptose_biosynthesis) |
| Cell Wall and Capsule | Capsular and extracellular polysacchrides | [Capsular Polysaccharides Biosynthesis and Assembly](https://rast.nmpdr.org/seedviewer.cgi?page=Subsystems&subsystem=Capsular_Polysaccharides_Biosynthesis_and_Assembly&organism=549.408) | [Polysaccharide export lipoprotein Wza](https://rast.nmpdr.org/seedviewer.cgi?page=FunctionalRole&role=Polysaccharide%20export%20lipoprotein%20Wza&subsystem_name=Capsular_Polysaccharides_Biosynthesis_and_Assembly) |
| Cell Wall and Capsule | Capsular and extracellular polysacchrides | [Capsular Polysaccharides Biosynthesis and Assembly](https://rast.nmpdr.org/seedviewer.cgi?page=Subsystems&subsystem=Capsular_Polysaccharides_Biosynthesis_and_Assembly&organism=549.408) | [Tyrosine-protein kinase Wzc (EC 2.7.10.2)](https://rast.nmpdr.org/seedviewer.cgi?page=FunctionalRole&role=Tyrosine-protein%20kinase%20Wzc%20(EC%202.7.10.2)&subsystem_name=Capsular_Polysaccharides_Biosynthesis_and_Assembly) |
| Cell Wall and Capsule | Capsular and extracellular polysacchrides | [Capsular Polysaccharides Biosynthesis and Assembly](https://rast.nmpdr.org/seedviewer.cgi?page=Subsystems&subsystem=Capsular_Polysaccharides_Biosynthesis_and_Assembly&organism=549.408) | [Putative capsular polysaccharide transport protein YegH](https://rast.nmpdr.org/seedviewer.cgi?page=FunctionalRole&role=Putative%20capsular%20polysaccharide%20transport%20protein%20YegH&subsystem_name=Capsular_Polysaccharides_Biosynthesis_and_Assembly) |
| Cell Wall and Capsule | Capsular and extracellular polysacchrides | [Capsular Polysaccharides Biosynthesis and Assembly](https://rast.nmpdr.org/seedviewer.cgi?page=Subsystems&subsystem=Capsular_Polysaccharides_Biosynthesis_and_Assembly&organism=549.408) | [Putative uncharacterized protein YmcB](https://rast.nmpdr.org/seedviewer.cgi?page=FunctionalRole&role=Putative%20uncharacterized%20protein%20YmcB&subsystem_name=Capsular_Polysaccharides_Biosynthesis_and_Assembly) |
| Cell Wall and Capsule | Capsular and extracellular polysacchrides | [Capsular Polysaccharides Biosynthesis and Assembly](https://rast.nmpdr.org/seedviewer.cgi?page=Subsystems&subsystem=Capsular_Polysaccharides_Biosynthesis_and_Assembly&organism=549.408) | [Low molecular weight protein-tyrosine-phosphatase Wzb (EC 3.1.3.48)](https://rast.nmpdr.org/seedviewer.cgi?page=FunctionalRole&role=Low%20molecular%20weight%20protein-tyrosine-phosphatase%20Wzb%20(EC%203.1.3.48)&subsystem_name=Capsular_Polysaccharides_Biosynthesis_and_Assembly) |
| Cell Wall and Capsule | Capsular and extracellular polysacchrides | [Rhamnose containing glycans](https://rast.nmpdr.org/seedviewer.cgi?page=Subsystems&subsystem=Rhamnose_containing_glycans&organism=549.408) | [UDP-glucose 4-epimerase (EC 5.1.3.2)](https://rast.nmpdr.org/seedviewer.cgi?page=FunctionalRole&role=UDP-glucose%204-epimerase%20(EC%205.1.3.2)&subsystem_name=Rhamnose_containing_glycans) |
| Cell Wall and Capsule | Capsular and extracellular polysacchrides | [Rhamnose containing glycans](https://rast.nmpdr.org/seedviewer.cgi?page=Subsystems&subsystem=Rhamnose_containing_glycans&organism=549.408) | [Teichoic acid export ATP-binding protein TagH (EC 3.6.3.40)](https://rast.nmpdr.org/seedviewer.cgi?page=FunctionalRole&role=Teichoic%20acid%20export%20ATP-binding%20protein%20TagH%20(EC%203.6.3.40)&subsystem_name=Rhamnose_containing_glycans) |
| Cell Wall and Capsule | Capsular and extracellular polysacchrides | [Rhamnose containing glycans](https://rast.nmpdr.org/seedviewer.cgi?page=Subsystems&subsystem=Rhamnose_containing_glycans&organism=549.408) | [dTDP-glucose 4,6-dehydratase (EC 4.2.1.46)](https://rast.nmpdr.org/seedviewer.cgi?page=FunctionalRole&role=dTDP-glucose%204,6-dehydratase%20(EC%204.2.1.46)&subsystem_name=Rhamnose_containing_glycans) |
| Cell Wall and Capsule | Capsular and extracellular polysacchrides | [Rhamnose containing glycans](https://rast.nmpdr.org/seedviewer.cgi?page=Subsystems&subsystem=Rhamnose_containing_glycans&organism=549.408) | [Glucose-1-phosphate thymidylyltransferase (EC 2.7.7.24)](https://rast.nmpdr.org/seedviewer.cgi?page=FunctionalRole&role=Glucose-1-phosphate%20thymidylyltransferase%20(EC%202.7.7.24)&subsystem_name=Rhamnose_containing_glycans) |
| Cell Wall and Capsule | Capsular and extracellular polysacchrides | [Sialic Acid Metabolism](https://rast.nmpdr.org/seedviewer.cgi?page=Subsystems&subsystem=Sialic_Acid_Metabolism&organism=549.408) | [PTS system, N-acetylglucosamine-specific IIA component (EC 2.7.1.69)](https://rast.nmpdr.org/seedviewer.cgi?page=FunctionalRole&role=PTS%20system,%20N-acetylglucosamine-specific%20IIA%20component%20(EC%202.7.1.69)&subsystem_name=Sialic_Acid_Metabolism) |
| Cell Wall and Capsule | Capsular and extracellular polysacchrides | [Sialic Acid Metabolism](https://rast.nmpdr.org/seedviewer.cgi?page=Subsystems&subsystem=Sialic_Acid_Metabolism&organism=549.408) | [PTS system, mannose-specific IIB component (EC 2.7.1.69)](https://rast.nmpdr.org/seedviewer.cgi?page=FunctionalRole&role=PTS%20system,%20mannose-specific%20IIB%20component%20(EC%202.7.1.69)&subsystem_name=Sialic_Acid_Metabolism) |
| Cell Wall and Capsule | Capsular and extracellular polysacchrides | [Sialic Acid Metabolism](https://rast.nmpdr.org/seedviewer.cgi?page=Subsystems&subsystem=Sialic_Acid_Metabolism&organism=549.408) | [Glucosamine-1-phosphate N-acetyltransferase (EC 2.3.1.157)](https://rast.nmpdr.org/seedviewer.cgi?page=FunctionalRole&role=Glucosamine-1-phosphate%20N-acetyltransferase%20(EC%202.3.1.157)&subsystem_name=Sialic_Acid_Metabolism) |
| Cell Wall and Capsule | Capsular and extracellular polysacchrides | [Sialic Acid Metabolism](https://rast.nmpdr.org/seedviewer.cgi?page=Subsystems&subsystem=Sialic_Acid_Metabolism&organism=549.408) | [Glucosamine-6-phosphate deaminase (EC 3.5.99.6)](https://rast.nmpdr.org/seedviewer.cgi?page=FunctionalRole&role=Glucosamine-6-phosphate%20deaminase%20(EC%203.5.99.6)&subsystem_name=Sialic_Acid_Metabolism) |
| Cell Wall and Capsule | Capsular and extracellular polysacchrides | [Sialic Acid Metabolism](https://rast.nmpdr.org/seedviewer.cgi?page=Subsystems&subsystem=Sialic_Acid_Metabolism&organism=549.408) | [Glucosamine--fructose-6-phosphate aminotransferase [isomerizing] (EC 2.6.1.16)](https://rast.nmpdr.org/seedviewer.cgi?page=FunctionalRole&role=Glucosamine--fructose-6-phosphate%20aminotransferase%20%5bisomerizing%5d%20(EC%202.6.1.16)&subsystem_name=Sialic_Acid_Metabolism) |
| Cell Wall and Capsule | Capsular and extracellular polysacchrides | [Sialic Acid Metabolism](https://rast.nmpdr.org/seedviewer.cgi?page=Subsystems&subsystem=Sialic_Acid_Metabolism&organism=549.408) | [PTS system, N-acetylmuramic acid-specific IIC component (EC 2.7.1.69)](https://rast.nmpdr.org/seedviewer.cgi?page=FunctionalRole&role=PTS%20system,%20N-acetylmuramic%20acid-specific%20IIC%20component%20(EC%202.7.1.69)&subsystem_name=Sialic_Acid_Metabolism) |
| Cell Wall and Capsule | Capsular and extracellular polysacchrides | [Sialic Acid Metabolism](https://rast.nmpdr.org/seedviewer.cgi?page=Subsystems&subsystem=Sialic_Acid_Metabolism&organism=549.408) | [UDP-N-acetylglucosamine 2-epimerase (EC 5.1.3.14)](https://rast.nmpdr.org/seedviewer.cgi?page=FunctionalRole&role=UDP-N-acetylglucosamine%202-epimerase%20(EC%205.1.3.14)&subsystem_name=Sialic_Acid_Metabolism) |
| Cell Wall and Capsule | Capsular and extracellular polysacchrides | [Sialic Acid Metabolism](https://rast.nmpdr.org/seedviewer.cgi?page=Subsystems&subsystem=Sialic_Acid_Metabolism&organism=549.408) | [PTS system, N-acetylglucosamine-specific IIB component (EC 2.7.1.69)](https://rast.nmpdr.org/seedviewer.cgi?page=FunctionalRole&role=PTS%20system,%20N-acetylglucosamine-specific%20IIB%20component%20(EC%202.7.1.69)&subsystem_name=Sialic_Acid_Metabolism) |
| Cell Wall and Capsule | Capsular and extracellular polysacchrides | [Sialic Acid Metabolism](https://rast.nmpdr.org/seedviewer.cgi?page=Subsystems&subsystem=Sialic_Acid_Metabolism&organism=549.408) | [PTS system, N-acetylmuramic acid-specific IIB component (EC 2.7.1.69)](https://rast.nmpdr.org/seedviewer.cgi?page=FunctionalRole&role=PTS%20system,%20N-acetylmuramic%20acid-specific%20IIB%20component%20(EC%202.7.1.69)&subsystem_name=Sialic_Acid_Metabolism) |
| Cell Wall and Capsule | Capsular and extracellular polysacchrides | [Sialic Acid Metabolism](https://rast.nmpdr.org/seedviewer.cgi?page=Subsystems&subsystem=Sialic_Acid_Metabolism&organism=549.408) | [N-acetylmannosamine kinase (EC 2.7.1.60)](https://rast.nmpdr.org/seedviewer.cgi?page=FunctionalRole&role=N-acetylmannosamine%20kinase%20(EC%202.7.1.60)&subsystem_name=Sialic_Acid_Metabolism) |
| Cell Wall and Capsule | Capsular and extracellular polysacchrides | [Sialic Acid Metabolism](https://rast.nmpdr.org/seedviewer.cgi?page=Subsystems&subsystem=Sialic_Acid_Metabolism&organism=549.408) | [Phosphoglucosamine mutase (EC 5.4.2.10)](https://rast.nmpdr.org/seedviewer.cgi?page=FunctionalRole&role=Phosphoglucosamine%20mutase%20(EC%205.4.2.10)&subsystem_name=Sialic_Acid_Metabolism) |
| Cell Wall and Capsule | Capsular and extracellular polysacchrides | [Sialic Acid Metabolism](https://rast.nmpdr.org/seedviewer.cgi?page=Subsystems&subsystem=Sialic_Acid_Metabolism&organism=549.408) | [PTS system, N-acetylglucosamine-specific IIC component (EC 2.7.1.69)](https://rast.nmpdr.org/seedviewer.cgi?page=FunctionalRole&role=PTS%20system,%20N-acetylglucosamine-specific%20IIC%20component%20(EC%202.7.1.69)&subsystem_name=Sialic_Acid_Metabolism) |
| Cell Wall and Capsule | Capsular and extracellular polysacchrides | [Sialic Acid Metabolism](https://rast.nmpdr.org/seedviewer.cgi?page=Subsystems&subsystem=Sialic_Acid_Metabolism&organism=549.408) | [N-acetylglucosamine-1-phosphate uridyltransferase (EC 2.7.7.23)](https://rast.nmpdr.org/seedviewer.cgi?page=FunctionalRole&role=N-acetylglucosamine-1-phosphate%20uridyltransferase%20(EC%202.7.7.23)&subsystem_name=Sialic_Acid_Metabolism) |
| Cell Wall and Capsule | Capsular and extracellular polysacchrides | [Sialic Acid Metabolism](https://rast.nmpdr.org/seedviewer.cgi?page=Subsystems&subsystem=Sialic_Acid_Metabolism&organism=549.408) | [TRAP-type transport system, small permease component, predicted N-acetylneuraminate transporter](https://rast.nmpdr.org/seedviewer.cgi?page=FunctionalRole&role=TRAP-type%20transport%20system,%20small%20permease%20component,%20predicted%20N-acetylneuraminate%20transporter&subsystem_name=Sialic_Acid_Metabolism) |
| Cell Wall and Capsule | Capsular and extracellular polysacchrides | [Sialic Acid Metabolism](https://rast.nmpdr.org/seedviewer.cgi?page=Subsystems&subsystem=Sialic_Acid_Metabolism&organism=549.408) | [N-acetylglucosamine-6-phosphate deacetylase (EC 3.5.1.25)](https://rast.nmpdr.org/seedviewer.cgi?page=FunctionalRole&role=N-acetylglucosamine-6-phosphate%20deacetylase%20(EC%203.5.1.25)&subsystem_name=Sialic_Acid_Metabolism) |
| Cell Wall and Capsule | Capsular and extracellular polysacchrides | [Sialic Acid Metabolism](https://rast.nmpdr.org/seedviewer.cgi?page=Subsystems&subsystem=Sialic_Acid_Metabolism&organism=549.408) | [Sialic acid utilization regulator, RpiR family](https://rast.nmpdr.org/seedviewer.cgi?page=FunctionalRole&role=Sialic%20acid%20utilization%20regulator,%20RpiR%20family&subsystem_name=Sialic_Acid_Metabolism) |
| Cell Wall and Capsule | Gram-Negative cell wall components | [Lipopolysaccharide assembly](https://rast.nmpdr.org/seedviewer.cgi?page=Subsystems&subsystem=Lipopolysaccharide_assembly&organism=549.408) | [Uncharacterized ABC transporter, permease component YrbE](https://rast.nmpdr.org/seedviewer.cgi?page=FunctionalRole&role=Uncharacterized%20ABC%20transporter,%20permease%20component%20YrbE&subsystem_name=Lipopolysaccharide_assembly) |
| Cell Wall and Capsule | Gram-Negative cell wall components | [Lipopolysaccharide assembly](https://rast.nmpdr.org/seedviewer.cgi?page=Subsystems&subsystem=Lipopolysaccharide_assembly&organism=549.408) | [Lipoprotein releasing system transmembrane protein LolC](https://rast.nmpdr.org/seedviewer.cgi?page=FunctionalRole&role=Lipoprotein%20releasing%20system%20transmembrane%20protein%20LolC&subsystem_name=Lipopolysaccharide_assembly) |
| Cell Wall and Capsule | Gram-Negative cell wall components | [Lipopolysaccharide assembly](https://rast.nmpdr.org/seedviewer.cgi?page=Subsystems&subsystem=Lipopolysaccharide_assembly&organism=549.408) | [Outer membrane protein YfgL, lipoprotein component of the protein assembly complex (forms a complex with YaeT, YfiO, and NlpB)](https://rast.nmpdr.org/seedviewer.cgi?page=FunctionalRole&role=Outer%20membrane%20protein%20YfgL,%20lipoprotein%20component%20of%20the%20protein%20assembly%20complex%20(forms%20a%20complex%20with%20YaeT,%20YfiO,%20and%20NlpB)&subsystem_name=Lipopolysaccharide_assembly) |
| Cell Wall and Capsule | Gram-Negative cell wall components | [Lipopolysaccharide assembly](https://rast.nmpdr.org/seedviewer.cgi?page=Subsystems&subsystem=Lipopolysaccharide_assembly&organism=549.408) | [Outer membrane lipoprotein carrier protein LolA](https://rast.nmpdr.org/seedviewer.cgi?page=FunctionalRole&role=Outer%20membrane%20lipoprotein%20carrier%20protein%20LolA&subsystem_name=Lipopolysaccharide_assembly) |
| Cell Wall and Capsule | Gram-Negative cell wall components | [Lipopolysaccharide assembly](https://rast.nmpdr.org/seedviewer.cgi?page=Subsystems&subsystem=Lipopolysaccharide_assembly&organism=549.408) | [Outer membrane lipoprotein SmpA, a component of the essential YaeT outer-membrane protein assembly complex](https://rast.nmpdr.org/seedviewer.cgi?page=FunctionalRole&role=Outer%20membrane%20lipoprotein%20SmpA,%20a%20component%20of%20the%20essential%20YaeT%20outer-membrane%20protein%20assembly%20complex&subsystem_name=Lipopolysaccharide_assembly) |
| Cell Wall and Capsule | Gram-Negative cell wall components | [Lipopolysaccharide assembly](https://rast.nmpdr.org/seedviewer.cgi?page=Subsystems&subsystem=Lipopolysaccharide_assembly&organism=549.408) | [Uncharacterized protein YrbK clustered with lipopolysaccharide transporters](https://rast.nmpdr.org/seedviewer.cgi?page=FunctionalRole&role=Uncharacterized%20protein%20YrbK%20clustered%20with%20lipopolysaccharide%20transporters&subsystem_name=Lipopolysaccharide_assembly) |
| Cell Wall and Capsule | Gram-Negative cell wall components | [Lipopolysaccharide assembly](https://rast.nmpdr.org/seedviewer.cgi?page=Subsystems&subsystem=Lipopolysaccharide_assembly&organism=549.408) | [Outer membrane protein assembly factor YaeT precursor](https://rast.nmpdr.org/seedviewer.cgi?page=FunctionalRole&role=Outer%20membrane%20protein%20assembly%20factor%20YaeT%20precursor&subsystem_name=Lipopolysaccharide_assembly) |
| Cell Wall and Capsule | Gram-Negative cell wall components | [Lipopolysaccharide assembly](https://rast.nmpdr.org/seedviewer.cgi?page=Subsystems&subsystem=Lipopolysaccharide_assembly&organism=549.408) | [Lipopolysaccharide ABC transporter, ATP-binding protein LptB](https://rast.nmpdr.org/seedviewer.cgi?page=FunctionalRole&role=Lipopolysaccharide%20ABC%20transporter,%20ATP-binding%20protein%20LptB&subsystem_name=Lipopolysaccharide_assembly) |
| Cell Wall and Capsule | Gram-Negative cell wall components | [Lipopolysaccharide assembly](https://rast.nmpdr.org/seedviewer.cgi?page=Subsystems&subsystem=Lipopolysaccharide_assembly&organism=549.408) | [Probable component of the lipoprotein assembly complex (forms a complex with YaeT, YfgL, and NlpB)](https://rast.nmpdr.org/seedviewer.cgi?page=FunctionalRole&role=Probable%20component%20of%20the%20lipoprotein%20assembly%20complex%20(forms%20a%20complex%20with%20YaeT,%20YfgL,%20and%20NlpB)&subsystem_name=Lipopolysaccharide_assembly) |
| Cell Wall and Capsule | Gram-Negative cell wall components | [Lipopolysaccharide assembly](https://rast.nmpdr.org/seedviewer.cgi?page=Subsystems&subsystem=Lipopolysaccharide_assembly&organism=549.408) | [Uncharacterized ABC transporter, periplasmic component YrbD](https://rast.nmpdr.org/seedviewer.cgi?page=FunctionalRole&role=Uncharacterized%20ABC%20transporter,%20periplasmic%20component%20YrbD&subsystem_name=Lipopolysaccharide_assembly) |
| Cell Wall and Capsule | Gram-Negative cell wall components | [Lipopolysaccharide assembly](https://rast.nmpdr.org/seedviewer.cgi?page=Subsystems&subsystem=Lipopolysaccharide_assembly&organism=549.408) | [LptA, protein essential for LPS transport across the periplasm](https://rast.nmpdr.org/seedviewer.cgi?page=FunctionalRole&role=LptA,%20protein%20essential%20for%20LPS%20transport%20across%20the%20periplasm&subsystem_name=Lipopolysaccharide_assembly) |
| Cell Wall and Capsule | Gram-Negative cell wall components | [Lipopolysaccharide assembly](https://rast.nmpdr.org/seedviewer.cgi?page=Subsystems&subsystem=Lipopolysaccharide_assembly&organism=549.408) | [HtrA protease/chaperone protein](https://rast.nmpdr.org/seedviewer.cgi?page=FunctionalRole&role=HtrA%20protease/chaperone%20protein&subsystem_name=Lipopolysaccharide_assembly) |
| Cell Wall and Capsule | Gram-Negative cell wall components | [Lipopolysaccharide assembly](https://rast.nmpdr.org/seedviewer.cgi?page=Subsystems&subsystem=Lipopolysaccharide_assembly&organism=549.408) | [Inner membrane protein YrbG, predicted calcium/sodium:proton antiporter](https://rast.nmpdr.org/seedviewer.cgi?page=FunctionalRole&role=Inner%20membrane%20protein%20YrbG,%20predicted%20calcium/sodium:proton%20antiporter&subsystem_name=Lipopolysaccharide_assembly) |
| Cell Wall and Capsule | Gram-Negative cell wall components | [Lipopolysaccharide assembly](https://rast.nmpdr.org/seedviewer.cgi?page=Subsystems&subsystem=Lipopolysaccharide_assembly&organism=549.408) | [Survival protein SurA precursor (Peptidyl-prolyl cis-trans isomerase SurA) (EC 5.2.1.8)](https://rast.nmpdr.org/seedviewer.cgi?page=FunctionalRole&role=Survival%20protein%20SurA%20precursor%20(Peptidyl-prolyl%20cis-trans%20isomerase%20SurA)%20(EC%205.2.1.8)&subsystem_name=Lipopolysaccharide_assembly) |
| Cell Wall and Capsule | Gram-Negative cell wall components | [Lipopolysaccharide assembly](https://rast.nmpdr.org/seedviewer.cgi?page=Subsystems&subsystem=Lipopolysaccharide_assembly&organism=549.408) | [Lipoprotein releasing system ATP-binding protein LolD](https://rast.nmpdr.org/seedviewer.cgi?page=FunctionalRole&role=Lipoprotein%20releasing%20system%20ATP-binding%20protein%20LolD&subsystem_name=Lipopolysaccharide_assembly) |
| Cell Wall and Capsule | Gram-Negative cell wall components | [Lipopolysaccharide assembly](https://rast.nmpdr.org/seedviewer.cgi?page=Subsystems&subsystem=Lipopolysaccharide_assembly&organism=549.408) | [Outer membrane protein NlpB, lipoprotein component of the protein assembly complex (forms a complex with YaeT, YfiO, and YfgL)](https://rast.nmpdr.org/seedviewer.cgi?page=FunctionalRole&role=Outer%20membrane%20protein%20NlpB,%20lipoprotein%20component%20of%20the%20protein%20assembly%20complex%20(forms%20a%20complex%20with%20YaeT,%20YfiO,%20and%20YfgL)&subsystem_name=Lipopolysaccharide_assembly) |
| Cell Wall and Capsule | Gram-Negative cell wall components | [Lipopolysaccharide assembly](https://rast.nmpdr.org/seedviewer.cgi?page=Subsystems&subsystem=Lipopolysaccharide_assembly&organism=549.408) | [LPS-assembly lipoprotein RlpB precursor (Rare lipoprotein B)](https://rast.nmpdr.org/seedviewer.cgi?page=FunctionalRole&role=LPS-assembly%20lipoprotein%20RlpB%20precursor%20(Rare%20lipoprotein%20B)&subsystem_name=Lipopolysaccharide_assembly) |
| Cell Wall and Capsule | Gram-Negative cell wall components | [Lipopolysaccharide assembly](https://rast.nmpdr.org/seedviewer.cgi?page=Subsystems&subsystem=Lipopolysaccharide_assembly&organism=549.408) | [Lipoprotein releasing system transmembrane protein LolE](https://rast.nmpdr.org/seedviewer.cgi?page=FunctionalRole&role=Lipoprotein%20releasing%20system%20transmembrane%20protein%20LolE&subsystem_name=Lipopolysaccharide_assembly) |
| Cell Wall and Capsule | Gram-Negative cell wall components | [Lipopolysaccharide assembly](https://rast.nmpdr.org/seedviewer.cgi?page=Subsystems&subsystem=Lipopolysaccharide_assembly&organism=549.408) | [Outer membrane protein Imp, required for envelope biogenesis](https://rast.nmpdr.org/seedviewer.cgi?page=FunctionalRole&role=Outer%20membrane%20protein%20Imp,%20required%20for%20envelope%20biogenesis&subsystem_name=Lipopolysaccharide_assembly) |
| Cell Wall and Capsule | Gram-Negative cell wall components | [Lipopolysaccharide assembly](https://rast.nmpdr.org/seedviewer.cgi?page=Subsystems&subsystem=Lipopolysaccharide_assembly&organism=549.408) | [Outer membrane protein H precursor](https://rast.nmpdr.org/seedviewer.cgi?page=FunctionalRole&role=Outer%20membrane%20protein%20H%20precursor&subsystem_name=Lipopolysaccharide_assembly) |
| Cell Wall and Capsule | Gram-Negative cell wall components | [Lipopolysaccharide assembly](https://rast.nmpdr.org/seedviewer.cgi?page=Subsystems&subsystem=Lipopolysaccharide_assembly&organism=549.408) | [Uncharacterized ABC transporter, auxiliary component YrbC](https://rast.nmpdr.org/seedviewer.cgi?page=FunctionalRole&role=Uncharacterized%20ABC%20transporter,%20auxiliary%20component%20YrbC&subsystem_name=Lipopolysaccharide_assembly) |
| Cell Wall and Capsule | Gram-Negative cell wall components | [Lipopolysaccharide assembly](https://rast.nmpdr.org/seedviewer.cgi?page=Subsystems&subsystem=Lipopolysaccharide_assembly&organism=549.408) | [Uncharacterized ABC transporter, ATP-binding protein YrbF](https://rast.nmpdr.org/seedviewer.cgi?page=FunctionalRole&role=Uncharacterized%20ABC%20transporter,%20ATP-binding%20protein%20YrbF&subsystem_name=Lipopolysaccharide_assembly) |
| Cell Wall and Capsule | Gram-Negative cell wall components | [Lipid A modifications](https://rast.nmpdr.org/seedviewer.cgi?page=Subsystems&subsystem=Lipid_A_modifications&organism=549.408) | [Sensor protein PhoQ (EC 2.7.13.3)](https://rast.nmpdr.org/seedviewer.cgi?page=FunctionalRole&role=Sensor%20protein%20PhoQ%20(EC%202.7.13.3)&subsystem_name=Lipid_A_modifications) |
| Cell Wall and Capsule | Gram-Negative cell wall components | [Lipid A modifications](https://rast.nmpdr.org/seedviewer.cgi?page=Subsystems&subsystem=Lipid_A_modifications&organism=549.408) | [Phosphoethanolamine transferase EptA specific for the 1 phosphate group of core-lipid A](https://rast.nmpdr.org/seedviewer.cgi?page=FunctionalRole&role=Phosphoethanolamine%20transferase%20EptA%20specific%20for%20the%201%20phosphate%20group%20of%20core-lipid%20A&subsystem_name=Lipid_A_modifications) |
| Cell Wall and Capsule | Gram-Negative cell wall components | [Lipid A modifications](https://rast.nmpdr.org/seedviewer.cgi?page=Subsystems&subsystem=Lipid_A_modifications&organism=549.408) | [Lipid A acylation protein PagP, palmitoyltransferase](https://rast.nmpdr.org/seedviewer.cgi?page=FunctionalRole&role=Lipid%20A%20acylation%20protein%20PagP,%20palmitoyltransferase&subsystem_name=Lipid_A_modifications) |
| Cell Wall and Capsule | Gram-Negative cell wall components | [Lipid A modifications](https://rast.nmpdr.org/seedviewer.cgi?page=Subsystems&subsystem=Lipid_A_modifications&organism=549.408) | [Phosphoethanolamine transferase specific for the outer Kdo residue of lipopolysaccharide](https://rast.nmpdr.org/seedviewer.cgi?page=FunctionalRole&role=Phosphoethanolamine%20transferase%20specific%20for%20the%20outer%20Kdo%20residue%20of%20lipopolysaccharide&subsystem_name=Lipid_A_modifications) |
| Cell Wall and Capsule | Gram-Negative cell wall components | [Lipid A modifications](https://rast.nmpdr.org/seedviewer.cgi?page=Subsystems&subsystem=Lipid_A_modifications&organism=549.408) | [Transcriptional regulatory protein basR/pmrA](https://rast.nmpdr.org/seedviewer.cgi?page=FunctionalRole&role=Transcriptional%20regulatory%20protein%20basR/pmrA&subsystem_name=Lipid_A_modifications) |
| Cell Wall and Capsule | Gram-Negative cell wall components | [Lipid A modifications](https://rast.nmpdr.org/seedviewer.cgi?page=Subsystems&subsystem=Lipid_A_modifications&organism=549.408) | [Transcriptional regulatory protein PhoP](https://rast.nmpdr.org/seedviewer.cgi?page=FunctionalRole&role=Transcriptional%20regulatory%20protein%20PhoP&subsystem_name=Lipid_A_modifications) |
| Cell Wall and Capsule | Gram-Negative cell wall components | [Lipid A modifications](https://rast.nmpdr.org/seedviewer.cgi?page=Subsystems&subsystem=Lipid_A_modifications&organism=549.408) | [Sensor protein basS/pmrB (EC 2.7.3.-)](https://rast.nmpdr.org/seedviewer.cgi?page=FunctionalRole&role=Sensor%20protein%20basS/pmrB%20(EC%202.7.3.-)&subsystem_name=Lipid_A_modifications) |
| Cell Wall and Capsule | Gram-Negative cell wall components | [Lipid A-Ara4N pathway ( Polymyxin resistance )](https://rast.nmpdr.org/seedviewer.cgi?page=Subsystems&subsystem=Lipid_A-Ara4N_pathway_(_Polymyxin_resistance_)&organism=549.408) | [Polymyxin resistance protein PmrL, sucrose-6 phosphate hydrolase](https://rast.nmpdr.org/seedviewer.cgi?page=FunctionalRole&role=Polymyxin%20resistance%20protein%20PmrL,%20sucrose-6%20phosphate%20hydrolase&subsystem_name=Lipid_A-Ara4N_pathway_(_Polymyxin_resistance_)) |
| Cell Wall and Capsule | Gram-Negative cell wall components | [Lipid A-Ara4N pathway ( Polymyxin resistance )](https://rast.nmpdr.org/seedviewer.cgi?page=Subsystems&subsystem=Lipid_A-Ara4N_pathway_(_Polymyxin_resistance_)&organism=549.408) | [Polymyxin resistance protein PmrJ, predicted deacetylase](https://rast.nmpdr.org/seedviewer.cgi?page=FunctionalRole&role=Polymyxin%20resistance%20protein%20PmrJ,%20predicted%20deacetylase&subsystem_name=Lipid_A-Ara4N_pathway_(_Polymyxin_resistance_)) |
| Cell Wall and Capsule | Gram-Negative cell wall components | [Lipid A-Ara4N pathway ( Polymyxin resistance )](https://rast.nmpdr.org/seedviewer.cgi?page=Subsystems&subsystem=Lipid_A-Ara4N_pathway_(_Polymyxin_resistance_)&organism=549.408) | [UDP-glucuronic acid oxidase (UDP-4-keto-hexauronic acid decarboxylating) (EC 1.1.1.305)](https://rast.nmpdr.org/seedviewer.cgi?page=FunctionalRole&role=UDP-glucuronic%20acid%20oxidase%20(UDP-4-keto-hexauronic%20acid%20decarboxylating)%20(EC%201.1.1.305)&subsystem_name=Lipid_A-Ara4N_pathway_(_Polymyxin_resistance_)) |
| Cell Wall and Capsule | Gram-Negative cell wall components | [Lipid A-Ara4N pathway ( Polymyxin resistance )](https://rast.nmpdr.org/seedviewer.cgi?page=Subsystems&subsystem=Lipid_A-Ara4N_pathway_(_Polymyxin_resistance_)&organism=549.408) | [UDP-4-amino-4-deoxy-L-arabinose--oxoglutarate aminotransferase (EC 2.6.1.-)](https://rast.nmpdr.org/seedviewer.cgi?page=FunctionalRole&role=UDP-4-amino-4-deoxy-L-arabinose--oxoglutarate%20aminotransferase%20(EC%202.6.1.-)&subsystem_name=Lipid_A-Ara4N_pathway_(_Polymyxin_resistance_)) |
| Cell Wall and Capsule | Gram-Negative cell wall components | [Lipid A-Ara4N pathway ( Polymyxin resistance )](https://rast.nmpdr.org/seedviewer.cgi?page=Subsystems&subsystem=Lipid_A-Ara4N_pathway_(_Polymyxin_resistance_)&organism=549.408) | [UDP-4-amino-4-deoxy-L-arabinose formyltransferase (EC 2.1.2.13)](https://rast.nmpdr.org/seedviewer.cgi?page=FunctionalRole&role=UDP-4-amino-4-deoxy-L-arabinose%20formyltransferase%20(EC%202.1.2.13)&subsystem_name=Lipid_A-Ara4N_pathway_(_Polymyxin_resistance_)) |
| Cell Wall and Capsule | Gram-Negative cell wall components | [Lipid A-Ara4N pathway ( Polymyxin resistance )](https://rast.nmpdr.org/seedviewer.cgi?page=Subsystems&subsystem=Lipid_A-Ara4N_pathway_(_Polymyxin_resistance_)&organism=549.408) | [Polymyxin resistance protein ArnC, glycosyl transferase (EC 2.4.-.-)](https://rast.nmpdr.org/seedviewer.cgi?page=FunctionalRole&role=Polymyxin%20resistance%20protein%20ArnC,%20glycosyl%20transferase%20(EC%202.4.-.-)&subsystem_name=Lipid_A-Ara4N_pathway_(_Polymyxin_resistance_)) |
| Cell Wall and Capsule | Gram-Negative cell wall components | [Lipid A-Ara4N pathway ( Polymyxin resistance )](https://rast.nmpdr.org/seedviewer.cgi?page=Subsystems&subsystem=Lipid_A-Ara4N_pathway_(_Polymyxin_resistance_)&organism=549.408) | [Polymyxin resistance protein ArnT, undecaprenyl phosphate-alpha-L-Ara4N transferase](https://rast.nmpdr.org/seedviewer.cgi?page=FunctionalRole&role=Polymyxin%20resistance%20protein%20ArnT,%20undecaprenyl%20phosphate-alpha-L-Ara4N%20transferase&subsystem_name=Lipid_A-Ara4N_pathway_(_Polymyxin_resistance_)) |
| Cell Wall and Capsule | Gram-Negative cell wall components | [Lipid A-Ara4N pathway ( Polymyxin resistance )](https://rast.nmpdr.org/seedviewer.cgi?page=Subsystems&subsystem=Lipid_A-Ara4N_pathway_(_Polymyxin_resistance_)&organism=549.408) | [Polymyxin resistance protein PmrM](https://rast.nmpdr.org/seedviewer.cgi?page=FunctionalRole&role=Polymyxin%20resistance%20protein%20PmrM&subsystem_name=Lipid_A-Ara4N_pathway_(_Polymyxin_resistance_)) |
| Cell Wall and Capsule | Gram-Negative cell wall components | [Peptidoglycan lipid II flippase](https://rast.nmpdr.org/seedviewer.cgi?page=Subsystems&subsystem=Peptidoglycan_lipid_II_flippase&organism=549.408) | [Protein of unknown function YceH](https://rast.nmpdr.org/seedviewer.cgi?page=FunctionalRole&role=Protein%20of%20unknown%20function%20YceH&subsystem_name=Peptidoglycan_lipid_II_flippase) |
| Cell Wall and Capsule | Gram-Negative cell wall components | [Peptidoglycan lipid II flippase](https://rast.nmpdr.org/seedviewer.cgi?page=Subsystems&subsystem=Peptidoglycan_lipid_II_flippase&organism=549.408) | [Virulence factor MviM](https://rast.nmpdr.org/seedviewer.cgi?page=FunctionalRole&role=Virulence%20factor%20MviM&subsystem_name=Peptidoglycan_lipid_II_flippase) |
| Cell Wall and Capsule | Gram-Negative cell wall components | [Peptidoglycan lipid II flippase](https://rast.nmpdr.org/seedviewer.cgi?page=Subsystems&subsystem=Peptidoglycan_lipid_II_flippase&organism=549.408) | [Proposed peptidoglycan lipid II flippase MurJ](https://rast.nmpdr.org/seedviewer.cgi?page=FunctionalRole&role=Proposed%20peptidoglycan%20lipid%20II%20flippase%20MurJ&subsystem_name=Peptidoglycan_lipid_II_flippase) |
| Cell Wall and Capsule | Gram-Negative cell wall components | [Inner membrane protein YhjD and conserved cluster involved in LPS biosynthesis](https://rast.nmpdr.org/seedviewer.cgi?page=Subsystems&subsystem=Inner_membrane_protein_YhjD_and_conserved_cluster_involved_in_LPS_biosynthesis&organism=549.408) | [Protein YhjJ, putative peptidase](https://rast.nmpdr.org/seedviewer.cgi?page=FunctionalRole&role=Protein%20YhjJ,%20putative%20peptidase&subsystem_name=Inner_membrane_protein_YhjD_and_conserved_cluster_involved_in_LPS_biosynthesis) |
| Cell Wall and Capsule | Gram-Negative cell wall components | [Inner membrane protein YhjD and conserved cluster involved in LPS biosynthesis](https://rast.nmpdr.org/seedviewer.cgi?page=Subsystems&subsystem=Inner_membrane_protein_YhjD_and_conserved_cluster_involved_in_LPS_biosynthesis&organism=549.408) | [LysR family transcriptional regulator YhjC](https://rast.nmpdr.org/seedviewer.cgi?page=FunctionalRole&role=LysR%20family%20transcriptional%20regulator%20YhjC&subsystem_name=Inner_membrane_protein_YhjD_and_conserved_cluster_involved_in_LPS_biosynthesis) |
| Cell Wall and Capsule | Gram-Negative cell wall components | [Inner membrane protein YhjD and conserved cluster involved in LPS biosynthesis](https://rast.nmpdr.org/seedviewer.cgi?page=Subsystems&subsystem=Inner_membrane_protein_YhjD_and_conserved_cluster_involved_in_LPS_biosynthesis&organism=549.408) | [Inner membrane protein YhjD](https://rast.nmpdr.org/seedviewer.cgi?page=FunctionalRole&role=Inner%20membrane%20protein%20YhjD&subsystem_name=Inner_membrane_protein_YhjD_and_conserved_cluster_involved_in_LPS_biosynthesis) |
| Cell Wall and Capsule | Gram-Negative cell wall components | [Inner membrane protein YhjD and conserved cluster involved in LPS biosynthesis](https://rast.nmpdr.org/seedviewer.cgi?page=Subsystems&subsystem=Inner_membrane_protein_YhjD_and_conserved_cluster_involved_in_LPS_biosynthesis&organism=549.408) | [Uncharacterized protein YhjG](https://rast.nmpdr.org/seedviewer.cgi?page=FunctionalRole&role=Uncharacterized%20protein%20YhjG&subsystem_name=Inner_membrane_protein_YhjD_and_conserved_cluster_involved_in_LPS_biosynthesis) |
| Cell Wall and Capsule | Gram-Negative cell wall components | [Inner membrane protein YhjD and conserved cluster involved in LPS biosynthesis](https://rast.nmpdr.org/seedviewer.cgi?page=Subsystems&subsystem=Inner_membrane_protein_YhjD_and_conserved_cluster_involved_in_LPS_biosynthesis&organism=549.408) | [Inner membrane metabolite transport protein YhjE](https://rast.nmpdr.org/seedviewer.cgi?page=FunctionalRole&role=Inner%20membrane%20metabolite%20transport%20protein%20YhjE&subsystem_name=Inner_membrane_protein_YhjD_and_conserved_cluster_involved_in_LPS_biosynthesis) |
| Cell Wall and Capsule | Gram-Negative cell wall components | [LOS core oligosaccharide biosynthesis](https://rast.nmpdr.org/seedviewer.cgi?page=Subsystems&subsystem=LOS_core_oligosaccharide_biosynthesis&organism=549.408) | [Phosphoheptose isomerase (EC 5.3.1.-)](https://rast.nmpdr.org/seedviewer.cgi?page=FunctionalRole&role=Phosphoheptose%20isomerase%20(EC%205.3.1.-)&subsystem_name=LOS_core_oligosaccharide_biosynthesis) |
| Cell Wall and Capsule | Gram-Negative cell wall components | [LOS core oligosaccharide biosynthesis](https://rast.nmpdr.org/seedviewer.cgi?page=Subsystems&subsystem=LOS_core_oligosaccharide_biosynthesis&organism=549.408) | [Beta-1,3-glucosyltransferase](https://rast.nmpdr.org/seedviewer.cgi?page=FunctionalRole&role=Beta-1,3-glucosyltransferase&subsystem_name=LOS_core_oligosaccharide_biosynthesis) |
| Cell Wall and Capsule | Gram-Negative cell wall components | [LOS core oligosaccharide biosynthesis](https://rast.nmpdr.org/seedviewer.cgi?page=Subsystems&subsystem=LOS_core_oligosaccharide_biosynthesis&organism=549.408) | [Lipopolysaccharide heptosyltransferase III (EC 2.4.1.-)](https://rast.nmpdr.org/seedviewer.cgi?page=FunctionalRole&role=Lipopolysaccharide%20heptosyltransferase%20III%20(EC%202.4.1.-)&subsystem_name=LOS_core_oligosaccharide_biosynthesis) |
| Cell Wall and Capsule | Gram-Negative cell wall components | [LOS core oligosaccharide biosynthesis](https://rast.nmpdr.org/seedviewer.cgi?page=Subsystems&subsystem=LOS_core_oligosaccharide_biosynthesis&organism=549.408) | [Phosphoheptose isomerase 1 (EC 5.3.1.-)](https://rast.nmpdr.org/seedviewer.cgi?page=FunctionalRole&role=Phosphoheptose%20isomerase%201%20(EC%205.3.1.-)&subsystem_name=LOS_core_oligosaccharide_biosynthesis) |
| Cell Wall and Capsule | Gram-Negative cell wall components | [LOS core oligosaccharide biosynthesis](https://rast.nmpdr.org/seedviewer.cgi?page=Subsystems&subsystem=LOS_core_oligosaccharide_biosynthesis&organism=549.408) | [ADP-L-glycero-D-manno-heptose-6-epimerase (EC 5.1.3.20)](https://rast.nmpdr.org/seedviewer.cgi?page=FunctionalRole&role=ADP-L-glycero-D-manno-heptose-6-epimerase%20(EC%205.1.3.20)&subsystem_name=LOS_core_oligosaccharide_biosynthesis) |
| Cell Wall and Capsule | Gram-Negative cell wall components | [LOS core oligosaccharide biosynthesis](https://rast.nmpdr.org/seedviewer.cgi?page=Subsystems&subsystem=LOS_core_oligosaccharide_biosynthesis&organism=549.408) | [D-glycero-D-manno-heptose 1,7-bisphosphate phosphatase (EC 3.1.1.-)](https://rast.nmpdr.org/seedviewer.cgi?page=FunctionalRole&role=D-glycero-D-manno-heptose%201,7-bisphosphate%20phosphatase%20(EC%203.1.1.-)&subsystem_name=LOS_core_oligosaccharide_biosynthesis) |
| Cell Wall and Capsule | Gram-Negative cell wall components | [LOS core oligosaccharide biosynthesis](https://rast.nmpdr.org/seedviewer.cgi?page=Subsystems&subsystem=LOS_core_oligosaccharide_biosynthesis&organism=549.408) | [O-antigen ligase](https://rast.nmpdr.org/seedviewer.cgi?page=FunctionalRole&role=O-antigen%20ligase&subsystem_name=LOS_core_oligosaccharide_biosynthesis) |
| Cell Wall and Capsule | Gram-Negative cell wall components | [LOS core oligosaccharide biosynthesis](https://rast.nmpdr.org/seedviewer.cgi?page=Subsystems&subsystem=LOS_core_oligosaccharide_biosynthesis&organism=549.408) | [Lipopolysaccharide heptosyltransferase I (EC 2.4.1.-)](https://rast.nmpdr.org/seedviewer.cgi?page=FunctionalRole&role=Lipopolysaccharide%20heptosyltransferase%20I%20(EC%202.4.1.-)&subsystem_name=LOS_core_oligosaccharide_biosynthesis) |
| Cell Wall and Capsule | Gram-Negative cell wall components | [LOS core oligosaccharide biosynthesis](https://rast.nmpdr.org/seedviewer.cgi?page=Subsystems&subsystem=LOS_core_oligosaccharide_biosynthesis&organism=549.408) | [ADP-heptose synthase (EC 2.7.-.-)](https://rast.nmpdr.org/seedviewer.cgi?page=FunctionalRole&role=ADP-heptose%20synthase%20(EC%202.7.-.-)&subsystem_name=LOS_core_oligosaccharide_biosynthesis) |
| Cell Wall and Capsule | Gram-Negative cell wall components | [LOS core oligosaccharide biosynthesis](https://rast.nmpdr.org/seedviewer.cgi?page=Subsystems&subsystem=LOS_core_oligosaccharide_biosynthesis&organism=549.408) | [Lipopolysaccharide 1,6-galactosyltransferase (EC 2.4.1.-)](https://rast.nmpdr.org/seedviewer.cgi?page=FunctionalRole&role=Lipopolysaccharide%201,6-galactosyltransferase%20(EC%202.4.1.-)&subsystem_name=LOS_core_oligosaccharide_biosynthesis) |
| Cell Wall and Capsule | Gram-Negative cell wall components | [LOS core oligosaccharide biosynthesis](https://rast.nmpdr.org/seedviewer.cgi?page=Subsystems&subsystem=LOS_core_oligosaccharide_biosynthesis&organism=549.408) | [Beta-1,4-galactosyltransferase](https://rast.nmpdr.org/seedviewer.cgi?page=FunctionalRole&role=Beta-1,4-galactosyltransferase&subsystem_name=LOS_core_oligosaccharide_biosynthesis) |
| Cell Wall and Capsule | Gram-Negative cell wall components | [LOS core oligosaccharide biosynthesis](https://rast.nmpdr.org/seedviewer.cgi?page=Subsystems&subsystem=LOS_core_oligosaccharide_biosynthesis&organism=549.408) | [ADP-heptose--lipooligosaccharide heptosyltransferase II (EC 2.4.1.-)](https://rast.nmpdr.org/seedviewer.cgi?page=FunctionalRole&role=ADP-heptose--lipooligosaccharide%20heptosyltransferase%20II%20(EC%202.4.1.-)&subsystem_name=LOS_core_oligosaccharide_biosynthesis) |
| Cell Wall and Capsule | Gram-Negative cell wall components | [LOS core oligosaccharide biosynthesis](https://rast.nmpdr.org/seedviewer.cgi?page=Subsystems&subsystem=LOS_core_oligosaccharide_biosynthesis&organism=549.408) | [UDP-glucose:(heptosyl) LPS alpha1,3-glucosyltransferase WaaG (EC 2.4.1.-)](https://rast.nmpdr.org/seedviewer.cgi?page=FunctionalRole&role=UDP-glucose:(heptosyl)%20LPS%20alpha1,3-glucosyltransferase%20WaaG%20(EC%202.4.1.-)&subsystem_name=LOS_core_oligosaccharide_biosynthesis) |
| Cell Wall and Capsule | Gram-Negative cell wall components | [LOS core oligosaccharide biosynthesis](https://rast.nmpdr.org/seedviewer.cgi?page=Subsystems&subsystem=LOS_core_oligosaccharide_biosynthesis&organism=549.408) | [D-glycero-beta-D-manno-heptose 7-phosphate kinase](https://rast.nmpdr.org/seedviewer.cgi?page=FunctionalRole&role=D-glycero-beta-D-manno-heptose%207-phosphate%20kinase&subsystem_name=LOS_core_oligosaccharide_biosynthesis) |
| Cell Wall and Capsule | Gram-Negative cell wall components | [Lipoprotein sorting system](https://rast.nmpdr.org/seedviewer.cgi?page=Subsystems&subsystem=Lipoprotein_sorting_system&organism=549.408) | [Lipoprotein releasing system transmembrane protein LolC](https://rast.nmpdr.org/seedviewer.cgi?page=FunctionalRole&role=Lipoprotein%20releasing%20system%20transmembrane%20protein%20LolC&subsystem_name=Lipoprotein_sorting_system) |
| Cell Wall and Capsule | Gram-Negative cell wall components | [Lipoprotein sorting system](https://rast.nmpdr.org/seedviewer.cgi?page=Subsystems&subsystem=Lipoprotein_sorting_system&organism=549.408) | [Lipoprotein releasing system ATP-binding protein LolD](https://rast.nmpdr.org/seedviewer.cgi?page=FunctionalRole&role=Lipoprotein%20releasing%20system%20ATP-binding%20protein%20LolD&subsystem_name=Lipoprotein_sorting_system) |
| Cell Wall and Capsule | Gram-Negative cell wall components | [Lipoprotein sorting system](https://rast.nmpdr.org/seedviewer.cgi?page=Subsystems&subsystem=Lipoprotein_sorting_system&organism=549.408) | [Lipoprotein releasing system transmembrane protein LolE](https://rast.nmpdr.org/seedviewer.cgi?page=FunctionalRole&role=Lipoprotein%20releasing%20system%20transmembrane%20protein%20LolE&subsystem_name=Lipoprotein_sorting_system) |
| Cell Wall and Capsule | Gram-Negative cell wall components | [Lipoprotein sorting system](https://rast.nmpdr.org/seedviewer.cgi?page=Subsystems&subsystem=Lipoprotein_sorting_system&organism=549.408) | [Outer membrane lipoprotein carrier protein LolA](https://rast.nmpdr.org/seedviewer.cgi?page=FunctionalRole&role=Outer%20membrane%20lipoprotein%20carrier%20protein%20LolA&subsystem_name=Lipoprotein_sorting_system) |
| Cell Wall and Capsule | Cell Wall and Capsule - no subcategory | [Murein Hydrolases](https://rast.nmpdr.org/seedviewer.cgi?page=Subsystems&subsystem=Murein_Hydrolases&organism=549.408) | [Membrane-bound lytic murein transglycosylase E (EC 3.2.1.-)](https://rast.nmpdr.org/seedviewer.cgi?page=FunctionalRole&role=Membrane-bound%20lytic%20murein%20transglycosylase%20E%20(EC%203.2.1.-)&subsystem_name=Murein_Hydrolases) |
| Cell Wall and Capsule | Cell Wall and Capsule - no subcategory | [Murein Hydrolases](https://rast.nmpdr.org/seedviewer.cgi?page=Subsystems&subsystem=Murein_Hydrolases&organism=549.408) | [Beta N-acetyl-glucosaminidase (EC 3.2.1.52)](https://rast.nmpdr.org/seedviewer.cgi?page=FunctionalRole&role=Beta%20N-acetyl-glucosaminidase%20(EC%203.2.1.52)&subsystem_name=Murein_Hydrolases) |
| Cell Wall and Capsule | Cell Wall and Capsule - no subcategory | [Murein Hydrolases](https://rast.nmpdr.org/seedviewer.cgi?page=Subsystems&subsystem=Murein_Hydrolases&organism=549.408) | [N-acetylmuramoyl-L-alanine amidase (EC 3.5.1.28)](https://rast.nmpdr.org/seedviewer.cgi?page=FunctionalRole&role=N-acetylmuramoyl-L-alanine%20amidase%20(EC%203.5.1.28)&subsystem_name=Murein_Hydrolases) |
| Cell Wall and Capsule | Cell Wall and Capsule - no subcategory | [Murein Hydrolases](https://rast.nmpdr.org/seedviewer.cgi?page=Subsystems&subsystem=Murein_Hydrolases&organism=549.408) | [D-alanyl-D-alanine carboxypeptidase (EC 3.4.16.4)](https://rast.nmpdr.org/seedviewer.cgi?page=FunctionalRole&role=D-alanyl-D-alanine%20carboxypeptidase%20(EC%203.4.16.4)&subsystem_name=Murein_Hydrolases) |
| Cell Wall and Capsule | Cell Wall and Capsule - no subcategory | [Murein Hydrolases](https://rast.nmpdr.org/seedviewer.cgi?page=Subsystems&subsystem=Murein_Hydrolases&organism=549.408) | [Membrane-bound lytic murein transglycosylase C precursor (EC 3.2.1.-)](https://rast.nmpdr.org/seedviewer.cgi?page=FunctionalRole&role=Membrane-bound%20lytic%20murein%20transglycosylase%20C%20precursor%20(EC%203.2.1.-)&subsystem_name=Murein_Hydrolases) |
| Cell Wall and Capsule | Cell Wall and Capsule - no subcategory | [Murein Hydrolases](https://rast.nmpdr.org/seedviewer.cgi?page=Subsystems&subsystem=Murein_Hydrolases&organism=549.408) | [Muramoyltetrapeptide carboxypeptidase (EC 3.4.17.13)](https://rast.nmpdr.org/seedviewer.cgi?page=FunctionalRole&role=Muramoyltetrapeptide%20carboxypeptidase%20(EC%203.4.17.13)&subsystem_name=Murein_Hydrolases) |
| Cell Wall and Capsule | Cell Wall and Capsule - no subcategory | [Murein Hydrolases](https://rast.nmpdr.org/seedviewer.cgi?page=Subsystems&subsystem=Murein_Hydrolases&organism=549.408) | [Membrane-bound lytic murein transglycosylase D precursor (EC 3.2.1.-)](https://rast.nmpdr.org/seedviewer.cgi?page=FunctionalRole&role=Membrane-bound%20lytic%20murein%20transglycosylase%20D%20precursor%20(EC%203.2.1.-)&subsystem_name=Murein_Hydrolases) |
| Cell Wall and Capsule | Cell Wall and Capsule - no subcategory | [Murein Hydrolases](https://rast.nmpdr.org/seedviewer.cgi?page=Subsystems&subsystem=Murein_Hydrolases&organism=549.408) | [Soluble lytic murein transglycosylase precursor (EC 3.2.1.-)](https://rast.nmpdr.org/seedviewer.cgi?page=FunctionalRole&role=Soluble%20lytic%20murein%20transglycosylase%20precursor%20(EC%203.2.1.-)&subsystem_name=Murein_Hydrolases) |
| Cell Wall and Capsule | Cell Wall and Capsule - no subcategory | [Murein Hydrolases](https://rast.nmpdr.org/seedviewer.cgi?page=Subsystems&subsystem=Murein_Hydrolases&organism=549.408) | [Membrane-bound lytic murein transglycosylase A precursor (EC 3.2.1.-)](https://rast.nmpdr.org/seedviewer.cgi?page=FunctionalRole&role=Membrane-bound%20lytic%20murein%20transglycosylase%20A%20precursor%20(EC%203.2.1.-)&subsystem_name=Murein_Hydrolases) |
| Cell Wall and Capsule | Cell Wall and Capsule - no subcategory | [Murein Hydrolases](https://rast.nmpdr.org/seedviewer.cgi?page=Subsystems&subsystem=Murein_Hydrolases&organism=549.408) | [Membrane-bound lytic murein transglycosylase B precursor (EC 3.2.1.-)](https://rast.nmpdr.org/seedviewer.cgi?page=FunctionalRole&role=Membrane-bound%20lytic%20murein%20transglycosylase%20B%20precursor%20(EC%203.2.1.-)&subsystem_name=Murein_Hydrolases) |
| Cell Wall and Capsule | Cell Wall and Capsule - no subcategory | [UDP-N-acetylmuramate from Fructose-6-phosphate Biosynthesis](https://rast.nmpdr.org/seedviewer.cgi?page=Subsystems&subsystem=UDP-N-acetylmuramate_from_Fructose-6-phosphate_Biosynthesis&organism=549.408) | [UDP-N-acetylenolpyruvoylglucosamine reductase (EC 1.1.1.158)](https://rast.nmpdr.org/seedviewer.cgi?page=FunctionalRole&role=UDP-N-acetylenolpyruvoylglucosamine%20reductase%20(EC%201.1.1.158)&subsystem_name=UDP-N-acetylmuramate_from_Fructose-6-phosphate_Biosynthesis) |
| Cell Wall and Capsule | Cell Wall and Capsule - no subcategory | [UDP-N-acetylmuramate from Fructose-6-phosphate Biosynthesis](https://rast.nmpdr.org/seedviewer.cgi?page=Subsystems&subsystem=UDP-N-acetylmuramate_from_Fructose-6-phosphate_Biosynthesis&organism=549.408) | [Glucosamine-1-phosphate N-acetyltransferase (EC 2.3.1.157)](https://rast.nmpdr.org/seedviewer.cgi?page=FunctionalRole&role=Glucosamine-1-phosphate%20N-acetyltransferase%20(EC%202.3.1.157)&subsystem_name=UDP-N-acetylmuramate_from_Fructose-6-phosphate_Biosynthesis) |
| Cell Wall and Capsule | Cell Wall and Capsule - no subcategory | [UDP-N-acetylmuramate from Fructose-6-phosphate Biosynthesis](https://rast.nmpdr.org/seedviewer.cgi?page=Subsystems&subsystem=UDP-N-acetylmuramate_from_Fructose-6-phosphate_Biosynthesis&organism=549.408) | [Glucosamine--fructose-6-phosphate aminotransferase [isomerizing] (EC 2.6.1.16)](https://rast.nmpdr.org/seedviewer.cgi?page=FunctionalRole&role=Glucosamine--fructose-6-phosphate%20aminotransferase%20%5bisomerizing%5d%20(EC%202.6.1.16)&subsystem_name=UDP-N-acetylmuramate_from_Fructose-6-phosphate_Biosynthesis) |
| Cell Wall and Capsule | Cell Wall and Capsule - no subcategory | [UDP-N-acetylmuramate from Fructose-6-phosphate Biosynthesis](https://rast.nmpdr.org/seedviewer.cgi?page=Subsystems&subsystem=UDP-N-acetylmuramate_from_Fructose-6-phosphate_Biosynthesis&organism=549.408) | [UDP-N-acetylglucosamine 1-carboxyvinyltransferase (EC 2.5.1.7)](https://rast.nmpdr.org/seedviewer.cgi?page=FunctionalRole&role=UDP-N-acetylglucosamine%201-carboxyvinyltransferase%20(EC%202.5.1.7)&subsystem_name=UDP-N-acetylmuramate_from_Fructose-6-phosphate_Biosynthesis) |
| Cell Wall and Capsule | Cell Wall and Capsule - no subcategory | [UDP-N-acetylmuramate from Fructose-6-phosphate Biosynthesis](https://rast.nmpdr.org/seedviewer.cgi?page=Subsystems&subsystem=UDP-N-acetylmuramate_from_Fructose-6-phosphate_Biosynthesis&organism=549.408) | [Phosphoglucosamine mutase (EC 5.4.2.10)](https://rast.nmpdr.org/seedviewer.cgi?page=FunctionalRole&role=Phosphoglucosamine%20mutase%20(EC%205.4.2.10)&subsystem_name=UDP-N-acetylmuramate_from_Fructose-6-phosphate_Biosynthesis) |
| Cell Wall and Capsule | Cell Wall and Capsule - no subcategory | [UDP-N-acetylmuramate from Fructose-6-phosphate Biosynthesis](https://rast.nmpdr.org/seedviewer.cgi?page=Subsystems&subsystem=UDP-N-acetylmuramate_from_Fructose-6-phosphate_Biosynthesis&organism=549.408) | [N-acetylglucosamine-1-phosphate uridyltransferase (EC 2.7.7.23)](https://rast.nmpdr.org/seedviewer.cgi?page=FunctionalRole&role=N-acetylglucosamine-1-phosphate%20uridyltransferase%20(EC%202.7.7.23)&subsystem_name=UDP-N-acetylmuramate_from_Fructose-6-phosphate_Biosynthesis) |
| Cell Wall and Capsule | Cell Wall and Capsule - no subcategory | [Peptidoglycan Crosslinking of Peptide Stems](https://rast.nmpdr.org/seedviewer.cgi?page=Subsystems&subsystem=Peptidoglycan_Crosslinking_of_Peptide_Stems&organism=549.408) | [L,D-transpeptidase YcfS](https://rast.nmpdr.org/seedviewer.cgi?page=FunctionalRole&role=L,D-transpeptidase%20YcfS&subsystem_name=Peptidoglycan_Crosslinking_of_Peptide_Stems) |
| Cell Wall and Capsule | Cell Wall and Capsule - no subcategory | [Peptidoglycan Crosslinking of Peptide Stems](https://rast.nmpdr.org/seedviewer.cgi?page=Subsystems&subsystem=Peptidoglycan_Crosslinking_of_Peptide_Stems&organism=549.408) | [Penicillin-binding protein AmpH](https://rast.nmpdr.org/seedviewer.cgi?page=FunctionalRole&role=Penicillin-binding%20protein%20AmpH&subsystem_name=Peptidoglycan_Crosslinking_of_Peptide_Stems) |
| Cell Wall and Capsule | Cell Wall and Capsule - no subcategory | [Peptidoglycan Crosslinking of Peptide Stems](https://rast.nmpdr.org/seedviewer.cgi?page=Subsystems&subsystem=Peptidoglycan_Crosslinking_of_Peptide_Stems&organism=549.408) | [L,D-transpeptidase YcbB](https://rast.nmpdr.org/seedviewer.cgi?page=FunctionalRole&role=L,D-transpeptidase%20YcbB&subsystem_name=Peptidoglycan_Crosslinking_of_Peptide_Stems) |
| Cell Wall and Capsule | Cell Wall and Capsule - no subcategory | [Peptidoglycan Crosslinking of Peptide Stems](https://rast.nmpdr.org/seedviewer.cgi?page=Subsystems&subsystem=Peptidoglycan_Crosslinking_of_Peptide_Stems&organism=549.408) | [L,D-transpeptidase YbiS](https://rast.nmpdr.org/seedviewer.cgi?page=FunctionalRole&role=L,D-transpeptidase%20YbiS&subsystem_name=Peptidoglycan_Crosslinking_of_Peptide_Stems) |
| Cell Wall and Capsule | Cell Wall and Capsule - no subcategory | [YjeE](https://rast.nmpdr.org/seedviewer.cgi?page=Subsystems&subsystem=YjeE&organism=549.408) | [TsaB protein, required for threonylcarbamoyladenosine (t(6)A) formation in tRNA](https://rast.nmpdr.org/seedviewer.cgi?page=FunctionalRole&role=TsaB%20protein,%20required%20for%20threonylcarbamoyladenosine%20(t(6)A)%20formation%20in%20tRNA&subsystem_name=YjeE) |
| Cell Wall and Capsule | Cell Wall and Capsule - no subcategory | [YjeE](https://rast.nmpdr.org/seedviewer.cgi?page=Subsystems&subsystem=YjeE&organism=549.408) | [NAD(P)HX epimerase](https://rast.nmpdr.org/seedviewer.cgi?page=FunctionalRole&role=NAD(P)HX%20epimerase&subsystem_name=YjeE) |
| Cell Wall and Capsule | Cell Wall and Capsule - no subcategory | [YjeE](https://rast.nmpdr.org/seedviewer.cgi?page=Subsystems&subsystem=YjeE&organism=549.408) | [TsaE protein, required for threonylcarbamoyladenosine t(6)A37 formation in tRNA](https://rast.nmpdr.org/seedviewer.cgi?page=FunctionalRole&role=TsaE%20protein,%20required%20for%20threonylcarbamoyladenosine%20t(6)A37%20formation%20in%20tRNA&subsystem_name=YjeE) |
| Cell Wall and Capsule | Cell Wall and Capsule - no subcategory | [YjeE](https://rast.nmpdr.org/seedviewer.cgi?page=Subsystems&subsystem=YjeE&organism=549.408) | [NAD(P)HX dehydratase](https://rast.nmpdr.org/seedviewer.cgi?page=FunctionalRole&role=NAD(P)HX%20dehydratase&subsystem_name=YjeE) |
| Cell Wall and Capsule | Cell Wall and Capsule - no subcategory | [Recycling of Peptidoglycan Amino Sugars](https://rast.nmpdr.org/seedviewer.cgi?page=Subsystems&subsystem=Recycling_of_Peptidoglycan_Amino_Sugars&organism=549.408) | [Beta N-acetyl-glucosaminidase (EC 3.2.1.52)](https://rast.nmpdr.org/seedviewer.cgi?page=FunctionalRole&role=Beta%20N-acetyl-glucosaminidase%20(EC%203.2.1.52)&subsystem_name=Recycling_of_Peptidoglycan_Amino_Sugars) |
| Cell Wall and Capsule | Cell Wall and Capsule - no subcategory | [Recycling of Peptidoglycan Amino Sugars](https://rast.nmpdr.org/seedviewer.cgi?page=Subsystems&subsystem=Recycling_of_Peptidoglycan_Amino_Sugars&organism=549.408) | [Anhydro-N-acetylmuramic acid kinase (EC 2.7.1.-)](https://rast.nmpdr.org/seedviewer.cgi?page=FunctionalRole&role=Anhydro-N-acetylmuramic%20acid%20kinase%20(EC%202.7.1.-)&subsystem_name=Recycling_of_Peptidoglycan_Amino_Sugars) |
| Cell Wall and Capsule | Cell Wall and Capsule - no subcategory | [Recycling of Peptidoglycan Amino Acids](https://rast.nmpdr.org/seedviewer.cgi?page=Subsystems&subsystem=Recycling_of_Peptidoglycan_Amino_Acids&organism=549.408) | [UDP-N-acetylmuramate:L-alanyl-gamma-D-glutamyl-meso-diaminopimelate ligase (EC 6.3.2.-)](https://rast.nmpdr.org/seedviewer.cgi?page=FunctionalRole&role=UDP-N-acetylmuramate:L-alanyl-gamma-D-glutamyl-meso-diaminopimelate%20ligase%20(EC%206.3.2.-)&subsystem_name=Recycling_of_Peptidoglycan_Amino_Acids) |
| Cell Wall and Capsule | Cell Wall and Capsule - no subcategory | [Recycling of Peptidoglycan Amino Acids](https://rast.nmpdr.org/seedviewer.cgi?page=Subsystems&subsystem=Recycling_of_Peptidoglycan_Amino_Acids&organism=549.408) | [L-alanine-DL-glutamate epimerase](https://rast.nmpdr.org/seedviewer.cgi?page=FunctionalRole&role=L-alanine-DL-glutamate%20epimerase&subsystem_name=Recycling_of_Peptidoglycan_Amino_Acids) |
| Cell Wall and Capsule | Cell Wall and Capsule - no subcategory | [Recycling of Peptidoglycan Amino Acids](https://rast.nmpdr.org/seedviewer.cgi?page=Subsystems&subsystem=Recycling_of_Peptidoglycan_Amino_Acids&organism=549.408) | [N-acetylmuramoyl-L-alanine amidase (EC 3.5.1.28)](https://rast.nmpdr.org/seedviewer.cgi?page=FunctionalRole&role=N-acetylmuramoyl-L-alanine%20amidase%20(EC%203.5.1.28)&subsystem_name=Recycling_of_Peptidoglycan_Amino_Acids) |
| Cell Wall and Capsule | Cell Wall and Capsule - no subcategory | [Recycling of Peptidoglycan Amino Acids](https://rast.nmpdr.org/seedviewer.cgi?page=Subsystems&subsystem=Recycling_of_Peptidoglycan_Amino_Acids&organism=549.408) | [Gamma-D-Glutamyl-meso-Diaminopimelate Amidase](https://rast.nmpdr.org/seedviewer.cgi?page=FunctionalRole&role=Gamma-D-Glutamyl-meso-Diaminopimelate%20Amidase&subsystem_name=Recycling_of_Peptidoglycan_Amino_Acids) |
| Cell Wall and Capsule | Cell Wall and Capsule - no subcategory | [Recycling of Peptidoglycan Amino Acids](https://rast.nmpdr.org/seedviewer.cgi?page=Subsystems&subsystem=Recycling_of_Peptidoglycan_Amino_Acids&organism=549.408) | [AmpG permease](https://rast.nmpdr.org/seedviewer.cgi?page=FunctionalRole&role=AmpG%20permease&subsystem_name=Recycling_of_Peptidoglycan_Amino_Acids) |
| Cell Wall and Capsule | Cell Wall and Capsule - no subcategory | [Recycling of Peptidoglycan Amino Acids](https://rast.nmpdr.org/seedviewer.cgi?page=Subsystems&subsystem=Recycling_of_Peptidoglycan_Amino_Acids&organism=549.408) | [Periplasmic Murein Peptide-Binding Protein MppA](https://rast.nmpdr.org/seedviewer.cgi?page=FunctionalRole&role=Periplasmic%20Murein%20Peptide-Binding%20Protein%20MppA&subsystem_name=Recycling_of_Peptidoglycan_Amino_Acids) |
| Cell Wall and Capsule | Cell Wall and Capsule - no subcategory | [Recycling of Peptidoglycan Amino Acids](https://rast.nmpdr.org/seedviewer.cgi?page=Subsystems&subsystem=Recycling_of_Peptidoglycan_Amino_Acids&organism=549.408) | [Muramoyltetrapeptide carboxypeptidase (EC 3.4.17.13)](https://rast.nmpdr.org/seedviewer.cgi?page=FunctionalRole&role=Muramoyltetrapeptide%20carboxypeptidase%20(EC%203.4.17.13)&subsystem_name=Recycling_of_Peptidoglycan_Amino_Acids) |
| Cell Wall and Capsule | Cell Wall and Capsule - no subcategory | [Recycling of Peptidoglycan Amino Acids](https://rast.nmpdr.org/seedviewer.cgi?page=Subsystems&subsystem=Recycling_of_Peptidoglycan_Amino_Acids&organism=549.408) | [N-acetylmuramoyl-L-alanine amidase (EC 3.5.1.28) AmpD](https://rast.nmpdr.org/seedviewer.cgi?page=FunctionalRole&role=N-acetylmuramoyl-L-alanine%20amidase%20(EC%203.5.1.28)%20AmpD&subsystem_name=Recycling_of_Peptidoglycan_Amino_Acids) |
| Cell Wall and Capsule | Cell Wall and Capsule - no subcategory | [Recycling of Peptidoglycan Amino Acids](https://rast.nmpdr.org/seedviewer.cgi?page=Subsystems&subsystem=Recycling_of_Peptidoglycan_Amino_Acids&organism=549.408) | [Aminoacyl-histidine dipeptidase (Peptidase D) (EC 3.4.13.3)](https://rast.nmpdr.org/seedviewer.cgi?page=FunctionalRole&role=Aminoacyl-histidine%20dipeptidase%20(Peptidase%20D)%20(EC%203.4.13.3)&subsystem_name=Recycling_of_Peptidoglycan_Amino_Acids) |
| Cell Wall and Capsule | Cell Wall and Capsule - no subcategory | [Peptidoglycan Biosynthesis](https://rast.nmpdr.org/seedviewer.cgi?page=Subsystems&subsystem=Peptidoglycan_Biosynthesis&organism=549.408) | [Glucosamine-1-phosphate N-acetyltransferase (EC 2.3.1.157)](https://rast.nmpdr.org/seedviewer.cgi?page=FunctionalRole&role=Glucosamine-1-phosphate%20N-acetyltransferase%20(EC%202.3.1.157)&subsystem_name=Peptidoglycan_Biosynthesis) |
| Cell Wall and Capsule | Cell Wall and Capsule - no subcategory | [Peptidoglycan Biosynthesis](https://rast.nmpdr.org/seedviewer.cgi?page=Subsystems&subsystem=Peptidoglycan_Biosynthesis&organism=549.408) | [UDP-N-acetylmuramoylalanyl-D-glutamate--2,6-diaminopimelate ligase (EC 6.3.2.13)](https://rast.nmpdr.org/seedviewer.cgi?page=FunctionalRole&role=UDP-N-acetylmuramoylalanyl-D-glutamate--2,6-diaminopimelate%20ligase%20(EC%206.3.2.13)&subsystem_name=Peptidoglycan_Biosynthesis) |
| Cell Wall and Capsule | Cell Wall and Capsule - no subcategory | [Peptidoglycan Biosynthesis](https://rast.nmpdr.org/seedviewer.cgi?page=Subsystems&subsystem=Peptidoglycan_Biosynthesis&organism=549.408) | [Monofunctional biosynthetic peptidoglycan transglycosylase (EC 2.4.2.-)](https://rast.nmpdr.org/seedviewer.cgi?page=FunctionalRole&role=Monofunctional%20biosynthetic%20peptidoglycan%20transglycosylase%20(EC%202.4.2.-)&subsystem_name=Peptidoglycan_Biosynthesis) |
| Cell Wall and Capsule | Cell Wall and Capsule - no subcategory | [Peptidoglycan Biosynthesis](https://rast.nmpdr.org/seedviewer.cgi?page=Subsystems&subsystem=Peptidoglycan_Biosynthesis&organism=549.408) | [D-alanyl-D-alanine carboxypeptidase (EC 3.4.16.4)](https://rast.nmpdr.org/seedviewer.cgi?page=FunctionalRole&role=D-alanyl-D-alanine%20carboxypeptidase%20(EC%203.4.16.4)&subsystem_name=Peptidoglycan_Biosynthesis) |
| Cell Wall and Capsule | Cell Wall and Capsule - no subcategory | [Peptidoglycan Biosynthesis](https://rast.nmpdr.org/seedviewer.cgi?page=Subsystems&subsystem=Peptidoglycan_Biosynthesis&organism=549.408) | [Cell division protein FtsI [Peptidoglycan synthetase] (EC 2.4.1.129)](https://rast.nmpdr.org/seedviewer.cgi?page=FunctionalRole&role=Cell%20division%20protein%20FtsI%20%5bPeptidoglycan%20synthetase%5d%20(EC%202.4.1.129)&subsystem_name=Peptidoglycan_Biosynthesis) |
| Cell Wall and Capsule | Cell Wall and Capsule - no subcategory | [Peptidoglycan Biosynthesis](https://rast.nmpdr.org/seedviewer.cgi?page=Subsystems&subsystem=Peptidoglycan_Biosynthesis&organism=549.408) | [UDP-N-acetylenolpyruvoylglucosamine reductase (EC 1.1.1.158)](https://rast.nmpdr.org/seedviewer.cgi?page=FunctionalRole&role=UDP-N-acetylenolpyruvoylglucosamine%20reductase%20(EC%201.1.1.158)&subsystem_name=Peptidoglycan_Biosynthesis) |
| Cell Wall and Capsule | Cell Wall and Capsule - no subcategory | [Peptidoglycan Biosynthesis](https://rast.nmpdr.org/seedviewer.cgi?page=Subsystems&subsystem=Peptidoglycan_Biosynthesis&organism=549.408) | [Penicillin-insensitive transglycosylase (EC 2.4.2.-) & transpeptidase PBP-1C](https://rast.nmpdr.org/seedviewer.cgi?page=FunctionalRole&role=Penicillin-insensitive%20transglycosylase%20(EC%202.4.2.-)%20&%20transpeptidase%20PBP-1C&subsystem_name=Peptidoglycan_Biosynthesis) |
| Cell Wall and Capsule | Cell Wall and Capsule - no subcategory | [Peptidoglycan Biosynthesis](https://rast.nmpdr.org/seedviewer.cgi?page=Subsystems&subsystem=Peptidoglycan_Biosynthesis&organism=549.408) | [Glutamine synthetase type I (EC 6.3.1.2)](https://rast.nmpdr.org/seedviewer.cgi?page=FunctionalRole&role=Glutamine%20synthetase%20type%20I%20(EC%206.3.1.2)&subsystem_name=Peptidoglycan_Biosynthesis) |
| Cell Wall and Capsule | Cell Wall and Capsule - no subcategory | [Peptidoglycan Biosynthesis](https://rast.nmpdr.org/seedviewer.cgi?page=Subsystems&subsystem=Peptidoglycan_Biosynthesis&organism=549.408) | [Rare lipoprotein A precursor](https://rast.nmpdr.org/seedviewer.cgi?page=FunctionalRole&role=Rare%20lipoprotein%20A%20precursor&subsystem_name=Peptidoglycan_Biosynthesis) |
| Cell Wall and Capsule | Cell Wall and Capsule - no subcategory | [Peptidoglycan Biosynthesis](https://rast.nmpdr.org/seedviewer.cgi?page=Subsystems&subsystem=Peptidoglycan_Biosynthesis&organism=549.408) | [UDP-N-acetylglucosamine 1-carboxyvinyltransferase (EC 2.5.1.7)](https://rast.nmpdr.org/seedviewer.cgi?page=FunctionalRole&role=UDP-N-acetylglucosamine%201-carboxyvinyltransferase%20(EC%202.5.1.7)&subsystem_name=Peptidoglycan_Biosynthesis) |
| Cell Wall and Capsule | Cell Wall and Capsule - no subcategory | [Peptidoglycan Biosynthesis](https://rast.nmpdr.org/seedviewer.cgi?page=Subsystems&subsystem=Peptidoglycan_Biosynthesis&organism=549.408) | [Membrane-bound lytic murein transglycosylase B precursor (EC 3.2.1.-)](https://rast.nmpdr.org/seedviewer.cgi?page=FunctionalRole&role=Membrane-bound%20lytic%20murein%20transglycosylase%20B%20precursor%20(EC%203.2.1.-)&subsystem_name=Peptidoglycan_Biosynthesis) |
| Cell Wall and Capsule | Cell Wall and Capsule - no subcategory | [Peptidoglycan Biosynthesis](https://rast.nmpdr.org/seedviewer.cgi?page=Subsystems&subsystem=Peptidoglycan_Biosynthesis&organism=549.408) | [UDP-N-acetylmuramoylalanine--D-glutamate ligase (EC 6.3.2.9)](https://rast.nmpdr.org/seedviewer.cgi?page=FunctionalRole&role=UDP-N-acetylmuramoylalanine--D-glutamate%20ligase%20(EC%206.3.2.9)&subsystem_name=Peptidoglycan_Biosynthesis) |
| Cell Wall and Capsule | Cell Wall and Capsule - no subcategory | [Peptidoglycan Biosynthesis](https://rast.nmpdr.org/seedviewer.cgi?page=Subsystems&subsystem=Peptidoglycan_Biosynthesis&organism=549.408) | [UDP-N-acetylglucosamine--N-acetylmuramyl-(pentapeptide) pyrophosphoryl-undecaprenol N-acetylglucosamine transferase (EC 2.4.1.227)](https://rast.nmpdr.org/seedviewer.cgi?page=FunctionalRole&role=UDP-N-acetylglucosamine--N-acetylmuramyl-(pentapeptide)%20pyrophosphoryl-undecaprenol%20N-acetylglucosamine%20transferase%20(EC%202.4.1.227)&subsystem_name=Peptidoglycan_Biosynthesis) |
| Cell Wall and Capsule | Cell Wall and Capsule - no subcategory | [Peptidoglycan Biosynthesis](https://rast.nmpdr.org/seedviewer.cgi?page=Subsystems&subsystem=Peptidoglycan_Biosynthesis&organism=549.408) | [Rod shape-determining protein RodA](https://rast.nmpdr.org/seedviewer.cgi?page=FunctionalRole&role=Rod%20shape-determining%20protein%20RodA&subsystem_name=Peptidoglycan_Biosynthesis) |
| Cell Wall and Capsule | Cell Wall and Capsule - no subcategory | [Peptidoglycan Biosynthesis](https://rast.nmpdr.org/seedviewer.cgi?page=Subsystems&subsystem=Peptidoglycan_Biosynthesis&organism=549.408) | [UDP-N-acetylmuramoylalanyl-D-glutamyl-2,6-diaminopimelate--D-alanyl-D-alanine ligase (EC 6.3.2.10)](https://rast.nmpdr.org/seedviewer.cgi?page=FunctionalRole&role=UDP-N-acetylmuramoylalanyl-D-glutamyl-2,6-diaminopimelate--D-alanyl-D-alanine%20ligase%20(EC%206.3.2.10)&subsystem_name=Peptidoglycan_Biosynthesis) |
| Cell Wall and Capsule | Cell Wall and Capsule - no subcategory | [Peptidoglycan Biosynthesis](https://rast.nmpdr.org/seedviewer.cgi?page=Subsystems&subsystem=Peptidoglycan_Biosynthesis&organism=549.408) | [Glutamate racemase (EC 5.1.1.3)](https://rast.nmpdr.org/seedviewer.cgi?page=FunctionalRole&role=Glutamate%20racemase%20(EC%205.1.1.3)&subsystem_name=Peptidoglycan_Biosynthesis) |
| Cell Wall and Capsule | Cell Wall and Capsule - no subcategory | [Peptidoglycan Biosynthesis](https://rast.nmpdr.org/seedviewer.cgi?page=Subsystems&subsystem=Peptidoglycan_Biosynthesis&organism=549.408) | [UDP-N-acetylmuramate--alanine ligase (EC 6.3.2.8)](https://rast.nmpdr.org/seedviewer.cgi?page=FunctionalRole&role=UDP-N-acetylmuramate--alanine%20ligase%20(EC%206.3.2.8)&subsystem_name=Peptidoglycan_Biosynthesis) |
| Cell Wall and Capsule | Cell Wall and Capsule - no subcategory | [Peptidoglycan Biosynthesis](https://rast.nmpdr.org/seedviewer.cgi?page=Subsystems&subsystem=Peptidoglycan_Biosynthesis&organism=549.408) | [Penicillin-binding protein 2 (PBP-2)](https://rast.nmpdr.org/seedviewer.cgi?page=FunctionalRole&role=Penicillin-binding%20protein%202%20(PBP-2)&subsystem_name=Peptidoglycan_Biosynthesis) |
| Cell Wall and Capsule | Cell Wall and Capsule - no subcategory | [Peptidoglycan Biosynthesis](https://rast.nmpdr.org/seedviewer.cgi?page=Subsystems&subsystem=Peptidoglycan_Biosynthesis&organism=549.408) | [D-alanine--D-alanine ligase (EC 6.3.2.4)](https://rast.nmpdr.org/seedviewer.cgi?page=FunctionalRole&role=D-alanine--D-alanine%20ligase%20(EC%206.3.2.4)&subsystem_name=Peptidoglycan_Biosynthesis) |
| Cell Wall and Capsule | Cell Wall and Capsule - no subcategory | [Peptidoglycan Biosynthesis](https://rast.nmpdr.org/seedviewer.cgi?page=Subsystems&subsystem=Peptidoglycan_Biosynthesis&organism=549.408) | [Phospho-N-acetylmuramoyl-pentapeptide-transferase (EC 2.7.8.13)](https://rast.nmpdr.org/seedviewer.cgi?page=FunctionalRole&role=Phospho-N-acetylmuramoyl-pentapeptide-transferase%20(EC%202.7.8.13)&subsystem_name=Peptidoglycan_Biosynthesis) |
| Cell Wall and Capsule | Cell Wall and Capsule - no subcategory | [Peptidoglycan Biosynthesis](https://rast.nmpdr.org/seedviewer.cgi?page=Subsystems&subsystem=Peptidoglycan_Biosynthesis&organism=549.408) | [N-acetylglucosamine-1-phosphate uridyltransferase (EC 2.7.7.23)](https://rast.nmpdr.org/seedviewer.cgi?page=FunctionalRole&role=N-acetylglucosamine-1-phosphate%20uridyltransferase%20(EC%202.7.7.23)&subsystem_name=Peptidoglycan_Biosynthesis) |
| Cell Wall and Capsule | Cell Wall and Capsule - no subcategory | [Peptidoglycan Biosynthesis](https://rast.nmpdr.org/seedviewer.cgi?page=Subsystems&subsystem=Peptidoglycan_Biosynthesis&organism=549.408) | [Multimodular transpeptidase-transglycosylase (EC 2.4.1.129) (EC 3.4.-.-)](https://rast.nmpdr.org/seedviewer.cgi?page=FunctionalRole&role=Multimodular%20transpeptidase-transglycosylase%20(EC%202.4.1.129)%20(EC%203.4.-.-)&subsystem_name=Peptidoglycan_Biosynthesis) |
| Cell Wall and Capsule | Cell Wall and Capsule - no subcategory | [Peptidoglycan Biosynthesis](https://rast.nmpdr.org/seedviewer.cgi?page=Subsystems&subsystem=Peptidoglycan_Biosynthesis&organism=549.408) | [Murein-DD-endopeptidase (EC 3.4.99.-)](https://rast.nmpdr.org/seedviewer.cgi?page=FunctionalRole&role=Murein-DD-endopeptidase%20(EC%203.4.99.-)&subsystem_name=Peptidoglycan_Biosynthesis) |
| Cell Wall and Capsule | Cell Wall and Capsule - no subcategory | [Peptidoglycan biosynthesis--gjo](https://rast.nmpdr.org/seedviewer.cgi?page=Subsystems&subsystem=Peptidoglycan_biosynthesis--gjo&organism=549.408) | [UDP-N-acetylmuramate:L-alanyl-gamma-D-glutamyl-meso-diaminopimelate ligase (EC 6.3.2.-)](https://rast.nmpdr.org/seedviewer.cgi?page=FunctionalRole&role=UDP-N-acetylmuramate:L-alanyl-gamma-D-glutamyl-meso-diaminopimelate%20ligase%20(EC%206.3.2.-)&subsystem_name=Peptidoglycan_biosynthesis--gjo) |
| Cell Wall and Capsule | Cell Wall and Capsule - no subcategory | [Peptidoglycan biosynthesis--gjo](https://rast.nmpdr.org/seedviewer.cgi?page=Subsystems&subsystem=Peptidoglycan_biosynthesis--gjo&organism=549.408) | [UDP-N-acetylmuramate--alanine ligase (EC 6.3.2.8)](https://rast.nmpdr.org/seedviewer.cgi?page=FunctionalRole&role=UDP-N-acetylmuramate--alanine%20ligase%20(EC%206.3.2.8)&subsystem_name=Peptidoglycan_biosynthesis--gjo) |
| Cell Wall and Capsule | Cell Wall and Capsule - no subcategory | [Peptidoglycan biosynthesis--gjo](https://rast.nmpdr.org/seedviewer.cgi?page=Subsystems&subsystem=Peptidoglycan_biosynthesis--gjo&organism=549.408) | [UDP-N-acetylmuramoylalanine--D-glutamate ligase (EC 6.3.2.9)](https://rast.nmpdr.org/seedviewer.cgi?page=FunctionalRole&role=UDP-N-acetylmuramoylalanine--D-glutamate%20ligase%20(EC%206.3.2.9)&subsystem_name=Peptidoglycan_biosynthesis--gjo) |
| Cell Wall and Capsule | Cell Wall and Capsule - no subcategory | [Peptidoglycan biosynthesis--gjo](https://rast.nmpdr.org/seedviewer.cgi?page=Subsystems&subsystem=Peptidoglycan_biosynthesis--gjo&organism=549.408) | [UDP-N-acetylmuramoylalanyl-D-glutamate--2,6-diaminopimelate ligase (EC 6.3.2.13)](https://rast.nmpdr.org/seedviewer.cgi?page=FunctionalRole&role=UDP-N-acetylmuramoylalanyl-D-glutamate--2,6-diaminopimelate%20ligase%20(EC%206.3.2.13)&subsystem_name=Peptidoglycan_biosynthesis--gjo) |
| Cell Wall and Capsule | Cell Wall and Capsule - no subcategory | [Peptidoglycan biosynthesis--gjo](https://rast.nmpdr.org/seedviewer.cgi?page=Subsystems&subsystem=Peptidoglycan_biosynthesis--gjo&organism=549.408) | [D-alanine--D-alanine ligase (EC 6.3.2.4)](https://rast.nmpdr.org/seedviewer.cgi?page=FunctionalRole&role=D-alanine--D-alanine%20ligase%20(EC%206.3.2.4)&subsystem_name=Peptidoglycan_biosynthesis--gjo) |
| Cell Wall and Capsule | Cell Wall and Capsule - no subcategory | [Peptidoglycan biosynthesis--gjo](https://rast.nmpdr.org/seedviewer.cgi?page=Subsystems&subsystem=Peptidoglycan_biosynthesis--gjo&organism=549.408) | [UDP-N-acetylmuramoylalanyl-D-glutamyl-2,6-diaminopimelate--D-alanyl-D-alanine ligase (EC 6.3.2.10)](https://rast.nmpdr.org/seedviewer.cgi?page=FunctionalRole&role=UDP-N-acetylmuramoylalanyl-D-glutamyl-2,6-diaminopimelate--D-alanyl-D-alanine%20ligase%20(EC%206.3.2.10)&subsystem_name=Peptidoglycan_biosynthesis--gjo) |
| Virulence, Disease and Defense | Adhesion | [Mediator of hyperadherence YidE in Enterobacteria and its conserved region](https://rast.nmpdr.org/seedviewer.cgi?page=Subsystems&subsystem=Mediator_of_hyperadherence_YidE_in_Enterobacteria_and_its_conserved_region&organism=549.408) | [16 kDa heat shock protein A](https://rast.nmpdr.org/seedviewer.cgi?page=FunctionalRole&role=16%20kDa%20heat%20shock%20protein%20A&subsystem_name=Mediator_of_hyperadherence_YidE_in_Enterobacteria_and_its_conserved_region) |
| Virulence, Disease and Defense | Adhesion | [Mediator of hyperadherence YidE in Enterobacteria and its conserved region](https://rast.nmpdr.org/seedviewer.cgi?page=Subsystems&subsystem=Mediator_of_hyperadherence_YidE_in_Enterobacteria_and_its_conserved_region&organism=549.408) | [Uncharacterized protein YidR](https://rast.nmpdr.org/seedviewer.cgi?page=FunctionalRole&role=Uncharacterized%20protein%20YidR&subsystem_name=Mediator_of_hyperadherence_YidE_in_Enterobacteria_and_its_conserved_region) |
| Virulence, Disease and Defense | Adhesion | [Mediator of hyperadherence YidE in Enterobacteria and its conserved region](https://rast.nmpdr.org/seedviewer.cgi?page=Subsystems&subsystem=Mediator_of_hyperadherence_YidE_in_Enterobacteria_and_its_conserved_region&organism=549.408) | [Outer membrane lipoprotein YidQ](https://rast.nmpdr.org/seedviewer.cgi?page=FunctionalRole&role=Outer%20membrane%20lipoprotein%20YidQ&subsystem_name=Mediator_of_hyperadherence_YidE_in_Enterobacteria_and_its_conserved_region) |
| Virulence, Disease and Defense | Bacteriocins, ribosomally synthesized antibacterial peptides | [Tolerance to colicin E2](https://rast.nmpdr.org/seedviewer.cgi?page=Subsystems&subsystem=Tolerance_to_colicin_E2&organism=549.408) | [Conserved uncharacterized protein CreA](https://rast.nmpdr.org/seedviewer.cgi?page=FunctionalRole&role=Conserved%20uncharacterized%20protein%20CreA&subsystem_name=Tolerance_to_colicin_E2) |
| Virulence, Disease and Defense | Bacteriocins, ribosomally synthesized antibacterial peptides | [Colicin V and Bacteriocin Production Cluster](https://rast.nmpdr.org/seedviewer.cgi?page=Subsystems&subsystem=Colicin_V_and_Bacteriocin_Production_Cluster&organism=549.408) | [Folylpolyglutamate synthase (EC 6.3.2.17)](https://rast.nmpdr.org/seedviewer.cgi?page=FunctionalRole&role=Folylpolyglutamate%20synthase%20(EC%206.3.2.17)&subsystem_name=Colicin_V_and_Bacteriocin_Production_Cluster) |
| Virulence, Disease and Defense | Bacteriocins, ribosomally synthesized antibacterial peptides | [Colicin V and Bacteriocin Production Cluster](https://rast.nmpdr.org/seedviewer.cgi?page=Subsystems&subsystem=Colicin_V_and_Bacteriocin_Production_Cluster&organism=549.408) | [Amidophosphoribosyltransferase (EC 2.4.2.14)](https://rast.nmpdr.org/seedviewer.cgi?page=FunctionalRole&role=Amidophosphoribosyltransferase%20(EC%202.4.2.14)&subsystem_name=Colicin_V_and_Bacteriocin_Production_Cluster) |
| Virulence, Disease and Defense | Bacteriocins, ribosomally synthesized antibacterial peptides | [Colicin V and Bacteriocin Production Cluster](https://rast.nmpdr.org/seedviewer.cgi?page=Subsystems&subsystem=Colicin_V_and_Bacteriocin_Production_Cluster&organism=549.408) | [Colicin V production protein](https://rast.nmpdr.org/seedviewer.cgi?page=FunctionalRole&role=Colicin%20V%20production%20protein&subsystem_name=Colicin_V_and_Bacteriocin_Production_Cluster) |
| Virulence, Disease and Defense | Bacteriocins, ribosomally synthesized antibacterial peptides | [Colicin V and Bacteriocin Production Cluster](https://rast.nmpdr.org/seedviewer.cgi?page=Subsystems&subsystem=Colicin_V_and_Bacteriocin_Production_Cluster&organism=549.408) | [Dihydrofolate synthase (EC 6.3.2.12)](https://rast.nmpdr.org/seedviewer.cgi?page=FunctionalRole&role=Dihydrofolate%20synthase%20(EC%206.3.2.12)&subsystem_name=Colicin_V_and_Bacteriocin_Production_Cluster) |
| Virulence, Disease and Defense | Bacteriocins, ribosomally synthesized antibacterial peptides | [Colicin V and Bacteriocin Production Cluster](https://rast.nmpdr.org/seedviewer.cgi?page=Subsystems&subsystem=Colicin_V_and_Bacteriocin_Production_Cluster&organism=549.408) | [Acetyl-coenzyme A carboxyl transferase beta chain (EC 6.4.1.2)](https://rast.nmpdr.org/seedviewer.cgi?page=FunctionalRole&role=Acetyl-coenzyme%20A%20carboxyl%20transferase%20beta%20chain%20(EC%206.4.1.2)&subsystem_name=Colicin_V_and_Bacteriocin_Production_Cluster) |
| Virulence, Disease and Defense | Bacteriocins, ribosomally synthesized antibacterial peptides | [Colicin V and Bacteriocin Production Cluster](https://rast.nmpdr.org/seedviewer.cgi?page=Subsystems&subsystem=Colicin_V_and_Bacteriocin_Production_Cluster&organism=549.408) | [DedA protein](https://rast.nmpdr.org/seedviewer.cgi?page=FunctionalRole&role=DedA%20protein&subsystem_name=Colicin_V_and_Bacteriocin_Production_Cluster) |
| Virulence, Disease and Defense | Bacteriocins, ribosomally synthesized antibacterial peptides | [Colicin V and Bacteriocin Production Cluster](https://rast.nmpdr.org/seedviewer.cgi?page=Subsystems&subsystem=Colicin_V_and_Bacteriocin_Production_Cluster&organism=549.408) | [DedD protein](https://rast.nmpdr.org/seedviewer.cgi?page=FunctionalRole&role=DedD%20protein&subsystem_name=Colicin_V_and_Bacteriocin_Production_Cluster) |
| Virulence, Disease and Defense | Bacteriocins, ribosomally synthesized antibacterial peptides | [Colicin V and Bacteriocin Production Cluster](https://rast.nmpdr.org/seedviewer.cgi?page=Subsystems&subsystem=Colicin_V_and_Bacteriocin_Production_Cluster&organism=549.408) | [tRNA pseudouridine synthase A (EC 4.2.1.70)](https://rast.nmpdr.org/seedviewer.cgi?page=FunctionalRole&role=tRNA%20pseudouridine%20synthase%20A%20(EC%204.2.1.70)&subsystem_name=Colicin_V_and_Bacteriocin_Production_Cluster) |
| Virulence, Disease and Defense | Resistance to antibiotics and toxic compounds | [The mdtABCD multidrug resistance cluster](https://rast.nmpdr.org/seedviewer.cgi?page=Subsystems&subsystem=The_mdtABCD_multidrug_resistance_cluster&organism=549.408) | [Multidrug transporter MdtC](https://rast.nmpdr.org/seedviewer.cgi?page=FunctionalRole&role=Multidrug%20transporter%20MdtC&subsystem_name=The_mdtABCD_multidrug_resistance_cluster) |
| Virulence, Disease and Defense | Resistance to antibiotics and toxic compounds | [The mdtABCD multidrug resistance cluster](https://rast.nmpdr.org/seedviewer.cgi?page=Subsystems&subsystem=The_mdtABCD_multidrug_resistance_cluster&organism=549.408) | [Response regulator BaeR](https://rast.nmpdr.org/seedviewer.cgi?page=FunctionalRole&role=Response%20regulator%20BaeR&subsystem_name=The_mdtABCD_multidrug_resistance_cluster) |
| Virulence, Disease and Defense | Resistance to antibiotics and toxic compounds | [The mdtABCD multidrug resistance cluster](https://rast.nmpdr.org/seedviewer.cgi?page=Subsystems&subsystem=The_mdtABCD_multidrug_resistance_cluster&organism=549.408) | [Multidrug transporter MdtB](https://rast.nmpdr.org/seedviewer.cgi?page=FunctionalRole&role=Multidrug%20transporter%20MdtB&subsystem_name=The_mdtABCD_multidrug_resistance_cluster) |
| Virulence, Disease and Defense | Resistance to antibiotics and toxic compounds | [The mdtABCD multidrug resistance cluster](https://rast.nmpdr.org/seedviewer.cgi?page=Subsystems&subsystem=The_mdtABCD_multidrug_resistance_cluster&organism=549.408) | [Probable RND efflux membrane fusion protein](https://rast.nmpdr.org/seedviewer.cgi?page=FunctionalRole&role=Probable%20RND%20efflux%20membrane%20fusion%20protein&subsystem_name=The_mdtABCD_multidrug_resistance_cluster) |
| Virulence, Disease and Defense | Resistance to antibiotics and toxic compounds | [The mdtABCD multidrug resistance cluster](https://rast.nmpdr.org/seedviewer.cgi?page=Subsystems&subsystem=The_mdtABCD_multidrug_resistance_cluster&organism=549.408) | [Sensory histidine kinase BaeS](https://rast.nmpdr.org/seedviewer.cgi?page=FunctionalRole&role=Sensory%20histidine%20kinase%20BaeS&subsystem_name=The_mdtABCD_multidrug_resistance_cluster) |
| Virulence, Disease and Defense | Resistance to antibiotics and toxic compounds | [The mdtABCD multidrug resistance cluster](https://rast.nmpdr.org/seedviewer.cgi?page=Subsystems&subsystem=The_mdtABCD_multidrug_resistance_cluster&organism=549.408) | [Multidrug transporter MdtD](https://rast.nmpdr.org/seedviewer.cgi?page=FunctionalRole&role=Multidrug%20transporter%20MdtD&subsystem_name=The_mdtABCD_multidrug_resistance_cluster) |
| Virulence, Disease and Defense | Resistance to antibiotics and toxic compounds | [Multiple Antibiotic Resistance MAR locus](https://rast.nmpdr.org/seedviewer.cgi?page=Subsystems&subsystem=Multiple_Antibiotic_Resistance_MAR_locus&organism=549.408) | [Multiple antibiotic resistance protein MarC](https://rast.nmpdr.org/seedviewer.cgi?page=FunctionalRole&role=Multiple%20antibiotic%20resistance%20protein%20MarC&subsystem_name=Multiple_Antibiotic_Resistance_MAR_locus) |
| Virulence, Disease and Defense | Resistance to antibiotics and toxic compounds | [Copper homeostasis](https://rast.nmpdr.org/seedviewer.cgi?page=Subsystems&subsystem=Copper_homeostasis&organism=549.408) | [Cytochrome c heme lyase subunit CcmF](https://rast.nmpdr.org/seedviewer.cgi?page=FunctionalRole&role=Cytochrome%20c%20heme%20lyase%20subunit%20CcmF&subsystem_name=Copper_homeostasis) |
| Virulence, Disease and Defense | Resistance to antibiotics and toxic compounds | [Copper homeostasis](https://rast.nmpdr.org/seedviewer.cgi?page=Subsystems&subsystem=Copper_homeostasis&organism=549.408) | [Cytochrome c heme lyase subunit CcmH](https://rast.nmpdr.org/seedviewer.cgi?page=FunctionalRole&role=Cytochrome%20c%20heme%20lyase%20subunit%20CcmH&subsystem_name=Copper_homeostasis) |
| Virulence, Disease and Defense | Resistance to antibiotics and toxic compounds | [Copper homeostasis](https://rast.nmpdr.org/seedviewer.cgi?page=Subsystems&subsystem=Copper_homeostasis&organism=549.408) | [Copper-translocating P-type ATPase (EC 3.6.3.4)](https://rast.nmpdr.org/seedviewer.cgi?page=FunctionalRole&role=Copper-translocating%20P-type%20ATPase%20(EC%203.6.3.4)&subsystem_name=Copper_homeostasis) |
| Virulence, Disease and Defense | Resistance to antibiotics and toxic compounds | [Copper homeostasis](https://rast.nmpdr.org/seedviewer.cgi?page=Subsystems&subsystem=Copper_homeostasis&organism=549.408) | [Blue copper oxidase CueO precursor](https://rast.nmpdr.org/seedviewer.cgi?page=FunctionalRole&role=Blue%20copper%20oxidase%20CueO%20precursor&subsystem_name=Copper_homeostasis) |
| Virulence, Disease and Defense | Resistance to antibiotics and toxic compounds | [Copper homeostasis](https://rast.nmpdr.org/seedviewer.cgi?page=Subsystems&subsystem=Copper_homeostasis&organism=549.408) | [Copper resistance protein D](https://rast.nmpdr.org/seedviewer.cgi?page=FunctionalRole&role=Copper%20resistance%20protein%20D&subsystem_name=Copper_homeostasis) |
| Virulence, Disease and Defense | Resistance to antibiotics and toxic compounds | [Copper homeostasis](https://rast.nmpdr.org/seedviewer.cgi?page=Subsystems&subsystem=Copper_homeostasis&organism=549.408) | [Copper resistance protein C precursor](https://rast.nmpdr.org/seedviewer.cgi?page=FunctionalRole&role=Copper%20resistance%20protein%20C%20precursor&subsystem_name=Copper_homeostasis) |
| Virulence, Disease and Defense | Resistance to antibiotics and toxic compounds | [Cobalt-zinc-cadmium resistance](https://rast.nmpdr.org/seedviewer.cgi?page=Subsystems&subsystem=Cobalt-zinc-cadmium_resistance&organism=549.408) | [DNA-binding heavy metal response regulator](https://rast.nmpdr.org/seedviewer.cgi?page=FunctionalRole&role=DNA-binding%20heavy%20metal%20response%20regulator&subsystem_name=Cobalt-zinc-cadmium_resistance) |
| Virulence, Disease and Defense | Resistance to antibiotics and toxic compounds | [Cobalt-zinc-cadmium resistance](https://rast.nmpdr.org/seedviewer.cgi?page=Subsystems&subsystem=Cobalt-zinc-cadmium_resistance&organism=549.408) | [Cobalt-zinc-cadmium resistance protein](https://rast.nmpdr.org/seedviewer.cgi?page=FunctionalRole&role=Cobalt-zinc-cadmium%20resistance%20protein&subsystem_name=Cobalt-zinc-cadmium_resistance) |
| Virulence, Disease and Defense | Resistance to antibiotics and toxic compounds | [Cobalt-zinc-cadmium resistance](https://rast.nmpdr.org/seedviewer.cgi?page=Subsystems&subsystem=Cobalt-zinc-cadmium_resistance&organism=549.408) | [Cobalt-zinc-cadmium resistance protein CzcA](https://rast.nmpdr.org/seedviewer.cgi?page=FunctionalRole&role=Cobalt-zinc-cadmium%20resistance%20protein%20CzcA&subsystem_name=Cobalt-zinc-cadmium_resistance) |
| Virulence, Disease and Defense | Resistance to antibiotics and toxic compounds | [Cobalt-zinc-cadmium resistance](https://rast.nmpdr.org/seedviewer.cgi?page=Subsystems&subsystem=Cobalt-zinc-cadmium_resistance&organism=549.408) | [Zinc transporter ZitB](https://rast.nmpdr.org/seedviewer.cgi?page=FunctionalRole&role=Zinc%20transporter%20ZitB&subsystem_name=Cobalt-zinc-cadmium_resistance) |
| Virulence, Disease and Defense | Resistance to antibiotics and toxic compounds | [Cobalt-zinc-cadmium resistance](https://rast.nmpdr.org/seedviewer.cgi?page=Subsystems&subsystem=Cobalt-zinc-cadmium_resistance&organism=549.408) | [Probable Co/Zn/Cd efflux system membrane fusion protein](https://rast.nmpdr.org/seedviewer.cgi?page=FunctionalRole&role=Probable%20Co/Zn/Cd%20efflux%20system%20membrane%20fusion%20protein&subsystem_name=Cobalt-zinc-cadmium_resistance) |
| Virulence, Disease and Defense | Resistance to antibiotics and toxic compounds | [Cobalt-zinc-cadmium resistance](https://rast.nmpdr.org/seedviewer.cgi?page=Subsystems&subsystem=Cobalt-zinc-cadmium_resistance&organism=549.408) | [Transcriptional regulator, MerR family](https://rast.nmpdr.org/seedviewer.cgi?page=FunctionalRole&role=Transcriptional%20regulator,%20MerR%20family&subsystem_name=Cobalt-zinc-cadmium_resistance) |
| Virulence, Disease and Defense | Resistance to antibiotics and toxic compounds | [Cobalt-zinc-cadmium resistance](https://rast.nmpdr.org/seedviewer.cgi?page=Subsystems&subsystem=Cobalt-zinc-cadmium_resistance&organism=549.408) | [Cation efflux system protein CusA](https://rast.nmpdr.org/seedviewer.cgi?page=FunctionalRole&role=Cation%20efflux%20system%20protein%20CusA&subsystem_name=Cobalt-zinc-cadmium_resistance) |
| Virulence, Disease and Defense | Resistance to antibiotics and toxic compounds | [Adaptation to d-cysteine](https://rast.nmpdr.org/seedviewer.cgi?page=Subsystems&subsystem=Adaptation_to_d-cysteine&organism=549.408) | [Cystine ABC transporter, permease protein](https://rast.nmpdr.org/seedviewer.cgi?page=FunctionalRole&role=Cystine%20ABC%20transporter,%20permease%20protein&subsystem_name=Adaptation_to_d-cysteine) |
| Virulence, Disease and Defense | Resistance to antibiotics and toxic compounds | [Adaptation to d-cysteine](https://rast.nmpdr.org/seedviewer.cgi?page=Subsystems&subsystem=Adaptation_to_d-cysteine&organism=549.408) | [Cystine ABC transporter, ATP-binding protein](https://rast.nmpdr.org/seedviewer.cgi?page=FunctionalRole&role=Cystine%20ABC%20transporter,%20ATP-binding%20protein&subsystem_name=Adaptation_to_d-cysteine) |
| Virulence, Disease and Defense | Resistance to antibiotics and toxic compounds | [Adaptation to d-cysteine](https://rast.nmpdr.org/seedviewer.cgi?page=Subsystems&subsystem=Adaptation_to_d-cysteine&organism=549.408) | [D-cysteine desulfhydrase (EC 4.4.1.15)](https://rast.nmpdr.org/seedviewer.cgi?page=FunctionalRole&role=D-cysteine%20desulfhydrase%20(EC%204.4.1.15)&subsystem_name=Adaptation_to_d-cysteine) |
| Virulence, Disease and Defense | Resistance to antibiotics and toxic compounds | [Multidrug Resistance, Tripartite Systems Found in Gram Negative Bacteria](https://rast.nmpdr.org/seedviewer.cgi?page=Subsystems&subsystem=Multidrug_Resistance,_Tripartite_Systems_Found_in_Gram_Negative_Bacteria&organism=549.408) | [Outer membrane component of tripartite multidrug resistance system](https://rast.nmpdr.org/seedviewer.cgi?page=FunctionalRole&role=Outer%20membrane%20component%20of%20tripartite%20multidrug%20resistance%20system&subsystem_name=Multidrug_Resistance,_Tripartite_Systems_Found_in_Gram_Negative_Bacteria) |
| Virulence, Disease and Defense | Resistance to antibiotics and toxic compounds | [Multidrug Resistance, Tripartite Systems Found in Gram Negative Bacteria](https://rast.nmpdr.org/seedviewer.cgi?page=Subsystems&subsystem=Multidrug_Resistance,_Tripartite_Systems_Found_in_Gram_Negative_Bacteria&organism=549.408) | [Membrane fusion component of tripartite multidrug resistance system](https://rast.nmpdr.org/seedviewer.cgi?page=FunctionalRole&role=Membrane%20fusion%20component%20of%20tripartite%20multidrug%20resistance%20system&subsystem_name=Multidrug_Resistance,_Tripartite_Systems_Found_in_Gram_Negative_Bacteria) |
| Virulence, Disease and Defense | Resistance to antibiotics and toxic compounds | [Multidrug Resistance, Tripartite Systems Found in Gram Negative Bacteria](https://rast.nmpdr.org/seedviewer.cgi?page=Subsystems&subsystem=Multidrug_Resistance,_Tripartite_Systems_Found_in_Gram_Negative_Bacteria&organism=549.408) | [Inner membrane component of tripartite multidrug resistance system](https://rast.nmpdr.org/seedviewer.cgi?page=FunctionalRole&role=Inner%20membrane%20component%20of%20tripartite%20multidrug%20resistance%20system&subsystem_name=Multidrug_Resistance,_Tripartite_Systems_Found_in_Gram_Negative_Bacteria) |
| Virulence, Disease and Defense | Resistance to antibiotics and toxic compounds | [Zinc resistance](https://rast.nmpdr.org/seedviewer.cgi?page=Subsystems&subsystem=Zinc_resistance&organism=549.408) | [Response regulator of zinc sigma-54-dependent two-component system](https://rast.nmpdr.org/seedviewer.cgi?page=FunctionalRole&role=Response%20regulator%20of%20zinc%20sigma-54-dependent%20two-component%20system&subsystem_name=Zinc_resistance) |
| Virulence, Disease and Defense | Resistance to antibiotics and toxic compounds | [Resistance to fluoroquinolones](https://rast.nmpdr.org/seedviewer.cgi?page=Subsystems&subsystem=Resistance_to_fluoroquinolones&organism=549.408) | [DNA gyrase subunit B (EC 5.99.1.3)](https://rast.nmpdr.org/seedviewer.cgi?page=FunctionalRole&role=DNA%20gyrase%20subunit%20B%20(EC%205.99.1.3)&subsystem_name=Resistance_to_fluoroquinolones) |
| Virulence, Disease and Defense | Resistance to antibiotics and toxic compounds | [Resistance to fluoroquinolones](https://rast.nmpdr.org/seedviewer.cgi?page=Subsystems&subsystem=Resistance_to_fluoroquinolones&organism=549.408) | [DNA gyrase subunit A (EC 5.99.1.3)](https://rast.nmpdr.org/seedviewer.cgi?page=FunctionalRole&role=DNA%20gyrase%20subunit%20A%20(EC%205.99.1.3)&subsystem_name=Resistance_to_fluoroquinolones) |
| Virulence, Disease and Defense | Resistance to antibiotics and toxic compounds | [Resistance to fluoroquinolones](https://rast.nmpdr.org/seedviewer.cgi?page=Subsystems&subsystem=Resistance_to_fluoroquinolones&organism=549.408) | [Topoisomerase IV subunit B (EC 5.99.1.-)](https://rast.nmpdr.org/seedviewer.cgi?page=FunctionalRole&role=Topoisomerase%20IV%20subunit%20B%20(EC%205.99.1.-)&subsystem_name=Resistance_to_fluoroquinolones) |
| Virulence, Disease and Defense | Resistance to antibiotics and toxic compounds | [Resistance to fluoroquinolones](https://rast.nmpdr.org/seedviewer.cgi?page=Subsystems&subsystem=Resistance_to_fluoroquinolones&organism=549.408) | [Topoisomerase IV subunit A (EC 5.99.1.-)](https://rast.nmpdr.org/seedviewer.cgi?page=FunctionalRole&role=Topoisomerase%20IV%20subunit%20A%20(EC%205.99.1.-)&subsystem_name=Resistance_to_fluoroquinolones) |
| Virulence, Disease and Defense | Resistance to antibiotics and toxic compounds | [Arsenic resistance](https://rast.nmpdr.org/seedviewer.cgi?page=Subsystems&subsystem=Arsenic_resistance&organism=549.408) | [Arsenic resistance protein ArsH](https://rast.nmpdr.org/seedviewer.cgi?page=FunctionalRole&role=Arsenic%20resistance%20protein%20ArsH&subsystem_name=Arsenic_resistance) |
| Virulence, Disease and Defense | Resistance to antibiotics and toxic compounds | [Arsenic resistance](https://rast.nmpdr.org/seedviewer.cgi?page=Subsystems&subsystem=Arsenic_resistance&organism=549.408) | [Arsenic efflux pump protein](https://rast.nmpdr.org/seedviewer.cgi?page=FunctionalRole&role=Arsenic%20efflux%20pump%20protein&subsystem_name=Arsenic_resistance) |
| Virulence, Disease and Defense | Resistance to antibiotics and toxic compounds | [Arsenic resistance](https://rast.nmpdr.org/seedviewer.cgi?page=Subsystems&subsystem=Arsenic_resistance&organism=549.408) | [Arsenate reductase (EC 1.20.4.1)](https://rast.nmpdr.org/seedviewer.cgi?page=FunctionalRole&role=Arsenate%20reductase%20(EC%201.20.4.1)&subsystem_name=Arsenic_resistance) |
| Virulence, Disease and Defense | Resistance to antibiotics and toxic compounds | [Copper homeostasis: copper tolerance](https://rast.nmpdr.org/seedviewer.cgi?page=Subsystems&subsystem=Copper_homeostasis:_copper_tolerance&organism=549.408) | [Copper homeostasis protein CutE](https://rast.nmpdr.org/seedviewer.cgi?page=FunctionalRole&role=Copper%20homeostasis%20protein%20CutE&subsystem_name=Copper_homeostasis:_copper_tolerance) |
| Virulence, Disease and Defense | Resistance to antibiotics and toxic compounds | [Copper homeostasis: copper tolerance](https://rast.nmpdr.org/seedviewer.cgi?page=Subsystems&subsystem=Copper_homeostasis:_copper_tolerance&organism=549.408) | [Magnesium and cobalt efflux protein CorC](https://rast.nmpdr.org/seedviewer.cgi?page=FunctionalRole&role=Magnesium%20and%20cobalt%20efflux%20protein%20CorC&subsystem_name=Copper_homeostasis:_copper_tolerance) |
| Virulence, Disease and Defense | Resistance to antibiotics and toxic compounds | [Fosfomycin resistance](https://rast.nmpdr.org/seedviewer.cgi?page=Subsystems&subsystem=Fosfomycin_resistance&organism=549.408) | [Fosfomycin resistance protein FosA](https://rast.nmpdr.org/seedviewer.cgi?page=FunctionalRole&role=Fosfomycin%20resistance%20protein%20FosA&subsystem_name=Fosfomycin_resistance) |
| Virulence, Disease and Defense | Resistance to antibiotics and toxic compounds | [Beta-lactamase](https://rast.nmpdr.org/seedviewer.cgi?page=Subsystems&subsystem=Beta-lactamase&organism=549.408) | [Beta-lactamase (EC 3.5.2.6)](https://rast.nmpdr.org/seedviewer.cgi?page=FunctionalRole&role=Beta-lactamase%20(EC%203.5.2.6)&subsystem_name=Beta-lactamase) |
| Virulence, Disease and Defense | Resistance to antibiotics and toxic compounds | [Beta-lactamase](https://rast.nmpdr.org/seedviewer.cgi?page=Subsystems&subsystem=Beta-lactamase&organism=549.408) | [Metal-dependent hydrolases of the beta-lactamase superfamily I](https://rast.nmpdr.org/seedviewer.cgi?page=FunctionalRole&role=Metal-dependent%20hydrolases%20of%20the%20beta-lactamase%20superfamily%20I&subsystem_name=Beta-lactamase) |
| Virulence, Disease and Defense | Resistance to antibiotics and toxic compounds | [Multidrug Resistance Efflux Pumps](https://rast.nmpdr.org/seedviewer.cgi?page=Subsystems&subsystem=Multidrug_Resistance_Efflux_Pumps&organism=549.408) | [RND efflux system, outer membrane lipoprotein, NodT family](https://rast.nmpdr.org/seedviewer.cgi?page=FunctionalRole&role=RND%20efflux%20system,%20outer%20membrane%20lipoprotein,%20NodT%20family&subsystem_name=Multidrug_Resistance_Efflux_Pumps) |
| Virulence, Disease and Defense | Resistance to antibiotics and toxic compounds | [Multidrug Resistance Efflux Pumps](https://rast.nmpdr.org/seedviewer.cgi?page=Subsystems&subsystem=Multidrug_Resistance_Efflux_Pumps&organism=549.408) | [RND efflux system, membrane fusion protein CmeA](https://rast.nmpdr.org/seedviewer.cgi?page=FunctionalRole&role=RND%20efflux%20system,%20membrane%20fusion%20protein%20CmeA&subsystem_name=Multidrug_Resistance_Efflux_Pumps) |
| Virulence, Disease and Defense | Resistance to antibiotics and toxic compounds | [Multidrug Resistance Efflux Pumps](https://rast.nmpdr.org/seedviewer.cgi?page=Subsystems&subsystem=Multidrug_Resistance_Efflux_Pumps&organism=549.408) | [Multi antimicrobial extrusion protein (Na(+)/drug antiporter), MATE family of MDR efflux pumps](https://rast.nmpdr.org/seedviewer.cgi?page=FunctionalRole&role=Multi%20antimicrobial%20extrusion%20protein%20(Na(+)/drug%20antiporter),%20MATE%20family%20of%20MDR%20efflux%20pumps&subsystem_name=Multidrug_Resistance_Efflux_Pumps) |
| Virulence, Disease and Defense | Resistance to antibiotics and toxic compounds | [Multidrug Resistance Efflux Pumps](https://rast.nmpdr.org/seedviewer.cgi?page=Subsystems&subsystem=Multidrug_Resistance_Efflux_Pumps&organism=549.408) | [Transcription repressor of multidrug efflux pump acrAB operon, TetR (AcrR) family](https://rast.nmpdr.org/seedviewer.cgi?page=FunctionalRole&role=Transcription%20repressor%20of%20multidrug%20efflux%20pump%20acrAB%20operon,%20TetR%20(AcrR)%20family&subsystem_name=Multidrug_Resistance_Efflux_Pumps) |
| Virulence, Disease and Defense | Resistance to antibiotics and toxic compounds | [Multidrug Resistance Efflux Pumps](https://rast.nmpdr.org/seedviewer.cgi?page=Subsystems&subsystem=Multidrug_Resistance_Efflux_Pumps&organism=549.408) | [RND efflux system, inner membrane transporter CmeB](https://rast.nmpdr.org/seedviewer.cgi?page=FunctionalRole&role=RND%20efflux%20system,%20inner%20membrane%20transporter%20CmeB&subsystem_name=Multidrug_Resistance_Efflux_Pumps) |
| Virulence, Disease and Defense | Resistance to antibiotics and toxic compounds | [Multidrug Resistance Efflux Pumps](https://rast.nmpdr.org/seedviewer.cgi?page=Subsystems&subsystem=Multidrug_Resistance_Efflux_Pumps&organism=549.408) | [Multidrug-efflux transporter, major facilitator superfamily (MFS) (TC 2.A.1)](https://rast.nmpdr.org/seedviewer.cgi?page=FunctionalRole&role=Multidrug-efflux%20transporter,%20major%20facilitator%20superfamily%20(MFS)%20(TC%202.A.1)&subsystem_name=Multidrug_Resistance_Efflux_Pumps) |
| Virulence, Disease and Defense | Resistance to antibiotics and toxic compounds | [Multidrug Resistance Efflux Pumps](https://rast.nmpdr.org/seedviewer.cgi?page=Subsystems&subsystem=Multidrug_Resistance_Efflux_Pumps&organism=549.408) | [Probable transcription regulator protein of MDR efflux pump cluster](https://rast.nmpdr.org/seedviewer.cgi?page=FunctionalRole&role=Probable%20transcription%20regulator%20protein%20of%20MDR%20efflux%20pump%20cluster&subsystem_name=Multidrug_Resistance_Efflux_Pumps) |
| Virulence, Disease and Defense | Resistance to antibiotics and toxic compounds | [Multidrug Resistance Efflux Pumps](https://rast.nmpdr.org/seedviewer.cgi?page=Subsystems&subsystem=Multidrug_Resistance_Efflux_Pumps&organism=549.408) | [Acriflavin resistance protein](https://rast.nmpdr.org/seedviewer.cgi?page=FunctionalRole&role=Acriflavin%20resistance%20protein&subsystem_name=Multidrug_Resistance_Efflux_Pumps) |
| Virulence, Disease and Defense | Resistance to antibiotics and toxic compounds | [Multidrug Resistance Efflux Pumps](https://rast.nmpdr.org/seedviewer.cgi?page=Subsystems&subsystem=Multidrug_Resistance_Efflux_Pumps&organism=549.408) | [Membrane fusion protein of RND family multidrug efflux pump](https://rast.nmpdr.org/seedviewer.cgi?page=FunctionalRole&role=Membrane%20fusion%20protein%20of%20RND%20family%20multidrug%20efflux%20pump&subsystem_name=Multidrug_Resistance_Efflux_Pumps) |
| Virulence, Disease and Defense | Resistance to antibiotics and toxic compounds | [Multidrug Resistance Efflux Pumps](https://rast.nmpdr.org/seedviewer.cgi?page=Subsystems&subsystem=Multidrug_Resistance_Efflux_Pumps&organism=549.408) | [Type I secretion outer membrane protein, TolC precursor](https://rast.nmpdr.org/seedviewer.cgi?page=FunctionalRole&role=Type%20I%20secretion%20outer%20membrane%20protein,%20TolC%20precursor&subsystem_name=Multidrug_Resistance_Efflux_Pumps) |
| Virulence, Disease and Defense | Invasion and intracellular resistance | [Mycobacterium virulence operon involved in protein synthesis (SSU ribosomal proteins)](https://rast.nmpdr.org/seedviewer.cgi?page=Subsystems&subsystem=Mycobacterium_virulence_operon_involved_in_protein_synthesis_(SSU_ribosomal_proteins)&organism=549.408) | [SSU ribosomal protein S7p (S5e)](https://rast.nmpdr.org/seedviewer.cgi?page=FunctionalRole&role=SSU%20ribosomal%20protein%20S7p%20(S5e)&subsystem_name=Mycobacterium_virulence_operon_involved_in_protein_synthesis_(SSU_ribosomal_proteins)) |
| Virulence, Disease and Defense | Invasion and intracellular resistance | [Mycobacterium virulence operon involved in protein synthesis (SSU ribosomal proteins)](https://rast.nmpdr.org/seedviewer.cgi?page=Subsystems&subsystem=Mycobacterium_virulence_operon_involved_in_protein_synthesis_(SSU_ribosomal_proteins)&organism=549.408) | [Translation elongation factor G](https://rast.nmpdr.org/seedviewer.cgi?page=FunctionalRole&role=Translation%20elongation%20factor%20G&subsystem_name=Mycobacterium_virulence_operon_involved_in_protein_synthesis_(SSU_ribosomal_proteins)) |
| Virulence, Disease and Defense | Invasion and intracellular resistance | [Mycobacterium virulence operon involved in protein synthesis (SSU ribosomal proteins)](https://rast.nmpdr.org/seedviewer.cgi?page=Subsystems&subsystem=Mycobacterium_virulence_operon_involved_in_protein_synthesis_(SSU_ribosomal_proteins)&organism=549.408) | [Translation elongation factor Tu](https://rast.nmpdr.org/seedviewer.cgi?page=FunctionalRole&role=Translation%20elongation%20factor%20Tu&subsystem_name=Mycobacterium_virulence_operon_involved_in_protein_synthesis_(SSU_ribosomal_proteins)) |
| Virulence, Disease and Defense | Invasion and intracellular resistance | [Mycobacterium virulence operon involved in protein synthesis (SSU ribosomal proteins)](https://rast.nmpdr.org/seedviewer.cgi?page=Subsystems&subsystem=Mycobacterium_virulence_operon_involved_in_protein_synthesis_(SSU_ribosomal_proteins)&organism=549.408) | [SSU ribosomal protein S12p (S23e)](https://rast.nmpdr.org/seedviewer.cgi?page=FunctionalRole&role=SSU%20ribosomal%20protein%20S12p%20(S23e)&subsystem_name=Mycobacterium_virulence_operon_involved_in_protein_synthesis_(SSU_ribosomal_proteins)) |
| Virulence, Disease and Defense | Invasion and intracellular resistance | [Mycobacterium virulence operon involved in DNA transcription](https://rast.nmpdr.org/seedviewer.cgi?page=Subsystems&subsystem=Mycobacterium_virulence_operon_involved_in_DNA_transcription&organism=549.408) | [DNA-directed RNA polymerase beta' subunit (EC 2.7.7.6)](https://rast.nmpdr.org/seedviewer.cgi?page=FunctionalRole&role=DNA-directed%20RNA%20polymerase%20beta) |
| Virulence, Disease and Defense | Invasion and intracellular resistance | [Mycobacterium virulence operon involved in DNA transcription](https://rast.nmpdr.org/seedviewer.cgi?page=Subsystems&subsystem=Mycobacterium_virulence_operon_involved_in_DNA_transcription&organism=549.408) | [DNA-directed RNA polymerase beta subunit (EC 2.7.7.6)](https://rast.nmpdr.org/seedviewer.cgi?page=FunctionalRole&role=DNA-directed%20RNA%20polymerase%20beta%20subunit%20(EC%202.7.7.6)&subsystem_name=Mycobacterium_virulence_operon_involved_in_DNA_transcription) |
| Virulence, Disease and Defense | Invasion and intracellular resistance | [Mycobacterium virulence operon possibly involved in quinolinate biosynthesis](https://rast.nmpdr.org/seedviewer.cgi?page=Subsystems&subsystem=Mycobacterium_virulence_operon_possibly_involved_in_quinolinate_biosynthesis&organism=549.408) | [Quinolinate synthetase (EC 2.5.1.72)](https://rast.nmpdr.org/seedviewer.cgi?page=FunctionalRole&role=Quinolinate%20synthetase%20(EC%202.5.1.72)&subsystem_name=Mycobacterium_virulence_operon_possibly_involved_in_quinolinate_biosynthesis) |
| Virulence, Disease and Defense | Invasion and intracellular resistance | [Mycobacterium virulence operon possibly involved in quinolinate biosynthesis](https://rast.nmpdr.org/seedviewer.cgi?page=Subsystems&subsystem=Mycobacterium_virulence_operon_possibly_involved_in_quinolinate_biosynthesis&organism=549.408) | [Quinolinate phosphoribosyltransferase [decarboxylating] (EC 2.4.2.19)](https://rast.nmpdr.org/seedviewer.cgi?page=FunctionalRole&role=Quinolinate%20phosphoribosyltransferase%20%5bdecarboxylating%5d%20(EC%202.4.2.19)&subsystem_name=Mycobacterium_virulence_operon_possibly_involved_in_quinolinate_biosynthesis) |
| Virulence, Disease and Defense | Invasion and intracellular resistance | [Mycobacterium virulence operon possibly involved in quinolinate biosynthesis](https://rast.nmpdr.org/seedviewer.cgi?page=Subsystems&subsystem=Mycobacterium_virulence_operon_possibly_involved_in_quinolinate_biosynthesis&organism=549.408) | [L-aspartate oxidase (EC 1.4.3.16)](https://rast.nmpdr.org/seedviewer.cgi?page=FunctionalRole&role=L-aspartate%20oxidase%20(EC%201.4.3.16)&subsystem_name=Mycobacterium_virulence_operon_possibly_involved_in_quinolinate_biosynthesis) |
| Virulence, Disease and Defense | Invasion and intracellular resistance | [Mycobacterium virulence operon involved in protein synthesis (LSU ribosomal proteins)](https://rast.nmpdr.org/seedviewer.cgi?page=Subsystems&subsystem=Mycobacterium_virulence_operon_involved_in_protein_synthesis_(LSU_ribosomal_proteins)&organism=549.408) | [LSU ribosomal protein L35p](https://rast.nmpdr.org/seedviewer.cgi?page=FunctionalRole&role=LSU%20ribosomal%20protein%20L35p&subsystem_name=Mycobacterium_virulence_operon_involved_in_protein_synthesis_(LSU_ribosomal_proteins)) |
| Virulence, Disease and Defense | Invasion and intracellular resistance | [Mycobacterium virulence operon involved in protein synthesis (LSU ribosomal proteins)](https://rast.nmpdr.org/seedviewer.cgi?page=Subsystems&subsystem=Mycobacterium_virulence_operon_involved_in_protein_synthesis_(LSU_ribosomal_proteins)&organism=549.408) | [Translation initiation factor 3](https://rast.nmpdr.org/seedviewer.cgi?page=FunctionalRole&role=Translation%20initiation%20factor%203&subsystem_name=Mycobacterium_virulence_operon_involved_in_protein_synthesis_(LSU_ribosomal_proteins)) |
| Virulence, Disease and Defense | Invasion and intracellular resistance | [Mycobacterium virulence operon involved in protein synthesis (LSU ribosomal proteins)](https://rast.nmpdr.org/seedviewer.cgi?page=Subsystems&subsystem=Mycobacterium_virulence_operon_involved_in_protein_synthesis_(LSU_ribosomal_proteins)&organism=549.408) | [LSU ribosomal protein L20p](https://rast.nmpdr.org/seedviewer.cgi?page=FunctionalRole&role=LSU%20ribosomal%20protein%20L20p&subsystem_name=Mycobacterium_virulence_operon_involved_in_protein_synthesis_(LSU_ribosomal_proteins)) |
| Potassium metabolism | Potassium metabolism - no subcategory | [Potassium homeostasis](https://rast.nmpdr.org/seedviewer.cgi?page=Subsystems&subsystem=Potassium_homeostasis&organism=549.408) | [Potassium-transporting ATPase A chain (EC 3.6.3.12) (TC 3.A.3.7.1)](https://rast.nmpdr.org/seedviewer.cgi?page=FunctionalRole&role=Potassium-transporting%20ATPase%20A%20chain%20(EC%203.6.3.12)%20(TC%203.A.3.7.1)&subsystem_name=Potassium_homeostasis) |
| Potassium metabolism | Potassium metabolism - no subcategory | [Potassium homeostasis](https://rast.nmpdr.org/seedviewer.cgi?page=Subsystems&subsystem=Potassium_homeostasis&organism=549.408) | [FKBP-type peptidyl-prolyl cis-trans isomerase SlyD (EC 5.2.1.8)](https://rast.nmpdr.org/seedviewer.cgi?page=FunctionalRole&role=FKBP-type%20peptidyl-prolyl%20cis-trans%20isomerase%20SlyD%20(EC%205.2.1.8)&subsystem_name=Potassium_homeostasis) |
| Potassium metabolism | Potassium metabolism - no subcategory | [Potassium homeostasis](https://rast.nmpdr.org/seedviewer.cgi?page=Subsystems&subsystem=Potassium_homeostasis&organism=549.408) | [Trk system potassium uptake protein TrkA](https://rast.nmpdr.org/seedviewer.cgi?page=FunctionalRole&role=Trk%20system%20potassium%20uptake%20protein%20TrkA&subsystem_name=Potassium_homeostasis) |
| Potassium metabolism | Potassium metabolism - no subcategory | [Potassium homeostasis](https://rast.nmpdr.org/seedviewer.cgi?page=Subsystems&subsystem=Potassium_homeostasis&organism=549.408) | [Potassium uptake protein TrkH](https://rast.nmpdr.org/seedviewer.cgi?page=FunctionalRole&role=Potassium%20uptake%20protein%20TrkH&subsystem_name=Potassium_homeostasis) |
| Potassium metabolism | Potassium metabolism - no subcategory | [Potassium homeostasis](https://rast.nmpdr.org/seedviewer.cgi?page=Subsystems&subsystem=Potassium_homeostasis&organism=549.408) | [Potassium-transporting ATPase C chain (EC 3.6.3.12) (TC 3.A.3.7.1)](https://rast.nmpdr.org/seedviewer.cgi?page=FunctionalRole&role=Potassium-transporting%20ATPase%20C%20chain%20(EC%203.6.3.12)%20(TC%203.A.3.7.1)&subsystem_name=Potassium_homeostasis) |
| Potassium metabolism | Potassium metabolism - no subcategory | [Potassium homeostasis](https://rast.nmpdr.org/seedviewer.cgi?page=Subsystems&subsystem=Potassium_homeostasis&organism=549.408) | [Putative cytoplasmic protein ,probably associated with Glutathione-regulated potassium-efflux](https://rast.nmpdr.org/seedviewer.cgi?page=FunctionalRole&role=Putative%20cytoplasmic%20protein%20,probably%20associated%20with%20Glutathione-regulated%20potassium-efflux&subsystem_name=Potassium_homeostasis) |
| Potassium metabolism | Potassium metabolism - no subcategory | [Potassium homeostasis](https://rast.nmpdr.org/seedviewer.cgi?page=Subsystems&subsystem=Potassium_homeostasis&organism=549.408) | [Osmosensitive K+ channel histidine kinase KdpD (EC 2.7.3.-)](https://rast.nmpdr.org/seedviewer.cgi?page=FunctionalRole&role=Osmosensitive%20K+%20channel%20histidine%20kinase%20KdpD%20(EC%202.7.3.-)&subsystem_name=Potassium_homeostasis) |
| Potassium metabolism | Potassium metabolism - no subcategory | [Potassium homeostasis](https://rast.nmpdr.org/seedviewer.cgi?page=Subsystems&subsystem=Potassium_homeostasis&organism=549.408) | [POTASSIUM/PROTON ANTIPORTER ROSB](https://rast.nmpdr.org/seedviewer.cgi?page=FunctionalRole&role=POTASSIUM/PROTON%20ANTIPORTER%20ROSB&subsystem_name=Potassium_homeostasis) |
| Potassium metabolism | Potassium metabolism - no subcategory | [Potassium homeostasis](https://rast.nmpdr.org/seedviewer.cgi?page=Subsystems&subsystem=Potassium_homeostasis&organism=549.408) | [Potassium-transporting ATPase B chain (EC 3.6.3.12) (TC 3.A.3.7.1)](https://rast.nmpdr.org/seedviewer.cgi?page=FunctionalRole&role=Potassium-transporting%20ATPase%20B%20chain%20(EC%203.6.3.12)%20(TC%203.A.3.7.1)&subsystem_name=Potassium_homeostasis) |
| Potassium metabolism | Potassium metabolism - no subcategory | [Potassium homeostasis](https://rast.nmpdr.org/seedviewer.cgi?page=Subsystems&subsystem=Potassium_homeostasis&organism=549.408) | [Potassium efflux system KefA protein](https://rast.nmpdr.org/seedviewer.cgi?page=FunctionalRole&role=Potassium%20efflux%20system%20KefA%20protein&subsystem_name=Potassium_homeostasis) |
| Potassium metabolism | Potassium metabolism - no subcategory | [Potassium homeostasis](https://rast.nmpdr.org/seedviewer.cgi?page=Subsystems&subsystem=Potassium_homeostasis&organism=549.408) | [Glutathione-regulated potassium-efflux system ATP-binding protein](https://rast.nmpdr.org/seedviewer.cgi?page=FunctionalRole&role=Glutathione-regulated%20potassium-efflux%20system%20ATP-binding%20protein&subsystem_name=Potassium_homeostasis) |
| Potassium metabolism | Potassium metabolism - no subcategory | [Potassium homeostasis](https://rast.nmpdr.org/seedviewer.cgi?page=Subsystems&subsystem=Potassium_homeostasis&organism=549.408) | [Large-conductance mechanosensitive channel](https://rast.nmpdr.org/seedviewer.cgi?page=FunctionalRole&role=Large-conductance%20mechanosensitive%20channel&subsystem_name=Potassium_homeostasis) |
| Potassium metabolism | Potassium metabolism - no subcategory | [Potassium homeostasis](https://rast.nmpdr.org/seedviewer.cgi?page=Subsystems&subsystem=Potassium_homeostasis&organism=549.408) | [FKBP-type peptidyl-prolyl cis-trans isomerase FkpA precursor (EC 5.2.1.8)](https://rast.nmpdr.org/seedviewer.cgi?page=FunctionalRole&role=FKBP-type%20peptidyl-prolyl%20cis-trans%20isomerase%20FkpA%20precursor%20(EC%205.2.1.8)&subsystem_name=Potassium_homeostasis) |
| Potassium metabolism | Potassium metabolism - no subcategory | [Potassium homeostasis](https://rast.nmpdr.org/seedviewer.cgi?page=Subsystems&subsystem=Potassium_homeostasis&organism=549.408) | [Glutathione-regulated potassium-efflux system ancillary protein KefG](https://rast.nmpdr.org/seedviewer.cgi?page=FunctionalRole&role=Glutathione-regulated%20potassium-efflux%20system%20ancillary%20protein%20KefG&subsystem_name=Potassium_homeostasis) |
| Potassium metabolism | Potassium metabolism - no subcategory | [Potassium homeostasis](https://rast.nmpdr.org/seedviewer.cgi?page=Subsystems&subsystem=Potassium_homeostasis&organism=549.408) | [Kup system potassium uptake protein](https://rast.nmpdr.org/seedviewer.cgi?page=FunctionalRole&role=Kup%20system%20potassium%20uptake%20protein&subsystem_name=Potassium_homeostasis) |
| Potassium metabolism | Potassium metabolism - no subcategory | [Hyperosmotic potassium uptake](https://rast.nmpdr.org/seedviewer.cgi?page=Subsystems&subsystem=Hyperosmotic_potassium_uptake&organism=549.408) | [Trk system potassium uptake protein TrkA](https://rast.nmpdr.org/seedviewer.cgi?page=FunctionalRole&role=Trk%20system%20potassium%20uptake%20protein%20TrkA&subsystem_name=Hyperosmotic_potassium_uptake) |
| Potassium metabolism | Potassium metabolism - no subcategory | [Hyperosmotic potassium uptake](https://rast.nmpdr.org/seedviewer.cgi?page=Subsystems&subsystem=Hyperosmotic_potassium_uptake&organism=549.408) | [Potassium uptake protein TrkH](https://rast.nmpdr.org/seedviewer.cgi?page=FunctionalRole&role=Potassium%20uptake%20protein%20TrkH&subsystem_name=Hyperosmotic_potassium_uptake) |
| Potassium metabolism | Potassium metabolism - no subcategory | [Glutathione-regulated potassium-efflux system and associated functions](https://rast.nmpdr.org/seedviewer.cgi?page=Subsystems&subsystem=Glutathione-regulated_potassium-efflux_system_and_associated_functions&organism=549.408) | [Glutathione-regulated potassium-efflux system ATP-binding protein](https://rast.nmpdr.org/seedviewer.cgi?page=FunctionalRole&role=Glutathione-regulated%20potassium-efflux%20system%20ATP-binding%20protein&subsystem_name=Glutathione-regulated_potassium-efflux_system_and_associated_functions) |
| Potassium metabolism | Potassium metabolism - no subcategory | [Glutathione-regulated potassium-efflux system and associated functions](https://rast.nmpdr.org/seedviewer.cgi?page=Subsystems&subsystem=Glutathione-regulated_potassium-efflux_system_and_associated_functions&organism=549.408) | [Glutathione-regulated potassium-efflux system protein KefB](https://rast.nmpdr.org/seedviewer.cgi?page=FunctionalRole&role=Glutathione-regulated%20potassium-efflux%20system%20protein%20KefB&subsystem_name=Glutathione-regulated_potassium-efflux_system_and_associated_functions) |
| Potassium metabolism | Potassium metabolism - no subcategory | [Glutathione-regulated potassium-efflux system and associated functions](https://rast.nmpdr.org/seedviewer.cgi?page=Subsystems&subsystem=Glutathione-regulated_potassium-efflux_system_and_associated_functions&organism=549.408) | [Glutathione-regulated potassium-efflux system ancillary protein KefG](https://rast.nmpdr.org/seedviewer.cgi?page=FunctionalRole&role=Glutathione-regulated%20potassium-efflux%20system%20ancillary%20protein%20KefG&subsystem_name=Glutathione-regulated_potassium-efflux_system_and_associated_functions) |
| Miscellaneous | Plant-Prokaryote DOE project | [YrdC-YciO-Sua5 protein family](https://rast.nmpdr.org/seedviewer.cgi?page=Subsystems&subsystem=YrdC-YciO-Sua5_protein_family&organism=549.408) | [Protein-N(5)-glutamine methyltransferase PrmC, methylates polypeptide chain release factors RF1 and RF2](https://rast.nmpdr.org/seedviewer.cgi?page=FunctionalRole&role=Protein-N(5)-glutamine%20methyltransferase%20PrmC,%20methylates%20polypeptide%20chain%20release%20factors%20RF1%20and%20RF2&subsystem_name=YrdC-YciO-Sua5_protein_family) |
| Miscellaneous | Plant-Prokaryote DOE project | [YrdC-YciO-Sua5 protein family](https://rast.nmpdr.org/seedviewer.cgi?page=Subsystems&subsystem=YrdC-YciO-Sua5_protein_family&organism=549.408) | [Hypothetical YciO protein, TsaC/YrdC paralog](https://rast.nmpdr.org/seedviewer.cgi?page=FunctionalRole&role=Hypothetical%20YciO%20protein,%20TsaC/YrdC%20paralog&subsystem_name=YrdC-YciO-Sua5_protein_family) |
| Miscellaneous | Plant-Prokaryote DOE project | [YrdC-YciO-Sua5 protein family](https://rast.nmpdr.org/seedviewer.cgi?page=Subsystems&subsystem=YrdC-YciO-Sua5_protein_family&organism=549.408) | [TsaB protein, required for threonylcarbamoyladenosine (t(6)A) formation in tRNA](https://rast.nmpdr.org/seedviewer.cgi?page=FunctionalRole&role=TsaB%20protein,%20required%20for%20threonylcarbamoyladenosine%20(t(6)A)%20formation%20in%20tRNA&subsystem_name=YrdC-YciO-Sua5_protein_family) |
| Miscellaneous | Plant-Prokaryote DOE project | [YrdC-YciO-Sua5 protein family](https://rast.nmpdr.org/seedviewer.cgi?page=Subsystems&subsystem=YrdC-YciO-Sua5_protein_family&organism=549.408) | [Protein-N(5)-glutamine methyltransferase PrmB, methylates LSU ribosomal protein L3p](https://rast.nmpdr.org/seedviewer.cgi?page=FunctionalRole&role=Protein-N(5)-glutamine%20methyltransferase%20PrmB,%20methylates%20LSU%20ribosomal%20protein%20L3p&subsystem_name=YrdC-YciO-Sua5_protein_family) |
| Miscellaneous | Plant-Prokaryote DOE project | [YrdC-YciO-Sua5 protein family](https://rast.nmpdr.org/seedviewer.cgi?page=Subsystems&subsystem=YrdC-YciO-Sua5_protein_family&organism=549.408) | [Serine hydroxymethyltransferase (EC 2.1.2.1)](https://rast.nmpdr.org/seedviewer.cgi?page=FunctionalRole&role=Serine%20hydroxymethyltransferase%20(EC%202.1.2.1)&subsystem_name=YrdC-YciO-Sua5_protein_family) |
| Miscellaneous | Plant-Prokaryote DOE project | [YrdC-YciO-Sua5 protein family](https://rast.nmpdr.org/seedviewer.cgi?page=Subsystems&subsystem=YrdC-YciO-Sua5_protein_family&organism=549.408) | [Rossmann fold nucleotide-binding protein Smf possibly involved in DNA uptake](https://rast.nmpdr.org/seedviewer.cgi?page=FunctionalRole&role=Rossmann%20fold%20nucleotide-binding%20protein%20Smf%20possibly%20involved%20in%20DNA%20uptake&subsystem_name=YrdC-YciO-Sua5_protein_family) |
| Miscellaneous | Plant-Prokaryote DOE project | [YrdC-YciO-Sua5 protein family](https://rast.nmpdr.org/seedviewer.cgi?page=Subsystems&subsystem=YrdC-YciO-Sua5_protein_family&organism=549.408) | [Peptide chain release factor 1](https://rast.nmpdr.org/seedviewer.cgi?page=FunctionalRole&role=Peptide%20chain%20release%20factor%201&subsystem_name=YrdC-YciO-Sua5_protein_family) |
| Miscellaneous | Plant-Prokaryote DOE project | [YrdC-YciO-Sua5 protein family](https://rast.nmpdr.org/seedviewer.cgi?page=Subsystems&subsystem=YrdC-YciO-Sua5_protein_family&organism=549.408) | [TsaD/Kae1/Qri7 protein, required for threonylcarbamoyladenosine t(6)A37 formation in tRNA](https://rast.nmpdr.org/seedviewer.cgi?page=FunctionalRole&role=TsaD/Kae1/Qri7%20protein,%20required%20for%20threonylcarbamoyladenosine%20t(6)A37%20formation%20in%20tRNA&subsystem_name=YrdC-YciO-Sua5_protein_family) |
| Miscellaneous | Plant-Prokaryote DOE project | [YrdC-YciO-Sua5 protein family](https://rast.nmpdr.org/seedviewer.cgi?page=Subsystems&subsystem=YrdC-YciO-Sua5_protein_family&organism=549.408) | [Ribosomal-protein-S18p-alanine acetyltransferase (EC 2.3.1.-)](https://rast.nmpdr.org/seedviewer.cgi?page=FunctionalRole&role=Ribosomal-protein-S18p-alanine%20acetyltransferase%20(EC%202.3.1.-)&subsystem_name=YrdC-YciO-Sua5_protein_family) |
| Miscellaneous | Plant-Prokaryote DOE project | [YrdC-YciO-Sua5 protein family](https://rast.nmpdr.org/seedviewer.cgi?page=Subsystems&subsystem=YrdC-YciO-Sua5_protein_family&organism=549.408) | [COG0613, Predicted metal-dependent phosphoesterases (PHP family)](https://rast.nmpdr.org/seedviewer.cgi?page=FunctionalRole&role=COG0613,%20Predicted%20metal-dependent%20phosphoesterases%20(PHP%20family)&subsystem_name=YrdC-YciO-Sua5_protein_family) |
| Miscellaneous | Plant-Prokaryote DOE project | [YrdC-YciO-Sua5 protein family](https://rast.nmpdr.org/seedviewer.cgi?page=Subsystems&subsystem=YrdC-YciO-Sua5_protein_family&organism=549.408) | [TsaE protein, required for threonylcarbamoyladenosine t(6)A37 formation in tRNA](https://rast.nmpdr.org/seedviewer.cgi?page=FunctionalRole&role=TsaE%20protein,%20required%20for%20threonylcarbamoyladenosine%20t(6)A37%20formation%20in%20tRNA&subsystem_name=YrdC-YciO-Sua5_protein_family) |
| Miscellaneous | Plant-Prokaryote DOE project | [Scaffold proteins for [4Fe-4S] cluster assembly (MRP family)](https://rast.nmpdr.org/seedviewer.cgi?page=Subsystems&subsystem=Scaffold_proteins_for_%5b4Fe-4S%5d_cluster_assembly_(MRP_family)&organism=549.408) | [Methionyl-tRNA synthetase (EC 6.1.1.10)](https://rast.nmpdr.org/seedviewer.cgi?page=FunctionalRole&role=Methionyl-tRNA%20synthetase%20(EC%206.1.1.10)&subsystem_name=Scaffold_proteins_for_%5b4Fe-4S%5d_cluster_assembly_(MRP_family)) |
| Miscellaneous | Plant-Prokaryote DOE project | [Scaffold proteins for [4Fe-4S] cluster assembly (MRP family)](https://rast.nmpdr.org/seedviewer.cgi?page=Subsystems&subsystem=Scaffold_proteins_for_%5b4Fe-4S%5d_cluster_assembly_(MRP_family)&organism=549.408) | [Iron-sulfur cluster-binding protein](https://rast.nmpdr.org/seedviewer.cgi?page=FunctionalRole&role=Iron-sulfur%20cluster-binding%20protein&subsystem_name=Scaffold_proteins_for_%5b4Fe-4S%5d_cluster_assembly_(MRP_family)) |
| Miscellaneous | Plant-Prokaryote DOE project | [Scaffold proteins for [4Fe-4S] cluster assembly (MRP family)](https://rast.nmpdr.org/seedviewer.cgi?page=Subsystems&subsystem=Scaffold_proteins_for_%5b4Fe-4S%5d_cluster_assembly_(MRP_family)&organism=549.408) | [S-adenosylmethionine:tRNA ribosyltransferase-isomerase (EC 5.-.-.-)](https://rast.nmpdr.org/seedviewer.cgi?page=FunctionalRole&role=S-adenosylmethionine:tRNA%20ribosyltransferase-isomerase%20(EC%205.-.-.-)&subsystem_name=Scaffold_proteins_for_%5b4Fe-4S%5d_cluster_assembly_(MRP_family)) |
| Miscellaneous | Plant-Prokaryote DOE project | [Scaffold proteins for [4Fe-4S] cluster assembly (MRP family)](https://rast.nmpdr.org/seedviewer.cgi?page=Subsystems&subsystem=Scaffold_proteins_for_%5b4Fe-4S%5d_cluster_assembly_(MRP_family)&organism=549.408) | [HflK protein](https://rast.nmpdr.org/seedviewer.cgi?page=FunctionalRole&role=HflK%20protein&subsystem_name=Scaffold_proteins_for_%5b4Fe-4S%5d_cluster_assembly_(MRP_family)) |
| Miscellaneous | Plant-Prokaryote DOE project | [Scaffold proteins for [4Fe-4S] cluster assembly (MRP family)](https://rast.nmpdr.org/seedviewer.cgi?page=Subsystems&subsystem=Scaffold_proteins_for_%5b4Fe-4S%5d_cluster_assembly_(MRP_family)&organism=549.408) | [Scaffold protein for [4Fe-4S] cluster assembly ApbC, MRP-like](https://rast.nmpdr.org/seedviewer.cgi?page=FunctionalRole&role=Scaffold%20protein%20for%20%5b4Fe-4S%5d%20cluster%20assembly%20ApbC,%20MRP-like&subsystem_name=Scaffold_proteins_for_%5b4Fe-4S%5d_cluster_assembly_(MRP_family)) |
| Miscellaneous | Plant-Prokaryote DOE project | [Scaffold proteins for [4Fe-4S] cluster assembly (MRP family)](https://rast.nmpdr.org/seedviewer.cgi?page=Subsystems&subsystem=Scaffold_proteins_for_%5b4Fe-4S%5d_cluster_assembly_(MRP_family)&organism=549.408) | [HflC protein](https://rast.nmpdr.org/seedviewer.cgi?page=FunctionalRole&role=HflC%20protein&subsystem_name=Scaffold_proteins_for_%5b4Fe-4S%5d_cluster_assembly_(MRP_family)) |
| Miscellaneous | Plant-Prokaryote DOE project | [Scaffold proteins for [4Fe-4S] cluster assembly (MRP family)](https://rast.nmpdr.org/seedviewer.cgi?page=Subsystems&subsystem=Scaffold_proteins_for_%5b4Fe-4S%5d_cluster_assembly_(MRP_family)&organism=549.408) | [tRNA-guanine transglycosylase (EC 2.4.2.29)](https://rast.nmpdr.org/seedviewer.cgi?page=FunctionalRole&role=tRNA-guanine%20transglycosylase%20(EC%202.4.2.29)&subsystem_name=Scaffold_proteins_for_%5b4Fe-4S%5d_cluster_assembly_(MRP_family)) |
| Miscellaneous | Plant-Prokaryote DOE project | [Scaffold proteins for [4Fe-4S] cluster assembly (MRP family)](https://rast.nmpdr.org/seedviewer.cgi?page=Subsystems&subsystem=Scaffold_proteins_for_%5b4Fe-4S%5d_cluster_assembly_(MRP_family)&organism=549.408) | [TsaE protein, required for threonylcarbamoyladenosine t(6)A37 formation in tRNA](https://rast.nmpdr.org/seedviewer.cgi?page=FunctionalRole&role=TsaE%20protein,%20required%20for%20threonylcarbamoyladenosine%20t(6)A37%20formation%20in%20tRNA&subsystem_name=Scaffold_proteins_for_%5b4Fe-4S%5d_cluster_assembly_(MRP_family)) |
| Miscellaneous | Plant-Prokaryote DOE project | [Scaffold proteins for [4Fe-4S] cluster assembly (MRP family)](https://rast.nmpdr.org/seedviewer.cgi?page=Subsystems&subsystem=Scaffold_proteins_for_%5b4Fe-4S%5d_cluster_assembly_(MRP_family)&organism=549.408) | [HtrA protease/chaperone protein](https://rast.nmpdr.org/seedviewer.cgi?page=FunctionalRole&role=HtrA%20protease/chaperone%20protein&subsystem_name=Scaffold_proteins_for_%5b4Fe-4S%5d_cluster_assembly_(MRP_family)) |
| Miscellaneous | Plant-Prokaryote DOE project | [Single-Rhodanese-domain proteins](https://rast.nmpdr.org/seedviewer.cgi?page=Subsystems&subsystem=Single-Rhodanese-domain_proteins&organism=549.408) | [Thiosulfate sulfurtransferase GlpE (EC 2.8.1.1)](https://rast.nmpdr.org/seedviewer.cgi?page=FunctionalRole&role=Thiosulfate%20sulfurtransferase%20GlpE%20(EC%202.8.1.1)&subsystem_name=Single-Rhodanese-domain_proteins) |
| Miscellaneous | Plant-Prokaryote DOE project | [Single-Rhodanese-domain proteins](https://rast.nmpdr.org/seedviewer.cgi?page=Subsystems&subsystem=Single-Rhodanese-domain_proteins&organism=549.408) | [Rhodanese domain protein, Enterobacterial subgroup, YceA homolog](https://rast.nmpdr.org/seedviewer.cgi?page=FunctionalRole&role=Rhodanese%20domain%20protein,%20Enterobacterial%20subgroup,%20YceA%20homolog&subsystem_name=Single-Rhodanese-domain_proteins) |
| Miscellaneous | Miscellaneous - no subcategory | [Phosphoglycerate mutase protein family](https://rast.nmpdr.org/seedviewer.cgi?page=Subsystems&subsystem=Phosphoglycerate_mutase_protein_family&organism=549.408) | [Adenosine (5')-pentaphospho-(5'')-adenosine pyrophosphohydrolase (EC 3.6.1.-)](https://rast.nmpdr.org/seedviewer.cgi?page=FunctionalRole&role=Adenosine%20(5) |
| Miscellaneous | Miscellaneous - no subcategory | [Phosphoglycerate mutase protein family](https://rast.nmpdr.org/seedviewer.cgi?page=Subsystems&subsystem=Phosphoglycerate_mutase_protein_family&organism=549.408) | [Phosphoglycerate mutase (EC 5.4.2.1)](https://rast.nmpdr.org/seedviewer.cgi?page=FunctionalRole&role=Phosphoglycerate%20mutase%20(EC%205.4.2.1)&subsystem_name=Phosphoglycerate_mutase_protein_family) |
| Miscellaneous | Miscellaneous - no subcategory | [DedA family of inner membrane proteins](https://rast.nmpdr.org/seedviewer.cgi?page=Subsystems&subsystem=DedA_family_of_inner_membrane_proteins&organism=549.408) | [DedA family inner membrane protein YabI](https://rast.nmpdr.org/seedviewer.cgi?page=FunctionalRole&role=DedA%20family%20inner%20membrane%20protein%20YabI&subsystem_name=DedA_family_of_inner_membrane_proteins) |
| Miscellaneous | Miscellaneous - no subcategory | [DedA family of inner membrane proteins](https://rast.nmpdr.org/seedviewer.cgi?page=Subsystems&subsystem=DedA_family_of_inner_membrane_proteins&organism=549.408) | [DedA family inner membrane protein YohD](https://rast.nmpdr.org/seedviewer.cgi?page=FunctionalRole&role=DedA%20family%20inner%20membrane%20protein%20YohD&subsystem_name=DedA_family_of_inner_membrane_proteins) |
| Miscellaneous | Miscellaneous - no subcategory | [DedA family of inner membrane proteins](https://rast.nmpdr.org/seedviewer.cgi?page=Subsystems&subsystem=DedA_family_of_inner_membrane_proteins&organism=549.408) | [DedA family inner membrane protein YghB](https://rast.nmpdr.org/seedviewer.cgi?page=FunctionalRole&role=DedA%20family%20inner%20membrane%20protein%20YghB&subsystem_name=DedA_family_of_inner_membrane_proteins) |
| Miscellaneous | Miscellaneous - no subcategory | [DedA family of inner membrane proteins](https://rast.nmpdr.org/seedviewer.cgi?page=Subsystems&subsystem=DedA_family_of_inner_membrane_proteins&organism=549.408) | [DedA family inner membrane protein YqjA](https://rast.nmpdr.org/seedviewer.cgi?page=FunctionalRole&role=DedA%20family%20inner%20membrane%20protein%20YqjA&subsystem_name=DedA_family_of_inner_membrane_proteins) |
| Miscellaneous | Miscellaneous - no subcategory | [DedA family of inner membrane proteins](https://rast.nmpdr.org/seedviewer.cgi?page=Subsystems&subsystem=DedA_family_of_inner_membrane_proteins&organism=549.408) | [DedA protein](https://rast.nmpdr.org/seedviewer.cgi?page=FunctionalRole&role=DedA%20protein&subsystem_name=DedA_family_of_inner_membrane_proteins) |
| Miscellaneous | Miscellaneous - no subcategory | [Muconate lactonizing enzyme family](https://rast.nmpdr.org/seedviewer.cgi?page=Subsystems&subsystem=Muconate_lactonizing_enzyme_family&organism=549.408) | [L-alanine-DL-glutamate epimerase](https://rast.nmpdr.org/seedviewer.cgi?page=FunctionalRole&role=L-alanine-DL-glutamate%20epimerase&subsystem_name=Muconate_lactonizing_enzyme_family) |
| Miscellaneous | Miscellaneous - no subcategory | [Muconate lactonizing enzyme family](https://rast.nmpdr.org/seedviewer.cgi?page=Subsystems&subsystem=Muconate_lactonizing_enzyme_family&organism=549.408) | [Muconate cycloisomerase (EC 5.5.1.1)](https://rast.nmpdr.org/seedviewer.cgi?page=FunctionalRole&role=Muconate%20cycloisomerase%20(EC%205.5.1.1)&subsystem_name=Muconate_lactonizing_enzyme_family) |
| Miscellaneous | Miscellaneous - no subcategory | [Broadly distributed proteins not in subsystems](https://rast.nmpdr.org/seedviewer.cgi?page=Subsystems&subsystem=Broadly_distributed_proteins_not_in_subsystems&organism=549.408) | [YpfJ protein, zinc metalloprotease superfamily](https://rast.nmpdr.org/seedviewer.cgi?page=FunctionalRole&role=YpfJ%20protein,%20zinc%20metalloprotease%20superfamily&subsystem_name=Broadly_distributed_proteins_not_in_subsystems) |
| Miscellaneous | Miscellaneous - no subcategory | [Broadly distributed proteins not in subsystems](https://rast.nmpdr.org/seedviewer.cgi?page=Subsystems&subsystem=Broadly_distributed_proteins_not_in_subsystems&organism=549.408) | [YbbM seven transmembrane helix protein](https://rast.nmpdr.org/seedviewer.cgi?page=FunctionalRole&role=YbbM%20seven%20transmembrane%20helix%20protein&subsystem_name=Broadly_distributed_proteins_not_in_subsystems) |
| Miscellaneous | Miscellaneous - no subcategory | [Broadly distributed proteins not in subsystems](https://rast.nmpdr.org/seedviewer.cgi?page=Subsystems&subsystem=Broadly_distributed_proteins_not_in_subsystems&organism=549.408) | [YrbA protein](https://rast.nmpdr.org/seedviewer.cgi?page=FunctionalRole&role=YrbA%20protein&subsystem_name=Broadly_distributed_proteins_not_in_subsystems) |
| Miscellaneous | Miscellaneous - no subcategory | [Broadly distributed proteins not in subsystems](https://rast.nmpdr.org/seedviewer.cgi?page=Subsystems&subsystem=Broadly_distributed_proteins_not_in_subsystems&organism=549.408) | [UPF0225 protein YchJ](https://rast.nmpdr.org/seedviewer.cgi?page=FunctionalRole&role=UPF0225%20protein%20YchJ&subsystem_name=Broadly_distributed_proteins_not_in_subsystems) |
| Miscellaneous | Miscellaneous - no subcategory | [Broadly distributed proteins not in subsystems](https://rast.nmpdr.org/seedviewer.cgi?page=Subsystems&subsystem=Broadly_distributed_proteins_not_in_subsystems&organism=549.408) | [Putative oxidoreductase YncB](https://rast.nmpdr.org/seedviewer.cgi?page=FunctionalRole&role=Putative%20oxidoreductase%20YncB&subsystem_name=Broadly_distributed_proteins_not_in_subsystems) |
| Miscellaneous | Miscellaneous - no subcategory | [Broadly distributed proteins not in subsystems](https://rast.nmpdr.org/seedviewer.cgi?page=Subsystems&subsystem=Broadly_distributed_proteins_not_in_subsystems&organism=549.408) | [UPF0028 protein YchK](https://rast.nmpdr.org/seedviewer.cgi?page=FunctionalRole&role=UPF0028%20protein%20YchK&subsystem_name=Broadly_distributed_proteins_not_in_subsystems) |
| Miscellaneous | Miscellaneous - no subcategory | [Broadly distributed proteins not in subsystems](https://rast.nmpdr.org/seedviewer.cgi?page=Subsystems&subsystem=Broadly_distributed_proteins_not_in_subsystems&organism=549.408) | [YbbL ABC transporter ATP-binding protein](https://rast.nmpdr.org/seedviewer.cgi?page=FunctionalRole&role=YbbL%20ABC%20transporter%20ATP-binding%20protein&subsystem_name=Broadly_distributed_proteins_not_in_subsystems) |
| Miscellaneous | Miscellaneous - no subcategory | [Broadly distributed proteins not in subsystems](https://rast.nmpdr.org/seedviewer.cgi?page=Subsystems&subsystem=Broadly_distributed_proteins_not_in_subsystems&organism=549.408) | [YciL protein](https://rast.nmpdr.org/seedviewer.cgi?page=FunctionalRole&role=YciL%20protein&subsystem_name=Broadly_distributed_proteins_not_in_subsystems) |
| Miscellaneous | Miscellaneous - no subcategory | [Broadly distributed proteins not in subsystems](https://rast.nmpdr.org/seedviewer.cgi?page=Subsystems&subsystem=Broadly_distributed_proteins_not_in_subsystems&organism=549.408) | [FIG002473: Protein YcaR in KDO2-Lipid A biosynthesis cluster](https://rast.nmpdr.org/seedviewer.cgi?page=FunctionalRole&role=FIG002473:%20Protein%20YcaR%20in%20KDO2-Lipid%20A%20biosynthesis%20cluster&subsystem_name=Broadly_distributed_proteins_not_in_subsystems) |
| Miscellaneous | Miscellaneous - no subcategory | [Broadly distributed proteins not in subsystems](https://rast.nmpdr.org/seedviewer.cgi?page=Subsystems&subsystem=Broadly_distributed_proteins_not_in_subsystems&organism=549.408) | [FIG001943: hypothetical protein YajQ](https://rast.nmpdr.org/seedviewer.cgi?page=FunctionalRole&role=FIG001943:%20hypothetical%20protein%20YajQ&subsystem_name=Broadly_distributed_proteins_not_in_subsystems) |
| Miscellaneous | Miscellaneous - no subcategory | [Broadly distributed proteins not in subsystems](https://rast.nmpdr.org/seedviewer.cgi?page=Subsystems&subsystem=Broadly_distributed_proteins_not_in_subsystems&organism=549.408) | [UPF0265 protein YeeX](https://rast.nmpdr.org/seedviewer.cgi?page=FunctionalRole&role=UPF0265%20protein%20YeeX&subsystem_name=Broadly_distributed_proteins_not_in_subsystems) |
| Phages, Prophages, Transposable elements, Plasmids | Phages, Prophages | [Phage tail proteins](https://rast.nmpdr.org/seedviewer.cgi?page=Subsystems&subsystem=Phage_tail_proteins&organism=549.408) | [Phage major tail tube protein](https://rast.nmpdr.org/seedviewer.cgi?page=FunctionalRole&role=Phage%20major%20tail%20tube%20protein&subsystem_name=Phage_tail_proteins) |
| Phages, Prophages, Transposable elements, Plasmids | Phages, Prophages | [Phage tail proteins](https://rast.nmpdr.org/seedviewer.cgi?page=Subsystems&subsystem=Phage_tail_proteins&organism=549.408) | [Phage tail sheath monomer](https://rast.nmpdr.org/seedviewer.cgi?page=FunctionalRole&role=Phage%20tail%20sheath%20monomer&subsystem_name=Phage_tail_proteins) |
| Phages, Prophages, Transposable elements, Plasmids | Phages, Prophages | [Phage tail proteins](https://rast.nmpdr.org/seedviewer.cgi?page=Subsystems&subsystem=Phage_tail_proteins&organism=549.408) | [Phage tail completion protein](https://rast.nmpdr.org/seedviewer.cgi?page=FunctionalRole&role=Phage%20tail%20completion%20protein&subsystem_name=Phage_tail_proteins) |
| Phages, Prophages, Transposable elements, Plasmids | Phages, Prophages | [Phage tail proteins](https://rast.nmpdr.org/seedviewer.cgi?page=Subsystems&subsystem=Phage_tail_proteins&organism=549.408) | [Phage tail protein](https://rast.nmpdr.org/seedviewer.cgi?page=FunctionalRole&role=Phage%20tail%20protein&subsystem_name=Phage_tail_proteins) |
| Phages, Prophages, Transposable elements, Plasmids | Phages, Prophages | [Phage baseplate proteins](https://rast.nmpdr.org/seedviewer.cgi?page=Subsystems&subsystem=Phage_baseplate_proteins&organism=549.408) | [Phage baseplate hub](https://rast.nmpdr.org/seedviewer.cgi?page=FunctionalRole&role=Phage%20baseplate%20hub&subsystem_name=Phage_baseplate_proteins) |
| Phages, Prophages, Transposable elements, Plasmids | Phages, Prophages | [Phage baseplate proteins](https://rast.nmpdr.org/seedviewer.cgi?page=Subsystems&subsystem=Phage_baseplate_proteins&organism=549.408) | [Phage baseplate](https://rast.nmpdr.org/seedviewer.cgi?page=FunctionalRole&role=Phage%20baseplate&subsystem_name=Phage_baseplate_proteins) |
| Phages, Prophages, Transposable elements, Plasmids | Phages, Prophages | [Phage replication](https://rast.nmpdr.org/seedviewer.cgi?page=Subsystems&subsystem=Phage_replication&organism=549.408) | [DNA polymerase III alpha subunit (EC 2.7.7.7)](https://rast.nmpdr.org/seedviewer.cgi?page=FunctionalRole&role=DNA%20polymerase%20III%20alpha%20subunit%20(EC%202.7.7.7)&subsystem_name=Phage_replication) |
| Phages, Prophages, Transposable elements, Plasmids | Phages, Prophages | [Phage replication](https://rast.nmpdr.org/seedviewer.cgi?page=Subsystems&subsystem=Phage_replication&organism=549.408) | [Phage replication protein](https://rast.nmpdr.org/seedviewer.cgi?page=FunctionalRole&role=Phage%20replication%20protein&subsystem_name=Phage_replication) |
| Phages, Prophages, Transposable elements, Plasmids | Phages, Prophages | [Phage packaging machinery](https://rast.nmpdr.org/seedviewer.cgi?page=Subsystems&subsystem=Phage_packaging_machinery&organism=549.408) | [Phage terminase, ATPase subunit](https://rast.nmpdr.org/seedviewer.cgi?page=FunctionalRole&role=Phage%20terminase,%20ATPase%20subunit&subsystem_name=Phage_packaging_machinery) |
| Phages, Prophages, Transposable elements, Plasmids | Phages, Prophages | [Phage packaging machinery](https://rast.nmpdr.org/seedviewer.cgi?page=Subsystems&subsystem=Phage_packaging_machinery&organism=549.408) | [Phage terminase, endonuclease subunit](https://rast.nmpdr.org/seedviewer.cgi?page=FunctionalRole&role=Phage%20terminase,%20endonuclease%20subunit&subsystem_name=Phage_packaging_machinery) |
| Phages, Prophages, Transposable elements, Plasmids | Phages, Prophages | [Phage packaging machinery](https://rast.nmpdr.org/seedviewer.cgi?page=Subsystems&subsystem=Phage_packaging_machinery&organism=549.408) | [Phage DNA-binding protein](https://rast.nmpdr.org/seedviewer.cgi?page=FunctionalRole&role=Phage%20DNA-binding%20protein&subsystem_name=Phage_packaging_machinery) |
| Phages, Prophages, Transposable elements, Plasmids | Phages, Prophages | [Phage tail proteins 2](https://rast.nmpdr.org/seedviewer.cgi?page=Subsystems&subsystem=Phage_tail_proteins_2&organism=549.408) | [Phage tail sheath monomer](https://rast.nmpdr.org/seedviewer.cgi?page=FunctionalRole&role=Phage%20tail%20sheath%20monomer&subsystem_name=Phage_tail_proteins_2) |
| Phages, Prophages, Transposable elements, Plasmids | Phages, Prophages | [Phage tail proteins 2](https://rast.nmpdr.org/seedviewer.cgi?page=Subsystems&subsystem=Phage_tail_proteins_2&organism=549.408) | [Phage tail completion protein](https://rast.nmpdr.org/seedviewer.cgi?page=FunctionalRole&role=Phage%20tail%20completion%20protein&subsystem_name=Phage_tail_proteins_2) |
| Phages, Prophages, Transposable elements, Plasmids | Phages, Prophages | [Phage tail proteins 2](https://rast.nmpdr.org/seedviewer.cgi?page=Subsystems&subsystem=Phage_tail_proteins_2&organism=549.408) | [Phage tape measure](https://rast.nmpdr.org/seedviewer.cgi?page=FunctionalRole&role=Phage%20tape%20measure&subsystem_name=Phage_tail_proteins_2) |
| Phages, Prophages, Transposable elements, Plasmids | Phages, Prophages | [Phage tail fiber proteins](https://rast.nmpdr.org/seedviewer.cgi?page=Subsystems&subsystem=Phage_tail_fiber_proteins&organism=549.408) | [Phage tail fiber protein](https://rast.nmpdr.org/seedviewer.cgi?page=FunctionalRole&role=Phage%20tail%20fiber%20protein&subsystem_name=Phage_tail_fiber_proteins) |
| Phages, Prophages, Transposable elements, Plasmids | Phages, Prophages | [Phage tail fiber proteins](https://rast.nmpdr.org/seedviewer.cgi?page=Subsystems&subsystem=Phage_tail_fiber_proteins&organism=549.408) | [Phage tail fibers](https://rast.nmpdr.org/seedviewer.cgi?page=FunctionalRole&role=Phage%20tail%20fibers&subsystem_name=Phage_tail_fiber_proteins) |
| Phages, Prophages, Transposable elements, Plasmids | Phages, Prophages | [Phage tail fiber proteins](https://rast.nmpdr.org/seedviewer.cgi?page=Subsystems&subsystem=Phage_tail_fiber_proteins&organism=549.408) | [Phage tail fiber assembly protein](https://rast.nmpdr.org/seedviewer.cgi?page=FunctionalRole&role=Phage%20tail%20fiber%20assembly%20protein&subsystem_name=Phage_tail_fiber_proteins) |
| Phages, Prophages, Transposable elements, Plasmids | Phages, Prophages | [Phage capsid proteins](https://rast.nmpdr.org/seedviewer.cgi?page=Subsystems&subsystem=Phage_capsid_proteins&organism=549.408) | [Phage major capsid protein](https://rast.nmpdr.org/seedviewer.cgi?page=FunctionalRole&role=Phage%20major%20capsid%20protein&subsystem_name=Phage_capsid_proteins) |
| Phages, Prophages, Transposable elements, Plasmids | Phages, Prophages | [Phage capsid proteins](https://rast.nmpdr.org/seedviewer.cgi?page=Subsystems&subsystem=Phage_capsid_proteins&organism=549.408) | [Phage head completion-stabilization protein](https://rast.nmpdr.org/seedviewer.cgi?page=FunctionalRole&role=Phage%20head%20completion-stabilization%20protein&subsystem_name=Phage_capsid_proteins) |
| Phages, Prophages, Transposable elements, Plasmids | Phages, Prophages | [Phage capsid proteins](https://rast.nmpdr.org/seedviewer.cgi?page=Subsystems&subsystem=Phage_capsid_proteins&organism=549.408) | [Phage capsid scaffolding protein](https://rast.nmpdr.org/seedviewer.cgi?page=FunctionalRole&role=Phage%20capsid%20scaffolding%20protein&subsystem_name=Phage_capsid_proteins) |
| Membrane Transport | Protein secretion system, Type II | [CBSS-562.2.peg.633](https://rast.nmpdr.org/seedviewer.cgi?page=Subsystems&subsystem=CBSS-562.2.peg.633&organism=549.408) | [Type IV fimbrial assembly, ATPase PilB](https://rast.nmpdr.org/seedviewer.cgi?page=FunctionalRole&role=Type%20IV%20fimbrial%20assembly,%20ATPase%20PilB&subsystem_name=CBSS-562.2.peg.633) |
| Membrane Transport | Protein secretion system, Type II | [CBSS-562.2.peg.633](https://rast.nmpdr.org/seedviewer.cgi?page=Subsystems&subsystem=CBSS-562.2.peg.633&organism=549.408) | [FIG002842: hypothetical protein](https://rast.nmpdr.org/seedviewer.cgi?page=FunctionalRole&role=FIG002842:%20hypothetical%20protein&subsystem_name=CBSS-562.2.peg.633) |
| Membrane Transport | Protein secretion system, Type II | [CBSS-562.2.peg.633](https://rast.nmpdr.org/seedviewer.cgi?page=Subsystems&subsystem=CBSS-562.2.peg.633&organism=549.408) | [Dephospho-CoA kinase (EC 2.7.1.24)](https://rast.nmpdr.org/seedviewer.cgi?page=FunctionalRole&role=Dephospho-CoA%20kinase%20(EC%202.7.1.24)&subsystem_name=CBSS-562.2.peg.633) |
| Membrane Transport | Protein secretion system, Type II | [CBSS-562.2.peg.633](https://rast.nmpdr.org/seedviewer.cgi?page=Subsystems&subsystem=CBSS-562.2.peg.633&organism=549.408) | [FIG003276: zinc-binding protein](https://rast.nmpdr.org/seedviewer.cgi?page=FunctionalRole&role=FIG003276:%20zinc-binding%20protein&subsystem_name=CBSS-562.2.peg.633) |
| Membrane Transport | Protein secretion system, Type II | [CBSS-562.2.peg.633](https://rast.nmpdr.org/seedviewer.cgi?page=Subsystems&subsystem=CBSS-562.2.peg.633&organism=549.408) | [Type IV fimbrial assembly protein PilC](https://rast.nmpdr.org/seedviewer.cgi?page=FunctionalRole&role=Type%20IV%20fimbrial%20assembly%20protein%20PilC&subsystem_name=CBSS-562.2.peg.633) |
| Membrane Transport | Protein secretion system, Type II | [CBSS-562.2.peg.633](https://rast.nmpdr.org/seedviewer.cgi?page=Subsystems&subsystem=CBSS-562.2.peg.633&organism=549.408) | [Mutator mutT protein (7,8-dihydro-8-oxoguanine-triphosphatase) (EC 3.6.1.-)](https://rast.nmpdr.org/seedviewer.cgi?page=FunctionalRole&role=Mutator%20mutT%20protein%20(7,8-dihydro-8-oxoguanine-triphosphatase)%20(EC%203.6.1.-)&subsystem_name=CBSS-562.2.peg.633) |
| Membrane Transport | ABC transporters | [Peptide ABC transport system Sap](https://rast.nmpdr.org/seedviewer.cgi?page=Subsystems&subsystem=Peptide_ABC_transport_system_Sap&organism=549.408) | [Peptide transport system ATP-binding protein SapD](https://rast.nmpdr.org/seedviewer.cgi?page=FunctionalRole&role=Peptide%20transport%20system%20ATP-binding%20protein%20SapD&subsystem_name=Peptide_ABC_transport_system_Sap) |
| Membrane Transport | ABC transporters | [Peptide ABC transport system Sap](https://rast.nmpdr.org/seedviewer.cgi?page=Subsystems&subsystem=Peptide_ABC_transport_system_Sap&organism=549.408) | [Peptide transport periplasmic protein SapA](https://rast.nmpdr.org/seedviewer.cgi?page=FunctionalRole&role=Peptide%20transport%20periplasmic%20protein%20SapA&subsystem_name=Peptide_ABC_transport_system_Sap) |
| Membrane Transport | ABC transporters | [Peptide ABC transport system Sap](https://rast.nmpdr.org/seedviewer.cgi?page=Subsystems&subsystem=Peptide_ABC_transport_system_Sap&organism=549.408) | [Peptide transport system permease protein SapC](https://rast.nmpdr.org/seedviewer.cgi?page=FunctionalRole&role=Peptide%20transport%20system%20permease%20protein%20SapC&subsystem_name=Peptide_ABC_transport_system_Sap) |
| Membrane Transport | ABC transporters | [Peptide ABC transport system Sap](https://rast.nmpdr.org/seedviewer.cgi?page=Subsystems&subsystem=Peptide_ABC_transport_system_Sap&organism=549.408) | [Peptide transport system permease protein SapB](https://rast.nmpdr.org/seedviewer.cgi?page=FunctionalRole&role=Peptide%20transport%20system%20permease%20protein%20SapB&subsystem_name=Peptide_ABC_transport_system_Sap) |
| Membrane Transport | ABC transporters | [Peptide ABC transport system Sap](https://rast.nmpdr.org/seedviewer.cgi?page=Subsystems&subsystem=Peptide_ABC_transport_system_Sap&organism=549.408) | [Peptide transport system ATP-binding protein SapF](https://rast.nmpdr.org/seedviewer.cgi?page=FunctionalRole&role=Peptide%20transport%20system%20ATP-binding%20protein%20SapF&subsystem_name=Peptide_ABC_transport_system_Sap) |
| Membrane Transport | ABC transporters | [ABC transporter alkylphosphonate (TC 3.A.1.9.1)](https://rast.nmpdr.org/seedviewer.cgi?page=Subsystems&subsystem=ABC_transporter_alkylphosphonate_(TC_3.A.1.9.1)&organism=549.408) | [Phosphonate ABC transporter permease protein phnE2 (TC 3.A.1.9.1)](https://rast.nmpdr.org/seedviewer.cgi?page=FunctionalRole&role=Phosphonate%20ABC%20transporter%20permease%20protein%20phnE2%20(TC%203.A.1.9.1)&subsystem_name=ABC_transporter_alkylphosphonate_(TC_3.A.1.9.1)) |
| Membrane Transport | ABC transporters | [ABC transporter alkylphosphonate (TC 3.A.1.9.1)](https://rast.nmpdr.org/seedviewer.cgi?page=Subsystems&subsystem=ABC_transporter_alkylphosphonate_(TC_3.A.1.9.1)&organism=549.408) | [Phosphonate ABC transporter ATP-binding protein (TC 3.A.1.9.1)](https://rast.nmpdr.org/seedviewer.cgi?page=FunctionalRole&role=Phosphonate%20ABC%20transporter%20ATP-binding%20protein%20(TC%203.A.1.9.1)&subsystem_name=ABC_transporter_alkylphosphonate_(TC_3.A.1.9.1)) |
| Membrane Transport | ABC transporters | [ABC transporter alkylphosphonate (TC 3.A.1.9.1)](https://rast.nmpdr.org/seedviewer.cgi?page=Subsystems&subsystem=ABC_transporter_alkylphosphonate_(TC_3.A.1.9.1)&organism=549.408) | [Phosphonate ABC transporter permease protein phnE1 (TC 3.A.1.9.1)](https://rast.nmpdr.org/seedviewer.cgi?page=FunctionalRole&role=Phosphonate%20ABC%20transporter%20permease%20protein%20phnE1%20(TC%203.A.1.9.1)&subsystem_name=ABC_transporter_alkylphosphonate_(TC_3.A.1.9.1)) |
| Membrane Transport | ABC transporters | [ABC transporter alkylphosphonate (TC 3.A.1.9.1)](https://rast.nmpdr.org/seedviewer.cgi?page=Subsystems&subsystem=ABC_transporter_alkylphosphonate_(TC_3.A.1.9.1)&organism=549.408) | [Phosphonate ABC transporter phosphate-binding periplasmic component (TC 3.A.1.9.1)](https://rast.nmpdr.org/seedviewer.cgi?page=FunctionalRole&role=Phosphonate%20ABC%20transporter%20phosphate-binding%20periplasmic%20component%20(TC%203.A.1.9.1)&subsystem_name=ABC_transporter_alkylphosphonate_(TC_3.A.1.9.1)) |
| Membrane Transport | ABC transporters | [ABC transporter oligopeptide (TC 3.A.1.5.1)](https://rast.nmpdr.org/seedviewer.cgi?page=Subsystems&subsystem=ABC_transporter_oligopeptide_(TC_3.A.1.5.1)&organism=549.408) | [Oligopeptide transport system permease protein OppC (TC 3.A.1.5.1)](https://rast.nmpdr.org/seedviewer.cgi?page=FunctionalRole&role=Oligopeptide%20transport%20system%20permease%20protein%20OppC%20(TC%203.A.1.5.1)&subsystem_name=ABC_transporter_oligopeptide_(TC_3.A.1.5.1)) |
| Membrane Transport | ABC transporters | [ABC transporter oligopeptide (TC 3.A.1.5.1)](https://rast.nmpdr.org/seedviewer.cgi?page=Subsystems&subsystem=ABC_transporter_oligopeptide_(TC_3.A.1.5.1)&organism=549.408) | [Oligopeptide transport system permease protein OppB (TC 3.A.1.5.1)](https://rast.nmpdr.org/seedviewer.cgi?page=FunctionalRole&role=Oligopeptide%20transport%20system%20permease%20protein%20OppB%20(TC%203.A.1.5.1)&subsystem_name=ABC_transporter_oligopeptide_(TC_3.A.1.5.1)) |
| Membrane Transport | ABC transporters | [ABC transporter oligopeptide (TC 3.A.1.5.1)](https://rast.nmpdr.org/seedviewer.cgi?page=Subsystems&subsystem=ABC_transporter_oligopeptide_(TC_3.A.1.5.1)&organism=549.408) | [Oligopeptide transport ATP-binding protein OppF (TC 3.A.1.5.1)](https://rast.nmpdr.org/seedviewer.cgi?page=FunctionalRole&role=Oligopeptide%20transport%20ATP-binding%20protein%20OppF%20(TC%203.A.1.5.1)&subsystem_name=ABC_transporter_oligopeptide_(TC_3.A.1.5.1)) |
| Membrane Transport | ABC transporters | [ABC transporter oligopeptide (TC 3.A.1.5.1)](https://rast.nmpdr.org/seedviewer.cgi?page=Subsystems&subsystem=ABC_transporter_oligopeptide_(TC_3.A.1.5.1)&organism=549.408) | [Oligopeptide ABC transporter, periplasmic oligopeptide-binding protein OppA (TC 3.A.1.5.1)](https://rast.nmpdr.org/seedviewer.cgi?page=FunctionalRole&role=Oligopeptide%20ABC%20transporter,%20periplasmic%20oligopeptide-binding%20protein%20OppA%20(TC%203.A.1.5.1)&subsystem_name=ABC_transporter_oligopeptide_(TC_3.A.1.5.1)) |
| Membrane Transport | ABC transporters | [ABC transporter branched-chain amino acid (TC 3.A.1.4.1)](https://rast.nmpdr.org/seedviewer.cgi?page=Subsystems&subsystem=ABC_transporter_branched-chain_amino_acid_(TC_3.A.1.4.1)&organism=549.408) | [High-affinity leucine-specific transport system, periplasmic binding protein LivK (TC 3.A.1.4.1)](https://rast.nmpdr.org/seedviewer.cgi?page=FunctionalRole&role=High-affinity%20leucine-specific%20transport%20system,%20periplasmic%20binding%20protein%20LivK%20(TC%203.A.1.4.1)&subsystem_name=ABC_transporter_branched-chain_amino_acid_(TC_3.A.1.4.1)) |
| Membrane Transport | ABC transporters | [ABC transporter branched-chain amino acid (TC 3.A.1.4.1)](https://rast.nmpdr.org/seedviewer.cgi?page=Subsystems&subsystem=ABC_transporter_branched-chain_amino_acid_(TC_3.A.1.4.1)&organism=549.408) | [High-affinity branched-chain amino acid transport system permease protein LivH (TC 3.A.1.4.1)](https://rast.nmpdr.org/seedviewer.cgi?page=FunctionalRole&role=High-affinity%20branched-chain%20amino%20acid%20transport%20system%20permease%20protein%20LivH%20(TC%203.A.1.4.1)&subsystem_name=ABC_transporter_branched-chain_amino_acid_(TC_3.A.1.4.1)) |
| Membrane Transport | ABC transporters | [ABC transporter branched-chain amino acid (TC 3.A.1.4.1)](https://rast.nmpdr.org/seedviewer.cgi?page=Subsystems&subsystem=ABC_transporter_branched-chain_amino_acid_(TC_3.A.1.4.1)&organism=549.408) | [Branched-chain amino acid transport ATP-binding protein LivF (TC 3.A.1.4.1)](https://rast.nmpdr.org/seedviewer.cgi?page=FunctionalRole&role=Branched-chain%20amino%20acid%20transport%20ATP-binding%20protein%20LivF%20(TC%203.A.1.4.1)&subsystem_name=ABC_transporter_branched-chain_amino_acid_(TC_3.A.1.4.1)) |
| Membrane Transport | ABC transporters | [ABC transporter branched-chain amino acid (TC 3.A.1.4.1)](https://rast.nmpdr.org/seedviewer.cgi?page=Subsystems&subsystem=ABC_transporter_branched-chain_amino_acid_(TC_3.A.1.4.1)&organism=549.408) | [Branched-chain amino acid transport system permease protein LivM (TC 3.A.1.4.1)](https://rast.nmpdr.org/seedviewer.cgi?page=FunctionalRole&role=Branched-chain%20amino%20acid%20transport%20system%20permease%20protein%20LivM%20(TC%203.A.1.4.1)&subsystem_name=ABC_transporter_branched-chain_amino_acid_(TC_3.A.1.4.1)) |
| Membrane Transport | ABC transporters | [ABC transporter branched-chain amino acid (TC 3.A.1.4.1)](https://rast.nmpdr.org/seedviewer.cgi?page=Subsystems&subsystem=ABC_transporter_branched-chain_amino_acid_(TC_3.A.1.4.1)&organism=549.408) | [Branched-chain amino acid transport ATP-binding protein LivG (TC 3.A.1.4.1)](https://rast.nmpdr.org/seedviewer.cgi?page=FunctionalRole&role=Branched-chain%20amino%20acid%20transport%20ATP-binding%20protein%20LivG%20(TC%203.A.1.4.1)&subsystem_name=ABC_transporter_branched-chain_amino_acid_(TC_3.A.1.4.1)) |
| Membrane Transport | ABC transporters | [ABC transporter dipeptide (TC 3.A.1.5.2)](https://rast.nmpdr.org/seedviewer.cgi?page=Subsystems&subsystem=ABC_transporter_dipeptide_(TC_3.A.1.5.2)&organism=549.408) | [Dipeptide transport system permease protein DppB (TC 3.A.1.5.2)](https://rast.nmpdr.org/seedviewer.cgi?page=FunctionalRole&role=Dipeptide%20transport%20system%20permease%20protein%20DppB%20(TC%203.A.1.5.2)&subsystem_name=ABC_transporter_dipeptide_(TC_3.A.1.5.2)) |
| Membrane Transport | ABC transporters | [ABC transporter dipeptide (TC 3.A.1.5.2)](https://rast.nmpdr.org/seedviewer.cgi?page=Subsystems&subsystem=ABC_transporter_dipeptide_(TC_3.A.1.5.2)&organism=549.408) | [Dipeptide transport ATP-binding protein DppF (TC 3.A.1.5.2)](https://rast.nmpdr.org/seedviewer.cgi?page=FunctionalRole&role=Dipeptide%20transport%20ATP-binding%20protein%20DppF%20(TC%203.A.1.5.2)&subsystem_name=ABC_transporter_dipeptide_(TC_3.A.1.5.2)) |
| Membrane Transport | ABC transporters | [ABC transporter dipeptide (TC 3.A.1.5.2)](https://rast.nmpdr.org/seedviewer.cgi?page=Subsystems&subsystem=ABC_transporter_dipeptide_(TC_3.A.1.5.2)&organism=549.408) | [Dipeptide transport ATP-binding protein DppD (TC 3.A.1.5.2)](https://rast.nmpdr.org/seedviewer.cgi?page=FunctionalRole&role=Dipeptide%20transport%20ATP-binding%20protein%20DppD%20(TC%203.A.1.5.2)&subsystem_name=ABC_transporter_dipeptide_(TC_3.A.1.5.2)) |
| Membrane Transport | ABC transporters | [ABC transporter dipeptide (TC 3.A.1.5.2)](https://rast.nmpdr.org/seedviewer.cgi?page=Subsystems&subsystem=ABC_transporter_dipeptide_(TC_3.A.1.5.2)&organism=549.408) | [Dipeptide transport system permease protein DppC (TC 3.A.1.5.2)](https://rast.nmpdr.org/seedviewer.cgi?page=FunctionalRole&role=Dipeptide%20transport%20system%20permease%20protein%20DppC%20(TC%203.A.1.5.2)&subsystem_name=ABC_transporter_dipeptide_(TC_3.A.1.5.2)) |
| Membrane Transport | ABC transporters | [ABC transporter dipeptide (TC 3.A.1.5.2)](https://rast.nmpdr.org/seedviewer.cgi?page=Subsystems&subsystem=ABC_transporter_dipeptide_(TC_3.A.1.5.2)&organism=549.408) | [Dipeptide-binding ABC transporter, periplasmic substrate-binding component (TC 3.A.1.5.2)](https://rast.nmpdr.org/seedviewer.cgi?page=FunctionalRole&role=Dipeptide-binding%20ABC%20transporter,%20periplasmic%20substrate-binding%20component%20(TC%203.A.1.5.2)&subsystem_name=ABC_transporter_dipeptide_(TC_3.A.1.5.2)) |
| Membrane Transport | Protein secretion system, Type VII (Chaperone/Usher pathway, CU) | [Type 1 pili (mannose-sensitive fimbriae, gamma-fimbriae)](https://rast.nmpdr.org/seedviewer.cgi?page=Subsystems&subsystem=Type_1_pili_(mannose-sensitive_fimbriae,_gamma-fimbriae)&organism=549.408) | [type 1 fimbriae major subunit FimA](https://rast.nmpdr.org/seedviewer.cgi?page=FunctionalRole&role=type%201%20fimbriae%20major%20subunit%20FimA&subsystem_name=Type_1_pili_(mannose-sensitive_fimbriae,_gamma-fimbriae)) |
| Membrane Transport | Protein secretion system, Type VII (Chaperone/Usher pathway, CU) | [Type 1 pili (mannose-sensitive fimbriae, gamma-fimbriae)](https://rast.nmpdr.org/seedviewer.cgi?page=Subsystems&subsystem=Type_1_pili_(mannose-sensitive_fimbriae,_gamma-fimbriae)&organism=549.408) | [type 1 fimbriae anchoring protein FimD](https://rast.nmpdr.org/seedviewer.cgi?page=FunctionalRole&role=type%201%20fimbriae%20anchoring%20protein%20FimD&subsystem_name=Type_1_pili_(mannose-sensitive_fimbriae,_gamma-fimbriae)) |
| Membrane Transport | Protein secretion system, Type VII (Chaperone/Usher pathway, CU) | [Type 1 pili (mannose-sensitive fimbriae, gamma-fimbriae)](https://rast.nmpdr.org/seedviewer.cgi?page=Subsystems&subsystem=Type_1_pili_(mannose-sensitive_fimbriae,_gamma-fimbriae)&organism=549.408) | [chaperone FimC](https://rast.nmpdr.org/seedviewer.cgi?page=FunctionalRole&role=chaperone%20FimC&subsystem_name=Type_1_pili_(mannose-sensitive_fimbriae,_gamma-fimbriae)) |
| Membrane Transport | Protein secretion system, Type VII (Chaperone/Usher pathway, CU) | [sigma-Fimbriae](https://rast.nmpdr.org/seedviewer.cgi?page=Subsystems&subsystem=sigma-Fimbriae&organism=549.408) | [Sigma-fimbriae usher protein](https://rast.nmpdr.org/seedviewer.cgi?page=FunctionalRole&role=Sigma-fimbriae%20usher%20protein&subsystem_name=sigma-Fimbriae) |
| Membrane Transport | Protein secretion system, Type VII (Chaperone/Usher pathway, CU) | [sigma-Fimbriae](https://rast.nmpdr.org/seedviewer.cgi?page=Subsystems&subsystem=sigma-Fimbriae&organism=549.408) | [Sigma-fimbriae uncharacterized paralogous subunit](https://rast.nmpdr.org/seedviewer.cgi?page=FunctionalRole&role=Sigma-fimbriae%20uncharacterized%20paralogous%20subunit&subsystem_name=sigma-Fimbriae) |
| Membrane Transport | Protein secretion system, Type VII (Chaperone/Usher pathway, CU) | [sigma-Fimbriae](https://rast.nmpdr.org/seedviewer.cgi?page=Subsystems&subsystem=sigma-Fimbriae&organism=549.408) | [Sigma-fimbriae tip adhesin](https://rast.nmpdr.org/seedviewer.cgi?page=FunctionalRole&role=Sigma-fimbriae%20tip%20adhesin&subsystem_name=sigma-Fimbriae) |
| Membrane Transport | Protein secretion system, Type VII (Chaperone/Usher pathway, CU) | [sigma-Fimbriae](https://rast.nmpdr.org/seedviewer.cgi?page=Subsystems&subsystem=sigma-Fimbriae&organism=549.408) | [Sigma-fimbriae chaperone protein](https://rast.nmpdr.org/seedviewer.cgi?page=FunctionalRole&role=Sigma-fimbriae%20chaperone%20protein&subsystem_name=sigma-Fimbriae) |
| Membrane Transport | Protein translocation across cytoplasmic membrane | [Bacterial signal recognition particle (SRP)](https://rast.nmpdr.org/seedviewer.cgi?page=Subsystems&subsystem=Bacterial_signal_recognition_particle_(SRP)&organism=549.408) | [Signal recognition particle, subunit Ffh SRP54 (TC 3.A.5.1.1)](https://rast.nmpdr.org/seedviewer.cgi?page=FunctionalRole&role=Signal%20recognition%20particle,%20subunit%20Ffh%20SRP54%20(TC%203.A.5.1.1)&subsystem_name=Bacterial_signal_recognition_particle_(SRP)) |
| Membrane Transport | Protein translocation across cytoplasmic membrane | [Bacterial signal recognition particle (SRP)](https://rast.nmpdr.org/seedviewer.cgi?page=Subsystems&subsystem=Bacterial_signal_recognition_particle_(SRP)&organism=549.408) | [Signal recognition particle receptor protein FtsY (=alpha subunit) (TC 3.A.5.1.1)](https://rast.nmpdr.org/seedviewer.cgi?page=FunctionalRole&role=Signal%20recognition%20particle%20receptor%20protein%20FtsY%20(=alpha%20subunit)%20(TC%203.A.5.1.1)&subsystem_name=Bacterial_signal_recognition_particle_(SRP)) |
| Membrane Transport | Protein translocation across cytoplasmic membrane | [Twin-arginine translocation system](https://rast.nmpdr.org/seedviewer.cgi?page=Subsystems&subsystem=Twin-arginine_translocation_system&organism=549.408) | [Twin-arginine translocation protein TatC](https://rast.nmpdr.org/seedviewer.cgi?page=FunctionalRole&role=Twin-arginine%20translocation%20protein%20TatC&subsystem_name=Twin-arginine_translocation_system) |
| Membrane Transport | Protein translocation across cytoplasmic membrane | [Twin-arginine translocation system](https://rast.nmpdr.org/seedviewer.cgi?page=Subsystems&subsystem=Twin-arginine_translocation_system&organism=549.408) | [Twin-arginine translocation protein TatA](https://rast.nmpdr.org/seedviewer.cgi?page=FunctionalRole&role=Twin-arginine%20translocation%20protein%20TatA&subsystem_name=Twin-arginine_translocation_system) |
| Membrane Transport | Protein translocation across cytoplasmic membrane | [Twin-arginine translocation system](https://rast.nmpdr.org/seedviewer.cgi?page=Subsystems&subsystem=Twin-arginine_translocation_system&organism=549.408) | [Deoxyribonuclease TatD](https://rast.nmpdr.org/seedviewer.cgi?page=FunctionalRole&role=Deoxyribonuclease%20TatD&subsystem_name=Twin-arginine_translocation_system) |
| Membrane Transport | Protein translocation across cytoplasmic membrane | [Twin-arginine translocation system](https://rast.nmpdr.org/seedviewer.cgi?page=Subsystems&subsystem=Twin-arginine_translocation_system&organism=549.408) | [Twin-arginine translocation protein TatE](https://rast.nmpdr.org/seedviewer.cgi?page=FunctionalRole&role=Twin-arginine%20translocation%20protein%20TatE&subsystem_name=Twin-arginine_translocation_system) |
| Membrane Transport | Protein translocation across cytoplasmic membrane | [Twin-arginine translocation system](https://rast.nmpdr.org/seedviewer.cgi?page=Subsystems&subsystem=Twin-arginine_translocation_system&organism=549.408) | [Twin-arginine translocation protein TatB](https://rast.nmpdr.org/seedviewer.cgi?page=FunctionalRole&role=Twin-arginine%20translocation%20protein%20TatB&subsystem_name=Twin-arginine_translocation_system) |
| Membrane Transport | Protein secretion system, Type V | [Two partner secretion pathway (TPS)](https://rast.nmpdr.org/seedviewer.cgi?page=Subsystems&subsystem=Two_partner_secretion_pathway_(TPS)&organism=549.408) | [Channel-forming transporter/cytolysins activator of TpsB family](https://rast.nmpdr.org/seedviewer.cgi?page=FunctionalRole&role=Channel-forming%20transporter/cytolysins%20activator%20of%20TpsB%20family&subsystem_name=Two_partner_secretion_pathway_(TPS)) |
| Membrane Transport | Protein secretion system, Type V | [Two partner secretion pathway (TPS)](https://rast.nmpdr.org/seedviewer.cgi?page=Subsystems&subsystem=Two_partner_secretion_pathway_(TPS)&organism=549.408) | [Putative large exoprotein involved in heme utilization or adhesion of ShlA/HecA/FhaA family](https://rast.nmpdr.org/seedviewer.cgi?page=FunctionalRole&role=Putative%20large%20exoprotein%20involved%20in%20heme%20utilization%20or%20adhesion%20of%20ShlA/HecA/FhaA%20family&subsystem_name=Two_partner_secretion_pathway_(TPS)) |
| Membrane Transport | Protein secretion system, Type I | [Type I secretion system for aggregation](https://rast.nmpdr.org/seedviewer.cgi?page=Subsystems&subsystem=Type_I_secretion_system_for_aggregation&organism=549.408) | [Type I secretion system, outer membrane component LapE](https://rast.nmpdr.org/seedviewer.cgi?page=FunctionalRole&role=Type%20I%20secretion%20system,%20outer%20membrane%20component%20LapE&subsystem_name=Type_I_secretion_system_for_aggregation) |
| Membrane Transport | Protein secretion system, Type I | [Type I secretion system for aggregation](https://rast.nmpdr.org/seedviewer.cgi?page=Subsystems&subsystem=Type_I_secretion_system_for_aggregation&organism=549.408) | [Type I secretion system, membrane fusion protein LapC](https://rast.nmpdr.org/seedviewer.cgi?page=FunctionalRole&role=Type%20I%20secretion%20system,%20membrane%20fusion%20protein%20LapC&subsystem_name=Type_I_secretion_system_for_aggregation) |
| Membrane Transport | Protein secretion system, Type I | [Type I secretion system for aggregation](https://rast.nmpdr.org/seedviewer.cgi?page=Subsystems&subsystem=Type_I_secretion_system_for_aggregation&organism=549.408) | [Type I secretion system ATPase, LssB family LapB](https://rast.nmpdr.org/seedviewer.cgi?page=FunctionalRole&role=Type%20I%20secretion%20system%20ATPase,%20LssB%20family%20LapB&subsystem_name=Type_I_secretion_system_for_aggregation) |
| Membrane Transport | Protein secretion system, Type I | [Type I secretion system for aggregation](https://rast.nmpdr.org/seedviewer.cgi?page=Subsystems&subsystem=Type_I_secretion_system_for_aggregation&organism=549.408) | [T1SS secreted agglutinin RTX](https://rast.nmpdr.org/seedviewer.cgi?page=FunctionalRole&role=T1SS%20secreted%20agglutinin%20RTX&subsystem_name=Type_I_secretion_system_for_aggregation) |
| Membrane Transport | Cation transporters | [Magnesium transport](https://rast.nmpdr.org/seedviewer.cgi?page=Subsystems&subsystem=Magnesium_transport&organism=549.408) | [Mg(2+) transport ATPase protein C](https://rast.nmpdr.org/seedviewer.cgi?page=FunctionalRole&role=Mg(2+)%20transport%20ATPase%20protein%20C&subsystem_name=Magnesium_transport) |
| Membrane Transport | Cation transporters | [Magnesium transport](https://rast.nmpdr.org/seedviewer.cgi?page=Subsystems&subsystem=Magnesium_transport&organism=549.408) | [Magnesium and cobalt transport protein CorA](https://rast.nmpdr.org/seedviewer.cgi?page=FunctionalRole&role=Magnesium%20and%20cobalt%20transport%20protein%20CorA&subsystem_name=Magnesium_transport) |
| Membrane Transport | Cation transporters | [Magnesium transport](https://rast.nmpdr.org/seedviewer.cgi?page=Subsystems&subsystem=Magnesium_transport&organism=549.408) | [Magnesium and cobalt efflux protein CorC](https://rast.nmpdr.org/seedviewer.cgi?page=FunctionalRole&role=Magnesium%20and%20cobalt%20efflux%20protein%20CorC&subsystem_name=Magnesium_transport) |
| Membrane Transport | Cation transporters | [Copper Transport System](https://rast.nmpdr.org/seedviewer.cgi?page=Subsystems&subsystem=Copper_Transport_System&organism=549.408) | [Copper-translocating P-type ATPase (EC 3.6.3.4)](https://rast.nmpdr.org/seedviewer.cgi?page=FunctionalRole&role=Copper-translocating%20P-type%20ATPase%20(EC%203.6.3.4)&subsystem_name=Copper_Transport_System) |
| Membrane Transport | Uni- Sym- and Antiporters | [Proton-dependent Peptide Transporters](https://rast.nmpdr.org/seedviewer.cgi?page=Subsystems&subsystem=Proton-dependent_Peptide_Transporters&organism=549.408) | [Di/tripeptide permease DtpA](https://rast.nmpdr.org/seedviewer.cgi?page=FunctionalRole&role=Di/tripeptide%20permease%20DtpA&subsystem_name=Proton-dependent_Peptide_Transporters) |
| Membrane Transport | Uni- Sym- and Antiporters | [NhaA, NhaD and Sodium-dependent phosphate transporters](https://rast.nmpdr.org/seedviewer.cgi?page=Subsystems&subsystem=NhaA,_NhaD_and_Sodium-dependent_phosphate_transporters&organism=549.408) | [Na+/H+ antiporter NhaA type](https://rast.nmpdr.org/seedviewer.cgi?page=FunctionalRole&role=Na+/H+%20antiporter%20NhaA%20type&subsystem_name=NhaA,_NhaD_and_Sodium-dependent_phosphate_transporters) |
| Membrane Transport | Uni- Sym- and Antiporters | [NhaA, NhaD and Sodium-dependent phosphate transporters](https://rast.nmpdr.org/seedviewer.cgi?page=Subsystems&subsystem=NhaA,_NhaD_and_Sodium-dependent_phosphate_transporters&organism=549.408) | [Sodium-dependent phosphate transporter](https://rast.nmpdr.org/seedviewer.cgi?page=FunctionalRole&role=Sodium-dependent%20phosphate%20transporter&subsystem_name=NhaA,_NhaD_and_Sodium-dependent_phosphate_transporters) |
| Membrane Transport | Uni- Sym- and Antiporters | [NhaA, NhaD and Sodium-dependent phosphate transporters](https://rast.nmpdr.org/seedviewer.cgi?page=Subsystems&subsystem=NhaA,_NhaD_and_Sodium-dependent_phosphate_transporters&organism=549.408) | [Transcriptional activator NhaR](https://rast.nmpdr.org/seedviewer.cgi?page=FunctionalRole&role=Transcriptional%20activator%20NhaR&subsystem_name=NhaA,_NhaD_and_Sodium-dependent_phosphate_transporters) |
| Membrane Transport | Membrane Transport - no subcategory | [Tricarboxylate transport system](https://rast.nmpdr.org/seedviewer.cgi?page=Subsystems&subsystem=Tricarboxylate_transport_system&organism=549.408) | [Tricarboxylate transport sensor protein TctE](https://rast.nmpdr.org/seedviewer.cgi?page=FunctionalRole&role=Tricarboxylate%20transport%20sensor%20protein%20TctE&subsystem_name=Tricarboxylate_transport_system) |
| Membrane Transport | Membrane Transport - no subcategory | [Tricarboxylate transport system](https://rast.nmpdr.org/seedviewer.cgi?page=Subsystems&subsystem=Tricarboxylate_transport_system&organism=549.408) | [Tricarboxylate transport membrane protein TctA](https://rast.nmpdr.org/seedviewer.cgi?page=FunctionalRole&role=Tricarboxylate%20transport%20membrane%20protein%20TctA&subsystem_name=Tricarboxylate_transport_system) |
| Membrane Transport | Membrane Transport - no subcategory | [Tricarboxylate transport system](https://rast.nmpdr.org/seedviewer.cgi?page=Subsystems&subsystem=Tricarboxylate_transport_system&organism=549.408) | [Tricarboxylate transport transcriptional regulator TctD](https://rast.nmpdr.org/seedviewer.cgi?page=FunctionalRole&role=Tricarboxylate%20transport%20transcriptional%20regulator%20TctD&subsystem_name=Tricarboxylate_transport_system) |
| Membrane Transport | Membrane Transport - no subcategory | [Tricarboxylate transport system](https://rast.nmpdr.org/seedviewer.cgi?page=Subsystems&subsystem=Tricarboxylate_transport_system&organism=549.408) | [Tricarboxylate transport protein TctB](https://rast.nmpdr.org/seedviewer.cgi?page=FunctionalRole&role=Tricarboxylate%20transport%20protein%20TctB&subsystem_name=Tricarboxylate_transport_system) |
| Membrane Transport | Membrane Transport - no subcategory | [Tricarboxylate transport system](https://rast.nmpdr.org/seedviewer.cgi?page=Subsystems&subsystem=Tricarboxylate_transport_system&organism=549.408) | [Tricarboxylate transport protein TctC](https://rast.nmpdr.org/seedviewer.cgi?page=FunctionalRole&role=Tricarboxylate%20transport%20protein%20TctC&subsystem_name=Tricarboxylate_transport_system) |
| Membrane Transport | Membrane Transport - no subcategory | [Ton and Tol transport systems](https://rast.nmpdr.org/seedviewer.cgi?page=Subsystems&subsystem=Ton_and_Tol_transport_systems&organism=549.408) | [Putative OMR family iron-siderophore receptor precursor](https://rast.nmpdr.org/seedviewer.cgi?page=FunctionalRole&role=Putative%20OMR%20family%20iron-siderophore%20receptor%20precursor&subsystem_name=Ton_and_Tol_transport_systems) |
| Membrane Transport | Membrane Transport - no subcategory | [Ton and Tol transport systems](https://rast.nmpdr.org/seedviewer.cgi?page=Subsystems&subsystem=Ton_and_Tol_transport_systems&organism=549.408) | [Ferric siderophore transport system, periplasmic binding protein TonB](https://rast.nmpdr.org/seedviewer.cgi?page=FunctionalRole&role=Ferric%20siderophore%20transport%20system,%20periplasmic%20binding%20protein%20TonB&subsystem_name=Ton_and_Tol_transport_systems) |
| Membrane Transport | Membrane Transport - no subcategory | [Ton and Tol transport systems](https://rast.nmpdr.org/seedviewer.cgi?page=Subsystems&subsystem=Ton_and_Tol_transport_systems&organism=549.408) | [Tol biopolymer transport system, TolR protein](https://rast.nmpdr.org/seedviewer.cgi?page=FunctionalRole&role=Tol%20biopolymer%20transport%20system,%20TolR%20protein&subsystem_name=Ton_and_Tol_transport_systems) |
| Membrane Transport | Membrane Transport - no subcategory | [Ton and Tol transport systems](https://rast.nmpdr.org/seedviewer.cgi?page=Subsystems&subsystem=Ton_and_Tol_transport_systems&organism=549.408) | [TonB-dependent receptor](https://rast.nmpdr.org/seedviewer.cgi?page=FunctionalRole&role=TonB-dependent%20receptor&subsystem_name=Ton_and_Tol_transport_systems) |
| Membrane Transport | Membrane Transport - no subcategory | [Ton and Tol transport systems](https://rast.nmpdr.org/seedviewer.cgi?page=Subsystems&subsystem=Ton_and_Tol_transport_systems&organism=549.408) | [Peptidoglycan-associated lipoprotein precursor](https://rast.nmpdr.org/seedviewer.cgi?page=FunctionalRole&role=Peptidoglycan-associated%20lipoprotein%20precursor&subsystem_name=Ton_and_Tol_transport_systems) |
| Membrane Transport | Membrane Transport - no subcategory | [Ton and Tol transport systems](https://rast.nmpdr.org/seedviewer.cgi?page=Subsystems&subsystem=Ton_and_Tol_transport_systems&organism=549.408) | [Outer membrane lipoprotein omp16 precursor](https://rast.nmpdr.org/seedviewer.cgi?page=FunctionalRole&role=Outer%20membrane%20lipoprotein%20omp16%20precursor&subsystem_name=Ton_and_Tol_transport_systems) |
| Membrane Transport | Membrane Transport - no subcategory | [Ton and Tol transport systems](https://rast.nmpdr.org/seedviewer.cgi?page=Subsystems&subsystem=Ton_and_Tol_transport_systems&organism=549.408) | [TPR repeat containing exported protein](https://rast.nmpdr.org/seedviewer.cgi?page=FunctionalRole&role=TPR%20repeat%20containing%20exported%20protein&subsystem_name=Ton_and_Tol_transport_systems) |
| Membrane Transport | Membrane Transport - no subcategory | [Ton and Tol transport systems](https://rast.nmpdr.org/seedviewer.cgi?page=Subsystems&subsystem=Ton_and_Tol_transport_systems&organism=549.408) | [tolB protein precursor, periplasmic protein involved in the tonb-independent uptake of group A colicins](https://rast.nmpdr.org/seedviewer.cgi?page=FunctionalRole&role=tolB%20protein%20precursor,%20periplasmic%20protein%20involved%20in%20the%20tonb-independent%20uptake%20of%20group%20A%20colicins&subsystem_name=Ton_and_Tol_transport_systems) |
| Membrane Transport | Membrane Transport - no subcategory | [Ton and Tol transport systems](https://rast.nmpdr.org/seedviewer.cgi?page=Subsystems&subsystem=Ton_and_Tol_transport_systems&organism=549.408) | [Biopolymer transport protein ExbD/TolR](https://rast.nmpdr.org/seedviewer.cgi?page=FunctionalRole&role=Biopolymer%20transport%20protein%20ExbD/TolR&subsystem_name=Ton_and_Tol_transport_systems) |
| Membrane Transport | Membrane Transport - no subcategory | [Ton and Tol transport systems](https://rast.nmpdr.org/seedviewer.cgi?page=Subsystems&subsystem=Ton_and_Tol_transport_systems&organism=549.408) | [TolA protein](https://rast.nmpdr.org/seedviewer.cgi?page=FunctionalRole&role=TolA%20protein&subsystem_name=Ton_and_Tol_transport_systems) |
| Membrane Transport | Membrane Transport - no subcategory | [Ton and Tol transport systems](https://rast.nmpdr.org/seedviewer.cgi?page=Subsystems&subsystem=Ton_and_Tol_transport_systems&organism=549.408) | [4-hydroxybenzoyl-CoA thioesterase family active site](https://rast.nmpdr.org/seedviewer.cgi?page=FunctionalRole&role=4-hydroxybenzoyl-CoA%20thioesterase%20family%20active%20site&subsystem_name=Ton_and_Tol_transport_systems) |
| Membrane Transport | Membrane Transport - no subcategory | [Ton and Tol transport systems](https://rast.nmpdr.org/seedviewer.cgi?page=Subsystems&subsystem=Ton_and_Tol_transport_systems&organism=549.408) | [Protein-L-isoaspartate O-methyltransferase (EC 2.1.1.77)](https://rast.nmpdr.org/seedviewer.cgi?page=FunctionalRole&role=Protein-L-isoaspartate%20O-methyltransferase%20(EC%202.1.1.77)&subsystem_name=Ton_and_Tol_transport_systems) |
| Membrane Transport | Membrane Transport - no subcategory | [Ton and Tol transport systems](https://rast.nmpdr.org/seedviewer.cgi?page=Subsystems&subsystem=Ton_and_Tol_transport_systems&organism=549.408) | [MotA/TolQ/ExbB proton channel family protein](https://rast.nmpdr.org/seedviewer.cgi?page=FunctionalRole&role=MotA/TolQ/ExbB%20proton%20channel%20family%20protein&subsystem_name=Ton_and_Tol_transport_systems) |
| Membrane Transport | Membrane Transport - no subcategory | [Ton and Tol transport systems](https://rast.nmpdr.org/seedviewer.cgi?page=Subsystems&subsystem=Ton_and_Tol_transport_systems&organism=549.408) | [Type I secretion outer membrane protein, TolC precursor](https://rast.nmpdr.org/seedviewer.cgi?page=FunctionalRole&role=Type%20I%20secretion%20outer%20membrane%20protein,%20TolC%20precursor&subsystem_name=Ton_and_Tol_transport_systems) |
| Membrane Transport | Membrane Transport - no subcategory | [Ton and Tol transport systems](https://rast.nmpdr.org/seedviewer.cgi?page=Subsystems&subsystem=Ton_and_Tol_transport_systems&organism=549.408) | [TonB-dependent hemin , ferrichrome receptor](https://rast.nmpdr.org/seedviewer.cgi?page=FunctionalRole&role=TonB-dependent%20hemin%20,%20ferrichrome%20receptor&subsystem_name=Ton_and_Tol_transport_systems) |
| Membrane Transport | TRAP transporters | [TRAP Transporter collection](https://rast.nmpdr.org/seedviewer.cgi?page=Subsystems&subsystem=TRAP_Transporter_collection&organism=549.408) | [TRAP-type C4-dicarboxylate transport system, large permease component](https://rast.nmpdr.org/seedviewer.cgi?page=FunctionalRole&role=TRAP-type%20C4-dicarboxylate%20transport%20system,%20large%20permease%20component&subsystem_name=TRAP_Transporter_collection) |
| Membrane Transport | TRAP transporters | [TRAP Transporter collection](https://rast.nmpdr.org/seedviewer.cgi?page=Subsystems&subsystem=TRAP_Transporter_collection&organism=549.408) | [TRAP-type C4-dicarboxylate transport system, periplasmic component](https://rast.nmpdr.org/seedviewer.cgi?page=FunctionalRole&role=TRAP-type%20C4-dicarboxylate%20transport%20system,%20periplasmic%20component&subsystem_name=TRAP_Transporter_collection) |
| Membrane Transport | Protein secretion system, Type VI | [Type VI secretion systems](https://rast.nmpdr.org/seedviewer.cgi?page=Subsystems&subsystem=Type_VI_secretion_systems&organism=549.408) | [IcmF-related protein](https://rast.nmpdr.org/seedviewer.cgi?page=FunctionalRole&role=IcmF-related%20protein&subsystem_name=Type_VI_secretion_systems) |
| Membrane Transport | Protein secretion system, Type VI | [Type VI secretion systems](https://rast.nmpdr.org/seedviewer.cgi?page=Subsystems&subsystem=Type_VI_secretion_systems&organism=549.408) | [Uncharacterized protein ImpI/VasC](https://rast.nmpdr.org/seedviewer.cgi?page=FunctionalRole&role=Uncharacterized%20protein%20ImpI/VasC&subsystem_name=Type_VI_secretion_systems) |
| Membrane Transport | Protein secretion system, Type VI | [Type VI secretion systems](https://rast.nmpdr.org/seedviewer.cgi?page=Subsystems&subsystem=Type_VI_secretion_systems&organism=549.408) | [Uncharacterized protein ImpF](https://rast.nmpdr.org/seedviewer.cgi?page=FunctionalRole&role=Uncharacterized%20protein%20ImpF&subsystem_name=Type_VI_secretion_systems) |
| Membrane Transport | Protein secretion system, Type VI | [Type VI secretion systems](https://rast.nmpdr.org/seedviewer.cgi?page=Subsystems&subsystem=Type_VI_secretion_systems&organism=549.408) | [Uncharacterized protein ImpB](https://rast.nmpdr.org/seedviewer.cgi?page=FunctionalRole&role=Uncharacterized%20protein%20ImpB&subsystem_name=Type_VI_secretion_systems) |
| Membrane Transport | Protein secretion system, Type VI | [Type VI secretion systems](https://rast.nmpdr.org/seedviewer.cgi?page=Subsystems&subsystem=Type_VI_secretion_systems&organism=549.408) | [Protein of avirulence locus ImpE](https://rast.nmpdr.org/seedviewer.cgi?page=FunctionalRole&role=Protein%20of%20avirulence%20locus%20ImpE&subsystem_name=Type_VI_secretion_systems) |
| Membrane Transport | Protein secretion system, Type VI | [Type VI secretion systems](https://rast.nmpdr.org/seedviewer.cgi?page=Subsystems&subsystem=Type_VI_secretion_systems&organism=549.408) | [Protein ImpG/VasA](https://rast.nmpdr.org/seedviewer.cgi?page=FunctionalRole&role=Protein%20ImpG/VasA&subsystem_name=Type_VI_secretion_systems) |
| Membrane Transport | Protein secretion system, Type VI | [Type VI secretion systems](https://rast.nmpdr.org/seedviewer.cgi?page=Subsystems&subsystem=Type_VI_secretion_systems&organism=549.408) | [Uncharacterized protein ImpA](https://rast.nmpdr.org/seedviewer.cgi?page=FunctionalRole&role=Uncharacterized%20protein%20ImpA&subsystem_name=Type_VI_secretion_systems) |
| Membrane Transport | Protein secretion system, Type VI | [Type VI secretion systems](https://rast.nmpdr.org/seedviewer.cgi?page=Subsystems&subsystem=Type_VI_secretion_systems&organism=549.408) | [Outer membrane protein ImpK/VasF, OmpA/MotB domain](https://rast.nmpdr.org/seedviewer.cgi?page=FunctionalRole&role=Outer%20membrane%20protein%20ImpK/VasF,%20OmpA/MotB%20domain&subsystem_name=Type_VI_secretion_systems) |
| Membrane Transport | Protein secretion system, Type VI | [Type VI secretion systems](https://rast.nmpdr.org/seedviewer.cgi?page=Subsystems&subsystem=Type_VI_secretion_systems&organism=549.408) | [ClpB protein](https://rast.nmpdr.org/seedviewer.cgi?page=FunctionalRole&role=ClpB%20protein&subsystem_name=Type_VI_secretion_systems) |
| Membrane Transport | Protein secretion system, Type VI | [Type VI secretion systems](https://rast.nmpdr.org/seedviewer.cgi?page=Subsystems&subsystem=Type_VI_secretion_systems&organism=549.408) | [Uncharacterized protein ImpC](https://rast.nmpdr.org/seedviewer.cgi?page=FunctionalRole&role=Uncharacterized%20protein%20ImpC&subsystem_name=Type_VI_secretion_systems) |
| Membrane Transport | Protein secretion system, Type VI | [Type VI secretion systems](https://rast.nmpdr.org/seedviewer.cgi?page=Subsystems&subsystem=Type_VI_secretion_systems&organism=549.408) | [Uncharacterized protein ImpJ/VasE](https://rast.nmpdr.org/seedviewer.cgi?page=FunctionalRole&role=Uncharacterized%20protein%20ImpJ/VasE&subsystem_name=Type_VI_secretion_systems) |
| Membrane Transport | Protein secretion system, Type VI | [Type VI secretion systems](https://rast.nmpdr.org/seedviewer.cgi?page=Subsystems&subsystem=Type_VI_secretion_systems&organism=549.408) | [Uncharacterized protein ImpD](https://rast.nmpdr.org/seedviewer.cgi?page=FunctionalRole&role=Uncharacterized%20protein%20ImpD&subsystem_name=Type_VI_secretion_systems) |
| Membrane Transport | Protein secretion system, Type VI | [Type VI secretion systems](https://rast.nmpdr.org/seedviewer.cgi?page=Subsystems&subsystem=Type_VI_secretion_systems&organism=549.408) | [Protein phosphatase ImpM](https://rast.nmpdr.org/seedviewer.cgi?page=FunctionalRole&role=Protein%20phosphatase%20ImpM&subsystem_name=Type_VI_secretion_systems) |
| Membrane Transport | Protein secretion system, Type VI | [Type VI secretion systems](https://rast.nmpdr.org/seedviewer.cgi?page=Subsystems&subsystem=Type_VI_secretion_systems&organism=549.408) | [VgrG protein](https://rast.nmpdr.org/seedviewer.cgi?page=FunctionalRole&role=VgrG%20protein&subsystem_name=Type_VI_secretion_systems) |
| Membrane Transport | Protein secretion system, Type VI | [Type VI secretion systems](https://rast.nmpdr.org/seedviewer.cgi?page=Subsystems&subsystem=Type_VI_secretion_systems&organism=549.408) | [Uncharacterized protein ImpH/VasB](https://rast.nmpdr.org/seedviewer.cgi?page=FunctionalRole&role=Uncharacterized%20protein%20ImpH/VasB&subsystem_name=Type_VI_secretion_systems) |
| Membrane Transport | Protein secretion system, Type VI | [Type VI secretion systems](https://rast.nmpdr.org/seedviewer.cgi?page=Subsystems&subsystem=Type_VI_secretion_systems&organism=549.408) | [Type VI secretion lipoprotein/VasD](https://rast.nmpdr.org/seedviewer.cgi?page=FunctionalRole&role=Type%20VI%20secretion%20lipoprotein/VasD&subsystem_name=Type_VI_secretion_systems) |
| Membrane Transport | Protein and nucleoprotein secretion system, Type IV | [Type IV pilus](https://rast.nmpdr.org/seedviewer.cgi?page=Subsystems&subsystem=Type_IV_pilus&organism=549.408) | [Type IV fimbrial assembly, ATPase PilB](https://rast.nmpdr.org/seedviewer.cgi?page=FunctionalRole&role=Type%20IV%20fimbrial%20assembly,%20ATPase%20PilB&subsystem_name=Type_IV_pilus) |
| Membrane Transport | Protein and nucleoprotein secretion system, Type IV | [Type IV pilus](https://rast.nmpdr.org/seedviewer.cgi?page=Subsystems&subsystem=Type_IV_pilus&organism=549.408) | [Type IV pilus biogenesis protein PilQ](https://rast.nmpdr.org/seedviewer.cgi?page=FunctionalRole&role=Type%20IV%20pilus%20biogenesis%20protein%20PilQ&subsystem_name=Type_IV_pilus) |
| Membrane Transport | Protein and nucleoprotein secretion system, Type IV | [Type IV pilus](https://rast.nmpdr.org/seedviewer.cgi?page=Subsystems&subsystem=Type_IV_pilus&organism=549.408) | [Type IV pilus biogenesis protein PilN](https://rast.nmpdr.org/seedviewer.cgi?page=FunctionalRole&role=Type%20IV%20pilus%20biogenesis%20protein%20PilN&subsystem_name=Type_IV_pilus) |
| Membrane Transport | Protein and nucleoprotein secretion system, Type IV | [Type IV pilus](https://rast.nmpdr.org/seedviewer.cgi?page=Subsystems&subsystem=Type_IV_pilus&organism=549.408) | [N-methyltransferase (EC 2.1.1.-)](https://rast.nmpdr.org/seedviewer.cgi?page=FunctionalRole&role=N-methyltransferase%20(EC%202.1.1.-)&subsystem_name=Type_IV_pilus) |
| Membrane Transport | Protein and nucleoprotein secretion system, Type IV | [Type IV pilus](https://rast.nmpdr.org/seedviewer.cgi?page=Subsystems&subsystem=Type_IV_pilus&organism=549.408) | [3-dehydroquinate synthase (EC 4.2.3.4)](https://rast.nmpdr.org/seedviewer.cgi?page=FunctionalRole&role=3-dehydroquinate%20synthase%20(EC%204.2.3.4)&subsystem_name=Type_IV_pilus) |
| Membrane Transport | Protein and nucleoprotein secretion system, Type IV | [Type IV pilus](https://rast.nmpdr.org/seedviewer.cgi?page=Subsystems&subsystem=Type_IV_pilus&organism=549.408) | [Type IV fimbrial assembly protein PilC](https://rast.nmpdr.org/seedviewer.cgi?page=FunctionalRole&role=Type%20IV%20fimbrial%20assembly%20protein%20PilC&subsystem_name=Type_IV_pilus) |
| Membrane Transport | Protein and nucleoprotein secretion system, Type IV | [Type IV pilus](https://rast.nmpdr.org/seedviewer.cgi?page=Subsystems&subsystem=Type_IV_pilus&organism=549.408) | [Twitching motility protein PilT](https://rast.nmpdr.org/seedviewer.cgi?page=FunctionalRole&role=Twitching%20motility%20protein%20PilT&subsystem_name=Type_IV_pilus) |
| Membrane Transport | Protein and nucleoprotein secretion system, Type IV | [Type IV pilus](https://rast.nmpdr.org/seedviewer.cgi?page=Subsystems&subsystem=Type_IV_pilus&organism=549.408) | [Leader peptidase (Prepilin peptidase) (EC 3.4.23.43)](https://rast.nmpdr.org/seedviewer.cgi?page=FunctionalRole&role=Leader%20peptidase%20(Prepilin%20peptidase)%20(EC%203.4.23.43)&subsystem_name=Type_IV_pilus) |
| Membrane Transport | Protein and nucleoprotein secretion system, Type IV | [Type IV pilus](https://rast.nmpdr.org/seedviewer.cgi?page=Subsystems&subsystem=Type_IV_pilus&organism=549.408) | [Type IV pilin PilA](https://rast.nmpdr.org/seedviewer.cgi?page=FunctionalRole&role=Type%20IV%20pilin%20PilA&subsystem_name=Type_IV_pilus) |
| Membrane Transport | Protein and nucleoprotein secretion system, Type IV | [Type IV pilus](https://rast.nmpdr.org/seedviewer.cgi?page=Subsystems&subsystem=Type_IV_pilus&organism=549.408) | [Multimodular transpeptidase-transglycosylase (EC 2.4.1.129) (EC 3.4.-.-)](https://rast.nmpdr.org/seedviewer.cgi?page=FunctionalRole&role=Multimodular%20transpeptidase-transglycosylase%20(EC%202.4.1.129)%20(EC%203.4.-.-)&subsystem_name=Type_IV_pilus) |
| Membrane Transport | Protein and nucleoprotein secretion system, Type IV | [Type IV pilus](https://rast.nmpdr.org/seedviewer.cgi?page=Subsystems&subsystem=Type_IV_pilus&organism=549.408) | [Type IV pilus biogenesis protein PilM](https://rast.nmpdr.org/seedviewer.cgi?page=FunctionalRole&role=Type%20IV%20pilus%20biogenesis%20protein%20PilM&subsystem_name=Type_IV_pilus) |
| Iron acquisition and metabolism | Siderophores | [Siderophore Enterobactin](https://rast.nmpdr.org/seedviewer.cgi?page=Subsystems&subsystem=Siderophore_Enterobactin&organism=549.408) | [Enterobactin esterase](https://rast.nmpdr.org/seedviewer.cgi?page=FunctionalRole&role=Enterobactin%20esterase&subsystem_name=Siderophore_Enterobactin) |
| Iron acquisition and metabolism | Siderophores | [Siderophore Enterobactin](https://rast.nmpdr.org/seedviewer.cgi?page=Subsystems&subsystem=Siderophore_Enterobactin&organism=549.408) | [2,3-dihydro-2,3-dihydroxybenzoate dehydrogenase (EC 1.3.1.28) [enterobactin] siderophore](https://rast.nmpdr.org/seedviewer.cgi?page=FunctionalRole&role=2,3-dihydro-2,3-dihydroxybenzoate%20dehydrogenase%20(EC%201.3.1.28)%20%5benterobactin%5d%20siderophore&subsystem_name=Siderophore_Enterobactin) |
| Iron acquisition and metabolism | Siderophores | [Siderophore Enterobactin](https://rast.nmpdr.org/seedviewer.cgi?page=Subsystems&subsystem=Siderophore_Enterobactin&organism=549.408) | [Ferric enterobactin transport system permease protein FepG (TC 3.A.1.14.2)](https://rast.nmpdr.org/seedviewer.cgi?page=FunctionalRole&role=Ferric%20enterobactin%20transport%20system%20permease%20protein%20FepG%20(TC%203.A.1.14.2)&subsystem_name=Siderophore_Enterobactin) |
| Iron acquisition and metabolism | Siderophores | [Siderophore Enterobactin](https://rast.nmpdr.org/seedviewer.cgi?page=Subsystems&subsystem=Siderophore_Enterobactin&organism=549.408) | [Ferric enterobactin-binding periplasmic protein FepB (TC 3.A.1.14.2)](https://rast.nmpdr.org/seedviewer.cgi?page=FunctionalRole&role=Ferric%20enterobactin-binding%20periplasmic%20protein%20FepB%20(TC%203.A.1.14.2)&subsystem_name=Siderophore_Enterobactin) |
| Iron acquisition and metabolism | Siderophores | [Siderophore Enterobactin](https://rast.nmpdr.org/seedviewer.cgi?page=Subsystems&subsystem=Siderophore_Enterobactin&organism=549.408) | [Ferric enterobactin transport ATP-binding protein FepC (TC 3.A.1.14.2)](https://rast.nmpdr.org/seedviewer.cgi?page=FunctionalRole&role=Ferric%20enterobactin%20transport%20ATP-binding%20protein%20FepC%20(TC%203.A.1.14.2)&subsystem_name=Siderophore_Enterobactin) |
| Iron acquisition and metabolism | Siderophores | [Siderophore Enterobactin](https://rast.nmpdr.org/seedviewer.cgi?page=Subsystems&subsystem=Siderophore_Enterobactin&organism=549.408) | [2,3-dihydroxybenzoate-AMP ligase (EC 2.7.7.58) [enterobactin] siderophore](https://rast.nmpdr.org/seedviewer.cgi?page=FunctionalRole&role=2,3-dihydroxybenzoate-AMP%20ligase%20(EC%202.7.7.58)%20%5benterobactin%5d%20siderophore&subsystem_name=Siderophore_Enterobactin) |
| Iron acquisition and metabolism | Siderophores | [Siderophore Enterobactin](https://rast.nmpdr.org/seedviewer.cgi?page=Subsystems&subsystem=Siderophore_Enterobactin&organism=549.408) | [FIG005032: Putative cytoplasmic protein YbdZ in enterobactin biosynthesis operon](https://rast.nmpdr.org/seedviewer.cgi?page=FunctionalRole&role=FIG005032:%20Putative%20cytoplasmic%20protein%20YbdZ%20in%20enterobactin%20biosynthesis%20operon&subsystem_name=Siderophore_Enterobactin) |
| Iron acquisition and metabolism | Siderophores | [Siderophore Enterobactin](https://rast.nmpdr.org/seedviewer.cgi?page=Subsystems&subsystem=Siderophore_Enterobactin&organism=549.408) | [Outer membrane receptor for ferric enterobactin and colicins B, D](https://rast.nmpdr.org/seedviewer.cgi?page=FunctionalRole&role=Outer%20membrane%20receptor%20for%20ferric%20enterobactin%20and%20colicins%20B,%20D&subsystem_name=Siderophore_Enterobactin) |
| Iron acquisition and metabolism | Siderophores | [Siderophore Enterobactin](https://rast.nmpdr.org/seedviewer.cgi?page=Subsystems&subsystem=Siderophore_Enterobactin&organism=549.408) | [Enterobactin exporter EntS](https://rast.nmpdr.org/seedviewer.cgi?page=FunctionalRole&role=Enterobactin%20exporter%20EntS&subsystem_name=Siderophore_Enterobactin) |
| Iron acquisition and metabolism | Siderophores | [Siderophore Enterobactin](https://rast.nmpdr.org/seedviewer.cgi?page=Subsystems&subsystem=Siderophore_Enterobactin&organism=549.408) | [Enterobactin synthetase component F, serine activating enzyme (EC 2.7.7.-)](https://rast.nmpdr.org/seedviewer.cgi?page=FunctionalRole&role=Enterobactin%20synthetase%20component%20F,%20serine%20activating%20enzyme%20(EC%202.7.7.-)&subsystem_name=Siderophore_Enterobactin) |
| Iron acquisition and metabolism | Siderophores | [Siderophore Enterobactin](https://rast.nmpdr.org/seedviewer.cgi?page=Subsystems&subsystem=Siderophore_Enterobactin&organism=549.408) | [Isochorismate synthase (EC 5.4.4.2) [enterobactin] siderophore](https://rast.nmpdr.org/seedviewer.cgi?page=FunctionalRole&role=Isochorismate%20synthase%20(EC%205.4.4.2)%20%5benterobactin%5d%20siderophore&subsystem_name=Siderophore_Enterobactin) |
| Iron acquisition and metabolism | Siderophores | [Siderophore Enterobactin](https://rast.nmpdr.org/seedviewer.cgi?page=Subsystems&subsystem=Siderophore_Enterobactin&organism=549.408) | [Apo-aryl carrier domain of EntB](https://rast.nmpdr.org/seedviewer.cgi?page=FunctionalRole&role=Apo-aryl%20carrier%20domain%20of%20EntB&subsystem_name=Siderophore_Enterobactin) |
| Iron acquisition and metabolism | Siderophores | [Siderophore Enterobactin](https://rast.nmpdr.org/seedviewer.cgi?page=Subsystems&subsystem=Siderophore_Enterobactin&organism=549.408) | [Proofreading thioesterase in enterobactin biosynthesis EntH](https://rast.nmpdr.org/seedviewer.cgi?page=FunctionalRole&role=Proofreading%20thioesterase%20in%20enterobactin%20biosynthesis%20EntH&subsystem_name=Siderophore_Enterobactin) |
| Iron acquisition and metabolism | Siderophores | [Siderophore Enterobactin](https://rast.nmpdr.org/seedviewer.cgi?page=Subsystems&subsystem=Siderophore_Enterobactin&organism=549.408) | [Isochorismatase (EC 3.3.2.1) [enterobactin] siderophore](https://rast.nmpdr.org/seedviewer.cgi?page=FunctionalRole&role=Isochorismatase%20(EC%203.3.2.1)%20%5benterobactin%5d%20siderophore&subsystem_name=Siderophore_Enterobactin) |
| Iron acquisition and metabolism | Siderophores | [Siderophore Enterobactin](https://rast.nmpdr.org/seedviewer.cgi?page=Subsystems&subsystem=Siderophore_Enterobactin&organism=549.408) | [Ferric enterobactin transport system permease protein FepD (TC 3.A.1.14.2)](https://rast.nmpdr.org/seedviewer.cgi?page=FunctionalRole&role=Ferric%20enterobactin%20transport%20system%20permease%20protein%20FepD%20(TC%203.A.1.14.2)&subsystem_name=Siderophore_Enterobactin) |
| Iron acquisition and metabolism | Siderophores | [Siderophore Aerobactin](https://rast.nmpdr.org/seedviewer.cgi?page=Subsystems&subsystem=Siderophore_Aerobactin&organism=549.408) | [Ferric hydroxamate outer membrane receptor FhuA](https://rast.nmpdr.org/seedviewer.cgi?page=FunctionalRole&role=Ferric%20hydroxamate%20outer%20membrane%20receptor%20FhuA&subsystem_name=Siderophore_Aerobactin) |
| Iron acquisition and metabolism | Siderophores | [Siderophore Aerobactin](https://rast.nmpdr.org/seedviewer.cgi?page=Subsystems&subsystem=Siderophore_Aerobactin&organism=549.408) | [Ferric hydroxamate ABC transporter (TC 3.A.1.14.3), permease component FhuB](https://rast.nmpdr.org/seedviewer.cgi?page=FunctionalRole&role=Ferric%20hydroxamate%20ABC%20transporter%20(TC%203.A.1.14.3),%20permease%20component%20FhuB&subsystem_name=Siderophore_Aerobactin) |
| Iron acquisition and metabolism | Siderophores | [Siderophore Aerobactin](https://rast.nmpdr.org/seedviewer.cgi?page=Subsystems&subsystem=Siderophore_Aerobactin&organism=549.408) | [Ferric hydroxamate ABC transporter (TC 3.A.1.14.3), ATP-binding protein FhuC](https://rast.nmpdr.org/seedviewer.cgi?page=FunctionalRole&role=Ferric%20hydroxamate%20ABC%20transporter%20(TC%203.A.1.14.3),%20ATP-binding%20protein%20FhuC&subsystem_name=Siderophore_Aerobactin) |
| Iron acquisition and metabolism | Siderophores | [Siderophore Aerobactin](https://rast.nmpdr.org/seedviewer.cgi?page=Subsystems&subsystem=Siderophore_Aerobactin&organism=549.408) | [Ferric hydroxamate ABC transporter (TC 3.A.1.14.3), periplasmic substrate binding protein FhuD](https://rast.nmpdr.org/seedviewer.cgi?page=FunctionalRole&role=Ferric%20hydroxamate%20ABC%20transporter%20(TC%203.A.1.14.3),%20periplasmic%20substrate%20binding%20protein%20FhuD&subsystem_name=Siderophore_Aerobactin) |
| Iron acquisition and metabolism | Iron acquisition and metabolism - no subcategory | [Iron acquisition in Streptococcus](https://rast.nmpdr.org/seedviewer.cgi?page=Subsystems&subsystem=Iron_acquisition_in_Streptococcus&organism=549.408) | [Ferric iron ABC transporter, ATP-binding protein](https://rast.nmpdr.org/seedviewer.cgi?page=FunctionalRole&role=Ferric%20iron%20ABC%20transporter,%20ATP-binding%20protein&subsystem_name=Iron_acquisition_in_Streptococcus) |
| Iron acquisition and metabolism | Iron acquisition and metabolism - no subcategory | [Iron acquisition in Streptococcus](https://rast.nmpdr.org/seedviewer.cgi?page=Subsystems&subsystem=Iron_acquisition_in_Streptococcus&organism=549.408) | [Ferric iron ABC transporter, iron-binding protein](https://rast.nmpdr.org/seedviewer.cgi?page=FunctionalRole&role=Ferric%20iron%20ABC%20transporter,%20iron-binding%20protein&subsystem_name=Iron_acquisition_in_Streptococcus) |
| Iron acquisition and metabolism | Iron acquisition and metabolism - no subcategory | [Iron acquisition in Streptococcus](https://rast.nmpdr.org/seedviewer.cgi?page=Subsystems&subsystem=Iron_acquisition_in_Streptococcus&organism=549.408) | [Ferric iron ABC transporter, permease protein](https://rast.nmpdr.org/seedviewer.cgi?page=FunctionalRole&role=Ferric%20iron%20ABC%20transporter,%20permease%20protein&subsystem_name=Iron_acquisition_in_Streptococcus) |
| Iron acquisition and metabolism | Iron acquisition and metabolism - no subcategory | [Heme, hemin uptake and utilization systems in GramPositives](https://rast.nmpdr.org/seedviewer.cgi?page=Subsystems&subsystem=Heme,_hemin_uptake_and_utilization_systems_in_GramPositives&organism=549.408) | [Hemin transport protein HmuS](https://rast.nmpdr.org/seedviewer.cgi?page=FunctionalRole&role=Hemin%20transport%20protein%20HmuS&subsystem_name=Heme,_hemin_uptake_and_utilization_systems_in_GramPositives) |
| Iron acquisition and metabolism | Iron acquisition and metabolism - no subcategory | [Ferrous iron transporter EfeUOB, low-pH-induced](https://rast.nmpdr.org/seedviewer.cgi?page=Subsystems&subsystem=Ferrous_iron_transporter_EfeUOB,_low-pH-induced&organism=549.408) | [Ferrous iron transport peroxidase EfeB](https://rast.nmpdr.org/seedviewer.cgi?page=FunctionalRole&role=Ferrous%20iron%20transport%20peroxidase%20EfeB&subsystem_name=Ferrous_iron_transporter_EfeUOB,_low-pH-induced) |
| Iron acquisition and metabolism | Iron acquisition and metabolism - no subcategory | [Ferrous iron transporter EfeUOB, low-pH-induced](https://rast.nmpdr.org/seedviewer.cgi?page=Subsystems&subsystem=Ferrous_iron_transporter_EfeUOB,_low-pH-induced&organism=549.408) | [Ferrous iron transport permease EfeU](https://rast.nmpdr.org/seedviewer.cgi?page=FunctionalRole&role=Ferrous%20iron%20transport%20permease%20EfeU&subsystem_name=Ferrous_iron_transporter_EfeUOB,_low-pH-induced) |
| Iron acquisition and metabolism | Iron acquisition and metabolism - no subcategory | [Ferrous iron transporter EfeUOB, low-pH-induced](https://rast.nmpdr.org/seedviewer.cgi?page=Subsystems&subsystem=Ferrous_iron_transporter_EfeUOB,_low-pH-induced&organism=549.408) | [Ferrous iron transport periplasmic protein EfeO, contains peptidase-M75 domain and (frequently) cupredoxin-like domain](https://rast.nmpdr.org/seedviewer.cgi?page=FunctionalRole&role=Ferrous%20iron%20transport%20periplasmic%20protein%20EfeO,%20contains%20peptidase-M75%20domain%20and%20(frequently)%20cupredoxin-like%20domain&subsystem_name=Ferrous_iron_transporter_EfeUOB,_low-pH-induced) |
| Iron acquisition and metabolism | Iron acquisition and metabolism - no subcategory | [Heme, hemin uptake and utilization systems in GramNegatives](https://rast.nmpdr.org/seedviewer.cgi?page=Subsystems&subsystem=Heme,_hemin_uptake_and_utilization_systems_in_GramNegatives&organism=549.408) | [Ferric siderophore transport system, periplasmic binding protein TonB](https://rast.nmpdr.org/seedviewer.cgi?page=FunctionalRole&role=Ferric%20siderophore%20transport%20system,%20periplasmic%20binding%20protein%20TonB&subsystem_name=Heme,_hemin_uptake_and_utilization_systems_in_GramNegatives) |
| Iron acquisition and metabolism | Iron acquisition and metabolism - no subcategory | [Heme, hemin uptake and utilization systems in GramNegatives](https://rast.nmpdr.org/seedviewer.cgi?page=Subsystems&subsystem=Heme,_hemin_uptake_and_utilization_systems_in_GramNegatives&organism=549.408) | [Ferric reductase (1.6.99.14)](https://rast.nmpdr.org/seedviewer.cgi?page=FunctionalRole&role=Ferric%20reductase%20(1.6.99.14)&subsystem_name=Heme,_hemin_uptake_and_utilization_systems_in_GramNegatives) |
| Iron acquisition and metabolism | Iron acquisition and metabolism - no subcategory | [Heme, hemin uptake and utilization systems in GramNegatives](https://rast.nmpdr.org/seedviewer.cgi?page=Subsystems&subsystem=Heme,_hemin_uptake_and_utilization_systems_in_GramNegatives&organism=549.408) | [Paraquat-inducible protein B](https://rast.nmpdr.org/seedviewer.cgi?page=FunctionalRole&role=Paraquat-inducible%20protein%20B&subsystem_name=Heme,_hemin_uptake_and_utilization_systems_in_GramNegatives) |
| Iron acquisition and metabolism | Iron acquisition and metabolism - no subcategory | [Heme, hemin uptake and utilization systems in GramNegatives](https://rast.nmpdr.org/seedviewer.cgi?page=Subsystems&subsystem=Heme,_hemin_uptake_and_utilization_systems_in_GramNegatives&organism=549.408) | [Periplasmic hemin-binding protein](https://rast.nmpdr.org/seedviewer.cgi?page=FunctionalRole&role=Periplasmic%20hemin-binding%20protein&subsystem_name=Heme,_hemin_uptake_and_utilization_systems_in_GramNegatives) |
| Iron acquisition and metabolism | Iron acquisition and metabolism - no subcategory | [Heme, hemin uptake and utilization systems in GramNegatives](https://rast.nmpdr.org/seedviewer.cgi?page=Subsystems&subsystem=Heme,_hemin_uptake_and_utilization_systems_in_GramNegatives&organism=549.408) | [ABC-type hemin transport system, ATPase component](https://rast.nmpdr.org/seedviewer.cgi?page=FunctionalRole&role=ABC-type%20hemin%20transport%20system,%20ATPase%20component&subsystem_name=Heme,_hemin_uptake_and_utilization_systems_in_GramNegatives) |
| Iron acquisition and metabolism | Iron acquisition and metabolism - no subcategory | [Heme, hemin uptake and utilization systems in GramNegatives](https://rast.nmpdr.org/seedviewer.cgi?page=Subsystems&subsystem=Heme,_hemin_uptake_and_utilization_systems_in_GramNegatives&organism=549.408) | [Hemin ABC transporter, permease protein](https://rast.nmpdr.org/seedviewer.cgi?page=FunctionalRole&role=Hemin%20ABC%20transporter,%20permease%20protein&subsystem_name=Heme,_hemin_uptake_and_utilization_systems_in_GramNegatives) |
| Iron acquisition and metabolism | Iron acquisition and metabolism - no subcategory | [Heme, hemin uptake and utilization systems in GramNegatives](https://rast.nmpdr.org/seedviewer.cgi?page=Subsystems&subsystem=Heme,_hemin_uptake_and_utilization_systems_in_GramNegatives&organism=549.408) | [Paraquat-inducible protein A](https://rast.nmpdr.org/seedviewer.cgi?page=FunctionalRole&role=Paraquat-inducible%20protein%20A&subsystem_name=Heme,_hemin_uptake_and_utilization_systems_in_GramNegatives) |
| Iron acquisition and metabolism | Iron acquisition and metabolism - no subcategory | [Heme, hemin uptake and utilization systems in GramNegatives](https://rast.nmpdr.org/seedviewer.cgi?page=Subsystems&subsystem=Heme,_hemin_uptake_and_utilization_systems_in_GramNegatives&organism=549.408) | [Hemin transport protein HmuS](https://rast.nmpdr.org/seedviewer.cgi?page=FunctionalRole&role=Hemin%20transport%20protein%20HmuS&subsystem_name=Heme,_hemin_uptake_and_utilization_systems_in_GramNegatives) |
| Iron acquisition and metabolism | Iron acquisition and metabolism - no subcategory | [Heme, hemin uptake and utilization systems in GramNegatives](https://rast.nmpdr.org/seedviewer.cgi?page=Subsystems&subsystem=Heme,_hemin_uptake_and_utilization_systems_in_GramNegatives&organism=549.408) | [TonB-dependent hemin , ferrichrome receptor](https://rast.nmpdr.org/seedviewer.cgi?page=FunctionalRole&role=TonB-dependent%20hemin%20,%20ferrichrome%20receptor&subsystem_name=Heme,_hemin_uptake_and_utilization_systems_in_GramNegatives) |
| Iron acquisition and metabolism | Iron acquisition and metabolism - no subcategory | [Heme, hemin uptake and utilization systems in GramNegatives](https://rast.nmpdr.org/seedviewer.cgi?page=Subsystems&subsystem=Heme,_hemin_uptake_and_utilization_systems_in_GramNegatives&organism=549.408) | [Ferrichrome transport ATP-binding protein FhuC (TC 3.A.1.14.3)](https://rast.nmpdr.org/seedviewer.cgi?page=FunctionalRole&role=Ferrichrome%20transport%20ATP-binding%20protein%20FhuC%20(TC%203.A.1.14.3)&subsystem_name=Heme,_hemin_uptake_and_utilization_systems_in_GramNegatives) |
| Iron acquisition and metabolism | Iron acquisition and metabolism - no subcategory | [Hemin transport system](https://rast.nmpdr.org/seedviewer.cgi?page=Subsystems&subsystem=Hemin_transport_system&organism=549.408) | [Periplasmic hemin-binding protein](https://rast.nmpdr.org/seedviewer.cgi?page=FunctionalRole&role=Periplasmic%20hemin-binding%20protein&subsystem_name=Hemin_transport_system) |
| Iron acquisition and metabolism | Iron acquisition and metabolism - no subcategory | [Hemin transport system](https://rast.nmpdr.org/seedviewer.cgi?page=Subsystems&subsystem=Hemin_transport_system&organism=549.408) | [ABC-type hemin transport system, ATPase component](https://rast.nmpdr.org/seedviewer.cgi?page=FunctionalRole&role=ABC-type%20hemin%20transport%20system,%20ATPase%20component&subsystem_name=Hemin_transport_system) |
| Iron acquisition and metabolism | Iron acquisition and metabolism - no subcategory | [Hemin transport system](https://rast.nmpdr.org/seedviewer.cgi?page=Subsystems&subsystem=Hemin_transport_system&organism=549.408) | [Ferric siderophore transport system, periplasmic binding protein TonB](https://rast.nmpdr.org/seedviewer.cgi?page=FunctionalRole&role=Ferric%20siderophore%20transport%20system,%20periplasmic%20binding%20protein%20TonB&subsystem_name=Hemin_transport_system) |
| Iron acquisition and metabolism | Iron acquisition and metabolism - no subcategory | [Hemin transport system](https://rast.nmpdr.org/seedviewer.cgi?page=Subsystems&subsystem=Hemin_transport_system&organism=549.408) | [Hemin ABC transporter, permease protein](https://rast.nmpdr.org/seedviewer.cgi?page=FunctionalRole&role=Hemin%20ABC%20transporter,%20permease%20protein&subsystem_name=Hemin_transport_system) |
| Iron acquisition and metabolism | Iron acquisition and metabolism - no subcategory | [Hemin transport system](https://rast.nmpdr.org/seedviewer.cgi?page=Subsystems&subsystem=Hemin_transport_system&organism=549.408) | [Ferric reductase (1.6.99.14)](https://rast.nmpdr.org/seedviewer.cgi?page=FunctionalRole&role=Ferric%20reductase%20(1.6.99.14)&subsystem_name=Hemin_transport_system) |
| Iron acquisition and metabolism | Iron acquisition and metabolism - no subcategory | [Hemin transport system](https://rast.nmpdr.org/seedviewer.cgi?page=Subsystems&subsystem=Hemin_transport_system&organism=549.408) | [Hemin transport protein HmuS](https://rast.nmpdr.org/seedviewer.cgi?page=FunctionalRole&role=Hemin%20transport%20protein%20HmuS&subsystem_name=Hemin_transport_system) |
| Iron acquisition and metabolism | Iron acquisition and metabolism - no subcategory | [Hemin transport system](https://rast.nmpdr.org/seedviewer.cgi?page=Subsystems&subsystem=Hemin_transport_system&organism=549.408) | [TonB-dependent hemin , ferrichrome receptor](https://rast.nmpdr.org/seedviewer.cgi?page=FunctionalRole&role=TonB-dependent%20hemin%20,%20ferrichrome%20receptor&subsystem_name=Hemin_transport_system) |
| RNA Metabolism | RNA processing and modification | [RNA pseudouridine syntheses](https://rast.nmpdr.org/seedviewer.cgi?page=Subsystems&subsystem=RNA_pseudouridine_syntheses&organism=549.408) | [Ribosomal large subunit pseudouridine synthase F (EC 4.2.1.70)](https://rast.nmpdr.org/seedviewer.cgi?page=FunctionalRole&role=Ribosomal%20large%20subunit%20pseudouridine%20synthase%20F%20(EC%204.2.1.70)&subsystem_name=RNA_pseudouridine_syntheses) |
| RNA Metabolism | RNA processing and modification | [RNA pseudouridine syntheses](https://rast.nmpdr.org/seedviewer.cgi?page=Subsystems&subsystem=RNA_pseudouridine_syntheses&organism=549.408) | [Ribosomal large subunit pseudouridine synthase A (EC 4.2.1.70)](https://rast.nmpdr.org/seedviewer.cgi?page=FunctionalRole&role=Ribosomal%20large%20subunit%20pseudouridine%20synthase%20A%20(EC%204.2.1.70)&subsystem_name=RNA_pseudouridine_syntheses) |
| RNA Metabolism | RNA processing and modification | [RNA pseudouridine syntheses](https://rast.nmpdr.org/seedviewer.cgi?page=Subsystems&subsystem=RNA_pseudouridine_syntheses&organism=549.408) | [Ribosomal large subunit pseudouridine synthase E (EC 4.2.1.70)](https://rast.nmpdr.org/seedviewer.cgi?page=FunctionalRole&role=Ribosomal%20large%20subunit%20pseudouridine%20synthase%20E%20(EC%204.2.1.70)&subsystem_name=RNA_pseudouridine_syntheses) |
| RNA Metabolism | RNA processing and modification | [RNA pseudouridine syntheses](https://rast.nmpdr.org/seedviewer.cgi?page=Subsystems&subsystem=RNA_pseudouridine_syntheses&organism=549.408) | [Ribosomal large subunit pseudouridine synthase D (EC 4.2.1.70)](https://rast.nmpdr.org/seedviewer.cgi?page=FunctionalRole&role=Ribosomal%20large%20subunit%20pseudouridine%20synthase%20D%20(EC%204.2.1.70)&subsystem_name=RNA_pseudouridine_syntheses) |
| RNA Metabolism | RNA processing and modification | [RNA pseudouridine syntheses](https://rast.nmpdr.org/seedviewer.cgi?page=Subsystems&subsystem=RNA_pseudouridine_syntheses&organism=549.408) | [Ribosomal large subunit pseudouridine synthase C (EC 4.2.1.70)](https://rast.nmpdr.org/seedviewer.cgi?page=FunctionalRole&role=Ribosomal%20large%20subunit%20pseudouridine%20synthase%20C%20(EC%204.2.1.70)&subsystem_name=RNA_pseudouridine_syntheses) |
| RNA Metabolism | RNA processing and modification | [RNA pseudouridine syntheses](https://rast.nmpdr.org/seedviewer.cgi?page=Subsystems&subsystem=RNA_pseudouridine_syntheses&organism=549.408) | [Ribosomal large subunit pseudouridine synthase B (EC 4.2.1.70)](https://rast.nmpdr.org/seedviewer.cgi?page=FunctionalRole&role=Ribosomal%20large%20subunit%20pseudouridine%20synthase%20B%20(EC%204.2.1.70)&subsystem_name=RNA_pseudouridine_syntheses) |
| RNA Metabolism | RNA processing and modification | [RNA pseudouridine syntheses](https://rast.nmpdr.org/seedviewer.cgi?page=Subsystems&subsystem=RNA_pseudouridine_syntheses&organism=549.408) | [tRNA pseudouridine synthase B (EC 4.2.1.70)](https://rast.nmpdr.org/seedviewer.cgi?page=FunctionalRole&role=tRNA%20pseudouridine%20synthase%20B%20(EC%204.2.1.70)&subsystem_name=RNA_pseudouridine_syntheses) |
| RNA Metabolism | RNA processing and modification | [RNA pseudouridine syntheses](https://rast.nmpdr.org/seedviewer.cgi?page=Subsystems&subsystem=RNA_pseudouridine_syntheses&organism=549.408) | [Ribosomal small subunit pseudouridine synthase A (EC 4.2.1.70)](https://rast.nmpdr.org/seedviewer.cgi?page=FunctionalRole&role=Ribosomal%20small%20subunit%20pseudouridine%20synthase%20A%20(EC%204.2.1.70)&subsystem_name=RNA_pseudouridine_syntheses) |
| RNA Metabolism | RNA processing and modification | [RNA pseudouridine syntheses](https://rast.nmpdr.org/seedviewer.cgi?page=Subsystems&subsystem=RNA_pseudouridine_syntheses&organism=549.408) | [tRNA pseudouridine synthase C (EC 4.2.1.70)](https://rast.nmpdr.org/seedviewer.cgi?page=FunctionalRole&role=tRNA%20pseudouridine%20synthase%20C%20(EC%204.2.1.70)&subsystem_name=RNA_pseudouridine_syntheses) |
| RNA Metabolism | RNA processing and modification | [RNA pseudouridine syntheses](https://rast.nmpdr.org/seedviewer.cgi?page=Subsystems&subsystem=RNA_pseudouridine_syntheses&organism=549.408) | [tRNA pseudouridine synthase A (EC 4.2.1.70)](https://rast.nmpdr.org/seedviewer.cgi?page=FunctionalRole&role=tRNA%20pseudouridine%20synthase%20A%20(EC%204.2.1.70)&subsystem_name=RNA_pseudouridine_syntheses) |
| RNA Metabolism | RNA processing and modification | [Polyadenylation bacterial](https://rast.nmpdr.org/seedviewer.cgi?page=Subsystems&subsystem=Polyadenylation_bacterial&organism=549.408) | [tRNA nucleotidyltransferase (EC 2.7.7.21) (EC 2.7.7.25)](https://rast.nmpdr.org/seedviewer.cgi?page=FunctionalRole&role=tRNA%20nucleotidyltransferase%20(EC%202.7.7.21)%20(EC%202.7.7.25)&subsystem_name=Polyadenylation_bacterial) |
| RNA Metabolism | RNA processing and modification | [Polyadenylation bacterial](https://rast.nmpdr.org/seedviewer.cgi?page=Subsystems&subsystem=Polyadenylation_bacterial&organism=549.408) | [Polyribonucleotide nucleotidyltransferase (EC 2.7.7.8)](https://rast.nmpdr.org/seedviewer.cgi?page=FunctionalRole&role=Polyribonucleotide%20nucleotidyltransferase%20(EC%202.7.7.8)&subsystem_name=Polyadenylation_bacterial) |
| RNA Metabolism | RNA processing and modification | [Polyadenylation bacterial](https://rast.nmpdr.org/seedviewer.cgi?page=Subsystems&subsystem=Polyadenylation_bacterial&organism=549.408) | [Poly(A) polymerase (EC 2.7.7.19)](https://rast.nmpdr.org/seedviewer.cgi?page=FunctionalRole&role=Poly(A)%20polymerase%20(EC%202.7.7.19)&subsystem_name=Polyadenylation_bacterial) |
| RNA Metabolism | RNA processing and modification | [Polyadenylation bacterial](https://rast.nmpdr.org/seedviewer.cgi?page=Subsystems&subsystem=Polyadenylation_bacterial&organism=549.408) | [RNA-binding protein Hfq](https://rast.nmpdr.org/seedviewer.cgi?page=FunctionalRole&role=RNA-binding%20protein%20Hfq&subsystem_name=Polyadenylation_bacterial) |
| RNA Metabolism | RNA processing and modification | [Possible RNA modification and stress response cluster](https://rast.nmpdr.org/seedviewer.cgi?page=Subsystems&subsystem=Possible_RNA_modification_and_stress_response_cluster&organism=549.408) | [Organic solvent tolerance protein precursor](https://rast.nmpdr.org/seedviewer.cgi?page=FunctionalRole&role=Organic%20solvent%20tolerance%20protein%20precursor&subsystem_name=Possible_RNA_modification_and_stress_response_cluster) |
| RNA Metabolism | RNA processing and modification | [Possible RNA modification and stress response cluster](https://rast.nmpdr.org/seedviewer.cgi?page=Subsystems&subsystem=Possible_RNA_modification_and_stress_response_cluster&organism=549.408) | [Bis(5'-nucleosyl)-tetraphosphatase, symmetrical (EC 3.6.1.41)](https://rast.nmpdr.org/seedviewer.cgi?page=FunctionalRole&role=Bis(5) |
| RNA Metabolism | RNA processing and modification | [Possible RNA modification and stress response cluster](https://rast.nmpdr.org/seedviewer.cgi?page=Subsystems&subsystem=Possible_RNA_modification_and_stress_response_cluster&organism=549.408) | [4-hydroxythreonine-4-phosphate dehydrogenase (EC 1.1.1.262)](https://rast.nmpdr.org/seedviewer.cgi?page=FunctionalRole&role=4-hydroxythreonine-4-phosphate%20dehydrogenase%20(EC%201.1.1.262)&subsystem_name=Possible_RNA_modification_and_stress_response_cluster) |
| RNA Metabolism | RNA processing and modification | [Possible RNA modification and stress response cluster](https://rast.nmpdr.org/seedviewer.cgi?page=Subsystems&subsystem=Possible_RNA_modification_and_stress_response_cluster&organism=549.408) | [Survival protein SurA precursor (Peptidyl-prolyl cis-trans isomerase SurA) (EC 5.2.1.8)](https://rast.nmpdr.org/seedviewer.cgi?page=FunctionalRole&role=Survival%20protein%20SurA%20precursor%20(Peptidyl-prolyl%20cis-trans%20isomerase%20SurA)%20(EC%205.2.1.8)&subsystem_name=Possible_RNA_modification_and_stress_response_cluster) |
| RNA Metabolism | RNA processing and modification | [Possible RNA modification and stress response cluster](https://rast.nmpdr.org/seedviewer.cgi?page=Subsystems&subsystem=Possible_RNA_modification_and_stress_response_cluster&organism=549.408) | [TsaB protein, required for threonylcarbamoyladenosine (t(6)A) formation in tRNA](https://rast.nmpdr.org/seedviewer.cgi?page=FunctionalRole&role=TsaB%20protein,%20required%20for%20threonylcarbamoyladenosine%20(t(6)A)%20formation%20in%20tRNA&subsystem_name=Possible_RNA_modification_and_stress_response_cluster) |
| RNA Metabolism | RNA processing and modification | [Possible RNA modification and stress response cluster](https://rast.nmpdr.org/seedviewer.cgi?page=Subsystems&subsystem=Possible_RNA_modification_and_stress_response_cluster&organism=549.408) | [SSU rRNA (adenine(1518)-N(6)/adenine(1519)-N(6))-dimethyltransferase (EC 2.1.1.182)](https://rast.nmpdr.org/seedviewer.cgi?page=FunctionalRole&role=SSU%20rRNA%20(adenine(1518)-N(6)/adenine(1519)-N(6))-dimethyltransferase%20(EC%202.1.1.182)&subsystem_name=Possible_RNA_modification_and_stress_response_cluster) |
| RNA Metabolism | RNA processing and modification | [Possible RNA modification and stress response cluster](https://rast.nmpdr.org/seedviewer.cgi?page=Subsystems&subsystem=Possible_RNA_modification_and_stress_response_cluster&organism=549.408) | [TsaD/Kae1/Qri7 protein, required for threonylcarbamoyladenosine t(6)A37 formation in tRNA](https://rast.nmpdr.org/seedviewer.cgi?page=FunctionalRole&role=TsaD/Kae1/Qri7%20protein,%20required%20for%20threonylcarbamoyladenosine%20t(6)A37%20formation%20in%20tRNA&subsystem_name=Possible_RNA_modification_and_stress_response_cluster) |
| RNA Metabolism | RNA processing and modification | [Possible RNA modification and stress response cluster](https://rast.nmpdr.org/seedviewer.cgi?page=Subsystems&subsystem=Possible_RNA_modification_and_stress_response_cluster&organism=549.408) | [DnaJ-like protein DjlA](https://rast.nmpdr.org/seedviewer.cgi?page=FunctionalRole&role=DnaJ-like%20protein%20DjlA&subsystem_name=Possible_RNA_modification_and_stress_response_cluster) |
| RNA Metabolism | RNA processing and modification | [Possible RNA modification and stress response cluster](https://rast.nmpdr.org/seedviewer.cgi?page=Subsystems&subsystem=Possible_RNA_modification_and_stress_response_cluster&organism=549.408) | [ApaG protein](https://rast.nmpdr.org/seedviewer.cgi?page=FunctionalRole&role=ApaG%20protein&subsystem_name=Possible_RNA_modification_and_stress_response_cluster) |
| RNA Metabolism | RNA processing and modification | [Possible RNA modification and stress response cluster](https://rast.nmpdr.org/seedviewer.cgi?page=Subsystems&subsystem=Possible_RNA_modification_and_stress_response_cluster&organism=549.408) | [Ribosomal-protein-S18p-alanine acetyltransferase (EC 2.3.1.-)](https://rast.nmpdr.org/seedviewer.cgi?page=FunctionalRole&role=Ribosomal-protein-S18p-alanine%20acetyltransferase%20(EC%202.3.1.-)&subsystem_name=Possible_RNA_modification_and_stress_response_cluster) |
| RNA Metabolism | RNA processing and modification | [Possible RNA modification and stress response cluster](https://rast.nmpdr.org/seedviewer.cgi?page=Subsystems&subsystem=Possible_RNA_modification_and_stress_response_cluster&organism=549.408) | [Outer membrane protein Imp, required for envelope biogenesis](https://rast.nmpdr.org/seedviewer.cgi?page=FunctionalRole&role=Outer%20membrane%20protein%20Imp,%20required%20for%20envelope%20biogenesis&subsystem_name=Possible_RNA_modification_and_stress_response_cluster) |
| RNA Metabolism | RNA processing and modification | [Possible RNA modification and stress response cluster](https://rast.nmpdr.org/seedviewer.cgi?page=Subsystems&subsystem=Possible_RNA_modification_and_stress_response_cluster&organism=549.408) | [TsaE protein, required for threonylcarbamoyladenosine t(6)A37 formation in tRNA](https://rast.nmpdr.org/seedviewer.cgi?page=FunctionalRole&role=TsaE%20protein,%20required%20for%20threonylcarbamoyladenosine%20t(6)A37%20formation%20in%20tRNA&subsystem_name=Possible_RNA_modification_and_stress_response_cluster) |
| RNA Metabolism | RNA processing and modification | [tRNA nucleotidyltransferase](https://rast.nmpdr.org/seedviewer.cgi?page=Subsystems&subsystem=tRNA_nucleotidyltransferase&organism=549.408) | [tRNA nucleotidyltransferase (EC 2.7.7.21) (EC 2.7.7.25)](https://rast.nmpdr.org/seedviewer.cgi?page=FunctionalRole&role=tRNA%20nucleotidyltransferase%20(EC%202.7.7.21)%20(EC%202.7.7.25)&subsystem_name=tRNA_nucleotidyltransferase) |
| RNA Metabolism | RNA processing and modification | [Methylthiotransferases](https://rast.nmpdr.org/seedviewer.cgi?page=Subsystems&subsystem=Methylthiotransferases&organism=549.408) | [tRNA-i(6)A37 methylthiotransferase](https://rast.nmpdr.org/seedviewer.cgi?page=FunctionalRole&role=tRNA-i(6)A37%20methylthiotransferase&subsystem_name=Methylthiotransferases) |
| RNA Metabolism | RNA processing and modification | [RNA processing and degradation, bacterial](https://rast.nmpdr.org/seedviewer.cgi?page=Subsystems&subsystem=RNA_processing_and_degradation,_bacterial&organism=549.408) | [3'-to-5' exoribonuclease RNase R](https://rast.nmpdr.org/seedviewer.cgi?page=FunctionalRole&role=3) |
| RNA Metabolism | RNA processing and modification | [RNA processing and degradation, bacterial](https://rast.nmpdr.org/seedviewer.cgi?page=Subsystems&subsystem=RNA_processing_and_degradation,_bacterial&organism=549.408) | [Exoribonuclease II (EC 3.1.13.1)](https://rast.nmpdr.org/seedviewer.cgi?page=FunctionalRole&role=Exoribonuclease%20II%20(EC%203.1.13.1)&subsystem_name=RNA_processing_and_degradation,_bacterial) |
| RNA Metabolism | RNA processing and modification | [RNA processing and degradation, bacterial](https://rast.nmpdr.org/seedviewer.cgi?page=Subsystems&subsystem=RNA_processing_and_degradation,_bacterial&organism=549.408) | [Ribonuclease E inhibitor RraA](https://rast.nmpdr.org/seedviewer.cgi?page=FunctionalRole&role=Ribonuclease%20E%20inhibitor%20RraA&subsystem_name=RNA_processing_and_degradation,_bacterial) |
| RNA Metabolism | RNA processing and modification | [RNA processing and degradation, bacterial](https://rast.nmpdr.org/seedviewer.cgi?page=Subsystems&subsystem=RNA_processing_and_degradation,_bacterial&organism=549.408) | [3'-to-5' oligoribonuclease (orn)](https://rast.nmpdr.org/seedviewer.cgi?page=FunctionalRole&role=3) |
| RNA Metabolism | RNA processing and modification | [RNA processing and degradation, bacterial](https://rast.nmpdr.org/seedviewer.cgi?page=Subsystems&subsystem=RNA_processing_and_degradation,_bacterial&organism=549.408) | [Cytoplasmic axial filament protein CafA and Ribonuclease G (EC 3.1.4.-)](https://rast.nmpdr.org/seedviewer.cgi?page=FunctionalRole&role=Cytoplasmic%20axial%20filament%20protein%20CafA%20and%20Ribonuclease%20G%20(EC%203.1.4.-)&subsystem_name=RNA_processing_and_degradation,_bacterial) |
| RNA Metabolism | RNA processing and modification | [RNA processing and degradation, bacterial](https://rast.nmpdr.org/seedviewer.cgi?page=Subsystems&subsystem=RNA_processing_and_degradation,_bacterial&organism=549.408) | [Ribonuclease E inhibitor RraB](https://rast.nmpdr.org/seedviewer.cgi?page=FunctionalRole&role=Ribonuclease%20E%20inhibitor%20RraB&subsystem_name=RNA_processing_and_degradation,_bacterial) |
| RNA Metabolism | RNA processing and modification | [RNA processing and degradation, bacterial](https://rast.nmpdr.org/seedviewer.cgi?page=Subsystems&subsystem=RNA_processing_and_degradation,_bacterial&organism=549.408) | [Ribonuclease III (EC 3.1.26.3)](https://rast.nmpdr.org/seedviewer.cgi?page=FunctionalRole&role=Ribonuclease%20III%20(EC%203.1.26.3)&subsystem_name=RNA_processing_and_degradation,_bacterial) |
| RNA Metabolism | RNA processing and modification | [RNA processing and degradation, bacterial](https://rast.nmpdr.org/seedviewer.cgi?page=Subsystems&subsystem=RNA_processing_and_degradation,_bacterial&organism=549.408) | [Ribonuclease E (EC 3.1.26.12)](https://rast.nmpdr.org/seedviewer.cgi?page=FunctionalRole&role=Ribonuclease%20E%20(EC%203.1.26.12)&subsystem_name=RNA_processing_and_degradation,_bacterial) |
| RNA Metabolism | RNA processing and modification | [RNA methylation](https://rast.nmpdr.org/seedviewer.cgi?page=Subsystems&subsystem=RNA_methylation&organism=549.408) | [tRNA:Cm32/Um32 methyltransferase](https://rast.nmpdr.org/seedviewer.cgi?page=FunctionalRole&role=tRNA:Cm32/Um32%20methyltransferase&subsystem_name=RNA_methylation) |
| RNA Metabolism | RNA processing and modification | [RNA methylation](https://rast.nmpdr.org/seedviewer.cgi?page=Subsystems&subsystem=RNA_methylation&organism=549.408) | [tRNA (guanosine(18)-2'-O)-methyltransferase (EC 2.1.1.34)](https://rast.nmpdr.org/seedviewer.cgi?page=FunctionalRole&role=tRNA%20(guanosine(18)-2) |
| RNA Metabolism | RNA processing and modification | [RNA methylation](https://rast.nmpdr.org/seedviewer.cgi?page=Subsystems&subsystem=RNA_methylation&organism=549.408) | [tRNA (adenine37-N(6))-methyltransferase TrmN6 (EC 2.1.1.223)](https://rast.nmpdr.org/seedviewer.cgi?page=FunctionalRole&role=tRNA%20(adenine37-N(6))-methyltransferase%20TrmN6%20(EC%202.1.1.223)&subsystem_name=RNA_methylation) |
| RNA Metabolism | RNA processing and modification | [RNA methylation](https://rast.nmpdr.org/seedviewer.cgi?page=Subsystems&subsystem=RNA_methylation&organism=549.408) | [Ribosomal RNA small subunit methyltransferase E (EC 2.1.1.-)](https://rast.nmpdr.org/seedviewer.cgi?page=FunctionalRole&role=Ribosomal%20RNA%20small%20subunit%20methyltransferase%20E%20(EC%202.1.1.-)&subsystem_name=RNA_methylation) |
| RNA Metabolism | RNA processing and modification | [RNA methylation](https://rast.nmpdr.org/seedviewer.cgi?page=Subsystems&subsystem=RNA_methylation&organism=549.408) | [Ribosomal RNA large subunit methyltransferase F (EC 2.1.1.51)](https://rast.nmpdr.org/seedviewer.cgi?page=FunctionalRole&role=Ribosomal%20RNA%20large%20subunit%20methyltransferase%20F%20(EC%202.1.1.51)&subsystem_name=RNA_methylation) |
| RNA Metabolism | RNA processing and modification | [RNA methylation](https://rast.nmpdr.org/seedviewer.cgi?page=Subsystems&subsystem=RNA_methylation&organism=549.408) | [Ribosomal RNA large subunit methyltransferase E (EC 2.1.1.-)](https://rast.nmpdr.org/seedviewer.cgi?page=FunctionalRole&role=Ribosomal%20RNA%20large%20subunit%20methyltransferase%20E%20(EC%202.1.1.-)&subsystem_name=RNA_methylation) |
| RNA Metabolism | RNA processing and modification | [RNA methylation](https://rast.nmpdr.org/seedviewer.cgi?page=Subsystems&subsystem=RNA_methylation&organism=549.408) | [tRNA (guanine46-N7-)-methyltransferase (EC 2.1.1.33)](https://rast.nmpdr.org/seedviewer.cgi?page=FunctionalRole&role=tRNA%20(guanine46-N7-)-methyltransferase%20(EC%202.1.1.33)&subsystem_name=RNA_methylation) |
| RNA Metabolism | RNA processing and modification | [RNA methylation](https://rast.nmpdr.org/seedviewer.cgi?page=Subsystems&subsystem=RNA_methylation&organism=549.408) | [hypothetical tRNA/rRNA methyltransferase yfiF [EC:2.1.1.-]](https://rast.nmpdr.org/seedviewer.cgi?page=FunctionalRole&role=hypothetical%20tRNA/rRNA%20methyltransferase%20yfiF%20%5bEC:2.1.1.-%5d&subsystem_name=RNA_methylation) |
| RNA Metabolism | RNA processing and modification | [RNA methylation](https://rast.nmpdr.org/seedviewer.cgi?page=Subsystems&subsystem=RNA_methylation&organism=549.408) | [5-methylaminomethyl-2-thiouridine-forming enzyme mnmC](https://rast.nmpdr.org/seedviewer.cgi?page=FunctionalRole&role=5-methylaminomethyl-2-thiouridine-forming%20enzyme%20mnmC&subsystem_name=RNA_methylation) |
| RNA Metabolism | RNA processing and modification | [RNA methylation](https://rast.nmpdr.org/seedviewer.cgi?page=Subsystems&subsystem=RNA_methylation&organism=549.408) | [LSU rRNA 2'-O-methyl-C2498 methyltransferase RlmM](https://rast.nmpdr.org/seedviewer.cgi?page=FunctionalRole&role=LSU%20rRNA%202) |
| RNA Metabolism | RNA processing and modification | [RNA methylation](https://rast.nmpdr.org/seedviewer.cgi?page=Subsystems&subsystem=RNA_methylation&organism=549.408) | [tRNA (cytidine(34)-2'-O)-methyltransferase (EC 2.1.1.207)](https://rast.nmpdr.org/seedviewer.cgi?page=FunctionalRole&role=tRNA%20(cytidine(34)-2) |
| RNA Metabolism | RNA processing and modification | [RNA methylation](https://rast.nmpdr.org/seedviewer.cgi?page=Subsystems&subsystem=RNA_methylation&organism=549.408) | [rRNA small subunit 7-methylguanosine (m7G) methyltransferase GidB](https://rast.nmpdr.org/seedviewer.cgi?page=FunctionalRole&role=rRNA%20small%20subunit%207-methylguanosine%20(m7G)%20methyltransferase%20GidB&subsystem_name=RNA_methylation) |
| RNA Metabolism | RNA processing and modification | [RNA methylation](https://rast.nmpdr.org/seedviewer.cgi?page=Subsystems&subsystem=RNA_methylation&organism=549.408) | [LSU m3Psi1915 methyltransferase RlmH](https://rast.nmpdr.org/seedviewer.cgi?page=FunctionalRole&role=LSU%20m3Psi1915%20methyltransferase%20RlmH&subsystem_name=RNA_methylation) |
| RNA Metabolism | RNA processing and modification | [RNA methylation](https://rast.nmpdr.org/seedviewer.cgi?page=Subsystems&subsystem=RNA_methylation&organism=549.408) | [23S rRNA (Uracil-5-) -methyltransferase rumB (EC 2.1.1.-)](https://rast.nmpdr.org/seedviewer.cgi?page=FunctionalRole&role=23S%20rRNA%20(Uracil-5-)%20-methyltransferase%20rumB%20(EC%202.1.1.-)&subsystem_name=RNA_methylation) |
| RNA Metabolism | RNA processing and modification | [RNA methylation](https://rast.nmpdr.org/seedviewer.cgi?page=Subsystems&subsystem=RNA_methylation&organism=549.408) | [16S rRNA (guanine(966)-N(2))-methyltransferase (EC 2.1.1.171)](https://rast.nmpdr.org/seedviewer.cgi?page=FunctionalRole&role=16S%20rRNA%20(guanine(966)-N(2))-methyltransferase%20(EC%202.1.1.171)&subsystem_name=RNA_methylation) |
| RNA Metabolism | RNA processing and modification | [RNA methylation](https://rast.nmpdr.org/seedviewer.cgi?page=Subsystems&subsystem=RNA_methylation&organism=549.408) | [Ribosomal RNA large subunit methyltransferase A (EC 2.1.1.51)](https://rast.nmpdr.org/seedviewer.cgi?page=FunctionalRole&role=Ribosomal%20RNA%20large%20subunit%20methyltransferase%20A%20(EC%202.1.1.51)&subsystem_name=RNA_methylation) |
| RNA Metabolism | RNA processing and modification | [RNA methylation](https://rast.nmpdr.org/seedviewer.cgi?page=Subsystems&subsystem=RNA_methylation&organism=549.408) | [23S rRNA (Uracil-5-) -methyltransferase RumA (EC 2.1.1.-)](https://rast.nmpdr.org/seedviewer.cgi?page=FunctionalRole&role=23S%20rRNA%20(Uracil-5-)%20-methyltransferase%20RumA%20(EC%202.1.1.-)&subsystem_name=RNA_methylation) |
| RNA Metabolism | RNA processing and modification | [RNA methylation](https://rast.nmpdr.org/seedviewer.cgi?page=Subsystems&subsystem=RNA_methylation&organism=549.408) | [LSU m5C1962 methyltransferase RlmI](https://rast.nmpdr.org/seedviewer.cgi?page=FunctionalRole&role=LSU%20m5C1962%20methyltransferase%20RlmI&subsystem_name=RNA_methylation) |
| RNA Metabolism | RNA processing and modification | [RNA methylation](https://rast.nmpdr.org/seedviewer.cgi?page=Subsystems&subsystem=RNA_methylation&organism=549.408) | [SSU rRNA (adenine(1518)-N(6)/adenine(1519)-N(6))-dimethyltransferase (EC 2.1.1.182)](https://rast.nmpdr.org/seedviewer.cgi?page=FunctionalRole&role=SSU%20rRNA%20(adenine(1518)-N(6)/adenine(1519)-N(6))-dimethyltransferase%20(EC%202.1.1.182)&subsystem_name=RNA_methylation) |
| RNA Metabolism | RNA processing and modification | [RNA methylation](https://rast.nmpdr.org/seedviewer.cgi?page=Subsystems&subsystem=RNA_methylation&organism=549.408) | [23S rRNA (guanosine-2'-O-) -methyltransferase rlmB (EC 2.1.1.-)](https://rast.nmpdr.org/seedviewer.cgi?page=FunctionalRole&role=23S%20rRNA%20(guanosine-2) |
| RNA Metabolism | RNA processing and modification | [RNA methylation](https://rast.nmpdr.org/seedviewer.cgi?page=Subsystems&subsystem=RNA_methylation&organism=549.408) | [Ribosomal RNA large subunit methyltransferase N (EC 2.1.1.-)](https://rast.nmpdr.org/seedviewer.cgi?page=FunctionalRole&role=Ribosomal%20RNA%20large%20subunit%20methyltransferase%20N%20(EC%202.1.1.-)&subsystem_name=RNA_methylation) |
| RNA Metabolism | RNA processing and modification | [RNA methylation](https://rast.nmpdr.org/seedviewer.cgi?page=Subsystems&subsystem=RNA_methylation&organism=549.408) | [tRNA (Guanine37-N1) -methyltransferase (EC 2.1.1.31)](https://rast.nmpdr.org/seedviewer.cgi?page=FunctionalRole&role=tRNA%20(Guanine37-N1)%20-methyltransferase%20(EC%202.1.1.31)&subsystem_name=RNA_methylation) |
| RNA Metabolism | RNA processing and modification | [RNA methylation](https://rast.nmpdr.org/seedviewer.cgi?page=Subsystems&subsystem=RNA_methylation&organism=549.408) | [23S rRNA (guanine-N-2-) -methyltransferase rlmL EC 2.1.1.-)](https://rast.nmpdr.org/seedviewer.cgi?page=FunctionalRole&role=23S%20rRNA%20(guanine-N-2-)%20-methyltransferase%20rlmL%20EC%202.1.1.-)&subsystem_name=RNA_methylation) |
| RNA Metabolism | RNA processing and modification | [RNA methylation](https://rast.nmpdr.org/seedviewer.cgi?page=Subsystems&subsystem=RNA_methylation&organism=549.408) | [23S rRNA (guanine-N-2-) -methyltransferase rlmG (EC 2.1.1.-)](https://rast.nmpdr.org/seedviewer.cgi?page=FunctionalRole&role=23S%20rRNA%20(guanine-N-2-)%20-methyltransferase%20rlmG%20(EC%202.1.1.-)&subsystem_name=RNA_methylation) |
| RNA Metabolism | RNA processing and modification | [RNA methylation](https://rast.nmpdr.org/seedviewer.cgi?page=Subsystems&subsystem=RNA_methylation&organism=549.408) | [Ribosomal RNA small subunit methyltransferase F (EC 2.1.1.-)](https://rast.nmpdr.org/seedviewer.cgi?page=FunctionalRole&role=Ribosomal%20RNA%20small%20subunit%20methyltransferase%20F%20(EC%202.1.1.-)&subsystem_name=RNA_methylation) |
| RNA Metabolism | RNA processing and modification | [RNA methylation](https://rast.nmpdr.org/seedviewer.cgi?page=Subsystems&subsystem=RNA_methylation&organism=549.408) | [tRNA-specific 2-thiouridylase MnmA](https://rast.nmpdr.org/seedviewer.cgi?page=FunctionalRole&role=tRNA-specific%202-thiouridylase%20MnmA&subsystem_name=RNA_methylation) |
| RNA Metabolism | RNA processing and modification | [RNA methylation](https://rast.nmpdr.org/seedviewer.cgi?page=Subsystems&subsystem=RNA_methylation&organism=549.408) | [Ribosomal RNA small subunit methyltransferase C (EC 2.1.1.52)](https://rast.nmpdr.org/seedviewer.cgi?page=FunctionalRole&role=Ribosomal%20RNA%20small%20subunit%20methyltransferase%20C%20(EC%202.1.1.52)&subsystem_name=RNA_methylation) |
| RNA Metabolism | RNA processing and modification | [ATP-dependent RNA helicases, bacterial](https://rast.nmpdr.org/seedviewer.cgi?page=Subsystems&subsystem=ATP-dependent_RNA_helicases,_bacterial&organism=549.408) | [ATP-dependent RNA helicase RhlE](https://rast.nmpdr.org/seedviewer.cgi?page=FunctionalRole&role=ATP-dependent%20RNA%20helicase%20RhlE&subsystem_name=ATP-dependent_RNA_helicases,_bacterial) |
| RNA Metabolism | RNA processing and modification | [ATP-dependent RNA helicases, bacterial](https://rast.nmpdr.org/seedviewer.cgi?page=Subsystems&subsystem=ATP-dependent_RNA_helicases,_bacterial&organism=549.408) | [ATP-dependent RNA helicase RhlB](https://rast.nmpdr.org/seedviewer.cgi?page=FunctionalRole&role=ATP-dependent%20RNA%20helicase%20RhlB&subsystem_name=ATP-dependent_RNA_helicases,_bacterial) |
| RNA Metabolism | RNA processing and modification | [ATP-dependent RNA helicases, bacterial](https://rast.nmpdr.org/seedviewer.cgi?page=Subsystems&subsystem=ATP-dependent_RNA_helicases,_bacterial&organism=549.408) | [ATP-dependent 23S rRNA helicase DbpA](https://rast.nmpdr.org/seedviewer.cgi?page=FunctionalRole&role=ATP-dependent%2023S%20rRNA%20helicase%20DbpA&subsystem_name=ATP-dependent_RNA_helicases,_bacterial) |
| RNA Metabolism | RNA processing and modification | [ATP-dependent RNA helicases, bacterial](https://rast.nmpdr.org/seedviewer.cgi?page=Subsystems&subsystem=ATP-dependent_RNA_helicases,_bacterial&organism=549.408) | [ATP-dependent RNA helicase SrmB](https://rast.nmpdr.org/seedviewer.cgi?page=FunctionalRole&role=ATP-dependent%20RNA%20helicase%20SrmB&subsystem_name=ATP-dependent_RNA_helicases,_bacterial) |
| RNA Metabolism | RNA processing and modification | [16S rRNA modification within P site of ribosome](https://rast.nmpdr.org/seedviewer.cgi?page=Subsystems&subsystem=16S_rRNA_modification_within_P_site_of_ribosome&organism=549.408) | [Penicillin-binding protein 2 (PBP-2)](https://rast.nmpdr.org/seedviewer.cgi?page=FunctionalRole&role=Penicillin-binding%20protein%202%20(PBP-2)&subsystem_name=16S_rRNA_modification_within_P_site_of_ribosome) |
| RNA Metabolism | RNA processing and modification | [16S rRNA modification within P site of ribosome](https://rast.nmpdr.org/seedviewer.cgi?page=Subsystems&subsystem=16S_rRNA_modification_within_P_site_of_ribosome&organism=549.408) | [Cell division protein FtsI [Peptidoglycan synthetase] (EC 2.4.1.129)](https://rast.nmpdr.org/seedviewer.cgi?page=FunctionalRole&role=Cell%20division%20protein%20FtsI%20%5bPeptidoglycan%20synthetase%5d%20(EC%202.4.1.129)&subsystem_name=16S_rRNA_modification_within_P_site_of_ribosome) |
| RNA Metabolism | RNA processing and modification | [16S rRNA modification within P site of ribosome](https://rast.nmpdr.org/seedviewer.cgi?page=Subsystems&subsystem=16S_rRNA_modification_within_P_site_of_ribosome&organism=549.408) | [Cell division protein MraZ](https://rast.nmpdr.org/seedviewer.cgi?page=FunctionalRole&role=Cell%20division%20protein%20MraZ&subsystem_name=16S_rRNA_modification_within_P_site_of_ribosome) |
| RNA Metabolism | RNA processing and modification | [16S rRNA modification within P site of ribosome](https://rast.nmpdr.org/seedviewer.cgi?page=Subsystems&subsystem=16S_rRNA_modification_within_P_site_of_ribosome&organism=549.408) | [rRNA small subunit methyltransferase H](https://rast.nmpdr.org/seedviewer.cgi?page=FunctionalRole&role=rRNA%20small%20subunit%20methyltransferase%20H&subsystem_name=16S_rRNA_modification_within_P_site_of_ribosome) |
| RNA Metabolism | RNA processing and modification | [16S rRNA modification within P site of ribosome](https://rast.nmpdr.org/seedviewer.cgi?page=Subsystems&subsystem=16S_rRNA_modification_within_P_site_of_ribosome&organism=549.408) | [rRNA small subunit methyltransferase I](https://rast.nmpdr.org/seedviewer.cgi?page=FunctionalRole&role=rRNA%20small%20subunit%20methyltransferase%20I&subsystem_name=16S_rRNA_modification_within_P_site_of_ribosome) |
| RNA Metabolism | RNA processing and modification | [16S rRNA modification within P site of ribosome](https://rast.nmpdr.org/seedviewer.cgi?page=Subsystems&subsystem=16S_rRNA_modification_within_P_site_of_ribosome&organism=549.408) | [Cell division protein FtsL](https://rast.nmpdr.org/seedviewer.cgi?page=FunctionalRole&role=Cell%20division%20protein%20FtsL&subsystem_name=16S_rRNA_modification_within_P_site_of_ribosome) |
| RNA Metabolism | RNA processing and modification | [tRNA modification Bacteria](https://rast.nmpdr.org/seedviewer.cgi?page=Subsystems&subsystem=tRNA_modification_Bacteria&organism=549.408) | [tRNA (guanosine(18)-2'-O)-methyltransferase (EC 2.1.1.34)](https://rast.nmpdr.org/seedviewer.cgi?page=FunctionalRole&role=tRNA%20(guanosine(18)-2) |
| RNA Metabolism | RNA processing and modification | [tRNA modification Bacteria](https://rast.nmpdr.org/seedviewer.cgi?page=Subsystems&subsystem=tRNA_modification_Bacteria&organism=549.408) | [Ribosomal large subunit pseudouridine synthase A (EC 4.2.1.70)](https://rast.nmpdr.org/seedviewer.cgi?page=FunctionalRole&role=Ribosomal%20large%20subunit%20pseudouridine%20synthase%20A%20(EC%204.2.1.70)&subsystem_name=tRNA_modification_Bacteria) |
| RNA Metabolism | RNA processing and modification | [tRNA modification Bacteria](https://rast.nmpdr.org/seedviewer.cgi?page=Subsystems&subsystem=tRNA_modification_Bacteria&organism=549.408) | [tRNA(Cytosine32)-2-thiocytidine synthetase](https://rast.nmpdr.org/seedviewer.cgi?page=FunctionalRole&role=tRNA(Cytosine32)-2-thiocytidine%20synthetase&subsystem_name=tRNA_modification_Bacteria) |
| RNA Metabolism | RNA processing and modification | [tRNA modification Bacteria](https://rast.nmpdr.org/seedviewer.cgi?page=Subsystems&subsystem=tRNA_modification_Bacteria&organism=549.408) | [tRNA(Ile)-lysidine synthetase (EC 6.3.4.19)](https://rast.nmpdr.org/seedviewer.cgi?page=FunctionalRole&role=tRNA(Ile)-lysidine%20synthetase%20(EC%206.3.4.19)&subsystem_name=tRNA_modification_Bacteria) |
| RNA Metabolism | RNA processing and modification | [tRNA modification Bacteria](https://rast.nmpdr.org/seedviewer.cgi?page=Subsystems&subsystem=tRNA_modification_Bacteria&organism=549.408) | [tRNA 5-methylaminomethyl-2-thiouridine synthase TusD](https://rast.nmpdr.org/seedviewer.cgi?page=FunctionalRole&role=tRNA%205-methylaminomethyl-2-thiouridine%20synthase%20TusD&subsystem_name=tRNA_modification_Bacteria) |
| RNA Metabolism | RNA processing and modification | [tRNA modification Bacteria](https://rast.nmpdr.org/seedviewer.cgi?page=Subsystems&subsystem=tRNA_modification_Bacteria&organism=549.408) | [tRNA 5-methylaminomethyl-2-thiouridine synthase TusA](https://rast.nmpdr.org/seedviewer.cgi?page=FunctionalRole&role=tRNA%205-methylaminomethyl-2-thiouridine%20synthase%20TusA&subsystem_name=tRNA_modification_Bacteria) |
| RNA Metabolism | RNA processing and modification | [tRNA modification Bacteria](https://rast.nmpdr.org/seedviewer.cgi?page=Subsystems&subsystem=tRNA_modification_Bacteria&organism=549.408) | [Iron-sulfur cluster assembly ATPase protein SufC](https://rast.nmpdr.org/seedviewer.cgi?page=FunctionalRole&role=Iron-sulfur%20cluster%20assembly%20ATPase%20protein%20SufC&subsystem_name=tRNA_modification_Bacteria) |
| RNA Metabolism | RNA processing and modification | [tRNA modification Bacteria](https://rast.nmpdr.org/seedviewer.cgi?page=Subsystems&subsystem=tRNA_modification_Bacteria&organism=549.408) | [tRNA 5-methylaminomethyl-2-thiouridine synthase TusC](https://rast.nmpdr.org/seedviewer.cgi?page=FunctionalRole&role=tRNA%205-methylaminomethyl-2-thiouridine%20synthase%20TusC&subsystem_name=tRNA_modification_Bacteria) |
| RNA Metabolism | RNA processing and modification | [tRNA modification Bacteria](https://rast.nmpdr.org/seedviewer.cgi?page=Subsystems&subsystem=tRNA_modification_Bacteria&organism=549.408) | [tRNA (guanine46-N7-)-methyltransferase (EC 2.1.1.33)](https://rast.nmpdr.org/seedviewer.cgi?page=FunctionalRole&role=tRNA%20(guanine46-N7-)-methyltransferase%20(EC%202.1.1.33)&subsystem_name=tRNA_modification_Bacteria) |
| RNA Metabolism | RNA processing and modification | [tRNA modification Bacteria](https://rast.nmpdr.org/seedviewer.cgi?page=Subsystems&subsystem=tRNA_modification_Bacteria&organism=549.408) | [tRNA-(ms[2]io[6]A)-hydroxylase (EC 1.-.-.-)](https://rast.nmpdr.org/seedviewer.cgi?page=FunctionalRole&role=tRNA-(ms%5b2%5dio%5b6%5dA)-hydroxylase%20(EC%201.-.-.-)&subsystem_name=tRNA_modification_Bacteria) |
| RNA Metabolism | RNA processing and modification | [tRNA modification Bacteria](https://rast.nmpdr.org/seedviewer.cgi?page=Subsystems&subsystem=tRNA_modification_Bacteria&organism=549.408) | [5-methylaminomethyl-2-thiouridine-forming enzyme mnmC](https://rast.nmpdr.org/seedviewer.cgi?page=FunctionalRole&role=5-methylaminomethyl-2-thiouridine-forming%20enzyme%20mnmC&subsystem_name=tRNA_modification_Bacteria) |
| RNA Metabolism | RNA processing and modification | [tRNA modification Bacteria](https://rast.nmpdr.org/seedviewer.cgi?page=Subsystems&subsystem=tRNA_modification_Bacteria&organism=549.408) | [FIG004453: protein YceG like](https://rast.nmpdr.org/seedviewer.cgi?page=FunctionalRole&role=FIG004453:%20protein%20YceG%20like&subsystem_name=tRNA_modification_Bacteria) |
| RNA Metabolism | RNA processing and modification | [tRNA modification Bacteria](https://rast.nmpdr.org/seedviewer.cgi?page=Subsystems&subsystem=tRNA_modification_Bacteria&organism=549.408) | [tRNA-dihydrouridine synthase C (EC 1.-.-.-)](https://rast.nmpdr.org/seedviewer.cgi?page=FunctionalRole&role=tRNA-dihydrouridine%20synthase%20C%20(EC%201.-.-.-)&subsystem_name=tRNA_modification_Bacteria) |
| RNA Metabolism | RNA processing and modification | [tRNA modification Bacteria](https://rast.nmpdr.org/seedviewer.cgi?page=Subsystems&subsystem=tRNA_modification_Bacteria&organism=549.408) | [Queuosine Biosynthesis QueE Radical SAM](https://rast.nmpdr.org/seedviewer.cgi?page=FunctionalRole&role=Queuosine%20Biosynthesis%20QueE%20Radical%20SAM&subsystem_name=tRNA_modification_Bacteria) |
| RNA Metabolism | RNA processing and modification | [tRNA modification Bacteria](https://rast.nmpdr.org/seedviewer.cgi?page=Subsystems&subsystem=tRNA_modification_Bacteria&organism=549.408) | [GTPase and tRNA-U34 5-formylation enzyme TrmE](https://rast.nmpdr.org/seedviewer.cgi?page=FunctionalRole&role=GTPase%20and%20tRNA-U34%205-formylation%20enzyme%20TrmE&subsystem_name=tRNA_modification_Bacteria) |
| RNA Metabolism | RNA processing and modification | [tRNA modification Bacteria](https://rast.nmpdr.org/seedviewer.cgi?page=Subsystems&subsystem=tRNA_modification_Bacteria&organism=549.408) | [tRNA dihydrouridine synthase B (EC 1.-.-.-)](https://rast.nmpdr.org/seedviewer.cgi?page=FunctionalRole&role=tRNA%20dihydrouridine%20synthase%20B%20(EC%201.-.-.-)&subsystem_name=tRNA_modification_Bacteria) |
| RNA Metabolism | RNA processing and modification | [tRNA modification Bacteria](https://rast.nmpdr.org/seedviewer.cgi?page=Subsystems&subsystem=tRNA_modification_Bacteria&organism=549.408) | [Cytidine deaminase (EC 3.5.4.5)](https://rast.nmpdr.org/seedviewer.cgi?page=FunctionalRole&role=Cytidine%20deaminase%20(EC%203.5.4.5)&subsystem_name=tRNA_modification_Bacteria) |
| RNA Metabolism | RNA processing and modification | [tRNA modification Bacteria](https://rast.nmpdr.org/seedviewer.cgi?page=Subsystems&subsystem=tRNA_modification_Bacteria&organism=549.408) | [tRNA dimethylallyltransferase (EC 2.5.1.75)](https://rast.nmpdr.org/seedviewer.cgi?page=FunctionalRole&role=tRNA%20dimethylallyltransferase%20(EC%202.5.1.75)&subsystem_name=tRNA_modification_Bacteria) |
| RNA Metabolism | RNA processing and modification | [tRNA modification Bacteria](https://rast.nmpdr.org/seedviewer.cgi?page=Subsystems&subsystem=tRNA_modification_Bacteria&organism=549.408) | [glutamyl-Q-tRNA synthetase](https://rast.nmpdr.org/seedviewer.cgi?page=FunctionalRole&role=glutamyl-Q-tRNA%20synthetase&subsystem_name=tRNA_modification_Bacteria) |
| RNA Metabolism | RNA processing and modification | [tRNA modification Bacteria](https://rast.nmpdr.org/seedviewer.cgi?page=Subsystems&subsystem=tRNA_modification_Bacteria&organism=549.408) | [COG0613, Predicted metal-dependent phosphoesterases (PHP family)](https://rast.nmpdr.org/seedviewer.cgi?page=FunctionalRole&role=COG0613,%20Predicted%20metal-dependent%20phosphoesterases%20(PHP%20family)&subsystem_name=tRNA_modification_Bacteria) |
| RNA Metabolism | RNA processing and modification | [tRNA modification Bacteria](https://rast.nmpdr.org/seedviewer.cgi?page=Subsystems&subsystem=tRNA_modification_Bacteria&organism=549.408) | [tRNA pseudouridine synthase A (EC 4.2.1.70)](https://rast.nmpdr.org/seedviewer.cgi?page=FunctionalRole&role=tRNA%20pseudouridine%20synthase%20A%20(EC%204.2.1.70)&subsystem_name=tRNA_modification_Bacteria) |
| RNA Metabolism | RNA processing and modification | [tRNA modification Bacteria](https://rast.nmpdr.org/seedviewer.cgi?page=Subsystems&subsystem=tRNA_modification_Bacteria&organism=549.408) | [tRNA:Cm32/Um32 methyltransferase](https://rast.nmpdr.org/seedviewer.cgi?page=FunctionalRole&role=tRNA:Cm32/Um32%20methyltransferase&subsystem_name=tRNA_modification_Bacteria) |
| RNA Metabolism | RNA processing and modification | [tRNA modification Bacteria](https://rast.nmpdr.org/seedviewer.cgi?page=Subsystems&subsystem=tRNA_modification_Bacteria&organism=549.408) | [S-adenosylmethionine:tRNA ribosyltransferase-isomerase (EC 5.-.-.-)](https://rast.nmpdr.org/seedviewer.cgi?page=FunctionalRole&role=S-adenosylmethionine:tRNA%20ribosyltransferase-isomerase%20(EC%205.-.-.-)&subsystem_name=tRNA_modification_Bacteria) |
| RNA Metabolism | RNA processing and modification | [tRNA modification Bacteria](https://rast.nmpdr.org/seedviewer.cgi?page=Subsystems&subsystem=tRNA_modification_Bacteria&organism=549.408) | [tRNA (5-methoxyuridine) 34 synthase](https://rast.nmpdr.org/seedviewer.cgi?page=FunctionalRole&role=tRNA%20(5-methoxyuridine)%2034%20synthase&subsystem_name=tRNA_modification_Bacteria) |
| RNA Metabolism | RNA processing and modification | [tRNA modification Bacteria](https://rast.nmpdr.org/seedviewer.cgi?page=Subsystems&subsystem=tRNA_modification_Bacteria&organism=549.408) | [Predicted P-loop ATPase fused to an acetyltransferase COG1444](https://rast.nmpdr.org/seedviewer.cgi?page=FunctionalRole&role=Predicted%20P-loop%20ATPase%20fused%20to%20an%20acetyltransferase%20COG1444&subsystem_name=tRNA_modification_Bacteria) |
| RNA Metabolism | RNA processing and modification | [tRNA modification Bacteria](https://rast.nmpdr.org/seedviewer.cgi?page=Subsystems&subsystem=tRNA_modification_Bacteria&organism=549.408) | [tRNA-specific adenosine-34 deaminase (EC 3.5.4.-)](https://rast.nmpdr.org/seedviewer.cgi?page=FunctionalRole&role=tRNA-specific%20adenosine-34%20deaminase%20(EC%203.5.4.-)&subsystem_name=tRNA_modification_Bacteria) |
| RNA Metabolism | RNA processing and modification | [tRNA modification Bacteria](https://rast.nmpdr.org/seedviewer.cgi?page=Subsystems&subsystem=tRNA_modification_Bacteria&organism=549.408) | [GTP cyclohydrolase I (EC 3.5.4.16) type 1](https://rast.nmpdr.org/seedviewer.cgi?page=FunctionalRole&role=GTP%20cyclohydrolase%20I%20(EC%203.5.4.16)%20type%201&subsystem_name=tRNA_modification_Bacteria) |
| RNA Metabolism | RNA processing and modification | [tRNA modification Bacteria](https://rast.nmpdr.org/seedviewer.cgi?page=Subsystems&subsystem=tRNA_modification_Bacteria&organism=549.408) | [Queuosine Biosynthesis QueC ATPase](https://rast.nmpdr.org/seedviewer.cgi?page=FunctionalRole&role=Queuosine%20Biosynthesis%20QueC%20ATPase&subsystem_name=tRNA_modification_Bacteria) |
| RNA Metabolism | RNA processing and modification | [tRNA modification Bacteria](https://rast.nmpdr.org/seedviewer.cgi?page=Subsystems&subsystem=tRNA_modification_Bacteria&organism=549.408) | [tRNA pseudouridine synthase C (EC 4.2.1.70)](https://rast.nmpdr.org/seedviewer.cgi?page=FunctionalRole&role=tRNA%20pseudouridine%20synthase%20C%20(EC%204.2.1.70)&subsystem_name=tRNA_modification_Bacteria) |
| RNA Metabolism | RNA processing and modification | [tRNA modification Bacteria](https://rast.nmpdr.org/seedviewer.cgi?page=Subsystems&subsystem=tRNA_modification_Bacteria&organism=549.408) | [hypothetical tRNA/rRNA methyltransferase yfiF [EC:2.1.1.-]](https://rast.nmpdr.org/seedviewer.cgi?page=FunctionalRole&role=hypothetical%20tRNA/rRNA%20methyltransferase%20yfiF%20%5bEC:2.1.1.-%5d&subsystem_name=tRNA_modification_Bacteria) |
| RNA Metabolism | RNA processing and modification | [tRNA modification Bacteria](https://rast.nmpdr.org/seedviewer.cgi?page=Subsystems&subsystem=tRNA_modification_Bacteria&organism=549.408) | [tRNA 5-methylaminomethyl-2-thiouridine synthase TusB](https://rast.nmpdr.org/seedviewer.cgi?page=FunctionalRole&role=tRNA%205-methylaminomethyl-2-thiouridine%20synthase%20TusB&subsystem_name=tRNA_modification_Bacteria) |
| RNA Metabolism | RNA processing and modification | [tRNA modification Bacteria](https://rast.nmpdr.org/seedviewer.cgi?page=Subsystems&subsystem=tRNA_modification_Bacteria&organism=549.408) | [LSU m3Psi1915 methyltransferase RlmH](https://rast.nmpdr.org/seedviewer.cgi?page=FunctionalRole&role=LSU%20m3Psi1915%20methyltransferase%20RlmH&subsystem_name=tRNA_modification_Bacteria) |
| RNA Metabolism | RNA processing and modification | [tRNA modification Bacteria](https://rast.nmpdr.org/seedviewer.cgi?page=Subsystems&subsystem=tRNA_modification_Bacteria&organism=549.408) | [tRNA-guanine transglycosylase (EC 2.4.2.29)](https://rast.nmpdr.org/seedviewer.cgi?page=FunctionalRole&role=tRNA-guanine%20transglycosylase%20(EC%202.4.2.29)&subsystem_name=tRNA_modification_Bacteria) |
| RNA Metabolism | RNA processing and modification | [tRNA modification Bacteria](https://rast.nmpdr.org/seedviewer.cgi?page=Subsystems&subsystem=tRNA_modification_Bacteria&organism=549.408) | [Queuosine biosynthesis QueD, PTPS-I](https://rast.nmpdr.org/seedviewer.cgi?page=FunctionalRole&role=Queuosine%20biosynthesis%20QueD,%20PTPS-I&subsystem_name=tRNA_modification_Bacteria) |
| RNA Metabolism | RNA processing and modification | [tRNA modification Bacteria](https://rast.nmpdr.org/seedviewer.cgi?page=Subsystems&subsystem=tRNA_modification_Bacteria&organism=549.408) | [tRNA dihydrouridine synthase A (EC 1.-.-.-)](https://rast.nmpdr.org/seedviewer.cgi?page=FunctionalRole&role=tRNA%20dihydrouridine%20synthase%20A%20(EC%201.-.-.-)&subsystem_name=tRNA_modification_Bacteria) |
| RNA Metabolism | RNA processing and modification | [tRNA modification Bacteria](https://rast.nmpdr.org/seedviewer.cgi?page=Subsystems&subsystem=tRNA_modification_Bacteria&organism=549.408) | [tRNA-i(6)A37 methylthiotransferase](https://rast.nmpdr.org/seedviewer.cgi?page=FunctionalRole&role=tRNA-i(6)A37%20methylthiotransferase&subsystem_name=tRNA_modification_Bacteria) |
| RNA Metabolism | RNA processing and modification | [tRNA modification Bacteria](https://rast.nmpdr.org/seedviewer.cgi?page=Subsystems&subsystem=tRNA_modification_Bacteria&organism=549.408) | [tRNA uridine 5-carboxymethylaminomethyl modification enzyme GidA](https://rast.nmpdr.org/seedviewer.cgi?page=FunctionalRole&role=tRNA%20uridine%205-carboxymethylaminomethyl%20modification%20enzyme%20GidA&subsystem_name=tRNA_modification_Bacteria) |
| RNA Metabolism | RNA processing and modification | [tRNA modification Bacteria](https://rast.nmpdr.org/seedviewer.cgi?page=Subsystems&subsystem=tRNA_modification_Bacteria&organism=549.408) | [COG1720: Uncharacterized conserved protein](https://rast.nmpdr.org/seedviewer.cgi?page=FunctionalRole&role=COG1720:%20Uncharacterized%20conserved%20protein&subsystem_name=tRNA_modification_Bacteria) |
| RNA Metabolism | RNA processing and modification | [tRNA modification Bacteria](https://rast.nmpdr.org/seedviewer.cgi?page=Subsystems&subsystem=tRNA_modification_Bacteria&organism=549.408) | [tRNA (uridine-5-oxyacetic acid methyl ester) 34 synthase](https://rast.nmpdr.org/seedviewer.cgi?page=FunctionalRole&role=tRNA%20(uridine-5-oxyacetic%20acid%20methyl%20ester)%2034%20synthase&subsystem_name=tRNA_modification_Bacteria) |
| RNA Metabolism | RNA processing and modification | [tRNA modification Bacteria](https://rast.nmpdr.org/seedviewer.cgi?page=Subsystems&subsystem=tRNA_modification_Bacteria&organism=549.408) | [tRNA pseudouridine 13 synthase (EC 4.2.1.-)](https://rast.nmpdr.org/seedviewer.cgi?page=FunctionalRole&role=tRNA%20pseudouridine%2013%20synthase%20(EC%204.2.1.-)&subsystem_name=tRNA_modification_Bacteria) |
| RNA Metabolism | RNA processing and modification | [tRNA modification Bacteria](https://rast.nmpdr.org/seedviewer.cgi?page=Subsystems&subsystem=tRNA_modification_Bacteria&organism=549.408) | [tRNA pseudouridine synthase B (EC 4.2.1.70)](https://rast.nmpdr.org/seedviewer.cgi?page=FunctionalRole&role=tRNA%20pseudouridine%20synthase%20B%20(EC%204.2.1.70)&subsystem_name=tRNA_modification_Bacteria) |
| RNA Metabolism | RNA processing and modification | [tRNA modification Bacteria](https://rast.nmpdr.org/seedviewer.cgi?page=Subsystems&subsystem=tRNA_modification_Bacteria&organism=549.408) | [Iron-sulfur cluster assembly protein SufD](https://rast.nmpdr.org/seedviewer.cgi?page=FunctionalRole&role=Iron-sulfur%20cluster%20assembly%20protein%20SufD&subsystem_name=tRNA_modification_Bacteria) |
| RNA Metabolism | RNA processing and modification | [tRNA modification Bacteria](https://rast.nmpdr.org/seedviewer.cgi?page=Subsystems&subsystem=tRNA_modification_Bacteria&organism=549.408) | [tRNA (Guanine37-N1) -methyltransferase (EC 2.1.1.31)](https://rast.nmpdr.org/seedviewer.cgi?page=FunctionalRole&role=tRNA%20(Guanine37-N1)%20-methyltransferase%20(EC%202.1.1.31)&subsystem_name=tRNA_modification_Bacteria) |
| RNA Metabolism | RNA processing and modification | [tRNA modification Bacteria](https://rast.nmpdr.org/seedviewer.cgi?page=Subsystems&subsystem=tRNA_modification_Bacteria&organism=549.408) | [Cysteine desulfurase (EC 2.8.1.7), IscS subfamily](https://rast.nmpdr.org/seedviewer.cgi?page=FunctionalRole&role=Cysteine%20desulfurase%20(EC%202.8.1.7),%20IscS%20subfamily&subsystem_name=tRNA_modification_Bacteria) |
| RNA Metabolism | RNA processing and modification | [tRNA modification Bacteria](https://rast.nmpdr.org/seedviewer.cgi?page=Subsystems&subsystem=tRNA_modification_Bacteria&organism=549.408) | [Cysteine desulfurase (EC 2.8.1.7), SufS subfamily](https://rast.nmpdr.org/seedviewer.cgi?page=FunctionalRole&role=Cysteine%20desulfurase%20(EC%202.8.1.7),%20SufS%20subfamily&subsystem_name=tRNA_modification_Bacteria) |
| RNA Metabolism | RNA processing and modification | [tRNA modification Bacteria](https://rast.nmpdr.org/seedviewer.cgi?page=Subsystems&subsystem=tRNA_modification_Bacteria&organism=549.408) | [Iron-sulfur cluster assembly protein SufB](https://rast.nmpdr.org/seedviewer.cgi?page=FunctionalRole&role=Iron-sulfur%20cluster%20assembly%20protein%20SufB&subsystem_name=tRNA_modification_Bacteria) |
| RNA Metabolism | RNA processing and modification | [mnm5U34 biosynthesis bacteria](https://rast.nmpdr.org/seedviewer.cgi?page=Subsystems&subsystem=mnm5U34_biosynthesis_bacteria&organism=549.408) | [tRNA uridine 5-carboxymethylaminomethyl modification enzyme GidA](https://rast.nmpdr.org/seedviewer.cgi?page=FunctionalRole&role=tRNA%20uridine%205-carboxymethylaminomethyl%20modification%20enzyme%20GidA&subsystem_name=mnm5U34_biosynthesis_bacteria) |
| RNA Metabolism | RNA processing and modification | [mnm5U34 biosynthesis bacteria](https://rast.nmpdr.org/seedviewer.cgi?page=Subsystems&subsystem=mnm5U34_biosynthesis_bacteria&organism=549.408) | [Cysteine desulfurase CsdA-CsdE (EC 2.8.1.7), main protein CsdA](https://rast.nmpdr.org/seedviewer.cgi?page=FunctionalRole&role=Cysteine%20desulfurase%20CsdA-CsdE%20(EC%202.8.1.7),%20main%20protein%20CsdA&subsystem_name=mnm5U34_biosynthesis_bacteria) |
| RNA Metabolism | RNA processing and modification | [mnm5U34 biosynthesis bacteria](https://rast.nmpdr.org/seedviewer.cgi?page=Subsystems&subsystem=mnm5U34_biosynthesis_bacteria&organism=549.408) | [tRNA 5-methylaminomethyl-2-thiouridine synthase TusD](https://rast.nmpdr.org/seedviewer.cgi?page=FunctionalRole&role=tRNA%205-methylaminomethyl-2-thiouridine%20synthase%20TusD&subsystem_name=mnm5U34_biosynthesis_bacteria) |
| RNA Metabolism | RNA processing and modification | [mnm5U34 biosynthesis bacteria](https://rast.nmpdr.org/seedviewer.cgi?page=Subsystems&subsystem=mnm5U34_biosynthesis_bacteria&organism=549.408) | [tRNA 5-methylaminomethyl-2-thiouridine synthase TusA](https://rast.nmpdr.org/seedviewer.cgi?page=FunctionalRole&role=tRNA%205-methylaminomethyl-2-thiouridine%20synthase%20TusA&subsystem_name=mnm5U34_biosynthesis_bacteria) |
| RNA Metabolism | RNA processing and modification | [mnm5U34 biosynthesis bacteria](https://rast.nmpdr.org/seedviewer.cgi?page=Subsystems&subsystem=mnm5U34_biosynthesis_bacteria&organism=549.408) | [tRNA 5-methylaminomethyl-2-thiouridine synthase TusC](https://rast.nmpdr.org/seedviewer.cgi?page=FunctionalRole&role=tRNA%205-methylaminomethyl-2-thiouridine%20synthase%20TusC&subsystem_name=mnm5U34_biosynthesis_bacteria) |
| RNA Metabolism | RNA processing and modification | [mnm5U34 biosynthesis bacteria](https://rast.nmpdr.org/seedviewer.cgi?page=Subsystems&subsystem=mnm5U34_biosynthesis_bacteria&organism=549.408) | [GTPase and tRNA-U34 5-formylation enzyme TrmE](https://rast.nmpdr.org/seedviewer.cgi?page=FunctionalRole&role=GTPase%20and%20tRNA-U34%205-formylation%20enzyme%20TrmE&subsystem_name=mnm5U34_biosynthesis_bacteria) |
| RNA Metabolism | RNA processing and modification | [mnm5U34 biosynthesis bacteria](https://rast.nmpdr.org/seedviewer.cgi?page=Subsystems&subsystem=mnm5U34_biosynthesis_bacteria&organism=549.408) | [5-methylaminomethyl-2-thiouridine-forming enzyme mnmC](https://rast.nmpdr.org/seedviewer.cgi?page=FunctionalRole&role=5-methylaminomethyl-2-thiouridine-forming%20enzyme%20mnmC&subsystem_name=mnm5U34_biosynthesis_bacteria) |
| RNA Metabolism | RNA processing and modification | [mnm5U34 biosynthesis bacteria](https://rast.nmpdr.org/seedviewer.cgi?page=Subsystems&subsystem=mnm5U34_biosynthesis_bacteria&organism=549.408) | [Cysteine desulfurase (EC 2.8.1.7), IscS subfamily](https://rast.nmpdr.org/seedviewer.cgi?page=FunctionalRole&role=Cysteine%20desulfurase%20(EC%202.8.1.7),%20IscS%20subfamily&subsystem_name=mnm5U34_biosynthesis_bacteria) |
| RNA Metabolism | RNA processing and modification | [mnm5U34 biosynthesis bacteria](https://rast.nmpdr.org/seedviewer.cgi?page=Subsystems&subsystem=mnm5U34_biosynthesis_bacteria&organism=549.408) | [Cysteine desulfurase (EC 2.8.1.7), SufS subfamily](https://rast.nmpdr.org/seedviewer.cgi?page=FunctionalRole&role=Cysteine%20desulfurase%20(EC%202.8.1.7),%20SufS%20subfamily&subsystem_name=mnm5U34_biosynthesis_bacteria) |
| RNA Metabolism | RNA processing and modification | [mnm5U34 biosynthesis bacteria](https://rast.nmpdr.org/seedviewer.cgi?page=Subsystems&subsystem=mnm5U34_biosynthesis_bacteria&organism=549.408) | [tRNA 5-methylaminomethyl-2-thiouridine synthase TusB](https://rast.nmpdr.org/seedviewer.cgi?page=FunctionalRole&role=tRNA%205-methylaminomethyl-2-thiouridine%20synthase%20TusB&subsystem_name=mnm5U34_biosynthesis_bacteria) |
| RNA Metabolism | RNA processing and modification | [Ribonuclease H](https://rast.nmpdr.org/seedviewer.cgi?page=Subsystems&subsystem=Ribonuclease_H&organism=549.408) | [Ribonuclease HI (EC 3.1.26.4)](https://rast.nmpdr.org/seedviewer.cgi?page=FunctionalRole&role=Ribonuclease%20HI%20(EC%203.1.26.4)&subsystem_name=Ribonuclease_H) |
| RNA Metabolism | RNA processing and modification | [Ribonuclease H](https://rast.nmpdr.org/seedviewer.cgi?page=Subsystems&subsystem=Ribonuclease_H&organism=549.408) | [Ribonuclease HII (EC 3.1.26.4)](https://rast.nmpdr.org/seedviewer.cgi?page=FunctionalRole&role=Ribonuclease%20HII%20(EC%203.1.26.4)&subsystem_name=Ribonuclease_H) |
| RNA Metabolism | RNA processing and modification | [Queuosine-Archaeosine Biosynthesis](https://rast.nmpdr.org/seedviewer.cgi?page=Subsystems&subsystem=Queuosine-Archaeosine_Biosynthesis&organism=549.408) | [Queuosine biosynthesis QueD, PTPS-I](https://rast.nmpdr.org/seedviewer.cgi?page=FunctionalRole&role=Queuosine%20biosynthesis%20QueD,%20PTPS-I&subsystem_name=Queuosine-Archaeosine_Biosynthesis) |
| RNA Metabolism | RNA processing and modification | [Queuosine-Archaeosine Biosynthesis](https://rast.nmpdr.org/seedviewer.cgi?page=Subsystems&subsystem=Queuosine-Archaeosine_Biosynthesis&organism=549.408) | [Queuosine Biosynthesis QueE Radical SAM](https://rast.nmpdr.org/seedviewer.cgi?page=FunctionalRole&role=Queuosine%20Biosynthesis%20QueE%20Radical%20SAM&subsystem_name=Queuosine-Archaeosine_Biosynthesis) |
| RNA Metabolism | RNA processing and modification | [Queuosine-Archaeosine Biosynthesis](https://rast.nmpdr.org/seedviewer.cgi?page=Subsystems&subsystem=Queuosine-Archaeosine_Biosynthesis&organism=549.408) | [Inosine-uridine preferring nucleoside hydrolase (EC 3.2.2.1)](https://rast.nmpdr.org/seedviewer.cgi?page=FunctionalRole&role=Inosine-uridine%20preferring%20nucleoside%20hydrolase%20(EC%203.2.2.1)&subsystem_name=Queuosine-Archaeosine_Biosynthesis) |
| RNA Metabolism | RNA processing and modification | [Queuosine-Archaeosine Biosynthesis](https://rast.nmpdr.org/seedviewer.cgi?page=Subsystems&subsystem=Queuosine-Archaeosine_Biosynthesis&organism=549.408) | [Putative preQ0 transporter](https://rast.nmpdr.org/seedviewer.cgi?page=FunctionalRole&role=Putative%20preQ0%20transporter&subsystem_name=Queuosine-Archaeosine_Biosynthesis) |
| RNA Metabolism | RNA processing and modification | [Queuosine-Archaeosine Biosynthesis](https://rast.nmpdr.org/seedviewer.cgi?page=Subsystems&subsystem=Queuosine-Archaeosine_Biosynthesis&organism=549.408) | [S-adenosylmethionine:tRNA ribosyltransferase-isomerase (EC 5.-.-.-)](https://rast.nmpdr.org/seedviewer.cgi?page=FunctionalRole&role=S-adenosylmethionine:tRNA%20ribosyltransferase-isomerase%20(EC%205.-.-.-)&subsystem_name=Queuosine-Archaeosine_Biosynthesis) |
| RNA Metabolism | RNA processing and modification | [Queuosine-Archaeosine Biosynthesis](https://rast.nmpdr.org/seedviewer.cgi?page=Subsystems&subsystem=Queuosine-Archaeosine_Biosynthesis&organism=549.408) | [Permease of the drug/metabolite transporter (DMT) superfamily](https://rast.nmpdr.org/seedviewer.cgi?page=FunctionalRole&role=Permease%20of%20the%20drug/metabolite%20transporter%20(DMT)%20superfamily&subsystem_name=Queuosine-Archaeosine_Biosynthesis) |
| RNA Metabolism | RNA processing and modification | [Queuosine-Archaeosine Biosynthesis](https://rast.nmpdr.org/seedviewer.cgi?page=Subsystems&subsystem=Queuosine-Archaeosine_Biosynthesis&organism=549.408) | [Radical SAM family enzyme, similar to coproporphyrinogen III oxidase, oxygen-independent, clustered with nucleoside-triphosphatase RdgB](https://rast.nmpdr.org/seedviewer.cgi?page=FunctionalRole&role=Radical%20SAM%20family%20enzyme,%20similar%20to%20coproporphyrinogen%20III%20oxidase,%20oxygen-independent,%20clustered%20with%20nucleoside-triphosphatase%20RdgB&subsystem_name=Queuosine-Archaeosine_Biosynthesis) |
| RNA Metabolism | RNA processing and modification | [Queuosine-Archaeosine Biosynthesis](https://rast.nmpdr.org/seedviewer.cgi?page=Subsystems&subsystem=Queuosine-Archaeosine_Biosynthesis&organism=549.408) | [Dihydroneopterin triphosphate pyrophosphohydolase type 2](https://rast.nmpdr.org/seedviewer.cgi?page=FunctionalRole&role=Dihydroneopterin%20triphosphate%20pyrophosphohydolase%20type%202&subsystem_name=Queuosine-Archaeosine_Biosynthesis) |
| RNA Metabolism | RNA processing and modification | [Queuosine-Archaeosine Biosynthesis](https://rast.nmpdr.org/seedviewer.cgi?page=Subsystems&subsystem=Queuosine-Archaeosine_Biosynthesis&organism=549.408) | [GTP cyclohydrolase I (EC 3.5.4.16) type 1](https://rast.nmpdr.org/seedviewer.cgi?page=FunctionalRole&role=GTP%20cyclohydrolase%20I%20(EC%203.5.4.16)%20type%201&subsystem_name=Queuosine-Archaeosine_Biosynthesis) |
| RNA Metabolism | RNA processing and modification | [Queuosine-Archaeosine Biosynthesis](https://rast.nmpdr.org/seedviewer.cgi?page=Subsystems&subsystem=Queuosine-Archaeosine_Biosynthesis&organism=549.408) | [Queuosine Biosynthesis QueC ATPase](https://rast.nmpdr.org/seedviewer.cgi?page=FunctionalRole&role=Queuosine%20Biosynthesis%20QueC%20ATPase&subsystem_name=Queuosine-Archaeosine_Biosynthesis) |
| RNA Metabolism | RNA processing and modification | [Queuosine-Archaeosine Biosynthesis](https://rast.nmpdr.org/seedviewer.cgi?page=Subsystems&subsystem=Queuosine-Archaeosine_Biosynthesis&organism=549.408) | [glutamyl-Q-tRNA synthetase](https://rast.nmpdr.org/seedviewer.cgi?page=FunctionalRole&role=glutamyl-Q-tRNA%20synthetase&subsystem_name=Queuosine-Archaeosine_Biosynthesis) |
| RNA Metabolism | RNA processing and modification | [Queuosine-Archaeosine Biosynthesis](https://rast.nmpdr.org/seedviewer.cgi?page=Subsystems&subsystem=Queuosine-Archaeosine_Biosynthesis&organism=549.408) | [Peptidyl-prolyl cis-trans isomerase PpiB (EC 5.2.1.8)](https://rast.nmpdr.org/seedviewer.cgi?page=FunctionalRole&role=Peptidyl-prolyl%20cis-trans%20isomerase%20PpiB%20(EC%205.2.1.8)&subsystem_name=Queuosine-Archaeosine_Biosynthesis) |
| RNA Metabolism | RNA processing and modification | [Queuosine-Archaeosine Biosynthesis](https://rast.nmpdr.org/seedviewer.cgi?page=Subsystems&subsystem=Queuosine-Archaeosine_Biosynthesis&organism=549.408) | [tRNA-guanine transglycosylase (EC 2.4.2.29)](https://rast.nmpdr.org/seedviewer.cgi?page=FunctionalRole&role=tRNA-guanine%20transglycosylase%20(EC%202.4.2.29)&subsystem_name=Queuosine-Archaeosine_Biosynthesis) |
| RNA Metabolism | RNA processing and modification | [tRNA processing](https://rast.nmpdr.org/seedviewer.cgi?page=Subsystems&subsystem=tRNA_processing&organism=549.408) | [Ribonuclease P protein component (EC 3.1.26.5)](https://rast.nmpdr.org/seedviewer.cgi?page=FunctionalRole&role=Ribonuclease%20P%20protein%20component%20(EC%203.1.26.5)&subsystem_name=tRNA_processing) |
| RNA Metabolism | RNA processing and modification | [tRNA processing](https://rast.nmpdr.org/seedviewer.cgi?page=Subsystems&subsystem=tRNA_processing&organism=549.408) | [tRNA-i(6)A37 methylthiotransferase](https://rast.nmpdr.org/seedviewer.cgi?page=FunctionalRole&role=tRNA-i(6)A37%20methylthiotransferase&subsystem_name=tRNA_processing) |
| RNA Metabolism | RNA processing and modification | [tRNA processing](https://rast.nmpdr.org/seedviewer.cgi?page=Subsystems&subsystem=tRNA_processing&organism=549.408) | [Ribonuclease D (EC 3.1.26.3)](https://rast.nmpdr.org/seedviewer.cgi?page=FunctionalRole&role=Ribonuclease%20D%20(EC%203.1.26.3)&subsystem_name=tRNA_processing) |
| RNA Metabolism | RNA processing and modification | [tRNA processing](https://rast.nmpdr.org/seedviewer.cgi?page=Subsystems&subsystem=tRNA_processing&organism=549.408) | [Ribonuclease T (EC 3.1.13.-)](https://rast.nmpdr.org/seedviewer.cgi?page=FunctionalRole&role=Ribonuclease%20T%20(EC%203.1.13.-)&subsystem_name=tRNA_processing) |
| RNA Metabolism | RNA processing and modification | [tRNA processing](https://rast.nmpdr.org/seedviewer.cgi?page=Subsystems&subsystem=tRNA_processing&organism=549.408) | [tRNA(Ile)-lysidine synthetase (EC 6.3.4.19)](https://rast.nmpdr.org/seedviewer.cgi?page=FunctionalRole&role=tRNA(Ile)-lysidine%20synthetase%20(EC%206.3.4.19)&subsystem_name=tRNA_processing) |
| RNA Metabolism | RNA processing and modification | [tRNA processing](https://rast.nmpdr.org/seedviewer.cgi?page=Subsystems&subsystem=tRNA_processing&organism=549.408) | [tRNA pseudouridine 13 synthase (EC 4.2.1.-)](https://rast.nmpdr.org/seedviewer.cgi?page=FunctionalRole&role=tRNA%20pseudouridine%2013%20synthase%20(EC%204.2.1.-)&subsystem_name=tRNA_processing) |
| RNA Metabolism | RNA processing and modification | [tRNA processing](https://rast.nmpdr.org/seedviewer.cgi?page=Subsystems&subsystem=tRNA_processing&organism=549.408) | [tRNA pseudouridine synthase B (EC 4.2.1.70)](https://rast.nmpdr.org/seedviewer.cgi?page=FunctionalRole&role=tRNA%20pseudouridine%20synthase%20B%20(EC%204.2.1.70)&subsystem_name=tRNA_processing) |
| RNA Metabolism | RNA processing and modification | [tRNA processing](https://rast.nmpdr.org/seedviewer.cgi?page=Subsystems&subsystem=tRNA_processing&organism=549.408) | [Ribonuclease Z (EC 3.1.26.11)](https://rast.nmpdr.org/seedviewer.cgi?page=FunctionalRole&role=Ribonuclease%20Z%20(EC%203.1.26.11)&subsystem_name=tRNA_processing) |
| RNA Metabolism | RNA processing and modification | [tRNA processing](https://rast.nmpdr.org/seedviewer.cgi?page=Subsystems&subsystem=tRNA_processing&organism=549.408) | [tRNA-specific adenosine-34 deaminase (EC 3.5.4.-)](https://rast.nmpdr.org/seedviewer.cgi?page=FunctionalRole&role=tRNA-specific%20adenosine-34%20deaminase%20(EC%203.5.4.-)&subsystem_name=tRNA_processing) |
| RNA Metabolism | RNA processing and modification | [tRNA processing](https://rast.nmpdr.org/seedviewer.cgi?page=Subsystems&subsystem=tRNA_processing&organism=549.408) | [tRNA dimethylallyltransferase (EC 2.5.1.75)](https://rast.nmpdr.org/seedviewer.cgi?page=FunctionalRole&role=tRNA%20dimethylallyltransferase%20(EC%202.5.1.75)&subsystem_name=tRNA_processing) |
| RNA Metabolism | RNA processing and modification | [tRNA processing](https://rast.nmpdr.org/seedviewer.cgi?page=Subsystems&subsystem=tRNA_processing&organism=549.408) | [Ribonuclease PH (EC 2.7.7.56)](https://rast.nmpdr.org/seedviewer.cgi?page=FunctionalRole&role=Ribonuclease%20PH%20(EC%202.7.7.56)&subsystem_name=tRNA_processing) |
| RNA Metabolism | RNA processing and modification | [tRNA processing](https://rast.nmpdr.org/seedviewer.cgi?page=Subsystems&subsystem=tRNA_processing&organism=549.408) | [tRNA pseudouridine synthase A (EC 4.2.1.70)](https://rast.nmpdr.org/seedviewer.cgi?page=FunctionalRole&role=tRNA%20pseudouridine%20synthase%20A%20(EC%204.2.1.70)&subsystem_name=tRNA_processing) |
| RNA Metabolism | RNA processing and modification | [RNA processing orphans](https://rast.nmpdr.org/seedviewer.cgi?page=Subsystems&subsystem=RNA_processing_orphans&organism=549.408) | [2'-5' RNA ligase](https://rast.nmpdr.org/seedviewer.cgi?page=FunctionalRole&role=2) |
| RNA Metabolism | Transcription | [Transcription initiation, bacterial sigma factors](https://rast.nmpdr.org/seedviewer.cgi?page=Subsystems&subsystem=Transcription_initiation,_bacterial_sigma_factors&organism=549.408) | [RNA polymerase sigma factor RpoD](https://rast.nmpdr.org/seedviewer.cgi?page=FunctionalRole&role=RNA%20polymerase%20sigma%20factor%20RpoD&subsystem_name=Transcription_initiation,_bacterial_sigma_factors) |
| RNA Metabolism | Transcription | [Transcription initiation, bacterial sigma factors](https://rast.nmpdr.org/seedviewer.cgi?page=Subsystems&subsystem=Transcription_initiation,_bacterial_sigma_factors&organism=549.408) | [Sigma factor RpoE regulatory protein RseC](https://rast.nmpdr.org/seedviewer.cgi?page=FunctionalRole&role=Sigma%20factor%20RpoE%20regulatory%20protein%20RseC&subsystem_name=Transcription_initiation,_bacterial_sigma_factors) |
| RNA Metabolism | Transcription | [Transcription initiation, bacterial sigma factors](https://rast.nmpdr.org/seedviewer.cgi?page=Subsystems&subsystem=Transcription_initiation,_bacterial_sigma_factors&organism=549.408) | [RNA polymerase sigma factor RpoS](https://rast.nmpdr.org/seedviewer.cgi?page=FunctionalRole&role=RNA%20polymerase%20sigma%20factor%20RpoS&subsystem_name=Transcription_initiation,_bacterial_sigma_factors) |
| RNA Metabolism | Transcription | [Transcription initiation, bacterial sigma factors](https://rast.nmpdr.org/seedviewer.cgi?page=Subsystems&subsystem=Transcription_initiation,_bacterial_sigma_factors&organism=549.408) | [Sigma factor RpoE negative regulatory protein RseA](https://rast.nmpdr.org/seedviewer.cgi?page=FunctionalRole&role=Sigma%20factor%20RpoE%20negative%20regulatory%20protein%20RseA&subsystem_name=Transcription_initiation,_bacterial_sigma_factors) |
| RNA Metabolism | Transcription | [Transcription initiation, bacterial sigma factors](https://rast.nmpdr.org/seedviewer.cgi?page=Subsystems&subsystem=Transcription_initiation,_bacterial_sigma_factors&organism=549.408) | [RNA polymerase sigma factor RpoE](https://rast.nmpdr.org/seedviewer.cgi?page=FunctionalRole&role=RNA%20polymerase%20sigma%20factor%20RpoE&subsystem_name=Transcription_initiation,_bacterial_sigma_factors) |
| RNA Metabolism | Transcription | [Transcription initiation, bacterial sigma factors](https://rast.nmpdr.org/seedviewer.cgi?page=Subsystems&subsystem=Transcription_initiation,_bacterial_sigma_factors&organism=549.408) | [Sigma factor RpoE negative regulatory protein RseB precursor](https://rast.nmpdr.org/seedviewer.cgi?page=FunctionalRole&role=Sigma%20factor%20RpoE%20negative%20regulatory%20protein%20RseB%20precursor&subsystem_name=Transcription_initiation,_bacterial_sigma_factors) |
| RNA Metabolism | Transcription | [Transcription initiation, bacterial sigma factors](https://rast.nmpdr.org/seedviewer.cgi?page=Subsystems&subsystem=Transcription_initiation,_bacterial_sigma_factors&organism=549.408) | [RNA polymerase sigma-54 factor RpoN](https://rast.nmpdr.org/seedviewer.cgi?page=FunctionalRole&role=RNA%20polymerase%20sigma-54%20factor%20RpoN&subsystem_name=Transcription_initiation,_bacterial_sigma_factors) |
| RNA Metabolism | Transcription | [Transcription initiation, bacterial sigma factors](https://rast.nmpdr.org/seedviewer.cgi?page=Subsystems&subsystem=Transcription_initiation,_bacterial_sigma_factors&organism=549.408) | [RNA polymerase sigma factor RpoH](https://rast.nmpdr.org/seedviewer.cgi?page=FunctionalRole&role=RNA%20polymerase%20sigma%20factor%20RpoH&subsystem_name=Transcription_initiation,_bacterial_sigma_factors) |
| RNA Metabolism | Transcription | [Transcription initiation, bacterial sigma factors](https://rast.nmpdr.org/seedviewer.cgi?page=Subsystems&subsystem=Transcription_initiation,_bacterial_sigma_factors&organism=549.408) | [RNA polymerase sigma-70 factor](https://rast.nmpdr.org/seedviewer.cgi?page=FunctionalRole&role=RNA%20polymerase%20sigma-70%20factor&subsystem_name=Transcription_initiation,_bacterial_sigma_factors) |
| RNA Metabolism | Transcription | [Transcription initiation, bacterial sigma factors](https://rast.nmpdr.org/seedviewer.cgi?page=Subsystems&subsystem=Transcription_initiation,_bacterial_sigma_factors&organism=549.408) | [RNA polymerase sigma factor for flagellar operon](https://rast.nmpdr.org/seedviewer.cgi?page=FunctionalRole&role=RNA%20polymerase%20sigma%20factor%20for%20flagellar%20operon&subsystem_name=Transcription_initiation,_bacterial_sigma_factors) |
| RNA Metabolism | Transcription | [RNA polymerase bacterial](https://rast.nmpdr.org/seedviewer.cgi?page=Subsystems&subsystem=RNA_polymerase_bacterial&organism=549.408) | [DNA-directed RNA polymerase alpha subunit (EC 2.7.7.6)](https://rast.nmpdr.org/seedviewer.cgi?page=FunctionalRole&role=DNA-directed%20RNA%20polymerase%20alpha%20subunit%20(EC%202.7.7.6)&subsystem_name=RNA_polymerase_bacterial) |
| RNA Metabolism | Transcription | [RNA polymerase bacterial](https://rast.nmpdr.org/seedviewer.cgi?page=Subsystems&subsystem=RNA_polymerase_bacterial&organism=549.408) | [DNA-directed RNA polymerase beta' subunit (EC 2.7.7.6)](https://rast.nmpdr.org/seedviewer.cgi?page=FunctionalRole&role=DNA-directed%20RNA%20polymerase%20beta) |
| RNA Metabolism | Transcription | [RNA polymerase bacterial](https://rast.nmpdr.org/seedviewer.cgi?page=Subsystems&subsystem=RNA_polymerase_bacterial&organism=549.408) | [DNA-directed RNA polymerase omega subunit (EC 2.7.7.6)](https://rast.nmpdr.org/seedviewer.cgi?page=FunctionalRole&role=DNA-directed%20RNA%20polymerase%20omega%20subunit%20(EC%202.7.7.6)&subsystem_name=RNA_polymerase_bacterial) |
| RNA Metabolism | Transcription | [RNA polymerase bacterial](https://rast.nmpdr.org/seedviewer.cgi?page=Subsystems&subsystem=RNA_polymerase_bacterial&organism=549.408) | [DNA-directed RNA polymerase beta subunit (EC 2.7.7.6)](https://rast.nmpdr.org/seedviewer.cgi?page=FunctionalRole&role=DNA-directed%20RNA%20polymerase%20beta%20subunit%20(EC%202.7.7.6)&subsystem_name=RNA_polymerase_bacterial) |
| RNA Metabolism | Transcription | [Transcription factors bacterial](https://rast.nmpdr.org/seedviewer.cgi?page=Subsystems&subsystem=Transcription_factors_bacterial&organism=549.408) | [Transcription termination protein NusB](https://rast.nmpdr.org/seedviewer.cgi?page=FunctionalRole&role=Transcription%20termination%20protein%20NusB&subsystem_name=Transcription_factors_bacterial) |
| RNA Metabolism | Transcription | [Transcription factors bacterial](https://rast.nmpdr.org/seedviewer.cgi?page=Subsystems&subsystem=Transcription_factors_bacterial&organism=549.408) | [Transcription accessory protein (S1 RNA-binding domain)](https://rast.nmpdr.org/seedviewer.cgi?page=FunctionalRole&role=Transcription%20accessory%20protein%20(S1%20RNA-binding%20domain)&subsystem_name=Transcription_factors_bacterial) |
| RNA Metabolism | Transcription | [Transcription factors bacterial](https://rast.nmpdr.org/seedviewer.cgi?page=Subsystems&subsystem=Transcription_factors_bacterial&organism=549.408) | [Transcription elongation factor GreA](https://rast.nmpdr.org/seedviewer.cgi?page=FunctionalRole&role=Transcription%20elongation%20factor%20GreA&subsystem_name=Transcription_factors_bacterial) |
| RNA Metabolism | Transcription | [Transcription factors bacterial](https://rast.nmpdr.org/seedviewer.cgi?page=Subsystems&subsystem=Transcription_factors_bacterial&organism=549.408) | [Transcription termination factor Rho](https://rast.nmpdr.org/seedviewer.cgi?page=FunctionalRole&role=Transcription%20termination%20factor%20Rho&subsystem_name=Transcription_factors_bacterial) |
| RNA Metabolism | Transcription | [Transcription factors bacterial](https://rast.nmpdr.org/seedviewer.cgi?page=Subsystems&subsystem=Transcription_factors_bacterial&organism=549.408) | [Transcription antitermination protein NusG](https://rast.nmpdr.org/seedviewer.cgi?page=FunctionalRole&role=Transcription%20antitermination%20protein%20NusG&subsystem_name=Transcription_factors_bacterial) |
| RNA Metabolism | Transcription | [Transcription factors bacterial](https://rast.nmpdr.org/seedviewer.cgi?page=Subsystems&subsystem=Transcription_factors_bacterial&organism=549.408) | [Transcription-repair coupling factor](https://rast.nmpdr.org/seedviewer.cgi?page=FunctionalRole&role=Transcription-repair%20coupling%20factor&subsystem_name=Transcription_factors_bacterial) |
| RNA Metabolism | Transcription | [Transcription factors bacterial](https://rast.nmpdr.org/seedviewer.cgi?page=Subsystems&subsystem=Transcription_factors_bacterial&organism=549.408) | [Transcriptional activator RfaH](https://rast.nmpdr.org/seedviewer.cgi?page=FunctionalRole&role=Transcriptional%20activator%20RfaH&subsystem_name=Transcription_factors_bacterial) |
| RNA Metabolism | Transcription | [Transcription factors bacterial](https://rast.nmpdr.org/seedviewer.cgi?page=Subsystems&subsystem=Transcription_factors_bacterial&organism=549.408) | [FIG000325: clustered with transcription termination protein NusA](https://rast.nmpdr.org/seedviewer.cgi?page=FunctionalRole&role=FIG000325:%20clustered%20with%20transcription%20termination%20protein%20NusA&subsystem_name=Transcription_factors_bacterial) |
| RNA Metabolism | Transcription | [Transcription factors bacterial](https://rast.nmpdr.org/seedviewer.cgi?page=Subsystems&subsystem=Transcription_factors_bacterial&organism=549.408) | [Rho-specific inhibitor of transcription termination (YaeO)](https://rast.nmpdr.org/seedviewer.cgi?page=FunctionalRole&role=Rho-specific%20inhibitor%20of%20transcription%20termination%20(YaeO)&subsystem_name=Transcription_factors_bacterial) |
| RNA Metabolism | Transcription | [Transcription factors bacterial](https://rast.nmpdr.org/seedviewer.cgi?page=Subsystems&subsystem=Transcription_factors_bacterial&organism=549.408) | [Transcription elongation factor GreB](https://rast.nmpdr.org/seedviewer.cgi?page=FunctionalRole&role=Transcription%20elongation%20factor%20GreB&subsystem_name=Transcription_factors_bacterial) |
| RNA Metabolism | Transcription | [Transcription factors bacterial](https://rast.nmpdr.org/seedviewer.cgi?page=Subsystems&subsystem=Transcription_factors_bacterial&organism=549.408) | [Transcription termination protein NusA](https://rast.nmpdr.org/seedviewer.cgi?page=FunctionalRole&role=Transcription%20termination%20protein%20NusA&subsystem_name=Transcription_factors_bacterial) |
| RNA Metabolism | Transcription | [Rrf2 family transcriptional regulators](https://rast.nmpdr.org/seedviewer.cgi?page=Subsystems&subsystem=Rrf2_family_transcriptional_regulators&organism=549.408) | [Nitrite-sensitive transcriptional repressor NsrR](https://rast.nmpdr.org/seedviewer.cgi?page=FunctionalRole&role=Nitrite-sensitive%20transcriptional%20repressor%20NsrR&subsystem_name=Rrf2_family_transcriptional_regulators) |
| RNA Metabolism | Transcription | [Rrf2 family transcriptional regulators](https://rast.nmpdr.org/seedviewer.cgi?page=Subsystems&subsystem=Rrf2_family_transcriptional_regulators&organism=549.408) | [Rrf2 family transcriptional regulator, group III](https://rast.nmpdr.org/seedviewer.cgi?page=FunctionalRole&role=Rrf2%20family%20transcriptional%20regulator,%20group%20III&subsystem_name=Rrf2_family_transcriptional_regulators) |
| RNA Metabolism | Transcription | [Rrf2 family transcriptional regulators](https://rast.nmpdr.org/seedviewer.cgi?page=Subsystems&subsystem=Rrf2_family_transcriptional_regulators&organism=549.408) | [Iron-sulfur cluster regulator IscR](https://rast.nmpdr.org/seedviewer.cgi?page=FunctionalRole&role=Iron-sulfur%20cluster%20regulator%20IscR&subsystem_name=Rrf2_family_transcriptional_regulators) |
| RNA Metabolism | RNA Metabolism - no subcategory | [Group II intron-associated genes](https://rast.nmpdr.org/seedviewer.cgi?page=Subsystems&subsystem=Group_II_intron-associated_genes&organism=549.408) | [Retron-type RNA-directed DNA polymerase (EC 2.7.7.49)](https://rast.nmpdr.org/seedviewer.cgi?page=FunctionalRole&role=Retron-type%20RNA-directed%20DNA%20polymerase%20(EC%202.7.7.49)&subsystem_name=Group_II_intron-associated_genes) |
| Nucleosides and Nucleotides | Pyrimidines | [pyrimidine conversions](https://rast.nmpdr.org/seedviewer.cgi?page=Subsystems&subsystem=pyrimidine_conversions&organism=549.408) | [Cytosine deaminase (EC 3.5.4.1)](https://rast.nmpdr.org/seedviewer.cgi?page=FunctionalRole&role=Cytosine%20deaminase%20(EC%203.5.4.1)&subsystem_name=pyrimidine_conversions) |
| Nucleosides and Nucleotides | Pyrimidines | [pyrimidine conversions](https://rast.nmpdr.org/seedviewer.cgi?page=Subsystems&subsystem=pyrimidine_conversions&organism=549.408) | [Thymidine kinase (EC 2.7.1.21)](https://rast.nmpdr.org/seedviewer.cgi?page=FunctionalRole&role=Thymidine%20kinase%20(EC%202.7.1.21)&subsystem_name=pyrimidine_conversions) |
| Nucleosides and Nucleotides | Pyrimidines | [pyrimidine conversions](https://rast.nmpdr.org/seedviewer.cgi?page=Subsystems&subsystem=pyrimidine_conversions&organism=549.408) | [Thymidylate synthase (EC 2.1.1.45)](https://rast.nmpdr.org/seedviewer.cgi?page=FunctionalRole&role=Thymidylate%20synthase%20(EC%202.1.1.45)&subsystem_name=pyrimidine_conversions) |
| Nucleosides and Nucleotides | Pyrimidines | [pyrimidine conversions](https://rast.nmpdr.org/seedviewer.cgi?page=Subsystems&subsystem=pyrimidine_conversions&organism=549.408) | [Thymidine phosphorylase (EC 2.4.2.4)](https://rast.nmpdr.org/seedviewer.cgi?page=FunctionalRole&role=Thymidine%20phosphorylase%20(EC%202.4.2.4)&subsystem_name=pyrimidine_conversions) |
| Nucleosides and Nucleotides | Pyrimidines | [pyrimidine conversions](https://rast.nmpdr.org/seedviewer.cgi?page=Subsystems&subsystem=pyrimidine_conversions&organism=549.408) | [2',3'-cyclic-nucleotide 2'-phosphodiesterase (EC 3.1.4.16)](https://rast.nmpdr.org/seedviewer.cgi?page=FunctionalRole&role=2) |
| Nucleosides and Nucleotides | Pyrimidines | [pyrimidine conversions](https://rast.nmpdr.org/seedviewer.cgi?page=Subsystems&subsystem=pyrimidine_conversions&organism=549.408) | [CTP synthase (EC 6.3.4.2)](https://rast.nmpdr.org/seedviewer.cgi?page=FunctionalRole&role=CTP%20synthase%20(EC%206.3.4.2)&subsystem_name=pyrimidine_conversions) |
| Nucleosides and Nucleotides | Pyrimidines | [pyrimidine conversions](https://rast.nmpdr.org/seedviewer.cgi?page=Subsystems&subsystem=pyrimidine_conversions&organism=549.408) | [Deoxycytidine triphosphate deaminase (EC 3.5.4.13)](https://rast.nmpdr.org/seedviewer.cgi?page=FunctionalRole&role=Deoxycytidine%20triphosphate%20deaminase%20(EC%203.5.4.13)&subsystem_name=pyrimidine_conversions) |
| Nucleosides and Nucleotides | Pyrimidines | [pyrimidine conversions](https://rast.nmpdr.org/seedviewer.cgi?page=Subsystems&subsystem=pyrimidine_conversions&organism=549.408) | [Nucleoside diphosphate kinase (EC 2.7.4.6)](https://rast.nmpdr.org/seedviewer.cgi?page=FunctionalRole&role=Nucleoside%20diphosphate%20kinase%20(EC%202.7.4.6)&subsystem_name=pyrimidine_conversions) |
| Nucleosides and Nucleotides | Pyrimidines | [pyrimidine conversions](https://rast.nmpdr.org/seedviewer.cgi?page=Subsystems&subsystem=pyrimidine_conversions&organism=549.408) | [Uridine kinase (EC 2.7.1.48) [C1]](https://rast.nmpdr.org/seedviewer.cgi?page=FunctionalRole&role=Uridine%20kinase%20(EC%202.7.1.48)%20%5bC1%5d&subsystem_name=pyrimidine_conversions) |
| Nucleosides and Nucleotides | Pyrimidines | [pyrimidine conversions](https://rast.nmpdr.org/seedviewer.cgi?page=Subsystems&subsystem=pyrimidine_conversions&organism=549.408) | [Thymidylate kinase (EC 2.7.4.9)](https://rast.nmpdr.org/seedviewer.cgi?page=FunctionalRole&role=Thymidylate%20kinase%20(EC%202.7.4.9)&subsystem_name=pyrimidine_conversions) |
| Nucleosides and Nucleotides | Pyrimidines | [pyrimidine conversions](https://rast.nmpdr.org/seedviewer.cgi?page=Subsystems&subsystem=pyrimidine_conversions&organism=549.408) | [Thioredoxin reductase (EC 1.8.1.9)](https://rast.nmpdr.org/seedviewer.cgi?page=FunctionalRole&role=Thioredoxin%20reductase%20(EC%201.8.1.9)&subsystem_name=pyrimidine_conversions) |
| Nucleosides and Nucleotides | Pyrimidines | [pyrimidine conversions](https://rast.nmpdr.org/seedviewer.cgi?page=Subsystems&subsystem=pyrimidine_conversions&organism=549.408) | [Uridine phosphorylase (EC 2.4.2.3)](https://rast.nmpdr.org/seedviewer.cgi?page=FunctionalRole&role=Uridine%20phosphorylase%20(EC%202.4.2.3)&subsystem_name=pyrimidine_conversions) |
| Nucleosides and Nucleotides | Pyrimidines | [pyrimidine conversions](https://rast.nmpdr.org/seedviewer.cgi?page=Subsystems&subsystem=pyrimidine_conversions&organism=549.408) | [Cytidylate kinase (EC 2.7.4.25)](https://rast.nmpdr.org/seedviewer.cgi?page=FunctionalRole&role=Cytidylate%20kinase%20(EC%202.7.4.25)&subsystem_name=pyrimidine_conversions) |
| Nucleosides and Nucleotides | Pyrimidines | [pyrimidine conversions](https://rast.nmpdr.org/seedviewer.cgi?page=Subsystems&subsystem=pyrimidine_conversions&organism=549.408) | [Purine nucleoside phosphorylase (EC 2.4.2.1)](https://rast.nmpdr.org/seedviewer.cgi?page=FunctionalRole&role=Purine%20nucleoside%20phosphorylase%20(EC%202.4.2.1)&subsystem_name=pyrimidine_conversions) |
| Nucleosides and Nucleotides | Pyrimidines | [pyrimidine conversions](https://rast.nmpdr.org/seedviewer.cgi?page=Subsystems&subsystem=pyrimidine_conversions&organism=549.408) | [5'-nucleotidase (EC 3.1.3.5)](https://rast.nmpdr.org/seedviewer.cgi?page=FunctionalRole&role=5) |
| Nucleosides and Nucleotides | Pyrimidines | [pyrimidine conversions](https://rast.nmpdr.org/seedviewer.cgi?page=Subsystems&subsystem=pyrimidine_conversions&organism=549.408) | [Cytidine deaminase (EC 3.5.4.5)](https://rast.nmpdr.org/seedviewer.cgi?page=FunctionalRole&role=Cytidine%20deaminase%20(EC%203.5.4.5)&subsystem_name=pyrimidine_conversions) |
| Nucleosides and Nucleotides | Pyrimidines | [pyrimidine conversions](https://rast.nmpdr.org/seedviewer.cgi?page=Subsystems&subsystem=pyrimidine_conversions&organism=549.408) | [Uracil phosphoribosyltransferase (EC 2.4.2.9)](https://rast.nmpdr.org/seedviewer.cgi?page=FunctionalRole&role=Uracil%20phosphoribosyltransferase%20(EC%202.4.2.9)&subsystem_name=pyrimidine_conversions) |
| Nucleosides and Nucleotides | Pyrimidines | [Pyrimidine utilization](https://rast.nmpdr.org/seedviewer.cgi?page=Subsystems&subsystem=Pyrimidine_utilization&organism=549.408) | [Uracil permease](https://rast.nmpdr.org/seedviewer.cgi?page=FunctionalRole&role=Uracil%20permease&subsystem_name=Pyrimidine_utilization) |
| Nucleosides and Nucleotides | Pyrimidines | [Pyrimidine utilization](https://rast.nmpdr.org/seedviewer.cgi?page=Subsystems&subsystem=Pyrimidine_utilization&organism=549.408) | [Predicted amidohydrolase RutB in novel pyrimidine catabolism pathway](https://rast.nmpdr.org/seedviewer.cgi?page=FunctionalRole&role=Predicted%20amidohydrolase%20RutB%20in%20novel%20pyrimidine%20catabolism%20pathway&subsystem_name=Pyrimidine_utilization) |
| Nucleosides and Nucleotides | Pyrimidines | [Pyrimidine utilization](https://rast.nmpdr.org/seedviewer.cgi?page=Subsystems&subsystem=Pyrimidine_utilization&organism=549.408) | [Predicted reductase RutE in novel pyrimidine catabolism pathway](https://rast.nmpdr.org/seedviewer.cgi?page=FunctionalRole&role=Predicted%20reductase%20RutE%20in%20novel%20pyrimidine%20catabolism%20pathway&subsystem_name=Pyrimidine_utilization) |
| Nucleosides and Nucleotides | Pyrimidines | [Pyrimidine utilization](https://rast.nmpdr.org/seedviewer.cgi?page=Subsystems&subsystem=Pyrimidine_utilization&organism=549.408) | [Possible hydrolase or acyltransferase RutD in novel pyrimidine catabolism pathway](https://rast.nmpdr.org/seedviewer.cgi?page=FunctionalRole&role=Possible%20hydrolase%20or%20acyltransferase%20RutD%20in%20novel%20pyrimidine%20catabolism%20pathway&subsystem_name=Pyrimidine_utilization) |
| Nucleosides and Nucleotides | Pyrimidines | [Pyrimidine utilization](https://rast.nmpdr.org/seedviewer.cgi?page=Subsystems&subsystem=Pyrimidine_utilization&organism=549.408) | [Predicted monooxygenase RutA in novel pyrimidine catabolism pathway](https://rast.nmpdr.org/seedviewer.cgi?page=FunctionalRole&role=Predicted%20monooxygenase%20RutA%20in%20novel%20pyrimidine%20catabolism%20pathway&subsystem_name=Pyrimidine_utilization) |
| Nucleosides and Nucleotides | Pyrimidines | [Pyrimidine utilization](https://rast.nmpdr.org/seedviewer.cgi?page=Subsystems&subsystem=Pyrimidine_utilization&organism=549.408) | [Predicted flavin reductase RutF in novel pyrimidine catabolism pathway](https://rast.nmpdr.org/seedviewer.cgi?page=FunctionalRole&role=Predicted%20flavin%20reductase%20RutF%20in%20novel%20pyrimidine%20catabolism%20pathway&subsystem_name=Pyrimidine_utilization) |
| Nucleosides and Nucleotides | Pyrimidines | [Pyrimidine utilization](https://rast.nmpdr.org/seedviewer.cgi?page=Subsystems&subsystem=Pyrimidine_utilization&organism=549.408) | [Transcriptional regulator RutR of pyrimidine catabolism (TetR family)](https://rast.nmpdr.org/seedviewer.cgi?page=FunctionalRole&role=Transcriptional%20regulator%20RutR%20of%20pyrimidine%20catabolism%20(TetR%20family)&subsystem_name=Pyrimidine_utilization) |
| Nucleosides and Nucleotides | Pyrimidines | [De Novo Pyrimidine Synthesis](https://rast.nmpdr.org/seedviewer.cgi?page=Subsystems&subsystem=De_Novo_Pyrimidine_Synthesis&organism=549.408) | [Uracil permease](https://rast.nmpdr.org/seedviewer.cgi?page=FunctionalRole&role=Uracil%20permease&subsystem_name=De_Novo_Pyrimidine_Synthesis) |
| Nucleosides and Nucleotides | Pyrimidines | [De Novo Pyrimidine Synthesis](https://rast.nmpdr.org/seedviewer.cgi?page=Subsystems&subsystem=De_Novo_Pyrimidine_Synthesis&organism=549.408) | [Orotidine 5'-phosphate decarboxylase (EC 4.1.1.23)](https://rast.nmpdr.org/seedviewer.cgi?page=FunctionalRole&role=Orotidine%205) |
| Nucleosides and Nucleotides | Pyrimidines | [De Novo Pyrimidine Synthesis](https://rast.nmpdr.org/seedviewer.cgi?page=Subsystems&subsystem=De_Novo_Pyrimidine_Synthesis&organism=549.408) | [Aspartate carbamoyltransferase (EC 2.1.3.2)](https://rast.nmpdr.org/seedviewer.cgi?page=FunctionalRole&role=Aspartate%20carbamoyltransferase%20(EC%202.1.3.2)&subsystem_name=De_Novo_Pyrimidine_Synthesis) |
| Nucleosides and Nucleotides | Pyrimidines | [De Novo Pyrimidine Synthesis](https://rast.nmpdr.org/seedviewer.cgi?page=Subsystems&subsystem=De_Novo_Pyrimidine_Synthesis&organism=549.408) | [Dihydroorotate dehydrogenase (EC 1.3.3.1)](https://rast.nmpdr.org/seedviewer.cgi?page=FunctionalRole&role=Dihydroorotate%20dehydrogenase%20(EC%201.3.3.1)&subsystem_name=De_Novo_Pyrimidine_Synthesis) |
| Nucleosides and Nucleotides | Pyrimidines | [De Novo Pyrimidine Synthesis](https://rast.nmpdr.org/seedviewer.cgi?page=Subsystems&subsystem=De_Novo_Pyrimidine_Synthesis&organism=549.408) | [Carbamoyl-phosphate synthase small chain (EC 6.3.5.5)](https://rast.nmpdr.org/seedviewer.cgi?page=FunctionalRole&role=Carbamoyl-phosphate%20synthase%20small%20chain%20(EC%206.3.5.5)&subsystem_name=De_Novo_Pyrimidine_Synthesis) |
| Nucleosides and Nucleotides | Pyrimidines | [De Novo Pyrimidine Synthesis](https://rast.nmpdr.org/seedviewer.cgi?page=Subsystems&subsystem=De_Novo_Pyrimidine_Synthesis&organism=549.408) | [Orotate phosphoribosyltransferase (EC 2.4.2.10)](https://rast.nmpdr.org/seedviewer.cgi?page=FunctionalRole&role=Orotate%20phosphoribosyltransferase%20(EC%202.4.2.10)&subsystem_name=De_Novo_Pyrimidine_Synthesis) |
| Nucleosides and Nucleotides | Pyrimidines | [De Novo Pyrimidine Synthesis](https://rast.nmpdr.org/seedviewer.cgi?page=Subsystems&subsystem=De_Novo_Pyrimidine_Synthesis&organism=549.408) | [Aspartate carbamoyltransferase regulatory chain (PyrI)](https://rast.nmpdr.org/seedviewer.cgi?page=FunctionalRole&role=Aspartate%20carbamoyltransferase%20regulatory%20chain%20(PyrI)&subsystem_name=De_Novo_Pyrimidine_Synthesis) |
| Nucleosides and Nucleotides | Pyrimidines | [De Novo Pyrimidine Synthesis](https://rast.nmpdr.org/seedviewer.cgi?page=Subsystems&subsystem=De_Novo_Pyrimidine_Synthesis&organism=549.408) | [Carbamoyl-phosphate synthase large chain (EC 6.3.5.5)](https://rast.nmpdr.org/seedviewer.cgi?page=FunctionalRole&role=Carbamoyl-phosphate%20synthase%20large%20chain%20(EC%206.3.5.5)&subsystem_name=De_Novo_Pyrimidine_Synthesis) |
| Nucleosides and Nucleotides | Pyrimidines | [De Novo Pyrimidine Synthesis](https://rast.nmpdr.org/seedviewer.cgi?page=Subsystems&subsystem=De_Novo_Pyrimidine_Synthesis&organism=549.408) | [Uracil phosphoribosyltransferase (EC 2.4.2.9)](https://rast.nmpdr.org/seedviewer.cgi?page=FunctionalRole&role=Uracil%20phosphoribosyltransferase%20(EC%202.4.2.9)&subsystem_name=De_Novo_Pyrimidine_Synthesis) |
| Nucleosides and Nucleotides | Pyrimidines | [De Novo Pyrimidine Synthesis](https://rast.nmpdr.org/seedviewer.cgi?page=Subsystems&subsystem=De_Novo_Pyrimidine_Synthesis&organism=549.408) | [Dihydroorotase (EC 3.5.2.3)](https://rast.nmpdr.org/seedviewer.cgi?page=FunctionalRole&role=Dihydroorotase%20(EC%203.5.2.3)&subsystem_name=De_Novo_Pyrimidine_Synthesis) |
| Nucleosides and Nucleotides | Purines | [De Novo Purine Biosynthesis](https://rast.nmpdr.org/seedviewer.cgi?page=Subsystems&subsystem=De_Novo_Purine_Biosynthesis&organism=549.408) | [Phosphoribosylamine--glycine ligase (EC 6.3.4.13)](https://rast.nmpdr.org/seedviewer.cgi?page=FunctionalRole&role=Phosphoribosylamine--glycine%20ligase%20(EC%206.3.4.13)&subsystem_name=De_Novo_Purine_Biosynthesis) |
| Nucleosides and Nucleotides | Purines | [De Novo Purine Biosynthesis](https://rast.nmpdr.org/seedviewer.cgi?page=Subsystems&subsystem=De_Novo_Purine_Biosynthesis&organism=549.408) | [IMP cyclohydrolase (EC 3.5.4.10)](https://rast.nmpdr.org/seedviewer.cgi?page=FunctionalRole&role=IMP%20cyclohydrolase%20(EC%203.5.4.10)&subsystem_name=De_Novo_Purine_Biosynthesis) |
| Nucleosides and Nucleotides | Purines | [De Novo Purine Biosynthesis](https://rast.nmpdr.org/seedviewer.cgi?page=Subsystems&subsystem=De_Novo_Purine_Biosynthesis&organism=549.408) | [Amidophosphoribosyltransferase (EC 2.4.2.14)](https://rast.nmpdr.org/seedviewer.cgi?page=FunctionalRole&role=Amidophosphoribosyltransferase%20(EC%202.4.2.14)&subsystem_name=De_Novo_Purine_Biosynthesis) |
| Nucleosides and Nucleotides | Purines | [De Novo Purine Biosynthesis](https://rast.nmpdr.org/seedviewer.cgi?page=Subsystems&subsystem=De_Novo_Purine_Biosynthesis&organism=549.408) | [Ribose-phosphate pyrophosphokinase (EC 2.7.6.1)](https://rast.nmpdr.org/seedviewer.cgi?page=FunctionalRole&role=Ribose-phosphate%20pyrophosphokinase%20(EC%202.7.6.1)&subsystem_name=De_Novo_Purine_Biosynthesis) |
| Nucleosides and Nucleotides | Purines | [De Novo Purine Biosynthesis](https://rast.nmpdr.org/seedviewer.cgi?page=Subsystems&subsystem=De_Novo_Purine_Biosynthesis&organism=549.408) | [Phosphoribosylaminoimidazole carboxylase catalytic subunit (EC 4.1.1.21)](https://rast.nmpdr.org/seedviewer.cgi?page=FunctionalRole&role=Phosphoribosylaminoimidazole%20carboxylase%20catalytic%20subunit%20(EC%204.1.1.21)&subsystem_name=De_Novo_Purine_Biosynthesis) |
| Nucleosides and Nucleotides | Purines | [De Novo Purine Biosynthesis](https://rast.nmpdr.org/seedviewer.cgi?page=Subsystems&subsystem=De_Novo_Purine_Biosynthesis&organism=549.408) | [Phosphoribosylglycinamide formyltransferase 2 (EC 2.1.2.-)](https://rast.nmpdr.org/seedviewer.cgi?page=FunctionalRole&role=Phosphoribosylglycinamide%20formyltransferase%202%20(EC%202.1.2.-)&subsystem_name=De_Novo_Purine_Biosynthesis) |
| Nucleosides and Nucleotides | Purines | [De Novo Purine Biosynthesis](https://rast.nmpdr.org/seedviewer.cgi?page=Subsystems&subsystem=De_Novo_Purine_Biosynthesis&organism=549.408) | [Phosphoribosylglycinamide formyltransferase (EC 2.1.2.2)](https://rast.nmpdr.org/seedviewer.cgi?page=FunctionalRole&role=Phosphoribosylglycinamide%20formyltransferase%20(EC%202.1.2.2)&subsystem_name=De_Novo_Purine_Biosynthesis) |
| Nucleosides and Nucleotides | Purines | [De Novo Purine Biosynthesis](https://rast.nmpdr.org/seedviewer.cgi?page=Subsystems&subsystem=De_Novo_Purine_Biosynthesis&organism=549.408) | [Phosphoribosylaminoimidazole-succinocarboxamide synthase (EC 6.3.2.6)](https://rast.nmpdr.org/seedviewer.cgi?page=FunctionalRole&role=Phosphoribosylaminoimidazole-succinocarboxamide%20synthase%20(EC%206.3.2.6)&subsystem_name=De_Novo_Purine_Biosynthesis) |
| Nucleosides and Nucleotides | Purines | [De Novo Purine Biosynthesis](https://rast.nmpdr.org/seedviewer.cgi?page=Subsystems&subsystem=De_Novo_Purine_Biosynthesis&organism=549.408) | [Adenylosuccinate lyase (EC 4.3.2.2)](https://rast.nmpdr.org/seedviewer.cgi?page=FunctionalRole&role=Adenylosuccinate%20lyase%20(EC%204.3.2.2)&subsystem_name=De_Novo_Purine_Biosynthesis) |
| Nucleosides and Nucleotides | Purines | [De Novo Purine Biosynthesis](https://rast.nmpdr.org/seedviewer.cgi?page=Subsystems&subsystem=De_Novo_Purine_Biosynthesis&organism=549.408) | [Phosphoribosylformylglycinamidine cyclo-ligase (EC 6.3.3.1)](https://rast.nmpdr.org/seedviewer.cgi?page=FunctionalRole&role=Phosphoribosylformylglycinamidine%20cyclo-ligase%20(EC%206.3.3.1)&subsystem_name=De_Novo_Purine_Biosynthesis) |
| Nucleosides and Nucleotides | Purines | [De Novo Purine Biosynthesis](https://rast.nmpdr.org/seedviewer.cgi?page=Subsystems&subsystem=De_Novo_Purine_Biosynthesis&organism=549.408) | [Phosphoribosylaminoimidazolecarboxamide formyltransferase (EC 2.1.2.3)](https://rast.nmpdr.org/seedviewer.cgi?page=FunctionalRole&role=Phosphoribosylaminoimidazolecarboxamide%20formyltransferase%20(EC%202.1.2.3)&subsystem_name=De_Novo_Purine_Biosynthesis) |
| Nucleosides and Nucleotides | Purines | [De Novo Purine Biosynthesis](https://rast.nmpdr.org/seedviewer.cgi?page=Subsystems&subsystem=De_Novo_Purine_Biosynthesis&organism=549.408) | [Phosphoribosylformylglycinamidine synthase, synthetase subunit (EC 6.3.5.3)](https://rast.nmpdr.org/seedviewer.cgi?page=FunctionalRole&role=Phosphoribosylformylglycinamidine%20synthase,%20synthetase%20subunit%20(EC%206.3.5.3)&subsystem_name=De_Novo_Purine_Biosynthesis) |
| Nucleosides and Nucleotides | Purines | [De Novo Purine Biosynthesis](https://rast.nmpdr.org/seedviewer.cgi?page=Subsystems&subsystem=De_Novo_Purine_Biosynthesis&organism=549.408) | [Phosphoribosylaminoimidazole carboxylase ATPase subunit (EC 4.1.1.21)](https://rast.nmpdr.org/seedviewer.cgi?page=FunctionalRole&role=Phosphoribosylaminoimidazole%20carboxylase%20ATPase%20subunit%20(EC%204.1.1.21)&subsystem_name=De_Novo_Purine_Biosynthesis) |
| Nucleosides and Nucleotides | Purines | [De Novo Purine Biosynthesis](https://rast.nmpdr.org/seedviewer.cgi?page=Subsystems&subsystem=De_Novo_Purine_Biosynthesis&organism=549.408) | [Phosphoribosylformylglycinamidine synthase, glutamine amidotransferase subunit (EC 6.3.5.3)](https://rast.nmpdr.org/seedviewer.cgi?page=FunctionalRole&role=Phosphoribosylformylglycinamidine%20synthase,%20glutamine%20amidotransferase%20subunit%20(EC%206.3.5.3)&subsystem_name=De_Novo_Purine_Biosynthesis) |
| Nucleosides and Nucleotides | Purines | [Xanthine Metabolism in Bacteria](https://rast.nmpdr.org/seedviewer.cgi?page=Subsystems&subsystem=Xanthine_Metabolism_in_Bacteria&organism=549.408) | [Xanthine permease](https://rast.nmpdr.org/seedviewer.cgi?page=FunctionalRole&role=Xanthine%20permease&subsystem_name=Xanthine_Metabolism_in_Bacteria) |
| Nucleosides and Nucleotides | Purines | [Purine nucleotide synthesis regulator](https://rast.nmpdr.org/seedviewer.cgi?page=Subsystems&subsystem=Purine_nucleotide_synthesis_regulator&organism=549.408) | [Purine nucleotide synthesis repressor](https://rast.nmpdr.org/seedviewer.cgi?page=FunctionalRole&role=Purine%20nucleotide%20synthesis%20repressor&subsystem_name=Purine_nucleotide_synthesis_regulator) |
| Nucleosides and Nucleotides | Purines | [Purine Utilization](https://rast.nmpdr.org/seedviewer.cgi?page=Subsystems&subsystem=Purine_Utilization&organism=549.408) | [Periplasmic aromatic aldehyde oxidoreductase, FAD binding subunit YagS](https://rast.nmpdr.org/seedviewer.cgi?page=FunctionalRole&role=Periplasmic%20aromatic%20aldehyde%20oxidoreductase,%20FAD%20binding%20subunit%20YagS&subsystem_name=Purine_Utilization) |
| Nucleosides and Nucleotides | Purines | [Purine Utilization](https://rast.nmpdr.org/seedviewer.cgi?page=Subsystems&subsystem=Purine_Utilization&organism=549.408) | [Guanine-hypoxanthine permease](https://rast.nmpdr.org/seedviewer.cgi?page=FunctionalRole&role=Guanine-hypoxanthine%20permease&subsystem_name=Purine_Utilization) |
| Nucleosides and Nucleotides | Purines | [Purine Utilization](https://rast.nmpdr.org/seedviewer.cgi?page=Subsystems&subsystem=Purine_Utilization&organism=549.408) | [Cytosine/purine/uracil/thiamine/allantoin permease family protein](https://rast.nmpdr.org/seedviewer.cgi?page=FunctionalRole&role=Cytosine/purine/uracil/thiamine/allantoin%20permease%20family%20protein&subsystem_name=Purine_Utilization) |
| Nucleosides and Nucleotides | Purines | [Purine Utilization](https://rast.nmpdr.org/seedviewer.cgi?page=Subsystems&subsystem=Purine_Utilization&organism=549.408) | [Xanthine/uracil/thiamine/ascorbate permease family protein](https://rast.nmpdr.org/seedviewer.cgi?page=FunctionalRole&role=Xanthine/uracil/thiamine/ascorbate%20permease%20family%20protein&subsystem_name=Purine_Utilization) |
| Nucleosides and Nucleotides | Purines | [Purine Utilization](https://rast.nmpdr.org/seedviewer.cgi?page=Subsystems&subsystem=Purine_Utilization&organism=549.408) | [Xanthine and CO dehydrogenases maturation factor, XdhC/CoxF family](https://rast.nmpdr.org/seedviewer.cgi?page=FunctionalRole&role=Xanthine%20and%20CO%20dehydrogenases%20maturation%20factor,%20XdhC/CoxF%20family&subsystem_name=Purine_Utilization) |
| Nucleosides and Nucleotides | Purines | [Purine Utilization](https://rast.nmpdr.org/seedviewer.cgi?page=Subsystems&subsystem=Purine_Utilization&organism=549.408) | [Xanthine dehydrogenase, molybdenum binding subunit (EC 1.17.1.4)](https://rast.nmpdr.org/seedviewer.cgi?page=FunctionalRole&role=Xanthine%20dehydrogenase,%20molybdenum%20binding%20subunit%20(EC%201.17.1.4)&subsystem_name=Purine_Utilization) |
| Nucleosides and Nucleotides | Purines | [Purine Utilization](https://rast.nmpdr.org/seedviewer.cgi?page=Subsystems&subsystem=Purine_Utilization&organism=549.408) | [Xanthine permease](https://rast.nmpdr.org/seedviewer.cgi?page=FunctionalRole&role=Xanthine%20permease&subsystem_name=Purine_Utilization) |
| Nucleosides and Nucleotides | Purines | [Purine Utilization](https://rast.nmpdr.org/seedviewer.cgi?page=Subsystems&subsystem=Purine_Utilization&organism=549.408) | [Guanine deaminase (EC 3.5.4.3)](https://rast.nmpdr.org/seedviewer.cgi?page=FunctionalRole&role=Guanine%20deaminase%20(EC%203.5.4.3)&subsystem_name=Purine_Utilization) |
| Nucleosides and Nucleotides | Purines | [Purine conversions](https://rast.nmpdr.org/seedviewer.cgi?page=Subsystems&subsystem=Purine_conversions&organism=549.408) | [GMP synthase [glutamine-hydrolyzing] (EC 6.3.5.2)](https://rast.nmpdr.org/seedviewer.cgi?page=FunctionalRole&role=GMP%20synthase%20%5bglutamine-hydrolyzing%5d%20(EC%206.3.5.2)&subsystem_name=Purine_conversions) |
| Nucleosides and Nucleotides | Purines | [Purine conversions](https://rast.nmpdr.org/seedviewer.cgi?page=Subsystems&subsystem=Purine_conversions&organism=549.408) | [Adenylosuccinate synthetase (EC 6.3.4.4)](https://rast.nmpdr.org/seedviewer.cgi?page=FunctionalRole&role=Adenylosuccinate%20synthetase%20(EC%206.3.4.4)&subsystem_name=Purine_conversions) |
| Nucleosides and Nucleotides | Purines | [Purine conversions](https://rast.nmpdr.org/seedviewer.cgi?page=Subsystems&subsystem=Purine_conversions&organism=549.408) | [Inosine-5'-monophosphate dehydrogenase (EC 1.1.1.205)](https://rast.nmpdr.org/seedviewer.cgi?page=FunctionalRole&role=Inosine-5) |
| Nucleosides and Nucleotides | Purines | [Purine conversions](https://rast.nmpdr.org/seedviewer.cgi?page=Subsystems&subsystem=Purine_conversions&organism=549.408) | [GMP reductase (EC 1.7.1.7)](https://rast.nmpdr.org/seedviewer.cgi?page=FunctionalRole&role=GMP%20reductase%20(EC%201.7.1.7)&subsystem_name=Purine_conversions) |
| Nucleosides and Nucleotides | Purines | [Purine conversions](https://rast.nmpdr.org/seedviewer.cgi?page=Subsystems&subsystem=Purine_conversions&organism=549.408) | [Hypoxanthine-guanine phosphoribosyltransferase (EC 2.4.2.8)](https://rast.nmpdr.org/seedviewer.cgi?page=FunctionalRole&role=Hypoxanthine-guanine%20phosphoribosyltransferase%20(EC%202.4.2.8)&subsystem_name=Purine_conversions) |
| Nucleosides and Nucleotides | Purines | [Purine conversions](https://rast.nmpdr.org/seedviewer.cgi?page=Subsystems&subsystem=Purine_conversions&organism=549.408) | [Adenylosuccinate lyase (EC 4.3.2.2)](https://rast.nmpdr.org/seedviewer.cgi?page=FunctionalRole&role=Adenylosuccinate%20lyase%20(EC%204.3.2.2)&subsystem_name=Purine_conversions) |
| Nucleosides and Nucleotides | Purines | [Purine conversions](https://rast.nmpdr.org/seedviewer.cgi?page=Subsystems&subsystem=Purine_conversions&organism=549.408) | [Polyphosphate kinase (EC 2.7.4.1)](https://rast.nmpdr.org/seedviewer.cgi?page=FunctionalRole&role=Polyphosphate%20kinase%20(EC%202.7.4.1)&subsystem_name=Purine_conversions) |
| Nucleosides and Nucleotides | Purines | [Purine conversions](https://rast.nmpdr.org/seedviewer.cgi?page=Subsystems&subsystem=Purine_conversions&organism=549.408) | [Inosine-guanosine kinase (EC 2.7.1.73)](https://rast.nmpdr.org/seedviewer.cgi?page=FunctionalRole&role=Inosine-guanosine%20kinase%20(EC%202.7.1.73)&subsystem_name=Purine_conversions) |
| Nucleosides and Nucleotides | Purines | [Purine conversions](https://rast.nmpdr.org/seedviewer.cgi?page=Subsystems&subsystem=Purine_conversions&organism=549.408) | [Adenine phosphoribosyltransferase (EC 2.4.2.7)](https://rast.nmpdr.org/seedviewer.cgi?page=FunctionalRole&role=Adenine%20phosphoribosyltransferase%20(EC%202.4.2.7)&subsystem_name=Purine_conversions) |
| Nucleosides and Nucleotides | Purines | [Purine conversions](https://rast.nmpdr.org/seedviewer.cgi?page=Subsystems&subsystem=Purine_conversions&organism=549.408) | [Guanine deaminase (EC 3.5.4.3)](https://rast.nmpdr.org/seedviewer.cgi?page=FunctionalRole&role=Guanine%20deaminase%20(EC%203.5.4.3)&subsystem_name=Purine_conversions) |
| Nucleosides and Nucleotides | Purines | [Purine conversions](https://rast.nmpdr.org/seedviewer.cgi?page=Subsystems&subsystem=Purine_conversions&organism=549.408) | [2',3'-cyclic-nucleotide 2'-phosphodiesterase (EC 3.1.4.16)](https://rast.nmpdr.org/seedviewer.cgi?page=FunctionalRole&role=2) |
| Nucleosides and Nucleotides | Purines | [Purine conversions](https://rast.nmpdr.org/seedviewer.cgi?page=Subsystems&subsystem=Purine_conversions&organism=549.408) | [Nucleoside diphosphate kinase (EC 2.7.4.6)](https://rast.nmpdr.org/seedviewer.cgi?page=FunctionalRole&role=Nucleoside%20diphosphate%20kinase%20(EC%202.7.4.6)&subsystem_name=Purine_conversions) |
| Nucleosides and Nucleotides | Purines | [Purine conversions](https://rast.nmpdr.org/seedviewer.cgi?page=Subsystems&subsystem=Purine_conversions&organism=549.408) | [Adenylate kinase (EC 2.7.4.3)](https://rast.nmpdr.org/seedviewer.cgi?page=FunctionalRole&role=Adenylate%20kinase%20(EC%202.7.4.3)&subsystem_name=Purine_conversions) |
| Nucleosides and Nucleotides | Purines | [Purine conversions](https://rast.nmpdr.org/seedviewer.cgi?page=Subsystems&subsystem=Purine_conversions&organism=549.408) | [Inosine-uridine preferring nucleoside hydrolase (EC 3.2.2.1)](https://rast.nmpdr.org/seedviewer.cgi?page=FunctionalRole&role=Inosine-uridine%20preferring%20nucleoside%20hydrolase%20(EC%203.2.2.1)&subsystem_name=Purine_conversions) |
| Nucleosides and Nucleotides | Purines | [Purine conversions](https://rast.nmpdr.org/seedviewer.cgi?page=Subsystems&subsystem=Purine_conversions&organism=549.408) | [AMP nucleosidase (EC 3.2.2.4)](https://rast.nmpdr.org/seedviewer.cgi?page=FunctionalRole&role=AMP%20nucleosidase%20(EC%203.2.2.4)&subsystem_name=Purine_conversions) |
| Nucleosides and Nucleotides | Purines | [Purine conversions](https://rast.nmpdr.org/seedviewer.cgi?page=Subsystems&subsystem=Purine_conversions&organism=549.408) | [Purine nucleoside phosphorylase (EC 2.4.2.1)](https://rast.nmpdr.org/seedviewer.cgi?page=FunctionalRole&role=Purine%20nucleoside%20phosphorylase%20(EC%202.4.2.1)&subsystem_name=Purine_conversions) |
| Nucleosides and Nucleotides | Purines | [Purine conversions](https://rast.nmpdr.org/seedviewer.cgi?page=Subsystems&subsystem=Purine_conversions&organism=549.408) | [5'-nucleotidase (EC 3.1.3.5)](https://rast.nmpdr.org/seedviewer.cgi?page=FunctionalRole&role=5) |
| Nucleosides and Nucleotides | Purines | [Purine conversions](https://rast.nmpdr.org/seedviewer.cgi?page=Subsystems&subsystem=Purine_conversions&organism=549.408) | [Adenosine deaminase (EC 3.5.4.4)](https://rast.nmpdr.org/seedviewer.cgi?page=FunctionalRole&role=Adenosine%20deaminase%20(EC%203.5.4.4)&subsystem_name=Purine_conversions) |
| Nucleosides and Nucleotides | Purines | [Purine conversions](https://rast.nmpdr.org/seedviewer.cgi?page=Subsystems&subsystem=Purine_conversions&organism=549.408) | [Xanthine-guanine phosphoribosyltransferase (EC 2.4.2.22)](https://rast.nmpdr.org/seedviewer.cgi?page=FunctionalRole&role=Xanthine-guanine%20phosphoribosyltransferase%20(EC%202.4.2.22)&subsystem_name=Purine_conversions) |
| Nucleosides and Nucleotides | Purines | [Purine conversions](https://rast.nmpdr.org/seedviewer.cgi?page=Subsystems&subsystem=Purine_conversions&organism=549.408) | [Guanylate kinase (EC 2.7.4.8)](https://rast.nmpdr.org/seedviewer.cgi?page=FunctionalRole&role=Guanylate%20kinase%20(EC%202.7.4.8)&subsystem_name=Purine_conversions) |
| Nucleosides and Nucleotides | Nucleosides and Nucleotides - no subcategory | [Ribonucleotide reduction](https://rast.nmpdr.org/seedviewer.cgi?page=Subsystems&subsystem=Ribonucleotide_reduction&organism=549.408) | [Glutaredoxin-like protein NrdH, required for reduction of Ribonucleotide reductase class Ib](https://rast.nmpdr.org/seedviewer.cgi?page=FunctionalRole&role=Glutaredoxin-like%20protein%20NrdH,%20required%20for%20reduction%20of%20Ribonucleotide%20reductase%20class%20Ib&subsystem_name=Ribonucleotide_reduction) |
| Nucleosides and Nucleotides | Nucleosides and Nucleotides - no subcategory | [Ribonucleotide reduction](https://rast.nmpdr.org/seedviewer.cgi?page=Subsystems&subsystem=Ribonucleotide_reduction&organism=549.408) | [Ribonucleotide reductase of class Ib (aerobic), alpha subunit (EC 1.17.4.1)](https://rast.nmpdr.org/seedviewer.cgi?page=FunctionalRole&role=Ribonucleotide%20reductase%20of%20class%20Ib%20(aerobic),%20alpha%20subunit%20(EC%201.17.4.1)&subsystem_name=Ribonucleotide_reduction) |
| Nucleosides and Nucleotides | Nucleosides and Nucleotides - no subcategory | [Ribonucleotide reduction](https://rast.nmpdr.org/seedviewer.cgi?page=Subsystems&subsystem=Ribonucleotide_reduction&organism=549.408) | [Ribonucleotide reductase of class III (anaerobic), large subunit (EC 1.17.4.2)](https://rast.nmpdr.org/seedviewer.cgi?page=FunctionalRole&role=Ribonucleotide%20reductase%20of%20class%20III%20(anaerobic),%20large%20subunit%20(EC%201.17.4.2)&subsystem_name=Ribonucleotide_reduction) |
| Nucleosides and Nucleotides | Nucleosides and Nucleotides - no subcategory | [Ribonucleotide reduction](https://rast.nmpdr.org/seedviewer.cgi?page=Subsystems&subsystem=Ribonucleotide_reduction&organism=549.408) | [Ribonucleotide reductase of class Ia (aerobic), alpha subunit (EC 1.17.4.1)](https://rast.nmpdr.org/seedviewer.cgi?page=FunctionalRole&role=Ribonucleotide%20reductase%20of%20class%20Ia%20(aerobic),%20alpha%20subunit%20(EC%201.17.4.1)&subsystem_name=Ribonucleotide_reduction) |
| Nucleosides and Nucleotides | Nucleosides and Nucleotides - no subcategory | [Ribonucleotide reduction](https://rast.nmpdr.org/seedviewer.cgi?page=Subsystems&subsystem=Ribonucleotide_reduction&organism=549.408) | [Ribonucleotide reductase of class Ib (aerobic), beta subunit (EC 1.17.4.1)](https://rast.nmpdr.org/seedviewer.cgi?page=FunctionalRole&role=Ribonucleotide%20reductase%20of%20class%20Ib%20(aerobic),%20beta%20subunit%20(EC%201.17.4.1)&subsystem_name=Ribonucleotide_reduction) |
| Nucleosides and Nucleotides | Nucleosides and Nucleotides - no subcategory | [Ribonucleotide reduction](https://rast.nmpdr.org/seedviewer.cgi?page=Subsystems&subsystem=Ribonucleotide_reduction&organism=549.408) | [Ribonucleotide reductase of class III (anaerobic), activating protein (EC 1.97.1.4)](https://rast.nmpdr.org/seedviewer.cgi?page=FunctionalRole&role=Ribonucleotide%20reductase%20of%20class%20III%20(anaerobic),%20activating%20protein%20(EC%201.97.1.4)&subsystem_name=Ribonucleotide_reduction) |
| Nucleosides and Nucleotides | Nucleosides and Nucleotides - no subcategory | [Ribonucleotide reduction](https://rast.nmpdr.org/seedviewer.cgi?page=Subsystems&subsystem=Ribonucleotide_reduction&organism=549.408) | [Ribonucleotide reduction protein NrdI](https://rast.nmpdr.org/seedviewer.cgi?page=FunctionalRole&role=Ribonucleotide%20reduction%20protein%20NrdI&subsystem_name=Ribonucleotide_reduction) |
| Nucleosides and Nucleotides | Nucleosides and Nucleotides - no subcategory | [Ribonucleotide reduction](https://rast.nmpdr.org/seedviewer.cgi?page=Subsystems&subsystem=Ribonucleotide_reduction&organism=549.408) | [Ribonucleotide reductase of class Ia (aerobic), beta subunit (EC 1.17.4.1)](https://rast.nmpdr.org/seedviewer.cgi?page=FunctionalRole&role=Ribonucleotide%20reductase%20of%20class%20Ia%20(aerobic),%20beta%20subunit%20(EC%201.17.4.1)&subsystem_name=Ribonucleotide_reduction) |
| Nucleosides and Nucleotides | Nucleosides and Nucleotides - no subcategory | [Ribonucleotide reduction](https://rast.nmpdr.org/seedviewer.cgi?page=Subsystems&subsystem=Ribonucleotide_reduction&organism=549.408) | [Ribonucleotide reductase transcriptional regulator NrdR](https://rast.nmpdr.org/seedviewer.cgi?page=FunctionalRole&role=Ribonucleotide%20reductase%20transcriptional%20regulator%20NrdR&subsystem_name=Ribonucleotide_reduction) |
| Nucleosides and Nucleotides | Nucleosides and Nucleotides - no subcategory | [Hydantoin metabolism](https://rast.nmpdr.org/seedviewer.cgi?page=Subsystems&subsystem=Hydantoin_metabolism&organism=549.408) | [N-carbamoyl-L-amino acid hydrolase (EC 3.5.1.87)](https://rast.nmpdr.org/seedviewer.cgi?page=FunctionalRole&role=N-carbamoyl-L-amino%20acid%20hydrolase%20(EC%203.5.1.87)&subsystem_name=Hydantoin_metabolism) |
| Nucleosides and Nucleotides | Nucleosides and Nucleotides - no subcategory | [Hydantoin metabolism](https://rast.nmpdr.org/seedviewer.cgi?page=Subsystems&subsystem=Hydantoin_metabolism&organism=549.408) | [N-methylhydantoinase B (EC 3.5.2.14)](https://rast.nmpdr.org/seedviewer.cgi?page=FunctionalRole&role=N-methylhydantoinase%20B%20(EC%203.5.2.14)&subsystem_name=Hydantoin_metabolism) |
| Nucleosides and Nucleotides | Nucleosides and Nucleotides - no subcategory | [Hydantoin metabolism](https://rast.nmpdr.org/seedviewer.cgi?page=Subsystems&subsystem=Hydantoin_metabolism&organism=549.408) | [N-methylhydantoinase A (EC 3.5.2.14)](https://rast.nmpdr.org/seedviewer.cgi?page=FunctionalRole&role=N-methylhydantoinase%20A%20(EC%203.5.2.14)&subsystem_name=Hydantoin_metabolism) |
| Nucleosides and Nucleotides | Nucleosides and Nucleotides - no subcategory | [Hydantoin metabolism](https://rast.nmpdr.org/seedviewer.cgi?page=Subsystems&subsystem=Hydantoin_metabolism&organism=549.408) | [Hydantoin racemase (EC 5.1.99.-)](https://rast.nmpdr.org/seedviewer.cgi?page=FunctionalRole&role=Hydantoin%20racemase%20(EC%205.1.99.-)&subsystem_name=Hydantoin_metabolism) |
| Nucleosides and Nucleotides | Nucleosides and Nucleotides - no subcategory | [Adenosyl nucleosidases](https://rast.nmpdr.org/seedviewer.cgi?page=Subsystems&subsystem=Adenosyl_nucleosidases&organism=549.408) | [S-adenosylhomocysteine nucleosidase (EC 3.2.2.9)](https://rast.nmpdr.org/seedviewer.cgi?page=FunctionalRole&role=S-adenosylhomocysteine%20nucleosidase%20(EC%203.2.2.9)&subsystem_name=Adenosyl_nucleosidases) |
| Nucleosides and Nucleotides | Nucleosides and Nucleotides - no subcategory | [Adenosyl nucleosidases](https://rast.nmpdr.org/seedviewer.cgi?page=Subsystems&subsystem=Adenosyl_nucleosidases&organism=549.408) | [Purine nucleoside phosphorylase (EC 2.4.2.1)](https://rast.nmpdr.org/seedviewer.cgi?page=FunctionalRole&role=Purine%20nucleoside%20phosphorylase%20(EC%202.4.2.1)&subsystem_name=Adenosyl_nucleosidases) |
| Nucleosides and Nucleotides | Nucleosides and Nucleotides - no subcategory | [Adenosyl nucleosidases](https://rast.nmpdr.org/seedviewer.cgi?page=Subsystems&subsystem=Adenosyl_nucleosidases&organism=549.408) | [5'-methylthioadenosine nucleosidase (EC 3.2.2.16)](https://rast.nmpdr.org/seedviewer.cgi?page=FunctionalRole&role=5) |
| Nucleosides and Nucleotides | Detoxification | [Nucleoside triphosphate pyrophosphohydrolase MazG](https://rast.nmpdr.org/seedviewer.cgi?page=Subsystems&subsystem=Nucleoside_triphosphate_pyrophosphohydrolase_MazG&organism=549.408) | [Nucleoside triphosphate pyrophosphohydrolase MazG (EC 3.6.1.8)](https://rast.nmpdr.org/seedviewer.cgi?page=FunctionalRole&role=Nucleoside%20triphosphate%20pyrophosphohydrolase%20MazG%20(EC%203.6.1.8)&subsystem_name=Nucleoside_triphosphate_pyrophosphohydrolase_MazG) |
| Nucleosides and Nucleotides | Detoxification | [Nudix proteins (nucleoside triphosphate hydrolases)](https://rast.nmpdr.org/seedviewer.cgi?page=Subsystems&subsystem=Nudix_proteins_(nucleoside_triphosphate_hydrolases)&organism=549.408) | [Putative Nudix hydrolase YfcD (EC 3.6.-.-)](https://rast.nmpdr.org/seedviewer.cgi?page=FunctionalRole&role=Putative%20Nudix%20hydrolase%20YfcD%20(EC%203.6.-.-)&subsystem_name=Nudix_proteins_(nucleoside_triphosphate_hydrolases)) |
| Nucleosides and Nucleotides | Detoxification | [Nudix proteins (nucleoside triphosphate hydrolases)](https://rast.nmpdr.org/seedviewer.cgi?page=Subsystems&subsystem=Nudix_proteins_(nucleoside_triphosphate_hydrolases)&organism=549.408) | [Hypothetical nudix hydrolase YeaB](https://rast.nmpdr.org/seedviewer.cgi?page=FunctionalRole&role=Hypothetical%20nudix%20hydrolase%20YeaB&subsystem_name=Nudix_proteins_(nucleoside_triphosphate_hydrolases)) |
| Nucleosides and Nucleotides | Detoxification | [Nudix proteins (nucleoside triphosphate hydrolases)](https://rast.nmpdr.org/seedviewer.cgi?page=Subsystems&subsystem=Nudix_proteins_(nucleoside_triphosphate_hydrolases)&organism=549.408) | [ADP-ribose pyrophosphatase (EC 3.6.1.13)](https://rast.nmpdr.org/seedviewer.cgi?page=FunctionalRole&role=ADP-ribose%20pyrophosphatase%20(EC%203.6.1.13)&subsystem_name=Nudix_proteins_(nucleoside_triphosphate_hydrolases)) |
| Nucleosides and Nucleotides | Detoxification | [Nudix proteins (nucleoside triphosphate hydrolases)](https://rast.nmpdr.org/seedviewer.cgi?page=Subsystems&subsystem=Nudix_proteins_(nucleoside_triphosphate_hydrolases)&organism=549.408) | [GDP-mannose pyrophosphatase YffH](https://rast.nmpdr.org/seedviewer.cgi?page=FunctionalRole&role=GDP-mannose%20pyrophosphatase%20YffH&subsystem_name=Nudix_proteins_(nucleoside_triphosphate_hydrolases)) |
| Nucleosides and Nucleotides | Detoxification | [Nudix proteins (nucleoside triphosphate hydrolases)](https://rast.nmpdr.org/seedviewer.cgi?page=Subsystems&subsystem=Nudix_proteins_(nucleoside_triphosphate_hydrolases)&organism=549.408) | [NADH pyrophosphatase (EC 3.6.1.22)](https://rast.nmpdr.org/seedviewer.cgi?page=FunctionalRole&role=NADH%20pyrophosphatase%20(EC%203.6.1.22)&subsystem_name=Nudix_proteins_(nucleoside_triphosphate_hydrolases)) |
| Nucleosides and Nucleotides | Detoxification | [Nudix proteins (nucleoside triphosphate hydrolases)](https://rast.nmpdr.org/seedviewer.cgi?page=Subsystems&subsystem=Nudix_proteins_(nucleoside_triphosphate_hydrolases)&organism=549.408) | [Nudix hydrolase family protein YffH](https://rast.nmpdr.org/seedviewer.cgi?page=FunctionalRole&role=Nudix%20hydrolase%20family%20protein%20YffH&subsystem_name=Nudix_proteins_(nucleoside_triphosphate_hydrolases)) |
| Nucleosides and Nucleotides | Detoxification | [Nudix proteins (nucleoside triphosphate hydrolases)](https://rast.nmpdr.org/seedviewer.cgi?page=Subsystems&subsystem=Nudix_proteins_(nucleoside_triphosphate_hydrolases)&organism=549.408) | [Deoxyuridine 5'-triphosphate nucleotidohydrolase (EC 3.6.1.23)](https://rast.nmpdr.org/seedviewer.cgi?page=FunctionalRole&role=Deoxyuridine%205) |
| Nucleosides and Nucleotides | Detoxification | [Nudix proteins (nucleoside triphosphate hydrolases)](https://rast.nmpdr.org/seedviewer.cgi?page=Subsystems&subsystem=Nudix_proteins_(nucleoside_triphosphate_hydrolases)&organism=549.408) | [ADP compounds hydrolase NudE (EC 3.6.1.-)](https://rast.nmpdr.org/seedviewer.cgi?page=FunctionalRole&role=ADP%20compounds%20hydrolase%20NudE%20(EC%203.6.1.-)&subsystem_name=Nudix_proteins_(nucleoside_triphosphate_hydrolases)) |
| Nucleosides and Nucleotides | Detoxification | [Nudix proteins (nucleoside triphosphate hydrolases)](https://rast.nmpdr.org/seedviewer.cgi?page=Subsystems&subsystem=Nudix_proteins_(nucleoside_triphosphate_hydrolases)&organism=549.408) | [Adenosine (5')-pentaphospho-(5'')-adenosine pyrophosphohydrolase (EC 3.6.1.-)](https://rast.nmpdr.org/seedviewer.cgi?page=FunctionalRole&role=Adenosine%20(5) |
| Nucleosides and Nucleotides | Detoxification | [Nudix proteins (nucleoside triphosphate hydrolases)](https://rast.nmpdr.org/seedviewer.cgi?page=Subsystems&subsystem=Nudix_proteins_(nucleoside_triphosphate_hydrolases)&organism=549.408) | [Nudix-like NDP and NTP phosphohydrolase YmfB](https://rast.nmpdr.org/seedviewer.cgi?page=FunctionalRole&role=Nudix-like%20NDP%20and%20NTP%20phosphohydrolase%20YmfB&subsystem_name=Nudix_proteins_(nucleoside_triphosphate_hydrolases)) |
| Nucleosides and Nucleotides | Detoxification | [Nudix proteins (nucleoside triphosphate hydrolases)](https://rast.nmpdr.org/seedviewer.cgi?page=Subsystems&subsystem=Nudix_proteins_(nucleoside_triphosphate_hydrolases)&organism=549.408) | [Mutator mutT protein (7,8-dihydro-8-oxoguanine-triphosphatase) (EC 3.6.1.-)](https://rast.nmpdr.org/seedviewer.cgi?page=FunctionalRole&role=Mutator%20mutT%20protein%20(7,8-dihydro-8-oxoguanine-triphosphatase)%20(EC%203.6.1.-)&subsystem_name=Nudix_proteins_(nucleoside_triphosphate_hydrolases)) |
| Nucleosides and Nucleotides | Detoxification | [Housecleaning nucleoside triphosphate pyrophosphatases](https://rast.nmpdr.org/seedviewer.cgi?page=Subsystems&subsystem=Housecleaning_nucleoside_triphosphate_pyrophosphatases&organism=549.408) | [Deoxyuridine 5'-triphosphate nucleotidohydrolase (EC 3.6.1.23)](https://rast.nmpdr.org/seedviewer.cgi?page=FunctionalRole&role=Deoxyuridine%205) |
| Nucleosides and Nucleotides | Detoxification | [Housecleaning nucleoside triphosphate pyrophosphatases](https://rast.nmpdr.org/seedviewer.cgi?page=Subsystems&subsystem=Housecleaning_nucleoside_triphosphate_pyrophosphatases&organism=549.408) | [5-nucleotidase SurE (EC 3.1.3.5)](https://rast.nmpdr.org/seedviewer.cgi?page=FunctionalRole&role=5-nucleotidase%20SurE%20(EC%203.1.3.5)&subsystem_name=Housecleaning_nucleoside_triphosphate_pyrophosphatases) |
| Nucleosides and Nucleotides | Detoxification | [Housecleaning nucleoside triphosphate pyrophosphatases](https://rast.nmpdr.org/seedviewer.cgi?page=Subsystems&subsystem=Housecleaning_nucleoside_triphosphate_pyrophosphatases&organism=549.408) | [Nucleotidase YfbR, HD superfamily](https://rast.nmpdr.org/seedviewer.cgi?page=FunctionalRole&role=Nucleotidase%20YfbR,%20HD%20superfamily&subsystem_name=Housecleaning_nucleoside_triphosphate_pyrophosphatases) |
| Nucleosides and Nucleotides | Detoxification | [Housecleaning nucleoside triphosphate pyrophosphatases](https://rast.nmpdr.org/seedviewer.cgi?page=Subsystems&subsystem=Housecleaning_nucleoside_triphosphate_pyrophosphatases&organism=549.408) | [Nucleoside 5-triphosphatase RdgB (dHAPTP, dITP, XTP-specific) (EC 3.6.1.15)](https://rast.nmpdr.org/seedviewer.cgi?page=FunctionalRole&role=Nucleoside%205-triphosphatase%20RdgB%20(dHAPTP,%20dITP,%20XTP-specific)%20(EC%203.6.1.15)&subsystem_name=Housecleaning_nucleoside_triphosphate_pyrophosphatases) |
| Nucleosides and Nucleotides | Detoxification | [Housecleaning nucleoside triphosphate pyrophosphatases](https://rast.nmpdr.org/seedviewer.cgi?page=Subsystems&subsystem=Housecleaning_nucleoside_triphosphate_pyrophosphatases&organism=549.408) | [5'-nucleotidase YjjG (EC 3.1.3.5)](https://rast.nmpdr.org/seedviewer.cgi?page=FunctionalRole&role=5) |
| Nucleosides and Nucleotides | Detoxification | [Housecleaning nucleoside triphosphate pyrophosphatases](https://rast.nmpdr.org/seedviewer.cgi?page=Subsystems&subsystem=Housecleaning_nucleoside_triphosphate_pyrophosphatases&organism=549.408) | [Inosine/xanthosine triphosphatase (EC 3.6.1.-)](https://rast.nmpdr.org/seedviewer.cgi?page=FunctionalRole&role=Inosine/xanthosine%20triphosphatase%20(EC%203.6.1.-)&subsystem_name=Housecleaning_nucleoside_triphosphate_pyrophosphatases) |
| Protein Metabolism | Protein folding | [GroEL GroES](https://rast.nmpdr.org/seedviewer.cgi?page=Subsystems&subsystem=GroEL_GroES&organism=549.408) | [Heat shock protein GrpE](https://rast.nmpdr.org/seedviewer.cgi?page=FunctionalRole&role=Heat%20shock%20protein%20GrpE&subsystem_name=GroEL_GroES) |
| Protein Metabolism | Protein folding | [GroEL GroES](https://rast.nmpdr.org/seedviewer.cgi?page=Subsystems&subsystem=GroEL_GroES&organism=549.408) | [Chaperone protein DnaK](https://rast.nmpdr.org/seedviewer.cgi?page=FunctionalRole&role=Chaperone%20protein%20DnaK&subsystem_name=GroEL_GroES) |
| Protein Metabolism | Protein folding | [GroEL GroES](https://rast.nmpdr.org/seedviewer.cgi?page=Subsystems&subsystem=GroEL_GroES&organism=549.408) | [Chaperone protein DnaJ](https://rast.nmpdr.org/seedviewer.cgi?page=FunctionalRole&role=Chaperone%20protein%20DnaJ&subsystem_name=GroEL_GroES) |
| Protein Metabolism | Protein folding | [GroEL GroES](https://rast.nmpdr.org/seedviewer.cgi?page=Subsystems&subsystem=GroEL_GroES&organism=549.408) | [Heat shock protein 60 family co-chaperone GroES](https://rast.nmpdr.org/seedviewer.cgi?page=FunctionalRole&role=Heat%20shock%20protein%2060%20family%20co-chaperone%20GroES&subsystem_name=GroEL_GroES) |
| Protein Metabolism | Protein folding | [GroEL GroES](https://rast.nmpdr.org/seedviewer.cgi?page=Subsystems&subsystem=GroEL_GroES&organism=549.408) | [Heat shock protein 60 family chaperone GroEL](https://rast.nmpdr.org/seedviewer.cgi?page=FunctionalRole&role=Heat%20shock%20protein%2060%20family%20chaperone%20GroEL&subsystem_name=GroEL_GroES) |
| Protein Metabolism | Protein folding | [Protein chaperones](https://rast.nmpdr.org/seedviewer.cgi?page=Subsystems&subsystem=Protein_chaperones&organism=549.408) | [Heat shock protein GrpE](https://rast.nmpdr.org/seedviewer.cgi?page=FunctionalRole&role=Heat%20shock%20protein%20GrpE&subsystem_name=Protein_chaperones) |
| Protein Metabolism | Protein folding | [Protein chaperones](https://rast.nmpdr.org/seedviewer.cgi?page=Subsystems&subsystem=Protein_chaperones&organism=549.408) | [Chaperone protein DnaK](https://rast.nmpdr.org/seedviewer.cgi?page=FunctionalRole&role=Chaperone%20protein%20DnaK&subsystem_name=Protein_chaperones) |
| Protein Metabolism | Protein folding | [Protein chaperones](https://rast.nmpdr.org/seedviewer.cgi?page=Subsystems&subsystem=Protein_chaperones&organism=549.408) | [Chaperone protein DnaJ](https://rast.nmpdr.org/seedviewer.cgi?page=FunctionalRole&role=Chaperone%20protein%20DnaJ&subsystem_name=Protein_chaperones) |
| Protein Metabolism | Protein folding | [Protein chaperones](https://rast.nmpdr.org/seedviewer.cgi?page=Subsystems&subsystem=Protein_chaperones&organism=549.408) | [Chaperone protein HtpG](https://rast.nmpdr.org/seedviewer.cgi?page=FunctionalRole&role=Chaperone%20protein%20HtpG&subsystem_name=Protein_chaperones) |
| Protein Metabolism | Protein folding | [Protein chaperones](https://rast.nmpdr.org/seedviewer.cgi?page=Subsystems&subsystem=Protein_chaperones&organism=549.408) | [ClpB protein](https://rast.nmpdr.org/seedviewer.cgi?page=FunctionalRole&role=ClpB%20protein&subsystem_name=Protein_chaperones) |
| Protein Metabolism | Protein folding | [Periplasmic disulfide interchange](https://rast.nmpdr.org/seedviewer.cgi?page=Subsystems&subsystem=Periplasmic_disulfide_interchange&organism=549.408) | [Periplasmic thiol:disulfide oxidoreductase DsbB, required for DsbA reoxidation](https://rast.nmpdr.org/seedviewer.cgi?page=FunctionalRole&role=Periplasmic%20thiol:disulfide%20oxidoreductase%20DsbB,%20required%20for%20DsbA%20reoxidation&subsystem_name=Periplasmic_disulfide_interchange) |
| Protein Metabolism | Protein folding | [Periplasmic disulfide interchange](https://rast.nmpdr.org/seedviewer.cgi?page=Subsystems&subsystem=Periplasmic_disulfide_interchange&organism=549.408) | [Cytochrome c-type biogenesis protein CcmG/DsbE, thiol:disulfide oxidoreductase](https://rast.nmpdr.org/seedviewer.cgi?page=FunctionalRole&role=Cytochrome%20c-type%20biogenesis%20protein%20CcmG/DsbE,%20thiol:disulfide%20oxidoreductase&subsystem_name=Periplasmic_disulfide_interchange) |
| Protein Metabolism | Protein folding | [Periplasmic disulfide interchange](https://rast.nmpdr.org/seedviewer.cgi?page=Subsystems&subsystem=Periplasmic_disulfide_interchange&organism=549.408) | [Periplasmic thiol:disulfide interchange protein DsbA](https://rast.nmpdr.org/seedviewer.cgi?page=FunctionalRole&role=Periplasmic%20thiol:disulfide%20interchange%20protein%20DsbA&subsystem_name=Periplasmic_disulfide_interchange) |
| Protein Metabolism | Protein folding | [Periplasmic disulfide interchange](https://rast.nmpdr.org/seedviewer.cgi?page=Subsystems&subsystem=Periplasmic_disulfide_interchange&organism=549.408) | [Thiol:disulfide interchange protein DsbC](https://rast.nmpdr.org/seedviewer.cgi?page=FunctionalRole&role=Thiol:disulfide%20interchange%20protein%20DsbC&subsystem_name=Periplasmic_disulfide_interchange) |
| Protein Metabolism | Protein folding | [Periplasmic disulfide interchange](https://rast.nmpdr.org/seedviewer.cgi?page=Subsystems&subsystem=Periplasmic_disulfide_interchange&organism=549.408) | [Cytochrome c-type biogenesis protein DsbD, protein-disulfide reductase (EC 1.8.1.8)](https://rast.nmpdr.org/seedviewer.cgi?page=FunctionalRole&role=Cytochrome%20c-type%20biogenesis%20protein%20DsbD,%20protein-disulfide%20reductase%20(EC%201.8.1.8)&subsystem_name=Periplasmic_disulfide_interchange) |
| Protein Metabolism | Protein folding | [Peptidyl-prolyl cis-trans isomerase](https://rast.nmpdr.org/seedviewer.cgi?page=Subsystems&subsystem=Peptidyl-prolyl_cis-trans_isomerase&organism=549.408) | [Survival protein SurA precursor (Peptidyl-prolyl cis-trans isomerase SurA) (EC 5.2.1.8)](https://rast.nmpdr.org/seedviewer.cgi?page=FunctionalRole&role=Survival%20protein%20SurA%20precursor%20(Peptidyl-prolyl%20cis-trans%20isomerase%20SurA)%20(EC%205.2.1.8)&subsystem_name=Peptidyl-prolyl_cis-trans_isomerase) |
| Protein Metabolism | Protein folding | [Peptidyl-prolyl cis-trans isomerase](https://rast.nmpdr.org/seedviewer.cgi?page=Subsystems&subsystem=Peptidyl-prolyl_cis-trans_isomerase&organism=549.408) | [Peptidyl-prolyl cis-trans isomerase PpiD (EC 5.2.1.8)](https://rast.nmpdr.org/seedviewer.cgi?page=FunctionalRole&role=Peptidyl-prolyl%20cis-trans%20isomerase%20PpiD%20(EC%205.2.1.8)&subsystem_name=Peptidyl-prolyl_cis-trans_isomerase) |
| Protein Metabolism | Protein folding | [Peptidyl-prolyl cis-trans isomerase](https://rast.nmpdr.org/seedviewer.cgi?page=Subsystems&subsystem=Peptidyl-prolyl_cis-trans_isomerase&organism=549.408) | [FKBP-type peptidyl-prolyl cis-trans isomerase SlyD (EC 5.2.1.8)](https://rast.nmpdr.org/seedviewer.cgi?page=FunctionalRole&role=FKBP-type%20peptidyl-prolyl%20cis-trans%20isomerase%20SlyD%20(EC%205.2.1.8)&subsystem_name=Peptidyl-prolyl_cis-trans_isomerase) |
| Protein Metabolism | Protein folding | [Peptidyl-prolyl cis-trans isomerase](https://rast.nmpdr.org/seedviewer.cgi?page=Subsystems&subsystem=Peptidyl-prolyl_cis-trans_isomerase&organism=549.408) | [Peptidyl-prolyl cis-trans isomerase PpiC (EC 5.2.1.8)](https://rast.nmpdr.org/seedviewer.cgi?page=FunctionalRole&role=Peptidyl-prolyl%20cis-trans%20isomerase%20PpiC%20(EC%205.2.1.8)&subsystem_name=Peptidyl-prolyl_cis-trans_isomerase) |
| Protein Metabolism | Protein folding | [Peptidyl-prolyl cis-trans isomerase](https://rast.nmpdr.org/seedviewer.cgi?page=Subsystems&subsystem=Peptidyl-prolyl_cis-trans_isomerase&organism=549.408) | [Peptidyl-prolyl cis-trans isomerase PpiA precursor (EC 5.2.1.8)](https://rast.nmpdr.org/seedviewer.cgi?page=FunctionalRole&role=Peptidyl-prolyl%20cis-trans%20isomerase%20PpiA%20precursor%20(EC%205.2.1.8)&subsystem_name=Peptidyl-prolyl_cis-trans_isomerase) |
| Protein Metabolism | Protein folding | [Peptidyl-prolyl cis-trans isomerase](https://rast.nmpdr.org/seedviewer.cgi?page=Subsystems&subsystem=Peptidyl-prolyl_cis-trans_isomerase&organism=549.408) | [FKBP-type peptidyl-prolyl cis-trans isomerase SlpA (EC 5.2.1.8)](https://rast.nmpdr.org/seedviewer.cgi?page=FunctionalRole&role=FKBP-type%20peptidyl-prolyl%20cis-trans%20isomerase%20SlpA%20(EC%205.2.1.8)&subsystem_name=Peptidyl-prolyl_cis-trans_isomerase) |
| Protein Metabolism | Protein folding | [Peptidyl-prolyl cis-trans isomerase](https://rast.nmpdr.org/seedviewer.cgi?page=Subsystems&subsystem=Peptidyl-prolyl_cis-trans_isomerase&organism=549.408) | [FKBP-type peptidyl-prolyl cis-trans isomerase FkpA precursor (EC 5.2.1.8)](https://rast.nmpdr.org/seedviewer.cgi?page=FunctionalRole&role=FKBP-type%20peptidyl-prolyl%20cis-trans%20isomerase%20FkpA%20precursor%20(EC%205.2.1.8)&subsystem_name=Peptidyl-prolyl_cis-trans_isomerase) |
| Protein Metabolism | Protein folding | [Peptidyl-prolyl cis-trans isomerase](https://rast.nmpdr.org/seedviewer.cgi?page=Subsystems&subsystem=Peptidyl-prolyl_cis-trans_isomerase&organism=549.408) | [Peptidyl-prolyl cis-trans isomerase PpiB (EC 5.2.1.8)](https://rast.nmpdr.org/seedviewer.cgi?page=FunctionalRole&role=Peptidyl-prolyl%20cis-trans%20isomerase%20PpiB%20(EC%205.2.1.8)&subsystem_name=Peptidyl-prolyl_cis-trans_isomerase) |
| Protein Metabolism | Protein folding | [Peptidyl-prolyl cis-trans isomerase](https://rast.nmpdr.org/seedviewer.cgi?page=Subsystems&subsystem=Peptidyl-prolyl_cis-trans_isomerase&organism=549.408) | [FKBP-type peptidyl-prolyl cis-trans isomerase FklB (EC 5.2.1.8)](https://rast.nmpdr.org/seedviewer.cgi?page=FunctionalRole&role=FKBP-type%20peptidyl-prolyl%20cis-trans%20isomerase%20FklB%20(EC%205.2.1.8)&subsystem_name=Peptidyl-prolyl_cis-trans_isomerase) |
| Protein Metabolism | Selenoproteins | [Selenoprotein O](https://rast.nmpdr.org/seedviewer.cgi?page=Subsystems&subsystem=Selenoprotein_O&organism=549.408) | [Selenoprotein O and cysteine-containing homologs](https://rast.nmpdr.org/seedviewer.cgi?page=FunctionalRole&role=Selenoprotein%20O%20and%20cysteine-containing%20homologs&subsystem_name=Selenoprotein_O) |
| Protein Metabolism | Protein biosynthesis | [tRNA aminoacylation, Val](https://rast.nmpdr.org/seedviewer.cgi?page=Subsystems&subsystem=tRNA_aminoacylation,_Val&organism=549.408) | [Valyl-tRNA synthetase (EC 6.1.1.9)](https://rast.nmpdr.org/seedviewer.cgi?page=FunctionalRole&role=Valyl-tRNA%20synthetase%20(EC%206.1.1.9)&subsystem_name=tRNA_aminoacylation,_Val) |
| Protein Metabolism | Protein biosynthesis | [tRNA aminoacylation, Met](https://rast.nmpdr.org/seedviewer.cgi?page=Subsystems&subsystem=tRNA_aminoacylation,_Met&organism=549.408) | [Methionyl-tRNA synthetase (EC 6.1.1.10)](https://rast.nmpdr.org/seedviewer.cgi?page=FunctionalRole&role=Methionyl-tRNA%20synthetase%20(EC%206.1.1.10)&subsystem_name=tRNA_aminoacylation,_Met) |
| Protein Metabolism | Protein biosynthesis | [tRNA aminoacylation, Ile](https://rast.nmpdr.org/seedviewer.cgi?page=Subsystems&subsystem=tRNA_aminoacylation,_Ile&organism=549.408) | [Isoleucyl-tRNA synthetase (EC 6.1.1.5)](https://rast.nmpdr.org/seedviewer.cgi?page=FunctionalRole&role=Isoleucyl-tRNA%20synthetase%20(EC%206.1.1.5)&subsystem_name=tRNA_aminoacylation,_Ile) |
| Protein Metabolism | Protein biosynthesis | [tRNA aminoacylation, Arg](https://rast.nmpdr.org/seedviewer.cgi?page=Subsystems&subsystem=tRNA_aminoacylation,_Arg&organism=549.408) | [Arginyl-tRNA synthetase (EC 6.1.1.19)](https://rast.nmpdr.org/seedviewer.cgi?page=FunctionalRole&role=Arginyl-tRNA%20synthetase%20(EC%206.1.1.19)&subsystem_name=tRNA_aminoacylation,_Arg) |
| Protein Metabolism | Protein biosynthesis | [Translation initiation factors bacterial](https://rast.nmpdr.org/seedviewer.cgi?page=Subsystems&subsystem=Translation_initiation_factors_bacterial&organism=549.408) | [Translation initiation factor SUI1-related protein](https://rast.nmpdr.org/seedviewer.cgi?page=FunctionalRole&role=Translation%20initiation%20factor%20SUI1-related%20protein&subsystem_name=Translation_initiation_factors_bacterial) |
| Protein Metabolism | Protein biosynthesis | [Translation initiation factors bacterial](https://rast.nmpdr.org/seedviewer.cgi?page=Subsystems&subsystem=Translation_initiation_factors_bacterial&organism=549.408) | [Translation initiation factor 1](https://rast.nmpdr.org/seedviewer.cgi?page=FunctionalRole&role=Translation%20initiation%20factor%201&subsystem_name=Translation_initiation_factors_bacterial) |
| Protein Metabolism | Protein biosynthesis | [Translation initiation factors bacterial](https://rast.nmpdr.org/seedviewer.cgi?page=Subsystems&subsystem=Translation_initiation_factors_bacterial&organism=549.408) | [Methionyl-tRNA formyltransferase (EC 2.1.2.9)](https://rast.nmpdr.org/seedviewer.cgi?page=FunctionalRole&role=Methionyl-tRNA%20formyltransferase%20(EC%202.1.2.9)&subsystem_name=Translation_initiation_factors_bacterial) |
| Protein Metabolism | Protein biosynthesis | [Translation initiation factors bacterial](https://rast.nmpdr.org/seedviewer.cgi?page=Subsystems&subsystem=Translation_initiation_factors_bacterial&organism=549.408) | [Translation initiation factor 3](https://rast.nmpdr.org/seedviewer.cgi?page=FunctionalRole&role=Translation%20initiation%20factor%203&subsystem_name=Translation_initiation_factors_bacterial) |
| Protein Metabolism | Protein biosynthesis | [Translation initiation factors bacterial](https://rast.nmpdr.org/seedviewer.cgi?page=Subsystems&subsystem=Translation_initiation_factors_bacterial&organism=549.408) | [Translation initiation factor 2](https://rast.nmpdr.org/seedviewer.cgi?page=FunctionalRole&role=Translation%20initiation%20factor%202&subsystem_name=Translation_initiation_factors_bacterial) |
| Protein Metabolism | Protein biosynthesis | [Translation initiation factors bacterial](https://rast.nmpdr.org/seedviewer.cgi?page=Subsystems&subsystem=Translation_initiation_factors_bacterial&organism=549.408) | [Ribosome-binding factor A](https://rast.nmpdr.org/seedviewer.cgi?page=FunctionalRole&role=Ribosome-binding%20factor%20A&subsystem_name=Translation_initiation_factors_bacterial) |
| Protein Metabolism | Protein biosynthesis | [Ribosome SSU bacterial](https://rast.nmpdr.org/seedviewer.cgi?page=Subsystems&subsystem=Ribosome_SSU_bacterial&organism=549.408) | [SSU ribosomal protein S11p (S14e)](https://rast.nmpdr.org/seedviewer.cgi?page=FunctionalRole&role=SSU%20ribosomal%20protein%20S11p%20(S14e)&subsystem_name=Ribosome_SSU_bacterial) |
| Protein Metabolism | Protein biosynthesis | [Ribosome SSU bacterial](https://rast.nmpdr.org/seedviewer.cgi?page=Subsystems&subsystem=Ribosome_SSU_bacterial&organism=549.408) | [SSU ribosomal protein S1p](https://rast.nmpdr.org/seedviewer.cgi?page=FunctionalRole&role=SSU%20ribosomal%20protein%20S1p&subsystem_name=Ribosome_SSU_bacterial) |
| Protein Metabolism | Protein biosynthesis | [Ribosome SSU bacterial](https://rast.nmpdr.org/seedviewer.cgi?page=Subsystems&subsystem=Ribosome_SSU_bacterial&organism=549.408) | [SSU ribosomal protein S6p](https://rast.nmpdr.org/seedviewer.cgi?page=FunctionalRole&role=SSU%20ribosomal%20protein%20S6p&subsystem_name=Ribosome_SSU_bacterial) |
| Protein Metabolism | Protein biosynthesis | [Ribosome SSU bacterial](https://rast.nmpdr.org/seedviewer.cgi?page=Subsystems&subsystem=Ribosome_SSU_bacterial&organism=549.408) | [SSU ribosomal protein S15p (S13e)](https://rast.nmpdr.org/seedviewer.cgi?page=FunctionalRole&role=SSU%20ribosomal%20protein%20S15p%20(S13e)&subsystem_name=Ribosome_SSU_bacterial) |
| Protein Metabolism | Protein biosynthesis | [Ribosome SSU bacterial](https://rast.nmpdr.org/seedviewer.cgi?page=Subsystems&subsystem=Ribosome_SSU_bacterial&organism=549.408) | [SSU ribosomal protein S14p (S29e)](https://rast.nmpdr.org/seedviewer.cgi?page=FunctionalRole&role=SSU%20ribosomal%20protein%20S14p%20(S29e)&subsystem_name=Ribosome_SSU_bacterial) |
| Protein Metabolism | Protein biosynthesis | [Ribosome SSU bacterial](https://rast.nmpdr.org/seedviewer.cgi?page=Subsystems&subsystem=Ribosome_SSU_bacterial&organism=549.408) | [SSU ribosomal protein S5p (S2e)](https://rast.nmpdr.org/seedviewer.cgi?page=FunctionalRole&role=SSU%20ribosomal%20protein%20S5p%20(S2e)&subsystem_name=Ribosome_SSU_bacterial) |
| Protein Metabolism | Protein biosynthesis | [Ribosome SSU bacterial](https://rast.nmpdr.org/seedviewer.cgi?page=Subsystems&subsystem=Ribosome_SSU_bacterial&organism=549.408) | [SSU ribosomal protein S18p](https://rast.nmpdr.org/seedviewer.cgi?page=FunctionalRole&role=SSU%20ribosomal%20protein%20S18p&subsystem_name=Ribosome_SSU_bacterial) |
| Protein Metabolism | Protein biosynthesis | [Ribosome SSU bacterial](https://rast.nmpdr.org/seedviewer.cgi?page=Subsystems&subsystem=Ribosome_SSU_bacterial&organism=549.408) | [SSU ribosomal protein S17p (S11e)](https://rast.nmpdr.org/seedviewer.cgi?page=FunctionalRole&role=SSU%20ribosomal%20protein%20S17p%20(S11e)&subsystem_name=Ribosome_SSU_bacterial) |
| Protein Metabolism | Protein biosynthesis | [Ribosome SSU bacterial](https://rast.nmpdr.org/seedviewer.cgi?page=Subsystems&subsystem=Ribosome_SSU_bacterial&organism=549.408) | [SSU ribosomal protein S20p](https://rast.nmpdr.org/seedviewer.cgi?page=FunctionalRole&role=SSU%20ribosomal%20protein%20S20p&subsystem_name=Ribosome_SSU_bacterial) |
| Protein Metabolism | Protein biosynthesis | [Ribosome SSU bacterial](https://rast.nmpdr.org/seedviewer.cgi?page=Subsystems&subsystem=Ribosome_SSU_bacterial&organism=549.408) | [SSU ribosomal protein S12p (S23e)](https://rast.nmpdr.org/seedviewer.cgi?page=FunctionalRole&role=SSU%20ribosomal%20protein%20S12p%20(S23e)&subsystem_name=Ribosome_SSU_bacterial) |
| Protein Metabolism | Protein biosynthesis | [Ribosome SSU bacterial](https://rast.nmpdr.org/seedviewer.cgi?page=Subsystems&subsystem=Ribosome_SSU_bacterial&organism=549.408) | [SSU ribosomal protein S9p (S16e)](https://rast.nmpdr.org/seedviewer.cgi?page=FunctionalRole&role=SSU%20ribosomal%20protein%20S9p%20(S16e)&subsystem_name=Ribosome_SSU_bacterial) |
| Protein Metabolism | Protein biosynthesis | [Ribosome SSU bacterial](https://rast.nmpdr.org/seedviewer.cgi?page=Subsystems&subsystem=Ribosome_SSU_bacterial&organism=549.408) | [SSU ribosomal protein S8p (S15Ae)](https://rast.nmpdr.org/seedviewer.cgi?page=FunctionalRole&role=SSU%20ribosomal%20protein%20S8p%20(S15Ae)&subsystem_name=Ribosome_SSU_bacterial) |
| Protein Metabolism | Protein biosynthesis | [Ribosome SSU bacterial](https://rast.nmpdr.org/seedviewer.cgi?page=Subsystems&subsystem=Ribosome_SSU_bacterial&organism=549.408) | [SSU ribosomal protein S4p (S9e)](https://rast.nmpdr.org/seedviewer.cgi?page=FunctionalRole&role=SSU%20ribosomal%20protein%20S4p%20(S9e)&subsystem_name=Ribosome_SSU_bacterial) |
| Protein Metabolism | Protein biosynthesis | [Ribosome SSU bacterial](https://rast.nmpdr.org/seedviewer.cgi?page=Subsystems&subsystem=Ribosome_SSU_bacterial&organism=549.408) | [SSU ribosomal protein S14p (S29e), zinc-independent](https://rast.nmpdr.org/seedviewer.cgi?page=FunctionalRole&role=SSU%20ribosomal%20protein%20S14p%20(S29e),%20zinc-independent&subsystem_name=Ribosome_SSU_bacterial) |
| Protein Metabolism | Protein biosynthesis | [Ribosome SSU bacterial](https://rast.nmpdr.org/seedviewer.cgi?page=Subsystems&subsystem=Ribosome_SSU_bacterial&organism=549.408) | [SSU ribosomal protein S19p (S15e)](https://rast.nmpdr.org/seedviewer.cgi?page=FunctionalRole&role=SSU%20ribosomal%20protein%20S19p%20(S15e)&subsystem_name=Ribosome_SSU_bacterial) |
| Protein Metabolism | Protein biosynthesis | [Ribosome SSU bacterial](https://rast.nmpdr.org/seedviewer.cgi?page=Subsystems&subsystem=Ribosome_SSU_bacterial&organism=549.408) | [SSU ribosomal protein S10p (S20e)](https://rast.nmpdr.org/seedviewer.cgi?page=FunctionalRole&role=SSU%20ribosomal%20protein%20S10p%20(S20e)&subsystem_name=Ribosome_SSU_bacterial) |
| Protein Metabolism | Protein biosynthesis | [Ribosome SSU bacterial](https://rast.nmpdr.org/seedviewer.cgi?page=Subsystems&subsystem=Ribosome_SSU_bacterial&organism=549.408) | [SSU ribosomal protein S16p](https://rast.nmpdr.org/seedviewer.cgi?page=FunctionalRole&role=SSU%20ribosomal%20protein%20S16p&subsystem_name=Ribosome_SSU_bacterial) |
| Protein Metabolism | Protein biosynthesis | [Ribosome SSU bacterial](https://rast.nmpdr.org/seedviewer.cgi?page=Subsystems&subsystem=Ribosome_SSU_bacterial&organism=549.408) | [SSU ribosomal protein S3p (S3e)](https://rast.nmpdr.org/seedviewer.cgi?page=FunctionalRole&role=SSU%20ribosomal%20protein%20S3p%20(S3e)&subsystem_name=Ribosome_SSU_bacterial) |
| Protein Metabolism | Protein biosynthesis | [Ribosome SSU bacterial](https://rast.nmpdr.org/seedviewer.cgi?page=Subsystems&subsystem=Ribosome_SSU_bacterial&organism=549.408) | [SSU ribosomal protein S18p, zinc-independent](https://rast.nmpdr.org/seedviewer.cgi?page=FunctionalRole&role=SSU%20ribosomal%20protein%20S18p,%20zinc-independent&subsystem_name=Ribosome_SSU_bacterial) |
| Protein Metabolism | Protein biosynthesis | [Ribosome SSU bacterial](https://rast.nmpdr.org/seedviewer.cgi?page=Subsystems&subsystem=Ribosome_SSU_bacterial&organism=549.408) | [SSU ribosomal protein S7p (S5e)](https://rast.nmpdr.org/seedviewer.cgi?page=FunctionalRole&role=SSU%20ribosomal%20protein%20S7p%20(S5e)&subsystem_name=Ribosome_SSU_bacterial) |
| Protein Metabolism | Protein biosynthesis | [Ribosome SSU bacterial](https://rast.nmpdr.org/seedviewer.cgi?page=Subsystems&subsystem=Ribosome_SSU_bacterial&organism=549.408) | [SSU ribosomal protein S2p (SAe)](https://rast.nmpdr.org/seedviewer.cgi?page=FunctionalRole&role=SSU%20ribosomal%20protein%20S2p%20(SAe)&subsystem_name=Ribosome_SSU_bacterial) |
| Protein Metabolism | Protein biosynthesis | [Ribosome SSU bacterial](https://rast.nmpdr.org/seedviewer.cgi?page=Subsystems&subsystem=Ribosome_SSU_bacterial&organism=549.408) | [SSU ribosomal protein S21p](https://rast.nmpdr.org/seedviewer.cgi?page=FunctionalRole&role=SSU%20ribosomal%20protein%20S21p&subsystem_name=Ribosome_SSU_bacterial) |
| Protein Metabolism | Protein biosynthesis | [Ribosome SSU bacterial](https://rast.nmpdr.org/seedviewer.cgi?page=Subsystems&subsystem=Ribosome_SSU_bacterial&organism=549.408) | [SSU ribosomal protein S13p (S18e)](https://rast.nmpdr.org/seedviewer.cgi?page=FunctionalRole&role=SSU%20ribosomal%20protein%20S13p%20(S18e)&subsystem_name=Ribosome_SSU_bacterial) |
| Protein Metabolism | Protein biosynthesis | [tRNA aminoacylation, Gly](https://rast.nmpdr.org/seedviewer.cgi?page=Subsystems&subsystem=tRNA_aminoacylation,_Gly&organism=549.408) | [Glycyl-tRNA synthetase alpha chain (EC 6.1.1.14)](https://rast.nmpdr.org/seedviewer.cgi?page=FunctionalRole&role=Glycyl-tRNA%20synthetase%20alpha%20chain%20(EC%206.1.1.14)&subsystem_name=tRNA_aminoacylation,_Gly) |
| Protein Metabolism | Protein biosynthesis | [tRNA aminoacylation, Gly](https://rast.nmpdr.org/seedviewer.cgi?page=Subsystems&subsystem=tRNA_aminoacylation,_Gly&organism=549.408) | [Glycyl-tRNA synthetase beta chain (EC 6.1.1.14)](https://rast.nmpdr.org/seedviewer.cgi?page=FunctionalRole&role=Glycyl-tRNA%20synthetase%20beta%20chain%20(EC%206.1.1.14)&subsystem_name=tRNA_aminoacylation,_Gly) |
| Protein Metabolism | Protein biosynthesis | [tRNA aminoacylation, Ala](https://rast.nmpdr.org/seedviewer.cgi?page=Subsystems&subsystem=tRNA_aminoacylation,_Ala&organism=549.408) | [Alanyl-tRNA synthetase (EC 6.1.1.7)](https://rast.nmpdr.org/seedviewer.cgi?page=FunctionalRole&role=Alanyl-tRNA%20synthetase%20(EC%206.1.1.7)&subsystem_name=tRNA_aminoacylation,_Ala) |
| Protein Metabolism | Protein biosynthesis | [Ribosome activity modulation](https://rast.nmpdr.org/seedviewer.cgi?page=Subsystems&subsystem=Ribosome_activity_modulation&organism=549.408) | [Ribosome hibernation protein YhbH](https://rast.nmpdr.org/seedviewer.cgi?page=FunctionalRole&role=Ribosome%20hibernation%20protein%20YhbH&subsystem_name=Ribosome_activity_modulation) |
| Protein Metabolism | Protein biosynthesis | [Ribosome activity modulation](https://rast.nmpdr.org/seedviewer.cgi?page=Subsystems&subsystem=Ribosome_activity_modulation&organism=549.408) | [Ribosome hibernation protein YfiA](https://rast.nmpdr.org/seedviewer.cgi?page=FunctionalRole&role=Ribosome%20hibernation%20protein%20YfiA&subsystem_name=Ribosome_activity_modulation) |
| Protein Metabolism | Protein biosynthesis | [tRNA aminoacylation, Trp](https://rast.nmpdr.org/seedviewer.cgi?page=Subsystems&subsystem=tRNA_aminoacylation,_Trp&organism=549.408) | [Tryptophanyl-tRNA synthetase (EC 6.1.1.2)](https://rast.nmpdr.org/seedviewer.cgi?page=FunctionalRole&role=Tryptophanyl-tRNA%20synthetase%20(EC%206.1.1.2)&subsystem_name=tRNA_aminoacylation,_Trp) |
| Protein Metabolism | Protein biosynthesis | [Ribosome biogenesis bacterial](https://rast.nmpdr.org/seedviewer.cgi?page=Subsystems&subsystem=Ribosome_biogenesis_bacterial&organism=549.408) | [16S rRNA processing protein RimM](https://rast.nmpdr.org/seedviewer.cgi?page=FunctionalRole&role=16S%20rRNA%20processing%20protein%20RimM&subsystem_name=Ribosome_biogenesis_bacterial) |
| Protein Metabolism | Protein biosynthesis | [Ribosome biogenesis bacterial](https://rast.nmpdr.org/seedviewer.cgi?page=Subsystems&subsystem=Ribosome_biogenesis_bacterial&organism=549.408) | [Ribosomal large subunit pseudouridine synthase A (EC 4.2.1.70)](https://rast.nmpdr.org/seedviewer.cgi?page=FunctionalRole&role=Ribosomal%20large%20subunit%20pseudouridine%20synthase%20A%20(EC%204.2.1.70)&subsystem_name=Ribosome_biogenesis_bacterial) |
| Protein Metabolism | Protein biosynthesis | [Ribosome biogenesis bacterial](https://rast.nmpdr.org/seedviewer.cgi?page=Subsystems&subsystem=Ribosome_biogenesis_bacterial&organism=549.408) | [TsaB protein, required for threonylcarbamoyladenosine (t(6)A) formation in tRNA](https://rast.nmpdr.org/seedviewer.cgi?page=FunctionalRole&role=TsaB%20protein,%20required%20for%20threonylcarbamoyladenosine%20(t(6)A)%20formation%20in%20tRNA&subsystem_name=Ribosome_biogenesis_bacterial) |
| Protein Metabolism | Protein biosynthesis | [Ribosome biogenesis bacterial](https://rast.nmpdr.org/seedviewer.cgi?page=Subsystems&subsystem=Ribosome_biogenesis_bacterial&organism=549.408) | [Ribosomal large subunit pseudouridine synthase D (EC 4.2.1.70)](https://rast.nmpdr.org/seedviewer.cgi?page=FunctionalRole&role=Ribosomal%20large%20subunit%20pseudouridine%20synthase%20D%20(EC%204.2.1.70)&subsystem_name=Ribosome_biogenesis_bacterial) |
| Protein Metabolism | Protein biosynthesis | [Ribosome biogenesis bacterial](https://rast.nmpdr.org/seedviewer.cgi?page=Subsystems&subsystem=Ribosome_biogenesis_bacterial&organism=549.408) | [Ribosomal large subunit pseudouridine synthase C (EC 4.2.1.70)](https://rast.nmpdr.org/seedviewer.cgi?page=FunctionalRole&role=Ribosomal%20large%20subunit%20pseudouridine%20synthase%20C%20(EC%204.2.1.70)&subsystem_name=Ribosome_biogenesis_bacterial) |
| Protein Metabolism | Protein biosynthesis | [Ribosome biogenesis bacterial](https://rast.nmpdr.org/seedviewer.cgi?page=Subsystems&subsystem=Ribosome_biogenesis_bacterial&organism=549.408) | [LSU m5C1962 methyltransferase RlmI](https://rast.nmpdr.org/seedviewer.cgi?page=FunctionalRole&role=LSU%20m5C1962%20methyltransferase%20RlmI&subsystem_name=Ribosome_biogenesis_bacterial) |
| Protein Metabolism | Protein biosynthesis | [Ribosome biogenesis bacterial](https://rast.nmpdr.org/seedviewer.cgi?page=Subsystems&subsystem=Ribosome_biogenesis_bacterial&organism=549.408) | [SSU rRNA (adenine(1518)-N(6)/adenine(1519)-N(6))-dimethyltransferase (EC 2.1.1.182)](https://rast.nmpdr.org/seedviewer.cgi?page=FunctionalRole&role=SSU%20rRNA%20(adenine(1518)-N(6)/adenine(1519)-N(6))-dimethyltransferase%20(EC%202.1.1.182)&subsystem_name=Ribosome_biogenesis_bacterial) |
| Protein Metabolism | Protein biosynthesis | [Ribosome biogenesis bacterial](https://rast.nmpdr.org/seedviewer.cgi?page=Subsystems&subsystem=Ribosome_biogenesis_bacterial&organism=549.408) | [tRNA (Guanine37-N1) -methyltransferase (EC 2.1.1.31)](https://rast.nmpdr.org/seedviewer.cgi?page=FunctionalRole&role=tRNA%20(Guanine37-N1)%20-methyltransferase%20(EC%202.1.1.31)&subsystem_name=Ribosome_biogenesis_bacterial) |
| Protein Metabolism | Protein biosynthesis | [Ribosome biogenesis bacterial](https://rast.nmpdr.org/seedviewer.cgi?page=Subsystems&subsystem=Ribosome_biogenesis_bacterial&organism=549.408) | [Ribosomal-protein-S18p-alanine acetyltransferase (EC 2.3.1.-)](https://rast.nmpdr.org/seedviewer.cgi?page=FunctionalRole&role=Ribosomal-protein-S18p-alanine%20acetyltransferase%20(EC%202.3.1.-)&subsystem_name=Ribosome_biogenesis_bacterial) |
| Protein Metabolism | Protein biosynthesis | [Ribosome biogenesis bacterial](https://rast.nmpdr.org/seedviewer.cgi?page=Subsystems&subsystem=Ribosome_biogenesis_bacterial&organism=549.408) | [Ribosomal protein S6 glutaminyl transferase](https://rast.nmpdr.org/seedviewer.cgi?page=FunctionalRole&role=Ribosomal%20protein%20S6%20glutaminyl%20transferase&subsystem_name=Ribosome_biogenesis_bacterial) |
| Protein Metabolism | Protein biosynthesis | [Ribosome biogenesis bacterial](https://rast.nmpdr.org/seedviewer.cgi?page=Subsystems&subsystem=Ribosome_biogenesis_bacterial&organism=549.408) | [LSU m3Psi1915 methyltransferase RlmH](https://rast.nmpdr.org/seedviewer.cgi?page=FunctionalRole&role=LSU%20m3Psi1915%20methyltransferase%20RlmH&subsystem_name=Ribosome_biogenesis_bacterial) |
| Protein Metabolism | Protein biosynthesis | [Ribosome biogenesis bacterial](https://rast.nmpdr.org/seedviewer.cgi?page=Subsystems&subsystem=Ribosome_biogenesis_bacterial&organism=549.408) | [Ribosomal protein L11 methyltransferase (EC 2.1.1.-)](https://rast.nmpdr.org/seedviewer.cgi?page=FunctionalRole&role=Ribosomal%20protein%20L11%20methyltransferase%20(EC%202.1.1.-)&subsystem_name=Ribosome_biogenesis_bacterial) |
| Protein Metabolism | Protein biosynthesis | [Ribosome biogenesis bacterial](https://rast.nmpdr.org/seedviewer.cgi?page=Subsystems&subsystem=Ribosome_biogenesis_bacterial&organism=549.408) | [Ribosomal-protein-S5p-alanine acetyltransferase](https://rast.nmpdr.org/seedviewer.cgi?page=FunctionalRole&role=Ribosomal-protein-S5p-alanine%20acetyltransferase&subsystem_name=Ribosome_biogenesis_bacterial) |
| Protein Metabolism | Protein biosynthesis | [Ribosome biogenesis bacterial](https://rast.nmpdr.org/seedviewer.cgi?page=Subsystems&subsystem=Ribosome_biogenesis_bacterial&organism=549.408) | [Ribonuclease E (EC 3.1.26.12)](https://rast.nmpdr.org/seedviewer.cgi?page=FunctionalRole&role=Ribonuclease%20E%20(EC%203.1.26.12)&subsystem_name=Ribosome_biogenesis_bacterial) |
| Protein Metabolism | Protein biosynthesis | [Ribosome LSU bacterial](https://rast.nmpdr.org/seedviewer.cgi?page=Subsystems&subsystem=Ribosome_LSU_bacterial&organism=549.408) | [LSU ribosomal protein L18p (L5e)](https://rast.nmpdr.org/seedviewer.cgi?page=FunctionalRole&role=LSU%20ribosomal%20protein%20L18p%20(L5e)&subsystem_name=Ribosome_LSU_bacterial) |
| Protein Metabolism | Protein biosynthesis | [Ribosome LSU bacterial](https://rast.nmpdr.org/seedviewer.cgi?page=Subsystems&subsystem=Ribosome_LSU_bacterial&organism=549.408) | [LSU ribosomal protein L11p (L12e)](https://rast.nmpdr.org/seedviewer.cgi?page=FunctionalRole&role=LSU%20ribosomal%20protein%20L11p%20(L12e)&subsystem_name=Ribosome_LSU_bacterial) |
| Protein Metabolism | Protein biosynthesis | [Ribosome LSU bacterial](https://rast.nmpdr.org/seedviewer.cgi?page=Subsystems&subsystem=Ribosome_LSU_bacterial&organism=549.408) | [LSU ribosomal protein L5p (L11e)](https://rast.nmpdr.org/seedviewer.cgi?page=FunctionalRole&role=LSU%20ribosomal%20protein%20L5p%20(L11e)&subsystem_name=Ribosome_LSU_bacterial) |
| Protein Metabolism | Protein biosynthesis | [Ribosome LSU bacterial](https://rast.nmpdr.org/seedviewer.cgi?page=Subsystems&subsystem=Ribosome_LSU_bacterial&organism=549.408) | [LSU ribosomal protein L23p (L23Ae)](https://rast.nmpdr.org/seedviewer.cgi?page=FunctionalRole&role=LSU%20ribosomal%20protein%20L23p%20(L23Ae)&subsystem_name=Ribosome_LSU_bacterial) |
| Protein Metabolism | Protein biosynthesis | [Ribosome LSU bacterial](https://rast.nmpdr.org/seedviewer.cgi?page=Subsystems&subsystem=Ribosome_LSU_bacterial&organism=549.408) | [LSU ribosomal protein L14p (L23e)](https://rast.nmpdr.org/seedviewer.cgi?page=FunctionalRole&role=LSU%20ribosomal%20protein%20L14p%20(L23e)&subsystem_name=Ribosome_LSU_bacterial) |
| Protein Metabolism | Protein biosynthesis | [Ribosome LSU bacterial](https://rast.nmpdr.org/seedviewer.cgi?page=Subsystems&subsystem=Ribosome_LSU_bacterial&organism=549.408) | [LSU ribosomal protein L31p, zinc-dependent](https://rast.nmpdr.org/seedviewer.cgi?page=FunctionalRole&role=LSU%20ribosomal%20protein%20L31p,%20zinc-dependent&subsystem_name=Ribosome_LSU_bacterial) |
| Protein Metabolism | Protein biosynthesis | [Ribosome LSU bacterial](https://rast.nmpdr.org/seedviewer.cgi?page=Subsystems&subsystem=Ribosome_LSU_bacterial&organism=549.408) | [LSU ribosomal protein L2p (L8e)](https://rast.nmpdr.org/seedviewer.cgi?page=FunctionalRole&role=LSU%20ribosomal%20protein%20L2p%20(L8e)&subsystem_name=Ribosome_LSU_bacterial) |
| Protein Metabolism | Protein biosynthesis | [Ribosome LSU bacterial](https://rast.nmpdr.org/seedviewer.cgi?page=Subsystems&subsystem=Ribosome_LSU_bacterial&organism=549.408) | [LSU ribosomal protein L31p](https://rast.nmpdr.org/seedviewer.cgi?page=FunctionalRole&role=LSU%20ribosomal%20protein%20L31p&subsystem_name=Ribosome_LSU_bacterial) |
| Protein Metabolism | Protein biosynthesis | [Ribosome LSU bacterial](https://rast.nmpdr.org/seedviewer.cgi?page=Subsystems&subsystem=Ribosome_LSU_bacterial&organism=549.408) | [LSU ribosomal protein L6p (L9e)](https://rast.nmpdr.org/seedviewer.cgi?page=FunctionalRole&role=LSU%20ribosomal%20protein%20L6p%20(L9e)&subsystem_name=Ribosome_LSU_bacterial) |
| Protein Metabolism | Protein biosynthesis | [Ribosome LSU bacterial](https://rast.nmpdr.org/seedviewer.cgi?page=Subsystems&subsystem=Ribosome_LSU_bacterial&organism=549.408) | [LSU ribosomal protein L21p](https://rast.nmpdr.org/seedviewer.cgi?page=FunctionalRole&role=LSU%20ribosomal%20protein%20L21p&subsystem_name=Ribosome_LSU_bacterial) |
| Protein Metabolism | Protein biosynthesis | [Ribosome LSU bacterial](https://rast.nmpdr.org/seedviewer.cgi?page=Subsystems&subsystem=Ribosome_LSU_bacterial&organism=549.408) | [LSU ribosomal protein L24p (L26e)](https://rast.nmpdr.org/seedviewer.cgi?page=FunctionalRole&role=LSU%20ribosomal%20protein%20L24p%20(L26e)&subsystem_name=Ribosome_LSU_bacterial) |
| Protein Metabolism | Protein biosynthesis | [Ribosome LSU bacterial](https://rast.nmpdr.org/seedviewer.cgi?page=Subsystems&subsystem=Ribosome_LSU_bacterial&organism=549.408) | [LSU ribosomal protein L29p (L35e)](https://rast.nmpdr.org/seedviewer.cgi?page=FunctionalRole&role=LSU%20ribosomal%20protein%20L29p%20(L35e)&subsystem_name=Ribosome_LSU_bacterial) |
| Protein Metabolism | Protein biosynthesis | [Ribosome LSU bacterial](https://rast.nmpdr.org/seedviewer.cgi?page=Subsystems&subsystem=Ribosome_LSU_bacterial&organism=549.408) | [LSU ribosomal protein L30p (L7e)](https://rast.nmpdr.org/seedviewer.cgi?page=FunctionalRole&role=LSU%20ribosomal%20protein%20L30p%20(L7e)&subsystem_name=Ribosome_LSU_bacterial) |
| Protein Metabolism | Protein biosynthesis | [Ribosome LSU bacterial](https://rast.nmpdr.org/seedviewer.cgi?page=Subsystems&subsystem=Ribosome_LSU_bacterial&organism=549.408) | [LSU ribosomal protein L1p (L10Ae)](https://rast.nmpdr.org/seedviewer.cgi?page=FunctionalRole&role=LSU%20ribosomal%20protein%20L1p%20(L10Ae)&subsystem_name=Ribosome_LSU_bacterial) |
| Protein Metabolism | Protein biosynthesis | [Ribosome LSU bacterial](https://rast.nmpdr.org/seedviewer.cgi?page=Subsystems&subsystem=Ribosome_LSU_bacterial&organism=549.408) | [LSU ribosomal protein L16p (L10e)](https://rast.nmpdr.org/seedviewer.cgi?page=FunctionalRole&role=LSU%20ribosomal%20protein%20L16p%20(L10e)&subsystem_name=Ribosome_LSU_bacterial) |
| Protein Metabolism | Protein biosynthesis | [Ribosome LSU bacterial](https://rast.nmpdr.org/seedviewer.cgi?page=Subsystems&subsystem=Ribosome_LSU_bacterial&organism=549.408) | [LSU ribosomal protein L25p](https://rast.nmpdr.org/seedviewer.cgi?page=FunctionalRole&role=LSU%20ribosomal%20protein%20L25p&subsystem_name=Ribosome_LSU_bacterial) |
| Protein Metabolism | Protein biosynthesis | [Ribosome LSU bacterial](https://rast.nmpdr.org/seedviewer.cgi?page=Subsystems&subsystem=Ribosome_LSU_bacterial&organism=549.408) | [LSU ribosomal protein L3p (L3e)](https://rast.nmpdr.org/seedviewer.cgi?page=FunctionalRole&role=LSU%20ribosomal%20protein%20L3p%20(L3e)&subsystem_name=Ribosome_LSU_bacterial) |
| Protein Metabolism | Protein biosynthesis | [Ribosome LSU bacterial](https://rast.nmpdr.org/seedviewer.cgi?page=Subsystems&subsystem=Ribosome_LSU_bacterial&organism=549.408) | [LSU ribosomal protein L15p (L27Ae)](https://rast.nmpdr.org/seedviewer.cgi?page=FunctionalRole&role=LSU%20ribosomal%20protein%20L15p%20(L27Ae)&subsystem_name=Ribosome_LSU_bacterial) |
| Protein Metabolism | Protein biosynthesis | [Ribosome LSU bacterial](https://rast.nmpdr.org/seedviewer.cgi?page=Subsystems&subsystem=Ribosome_LSU_bacterial&organism=549.408) | [LSU ribosomal protein L33p, zinc-independent](https://rast.nmpdr.org/seedviewer.cgi?page=FunctionalRole&role=LSU%20ribosomal%20protein%20L33p,%20zinc-independent&subsystem_name=Ribosome_LSU_bacterial) |
| Protein Metabolism | Protein biosynthesis | [Ribosome LSU bacterial](https://rast.nmpdr.org/seedviewer.cgi?page=Subsystems&subsystem=Ribosome_LSU_bacterial&organism=549.408) | [LSU ribosomal protein L13p (L13Ae)](https://rast.nmpdr.org/seedviewer.cgi?page=FunctionalRole&role=LSU%20ribosomal%20protein%20L13p%20(L13Ae)&subsystem_name=Ribosome_LSU_bacterial) |
| Protein Metabolism | Protein biosynthesis | [Ribosome LSU bacterial](https://rast.nmpdr.org/seedviewer.cgi?page=Subsystems&subsystem=Ribosome_LSU_bacterial&organism=549.408) | [LSU ribosomal protein L22p (L17e)](https://rast.nmpdr.org/seedviewer.cgi?page=FunctionalRole&role=LSU%20ribosomal%20protein%20L22p%20(L17e)&subsystem_name=Ribosome_LSU_bacterial) |
| Protein Metabolism | Protein biosynthesis | [Ribosome LSU bacterial](https://rast.nmpdr.org/seedviewer.cgi?page=Subsystems&subsystem=Ribosome_LSU_bacterial&organism=549.408) | [LSU ribosomal protein L34p](https://rast.nmpdr.org/seedviewer.cgi?page=FunctionalRole&role=LSU%20ribosomal%20protein%20L34p&subsystem_name=Ribosome_LSU_bacterial) |
| Protein Metabolism | Protein biosynthesis | [Ribosome LSU bacterial](https://rast.nmpdr.org/seedviewer.cgi?page=Subsystems&subsystem=Ribosome_LSU_bacterial&organism=549.408) | [LSU ribosomal protein L10p (P0)](https://rast.nmpdr.org/seedviewer.cgi?page=FunctionalRole&role=LSU%20ribosomal%20protein%20L10p%20(P0)&subsystem_name=Ribosome_LSU_bacterial) |
| Protein Metabolism | Protein biosynthesis | [Ribosome LSU bacterial](https://rast.nmpdr.org/seedviewer.cgi?page=Subsystems&subsystem=Ribosome_LSU_bacterial&organism=549.408) | [LSU ribosomal protein L9p](https://rast.nmpdr.org/seedviewer.cgi?page=FunctionalRole&role=LSU%20ribosomal%20protein%20L9p&subsystem_name=Ribosome_LSU_bacterial) |
| Protein Metabolism | Protein biosynthesis | [Ribosome LSU bacterial](https://rast.nmpdr.org/seedviewer.cgi?page=Subsystems&subsystem=Ribosome_LSU_bacterial&organism=549.408) | [LSU ribosomal protein L7/L12 (P1/P2)](https://rast.nmpdr.org/seedviewer.cgi?page=FunctionalRole&role=LSU%20ribosomal%20protein%20L7/L12%20(P1/P2)&subsystem_name=Ribosome_LSU_bacterial) |
| Protein Metabolism | Protein biosynthesis | [Ribosome LSU bacterial](https://rast.nmpdr.org/seedviewer.cgi?page=Subsystems&subsystem=Ribosome_LSU_bacterial&organism=549.408) | [LSU ribosomal protein L28p](https://rast.nmpdr.org/seedviewer.cgi?page=FunctionalRole&role=LSU%20ribosomal%20protein%20L28p&subsystem_name=Ribosome_LSU_bacterial) |
| Protein Metabolism | Protein biosynthesis | [Ribosome LSU bacterial](https://rast.nmpdr.org/seedviewer.cgi?page=Subsystems&subsystem=Ribosome_LSU_bacterial&organism=549.408) | [LSU ribosomal protein L19p](https://rast.nmpdr.org/seedviewer.cgi?page=FunctionalRole&role=LSU%20ribosomal%20protein%20L19p&subsystem_name=Ribosome_LSU_bacterial) |
| Protein Metabolism | Protein biosynthesis | [Ribosome LSU bacterial](https://rast.nmpdr.org/seedviewer.cgi?page=Subsystems&subsystem=Ribosome_LSU_bacterial&organism=549.408) | [LSU ribosomal protein L35p](https://rast.nmpdr.org/seedviewer.cgi?page=FunctionalRole&role=LSU%20ribosomal%20protein%20L35p&subsystem_name=Ribosome_LSU_bacterial) |
| Protein Metabolism | Protein biosynthesis | [Ribosome LSU bacterial](https://rast.nmpdr.org/seedviewer.cgi?page=Subsystems&subsystem=Ribosome_LSU_bacterial&organism=549.408) | [LSU ribosomal protein L36p](https://rast.nmpdr.org/seedviewer.cgi?page=FunctionalRole&role=LSU%20ribosomal%20protein%20L36p&subsystem_name=Ribosome_LSU_bacterial) |
| Protein Metabolism | Protein biosynthesis | [Ribosome LSU bacterial](https://rast.nmpdr.org/seedviewer.cgi?page=Subsystems&subsystem=Ribosome_LSU_bacterial&organism=549.408) | [LSU ribosomal protein L20p](https://rast.nmpdr.org/seedviewer.cgi?page=FunctionalRole&role=LSU%20ribosomal%20protein%20L20p&subsystem_name=Ribosome_LSU_bacterial) |
| Protein Metabolism | Protein biosynthesis | [Ribosome LSU bacterial](https://rast.nmpdr.org/seedviewer.cgi?page=Subsystems&subsystem=Ribosome_LSU_bacterial&organism=549.408) | [LSU ribosomal protein L32p](https://rast.nmpdr.org/seedviewer.cgi?page=FunctionalRole&role=LSU%20ribosomal%20protein%20L32p&subsystem_name=Ribosome_LSU_bacterial) |
| Protein Metabolism | Protein biosynthesis | [Ribosome LSU bacterial](https://rast.nmpdr.org/seedviewer.cgi?page=Subsystems&subsystem=Ribosome_LSU_bacterial&organism=549.408) | [LSU ribosomal protein L33p](https://rast.nmpdr.org/seedviewer.cgi?page=FunctionalRole&role=LSU%20ribosomal%20protein%20L33p&subsystem_name=Ribosome_LSU_bacterial) |
| Protein Metabolism | Protein biosynthesis | [Ribosome LSU bacterial](https://rast.nmpdr.org/seedviewer.cgi?page=Subsystems&subsystem=Ribosome_LSU_bacterial&organism=549.408) | [LSU ribosomal protein L17p](https://rast.nmpdr.org/seedviewer.cgi?page=FunctionalRole&role=LSU%20ribosomal%20protein%20L17p&subsystem_name=Ribosome_LSU_bacterial) |
| Protein Metabolism | Protein biosynthesis | [Ribosome LSU bacterial](https://rast.nmpdr.org/seedviewer.cgi?page=Subsystems&subsystem=Ribosome_LSU_bacterial&organism=549.408) | [LSU ribosomal protein L27p](https://rast.nmpdr.org/seedviewer.cgi?page=FunctionalRole&role=LSU%20ribosomal%20protein%20L27p&subsystem_name=Ribosome_LSU_bacterial) |
| Protein Metabolism | Protein biosynthesis | [Ribosome LSU bacterial](https://rast.nmpdr.org/seedviewer.cgi?page=Subsystems&subsystem=Ribosome_LSU_bacterial&organism=549.408) | [LSU ribosomal protein L4p (L1e)](https://rast.nmpdr.org/seedviewer.cgi?page=FunctionalRole&role=LSU%20ribosomal%20protein%20L4p%20(L1e)&subsystem_name=Ribosome_LSU_bacterial) |
| Protein Metabolism | Protein biosynthesis | [Programmed frameshift](https://rast.nmpdr.org/seedviewer.cgi?page=Subsystems&subsystem=Programmed_frameshift&organism=549.408) | [programmed frameshift-containing](https://rast.nmpdr.org/seedviewer.cgi?page=FunctionalRole&role=programmed%20frameshift-containing&subsystem_name=Programmed_frameshift) |
| Protein Metabolism | Protein biosynthesis | [Programmed frameshift](https://rast.nmpdr.org/seedviewer.cgi?page=Subsystems&subsystem=Programmed_frameshift&organism=549.408) | [Peptide chain release factor 2](https://rast.nmpdr.org/seedviewer.cgi?page=FunctionalRole&role=Peptide%20chain%20release%20factor%202&subsystem_name=Programmed_frameshift) |
| Protein Metabolism | Protein biosynthesis | [tRNA aminoacylation, Cys](https://rast.nmpdr.org/seedviewer.cgi?page=Subsystems&subsystem=tRNA_aminoacylation,_Cys&organism=549.408) | [Cysteinyl-tRNA synthetase (EC 6.1.1.16)](https://rast.nmpdr.org/seedviewer.cgi?page=FunctionalRole&role=Cysteinyl-tRNA%20synthetase%20(EC%206.1.1.16)&subsystem_name=tRNA_aminoacylation,_Cys) |
| Protein Metabolism | Protein biosynthesis | [Translation termination factors bacterial](https://rast.nmpdr.org/seedviewer.cgi?page=Subsystems&subsystem=Translation_termination_factors_bacterial&organism=549.408) | [Protein-N(5)-glutamine methyltransferase PrmC, methylates polypeptide chain release factors RF1 and RF2](https://rast.nmpdr.org/seedviewer.cgi?page=FunctionalRole&role=Protein-N(5)-glutamine%20methyltransferase%20PrmC,%20methylates%20polypeptide%20chain%20release%20factors%20RF1%20and%20RF2&subsystem_name=Translation_termination_factors_bacterial) |
| Protein Metabolism | Protein biosynthesis | [Translation termination factors bacterial](https://rast.nmpdr.org/seedviewer.cgi?page=Subsystems&subsystem=Translation_termination_factors_bacterial&organism=549.408) | [Ribosome recycling factor](https://rast.nmpdr.org/seedviewer.cgi?page=FunctionalRole&role=Ribosome%20recycling%20factor&subsystem_name=Translation_termination_factors_bacterial) |
| Protein Metabolism | Protein biosynthesis | [Translation termination factors bacterial](https://rast.nmpdr.org/seedviewer.cgi?page=Subsystems&subsystem=Translation_termination_factors_bacterial&organism=549.408) | [Peptidyl-tRNA hydrolase (EC 3.1.1.29)](https://rast.nmpdr.org/seedviewer.cgi?page=FunctionalRole&role=Peptidyl-tRNA%20hydrolase%20(EC%203.1.1.29)&subsystem_name=Translation_termination_factors_bacterial) |
| Protein Metabolism | Protein biosynthesis | [Translation termination factors bacterial](https://rast.nmpdr.org/seedviewer.cgi?page=Subsystems&subsystem=Translation_termination_factors_bacterial&organism=549.408) | [Peptide deformylase (EC 3.5.1.88)](https://rast.nmpdr.org/seedviewer.cgi?page=FunctionalRole&role=Peptide%20deformylase%20(EC%203.5.1.88)&subsystem_name=Translation_termination_factors_bacterial) |
| Protein Metabolism | Protein biosynthesis | [Translation termination factors bacterial](https://rast.nmpdr.org/seedviewer.cgi?page=Subsystems&subsystem=Translation_termination_factors_bacterial&organism=549.408) | [Hypothetical protein YaeJ with similarity to translation release factor](https://rast.nmpdr.org/seedviewer.cgi?page=FunctionalRole&role=Hypothetical%20protein%20YaeJ%20with%20similarity%20to%20translation%20release%20factor&subsystem_name=Translation_termination_factors_bacterial) |
| Protein Metabolism | Protein biosynthesis | [Translation termination factors bacterial](https://rast.nmpdr.org/seedviewer.cgi?page=Subsystems&subsystem=Translation_termination_factors_bacterial&organism=549.408) | [Peptide chain release factor 3](https://rast.nmpdr.org/seedviewer.cgi?page=FunctionalRole&role=Peptide%20chain%20release%20factor%203&subsystem_name=Translation_termination_factors_bacterial) |
| Protein Metabolism | Protein biosynthesis | [Translation termination factors bacterial](https://rast.nmpdr.org/seedviewer.cgi?page=Subsystems&subsystem=Translation_termination_factors_bacterial&organism=549.408) | [tmRNA-binding protein SmpB](https://rast.nmpdr.org/seedviewer.cgi?page=FunctionalRole&role=tmRNA-binding%20protein%20SmpB&subsystem_name=Translation_termination_factors_bacterial) |
| Protein Metabolism | Protein biosynthesis | [Translation termination factors bacterial](https://rast.nmpdr.org/seedviewer.cgi?page=Subsystems&subsystem=Translation_termination_factors_bacterial&organism=549.408) | [Peptide chain release factor 1](https://rast.nmpdr.org/seedviewer.cgi?page=FunctionalRole&role=Peptide%20chain%20release%20factor%201&subsystem_name=Translation_termination_factors_bacterial) |
| Protein Metabolism | Protein biosynthesis | [Translation termination factors bacterial](https://rast.nmpdr.org/seedviewer.cgi?page=Subsystems&subsystem=Translation_termination_factors_bacterial&organism=549.408) | [Methionine aminopeptidase (EC 3.4.11.18)](https://rast.nmpdr.org/seedviewer.cgi?page=FunctionalRole&role=Methionine%20aminopeptidase%20(EC%203.4.11.18)&subsystem_name=Translation_termination_factors_bacterial) |
| Protein Metabolism | Protein biosynthesis | [Translation termination factors bacterial](https://rast.nmpdr.org/seedviewer.cgi?page=Subsystems&subsystem=Translation_termination_factors_bacterial&organism=549.408) | [Peptide chain release factor 2](https://rast.nmpdr.org/seedviewer.cgi?page=FunctionalRole&role=Peptide%20chain%20release%20factor%202&subsystem_name=Translation_termination_factors_bacterial) |
| Protein Metabolism | Protein biosynthesis | [tRNA aminoacylation, His](https://rast.nmpdr.org/seedviewer.cgi?page=Subsystems&subsystem=tRNA_aminoacylation,_His&organism=549.408) | [Histidyl-tRNA synthetase (EC 6.1.1.21)](https://rast.nmpdr.org/seedviewer.cgi?page=FunctionalRole&role=Histidyl-tRNA%20synthetase%20(EC%206.1.1.21)&subsystem_name=tRNA_aminoacylation,_His) |
| Protein Metabolism | Protein biosynthesis | [Universal GTPases](https://rast.nmpdr.org/seedviewer.cgi?page=Subsystems&subsystem=Universal_GTPases&organism=549.408) | [Signal recognition particle, subunit Ffh SRP54 (TC 3.A.5.1.1)](https://rast.nmpdr.org/seedviewer.cgi?page=FunctionalRole&role=Signal%20recognition%20particle,%20subunit%20Ffh%20SRP54%20(TC%203.A.5.1.1)&subsystem_name=Universal_GTPases) |
| Protein Metabolism | Protein biosynthesis | [Universal GTPases](https://rast.nmpdr.org/seedviewer.cgi?page=Subsystems&subsystem=Universal_GTPases&organism=549.408) | [GTP-binding protein EngA](https://rast.nmpdr.org/seedviewer.cgi?page=FunctionalRole&role=GTP-binding%20protein%20EngA&subsystem_name=Universal_GTPases) |
| Protein Metabolism | Protein biosynthesis | [Universal GTPases](https://rast.nmpdr.org/seedviewer.cgi?page=Subsystems&subsystem=Universal_GTPases&organism=549.408) | [GTP-binding protein HflX](https://rast.nmpdr.org/seedviewer.cgi?page=FunctionalRole&role=GTP-binding%20protein%20HflX&subsystem_name=Universal_GTPases) |
| Protein Metabolism | Protein biosynthesis | [Universal GTPases](https://rast.nmpdr.org/seedviewer.cgi?page=Subsystems&subsystem=Universal_GTPases&organism=549.408) | [Translation elongation factor G](https://rast.nmpdr.org/seedviewer.cgi?page=FunctionalRole&role=Translation%20elongation%20factor%20G&subsystem_name=Universal_GTPases) |
| Protein Metabolism | Protein biosynthesis | [Universal GTPases](https://rast.nmpdr.org/seedviewer.cgi?page=Subsystems&subsystem=Universal_GTPases&organism=549.408) | [Signal recognition particle receptor protein FtsY (=alpha subunit) (TC 3.A.5.1.1)](https://rast.nmpdr.org/seedviewer.cgi?page=FunctionalRole&role=Signal%20recognition%20particle%20receptor%20protein%20FtsY%20(=alpha%20subunit)%20(TC%203.A.5.1.1)&subsystem_name=Universal_GTPases) |
| Protein Metabolism | Protein biosynthesis | [Universal GTPases](https://rast.nmpdr.org/seedviewer.cgi?page=Subsystems&subsystem=Universal_GTPases&organism=549.408) | [GTP-binding protein Era](https://rast.nmpdr.org/seedviewer.cgi?page=FunctionalRole&role=GTP-binding%20protein%20Era&subsystem_name=Universal_GTPases) |
| Protein Metabolism | Protein biosynthesis | [Universal GTPases](https://rast.nmpdr.org/seedviewer.cgi?page=Subsystems&subsystem=Universal_GTPases&organism=549.408) | [GTP-binding protein TypA/BipA](https://rast.nmpdr.org/seedviewer.cgi?page=FunctionalRole&role=GTP-binding%20protein%20TypA/BipA&subsystem_name=Universal_GTPases) |
| Protein Metabolism | Protein biosynthesis | [Universal GTPases](https://rast.nmpdr.org/seedviewer.cgi?page=Subsystems&subsystem=Universal_GTPases&organism=549.408) | [GTPase and tRNA-U34 5-formylation enzyme TrmE](https://rast.nmpdr.org/seedviewer.cgi?page=FunctionalRole&role=GTPase%20and%20tRNA-U34%205-formylation%20enzyme%20TrmE&subsystem_name=Universal_GTPases) |
| Protein Metabolism | Protein biosynthesis | [Universal GTPases](https://rast.nmpdr.org/seedviewer.cgi?page=Subsystems&subsystem=Universal_GTPases&organism=549.408) | [Translation elongation factor LepA](https://rast.nmpdr.org/seedviewer.cgi?page=FunctionalRole&role=Translation%20elongation%20factor%20LepA&subsystem_name=Universal_GTPases) |
| Protein Metabolism | Protein biosynthesis | [Universal GTPases](https://rast.nmpdr.org/seedviewer.cgi?page=Subsystems&subsystem=Universal_GTPases&organism=549.408) | [Translation elongation factor Tu](https://rast.nmpdr.org/seedviewer.cgi?page=FunctionalRole&role=Translation%20elongation%20factor%20Tu&subsystem_name=Universal_GTPases) |
| Protein Metabolism | Protein biosynthesis | [Universal GTPases](https://rast.nmpdr.org/seedviewer.cgi?page=Subsystems&subsystem=Universal_GTPases&organism=549.408) | [GTP-binding and nucleic acid-binding protein YchF](https://rast.nmpdr.org/seedviewer.cgi?page=FunctionalRole&role=GTP-binding%20and%20nucleic%20acid-binding%20protein%20YchF&subsystem_name=Universal_GTPases) |
| Protein Metabolism | Protein biosynthesis | [Universal GTPases](https://rast.nmpdr.org/seedviewer.cgi?page=Subsystems&subsystem=Universal_GTPases&organism=549.408) | [Translation initiation factor 2](https://rast.nmpdr.org/seedviewer.cgi?page=FunctionalRole&role=Translation%20initiation%20factor%202&subsystem_name=Universal_GTPases) |
| Protein Metabolism | Protein biosynthesis | [Universal GTPases](https://rast.nmpdr.org/seedviewer.cgi?page=Subsystems&subsystem=Universal_GTPases&organism=549.408) | [Ribosome small subunit-stimulated GTPase EngC](https://rast.nmpdr.org/seedviewer.cgi?page=FunctionalRole&role=Ribosome%20small%20subunit-stimulated%20GTPase%20EngC&subsystem_name=Universal_GTPases) |
| Protein Metabolism | Protein biosynthesis | [Universal GTPases](https://rast.nmpdr.org/seedviewer.cgi?page=Subsystems&subsystem=Universal_GTPases&organism=549.408) | [GTP-binding protein EngB](https://rast.nmpdr.org/seedviewer.cgi?page=FunctionalRole&role=GTP-binding%20protein%20EngB&subsystem_name=Universal_GTPases) |
| Protein Metabolism | Protein biosynthesis | [Universal GTPases](https://rast.nmpdr.org/seedviewer.cgi?page=Subsystems&subsystem=Universal_GTPases&organism=549.408) | [GTP-binding protein Obg](https://rast.nmpdr.org/seedviewer.cgi?page=FunctionalRole&role=GTP-binding%20protein%20Obg&subsystem_name=Universal_GTPases) |
| Protein Metabolism | Protein biosynthesis | [tRNA aminoacylation, Asp and Asn](https://rast.nmpdr.org/seedviewer.cgi?page=Subsystems&subsystem=tRNA_aminoacylation,_Asp_and_Asn&organism=549.408) | [Aspartyl-tRNA synthetase (EC 6.1.1.12)](https://rast.nmpdr.org/seedviewer.cgi?page=FunctionalRole&role=Aspartyl-tRNA%20synthetase%20(EC%206.1.1.12)&subsystem_name=tRNA_aminoacylation,_Asp_and_Asn) |
| Protein Metabolism | Protein biosynthesis | [tRNA aminoacylation, Asp and Asn](https://rast.nmpdr.org/seedviewer.cgi?page=Subsystems&subsystem=tRNA_aminoacylation,_Asp_and_Asn&organism=549.408) | [Asparaginyl-tRNA synthetase (EC 6.1.1.22)](https://rast.nmpdr.org/seedviewer.cgi?page=FunctionalRole&role=Asparaginyl-tRNA%20synthetase%20(EC%206.1.1.22)&subsystem_name=tRNA_aminoacylation,_Asp_and_Asn) |
| Protein Metabolism | Protein biosynthesis | [tRNA aminoacylation, Asp and Asn](https://rast.nmpdr.org/seedviewer.cgi?page=Subsystems&subsystem=tRNA_aminoacylation,_Asp_and_Asn&organism=549.408) | [Aspartyl-tRNA(Asn) amidotransferase subunit A (EC 6.3.5.6)](https://rast.nmpdr.org/seedviewer.cgi?page=FunctionalRole&role=Aspartyl-tRNA(Asn)%20amidotransferase%20subunit%20A%20(EC%206.3.5.6)&subsystem_name=tRNA_aminoacylation,_Asp_and_Asn) |
| Protein Metabolism | Protein biosynthesis | [Translation elongation factors bacterial](https://rast.nmpdr.org/seedviewer.cgi?page=Subsystems&subsystem=Translation_elongation_factors_bacterial&organism=549.408) | [Translation elongation factor G](https://rast.nmpdr.org/seedviewer.cgi?page=FunctionalRole&role=Translation%20elongation%20factor%20G&subsystem_name=Translation_elongation_factors_bacterial) |
| Protein Metabolism | Protein biosynthesis | [Translation elongation factors bacterial](https://rast.nmpdr.org/seedviewer.cgi?page=Subsystems&subsystem=Translation_elongation_factors_bacterial&organism=549.408) | [Translation elongation factor LepA](https://rast.nmpdr.org/seedviewer.cgi?page=FunctionalRole&role=Translation%20elongation%20factor%20LepA&subsystem_name=Translation_elongation_factors_bacterial) |
| Protein Metabolism | Protein biosynthesis | [Translation elongation factors bacterial](https://rast.nmpdr.org/seedviewer.cgi?page=Subsystems&subsystem=Translation_elongation_factors_bacterial&organism=549.408) | [Translation elongation factor Tu](https://rast.nmpdr.org/seedviewer.cgi?page=FunctionalRole&role=Translation%20elongation%20factor%20Tu&subsystem_name=Translation_elongation_factors_bacterial) |
| Protein Metabolism | Protein biosynthesis | [Translation elongation factors bacterial](https://rast.nmpdr.org/seedviewer.cgi?page=Subsystems&subsystem=Translation_elongation_factors_bacterial&organism=549.408) | [Translation elongation factor P-related protein](https://rast.nmpdr.org/seedviewer.cgi?page=FunctionalRole&role=Translation%20elongation%20factor%20P-related%20protein&subsystem_name=Translation_elongation_factors_bacterial) |
| Protein Metabolism | Protein biosynthesis | [Translation elongation factors bacterial](https://rast.nmpdr.org/seedviewer.cgi?page=Subsystems&subsystem=Translation_elongation_factors_bacterial&organism=549.408) | [Translation elongation factor Ts](https://rast.nmpdr.org/seedviewer.cgi?page=FunctionalRole&role=Translation%20elongation%20factor%20Ts&subsystem_name=Translation_elongation_factors_bacterial) |
| Protein Metabolism | Protein biosynthesis | [Translation elongation factors bacterial](https://rast.nmpdr.org/seedviewer.cgi?page=Subsystems&subsystem=Translation_elongation_factors_bacterial&organism=549.408) | [Translation elongation factor P](https://rast.nmpdr.org/seedviewer.cgi?page=FunctionalRole&role=Translation%20elongation%20factor%20P&subsystem_name=Translation_elongation_factors_bacterial) |
| Protein Metabolism | Protein biosynthesis | [tRNAs](https://rast.nmpdr.org/seedviewer.cgi?page=Subsystems&subsystem=tRNAs&organism=549.408) | [tRNA-Phe-GAA](https://rast.nmpdr.org/seedviewer.cgi?page=FunctionalRole&role=tRNA-Phe-GAA&subsystem_name=tRNAs) |
| Protein Metabolism | Protein biosynthesis | [tRNAs](https://rast.nmpdr.org/seedviewer.cgi?page=Subsystems&subsystem=tRNAs&organism=549.408) | [tRNA-Leu-CAG](https://rast.nmpdr.org/seedviewer.cgi?page=FunctionalRole&role=tRNA-Leu-CAG&subsystem_name=tRNAs) |
| Protein Metabolism | Protein biosynthesis | [tRNAs](https://rast.nmpdr.org/seedviewer.cgi?page=Subsystems&subsystem=tRNAs&organism=549.408) | [tRNA-Pro-GGG](https://rast.nmpdr.org/seedviewer.cgi?page=FunctionalRole&role=tRNA-Pro-GGG&subsystem_name=tRNAs) |
| Protein Metabolism | Protein biosynthesis | [tRNAs](https://rast.nmpdr.org/seedviewer.cgi?page=Subsystems&subsystem=tRNAs&organism=549.408) | [tRNA-Arg-ACG](https://rast.nmpdr.org/seedviewer.cgi?page=FunctionalRole&role=tRNA-Arg-ACG&subsystem_name=tRNAs) |
| Protein Metabolism | Protein biosynthesis | [tRNAs](https://rast.nmpdr.org/seedviewer.cgi?page=Subsystems&subsystem=tRNAs&organism=549.408) | [tRNA-Val-GAC](https://rast.nmpdr.org/seedviewer.cgi?page=FunctionalRole&role=tRNA-Val-GAC&subsystem_name=tRNAs) |
| Protein Metabolism | Protein biosynthesis | [tRNAs](https://rast.nmpdr.org/seedviewer.cgi?page=Subsystems&subsystem=tRNAs&organism=549.408) | [tRNA-Trp-CCA](https://rast.nmpdr.org/seedviewer.cgi?page=FunctionalRole&role=tRNA-Trp-CCA&subsystem_name=tRNAs) |
| Protein Metabolism | Protein biosynthesis | [tRNAs](https://rast.nmpdr.org/seedviewer.cgi?page=Subsystems&subsystem=tRNAs&organism=549.408) | [tRNA-Leu-CAA](https://rast.nmpdr.org/seedviewer.cgi?page=FunctionalRole&role=tRNA-Leu-CAA&subsystem_name=tRNAs) |
| Protein Metabolism | Protein biosynthesis | [tRNAs](https://rast.nmpdr.org/seedviewer.cgi?page=Subsystems&subsystem=tRNAs&organism=549.408) | [tRNA-Gly-GCC](https://rast.nmpdr.org/seedviewer.cgi?page=FunctionalRole&role=tRNA-Gly-GCC&subsystem_name=tRNAs) |
| Protein Metabolism | Protein biosynthesis | [tRNAs](https://rast.nmpdr.org/seedviewer.cgi?page=Subsystems&subsystem=tRNAs&organism=549.408) | [tRNA-Leu-GAG](https://rast.nmpdr.org/seedviewer.cgi?page=FunctionalRole&role=tRNA-Leu-GAG&subsystem_name=tRNAs) |
| Protein Metabolism | Protein biosynthesis | [tRNAs](https://rast.nmpdr.org/seedviewer.cgi?page=Subsystems&subsystem=tRNAs&organism=549.408) | [tRNA-Arg-CCG](https://rast.nmpdr.org/seedviewer.cgi?page=FunctionalRole&role=tRNA-Arg-CCG&subsystem_name=tRNAs) |
| Protein Metabolism | Protein biosynthesis | [tRNAs](https://rast.nmpdr.org/seedviewer.cgi?page=Subsystems&subsystem=tRNAs&organism=549.408) | [tRNA-Ser-GGA](https://rast.nmpdr.org/seedviewer.cgi?page=FunctionalRole&role=tRNA-Ser-GGA&subsystem_name=tRNAs) |
| Protein Metabolism | Protein biosynthesis | [tRNAs](https://rast.nmpdr.org/seedviewer.cgi?page=Subsystems&subsystem=tRNAs&organism=549.408) | [tRNA-Ala-GGC](https://rast.nmpdr.org/seedviewer.cgi?page=FunctionalRole&role=tRNA-Ala-GGC&subsystem_name=tRNAs) |
| Protein Metabolism | Protein biosynthesis | [tRNAs](https://rast.nmpdr.org/seedviewer.cgi?page=Subsystems&subsystem=tRNAs&organism=549.408) | [tRNA-Cys-GCA](https://rast.nmpdr.org/seedviewer.cgi?page=FunctionalRole&role=tRNA-Cys-GCA&subsystem_name=tRNAs) |
| Protein Metabolism | Protein biosynthesis | [tRNA aminoacylation, Lys](https://rast.nmpdr.org/seedviewer.cgi?page=Subsystems&subsystem=tRNA_aminoacylation,_Lys&organism=549.408) | [Lysyl-tRNA synthetase (class II) (EC 6.1.1.6)](https://rast.nmpdr.org/seedviewer.cgi?page=FunctionalRole&role=Lysyl-tRNA%20synthetase%20(class%20II)%20(EC%206.1.1.6)&subsystem_name=tRNA_aminoacylation,_Lys) |
| Protein Metabolism | Protein biosynthesis | [tRNA aminoacylation, Thr](https://rast.nmpdr.org/seedviewer.cgi?page=Subsystems&subsystem=tRNA_aminoacylation,_Thr&organism=549.408) | [Threonyl-tRNA synthetase (EC 6.1.1.3)](https://rast.nmpdr.org/seedviewer.cgi?page=FunctionalRole&role=Threonyl-tRNA%20synthetase%20(EC%206.1.1.3)&subsystem_name=tRNA_aminoacylation,_Thr) |
| Protein Metabolism | Protein biosynthesis | [tRNA aminoacylation, Pro](https://rast.nmpdr.org/seedviewer.cgi?page=Subsystems&subsystem=tRNA_aminoacylation,_Pro&organism=549.408) | [Prolyl-tRNA synthetase (EC 6.1.1.15), bacterial type](https://rast.nmpdr.org/seedviewer.cgi?page=FunctionalRole&role=Prolyl-tRNA%20synthetase%20(EC%206.1.1.15),%20bacterial%20type&subsystem_name=tRNA_aminoacylation,_Pro) |
| Protein Metabolism | Protein biosynthesis | [tRNA aminoacylation, Pro](https://rast.nmpdr.org/seedviewer.cgi?page=Subsystems&subsystem=tRNA_aminoacylation,_Pro&organism=549.408) | [Cys-tRNA(Pro) deacylase YbaK](https://rast.nmpdr.org/seedviewer.cgi?page=FunctionalRole&role=Cys-tRNA(Pro)%20deacylase%20YbaK&subsystem_name=tRNA_aminoacylation,_Pro) |
| Protein Metabolism | Protein biosynthesis | [tRNA aminoacylation, Pro](https://rast.nmpdr.org/seedviewer.cgi?page=Subsystems&subsystem=tRNA_aminoacylation,_Pro&organism=549.408) | [tRNA proofreading protein STM4549](https://rast.nmpdr.org/seedviewer.cgi?page=FunctionalRole&role=tRNA%20proofreading%20protein%20STM4549&subsystem_name=tRNA_aminoacylation,_Pro) |
| Protein Metabolism | Protein biosynthesis | [Translation elongation factor G family](https://rast.nmpdr.org/seedviewer.cgi?page=Subsystems&subsystem=Translation_elongation_factor_G_family&organism=549.408) | [Translation elongation factor G](https://rast.nmpdr.org/seedviewer.cgi?page=FunctionalRole&role=Translation%20elongation%20factor%20G&subsystem_name=Translation_elongation_factor_G_family) |
| Protein Metabolism | Protein biosynthesis | [tRNA aminoacylation, Glu and Gln](https://rast.nmpdr.org/seedviewer.cgi?page=Subsystems&subsystem=tRNA_aminoacylation,_Glu_and_Gln&organism=549.408) | [Glutamyl-tRNA(Gln) amidotransferase subunit A (EC 6.3.5.7)](https://rast.nmpdr.org/seedviewer.cgi?page=FunctionalRole&role=Glutamyl-tRNA(Gln)%20amidotransferase%20subunit%20A%20(EC%206.3.5.7)&subsystem_name=tRNA_aminoacylation,_Glu_and_Gln) |
| Protein Metabolism | Protein biosynthesis | [tRNA aminoacylation, Glu and Gln](https://rast.nmpdr.org/seedviewer.cgi?page=Subsystems&subsystem=tRNA_aminoacylation,_Glu_and_Gln&organism=549.408) | [Glutamyl-tRNA synthetase (EC 6.1.1.17)](https://rast.nmpdr.org/seedviewer.cgi?page=FunctionalRole&role=Glutamyl-tRNA%20synthetase%20(EC%206.1.1.17)&subsystem_name=tRNA_aminoacylation,_Glu_and_Gln) |
| Protein Metabolism | Protein biosynthesis | [tRNA aminoacylation, Glu and Gln](https://rast.nmpdr.org/seedviewer.cgi?page=Subsystems&subsystem=tRNA_aminoacylation,_Glu_and_Gln&organism=549.408) | [Glutaminyl-tRNA synthetase (EC 6.1.1.18)](https://rast.nmpdr.org/seedviewer.cgi?page=FunctionalRole&role=Glutaminyl-tRNA%20synthetase%20(EC%206.1.1.18)&subsystem_name=tRNA_aminoacylation,_Glu_and_Gln) |
| Protein Metabolism | Protein biosynthesis | [tRNA aminoacylation, Ser](https://rast.nmpdr.org/seedviewer.cgi?page=Subsystems&subsystem=tRNA_aminoacylation,_Ser&organism=549.408) | [Seryl-tRNA synthetase (EC 6.1.1.11)](https://rast.nmpdr.org/seedviewer.cgi?page=FunctionalRole&role=Seryl-tRNA%20synthetase%20(EC%206.1.1.11)&subsystem_name=tRNA_aminoacylation,_Ser) |
| Protein Metabolism | Protein biosynthesis | [tRNA aminoacylation, Tyr](https://rast.nmpdr.org/seedviewer.cgi?page=Subsystems&subsystem=tRNA_aminoacylation,_Tyr&organism=549.408) | [Tyrosyl-tRNA synthetase (EC 6.1.1.1)](https://rast.nmpdr.org/seedviewer.cgi?page=FunctionalRole&role=Tyrosyl-tRNA%20synthetase%20(EC%206.1.1.1)&subsystem_name=tRNA_aminoacylation,_Tyr) |
| Protein Metabolism | Protein biosynthesis | [tRNA aminoacylation, Leu](https://rast.nmpdr.org/seedviewer.cgi?page=Subsystems&subsystem=tRNA_aminoacylation,_Leu&organism=549.408) | [Leucyl-tRNA synthetase (EC 6.1.1.4)](https://rast.nmpdr.org/seedviewer.cgi?page=FunctionalRole&role=Leucyl-tRNA%20synthetase%20(EC%206.1.1.4)&subsystem_name=tRNA_aminoacylation,_Leu) |
| Protein Metabolism | Protein biosynthesis | [Glycyl-tRNA synthetase](https://rast.nmpdr.org/seedviewer.cgi?page=Subsystems&subsystem=Glycyl-tRNA_synthetase&organism=549.408) | [Glycyl-tRNA synthetase alpha chain (EC 6.1.1.14)](https://rast.nmpdr.org/seedviewer.cgi?page=FunctionalRole&role=Glycyl-tRNA%20synthetase%20alpha%20chain%20(EC%206.1.1.14)&subsystem_name=Glycyl-tRNA_synthetase) |
| Protein Metabolism | Protein biosynthesis | [Glycyl-tRNA synthetase](https://rast.nmpdr.org/seedviewer.cgi?page=Subsystems&subsystem=Glycyl-tRNA_synthetase&organism=549.408) | [Glycyl-tRNA synthetase beta chain (EC 6.1.1.14)](https://rast.nmpdr.org/seedviewer.cgi?page=FunctionalRole&role=Glycyl-tRNA%20synthetase%20beta%20chain%20(EC%206.1.1.14)&subsystem_name=Glycyl-tRNA_synthetase) |
| Protein Metabolism | Protein biosynthesis | [tRNA aminoacylation, Phe](https://rast.nmpdr.org/seedviewer.cgi?page=Subsystems&subsystem=tRNA_aminoacylation,_Phe&organism=549.408) | [Phenylalanyl-tRNA synthetase alpha chain (EC 6.1.1.20)](https://rast.nmpdr.org/seedviewer.cgi?page=FunctionalRole&role=Phenylalanyl-tRNA%20synthetase%20alpha%20chain%20(EC%206.1.1.20)&subsystem_name=tRNA_aminoacylation,_Phe) |
| Protein Metabolism | Protein biosynthesis | [tRNA aminoacylation, Phe](https://rast.nmpdr.org/seedviewer.cgi?page=Subsystems&subsystem=tRNA_aminoacylation,_Phe&organism=549.408) | [Phenylalanyl-tRNA synthetase beta chain (EC 6.1.1.20)](https://rast.nmpdr.org/seedviewer.cgi?page=FunctionalRole&role=Phenylalanyl-tRNA%20synthetase%20beta%20chain%20(EC%206.1.1.20)&subsystem_name=tRNA_aminoacylation,_Phe) |
| Protein Metabolism | Protein processing and modification | [Translation elongation factor P lysylation](https://rast.nmpdr.org/seedviewer.cgi?page=Subsystems&subsystem=Translation_elongation_factor_P_lysylation&organism=549.408) | [Translation elongation factor P Lys34:lysine transferase](https://rast.nmpdr.org/seedviewer.cgi?page=FunctionalRole&role=Translation%20elongation%20factor%20P%20Lys34:lysine%20transferase&subsystem_name=Translation_elongation_factor_P_lysylation) |
| Protein Metabolism | Protein processing and modification | [Translation elongation factor P lysylation](https://rast.nmpdr.org/seedviewer.cgi?page=Subsystems&subsystem=Translation_elongation_factor_P_lysylation&organism=549.408) | [Translation elongation factor P](https://rast.nmpdr.org/seedviewer.cgi?page=FunctionalRole&role=Translation%20elongation%20factor%20P&subsystem_name=Translation_elongation_factor_P_lysylation) |
| Protein Metabolism | Protein processing and modification | [Protein-L-isoaspartate O-methyltransferase](https://rast.nmpdr.org/seedviewer.cgi?page=Subsystems&subsystem=Protein-L-isoaspartate_O-methyltransferase&organism=549.408) | [Protein-L-isoaspartate O-methyltransferase (EC 2.1.1.77)](https://rast.nmpdr.org/seedviewer.cgi?page=FunctionalRole&role=Protein-L-isoaspartate%20O-methyltransferase%20(EC%202.1.1.77)&subsystem_name=Protein-L-isoaspartate_O-methyltransferase) |
| Protein Metabolism | Protein processing and modification | [Lipoprotein Biosynthesis](https://rast.nmpdr.org/seedviewer.cgi?page=Subsystems&subsystem=Lipoprotein_Biosynthesis&organism=549.408) | [Apolipoprotein N-acyltransferase (EC 2.3.1.-)](https://rast.nmpdr.org/seedviewer.cgi?page=FunctionalRole&role=Apolipoprotein%20N-acyltransferase%20(EC%202.3.1.-)&subsystem_name=Lipoprotein_Biosynthesis) |
| Protein Metabolism | Protein processing and modification | [Lipoprotein Biosynthesis](https://rast.nmpdr.org/seedviewer.cgi?page=Subsystems&subsystem=Lipoprotein_Biosynthesis&organism=549.408) | [Lipoprotein signal peptidase (EC 3.4.23.36)](https://rast.nmpdr.org/seedviewer.cgi?page=FunctionalRole&role=Lipoprotein%20signal%20peptidase%20(EC%203.4.23.36)&subsystem_name=Lipoprotein_Biosynthesis) |
| Protein Metabolism | Protein processing and modification | [Lipoprotein Biosynthesis](https://rast.nmpdr.org/seedviewer.cgi?page=Subsystems&subsystem=Lipoprotein_Biosynthesis&organism=549.408) | [Prolipoprotein diacylglyceryl transferase (EC 2.4.99.-)](https://rast.nmpdr.org/seedviewer.cgi?page=FunctionalRole&role=Prolipoprotein%20diacylglyceryl%20transferase%20(EC%202.4.99.-)&subsystem_name=Lipoprotein_Biosynthesis) |
| Protein Metabolism | Protein processing and modification | [Signal peptidase](https://rast.nmpdr.org/seedviewer.cgi?page=Subsystems&subsystem=Signal_peptidase&organism=549.408) | [Signal peptidase I (EC 3.4.21.89)](https://rast.nmpdr.org/seedviewer.cgi?page=FunctionalRole&role=Signal%20peptidase%20I%20(EC%203.4.21.89)&subsystem_name=Signal_peptidase) |
| Protein Metabolism | Protein processing and modification | [Signal peptidase](https://rast.nmpdr.org/seedviewer.cgi?page=Subsystems&subsystem=Signal_peptidase&organism=549.408) | [Lipoprotein signal peptidase (EC 3.4.23.36)](https://rast.nmpdr.org/seedviewer.cgi?page=FunctionalRole&role=Lipoprotein%20signal%20peptidase%20(EC%203.4.23.36)&subsystem_name=Signal_peptidase) |
| Protein Metabolism | Protein processing and modification | [G3E family of P-loop GTPases (metallocenter biosynthesis)](https://rast.nmpdr.org/seedviewer.cgi?page=Subsystems&subsystem=G3E_family_of_P-loop_GTPases_(metallocenter_biosynthesis)&organism=549.408) | [FKBP-type peptidyl-prolyl cis-trans isomerase SlyD (EC 5.2.1.8)](https://rast.nmpdr.org/seedviewer.cgi?page=FunctionalRole&role=FKBP-type%20peptidyl-prolyl%20cis-trans%20isomerase%20SlyD%20(EC%205.2.1.8)&subsystem_name=G3E_family_of_P-loop_GTPases_(metallocenter_biosynthesis)) |
| Protein Metabolism | Protein processing and modification | [G3E family of P-loop GTPases (metallocenter biosynthesis)](https://rast.nmpdr.org/seedviewer.cgi?page=Subsystems&subsystem=G3E_family_of_P-loop_GTPases_(metallocenter_biosynthesis)&organism=549.408) | [Putative metal chaperone, involved in Zn homeostasis, GTPase of COG0523 family](https://rast.nmpdr.org/seedviewer.cgi?page=FunctionalRole&role=Putative%20metal%20chaperone,%20involved%20in%20Zn%20homeostasis,%20GTPase%20of%20COG0523%20family&subsystem_name=G3E_family_of_P-loop_GTPases_(metallocenter_biosynthesis)) |
| Protein Metabolism | Protein processing and modification | [Ribosomal protein S5p acylation](https://rast.nmpdr.org/seedviewer.cgi?page=Subsystems&subsystem=Ribosomal_protein_S5p_acylation&organism=549.408) | [SSU ribosomal protein S5p (S2e)](https://rast.nmpdr.org/seedviewer.cgi?page=FunctionalRole&role=SSU%20ribosomal%20protein%20S5p%20(S2e)&subsystem_name=Ribosomal_protein_S5p_acylation) |
| Protein Metabolism | Protein processing and modification | [Ribosomal protein S5p acylation](https://rast.nmpdr.org/seedviewer.cgi?page=Subsystems&subsystem=Ribosomal_protein_S5p_acylation&organism=549.408) | [Ribosomal-protein-S5p-alanine acetyltransferase](https://rast.nmpdr.org/seedviewer.cgi?page=FunctionalRole&role=Ribosomal-protein-S5p-alanine%20acetyltransferase&subsystem_name=Ribosomal_protein_S5p_acylation) |
| Protein Metabolism | Protein processing and modification | [Protein deglycation](https://rast.nmpdr.org/seedviewer.cgi?page=Subsystems&subsystem=Protein_deglycation&organism=549.408) | [Ribulosamine/erythrulosamine 3-kinase potentially involved in protein deglycation](https://rast.nmpdr.org/seedviewer.cgi?page=FunctionalRole&role=Ribulosamine/erythrulosamine%203-kinase%20potentially%20involved%20in%20protein%20deglycation&subsystem_name=Protein_deglycation) |
| Protein Metabolism | Protein processing and modification | [Protein deglycation](https://rast.nmpdr.org/seedviewer.cgi?page=Subsystems&subsystem=Protein_deglycation&organism=549.408) | [Phytoene synthase (EC 2.5.1.32)](https://rast.nmpdr.org/seedviewer.cgi?page=FunctionalRole&role=Phytoene%20synthase%20(EC%202.5.1.32)&subsystem_name=Protein_deglycation) |
| Protein Metabolism | Protein processing and modification | [Protein deglycation](https://rast.nmpdr.org/seedviewer.cgi?page=Subsystems&subsystem=Protein_deglycation&organism=549.408) | [Phytoene dehydrogenase (EC 1.14.99.-)](https://rast.nmpdr.org/seedviewer.cgi?page=FunctionalRole&role=Phytoene%20dehydrogenase%20(EC%201.14.99.-)&subsystem_name=Protein_deglycation) |
| Protein Metabolism | Protein processing and modification | [Peptide methionine sulfoxide reductase](https://rast.nmpdr.org/seedviewer.cgi?page=Subsystems&subsystem=Peptide_methionine_sulfoxide_reductase&organism=549.408) | [Peptide methionine sulfoxide reductase MsrA (EC 1.8.4.11)](https://rast.nmpdr.org/seedviewer.cgi?page=FunctionalRole&role=Peptide%20methionine%20sulfoxide%20reductase%20MsrA%20(EC%201.8.4.11)&subsystem_name=Peptide_methionine_sulfoxide_reductase) |
| Protein Metabolism | Protein processing and modification | [Peptide methionine sulfoxide reductase](https://rast.nmpdr.org/seedviewer.cgi?page=Subsystems&subsystem=Peptide_methionine_sulfoxide_reductase&organism=549.408) | [Peptide methionine sulfoxide reductase MsrB (EC 1.8.4.12)](https://rast.nmpdr.org/seedviewer.cgi?page=FunctionalRole&role=Peptide%20methionine%20sulfoxide%20reductase%20MsrB%20(EC%201.8.4.12)&subsystem_name=Peptide_methionine_sulfoxide_reductase) |
| Protein Metabolism | Protein degradation | [Putative TldE-TldD proteolytic complex](https://rast.nmpdr.org/seedviewer.cgi?page=Subsystems&subsystem=Putative_TldE-TldD_proteolytic_complex&organism=549.408) | [TldE protein, part of TldE/TldD proteolytic complex](https://rast.nmpdr.org/seedviewer.cgi?page=FunctionalRole&role=TldE%20protein,%20part%20of%20TldE/TldD%20proteolytic%20complex&subsystem_name=Putative_TldE-TldD_proteolytic_complex) |
| Protein Metabolism | Protein degradation | [Putative TldE-TldD proteolytic complex](https://rast.nmpdr.org/seedviewer.cgi?page=Subsystems&subsystem=Putative_TldE-TldD_proteolytic_complex&organism=549.408) | [FIG138315: Putative alpha helix protein](https://rast.nmpdr.org/seedviewer.cgi?page=FunctionalRole&role=FIG138315:%20Putative%20alpha%20helix%20protein&subsystem_name=Putative_TldE-TldD_proteolytic_complex) |
| Protein Metabolism | Protein degradation | [Putative TldE-TldD proteolytic complex](https://rast.nmpdr.org/seedviewer.cgi?page=Subsystems&subsystem=Putative_TldE-TldD_proteolytic_complex&organism=549.408) | [TldD protein, part of TldE/TldD proteolytic complex](https://rast.nmpdr.org/seedviewer.cgi?page=FunctionalRole&role=TldD%20protein,%20part%20of%20TldE/TldD%20proteolytic%20complex&subsystem_name=Putative_TldE-TldD_proteolytic_complex) |
| Protein Metabolism | Protein degradation | [Protein degradation](https://rast.nmpdr.org/seedviewer.cgi?page=Subsystems&subsystem=Protein_degradation&organism=549.408) | [Oligopeptidase A (EC 3.4.24.70)](https://rast.nmpdr.org/seedviewer.cgi?page=FunctionalRole&role=Oligopeptidase%20A%20(EC%203.4.24.70)&subsystem_name=Protein_degradation) |
| Protein Metabolism | Protein degradation | [Protein degradation](https://rast.nmpdr.org/seedviewer.cgi?page=Subsystems&subsystem=Protein_degradation&organism=549.408) | [Leucyl/phenylalanyl-tRNA--protein transferase (EC 2.3.2.6)](https://rast.nmpdr.org/seedviewer.cgi?page=FunctionalRole&role=Leucyl/phenylalanyl-tRNA--protein%20transferase%20(EC%202.3.2.6)&subsystem_name=Protein_degradation) |
| Protein Metabolism | Protein degradation | [Protein degradation](https://rast.nmpdr.org/seedviewer.cgi?page=Subsystems&subsystem=Protein_degradation&organism=549.408) | [Dipeptidyl carboxypeptidase Dcp (EC 3.4.15.5)](https://rast.nmpdr.org/seedviewer.cgi?page=FunctionalRole&role=Dipeptidyl%20carboxypeptidase%20Dcp%20(EC%203.4.15.5)&subsystem_name=Protein_degradation) |
| Protein Metabolism | Protein degradation | [Protein degradation](https://rast.nmpdr.org/seedviewer.cgi?page=Subsystems&subsystem=Protein_degradation&organism=549.408) | [Asp-X dipeptidase](https://rast.nmpdr.org/seedviewer.cgi?page=FunctionalRole&role=Asp-X%20dipeptidase&subsystem_name=Protein_degradation) |
| Protein Metabolism | Protein degradation | [Aminopeptidases (EC 3.4.11.-)](https://rast.nmpdr.org/seedviewer.cgi?page=Subsystems&subsystem=Aminopeptidases_(EC_3.4.11.-)&organism=549.408) | [Peptidase B (EC 3.4.11.23)](https://rast.nmpdr.org/seedviewer.cgi?page=FunctionalRole&role=Peptidase%20B%20(EC%203.4.11.23)&subsystem_name=Aminopeptidases_(EC_3.4.11.-)) |
| Protein Metabolism | Protein degradation | [Aminopeptidases (EC 3.4.11.-)](https://rast.nmpdr.org/seedviewer.cgi?page=Subsystems&subsystem=Aminopeptidases_(EC_3.4.11.-)&organism=549.408) | [Xaa-Pro aminopeptidase (EC 3.4.11.9)](https://rast.nmpdr.org/seedviewer.cgi?page=FunctionalRole&role=Xaa-Pro%20aminopeptidase%20(EC%203.4.11.9)&subsystem_name=Aminopeptidases_(EC_3.4.11.-)) |
| Protein Metabolism | Protein degradation | [Aminopeptidases (EC 3.4.11.-)](https://rast.nmpdr.org/seedviewer.cgi?page=Subsystems&subsystem=Aminopeptidases_(EC_3.4.11.-)&organism=549.408) | [Membrane alanine aminopeptidase N (EC 3.4.11.2)](https://rast.nmpdr.org/seedviewer.cgi?page=FunctionalRole&role=Membrane%20alanine%20aminopeptidase%20N%20(EC%203.4.11.2)&subsystem_name=Aminopeptidases_(EC_3.4.11.-)) |
| Protein Metabolism | Protein degradation | [Aminopeptidases (EC 3.4.11.-)](https://rast.nmpdr.org/seedviewer.cgi?page=Subsystems&subsystem=Aminopeptidases_(EC_3.4.11.-)&organism=549.408) | [Cytosol aminopeptidase PepA (EC 3.4.11.1)](https://rast.nmpdr.org/seedviewer.cgi?page=FunctionalRole&role=Cytosol%20aminopeptidase%20PepA%20(EC%203.4.11.1)&subsystem_name=Aminopeptidases_(EC_3.4.11.-)) |
| Protein Metabolism | Protein degradation | [Metallocarboxypeptidases (EC 3.4.17.-)](https://rast.nmpdr.org/seedviewer.cgi?page=Subsystems&subsystem=Metallocarboxypeptidases_(EC_3.4.17.-)&organism=549.408) | [D-alanyl-D-alanine carboxypeptidase (EC 3.4.16.4)](https://rast.nmpdr.org/seedviewer.cgi?page=FunctionalRole&role=D-alanyl-D-alanine%20carboxypeptidase%20(EC%203.4.16.4)&subsystem_name=Metallocarboxypeptidases_(EC_3.4.17.-)) |
| Protein Metabolism | Protein degradation | [Metallocarboxypeptidases (EC 3.4.17.-)](https://rast.nmpdr.org/seedviewer.cgi?page=Subsystems&subsystem=Metallocarboxypeptidases_(EC_3.4.17.-)&organism=549.408) | [Muramoyltetrapeptide carboxypeptidase (EC 3.4.17.13)](https://rast.nmpdr.org/seedviewer.cgi?page=FunctionalRole&role=Muramoyltetrapeptide%20carboxypeptidase%20(EC%203.4.17.13)&subsystem_name=Metallocarboxypeptidases_(EC_3.4.17.-)) |
| Protein Metabolism | Protein degradation | [Metallocarboxypeptidases (EC 3.4.17.-)](https://rast.nmpdr.org/seedviewer.cgi?page=Subsystems&subsystem=Metallocarboxypeptidases_(EC_3.4.17.-)&organism=549.408) | [Thermostable carboxypeptidase 1 (EC 3.4.17.19)](https://rast.nmpdr.org/seedviewer.cgi?page=FunctionalRole&role=Thermostable%20carboxypeptidase%201%20(EC%203.4.17.19)&subsystem_name=Metallocarboxypeptidases_(EC_3.4.17.-)) |
| Protein Metabolism | Protein degradation | [Dipeptidases (EC 3.4.13.-)](https://rast.nmpdr.org/seedviewer.cgi?page=Subsystems&subsystem=Dipeptidases_(EC_3.4.13.-)&organism=549.408) | [Xaa-Pro dipeptidase PepQ (EC 3.4.13.9)](https://rast.nmpdr.org/seedviewer.cgi?page=FunctionalRole&role=Xaa-Pro%20dipeptidase%20PepQ%20(EC%203.4.13.9)&subsystem_name=Dipeptidases_(EC_3.4.13.-)) |
| Protein Metabolism | Protein degradation | [Dipeptidases (EC 3.4.13.-)](https://rast.nmpdr.org/seedviewer.cgi?page=Subsystems&subsystem=Dipeptidases_(EC_3.4.13.-)&organism=549.408) | [Aminoacyl-histidine dipeptidase (Peptidase D) (EC 3.4.13.3)](https://rast.nmpdr.org/seedviewer.cgi?page=FunctionalRole&role=Aminoacyl-histidine%20dipeptidase%20(Peptidase%20D)%20(EC%203.4.13.3)&subsystem_name=Dipeptidases_(EC_3.4.13.-)) |
| Protein Metabolism | Protein degradation | [Dipeptidases (EC 3.4.13.-)](https://rast.nmpdr.org/seedviewer.cgi?page=Subsystems&subsystem=Dipeptidases_(EC_3.4.13.-)&organism=549.408) | [Alpha-aspartyl dipeptidase Peptidase E (EC 3.4.13.21)](https://rast.nmpdr.org/seedviewer.cgi?page=FunctionalRole&role=Alpha-aspartyl%20dipeptidase%20Peptidase%20E%20(EC%203.4.13.21)&subsystem_name=Dipeptidases_(EC_3.4.13.-)) |
| Protein Metabolism | Protein degradation | [Proteasome bacterial](https://rast.nmpdr.org/seedviewer.cgi?page=Subsystems&subsystem=Proteasome_bacterial&organism=549.408) | [ATP-dependent Clp protease ATP-binding subunit ClpX](https://rast.nmpdr.org/seedviewer.cgi?page=FunctionalRole&role=ATP-dependent%20Clp%20protease%20ATP-binding%20subunit%20ClpX&subsystem_name=Proteasome_bacterial) |
| Protein Metabolism | Protein degradation | [Proteasome bacterial](https://rast.nmpdr.org/seedviewer.cgi?page=Subsystems&subsystem=Proteasome_bacterial&organism=549.408) | [ATP-dependent protease La (EC 3.4.21.53) Type II](https://rast.nmpdr.org/seedviewer.cgi?page=FunctionalRole&role=ATP-dependent%20protease%20La%20(EC%203.4.21.53)%20Type%20II&subsystem_name=Proteasome_bacterial) |
| Protein Metabolism | Protein degradation | [Proteasome bacterial](https://rast.nmpdr.org/seedviewer.cgi?page=Subsystems&subsystem=Proteasome_bacterial&organism=549.408) | [ATP-dependent hsl protease ATP-binding subunit HslU](https://rast.nmpdr.org/seedviewer.cgi?page=FunctionalRole&role=ATP-dependent%20hsl%20protease%20ATP-binding%20subunit%20HslU&subsystem_name=Proteasome_bacterial) |
| Protein Metabolism | Protein degradation | [Proteasome bacterial](https://rast.nmpdr.org/seedviewer.cgi?page=Subsystems&subsystem=Proteasome_bacterial&organism=549.408) | [ATP-dependent Clp protease proteolytic subunit (EC 3.4.21.92)](https://rast.nmpdr.org/seedviewer.cgi?page=FunctionalRole&role=ATP-dependent%20Clp%20protease%20proteolytic%20subunit%20(EC%203.4.21.92)&subsystem_name=Proteasome_bacterial) |
| Protein Metabolism | Protein degradation | [Proteasome bacterial](https://rast.nmpdr.org/seedviewer.cgi?page=Subsystems&subsystem=Proteasome_bacterial&organism=549.408) | [ATP-dependent protease HslV (EC 3.4.25.-)](https://rast.nmpdr.org/seedviewer.cgi?page=FunctionalRole&role=ATP-dependent%20protease%20HslV%20(EC%203.4.25.-)&subsystem_name=Proteasome_bacterial) |
| Protein Metabolism | Protein degradation | [Proteasome bacterial](https://rast.nmpdr.org/seedviewer.cgi?page=Subsystems&subsystem=Proteasome_bacterial&organism=549.408) | [ATP-dependent protease La (EC 3.4.21.53) Type I](https://rast.nmpdr.org/seedviewer.cgi?page=FunctionalRole&role=ATP-dependent%20protease%20La%20(EC%203.4.21.53)%20Type%20I&subsystem_name=Proteasome_bacterial) |
| Protein Metabolism | Protein degradation | [Omega peptidases (EC 3.4.19.-)](https://rast.nmpdr.org/seedviewer.cgi?page=Subsystems&subsystem=Omega_peptidases_(EC_3.4.19.-)&organism=549.408) | [Isoaspartyl aminopeptidase (EC 3.4.19.5)](https://rast.nmpdr.org/seedviewer.cgi?page=FunctionalRole&role=Isoaspartyl%20aminopeptidase%20(EC%203.4.19.5)&subsystem_name=Omega_peptidases_(EC_3.4.19.-)) |
| Protein Metabolism | Protein degradation | [Omega peptidases (EC 3.4.19.-)](https://rast.nmpdr.org/seedviewer.cgi?page=Subsystems&subsystem=Omega_peptidases_(EC_3.4.19.-)&organism=549.408) | [Pyrrolidone-carboxylate peptidase (EC 3.4.19.3)](https://rast.nmpdr.org/seedviewer.cgi?page=FunctionalRole&role=Pyrrolidone-carboxylate%20peptidase%20(EC%203.4.19.3)&subsystem_name=Omega_peptidases_(EC_3.4.19.-)) |
| Protein Metabolism | Protein degradation | [Proteolysis in bacteria, ATP-dependent](https://rast.nmpdr.org/seedviewer.cgi?page=Subsystems&subsystem=Proteolysis_in_bacteria,_ATP-dependent&organism=549.408) | [DNA repair protein RadA](https://rast.nmpdr.org/seedviewer.cgi?page=FunctionalRole&role=DNA%20repair%20protein%20RadA&subsystem_name=Proteolysis_in_bacteria,_ATP-dependent) |
| Protein Metabolism | Protein degradation | [Proteolysis in bacteria, ATP-dependent](https://rast.nmpdr.org/seedviewer.cgi?page=Subsystems&subsystem=Proteolysis_in_bacteria,_ATP-dependent&organism=549.408) | [ATP-dependent Clp protease ATP-binding subunit ClpX](https://rast.nmpdr.org/seedviewer.cgi?page=FunctionalRole&role=ATP-dependent%20Clp%20protease%20ATP-binding%20subunit%20ClpX&subsystem_name=Proteolysis_in_bacteria,_ATP-dependent) |
| Protein Metabolism | Protein degradation | [Proteolysis in bacteria, ATP-dependent](https://rast.nmpdr.org/seedviewer.cgi?page=Subsystems&subsystem=Proteolysis_in_bacteria,_ATP-dependent&organism=549.408) | [ClpXP protease specificity-enhancing factor](https://rast.nmpdr.org/seedviewer.cgi?page=FunctionalRole&role=ClpXP%20protease%20specificity-enhancing%20factor&subsystem_name=Proteolysis_in_bacteria,_ATP-dependent) |
| Protein Metabolism | Protein degradation | [Proteolysis in bacteria, ATP-dependent](https://rast.nmpdr.org/seedviewer.cgi?page=Subsystems&subsystem=Proteolysis_in_bacteria,_ATP-dependent&organism=549.408) | [ATP-dependent Clp protease ATP-binding subunit ClpA](https://rast.nmpdr.org/seedviewer.cgi?page=FunctionalRole&role=ATP-dependent%20Clp%20protease%20ATP-binding%20subunit%20ClpA&subsystem_name=Proteolysis_in_bacteria,_ATP-dependent) |
| Protein Metabolism | Protein degradation | [Proteolysis in bacteria, ATP-dependent](https://rast.nmpdr.org/seedviewer.cgi?page=Subsystems&subsystem=Proteolysis_in_bacteria,_ATP-dependent&organism=549.408) | [ATP-dependent protease La (EC 3.4.21.53) Type II](https://rast.nmpdr.org/seedviewer.cgi?page=FunctionalRole&role=ATP-dependent%20protease%20La%20(EC%203.4.21.53)%20Type%20II&subsystem_name=Proteolysis_in_bacteria,_ATP-dependent) |
| Protein Metabolism | Protein degradation | [Proteolysis in bacteria, ATP-dependent](https://rast.nmpdr.org/seedviewer.cgi?page=Subsystems&subsystem=Proteolysis_in_bacteria,_ATP-dependent&organism=549.408) | [Outer membrane stress sensor protease DegS](https://rast.nmpdr.org/seedviewer.cgi?page=FunctionalRole&role=Outer%20membrane%20stress%20sensor%20protease%20DegS&subsystem_name=Proteolysis_in_bacteria,_ATP-dependent) |
| Protein Metabolism | Protein degradation | [Proteolysis in bacteria, ATP-dependent](https://rast.nmpdr.org/seedviewer.cgi?page=Subsystems&subsystem=Proteolysis_in_bacteria,_ATP-dependent&organism=549.408) | [Outer membrane stress sensor protease DegQ, serine protease](https://rast.nmpdr.org/seedviewer.cgi?page=FunctionalRole&role=Outer%20membrane%20stress%20sensor%20protease%20DegQ,%20serine%20protease&subsystem_name=Proteolysis_in_bacteria,_ATP-dependent) |
| Protein Metabolism | Protein degradation | [Proteolysis in bacteria, ATP-dependent](https://rast.nmpdr.org/seedviewer.cgi?page=Subsystems&subsystem=Proteolysis_in_bacteria,_ATP-dependent&organism=549.408) | [ClpB protein](https://rast.nmpdr.org/seedviewer.cgi?page=FunctionalRole&role=ClpB%20protein&subsystem_name=Proteolysis_in_bacteria,_ATP-dependent) |
| Protein Metabolism | Protein degradation | [Proteolysis in bacteria, ATP-dependent](https://rast.nmpdr.org/seedviewer.cgi?page=Subsystems&subsystem=Proteolysis_in_bacteria,_ATP-dependent&organism=549.408) | [ATP-dependent protease HslV (EC 3.4.25.-)](https://rast.nmpdr.org/seedviewer.cgi?page=FunctionalRole&role=ATP-dependent%20protease%20HslV%20(EC%203.4.25.-)&subsystem_name=Proteolysis_in_bacteria,_ATP-dependent) |
| Protein Metabolism | Protein degradation | [Proteolysis in bacteria, ATP-dependent](https://rast.nmpdr.org/seedviewer.cgi?page=Subsystems&subsystem=Proteolysis_in_bacteria,_ATP-dependent&organism=549.408) | [ATP-dependent protease La (EC 3.4.21.53) Type I](https://rast.nmpdr.org/seedviewer.cgi?page=FunctionalRole&role=ATP-dependent%20protease%20La%20(EC%203.4.21.53)%20Type%20I&subsystem_name=Proteolysis_in_bacteria,_ATP-dependent) |
| Protein Metabolism | Protein degradation | [Proteolysis in bacteria, ATP-dependent](https://rast.nmpdr.org/seedviewer.cgi?page=Subsystems&subsystem=Proteolysis_in_bacteria,_ATP-dependent&organism=549.408) | [ATP-dependent Clp protease adaptor protein ClpS](https://rast.nmpdr.org/seedviewer.cgi?page=FunctionalRole&role=ATP-dependent%20Clp%20protease%20adaptor%20protein%20ClpS&subsystem_name=Proteolysis_in_bacteria,_ATP-dependent) |
| Protein Metabolism | Protein degradation | [Proteolysis in bacteria, ATP-dependent](https://rast.nmpdr.org/seedviewer.cgi?page=Subsystems&subsystem=Proteolysis_in_bacteria,_ATP-dependent&organism=549.408) | [ATPase, AFG1 family](https://rast.nmpdr.org/seedviewer.cgi?page=FunctionalRole&role=ATPase,%20AFG1%20family&subsystem_name=Proteolysis_in_bacteria,_ATP-dependent) |
| Protein Metabolism | Protein degradation | [Proteolysis in bacteria, ATP-dependent](https://rast.nmpdr.org/seedviewer.cgi?page=Subsystems&subsystem=Proteolysis_in_bacteria,_ATP-dependent&organism=549.408) | [ATP-dependent Clp protease proteolytic subunit (EC 3.4.21.92)](https://rast.nmpdr.org/seedviewer.cgi?page=FunctionalRole&role=ATP-dependent%20Clp%20protease%20proteolytic%20subunit%20(EC%203.4.21.92)&subsystem_name=Proteolysis_in_bacteria,_ATP-dependent) |
| Protein Metabolism | Protein degradation | [Proteolysis in bacteria, ATP-dependent](https://rast.nmpdr.org/seedviewer.cgi?page=Subsystems&subsystem=Proteolysis_in_bacteria,_ATP-dependent&organism=549.408) | [ATP-dependent hsl protease ATP-binding subunit HslU](https://rast.nmpdr.org/seedviewer.cgi?page=FunctionalRole&role=ATP-dependent%20hsl%20protease%20ATP-binding%20subunit%20HslU&subsystem_name=Proteolysis_in_bacteria,_ATP-dependent) |
| Cell Division and Cell Cycle | Cell Division and Cell Cycle - no subcategory | [MukBEF Chromosome Condensation](https://rast.nmpdr.org/seedviewer.cgi?page=Subsystems&subsystem=MukBEF_Chromosome_Condensation&organism=549.408) | [Chromosome partition protein MukF](https://rast.nmpdr.org/seedviewer.cgi?page=FunctionalRole&role=Chromosome%20partition%20protein%20MukF&subsystem_name=MukBEF_Chromosome_Condensation) |
| Cell Division and Cell Cycle | Cell Division and Cell Cycle - no subcategory | [MukBEF Chromosome Condensation](https://rast.nmpdr.org/seedviewer.cgi?page=Subsystems&subsystem=MukBEF_Chromosome_Condensation&organism=549.408) | [S-adenosylmethionine-dependent methyltransferase Functionally Coupled to the MukBEF Chromosome Partitioning Mechanism](https://rast.nmpdr.org/seedviewer.cgi?page=FunctionalRole&role=S-adenosylmethionine-dependent%20methyltransferase%20Functionally%20Coupled%20to%20the%20MukBEF%20Chromosome%20Partitioning%20Mechanism&subsystem_name=MukBEF_Chromosome_Condensation) |
| Cell Division and Cell Cycle | Cell Division and Cell Cycle - no subcategory | [MukBEF Chromosome Condensation](https://rast.nmpdr.org/seedviewer.cgi?page=Subsystems&subsystem=MukBEF_Chromosome_Condensation&organism=549.408) | [Chromosome partition protein MukB](https://rast.nmpdr.org/seedviewer.cgi?page=FunctionalRole&role=Chromosome%20partition%20protein%20MukB&subsystem_name=MukBEF_Chromosome_Condensation) |
| Cell Division and Cell Cycle | Cell Division and Cell Cycle - no subcategory | [MukBEF Chromosome Condensation](https://rast.nmpdr.org/seedviewer.cgi?page=Subsystems&subsystem=MukBEF_Chromosome_Condensation&organism=549.408) | [Chromosome partition protein MukE](https://rast.nmpdr.org/seedviewer.cgi?page=FunctionalRole&role=Chromosome%20partition%20protein%20MukE&subsystem_name=MukBEF_Chromosome_Condensation) |
| Cell Division and Cell Cycle | Cell Division and Cell Cycle - no subcategory | [MukBEF Chromosome Condensation](https://rast.nmpdr.org/seedviewer.cgi?page=Subsystems&subsystem=MukBEF_Chromosome_Condensation&organism=549.408) | [Membrane Protein Functionally coupled to the MukBEF Chromosome Partitioning Mechanism](https://rast.nmpdr.org/seedviewer.cgi?page=FunctionalRole&role=Membrane%20Protein%20Functionally%20coupled%20to%20the%20MukBEF%20Chromosome%20Partitioning%20Mechanism&subsystem_name=MukBEF_Chromosome_Condensation) |
| Cell Division and Cell Cycle | Cell Division and Cell Cycle - no subcategory | [YgjD and YeaZ](https://rast.nmpdr.org/seedviewer.cgi?page=Subsystems&subsystem=YgjD_and_YeaZ&organism=549.408) | [TsaD/Kae1/Qri7 protein, required for threonylcarbamoyladenosine t(6)A37 formation in tRNA](https://rast.nmpdr.org/seedviewer.cgi?page=FunctionalRole&role=TsaD/Kae1/Qri7%20protein,%20required%20for%20threonylcarbamoyladenosine%20t(6)A37%20formation%20in%20tRNA&subsystem_name=YgjD_and_YeaZ) |
| Cell Division and Cell Cycle | Cell Division and Cell Cycle - no subcategory | [YgjD and YeaZ](https://rast.nmpdr.org/seedviewer.cgi?page=Subsystems&subsystem=YgjD_and_YeaZ&organism=549.408) | [TsaB protein, required for threonylcarbamoyladenosine (t(6)A) formation in tRNA](https://rast.nmpdr.org/seedviewer.cgi?page=FunctionalRole&role=TsaB%20protein,%20required%20for%20threonylcarbamoyladenosine%20(t(6)A)%20formation%20in%20tRNA&subsystem_name=YgjD_and_YeaZ) |
| Cell Division and Cell Cycle | Cell Division and Cell Cycle - no subcategory | [Macromolecular synthesis operon](https://rast.nmpdr.org/seedviewer.cgi?page=Subsystems&subsystem=Macromolecular_synthesis_operon&organism=549.408) | [TsaD/Kae1/Qri7 protein, required for threonylcarbamoyladenosine t(6)A37 formation in tRNA](https://rast.nmpdr.org/seedviewer.cgi?page=FunctionalRole&role=TsaD/Kae1/Qri7%20protein,%20required%20for%20threonylcarbamoyladenosine%20t(6)A37%20formation%20in%20tRNA&subsystem_name=Macromolecular_synthesis_operon) |
| Cell Division and Cell Cycle | Cell Division and Cell Cycle - no subcategory | [Macromolecular synthesis operon](https://rast.nmpdr.org/seedviewer.cgi?page=Subsystems&subsystem=Macromolecular_synthesis_operon&organism=549.408) | [RNA polymerase sigma factor RpoD](https://rast.nmpdr.org/seedviewer.cgi?page=FunctionalRole&role=RNA%20polymerase%20sigma%20factor%20RpoD&subsystem_name=Macromolecular_synthesis_operon) |
| Cell Division and Cell Cycle | Cell Division and Cell Cycle - no subcategory | [Macromolecular synthesis operon](https://rast.nmpdr.org/seedviewer.cgi?page=Subsystems&subsystem=Macromolecular_synthesis_operon&organism=549.408) | [DNA primase (EC 2.7.7.-)](https://rast.nmpdr.org/seedviewer.cgi?page=FunctionalRole&role=DNA%20primase%20(EC%202.7.7.-)&subsystem_name=Macromolecular_synthesis_operon) |
| Cell Division and Cell Cycle | Cell Division and Cell Cycle - no subcategory | [Macromolecular synthesis operon](https://rast.nmpdr.org/seedviewer.cgi?page=Subsystems&subsystem=Macromolecular_synthesis_operon&organism=549.408) | [SSU ribosomal protein S21p](https://rast.nmpdr.org/seedviewer.cgi?page=FunctionalRole&role=SSU%20ribosomal%20protein%20S21p&subsystem_name=Macromolecular_synthesis_operon) |
| Cell Division and Cell Cycle | Cell Division and Cell Cycle - no subcategory | [Macromolecular synthesis operon](https://rast.nmpdr.org/seedviewer.cgi?page=Subsystems&subsystem=Macromolecular_synthesis_operon&organism=549.408) | [Carbamoyl-phosphate synthase small chain (EC 6.3.5.5)](https://rast.nmpdr.org/seedviewer.cgi?page=FunctionalRole&role=Carbamoyl-phosphate%20synthase%20small%20chain%20(EC%206.3.5.5)&subsystem_name=Macromolecular_synthesis_operon) |
| Cell Division and Cell Cycle | Cell Division and Cell Cycle - no subcategory | [Macromolecular synthesis operon](https://rast.nmpdr.org/seedviewer.cgi?page=Subsystems&subsystem=Macromolecular_synthesis_operon&organism=549.408) | [Carbamoyl-phosphate synthase large chain (EC 6.3.5.5)](https://rast.nmpdr.org/seedviewer.cgi?page=FunctionalRole&role=Carbamoyl-phosphate%20synthase%20large%20chain%20(EC%206.3.5.5)&subsystem_name=Macromolecular_synthesis_operon) |
| Cell Division and Cell Cycle | Cell Division and Cell Cycle - no subcategory | [Bacterial Cytoskeleton](https://rast.nmpdr.org/seedviewer.cgi?page=Subsystems&subsystem=Bacterial_Cytoskeleton&organism=549.408) | [Septum site-determining protein MinC](https://rast.nmpdr.org/seedviewer.cgi?page=FunctionalRole&role=Septum%20site-determining%20protein%20MinC&subsystem_name=Bacterial_Cytoskeleton) |
| Cell Division and Cell Cycle | Cell Division and Cell Cycle - no subcategory | [Bacterial Cytoskeleton](https://rast.nmpdr.org/seedviewer.cgi?page=Subsystems&subsystem=Bacterial_Cytoskeleton&organism=549.408) | [Cell division protein FtsI [Peptidoglycan synthetase] (EC 2.4.1.129)](https://rast.nmpdr.org/seedviewer.cgi?page=FunctionalRole&role=Cell%20division%20protein%20FtsI%20%5bPeptidoglycan%20synthetase%5d%20(EC%202.4.1.129)&subsystem_name=Bacterial_Cytoskeleton) |
| Cell Division and Cell Cycle | Cell Division and Cell Cycle - no subcategory | [Bacterial Cytoskeleton](https://rast.nmpdr.org/seedviewer.cgi?page=Subsystems&subsystem=Bacterial_Cytoskeleton&organism=549.408) | [Cell division protein FtsW](https://rast.nmpdr.org/seedviewer.cgi?page=FunctionalRole&role=Cell%20division%20protein%20FtsW&subsystem_name=Bacterial_Cytoskeleton) |
| Cell Division and Cell Cycle | Cell Division and Cell Cycle - no subcategory | [Bacterial Cytoskeleton](https://rast.nmpdr.org/seedviewer.cgi?page=Subsystems&subsystem=Bacterial_Cytoskeleton&organism=549.408) | [Chromosome (plasmid) partitioning protein ParB](https://rast.nmpdr.org/seedviewer.cgi?page=FunctionalRole&role=Chromosome%20(plasmid)%20partitioning%20protein%20ParB&subsystem_name=Bacterial_Cytoskeleton) |
| Cell Division and Cell Cycle | Cell Division and Cell Cycle - no subcategory | [Bacterial Cytoskeleton](https://rast.nmpdr.org/seedviewer.cgi?page=Subsystems&subsystem=Bacterial_Cytoskeleton&organism=549.408) | [Rod shape-determining protein MreD](https://rast.nmpdr.org/seedviewer.cgi?page=FunctionalRole&role=Rod%20shape-determining%20protein%20MreD&subsystem_name=Bacterial_Cytoskeleton) |
| Cell Division and Cell Cycle | Cell Division and Cell Cycle - no subcategory | [Bacterial Cytoskeleton](https://rast.nmpdr.org/seedviewer.cgi?page=Subsystems&subsystem=Bacterial_Cytoskeleton&organism=549.408) | [Cell division protein FtsL](https://rast.nmpdr.org/seedviewer.cgi?page=FunctionalRole&role=Cell%20division%20protein%20FtsL&subsystem_name=Bacterial_Cytoskeleton) |
| Cell Division and Cell Cycle | Cell Division and Cell Cycle - no subcategory | [Bacterial Cytoskeleton](https://rast.nmpdr.org/seedviewer.cgi?page=Subsystems&subsystem=Bacterial_Cytoskeleton&organism=549.408) | [Cell division protein FtsK](https://rast.nmpdr.org/seedviewer.cgi?page=FunctionalRole&role=Cell%20division%20protein%20FtsK&subsystem_name=Bacterial_Cytoskeleton) |
| Cell Division and Cell Cycle | Cell Division and Cell Cycle - no subcategory | [Bacterial Cytoskeleton](https://rast.nmpdr.org/seedviewer.cgi?page=Subsystems&subsystem=Bacterial_Cytoskeleton&organism=549.408) | [Septum formation protein Maf](https://rast.nmpdr.org/seedviewer.cgi?page=FunctionalRole&role=Septum%20formation%20protein%20Maf&subsystem_name=Bacterial_Cytoskeleton) |
| Cell Division and Cell Cycle | Cell Division and Cell Cycle - no subcategory | [Bacterial Cytoskeleton](https://rast.nmpdr.org/seedviewer.cgi?page=Subsystems&subsystem=Bacterial_Cytoskeleton&organism=549.408) | [Septum site-determining protein MinD](https://rast.nmpdr.org/seedviewer.cgi?page=FunctionalRole&role=Septum%20site-determining%20protein%20MinD&subsystem_name=Bacterial_Cytoskeleton) |
| Cell Division and Cell Cycle | Cell Division and Cell Cycle - no subcategory | [Bacterial Cytoskeleton](https://rast.nmpdr.org/seedviewer.cgi?page=Subsystems&subsystem=Bacterial_Cytoskeleton&organism=549.408) | [Chromosome (plasmid) partitioning protein ParA](https://rast.nmpdr.org/seedviewer.cgi?page=FunctionalRole&role=Chromosome%20(plasmid)%20partitioning%20protein%20ParA&subsystem_name=Bacterial_Cytoskeleton) |
| Cell Division and Cell Cycle | Cell Division and Cell Cycle - no subcategory | [Bacterial Cytoskeleton](https://rast.nmpdr.org/seedviewer.cgi?page=Subsystems&subsystem=Bacterial_Cytoskeleton&organism=549.408) | [Rod shape-determining protein RodA](https://rast.nmpdr.org/seedviewer.cgi?page=FunctionalRole&role=Rod%20shape-determining%20protein%20RodA&subsystem_name=Bacterial_Cytoskeleton) |
| Cell Division and Cell Cycle | Cell Division and Cell Cycle - no subcategory | [Bacterial Cytoskeleton](https://rast.nmpdr.org/seedviewer.cgi?page=Subsystems&subsystem=Bacterial_Cytoskeleton&organism=549.408) | [Cell division protein DivIC (FtsB), stabilizes FtsL against RasP cleavage](https://rast.nmpdr.org/seedviewer.cgi?page=FunctionalRole&role=Cell%20division%20protein%20DivIC%20(FtsB),%20stabilizes%20FtsL%20against%20RasP%20cleavage&subsystem_name=Bacterial_Cytoskeleton) |
| Cell Division and Cell Cycle | Cell Division and Cell Cycle - no subcategory | [Bacterial Cytoskeleton](https://rast.nmpdr.org/seedviewer.cgi?page=Subsystems&subsystem=Bacterial_Cytoskeleton&organism=549.408) | [Cell division protein FtsZ (EC 3.4.24.-)](https://rast.nmpdr.org/seedviewer.cgi?page=FunctionalRole&role=Cell%20division%20protein%20FtsZ%20(EC%203.4.24.-)&subsystem_name=Bacterial_Cytoskeleton) |
| Cell Division and Cell Cycle | Cell Division and Cell Cycle - no subcategory | [Bacterial Cytoskeleton](https://rast.nmpdr.org/seedviewer.cgi?page=Subsystems&subsystem=Bacterial_Cytoskeleton&organism=549.408) | [Cell division protein FtsN](https://rast.nmpdr.org/seedviewer.cgi?page=FunctionalRole&role=Cell%20division%20protein%20FtsN&subsystem_name=Bacterial_Cytoskeleton) |
| Cell Division and Cell Cycle | Cell Division and Cell Cycle - no subcategory | [Bacterial Cytoskeleton](https://rast.nmpdr.org/seedviewer.cgi?page=Subsystems&subsystem=Bacterial_Cytoskeleton&organism=549.408) | [Cell division protein FtsA](https://rast.nmpdr.org/seedviewer.cgi?page=FunctionalRole&role=Cell%20division%20protein%20FtsA&subsystem_name=Bacterial_Cytoskeleton) |
| Cell Division and Cell Cycle | Cell Division and Cell Cycle - no subcategory | [Bacterial Cytoskeleton](https://rast.nmpdr.org/seedviewer.cgi?page=Subsystems&subsystem=Bacterial_Cytoskeleton&organism=549.408) | [Rod shape-determining protein MreB](https://rast.nmpdr.org/seedviewer.cgi?page=FunctionalRole&role=Rod%20shape-determining%20protein%20MreB&subsystem_name=Bacterial_Cytoskeleton) |
| Cell Division and Cell Cycle | Cell Division and Cell Cycle - no subcategory | [Bacterial Cytoskeleton](https://rast.nmpdr.org/seedviewer.cgi?page=Subsystems&subsystem=Bacterial_Cytoskeleton&organism=549.408) | [Rod shape-determining protein MreC](https://rast.nmpdr.org/seedviewer.cgi?page=FunctionalRole&role=Rod%20shape-determining%20protein%20MreC&subsystem_name=Bacterial_Cytoskeleton) |
| Cell Division and Cell Cycle | Cell Division and Cell Cycle - no subcategory | [Bacterial Cytoskeleton](https://rast.nmpdr.org/seedviewer.cgi?page=Subsystems&subsystem=Bacterial_Cytoskeleton&organism=549.408) | [Cell division protein FtsQ](https://rast.nmpdr.org/seedviewer.cgi?page=FunctionalRole&role=Cell%20division%20protein%20FtsQ&subsystem_name=Bacterial_Cytoskeleton) |
| Cell Division and Cell Cycle | Cell Division and Cell Cycle - no subcategory | [Bacterial Cytoskeleton](https://rast.nmpdr.org/seedviewer.cgi?page=Subsystems&subsystem=Bacterial_Cytoskeleton&organism=549.408) | [Cell division protein MraZ](https://rast.nmpdr.org/seedviewer.cgi?page=FunctionalRole&role=Cell%20division%20protein%20MraZ&subsystem_name=Bacterial_Cytoskeleton) |
| Cell Division and Cell Cycle | Cell Division and Cell Cycle - no subcategory | [Bacterial Cytoskeleton](https://rast.nmpdr.org/seedviewer.cgi?page=Subsystems&subsystem=Bacterial_Cytoskeleton&organism=549.408) | [Cell division topological specificity factor MinE](https://rast.nmpdr.org/seedviewer.cgi?page=FunctionalRole&role=Cell%20division%20topological%20specificity%20factor%20MinE&subsystem_name=Bacterial_Cytoskeleton) |
| Cell Division and Cell Cycle | Cell Division and Cell Cycle - no subcategory | [Bacterial Cytoskeleton](https://rast.nmpdr.org/seedviewer.cgi?page=Subsystems&subsystem=Bacterial_Cytoskeleton&organism=549.408) | [Z-ring-associated protein ZapA](https://rast.nmpdr.org/seedviewer.cgi?page=FunctionalRole&role=Z-ring-associated%20protein%20ZapA&subsystem_name=Bacterial_Cytoskeleton) |
| Cell Division and Cell Cycle | Cell Division and Cell Cycle - no subcategory | [Bacterial Cytoskeleton](https://rast.nmpdr.org/seedviewer.cgi?page=Subsystems&subsystem=Bacterial_Cytoskeleton&organism=549.408) | [Cell division protein ZipA](https://rast.nmpdr.org/seedviewer.cgi?page=FunctionalRole&role=Cell%20division%20protein%20ZipA&subsystem_name=Bacterial_Cytoskeleton) |
| Cell Division and Cell Cycle | Cell Division and Cell Cycle - no subcategory | [Intracellular septation in Enterobacteria](https://rast.nmpdr.org/seedviewer.cgi?page=Subsystems&subsystem=Intracellular_septation_in_Enterobacteria&organism=549.408) | [Intracellular septation protein IspA](https://rast.nmpdr.org/seedviewer.cgi?page=FunctionalRole&role=Intracellular%20septation%20protein%20IspA&subsystem_name=Intracellular_septation_in_Enterobacteria) |
| Cell Division and Cell Cycle | Cell Division and Cell Cycle - no subcategory | [Intracellular septation in Enterobacteria](https://rast.nmpdr.org/seedviewer.cgi?page=Subsystems&subsystem=Intracellular_septation_in_Enterobacteria&organism=549.408) | [Acyl-CoA thioesterase YciA, involved in membrane biogenesis](https://rast.nmpdr.org/seedviewer.cgi?page=FunctionalRole&role=Acyl-CoA%20thioesterase%20YciA,%20involved%20in%20membrane%20biogenesis&subsystem_name=Intracellular_septation_in_Enterobacteria) |
| Cell Division and Cell Cycle | Cell Division and Cell Cycle - no subcategory | [Intracellular septation in Enterobacteria](https://rast.nmpdr.org/seedviewer.cgi?page=Subsystems&subsystem=Intracellular_septation_in_Enterobacteria&organism=549.408) | [Membrane protein YciC, linked to IspA](https://rast.nmpdr.org/seedviewer.cgi?page=FunctionalRole&role=Membrane%20protein%20YciC,%20linked%20to%20IspA&subsystem_name=Intracellular_septation_in_Enterobacteria) |
| Motility and Chemotaxis | Motility and Chemotaxis - no subcategory | [Bacterial Chemotaxis](https://rast.nmpdr.org/seedviewer.cgi?page=Subsystems&subsystem=Bacterial_Chemotaxis&organism=549.408) | [Chemotaxis protein CheV (EC 2.7.3.-)](https://rast.nmpdr.org/seedviewer.cgi?page=FunctionalRole&role=Chemotaxis%20protein%20CheV%20(EC%202.7.3.-)&subsystem_name=Bacterial_Chemotaxis) |
| Motility and Chemotaxis | Motility and Chemotaxis - no subcategory | [Bacterial Chemotaxis](https://rast.nmpdr.org/seedviewer.cgi?page=Subsystems&subsystem=Bacterial_Chemotaxis&organism=549.408) | [Positive regulator of CheA protein activity (CheW)](https://rast.nmpdr.org/seedviewer.cgi?page=FunctionalRole&role=Positive%20regulator%20of%20CheA%20protein%20activity%20(CheW)&subsystem_name=Bacterial_Chemotaxis) |
| Motility and Chemotaxis | Motility and Chemotaxis - no subcategory | [Bacterial Chemotaxis](https://rast.nmpdr.org/seedviewer.cgi?page=Subsystems&subsystem=Bacterial_Chemotaxis&organism=549.408) | [Flagellar motor switch protein FliN](https://rast.nmpdr.org/seedviewer.cgi?page=FunctionalRole&role=Flagellar%20motor%20switch%20protein%20FliN&subsystem_name=Bacterial_Chemotaxis) |
| Motility and Chemotaxis | Motility and Chemotaxis - no subcategory | [Bacterial Chemotaxis](https://rast.nmpdr.org/seedviewer.cgi?page=Subsystems&subsystem=Bacterial_Chemotaxis&organism=549.408) | [Galactose/methyl galactoside ABC transport system, D-galactose-binding periplasmic protein MglB (TC 3.A.1.2.3)](https://rast.nmpdr.org/seedviewer.cgi?page=FunctionalRole&role=Galactose/methyl%20galactoside%20ABC%20transport%20system,%20D-galactose-binding%20periplasmic%20protein%20MglB%20(TC%203.A.1.2.3)&subsystem_name=Bacterial_Chemotaxis) |
| Motility and Chemotaxis | Motility and Chemotaxis - no subcategory | [Bacterial Chemotaxis](https://rast.nmpdr.org/seedviewer.cgi?page=Subsystems&subsystem=Bacterial_Chemotaxis&organism=549.408) | [Chemotaxis response regulator protein-glutamate methylesterase CheB (EC 3.1.1.61)](https://rast.nmpdr.org/seedviewer.cgi?page=FunctionalRole&role=Chemotaxis%20response%20regulator%20protein-glutamate%20methylesterase%20CheB%20(EC%203.1.1.61)&subsystem_name=Bacterial_Chemotaxis) |
| Motility and Chemotaxis | Motility and Chemotaxis - no subcategory | [Bacterial Chemotaxis](https://rast.nmpdr.org/seedviewer.cgi?page=Subsystems&subsystem=Bacterial_Chemotaxis&organism=549.408) | [Aerotaxis sensor receptor protein](https://rast.nmpdr.org/seedviewer.cgi?page=FunctionalRole&role=Aerotaxis%20sensor%20receptor%20protein&subsystem_name=Bacterial_Chemotaxis) |
| Motility and Chemotaxis | Motility and Chemotaxis - no subcategory | [Bacterial Chemotaxis](https://rast.nmpdr.org/seedviewer.cgi?page=Subsystems&subsystem=Bacterial_Chemotaxis&organism=549.408) | [Chemotaxis protein methyltransferase CheR (EC 2.1.1.80)](https://rast.nmpdr.org/seedviewer.cgi?page=FunctionalRole&role=Chemotaxis%20protein%20methyltransferase%20CheR%20(EC%202.1.1.80)&subsystem_name=Bacterial_Chemotaxis) |
| Motility and Chemotaxis | Motility and Chemotaxis - no subcategory | [Bacterial Chemotaxis](https://rast.nmpdr.org/seedviewer.cgi?page=Subsystems&subsystem=Bacterial_Chemotaxis&organism=549.408) | [Ribose ABC transporter, periplasmic ribose-binding protein RbsB (TC 3.A.1.2.1)](https://rast.nmpdr.org/seedviewer.cgi?page=FunctionalRole&role=Ribose%20ABC%20transporter,%20periplasmic%20ribose-binding%20protein%20RbsB%20(TC%203.A.1.2.1)&subsystem_name=Bacterial_Chemotaxis) |
| Motility and Chemotaxis | Motility and Chemotaxis - no subcategory | [Bacterial Chemotaxis](https://rast.nmpdr.org/seedviewer.cgi?page=Subsystems&subsystem=Bacterial_Chemotaxis&organism=549.408) | [Dipeptide-binding ABC transporter, periplasmic substrate-binding component (TC 3.A.1.5.2)](https://rast.nmpdr.org/seedviewer.cgi?page=FunctionalRole&role=Dipeptide-binding%20ABC%20transporter,%20periplasmic%20substrate-binding%20component%20(TC%203.A.1.5.2)&subsystem_name=Bacterial_Chemotaxis) |
| Motility and Chemotaxis | Motility and Chemotaxis - no subcategory | [Bacterial Chemotaxis](https://rast.nmpdr.org/seedviewer.cgi?page=Subsystems&subsystem=Bacterial_Chemotaxis&organism=549.408) | [Chemotaxis response - phosphatase CheZ](https://rast.nmpdr.org/seedviewer.cgi?page=FunctionalRole&role=Chemotaxis%20response%20-%20phosphatase%20CheZ&subsystem_name=Bacterial_Chemotaxis) |
| Motility and Chemotaxis | Motility and Chemotaxis - no subcategory | [Bacterial Chemotaxis](https://rast.nmpdr.org/seedviewer.cgi?page=Subsystems&subsystem=Bacterial_Chemotaxis&organism=549.408) | [Flagellar motor switch protein FliM](https://rast.nmpdr.org/seedviewer.cgi?page=FunctionalRole&role=Flagellar%20motor%20switch%20protein%20FliM&subsystem_name=Bacterial_Chemotaxis) |
| Motility and Chemotaxis | Motility and Chemotaxis - no subcategory | [Bacterial Chemotaxis](https://rast.nmpdr.org/seedviewer.cgi?page=Subsystems&subsystem=Bacterial_Chemotaxis&organism=549.408) | [Signal transduction histidine kinase CheA (EC 2.7.3.-)](https://rast.nmpdr.org/seedviewer.cgi?page=FunctionalRole&role=Signal%20transduction%20histidine%20kinase%20CheA%20(EC%202.7.3.-)&subsystem_name=Bacterial_Chemotaxis) |
| Motility and Chemotaxis | Motility and Chemotaxis - no subcategory | [Bacterial Chemotaxis](https://rast.nmpdr.org/seedviewer.cgi?page=Subsystems&subsystem=Bacterial_Chemotaxis&organism=549.408) | [Flagellar motor switch protein FliG](https://rast.nmpdr.org/seedviewer.cgi?page=FunctionalRole&role=Flagellar%20motor%20switch%20protein%20FliG&subsystem_name=Bacterial_Chemotaxis) |
| Motility and Chemotaxis | Motility and Chemotaxis - no subcategory | [Bacterial Chemotaxis](https://rast.nmpdr.org/seedviewer.cgi?page=Subsystems&subsystem=Bacterial_Chemotaxis&organism=549.408) | [Maltose/maltodextrin ABC transporter, substrate binding periplasmic protein MalE](https://rast.nmpdr.org/seedviewer.cgi?page=FunctionalRole&role=Maltose/maltodextrin%20ABC%20transporter,%20substrate%20binding%20periplasmic%20protein%20MalE&subsystem_name=Bacterial_Chemotaxis) |
| Motility and Chemotaxis | Motility and Chemotaxis - no subcategory | [Bacterial Chemotaxis](https://rast.nmpdr.org/seedviewer.cgi?page=Subsystems&subsystem=Bacterial_Chemotaxis&organism=549.408) | [Methyl-accepting chemotaxis protein I (serine chemoreceptor protein)](https://rast.nmpdr.org/seedviewer.cgi?page=FunctionalRole&role=Methyl-accepting%20chemotaxis%20protein%20I%20(serine%20chemoreceptor%20protein)&subsystem_name=Bacterial_Chemotaxis) |
| Motility and Chemotaxis | Motility and Chemotaxis - no subcategory | [Bacterial Chemotaxis](https://rast.nmpdr.org/seedviewer.cgi?page=Subsystems&subsystem=Bacterial_Chemotaxis&organism=549.408) | [Methyl-accepting chemotaxis protein III (ribose and galactose chemoreceptor protein)](https://rast.nmpdr.org/seedviewer.cgi?page=FunctionalRole&role=Methyl-accepting%20chemotaxis%20protein%20III%20(ribose%20and%20galactose%20chemoreceptor%20protein)&subsystem_name=Bacterial_Chemotaxis) |
| Motility and Chemotaxis | Motility and Chemotaxis - no subcategory | [Bacterial Chemotaxis](https://rast.nmpdr.org/seedviewer.cgi?page=Subsystems&subsystem=Bacterial_Chemotaxis&organism=549.408) | [Chemotaxis regulator - transmits chemoreceptor signals to flagelllar motor components CheY](https://rast.nmpdr.org/seedviewer.cgi?page=FunctionalRole&role=Chemotaxis%20regulator%20-%20transmits%20chemoreceptor%20signals%20to%20flagelllar%20motor%20components%20CheY&subsystem_name=Bacterial_Chemotaxis) |
| Motility and Chemotaxis | Flagellar motility in Prokaryota | [Flagellum in Campylobacter](https://rast.nmpdr.org/seedviewer.cgi?page=Subsystems&subsystem=Flagellum_in_Campylobacter&organism=549.408) | [Flagellar basal-body rod protein FlgC](https://rast.nmpdr.org/seedviewer.cgi?page=FunctionalRole&role=Flagellar%20basal-body%20rod%20protein%20FlgC&subsystem_name=Flagellum_in_Campylobacter) |
| Motility and Chemotaxis | Flagellar motility in Prokaryota | [Flagellum in Campylobacter](https://rast.nmpdr.org/seedviewer.cgi?page=Subsystems&subsystem=Flagellum_in_Campylobacter&organism=549.408) | [Flagellin protein FlaA](https://rast.nmpdr.org/seedviewer.cgi?page=FunctionalRole&role=Flagellin%20protein%20FlaA&subsystem_name=Flagellum_in_Campylobacter) |
| Motility and Chemotaxis | Flagellar motility in Prokaryota | [Flagellum in Campylobacter](https://rast.nmpdr.org/seedviewer.cgi?page=Subsystems&subsystem=Flagellum_in_Campylobacter&organism=549.408) | [Cell division protein FtsI [Peptidoglycan synthetase] (EC 2.4.1.129)](https://rast.nmpdr.org/seedviewer.cgi?page=FunctionalRole&role=Cell%20division%20protein%20FtsI%20%5bPeptidoglycan%20synthetase%5d%20(EC%202.4.1.129)&subsystem_name=Flagellum_in_Campylobacter) |
| Motility and Chemotaxis | Flagellar motility in Prokaryota | [Flagellum in Campylobacter](https://rast.nmpdr.org/seedviewer.cgi?page=Subsystems&subsystem=Flagellum_in_Campylobacter&organism=549.408) | [Flagellar hook-basal body complex protein FliE](https://rast.nmpdr.org/seedviewer.cgi?page=FunctionalRole&role=Flagellar%20hook-basal%20body%20complex%20protein%20FliE&subsystem_name=Flagellum_in_Campylobacter) |
| Motility and Chemotaxis | Flagellar motility in Prokaryota | [Flagellum in Campylobacter](https://rast.nmpdr.org/seedviewer.cgi?page=Subsystems&subsystem=Flagellum_in_Campylobacter&organism=549.408) | [Flagellar basal-body rod protein FlgB](https://rast.nmpdr.org/seedviewer.cgi?page=FunctionalRole&role=Flagellar%20basal-body%20rod%20protein%20FlgB&subsystem_name=Flagellum_in_Campylobacter) |
| Motility and Chemotaxis | Flagellar motility in Prokaryota | [Flagellum](https://rast.nmpdr.org/seedviewer.cgi?page=Subsystems&subsystem=Flagellum&organism=549.408) | [RNA polymerase sigma factor RpoD](https://rast.nmpdr.org/seedviewer.cgi?page=FunctionalRole&role=RNA%20polymerase%20sigma%20factor%20RpoD&subsystem_name=Flagellum) |
| Motility and Chemotaxis | Flagellar motility in Prokaryota | [Flagellum](https://rast.nmpdr.org/seedviewer.cgi?page=Subsystems&subsystem=Flagellum&organism=549.408) | [Flagellar biosynthesis protein FliL](https://rast.nmpdr.org/seedviewer.cgi?page=FunctionalRole&role=Flagellar%20biosynthesis%20protein%20FliL&subsystem_name=Flagellum) |
| Motility and Chemotaxis | Flagellar motility in Prokaryota | [Flagellum](https://rast.nmpdr.org/seedviewer.cgi?page=Subsystems&subsystem=Flagellum&organism=549.408) | [Flagellar hook-associated protein FliD](https://rast.nmpdr.org/seedviewer.cgi?page=FunctionalRole&role=Flagellar%20hook-associated%20protein%20FliD&subsystem_name=Flagellum) |
| Motility and Chemotaxis | Flagellar motility in Prokaryota | [Flagellum](https://rast.nmpdr.org/seedviewer.cgi?page=Subsystems&subsystem=Flagellum&organism=549.408) | [Flagellar hook-associated protein FlgK](https://rast.nmpdr.org/seedviewer.cgi?page=FunctionalRole&role=Flagellar%20hook-associated%20protein%20FlgK&subsystem_name=Flagellum) |
| Motility and Chemotaxis | Flagellar motility in Prokaryota | [Flagellum](https://rast.nmpdr.org/seedviewer.cgi?page=Subsystems&subsystem=Flagellum&organism=549.408) | [Flagellar assembly protein FliH](https://rast.nmpdr.org/seedviewer.cgi?page=FunctionalRole&role=Flagellar%20assembly%20protein%20FliH&subsystem_name=Flagellum) |
| Motility and Chemotaxis | Flagellar motility in Prokaryota | [Flagellum](https://rast.nmpdr.org/seedviewer.cgi?page=Subsystems&subsystem=Flagellum&organism=549.408) | [Flagellar biosynthesis protein FliR](https://rast.nmpdr.org/seedviewer.cgi?page=FunctionalRole&role=Flagellar%20biosynthesis%20protein%20FliR&subsystem_name=Flagellum) |
| Motility and Chemotaxis | Flagellar motility in Prokaryota | [Flagellum](https://rast.nmpdr.org/seedviewer.cgi?page=Subsystems&subsystem=Flagellum&organism=549.408) | [Flagellar biosynthesis protein FliP](https://rast.nmpdr.org/seedviewer.cgi?page=FunctionalRole&role=Flagellar%20biosynthesis%20protein%20FliP&subsystem_name=Flagellum) |
| Motility and Chemotaxis | Flagellar motility in Prokaryota | [Flagellum](https://rast.nmpdr.org/seedviewer.cgi?page=Subsystems&subsystem=Flagellum&organism=549.408) | [Flagellar hook protein FlgE](https://rast.nmpdr.org/seedviewer.cgi?page=FunctionalRole&role=Flagellar%20hook%20protein%20FlgE&subsystem_name=Flagellum) |
| Motility and Chemotaxis | Flagellar motility in Prokaryota | [Flagellum](https://rast.nmpdr.org/seedviewer.cgi?page=Subsystems&subsystem=Flagellum&organism=549.408) | [Flagellar M-ring protein FliF](https://rast.nmpdr.org/seedviewer.cgi?page=FunctionalRole&role=Flagellar%20M-ring%20protein%20FliF&subsystem_name=Flagellum) |
| Motility and Chemotaxis | Flagellar motility in Prokaryota | [Flagellum](https://rast.nmpdr.org/seedviewer.cgi?page=Subsystems&subsystem=Flagellum&organism=549.408) | [Flagellar basal-body rod modification protein FlgD](https://rast.nmpdr.org/seedviewer.cgi?page=FunctionalRole&role=Flagellar%20basal-body%20rod%20modification%20protein%20FlgD&subsystem_name=Flagellum) |
| Motility and Chemotaxis | Flagellar motility in Prokaryota | [Flagellum](https://rast.nmpdr.org/seedviewer.cgi?page=Subsystems&subsystem=Flagellum&organism=549.408) | [Flagellar biosynthesis protein FliC](https://rast.nmpdr.org/seedviewer.cgi?page=FunctionalRole&role=Flagellar%20biosynthesis%20protein%20FliC&subsystem_name=Flagellum) |
| Motility and Chemotaxis | Flagellar motility in Prokaryota | [Flagellum](https://rast.nmpdr.org/seedviewer.cgi?page=Subsystems&subsystem=Flagellum&organism=549.408) | [Flagellar P-ring protein FlgI](https://rast.nmpdr.org/seedviewer.cgi?page=FunctionalRole&role=Flagellar%20P-ring%20protein%20FlgI&subsystem_name=Flagellum) |
| Motility and Chemotaxis | Flagellar motility in Prokaryota | [Flagellum](https://rast.nmpdr.org/seedviewer.cgi?page=Subsystems&subsystem=Flagellum&organism=549.408) | [Flagellar basal-body rod protein FlgC](https://rast.nmpdr.org/seedviewer.cgi?page=FunctionalRole&role=Flagellar%20basal-body%20rod%20protein%20FlgC&subsystem_name=Flagellum) |
| Motility and Chemotaxis | Flagellar motility in Prokaryota | [Flagellum](https://rast.nmpdr.org/seedviewer.cgi?page=Subsystems&subsystem=Flagellum&organism=549.408) | [Flagellar biosynthesis protein FlhB](https://rast.nmpdr.org/seedviewer.cgi?page=FunctionalRole&role=Flagellar%20biosynthesis%20protein%20FlhB&subsystem_name=Flagellum) |
| Motility and Chemotaxis | Flagellar motility in Prokaryota | [Flagellum](https://rast.nmpdr.org/seedviewer.cgi?page=Subsystems&subsystem=Flagellum&organism=549.408) | [Flagellar hook-basal body complex protein FliE](https://rast.nmpdr.org/seedviewer.cgi?page=FunctionalRole&role=Flagellar%20hook-basal%20body%20complex%20protein%20FliE&subsystem_name=Flagellum) |
| Motility and Chemotaxis | Flagellar motility in Prokaryota | [Flagellum](https://rast.nmpdr.org/seedviewer.cgi?page=Subsystems&subsystem=Flagellum&organism=549.408) | [Flagellar biosynthesis protein FliZ](https://rast.nmpdr.org/seedviewer.cgi?page=FunctionalRole&role=Flagellar%20biosynthesis%20protein%20FliZ&subsystem_name=Flagellum) |
| Motility and Chemotaxis | Flagellar motility in Prokaryota | [Flagellum](https://rast.nmpdr.org/seedviewer.cgi?page=Subsystems&subsystem=Flagellum&organism=549.408) | [Flagellar basal-body rod protein FlgB](https://rast.nmpdr.org/seedviewer.cgi?page=FunctionalRole&role=Flagellar%20basal-body%20rod%20protein%20FlgB&subsystem_name=Flagellum) |
| Motility and Chemotaxis | Flagellar motility in Prokaryota | [Flagellum](https://rast.nmpdr.org/seedviewer.cgi?page=Subsystems&subsystem=Flagellum&organism=549.408) | [Flagellar biosynthesis protein FliS](https://rast.nmpdr.org/seedviewer.cgi?page=FunctionalRole&role=Flagellar%20biosynthesis%20protein%20FliS&subsystem_name=Flagellum) |
| Motility and Chemotaxis | Flagellar motility in Prokaryota | [Flagellum](https://rast.nmpdr.org/seedviewer.cgi?page=Subsystems&subsystem=Flagellum&organism=549.408) | [Flagellar basal-body rod protein FlgF](https://rast.nmpdr.org/seedviewer.cgi?page=FunctionalRole&role=Flagellar%20basal-body%20rod%20protein%20FlgF&subsystem_name=Flagellum) |
| Motility and Chemotaxis | Flagellar motility in Prokaryota | [Flagellum](https://rast.nmpdr.org/seedviewer.cgi?page=Subsystems&subsystem=Flagellum&organism=549.408) | [Flagellar L-ring protein FlgH](https://rast.nmpdr.org/seedviewer.cgi?page=FunctionalRole&role=Flagellar%20L-ring%20protein%20FlgH&subsystem_name=Flagellum) |
| Motility and Chemotaxis | Flagellar motility in Prokaryota | [Flagellum](https://rast.nmpdr.org/seedviewer.cgi?page=Subsystems&subsystem=Flagellum&organism=549.408) | [Flagellin protein FlaA](https://rast.nmpdr.org/seedviewer.cgi?page=FunctionalRole&role=Flagellin%20protein%20FlaA&subsystem_name=Flagellum) |
| Motility and Chemotaxis | Flagellar motility in Prokaryota | [Flagellum](https://rast.nmpdr.org/seedviewer.cgi?page=Subsystems&subsystem=Flagellum&organism=549.408) | [Flagellar transcriptional activator FlhD](https://rast.nmpdr.org/seedviewer.cgi?page=FunctionalRole&role=Flagellar%20transcriptional%20activator%20FlhD&subsystem_name=Flagellum) |
| Motility and Chemotaxis | Flagellar motility in Prokaryota | [Flagellum](https://rast.nmpdr.org/seedviewer.cgi?page=Subsystems&subsystem=Flagellum&organism=549.408) | [Flagellar protein FlgJ [peptidoglycan hydrolase] (EC 3.2.1.-)](https://rast.nmpdr.org/seedviewer.cgi?page=FunctionalRole&role=Flagellar%20protein%20FlgJ%20%5bpeptidoglycan%20hydrolase%5d%20(EC%203.2.1.-)&subsystem_name=Flagellum) |
| Motility and Chemotaxis | Flagellar motility in Prokaryota | [Flagellum](https://rast.nmpdr.org/seedviewer.cgi?page=Subsystems&subsystem=Flagellum&organism=549.408) | [Flagellar basal-body P-ring formation protein FlgA](https://rast.nmpdr.org/seedviewer.cgi?page=FunctionalRole&role=Flagellar%20basal-body%20P-ring%20formation%20protein%20FlgA&subsystem_name=Flagellum) |
| Motility and Chemotaxis | Flagellar motility in Prokaryota | [Flagellum](https://rast.nmpdr.org/seedviewer.cgi?page=Subsystems&subsystem=Flagellum&organism=549.408) | [Flagellar motor switch protein FliN](https://rast.nmpdr.org/seedviewer.cgi?page=FunctionalRole&role=Flagellar%20motor%20switch%20protein%20FliN&subsystem_name=Flagellum) |
| Motility and Chemotaxis | Flagellar motility in Prokaryota | [Flagellum](https://rast.nmpdr.org/seedviewer.cgi?page=Subsystems&subsystem=Flagellum&organism=549.408) | [Flagellar protein FlhE](https://rast.nmpdr.org/seedviewer.cgi?page=FunctionalRole&role=Flagellar%20protein%20FlhE&subsystem_name=Flagellum) |
| Motility and Chemotaxis | Flagellar motility in Prokaryota | [Flagellum](https://rast.nmpdr.org/seedviewer.cgi?page=Subsystems&subsystem=Flagellum&organism=549.408) | [Flagellar basal-body rod protein FlgG](https://rast.nmpdr.org/seedviewer.cgi?page=FunctionalRole&role=Flagellar%20basal-body%20rod%20protein%20FlgG&subsystem_name=Flagellum) |
| Motility and Chemotaxis | Flagellar motility in Prokaryota | [Flagellum](https://rast.nmpdr.org/seedviewer.cgi?page=Subsystems&subsystem=Flagellum&organism=549.408) | [Flagellar biosynthesis protein FlgN](https://rast.nmpdr.org/seedviewer.cgi?page=FunctionalRole&role=Flagellar%20biosynthesis%20protein%20FlgN&subsystem_name=Flagellum) |
| Motility and Chemotaxis | Flagellar motility in Prokaryota | [Flagellum](https://rast.nmpdr.org/seedviewer.cgi?page=Subsystems&subsystem=Flagellum&organism=549.408) | [RNA polymerase sigma-54 factor RpoN](https://rast.nmpdr.org/seedviewer.cgi?page=FunctionalRole&role=RNA%20polymerase%20sigma-54%20factor%20RpoN&subsystem_name=Flagellum) |
| Motility and Chemotaxis | Flagellar motility in Prokaryota | [Flagellum](https://rast.nmpdr.org/seedviewer.cgi?page=Subsystems&subsystem=Flagellum&organism=549.408) | [Flagellar motor rotation protein MotB](https://rast.nmpdr.org/seedviewer.cgi?page=FunctionalRole&role=Flagellar%20motor%20rotation%20protein%20MotB&subsystem_name=Flagellum) |
| Motility and Chemotaxis | Flagellar motility in Prokaryota | [Flagellum](https://rast.nmpdr.org/seedviewer.cgi?page=Subsystems&subsystem=Flagellum&organism=549.408) | [Flagellar protein FliJ](https://rast.nmpdr.org/seedviewer.cgi?page=FunctionalRole&role=Flagellar%20protein%20FliJ&subsystem_name=Flagellum) |
| Motility and Chemotaxis | Flagellar motility in Prokaryota | [Flagellum](https://rast.nmpdr.org/seedviewer.cgi?page=Subsystems&subsystem=Flagellum&organism=549.408) | [Flagellar hook-associated protein FlgL](https://rast.nmpdr.org/seedviewer.cgi?page=FunctionalRole&role=Flagellar%20hook-associated%20protein%20FlgL&subsystem_name=Flagellum) |
| Motility and Chemotaxis | Flagellar motility in Prokaryota | [Flagellum](https://rast.nmpdr.org/seedviewer.cgi?page=Subsystems&subsystem=Flagellum&organism=549.408) | [Flagellum-specific ATP synthase FliI](https://rast.nmpdr.org/seedviewer.cgi?page=FunctionalRole&role=Flagellum-specific%20ATP%20synthase%20FliI&subsystem_name=Flagellum) |
| Motility and Chemotaxis | Flagellar motility in Prokaryota | [Flagellum](https://rast.nmpdr.org/seedviewer.cgi?page=Subsystems&subsystem=Flagellum&organism=549.408) | [Flagellar biosynthesis protein FlhA](https://rast.nmpdr.org/seedviewer.cgi?page=FunctionalRole&role=Flagellar%20biosynthesis%20protein%20FlhA&subsystem_name=Flagellum) |
| Motility and Chemotaxis | Flagellar motility in Prokaryota | [Flagellum](https://rast.nmpdr.org/seedviewer.cgi?page=Subsystems&subsystem=Flagellum&organism=549.408) | [Flagellar biosynthesis protein FliQ](https://rast.nmpdr.org/seedviewer.cgi?page=FunctionalRole&role=Flagellar%20biosynthesis%20protein%20FliQ&subsystem_name=Flagellum) |
| Motility and Chemotaxis | Flagellar motility in Prokaryota | [Flagellum](https://rast.nmpdr.org/seedviewer.cgi?page=Subsystems&subsystem=Flagellum&organism=549.408) | [Flagellar hook-length control protein FliK](https://rast.nmpdr.org/seedviewer.cgi?page=FunctionalRole&role=Flagellar%20hook-length%20control%20protein%20FliK&subsystem_name=Flagellum) |
| Motility and Chemotaxis | Flagellar motility in Prokaryota | [Flagellum](https://rast.nmpdr.org/seedviewer.cgi?page=Subsystems&subsystem=Flagellum&organism=549.408) | [Flagellar transcriptional activator FlhC](https://rast.nmpdr.org/seedviewer.cgi?page=FunctionalRole&role=Flagellar%20transcriptional%20activator%20FlhC&subsystem_name=Flagellum) |
| Motility and Chemotaxis | Flagellar motility in Prokaryota | [Flagellum](https://rast.nmpdr.org/seedviewer.cgi?page=Subsystems&subsystem=Flagellum&organism=549.408) | [Flagellar motor switch protein FliM](https://rast.nmpdr.org/seedviewer.cgi?page=FunctionalRole&role=Flagellar%20motor%20switch%20protein%20FliM&subsystem_name=Flagellum) |
| Motility and Chemotaxis | Flagellar motility in Prokaryota | [Flagellum](https://rast.nmpdr.org/seedviewer.cgi?page=Subsystems&subsystem=Flagellum&organism=549.408) | [Flagellar motor rotation protein MotA](https://rast.nmpdr.org/seedviewer.cgi?page=FunctionalRole&role=Flagellar%20motor%20rotation%20protein%20MotA&subsystem_name=Flagellum) |
| Motility and Chemotaxis | Flagellar motility in Prokaryota | [Flagellum](https://rast.nmpdr.org/seedviewer.cgi?page=Subsystems&subsystem=Flagellum&organism=549.408) | [Flagellar motor switch protein FliG](https://rast.nmpdr.org/seedviewer.cgi?page=FunctionalRole&role=Flagellar%20motor%20switch%20protein%20FliG&subsystem_name=Flagellum) |
| Motility and Chemotaxis | Flagellar motility in Prokaryota | [Flagellum](https://rast.nmpdr.org/seedviewer.cgi?page=Subsystems&subsystem=Flagellum&organism=549.408) | [Negative regulator of flagellin synthesis FlgM](https://rast.nmpdr.org/seedviewer.cgi?page=FunctionalRole&role=Negative%20regulator%20of%20flagellin%20synthesis%20FlgM&subsystem_name=Flagellum) |
| Motility and Chemotaxis | Flagellar motility in Prokaryota | [Flagellum](https://rast.nmpdr.org/seedviewer.cgi?page=Subsystems&subsystem=Flagellum&organism=549.408) | [RNA polymerase sigma factor for flagellar operon](https://rast.nmpdr.org/seedviewer.cgi?page=FunctionalRole&role=RNA%20polymerase%20sigma%20factor%20for%20flagellar%20operon&subsystem_name=Flagellum) |
| Motility and Chemotaxis | Flagellar motility in Prokaryota | [Flagellum](https://rast.nmpdr.org/seedviewer.cgi?page=Subsystems&subsystem=Flagellum&organism=549.408) | [Flagellar biosynthesis protein FliT](https://rast.nmpdr.org/seedviewer.cgi?page=FunctionalRole&role=Flagellar%20biosynthesis%20protein%20FliT&subsystem_name=Flagellum) |
| Motility and Chemotaxis | Flagellar motility in Prokaryota | [Flagellar motility](https://rast.nmpdr.org/seedviewer.cgi?page=Subsystems&subsystem=Flagellar_motility&organism=549.408) | [Flagellar motor switch protein FliM](https://rast.nmpdr.org/seedviewer.cgi?page=FunctionalRole&role=Flagellar%20motor%20switch%20protein%20FliM&subsystem_name=Flagellar_motility) |
| Motility and Chemotaxis | Flagellar motility in Prokaryota | [Flagellar motility](https://rast.nmpdr.org/seedviewer.cgi?page=Subsystems&subsystem=Flagellar_motility&organism=549.408) | [Flagellar L-ring protein FlgH](https://rast.nmpdr.org/seedviewer.cgi?page=FunctionalRole&role=Flagellar%20L-ring%20protein%20FlgH&subsystem_name=Flagellar_motility) |
| Motility and Chemotaxis | Flagellar motility in Prokaryota | [Flagellar motility](https://rast.nmpdr.org/seedviewer.cgi?page=Subsystems&subsystem=Flagellar_motility&organism=549.408) | [Chemotaxis protein CheV (EC 2.7.3.-)](https://rast.nmpdr.org/seedviewer.cgi?page=FunctionalRole&role=Chemotaxis%20protein%20CheV%20(EC%202.7.3.-)&subsystem_name=Flagellar_motility) |
| Motility and Chemotaxis | Flagellar motility in Prokaryota | [Flagellar motility](https://rast.nmpdr.org/seedviewer.cgi?page=Subsystems&subsystem=Flagellar_motility&organism=549.408) | [Flagellar motor switch protein FliN](https://rast.nmpdr.org/seedviewer.cgi?page=FunctionalRole&role=Flagellar%20motor%20switch%20protein%20FliN&subsystem_name=Flagellar_motility) |
| Motility and Chemotaxis | Flagellar motility in Prokaryota | [Flagellar motility](https://rast.nmpdr.org/seedviewer.cgi?page=Subsystems&subsystem=Flagellar_motility&organism=549.408) | [Flagellar biosynthesis protein FlhB](https://rast.nmpdr.org/seedviewer.cgi?page=FunctionalRole&role=Flagellar%20biosynthesis%20protein%20FlhB&subsystem_name=Flagellar_motility) |
| Motility and Chemotaxis | Flagellar motility in Prokaryota | [Flagellar motility](https://rast.nmpdr.org/seedviewer.cgi?page=Subsystems&subsystem=Flagellar_motility&organism=549.408) | [Signal transduction histidine kinase CheA (EC 2.7.3.-)](https://rast.nmpdr.org/seedviewer.cgi?page=FunctionalRole&role=Signal%20transduction%20histidine%20kinase%20CheA%20(EC%202.7.3.-)&subsystem_name=Flagellar_motility) |
| Motility and Chemotaxis | Flagellar motility in Prokaryota | [Flagellar motility](https://rast.nmpdr.org/seedviewer.cgi?page=Subsystems&subsystem=Flagellar_motility&organism=549.408) | [Flagellar motor rotation protein MotA](https://rast.nmpdr.org/seedviewer.cgi?page=FunctionalRole&role=Flagellar%20motor%20rotation%20protein%20MotA&subsystem_name=Flagellar_motility) |
| Motility and Chemotaxis | Flagellar motility in Prokaryota | [Flagellar motility](https://rast.nmpdr.org/seedviewer.cgi?page=Subsystems&subsystem=Flagellar_motility&organism=549.408) | [Flagellar biosynthesis protein FliR](https://rast.nmpdr.org/seedviewer.cgi?page=FunctionalRole&role=Flagellar%20biosynthesis%20protein%20FliR&subsystem_name=Flagellar_motility) |
| Motility and Chemotaxis | Flagellar motility in Prokaryota | [Flagellar motility](https://rast.nmpdr.org/seedviewer.cgi?page=Subsystems&subsystem=Flagellar_motility&organism=549.408) | [RNA polymerase sigma-54 factor RpoN](https://rast.nmpdr.org/seedviewer.cgi?page=FunctionalRole&role=RNA%20polymerase%20sigma-54%20factor%20RpoN&subsystem_name=Flagellar_motility) |
| Motility and Chemotaxis | Flagellar motility in Prokaryota | [Flagellar motility](https://rast.nmpdr.org/seedviewer.cgi?page=Subsystems&subsystem=Flagellar_motility&organism=549.408) | [Flagellar motor rotation protein MotB](https://rast.nmpdr.org/seedviewer.cgi?page=FunctionalRole&role=Flagellar%20motor%20rotation%20protein%20MotB&subsystem_name=Flagellar_motility) |
| Motility and Chemotaxis | Flagellar motility in Prokaryota | [Flagellar motility](https://rast.nmpdr.org/seedviewer.cgi?page=Subsystems&subsystem=Flagellar_motility&organism=549.408) | [Flagellum-specific ATP synthase FliI](https://rast.nmpdr.org/seedviewer.cgi?page=FunctionalRole&role=Flagellum-specific%20ATP%20synthase%20FliI&subsystem_name=Flagellar_motility) |
| Motility and Chemotaxis | Flagellar motility in Prokaryota | [Flagellar motility](https://rast.nmpdr.org/seedviewer.cgi?page=Subsystems&subsystem=Flagellar_motility&organism=549.408) | [Flagellar biosynthesis protein FlhA](https://rast.nmpdr.org/seedviewer.cgi?page=FunctionalRole&role=Flagellar%20biosynthesis%20protein%20FlhA&subsystem_name=Flagellar_motility) |
| Motility and Chemotaxis | Flagellar motility in Prokaryota | [Flagellar motility](https://rast.nmpdr.org/seedviewer.cgi?page=Subsystems&subsystem=Flagellar_motility&organism=549.408) | [RNA polymerase sigma factor for flagellar operon](https://rast.nmpdr.org/seedviewer.cgi?page=FunctionalRole&role=RNA%20polymerase%20sigma%20factor%20for%20flagellar%20operon&subsystem_name=Flagellar_motility) |
| Motility and Chemotaxis | Flagellar motility in Prokaryota | [Flagellar motility](https://rast.nmpdr.org/seedviewer.cgi?page=Subsystems&subsystem=Flagellar_motility&organism=549.408) | [Flagellar basal-body rod modification protein FlgD](https://rast.nmpdr.org/seedviewer.cgi?page=FunctionalRole&role=Flagellar%20basal-body%20rod%20modification%20protein%20FlgD&subsystem_name=Flagellar_motility) |
| Motility and Chemotaxis | Flagellar motility in Prokaryota | [Flagellar motility](https://rast.nmpdr.org/seedviewer.cgi?page=Subsystems&subsystem=Flagellar_motility&organism=549.408) | [Chemotaxis regulator - transmits chemoreceptor signals to flagelllar motor components CheY](https://rast.nmpdr.org/seedviewer.cgi?page=FunctionalRole&role=Chemotaxis%20regulator%20-%20transmits%20chemoreceptor%20signals%20to%20flagelllar%20motor%20components%20CheY&subsystem_name=Flagellar_motility) |
| Regulation and Cell signaling | Regulation and Cell signaling - no subcategory | [Rcs phosphorelay signal transduction pathway](https://rast.nmpdr.org/seedviewer.cgi?page=Subsystems&subsystem=Rcs_phosphorelay_signal_transduction_pathway&organism=549.408) | [DNA-binding capsular synthesis response regulator RcsB](https://rast.nmpdr.org/seedviewer.cgi?page=FunctionalRole&role=DNA-binding%20capsular%20synthesis%20response%20regulator%20RcsB&subsystem_name=Rcs_phosphorelay_signal_transduction_pathway) |
| Regulation and Cell signaling | Regulation and Cell signaling - no subcategory | [Rcs phosphorelay signal transduction pathway](https://rast.nmpdr.org/seedviewer.cgi?page=Subsystems&subsystem=Rcs_phosphorelay_signal_transduction_pathway&organism=549.408) | [DnaJ-like protein DjlA](https://rast.nmpdr.org/seedviewer.cgi?page=FunctionalRole&role=DnaJ-like%20protein%20DjlA&subsystem_name=Rcs_phosphorelay_signal_transduction_pathway) |
| Regulation and Cell signaling | Regulation and Cell signaling - no subcategory | [Rcs phosphorelay signal transduction pathway](https://rast.nmpdr.org/seedviewer.cgi?page=Subsystems&subsystem=Rcs_phosphorelay_signal_transduction_pathway&organism=549.408) | [Two-component sensor protein RcsC (EC 2.7.3.-)](https://rast.nmpdr.org/seedviewer.cgi?page=FunctionalRole&role=Two-component%20sensor%20protein%20RcsC%20(EC%202.7.3.-)&subsystem_name=Rcs_phosphorelay_signal_transduction_pathway) |
| Regulation and Cell signaling | Regulation and Cell signaling - no subcategory | [Rcs phosphorelay signal transduction pathway](https://rast.nmpdr.org/seedviewer.cgi?page=Subsystems&subsystem=Rcs_phosphorelay_signal_transduction_pathway&organism=549.408) | [Two-component sensor protein RcsD (EC 2.7.3.-)](https://rast.nmpdr.org/seedviewer.cgi?page=FunctionalRole&role=Two-component%20sensor%20protein%20RcsD%20(EC%202.7.3.-)&subsystem_name=Rcs_phosphorelay_signal_transduction_pathway) |
| Regulation and Cell signaling | Regulation and Cell signaling - no subcategory | [Rcs phosphorelay signal transduction pathway](https://rast.nmpdr.org/seedviewer.cgi?page=Subsystems&subsystem=Rcs_phosphorelay_signal_transduction_pathway&organism=549.408) | [Protein RcsF](https://rast.nmpdr.org/seedviewer.cgi?page=FunctionalRole&role=Protein%20RcsF&subsystem_name=Rcs_phosphorelay_signal_transduction_pathway) |
| Regulation and Cell signaling | Regulation and Cell signaling - no subcategory | [Rcs phosphorelay signal transduction pathway](https://rast.nmpdr.org/seedviewer.cgi?page=Subsystems&subsystem=Rcs_phosphorelay_signal_transduction_pathway&organism=549.408) | [Colanic acid capsular biosynthesis activation accesory protein RcsA, co-regulator with RcsB](https://rast.nmpdr.org/seedviewer.cgi?page=FunctionalRole&role=Colanic%20acid%20capsular%20biosynthesis%20activation%20accesory%20protein%20RcsA,%20co-regulator%20with%20RcsB&subsystem_name=Rcs_phosphorelay_signal_transduction_pathway) |
| Regulation and Cell signaling | Regulation and Cell signaling - no subcategory | [cAMP signaling in bacteria](https://rast.nmpdr.org/seedviewer.cgi?page=Subsystems&subsystem=cAMP_signaling_in_bacteria&organism=549.408) | [Hydrolase, alpha/beta fold family functionally coupled to Phosphoribulokinase](https://rast.nmpdr.org/seedviewer.cgi?page=FunctionalRole&role=Hydrolase,%20alpha/beta%20fold%20family%20functionally%20coupled%20to%20Phosphoribulokinase&subsystem_name=cAMP_signaling_in_bacteria) |
| Regulation and Cell signaling | Regulation and Cell signaling - no subcategory | [cAMP signaling in bacteria](https://rast.nmpdr.org/seedviewer.cgi?page=Subsystems&subsystem=cAMP_signaling_in_bacteria&organism=549.408) | [Adenylate cyclase (EC 4.6.1.1)](https://rast.nmpdr.org/seedviewer.cgi?page=FunctionalRole&role=Adenylate%20cyclase%20(EC%204.6.1.1)&subsystem_name=cAMP_signaling_in_bacteria) |
| Regulation and Cell signaling | Regulation and Cell signaling - no subcategory | [cAMP signaling in bacteria](https://rast.nmpdr.org/seedviewer.cgi?page=Subsystems&subsystem=cAMP_signaling_in_bacteria&organism=549.408) | [3',5'-cyclic-nucleotide phosphodiesterase (EC 3.1.4.17)](https://rast.nmpdr.org/seedviewer.cgi?page=FunctionalRole&role=3) |
| Regulation and Cell signaling | Regulation and Cell signaling - no subcategory | [cAMP signaling in bacteria](https://rast.nmpdr.org/seedviewer.cgi?page=Subsystems&subsystem=cAMP_signaling_in_bacteria&organism=549.408) | [Cyclic AMP receptor protein](https://rast.nmpdr.org/seedviewer.cgi?page=FunctionalRole&role=Cyclic%20AMP%20receptor%20protein&subsystem_name=cAMP_signaling_in_bacteria) |
| Regulation and Cell signaling | Regulation and Cell signaling - no subcategory | [cAMP signaling in bacteria](https://rast.nmpdr.org/seedviewer.cgi?page=Subsystems&subsystem=cAMP_signaling_in_bacteria&organism=549.408) | [ElaA protein](https://rast.nmpdr.org/seedviewer.cgi?page=FunctionalRole&role=ElaA%20protein&subsystem_name=cAMP_signaling_in_bacteria) |
| Regulation and Cell signaling | Regulation and Cell signaling - no subcategory | [cAMP signaling in bacteria](https://rast.nmpdr.org/seedviewer.cgi?page=Subsystems&subsystem=cAMP_signaling_in_bacteria&organism=549.408) | [ATP-dependent Clp protease proteolytic subunit (EC 3.4.21.92)](https://rast.nmpdr.org/seedviewer.cgi?page=FunctionalRole&role=ATP-dependent%20Clp%20protease%20proteolytic%20subunit%20(EC%203.4.21.92)&subsystem_name=cAMP_signaling_in_bacteria) |
| Regulation and Cell signaling | Regulation and Cell signaling - no subcategory | [cAMP signaling in bacteria](https://rast.nmpdr.org/seedviewer.cgi?page=Subsystems&subsystem=cAMP_signaling_in_bacteria&organism=549.408) | [Adenine phosphoribosyltransferase (EC 2.4.2.7)](https://rast.nmpdr.org/seedviewer.cgi?page=FunctionalRole&role=Adenine%20phosphoribosyltransferase%20(EC%202.4.2.7)&subsystem_name=cAMP_signaling_in_bacteria) |
| Regulation and Cell signaling | Regulation and Cell signaling - no subcategory | [cAMP signaling in bacteria](https://rast.nmpdr.org/seedviewer.cgi?page=Subsystems&subsystem=cAMP_signaling_in_bacteria&organism=549.408) | [Putative hemin-binding lipoprotein](https://rast.nmpdr.org/seedviewer.cgi?page=FunctionalRole&role=Putative%20hemin-binding%20lipoprotein&subsystem_name=cAMP_signaling_in_bacteria) |
| Regulation and Cell signaling | Regulation and Cell signaling - no subcategory | [LysR-family proteins in Salmonella enterica Typhimurium](https://rast.nmpdr.org/seedviewer.cgi?page=Subsystems&subsystem=LysR-family_proteins_in_Salmonella_enterica_Typhimurium&organism=549.408) | [LysR-family transcriptional regulator YdhB](https://rast.nmpdr.org/seedviewer.cgi?page=FunctionalRole&role=LysR-family%20transcriptional%20regulator%20YdhB&subsystem_name=LysR-family_proteins_in_Salmonella_enterica_Typhimurium) |
| Regulation and Cell signaling | Regulation and Cell signaling - no subcategory | [LysR-family proteins in Salmonella enterica Typhimurium](https://rast.nmpdr.org/seedviewer.cgi?page=Subsystems&subsystem=LysR-family_proteins_in_Salmonella_enterica_Typhimurium&organism=549.408) | [LysR family transcriptional regulator YneJ](https://rast.nmpdr.org/seedviewer.cgi?page=FunctionalRole&role=LysR%20family%20transcriptional%20regulator%20YneJ&subsystem_name=LysR-family_proteins_in_Salmonella_enterica_Typhimurium) |
| Regulation and Cell signaling | Regulation and Cell signaling - no subcategory | [LysR-family proteins in Salmonella enterica Typhimurium](https://rast.nmpdr.org/seedviewer.cgi?page=Subsystems&subsystem=LysR-family_proteins_in_Salmonella_enterica_Typhimurium&organism=549.408) | [LysR-family transcriptional regulator YhaJ](https://rast.nmpdr.org/seedviewer.cgi?page=FunctionalRole&role=LysR-family%20transcriptional%20regulator%20YhaJ&subsystem_name=LysR-family_proteins_in_Salmonella_enterica_Typhimurium) |
| Regulation and Cell signaling | Regulation and Cell signaling - no subcategory | [LysR-family proteins in Salmonella enterica Typhimurium](https://rast.nmpdr.org/seedviewer.cgi?page=Subsystems&subsystem=LysR-family_proteins_in_Salmonella_enterica_Typhimurium&organism=549.408) | [LysR family transcriptional regulator YnfL](https://rast.nmpdr.org/seedviewer.cgi?page=FunctionalRole&role=LysR%20family%20transcriptional%20regulator%20YnfL&subsystem_name=LysR-family_proteins_in_Salmonella_enterica_Typhimurium) |
| Regulation and Cell signaling | Regulation and Cell signaling - no subcategory | [LysR-family proteins in Salmonella enterica Typhimurium](https://rast.nmpdr.org/seedviewer.cgi?page=Subsystems&subsystem=LysR-family_proteins_in_Salmonella_enterica_Typhimurium&organism=549.408) | [Hydrogen peroxide-inducible genes activator](https://rast.nmpdr.org/seedviewer.cgi?page=FunctionalRole&role=Hydrogen%20peroxide-inducible%20genes%20activator&subsystem_name=LysR-family_proteins_in_Salmonella_enterica_Typhimurium) |
| Regulation and Cell signaling | Regulation and Cell signaling - no subcategory | [LysR-family proteins in Salmonella enterica Typhimurium](https://rast.nmpdr.org/seedviewer.cgi?page=Subsystems&subsystem=LysR-family_proteins_in_Salmonella_enterica_Typhimurium&organism=549.408) | [LysR family transcriptional regulator STM3121](https://rast.nmpdr.org/seedviewer.cgi?page=FunctionalRole&role=LysR%20family%20transcriptional%20regulator%20STM3121&subsystem_name=LysR-family_proteins_in_Salmonella_enterica_Typhimurium) |
| Regulation and Cell signaling | Regulation and Cell signaling - no subcategory | [LysR-family proteins in Salmonella enterica Typhimurium](https://rast.nmpdr.org/seedviewer.cgi?page=Subsystems&subsystem=LysR-family_proteins_in_Salmonella_enterica_Typhimurium&organism=549.408) | [LysR family transcriptional regulator YfeR](https://rast.nmpdr.org/seedviewer.cgi?page=FunctionalRole&role=LysR%20family%20transcriptional%20regulator%20YfeR&subsystem_name=LysR-family_proteins_in_Salmonella_enterica_Typhimurium) |
| Regulation and Cell signaling | Regulation and Cell signaling - no subcategory | [LysR-family proteins in Salmonella enterica Typhimurium](https://rast.nmpdr.org/seedviewer.cgi?page=Subsystems&subsystem=LysR-family_proteins_in_Salmonella_enterica_Typhimurium&organism=549.408) | [Transcriptional activator protein LysR](https://rast.nmpdr.org/seedviewer.cgi?page=FunctionalRole&role=Transcriptional%20activator%20protein%20LysR&subsystem_name=LysR-family_proteins_in_Salmonella_enterica_Typhimurium) |
| Regulation and Cell signaling | Regulation and Cell signaling - no subcategory | [LysR-family proteins in Salmonella enterica Typhimurium](https://rast.nmpdr.org/seedviewer.cgi?page=Subsystems&subsystem=LysR-family_proteins_in_Salmonella_enterica_Typhimurium&organism=549.408) | [LysR-family transcriptional regulator STM3020](https://rast.nmpdr.org/seedviewer.cgi?page=FunctionalRole&role=LysR-family%20transcriptional%20regulator%20STM3020&subsystem_name=LysR-family_proteins_in_Salmonella_enterica_Typhimurium) |
| Regulation and Cell signaling | Regulation and Cell signaling - no subcategory | [LysR-family proteins in Salmonella enterica Typhimurium](https://rast.nmpdr.org/seedviewer.cgi?page=Subsystems&subsystem=LysR-family_proteins_in_Salmonella_enterica_Typhimurium&organism=549.408) | [LysR family transcriptional regulator YdcI](https://rast.nmpdr.org/seedviewer.cgi?page=FunctionalRole&role=LysR%20family%20transcriptional%20regulator%20YdcI&subsystem_name=LysR-family_proteins_in_Salmonella_enterica_Typhimurium) |
| Regulation and Cell signaling | Regulation and Cell signaling - no subcategory | [LysR-family proteins in Salmonella enterica Typhimurium](https://rast.nmpdr.org/seedviewer.cgi?page=Subsystems&subsystem=LysR-family_proteins_in_Salmonella_enterica_Typhimurium&organism=549.408) | [LysR family transcriptional regulator lrhA](https://rast.nmpdr.org/seedviewer.cgi?page=FunctionalRole&role=LysR%20family%20transcriptional%20regulator%20lrhA&subsystem_name=LysR-family_proteins_in_Salmonella_enterica_Typhimurium) |
| Regulation and Cell signaling | Regulation and Cell signaling - no subcategory | [LysR-family proteins in Salmonella enterica Typhimurium](https://rast.nmpdr.org/seedviewer.cgi?page=Subsystems&subsystem=LysR-family_proteins_in_Salmonella_enterica_Typhimurium&organism=549.408) | [HTH-type transcriptional regulator IlvY](https://rast.nmpdr.org/seedviewer.cgi?page=FunctionalRole&role=HTH-type%20transcriptional%20regulator%20IlvY&subsystem_name=LysR-family_proteins_in_Salmonella_enterica_Typhimurium) |
| Regulation and Cell signaling | Regulation and Cell signaling - no subcategory | [LysR-family proteins in Salmonella enterica Typhimurium](https://rast.nmpdr.org/seedviewer.cgi?page=Subsystems&subsystem=LysR-family_proteins_in_Salmonella_enterica_Typhimurium&organism=549.408) | [LysR family transcriptional regulator YafC](https://rast.nmpdr.org/seedviewer.cgi?page=FunctionalRole&role=LysR%20family%20transcriptional%20regulator%20YafC&subsystem_name=LysR-family_proteins_in_Salmonella_enterica_Typhimurium) |
| Regulation and Cell signaling | Regulation and Cell signaling - no subcategory | [LysR-family proteins in Salmonella enterica Typhimurium](https://rast.nmpdr.org/seedviewer.cgi?page=Subsystems&subsystem=LysR-family_proteins_in_Salmonella_enterica_Typhimurium&organism=549.408) | [Transcriptional activator MetR](https://rast.nmpdr.org/seedviewer.cgi?page=FunctionalRole&role=Transcriptional%20activator%20MetR&subsystem_name=LysR-family_proteins_in_Salmonella_enterica_Typhimurium) |
| Regulation and Cell signaling | Regulation and Cell signaling - no subcategory | [LysR-family proteins in Salmonella enterica Typhimurium](https://rast.nmpdr.org/seedviewer.cgi?page=Subsystems&subsystem=LysR-family_proteins_in_Salmonella_enterica_Typhimurium&organism=549.408) | [LysR family transcriptional regulator STM2281](https://rast.nmpdr.org/seedviewer.cgi?page=FunctionalRole&role=LysR%20family%20transcriptional%20regulator%20STM2281&subsystem_name=LysR-family_proteins_in_Salmonella_enterica_Typhimurium) |
| Regulation and Cell signaling | Regulation and Cell signaling - no subcategory | [LysR-family proteins in Salmonella enterica Typhimurium](https://rast.nmpdr.org/seedviewer.cgi?page=Subsystems&subsystem=LysR-family_proteins_in_Salmonella_enterica_Typhimurium&organism=549.408) | [LysR family transcriptional regulator QseA](https://rast.nmpdr.org/seedviewer.cgi?page=FunctionalRole&role=LysR%20family%20transcriptional%20regulator%20QseA&subsystem_name=LysR-family_proteins_in_Salmonella_enterica_Typhimurium) |
| Regulation and Cell signaling | Regulation and Cell signaling - no subcategory | [CytR regulation](https://rast.nmpdr.org/seedviewer.cgi?page=Subsystems&subsystem=CytR_regulation&organism=549.408) | [Cyclic AMP receptor protein](https://rast.nmpdr.org/seedviewer.cgi?page=FunctionalRole&role=Cyclic%20AMP%20receptor%20protein&subsystem_name=CytR_regulation) |
| Regulation and Cell signaling | Regulation and Cell signaling - no subcategory | [CytR regulation](https://rast.nmpdr.org/seedviewer.cgi?page=Subsystems&subsystem=CytR_regulation&organism=549.408) | [Transcriptional (co)regulator CytR](https://rast.nmpdr.org/seedviewer.cgi?page=FunctionalRole&role=Transcriptional%20(co)regulator%20CytR&subsystem_name=CytR_regulation) |
| Regulation and Cell signaling | Regulation and Cell signaling - no subcategory | [Orphan regulatory proteins](https://rast.nmpdr.org/seedviewer.cgi?page=Subsystems&subsystem=Orphan_regulatory_proteins&organism=549.408) | [Putative sensory histidine kinase YfhA](https://rast.nmpdr.org/seedviewer.cgi?page=FunctionalRole&role=Putative%20sensory%20histidine%20kinase%20YfhA&subsystem_name=Orphan_regulatory_proteins) |
| Regulation and Cell signaling | Regulation and Cell signaling - no subcategory | [Orphan regulatory proteins](https://rast.nmpdr.org/seedviewer.cgi?page=Subsystems&subsystem=Orphan_regulatory_proteins&organism=549.408) | [Two-component system response regulator QseB](https://rast.nmpdr.org/seedviewer.cgi?page=FunctionalRole&role=Two-component%20system%20response%20regulator%20QseB&subsystem_name=Orphan_regulatory_proteins) |
| Regulation and Cell signaling | Regulation and Cell signaling - no subcategory | [Orphan regulatory proteins](https://rast.nmpdr.org/seedviewer.cgi?page=Subsystems&subsystem=Orphan_regulatory_proteins&organism=549.408) | [Sensory histidine kinase QseC](https://rast.nmpdr.org/seedviewer.cgi?page=FunctionalRole&role=Sensory%20histidine%20kinase%20QseC&subsystem_name=Orphan_regulatory_proteins) |
| Regulation and Cell signaling | Regulation and Cell signaling - no subcategory | [Orphan regulatory proteins](https://rast.nmpdr.org/seedviewer.cgi?page=Subsystems&subsystem=Orphan_regulatory_proteins&organism=549.408) | [DNA transformation protein TfoX](https://rast.nmpdr.org/seedviewer.cgi?page=FunctionalRole&role=DNA%20transformation%20protein%20TfoX&subsystem_name=Orphan_regulatory_proteins) |
| Regulation and Cell signaling | Regulation and Cell signaling - no subcategory | [Orphan regulatory proteins](https://rast.nmpdr.org/seedviewer.cgi?page=Subsystems&subsystem=Orphan_regulatory_proteins&organism=549.408) | [Copper-sensing two-component system response regulator CpxR](https://rast.nmpdr.org/seedviewer.cgi?page=FunctionalRole&role=Copper-sensing%20two-component%20system%20response%20regulator%20CpxR&subsystem_name=Orphan_regulatory_proteins) |
| Regulation and Cell signaling | Regulation and Cell signaling - no subcategory | [Orphan regulatory proteins](https://rast.nmpdr.org/seedviewer.cgi?page=Subsystems&subsystem=Orphan_regulatory_proteins&organism=549.408) | [Copper sensory histidine kinase CpxA](https://rast.nmpdr.org/seedviewer.cgi?page=FunctionalRole&role=Copper%20sensory%20histidine%20kinase%20CpxA&subsystem_name=Orphan_regulatory_proteins) |
| Regulation and Cell signaling | Regulation and Cell signaling - no subcategory | [Orphan regulatory proteins](https://rast.nmpdr.org/seedviewer.cgi?page=Subsystems&subsystem=Orphan_regulatory_proteins&organism=549.408) | [Transcriptional regulatory protein RstA](https://rast.nmpdr.org/seedviewer.cgi?page=FunctionalRole&role=Transcriptional%20regulatory%20protein%20RstA&subsystem_name=Orphan_regulatory_proteins) |
| Regulation and Cell signaling | Regulation and Cell signaling - no subcategory | [Orphan regulatory proteins](https://rast.nmpdr.org/seedviewer.cgi?page=Subsystems&subsystem=Orphan_regulatory_proteins&organism=549.408) | [Sensory histidine kinase in two-component regulatory system with RstA](https://rast.nmpdr.org/seedviewer.cgi?page=FunctionalRole&role=Sensory%20histidine%20kinase%20in%20two-component%20regulatory%20system%20with%20RstA&subsystem_name=Orphan_regulatory_proteins) |
| Regulation and Cell signaling | Regulation and Cell signaling - no subcategory | [Orphan regulatory proteins](https://rast.nmpdr.org/seedviewer.cgi?page=Subsystems&subsystem=Orphan_regulatory_proteins&organism=549.408) | [Glycine cleavage system transcriptional activator GcvA](https://rast.nmpdr.org/seedviewer.cgi?page=FunctionalRole&role=Glycine%20cleavage%20system%20transcriptional%20activator%20GcvA&subsystem_name=Orphan_regulatory_proteins) |
| Regulation and Cell signaling | Regulation and Cell signaling - no subcategory | [Orphan regulatory proteins](https://rast.nmpdr.org/seedviewer.cgi?page=Subsystems&subsystem=Orphan_regulatory_proteins&organism=549.408) | [Transcriptional regulatory protein basR/pmrA](https://rast.nmpdr.org/seedviewer.cgi?page=FunctionalRole&role=Transcriptional%20regulatory%20protein%20basR/pmrA&subsystem_name=Orphan_regulatory_proteins) |
| Regulation and Cell signaling | Regulation and Cell signaling - no subcategory | [Orphan regulatory proteins](https://rast.nmpdr.org/seedviewer.cgi?page=Subsystems&subsystem=Orphan_regulatory_proteins&organism=549.408) | [Putative two component system histidine kinase YedV](https://rast.nmpdr.org/seedviewer.cgi?page=FunctionalRole&role=Putative%20two%20component%20system%20histidine%20kinase%20YedV&subsystem_name=Orphan_regulatory_proteins) |
| Regulation and Cell signaling | Regulation and Cell signaling - no subcategory | [Orphan regulatory proteins](https://rast.nmpdr.org/seedviewer.cgi?page=Subsystems&subsystem=Orphan_regulatory_proteins&organism=549.408) | [Putative sensor-like histidine kinase YfhK](https://rast.nmpdr.org/seedviewer.cgi?page=FunctionalRole&role=Putative%20sensor-like%20histidine%20kinase%20YfhK&subsystem_name=Orphan_regulatory_proteins) |
| Regulation and Cell signaling | Regulation and Cell signaling - no subcategory | [Orphan regulatory proteins](https://rast.nmpdr.org/seedviewer.cgi?page=Subsystems&subsystem=Orphan_regulatory_proteins&organism=549.408) | [Sensor protein basS/pmrB (EC 2.7.3.-)](https://rast.nmpdr.org/seedviewer.cgi?page=FunctionalRole&role=Sensor%20protein%20basS/pmrB%20(EC%202.7.3.-)&subsystem_name=Orphan_regulatory_proteins) |
| Regulation and Cell signaling | Regulation and Cell signaling - no subcategory | [Orphan regulatory proteins](https://rast.nmpdr.org/seedviewer.cgi?page=Subsystems&subsystem=Orphan_regulatory_proteins&organism=549.408) | [Glycine cleavage system transcriptional activator](https://rast.nmpdr.org/seedviewer.cgi?page=FunctionalRole&role=Glycine%20cleavage%20system%20transcriptional%20activator&subsystem_name=Orphan_regulatory_proteins) |
| Regulation and Cell signaling | Regulation and Cell signaling - no subcategory | [Orphan regulatory proteins](https://rast.nmpdr.org/seedviewer.cgi?page=Subsystems&subsystem=Orphan_regulatory_proteins&organism=549.408) | [Glycine cleavage system transcriptional antiactivator GcvR](https://rast.nmpdr.org/seedviewer.cgi?page=FunctionalRole&role=Glycine%20cleavage%20system%20transcriptional%20antiactivator%20GcvR&subsystem_name=Orphan_regulatory_proteins) |
| Regulation and Cell signaling | Regulation and Cell signaling - no subcategory | [LysR-family proteins in Escherichia coli](https://rast.nmpdr.org/seedviewer.cgi?page=Subsystems&subsystem=LysR-family_proteins_in_Escherichia_coli&organism=549.408) | [Alkanesulfonate utilization operon LysR-family regulator CbI](https://rast.nmpdr.org/seedviewer.cgi?page=FunctionalRole&role=Alkanesulfonate%20utilization%20operon%20LysR-family%20regulator%20CbI&subsystem_name=LysR-family_proteins_in_Escherichia_coli) |
| Regulation and Cell signaling | Regulation and Cell signaling - no subcategory | [LysR-family proteins in Escherichia coli](https://rast.nmpdr.org/seedviewer.cgi?page=Subsystems&subsystem=LysR-family_proteins_in_Escherichia_coli&organism=549.408) | [LysR family transcriptional regulator YneJ](https://rast.nmpdr.org/seedviewer.cgi?page=FunctionalRole&role=LysR%20family%20transcriptional%20regulator%20YneJ&subsystem_name=LysR-family_proteins_in_Escherichia_coli) |
| Regulation and Cell signaling | Regulation and Cell signaling - no subcategory | [LysR-family proteins in Escherichia coli](https://rast.nmpdr.org/seedviewer.cgi?page=Subsystems&subsystem=LysR-family_proteins_in_Escherichia_coli&organism=549.408) | [LysR-family transcriptional regulator YhaJ](https://rast.nmpdr.org/seedviewer.cgi?page=FunctionalRole&role=LysR-family%20transcriptional%20regulator%20YhaJ&subsystem_name=LysR-family_proteins_in_Escherichia_coli) |
| Regulation and Cell signaling | Regulation and Cell signaling - no subcategory | [LysR-family proteins in Escherichia coli](https://rast.nmpdr.org/seedviewer.cgi?page=Subsystems&subsystem=LysR-family_proteins_in_Escherichia_coli&organism=549.408) | [Cys regulon transcriptional activator CysB](https://rast.nmpdr.org/seedviewer.cgi?page=FunctionalRole&role=Cys%20regulon%20transcriptional%20activator%20CysB&subsystem_name=LysR-family_proteins_in_Escherichia_coli) |
| Regulation and Cell signaling | Regulation and Cell signaling - no subcategory | [LysR-family proteins in Escherichia coli](https://rast.nmpdr.org/seedviewer.cgi?page=Subsystems&subsystem=LysR-family_proteins_in_Escherichia_coli&organism=549.408) | [LysR family transcriptional regulator YnfL](https://rast.nmpdr.org/seedviewer.cgi?page=FunctionalRole&role=LysR%20family%20transcriptional%20regulator%20YnfL&subsystem_name=LysR-family_proteins_in_Escherichia_coli) |
| Regulation and Cell signaling | Regulation and Cell signaling - no subcategory | [LysR-family proteins in Escherichia coli](https://rast.nmpdr.org/seedviewer.cgi?page=Subsystems&subsystem=LysR-family_proteins_in_Escherichia_coli&organism=549.408) | [LysR family transcriptional regulator YbhD](https://rast.nmpdr.org/seedviewer.cgi?page=FunctionalRole&role=LysR%20family%20transcriptional%20regulator%20YbhD&subsystem_name=LysR-family_proteins_in_Escherichia_coli) |
| Regulation and Cell signaling | Regulation and Cell signaling - no subcategory | [LysR-family proteins in Escherichia coli](https://rast.nmpdr.org/seedviewer.cgi?page=Subsystems&subsystem=LysR-family_proteins_in_Escherichia_coli&organism=549.408) | [Hydrogen peroxide-inducible genes activator](https://rast.nmpdr.org/seedviewer.cgi?page=FunctionalRole&role=Hydrogen%20peroxide-inducible%20genes%20activator&subsystem_name=LysR-family_proteins_in_Escherichia_coli) |
| Regulation and Cell signaling | Regulation and Cell signaling - no subcategory | [LysR-family proteins in Escherichia coli](https://rast.nmpdr.org/seedviewer.cgi?page=Subsystems&subsystem=LysR-family_proteins_in_Escherichia_coli&organism=549.408) | [Nitrogen assimilation regulatory protein Nac](https://rast.nmpdr.org/seedviewer.cgi?page=FunctionalRole&role=Nitrogen%20assimilation%20regulatory%20protein%20Nac&subsystem_name=LysR-family_proteins_in_Escherichia_coli) |
| Regulation and Cell signaling | Regulation and Cell signaling - no subcategory | [LysR-family proteins in Escherichia coli](https://rast.nmpdr.org/seedviewer.cgi?page=Subsystems&subsystem=LysR-family_proteins_in_Escherichia_coli&organism=549.408) | [LysR family transcriptional regulator YfeR](https://rast.nmpdr.org/seedviewer.cgi?page=FunctionalRole&role=LysR%20family%20transcriptional%20regulator%20YfeR&subsystem_name=LysR-family_proteins_in_Escherichia_coli) |
| Regulation and Cell signaling | Regulation and Cell signaling - no subcategory | [LysR-family proteins in Escherichia coli](https://rast.nmpdr.org/seedviewer.cgi?page=Subsystems&subsystem=LysR-family_proteins_in_Escherichia_coli&organism=549.408) | [Transcriptional activator protein LysR](https://rast.nmpdr.org/seedviewer.cgi?page=FunctionalRole&role=Transcriptional%20activator%20protein%20LysR&subsystem_name=LysR-family_proteins_in_Escherichia_coli) |
| Regulation and Cell signaling | Regulation and Cell signaling - no subcategory | [LysR-family proteins in Escherichia coli](https://rast.nmpdr.org/seedviewer.cgi?page=Subsystems&subsystem=LysR-family_proteins_in_Escherichia_coli&organism=549.408) | [LysR family transcriptional regulator YdcI](https://rast.nmpdr.org/seedviewer.cgi?page=FunctionalRole&role=LysR%20family%20transcriptional%20regulator%20YdcI&subsystem_name=LysR-family_proteins_in_Escherichia_coli) |
| Regulation and Cell signaling | Regulation and Cell signaling - no subcategory | [LysR-family proteins in Escherichia coli](https://rast.nmpdr.org/seedviewer.cgi?page=Subsystems&subsystem=LysR-family_proteins_in_Escherichia_coli&organism=549.408) | [LysR family transcriptional regulator YhjC](https://rast.nmpdr.org/seedviewer.cgi?page=FunctionalRole&role=LysR%20family%20transcriptional%20regulator%20YhjC&subsystem_name=LysR-family_proteins_in_Escherichia_coli) |
| Regulation and Cell signaling | Regulation and Cell signaling - no subcategory | [LysR-family proteins in Escherichia coli](https://rast.nmpdr.org/seedviewer.cgi?page=Subsystems&subsystem=LysR-family_proteins_in_Escherichia_coli&organism=549.408) | [LysR family transcriptional regulator YeiE](https://rast.nmpdr.org/seedviewer.cgi?page=FunctionalRole&role=LysR%20family%20transcriptional%20regulator%20YeiE&subsystem_name=LysR-family_proteins_in_Escherichia_coli) |
| Regulation and Cell signaling | Regulation and Cell signaling - no subcategory | [LysR-family proteins in Escherichia coli](https://rast.nmpdr.org/seedviewer.cgi?page=Subsystems&subsystem=LysR-family_proteins_in_Escherichia_coli&organism=549.408) | [LysR family transcriptional regulator lrhA](https://rast.nmpdr.org/seedviewer.cgi?page=FunctionalRole&role=LysR%20family%20transcriptional%20regulator%20lrhA&subsystem_name=LysR-family_proteins_in_Escherichia_coli) |
| Regulation and Cell signaling | Regulation and Cell signaling - no subcategory | [LysR-family proteins in Escherichia coli](https://rast.nmpdr.org/seedviewer.cgi?page=Subsystems&subsystem=LysR-family_proteins_in_Escherichia_coli&organism=549.408) | [Regulatory protein (induces abgABT, used to catabolize p-aminobenzoyl-glutamate)](https://rast.nmpdr.org/seedviewer.cgi?page=FunctionalRole&role=Regulatory%20protein%20(induces%20abgABT,%20used%20to%20catabolize%20p-aminobenzoyl-glutamate)&subsystem_name=LysR-family_proteins_in_Escherichia_coli) |
| Regulation and Cell signaling | Regulation and Cell signaling - no subcategory | [LysR-family proteins in Escherichia coli](https://rast.nmpdr.org/seedviewer.cgi?page=Subsystems&subsystem=LysR-family_proteins_in_Escherichia_coli&organism=549.408) | [Glycine cleavage system transcriptional activator GcvA](https://rast.nmpdr.org/seedviewer.cgi?page=FunctionalRole&role=Glycine%20cleavage%20system%20transcriptional%20activator%20GcvA&subsystem_name=LysR-family_proteins_in_Escherichia_coli) |
| Regulation and Cell signaling | Regulation and Cell signaling - no subcategory | [LysR-family proteins in Escherichia coli](https://rast.nmpdr.org/seedviewer.cgi?page=Subsystems&subsystem=LysR-family_proteins_in_Escherichia_coli&organism=549.408) | [HTH-type transcriptional regulator IlvY](https://rast.nmpdr.org/seedviewer.cgi?page=FunctionalRole&role=HTH-type%20transcriptional%20regulator%20IlvY&subsystem_name=LysR-family_proteins_in_Escherichia_coli) |
| Regulation and Cell signaling | Regulation and Cell signaling - no subcategory | [LysR-family proteins in Escherichia coli](https://rast.nmpdr.org/seedviewer.cgi?page=Subsystems&subsystem=LysR-family_proteins_in_Escherichia_coli&organism=549.408) | [Transcriptional activator MetR](https://rast.nmpdr.org/seedviewer.cgi?page=FunctionalRole&role=Transcriptional%20activator%20MetR&subsystem_name=LysR-family_proteins_in_Escherichia_coli) |
| Regulation and Cell signaling | Regulation and Cell signaling - no subcategory | [LysR-family proteins in Escherichia coli](https://rast.nmpdr.org/seedviewer.cgi?page=Subsystems&subsystem=LysR-family_proteins_in_Escherichia_coli&organism=549.408) | [LysR family transcriptional regulator YafC](https://rast.nmpdr.org/seedviewer.cgi?page=FunctionalRole&role=LysR%20family%20transcriptional%20regulator%20YafC&subsystem_name=LysR-family_proteins_in_Escherichia_coli) |
| Regulation and Cell signaling | Regulation and Cell signaling - no subcategory | [LysR-family proteins in Escherichia coli](https://rast.nmpdr.org/seedviewer.cgi?page=Subsystems&subsystem=LysR-family_proteins_in_Escherichia_coli&organism=549.408) | [Chromosome initiation inhibitor](https://rast.nmpdr.org/seedviewer.cgi?page=FunctionalRole&role=Chromosome%20initiation%20inhibitor&subsystem_name=LysR-family_proteins_in_Escherichia_coli) |
| Regulation and Cell signaling | Regulation and Cell signaling - no subcategory | [LysR-family proteins in Escherichia coli](https://rast.nmpdr.org/seedviewer.cgi?page=Subsystems&subsystem=LysR-family_proteins_in_Escherichia_coli&organism=549.408) | [LysR family transcriptional regulator QseA](https://rast.nmpdr.org/seedviewer.cgi?page=FunctionalRole&role=LysR%20family%20transcriptional%20regulator%20QseA&subsystem_name=LysR-family_proteins_in_Escherichia_coli) |
| Regulation and Cell signaling | Regulation and Cell signaling - no subcategory | [Stringent Response, (p)ppGpp metabolism](https://rast.nmpdr.org/seedviewer.cgi?page=Subsystems&subsystem=Stringent_Response,_(p)ppGpp_metabolism&organism=549.408) | [Guanosine-5'-triphosphate,3'-diphosphate pyrophosphatase (EC 3.6.1.40)](https://rast.nmpdr.org/seedviewer.cgi?page=FunctionalRole&role=Guanosine-5) |
| Regulation and Cell signaling | Regulation and Cell signaling - no subcategory | [Stringent Response, (p)ppGpp metabolism](https://rast.nmpdr.org/seedviewer.cgi?page=Subsystems&subsystem=Stringent_Response,_(p)ppGpp_metabolism&organism=549.408) | [GTP pyrophosphokinase (EC 2.7.6.5), (p)ppGpp synthetase II](https://rast.nmpdr.org/seedviewer.cgi?page=FunctionalRole&role=GTP%20pyrophosphokinase%20(EC%202.7.6.5),%20(p)ppGpp%20synthetase%20II&subsystem_name=Stringent_Response,_(p)ppGpp_metabolism) |
| Regulation and Cell signaling | Regulation and Cell signaling - no subcategory | [Stringent Response, (p)ppGpp metabolism](https://rast.nmpdr.org/seedviewer.cgi?page=Subsystems&subsystem=Stringent_Response,_(p)ppGpp_metabolism&organism=549.408) | [Guanosine-3',5'-bis(diphosphate) 3'-pyrophosphohydrolase (EC 3.1.7.2)](https://rast.nmpdr.org/seedviewer.cgi?page=FunctionalRole&role=Guanosine-3) |
| Regulation and Cell signaling | Regulation and Cell signaling - no subcategory | [Stringent Response, (p)ppGpp metabolism](https://rast.nmpdr.org/seedviewer.cgi?page=Subsystems&subsystem=Stringent_Response,_(p)ppGpp_metabolism&organism=549.408) | [GTP pyrophosphokinase (EC 2.7.6.5), (p)ppGpp synthetase I](https://rast.nmpdr.org/seedviewer.cgi?page=FunctionalRole&role=GTP%20pyrophosphokinase%20(EC%202.7.6.5),%20(p)ppGpp%20synthetase%20I&subsystem_name=Stringent_Response,_(p)ppGpp_metabolism) |
| Regulation and Cell signaling | Regulation and Cell signaling - no subcategory | [Zinc regulated enzymes](https://rast.nmpdr.org/seedviewer.cgi?page=Subsystems&subsystem=Zinc_regulated_enzymes&organism=549.408) | [Queuosine biosynthesis QueD, PTPS-I](https://rast.nmpdr.org/seedviewer.cgi?page=FunctionalRole&role=Queuosine%20biosynthesis%20QueD,%20PTPS-I&subsystem_name=Zinc_regulated_enzymes) |
| Regulation and Cell signaling | Regulation and Cell signaling - no subcategory | [Zinc regulated enzymes](https://rast.nmpdr.org/seedviewer.cgi?page=Subsystems&subsystem=Zinc_regulated_enzymes&organism=549.408) | [Carbonic anhydrase (EC 4.2.1.1)](https://rast.nmpdr.org/seedviewer.cgi?page=FunctionalRole&role=Carbonic%20anhydrase%20(EC%204.2.1.1)&subsystem_name=Zinc_regulated_enzymes) |
| Regulation and Cell signaling | Regulation and Cell signaling - no subcategory | [Zinc regulated enzymes](https://rast.nmpdr.org/seedviewer.cgi?page=Subsystems&subsystem=Zinc_regulated_enzymes&organism=549.408) | [Putative metal chaperone, involved in Zn homeostasis, GTPase of COG0523 family](https://rast.nmpdr.org/seedviewer.cgi?page=FunctionalRole&role=Putative%20metal%20chaperone,%20involved%20in%20Zn%20homeostasis,%20GTPase%20of%20COG0523%20family&subsystem_name=Zinc_regulated_enzymes) |
| Regulation and Cell signaling | Regulation and Cell signaling - no subcategory | [Zinc regulated enzymes](https://rast.nmpdr.org/seedviewer.cgi?page=Subsystems&subsystem=Zinc_regulated_enzymes&organism=549.408) | [N-acetylmuramoyl-L-alanine amidase (EC 3.5.1.28)](https://rast.nmpdr.org/seedviewer.cgi?page=FunctionalRole&role=N-acetylmuramoyl-L-alanine%20amidase%20(EC%203.5.1.28)&subsystem_name=Zinc_regulated_enzymes) |
| Regulation and Cell signaling | Regulation and Cell signaling - no subcategory | [Zinc regulated enzymes](https://rast.nmpdr.org/seedviewer.cgi?page=Subsystems&subsystem=Zinc_regulated_enzymes&organism=549.408) | [C4-type zinc finger protein, DksA/TraR family](https://rast.nmpdr.org/seedviewer.cgi?page=FunctionalRole&role=C4-type%20zinc%20finger%20protein,%20DksA/TraR%20family&subsystem_name=Zinc_regulated_enzymes) |
| Regulation and Cell signaling | Regulation and Cell signaling - no subcategory | [Zinc regulated enzymes](https://rast.nmpdr.org/seedviewer.cgi?page=Subsystems&subsystem=Zinc_regulated_enzymes&organism=549.408) | [Porphobilinogen synthase (EC 4.2.1.24)](https://rast.nmpdr.org/seedviewer.cgi?page=FunctionalRole&role=Porphobilinogen%20synthase%20(EC%204.2.1.24)&subsystem_name=Zinc_regulated_enzymes) |
| Regulation and Cell signaling | Regulation and Cell signaling - no subcategory | [Zinc regulated enzymes](https://rast.nmpdr.org/seedviewer.cgi?page=Subsystems&subsystem=Zinc_regulated_enzymes&organism=549.408) | [Zinc uptake regulation protein ZUR](https://rast.nmpdr.org/seedviewer.cgi?page=FunctionalRole&role=Zinc%20uptake%20regulation%20protein%20ZUR&subsystem_name=Zinc_regulated_enzymes) |
| Regulation and Cell signaling | Regulation and Cell signaling - no subcategory | [Zinc regulated enzymes](https://rast.nmpdr.org/seedviewer.cgi?page=Subsystems&subsystem=Zinc_regulated_enzymes&organism=549.408) | [Cysteinyl-tRNA synthetase (EC 6.1.1.16)](https://rast.nmpdr.org/seedviewer.cgi?page=FunctionalRole&role=Cysteinyl-tRNA%20synthetase%20(EC%206.1.1.16)&subsystem_name=Zinc_regulated_enzymes) |
| Regulation and Cell signaling | Regulation and Cell signaling - no subcategory | [Zinc regulated enzymes](https://rast.nmpdr.org/seedviewer.cgi?page=Subsystems&subsystem=Zinc_regulated_enzymes&organism=549.408) | [GTP cyclohydrolase I (EC 3.5.4.16) type 1](https://rast.nmpdr.org/seedviewer.cgi?page=FunctionalRole&role=GTP%20cyclohydrolase%20I%20(EC%203.5.4.16)%20type%201&subsystem_name=Zinc_regulated_enzymes) |
| Regulation and Cell signaling | Regulation and Cell signaling - no subcategory | [Zinc regulated enzymes](https://rast.nmpdr.org/seedviewer.cgi?page=Subsystems&subsystem=Zinc_regulated_enzymes&organism=549.408) | [Phosphoribosyl-AMP cyclohydrolase (EC 3.5.4.19)](https://rast.nmpdr.org/seedviewer.cgi?page=FunctionalRole&role=Phosphoribosyl-AMP%20cyclohydrolase%20(EC%203.5.4.19)&subsystem_name=Zinc_regulated_enzymes) |
| Regulation and Cell signaling | Regulation and Cell signaling - no subcategory | [Zinc regulated enzymes](https://rast.nmpdr.org/seedviewer.cgi?page=Subsystems&subsystem=Zinc_regulated_enzymes&organism=549.408) | [Dihydroorotase (EC 3.5.2.3)](https://rast.nmpdr.org/seedviewer.cgi?page=FunctionalRole&role=Dihydroorotase%20(EC%203.5.2.3)&subsystem_name=Zinc_regulated_enzymes) |
| Regulation and Cell signaling | Regulation of virulence | [BarA-UvrY(SirA) two-component regulatory system](https://rast.nmpdr.org/seedviewer.cgi?page=Subsystems&subsystem=BarA-UvrY(SirA)_two-component_regulatory_system&organism=549.408) | [Excinuclease ABC subunit C](https://rast.nmpdr.org/seedviewer.cgi?page=FunctionalRole&role=Excinuclease%20ABC%20subunit%20C&subsystem_name=BarA-UvrY(SirA)_two-component_regulatory_system) |
| Regulation and Cell signaling | Regulation of virulence | [BarA-UvrY(SirA) two-component regulatory system](https://rast.nmpdr.org/seedviewer.cgi?page=Subsystems&subsystem=BarA-UvrY(SirA)_two-component_regulatory_system&organism=549.408) | [CDP-diacylglycerol--glycerol-3-phosphate 3-phosphatidyltransferase (EC 2.7.8.5)](https://rast.nmpdr.org/seedviewer.cgi?page=FunctionalRole&role=CDP-diacylglycerol--glycerol-3-phosphate%203-phosphatidyltransferase%20(EC%202.7.8.5)&subsystem_name=BarA-UvrY(SirA)_two-component_regulatory_system) |
| Regulation and Cell signaling | Regulation of virulence | [BarA-UvrY(SirA) two-component regulatory system](https://rast.nmpdr.org/seedviewer.cgi?page=Subsystems&subsystem=BarA-UvrY(SirA)_two-component_regulatory_system&organism=549.408) | [BarA sensory histidine kinase (= VarS = GacS)](https://rast.nmpdr.org/seedviewer.cgi?page=FunctionalRole&role=BarA%20sensory%20histidine%20kinase%20(=%20VarS%20=%20GacS)&subsystem_name=BarA-UvrY(SirA)_two-component_regulatory_system) |
| Regulation and Cell signaling | Regulation of virulence | [BarA-UvrY(SirA) two-component regulatory system](https://rast.nmpdr.org/seedviewer.cgi?page=Subsystems&subsystem=BarA-UvrY(SirA)_two-component_regulatory_system&organism=549.408) | [23S rRNA (Uracil-5-) -methyltransferase RumA (EC 2.1.1.-)](https://rast.nmpdr.org/seedviewer.cgi?page=FunctionalRole&role=23S%20rRNA%20(Uracil-5-)%20-methyltransferase%20RumA%20(EC%202.1.1.-)&subsystem_name=BarA-UvrY(SirA)_two-component_regulatory_system) |
| Regulation and Cell signaling | Regulation of virulence | [BarA-UvrY(SirA) two-component regulatory system](https://rast.nmpdr.org/seedviewer.cgi?page=Subsystems&subsystem=BarA-UvrY(SirA)_two-component_regulatory_system&organism=549.408) | [GTP pyrophosphokinase (EC 2.7.6.5), (p)ppGpp synthetase I](https://rast.nmpdr.org/seedviewer.cgi?page=FunctionalRole&role=GTP%20pyrophosphokinase%20(EC%202.7.6.5),%20(p)ppGpp%20synthetase%20I&subsystem_name=BarA-UvrY(SirA)_two-component_regulatory_system) |
| Regulation and Cell signaling | Regulation of virulence | [BarA-UvrY(SirA) two-component regulatory system](https://rast.nmpdr.org/seedviewer.cgi?page=Subsystems&subsystem=BarA-UvrY(SirA)_two-component_regulatory_system&organism=549.408) | [BarA-associated response regulator UvrY (= GacA = SirA)](https://rast.nmpdr.org/seedviewer.cgi?page=FunctionalRole&role=BarA-associated%20response%20regulator%20UvrY%20(=%20GacA%20=%20SirA)&subsystem_name=BarA-UvrY(SirA)_two-component_regulatory_system) |
| Regulation and Cell signaling | Regulation of virulence | [A conserved operon linked to TyrR and possibly involved in virulence](https://rast.nmpdr.org/seedviewer.cgi?page=Subsystems&subsystem=A_conserved_operon_linked_to_TyrR_and_possibly_involved_in_virulence&organism=549.408) | [Membrane protein YcjF](https://rast.nmpdr.org/seedviewer.cgi?page=FunctionalRole&role=Membrane%20protein%20YcjF&subsystem_name=A_conserved_operon_linked_to_TyrR_and_possibly_involved_in_virulence) |
| Regulation and Cell signaling | Regulation of virulence | [A conserved operon linked to TyrR and possibly involved in virulence](https://rast.nmpdr.org/seedviewer.cgi?page=Subsystems&subsystem=A_conserved_operon_linked_to_TyrR_and_possibly_involved_in_virulence&organism=549.408) | [Conserved protein YcjX with nucleoside triphosphate hydrolase domain](https://rast.nmpdr.org/seedviewer.cgi?page=FunctionalRole&role=Conserved%20protein%20YcjX%20with%20nucleoside%20triphosphate%20hydrolase%20domain&subsystem_name=A_conserved_operon_linked_to_TyrR_and_possibly_involved_in_virulence) |
| Regulation and Cell signaling | Programmed Cell Death and Toxin-antitoxin Systems | [Murein hydrolase regulation and cell death](https://rast.nmpdr.org/seedviewer.cgi?page=Subsystems&subsystem=Murein_hydrolase_regulation_and_cell_death&organism=549.408) | [LrgA-associated membrane protein LrgB](https://rast.nmpdr.org/seedviewer.cgi?page=FunctionalRole&role=LrgA-associated%20membrane%20protein%20LrgB&subsystem_name=Murein_hydrolase_regulation_and_cell_death) |
| Regulation and Cell signaling | Programmed Cell Death and Toxin-antitoxin Systems | [Murein hydrolase regulation and cell death](https://rast.nmpdr.org/seedviewer.cgi?page=Subsystems&subsystem=Murein_hydrolase_regulation_and_cell_death&organism=549.408) | [Autolysis histidine kinase LytS](https://rast.nmpdr.org/seedviewer.cgi?page=FunctionalRole&role=Autolysis%20histidine%20kinase%20LytS&subsystem_name=Murein_hydrolase_regulation_and_cell_death) |
| Regulation and Cell signaling | Programmed Cell Death and Toxin-antitoxin Systems | [Murein hydrolase regulation and cell death](https://rast.nmpdr.org/seedviewer.cgi?page=Subsystems&subsystem=Murein_hydrolase_regulation_and_cell_death&organism=549.408) | [Preprotein translocase subunit SecG (TC 3.A.5.1.1)](https://rast.nmpdr.org/seedviewer.cgi?page=FunctionalRole&role=Preprotein%20translocase%20subunit%20SecG%20(TC%203.A.5.1.1)&subsystem_name=Murein_hydrolase_regulation_and_cell_death) |
| Regulation and Cell signaling | Programmed Cell Death and Toxin-antitoxin Systems | [Murein hydrolase regulation and cell death](https://rast.nmpdr.org/seedviewer.cgi?page=Subsystems&subsystem=Murein_hydrolase_regulation_and_cell_death&organism=549.408) | [LysR family regulatory protein CidR](https://rast.nmpdr.org/seedviewer.cgi?page=FunctionalRole&role=LysR%20family%20regulatory%20protein%20CidR&subsystem_name=Murein_hydrolase_regulation_and_cell_death) |
| Regulation and Cell signaling | Programmed Cell Death and Toxin-antitoxin Systems | [Murein hydrolase regulation and cell death](https://rast.nmpdr.org/seedviewer.cgi?page=Subsystems&subsystem=Murein_hydrolase_regulation_and_cell_death&organism=549.408) | [Antiholin-like protein LrgA](https://rast.nmpdr.org/seedviewer.cgi?page=FunctionalRole&role=Antiholin-like%20protein%20LrgA&subsystem_name=Murein_hydrolase_regulation_and_cell_death) |
| Regulation and Cell signaling | Programmed Cell Death and Toxin-antitoxin Systems | [Murein hydrolase regulation and cell death](https://rast.nmpdr.org/seedviewer.cgi?page=Subsystems&subsystem=Murein_hydrolase_regulation_and_cell_death&organism=549.408) | [CidA-associated membrane protein CidB](https://rast.nmpdr.org/seedviewer.cgi?page=FunctionalRole&role=CidA-associated%20membrane%20protein%20CidB&subsystem_name=Murein_hydrolase_regulation_and_cell_death) |
| Regulation and Cell signaling | Programmed Cell Death and Toxin-antitoxin Systems | [Murein hydrolase regulation and cell death](https://rast.nmpdr.org/seedviewer.cgi?page=Subsystems&subsystem=Murein_hydrolase_regulation_and_cell_death&organism=549.408) | [Cytidine deaminase (EC 3.5.4.5)](https://rast.nmpdr.org/seedviewer.cgi?page=FunctionalRole&role=Cytidine%20deaminase%20(EC%203.5.4.5)&subsystem_name=Murein_hydrolase_regulation_and_cell_death) |
| Regulation and Cell signaling | Programmed Cell Death and Toxin-antitoxin Systems | [Murein hydrolase regulation and cell death](https://rast.nmpdr.org/seedviewer.cgi?page=Subsystems&subsystem=Murein_hydrolase_regulation_and_cell_death&organism=549.408) | [tRNA-dihydrouridine synthase C (EC 1.-.-.-)](https://rast.nmpdr.org/seedviewer.cgi?page=FunctionalRole&role=tRNA-dihydrouridine%20synthase%20C%20(EC%201.-.-.-)&subsystem_name=Murein_hydrolase_regulation_and_cell_death) |
| Regulation and Cell signaling | Programmed Cell Death and Toxin-antitoxin Systems | [Murein hydrolase regulation and cell death](https://rast.nmpdr.org/seedviewer.cgi?page=Subsystems&subsystem=Murein_hydrolase_regulation_and_cell_death&organism=549.408) | [Holin-like protein CidA](https://rast.nmpdr.org/seedviewer.cgi?page=FunctionalRole&role=Holin-like%20protein%20CidA&subsystem_name=Murein_hydrolase_regulation_and_cell_death) |
| Regulation and Cell signaling | Programmed Cell Death and Toxin-antitoxin Systems | [Toxin-antitoxin replicon stabilization systems](https://rast.nmpdr.org/seedviewer.cgi?page=Subsystems&subsystem=Toxin-antitoxin_replicon_stabilization_systems&organism=549.408) | [RelB/StbD replicon stabilization protein (antitoxin to RelE/StbE)](https://rast.nmpdr.org/seedviewer.cgi?page=FunctionalRole&role=RelB/StbD%20replicon%20stabilization%20protein%20(antitoxin%20to%20RelE/StbE)&subsystem_name=Toxin-antitoxin_replicon_stabilization_systems) |
| Regulation and Cell signaling | Programmed Cell Death and Toxin-antitoxin Systems | [Toxin-antitoxin replicon stabilization systems](https://rast.nmpdr.org/seedviewer.cgi?page=Subsystems&subsystem=Toxin-antitoxin_replicon_stabilization_systems&organism=549.408) | [DNA-damage-inducible protein J](https://rast.nmpdr.org/seedviewer.cgi?page=FunctionalRole&role=DNA-damage-inducible%20protein%20J&subsystem_name=Toxin-antitoxin_replicon_stabilization_systems) |
| Regulation and Cell signaling | Programmed Cell Death and Toxin-antitoxin Systems | [Toxin-antitoxin replicon stabilization systems](https://rast.nmpdr.org/seedviewer.cgi?page=Subsystems&subsystem=Toxin-antitoxin_replicon_stabilization_systems&organism=549.408) | [RelE/StbE replicon stabilization toxin](https://rast.nmpdr.org/seedviewer.cgi?page=FunctionalRole&role=RelE/StbE%20replicon%20stabilization%20toxin&subsystem_name=Toxin-antitoxin_replicon_stabilization_systems) |
| Regulation and Cell signaling | Programmed Cell Death and Toxin-antitoxin Systems | [Toxin-antitoxin replicon stabilization systems](https://rast.nmpdr.org/seedviewer.cgi?page=Subsystems&subsystem=Toxin-antitoxin_replicon_stabilization_systems&organism=549.408) | [HigA protein (antitoxin to HigB)](https://rast.nmpdr.org/seedviewer.cgi?page=FunctionalRole&role=HigA%20protein%20(antitoxin%20to%20HigB)&subsystem_name=Toxin-antitoxin_replicon_stabilization_systems) |
| Secondary Metabolism | Plant Hormones | [Auxin biosynthesis](https://rast.nmpdr.org/seedviewer.cgi?page=Subsystems&subsystem=Auxin_biosynthesis&organism=549.408) | [Tryptophan synthase alpha chain (EC 4.2.1.20)](https://rast.nmpdr.org/seedviewer.cgi?page=FunctionalRole&role=Tryptophan%20synthase%20alpha%20chain%20(EC%204.2.1.20)&subsystem_name=Auxin_biosynthesis) |
| Secondary Metabolism | Plant Hormones | [Auxin biosynthesis](https://rast.nmpdr.org/seedviewer.cgi?page=Subsystems&subsystem=Auxin_biosynthesis&organism=549.408) | [Anthranilate phosphoribosyltransferase (EC 2.4.2.18)](https://rast.nmpdr.org/seedviewer.cgi?page=FunctionalRole&role=Anthranilate%20phosphoribosyltransferase%20(EC%202.4.2.18)&subsystem_name=Auxin_biosynthesis) |
| Secondary Metabolism | Plant Hormones | [Auxin biosynthesis](https://rast.nmpdr.org/seedviewer.cgi?page=Subsystems&subsystem=Auxin_biosynthesis&organism=549.408) | [Tryptophan synthase beta chain (EC 4.2.1.20)](https://rast.nmpdr.org/seedviewer.cgi?page=FunctionalRole&role=Tryptophan%20synthase%20beta%20chain%20(EC%204.2.1.20)&subsystem_name=Auxin_biosynthesis) |
| Secondary Metabolism | Plant Hormones | [Auxin biosynthesis](https://rast.nmpdr.org/seedviewer.cgi?page=Subsystems&subsystem=Auxin_biosynthesis&organism=549.408) | [Phosphoribosylanthranilate isomerase (EC 5.3.1.24)](https://rast.nmpdr.org/seedviewer.cgi?page=FunctionalRole&role=Phosphoribosylanthranilate%20isomerase%20(EC%205.3.1.24)&subsystem_name=Auxin_biosynthesis) |
| DNA Metabolism | DNA repair | [Uracil-DNA glycosylase](https://rast.nmpdr.org/seedviewer.cgi?page=Subsystems&subsystem=Uracil-DNA_glycosylase&organism=549.408) | [Uracil-DNA glycosylase, family 1](https://rast.nmpdr.org/seedviewer.cgi?page=FunctionalRole&role=Uracil-DNA%20glycosylase,%20family%201&subsystem_name=Uracil-DNA_glycosylase) |
| DNA Metabolism | DNA repair | [Uracil-DNA glycosylase](https://rast.nmpdr.org/seedviewer.cgi?page=Subsystems&subsystem=Uracil-DNA_glycosylase&organism=549.408) | [G:T/U mismatch-specific uracil/thymine DNA-glycosylase](https://rast.nmpdr.org/seedviewer.cgi?page=FunctionalRole&role=G:T/U%20mismatch-specific%20uracil/thymine%20DNA-glycosylase&subsystem_name=Uracil-DNA_glycosylase) |
| DNA Metabolism | DNA repair | [DNA repair, bacterial MutL-MutS system](https://rast.nmpdr.org/seedviewer.cgi?page=Subsystems&subsystem=DNA_repair,_bacterial_MutL-MutS_system&organism=549.408) | [DNA mismatch repair protein MutS](https://rast.nmpdr.org/seedviewer.cgi?page=FunctionalRole&role=DNA%20mismatch%20repair%20protein%20MutS&subsystem_name=DNA_repair,_bacterial_MutL-MutS_system) |
| DNA Metabolism | DNA repair | [DNA repair, bacterial MutL-MutS system](https://rast.nmpdr.org/seedviewer.cgi?page=Subsystems&subsystem=DNA_repair,_bacterial_MutL-MutS_system&organism=549.408) | [DNA mismatch repair protein MutL](https://rast.nmpdr.org/seedviewer.cgi?page=FunctionalRole&role=DNA%20mismatch%20repair%20protein%20MutL&subsystem_name=DNA_repair,_bacterial_MutL-MutS_system) |
| DNA Metabolism | DNA repair | [DNA repair, UvrABC system](https://rast.nmpdr.org/seedviewer.cgi?page=Subsystems&subsystem=DNA_repair,_UvrABC_system&organism=549.408) | [Excinuclease ABC subunit C](https://rast.nmpdr.org/seedviewer.cgi?page=FunctionalRole&role=Excinuclease%20ABC%20subunit%20C&subsystem_name=DNA_repair,_UvrABC_system) |
| DNA Metabolism | DNA repair | [DNA repair, UvrABC system](https://rast.nmpdr.org/seedviewer.cgi?page=Subsystems&subsystem=DNA_repair,_UvrABC_system&organism=549.408) | [Excinuclease ABC subunit A paralog of unknown function](https://rast.nmpdr.org/seedviewer.cgi?page=FunctionalRole&role=Excinuclease%20ABC%20subunit%20A%20paralog%20of%20unknown%20function&subsystem_name=DNA_repair,_UvrABC_system) |
| DNA Metabolism | DNA repair | [DNA repair, UvrABC system](https://rast.nmpdr.org/seedviewer.cgi?page=Subsystems&subsystem=DNA_repair,_UvrABC_system&organism=549.408) | [Excinuclease cho (excinuclease ABC alternative C subunit)](https://rast.nmpdr.org/seedviewer.cgi?page=FunctionalRole&role=Excinuclease%20cho%20(excinuclease%20ABC%20alternative%20C%20subunit)&subsystem_name=DNA_repair,_UvrABC_system) |
| DNA Metabolism | DNA repair | [DNA repair, UvrABC system](https://rast.nmpdr.org/seedviewer.cgi?page=Subsystems&subsystem=DNA_repair,_UvrABC_system&organism=549.408) | [Excinuclease ABC subunit A](https://rast.nmpdr.org/seedviewer.cgi?page=FunctionalRole&role=Excinuclease%20ABC%20subunit%20A&subsystem_name=DNA_repair,_UvrABC_system) |
| DNA Metabolism | DNA repair | [DNA repair, UvrABC system](https://rast.nmpdr.org/seedviewer.cgi?page=Subsystems&subsystem=DNA_repair,_UvrABC_system&organism=549.408) | [Excinuclease ABC subunit B](https://rast.nmpdr.org/seedviewer.cgi?page=FunctionalRole&role=Excinuclease%20ABC%20subunit%20B&subsystem_name=DNA_repair,_UvrABC_system) |
| DNA Metabolism | DNA repair | [DNA repair, bacterial photolyase](https://rast.nmpdr.org/seedviewer.cgi?page=Subsystems&subsystem=DNA_repair,_bacterial_photolyase&organism=549.408) | [Deoxyribodipyrimidine photolyase (EC 4.1.99.3)](https://rast.nmpdr.org/seedviewer.cgi?page=FunctionalRole&role=Deoxyribodipyrimidine%20photolyase%20(EC%204.1.99.3)&subsystem_name=DNA_repair,_bacterial_photolyase) |
| DNA Metabolism | DNA repair | [DNA repair, bacterial DinG and relatives](https://rast.nmpdr.org/seedviewer.cgi?page=Subsystems&subsystem=DNA_repair,_bacterial_DinG_and_relatives&organism=549.408) | [DinG family ATP-dependent helicase YoaA](https://rast.nmpdr.org/seedviewer.cgi?page=FunctionalRole&role=DinG%20family%20ATP-dependent%20helicase%20YoaA&subsystem_name=DNA_repair,_bacterial_DinG_and_relatives) |
| DNA Metabolism | DNA repair | [DNA repair, bacterial DinG and relatives](https://rast.nmpdr.org/seedviewer.cgi?page=Subsystems&subsystem=DNA_repair,_bacterial_DinG_and_relatives&organism=549.408) | [ATP-dependent helicase DinG/Rad3](https://rast.nmpdr.org/seedviewer.cgi?page=FunctionalRole&role=ATP-dependent%20helicase%20DinG/Rad3&subsystem_name=DNA_repair,_bacterial_DinG_and_relatives) |
| DNA Metabolism | DNA repair | [2-phosphoglycolate salvage](https://rast.nmpdr.org/seedviewer.cgi?page=Subsystems&subsystem=2-phosphoglycolate_salvage&organism=549.408) | [Putative phosphatase YieH](https://rast.nmpdr.org/seedviewer.cgi?page=FunctionalRole&role=Putative%20phosphatase%20YieH&subsystem_name=2-phosphoglycolate_salvage) |
| DNA Metabolism | DNA repair | [2-phosphoglycolate salvage](https://rast.nmpdr.org/seedviewer.cgi?page=Subsystems&subsystem=2-phosphoglycolate_salvage&organism=549.408) | [Phosphoglycolate phosphatase (EC 3.1.3.18)](https://rast.nmpdr.org/seedviewer.cgi?page=FunctionalRole&role=Phosphoglycolate%20phosphatase%20(EC%203.1.3.18)&subsystem_name=2-phosphoglycolate_salvage) |
| DNA Metabolism | DNA repair | [2-phosphoglycolate salvage](https://rast.nmpdr.org/seedviewer.cgi?page=Subsystems&subsystem=2-phosphoglycolate_salvage&organism=549.408) | [Putative phosphatase YqaB](https://rast.nmpdr.org/seedviewer.cgi?page=FunctionalRole&role=Putative%20phosphatase%20YqaB&subsystem_name=2-phosphoglycolate_salvage) |
| DNA Metabolism | DNA repair | [2-phosphoglycolate salvage](https://rast.nmpdr.org/seedviewer.cgi?page=Subsystems&subsystem=2-phosphoglycolate_salvage&organism=549.408) | [Putative phosphatase YfbT](https://rast.nmpdr.org/seedviewer.cgi?page=FunctionalRole&role=Putative%20phosphatase%20YfbT&subsystem_name=2-phosphoglycolate_salvage) |
| DNA Metabolism | DNA repair | [2-phosphoglycolate salvage](https://rast.nmpdr.org/seedviewer.cgi?page=Subsystems&subsystem=2-phosphoglycolate_salvage&organism=549.408) | [2-deoxyglucose-6-phosphate hydrolase YniC](https://rast.nmpdr.org/seedviewer.cgi?page=FunctionalRole&role=2-deoxyglucose-6-phosphate%20hydrolase%20YniC&subsystem_name=2-phosphoglycolate_salvage) |
| DNA Metabolism | DNA repair | [RecA and RecX](https://rast.nmpdr.org/seedviewer.cgi?page=Subsystems&subsystem=RecA_and_RecX&organism=549.408) | [RecA protein](https://rast.nmpdr.org/seedviewer.cgi?page=FunctionalRole&role=RecA%20protein&subsystem_name=RecA_and_RecX) |
| DNA Metabolism | DNA repair | [RecA and RecX](https://rast.nmpdr.org/seedviewer.cgi?page=Subsystems&subsystem=RecA_and_RecX&organism=549.408) | [Regulatory protein RecX](https://rast.nmpdr.org/seedviewer.cgi?page=FunctionalRole&role=Regulatory%20protein%20RecX&subsystem_name=RecA_and_RecX) |
| DNA Metabolism | DNA repair | [DNA repair system including RecA, MutS and a hypothetical protein](https://rast.nmpdr.org/seedviewer.cgi?page=Subsystems&subsystem=DNA_repair_system_including_RecA,_MutS_and_a_hypothetical_protein&organism=549.408) | [DNA mismatch repair protein MutS](https://rast.nmpdr.org/seedviewer.cgi?page=FunctionalRole&role=DNA%20mismatch%20repair%20protein%20MutS&subsystem_name=DNA_repair_system_including_RecA,_MutS_and_a_hypothetical_protein) |
| DNA Metabolism | DNA repair | [DNA repair system including RecA, MutS and a hypothetical protein](https://rast.nmpdr.org/seedviewer.cgi?page=Subsystems&subsystem=DNA_repair_system_including_RecA,_MutS_and_a_hypothetical_protein&organism=549.408) | [RecA protein](https://rast.nmpdr.org/seedviewer.cgi?page=FunctionalRole&role=RecA%20protein&subsystem_name=DNA_repair_system_including_RecA,_MutS_and_a_hypothetical_protein) |
| DNA Metabolism | DNA repair | [DNA repair system including RecA, MutS and a hypothetical protein](https://rast.nmpdr.org/seedviewer.cgi?page=Subsystems&subsystem=DNA_repair_system_including_RecA,_MutS_and_a_hypothetical_protein&organism=549.408) | [Regulatory protein RecX](https://rast.nmpdr.org/seedviewer.cgi?page=FunctionalRole&role=Regulatory%20protein%20RecX&subsystem_name=DNA_repair_system_including_RecA,_MutS_and_a_hypothetical_protein) |
| DNA Metabolism | DNA repair | [DNA repair, bacterial](https://rast.nmpdr.org/seedviewer.cgi?page=Subsystems&subsystem=DNA_repair,_bacterial&organism=549.408) | [DNA mismatch repair endonuclease MutH](https://rast.nmpdr.org/seedviewer.cgi?page=FunctionalRole&role=DNA%20mismatch%20repair%20endonuclease%20MutH&subsystem_name=DNA_repair,_bacterial) |
| DNA Metabolism | DNA repair | [DNA repair, bacterial](https://rast.nmpdr.org/seedviewer.cgi?page=Subsystems&subsystem=DNA_repair,_bacterial&organism=549.408) | [Exonuclease SbcD](https://rast.nmpdr.org/seedviewer.cgi?page=FunctionalRole&role=Exonuclease%20SbcD&subsystem_name=DNA_repair,_bacterial) |
| DNA Metabolism | DNA repair | [DNA repair, bacterial](https://rast.nmpdr.org/seedviewer.cgi?page=Subsystems&subsystem=DNA_repair,_bacterial&organism=549.408) | [ADA regulatory protein](https://rast.nmpdr.org/seedviewer.cgi?page=FunctionalRole&role=ADA%20regulatory%20protein&subsystem_name=DNA_repair,_bacterial) |
| DNA Metabolism | DNA repair | [DNA repair, bacterial](https://rast.nmpdr.org/seedviewer.cgi?page=Subsystems&subsystem=DNA_repair,_bacterial&organism=549.408) | [SOS-response repressor and protease LexA (EC 3.4.21.88)](https://rast.nmpdr.org/seedviewer.cgi?page=FunctionalRole&role=SOS-response%20repressor%20and%20protease%20LexA%20(EC%203.4.21.88)&subsystem_name=DNA_repair,_bacterial) |
| DNA Metabolism | DNA repair | [DNA repair, bacterial](https://rast.nmpdr.org/seedviewer.cgi?page=Subsystems&subsystem=DNA_repair,_bacterial&organism=549.408) | [DNA repair protein RadC](https://rast.nmpdr.org/seedviewer.cgi?page=FunctionalRole&role=DNA%20repair%20protein%20RadC&subsystem_name=DNA_repair,_bacterial) |
| DNA Metabolism | DNA repair | [DNA repair, bacterial](https://rast.nmpdr.org/seedviewer.cgi?page=Subsystems&subsystem=DNA_repair,_bacterial&organism=549.408) | [DNA-cytosine methyltransferase (EC 2.1.1.37)](https://rast.nmpdr.org/seedviewer.cgi?page=FunctionalRole&role=DNA-cytosine%20methyltransferase%20(EC%202.1.1.37)&subsystem_name=DNA_repair,_bacterial) |
| DNA Metabolism | DNA repair | [DNA repair, bacterial](https://rast.nmpdr.org/seedviewer.cgi?page=Subsystems&subsystem=DNA_repair,_bacterial&organism=549.408) | [DNA polymerase IV (EC 2.7.7.7)](https://rast.nmpdr.org/seedviewer.cgi?page=FunctionalRole&role=DNA%20polymerase%20IV%20(EC%202.7.7.7)&subsystem_name=DNA_repair,_bacterial) |
| DNA Metabolism | DNA repair | [DNA repair, bacterial](https://rast.nmpdr.org/seedviewer.cgi?page=Subsystems&subsystem=DNA_repair,_bacterial&organism=549.408) | [Very-short-patch mismatch repair endonuclease (G-T specific)](https://rast.nmpdr.org/seedviewer.cgi?page=FunctionalRole&role=Very-short-patch%20mismatch%20repair%20endonuclease%20(G-T%20specific)&subsystem_name=DNA_repair,_bacterial) |
| DNA Metabolism | DNA repair | [DNA repair, bacterial](https://rast.nmpdr.org/seedviewer.cgi?page=Subsystems&subsystem=DNA_repair,_bacterial&organism=549.408) | [Exonuclease SbcC](https://rast.nmpdr.org/seedviewer.cgi?page=FunctionalRole&role=Exonuclease%20SbcC&subsystem_name=DNA_repair,_bacterial) |
| DNA Metabolism | DNA repair | [DNA repair, bacterial](https://rast.nmpdr.org/seedviewer.cgi?page=Subsystems&subsystem=DNA_repair,_bacterial&organism=549.408) | [Endonuclease V (EC 3.1.21.7)](https://rast.nmpdr.org/seedviewer.cgi?page=FunctionalRole&role=Endonuclease%20V%20(EC%203.1.21.7)&subsystem_name=DNA_repair,_bacterial) |
| DNA Metabolism | DNA repair | [DNA repair, bacterial](https://rast.nmpdr.org/seedviewer.cgi?page=Subsystems&subsystem=DNA_repair,_bacterial&organism=549.408) | [DNA-damage-inducible protein J](https://rast.nmpdr.org/seedviewer.cgi?page=FunctionalRole&role=DNA-damage-inducible%20protein%20J&subsystem_name=DNA_repair,_bacterial) |
| DNA Metabolism | DNA repair | [DNA repair, bacterial](https://rast.nmpdr.org/seedviewer.cgi?page=Subsystems&subsystem=DNA_repair,_bacterial&organism=549.408) | [Single-stranded DNA-binding protein](https://rast.nmpdr.org/seedviewer.cgi?page=FunctionalRole&role=Single-stranded%20DNA-binding%20protein&subsystem_name=DNA_repair,_bacterial) |
| DNA Metabolism | DNA repair | [DNA repair, bacterial](https://rast.nmpdr.org/seedviewer.cgi?page=Subsystems&subsystem=DNA_repair,_bacterial&organism=549.408) | [Alkylated DNA repair protein AlkB](https://rast.nmpdr.org/seedviewer.cgi?page=FunctionalRole&role=Alkylated%20DNA%20repair%20protein%20AlkB&subsystem_name=DNA_repair,_bacterial) |
| DNA Metabolism | DNA repair | [DNA repair, bacterial](https://rast.nmpdr.org/seedviewer.cgi?page=Subsystems&subsystem=DNA_repair,_bacterial&organism=549.408) | [Exodeoxyribonuclease VII small subunit (EC 3.1.11.6)](https://rast.nmpdr.org/seedviewer.cgi?page=FunctionalRole&role=Exodeoxyribonuclease%20VII%20small%20subunit%20(EC%203.1.11.6)&subsystem_name=DNA_repair,_bacterial) |
| DNA Metabolism | DNA repair | [DNA repair, bacterial](https://rast.nmpdr.org/seedviewer.cgi?page=Subsystems&subsystem=DNA_repair,_bacterial&organism=549.408) | [DNA repair protein RadA](https://rast.nmpdr.org/seedviewer.cgi?page=FunctionalRole&role=DNA%20repair%20protein%20RadA&subsystem_name=DNA_repair,_bacterial) |
| DNA Metabolism | DNA repair | [DNA repair, bacterial](https://rast.nmpdr.org/seedviewer.cgi?page=Subsystems&subsystem=DNA_repair,_bacterial&organism=549.408) | [Exodeoxyribonuclease VII large subunit (EC 3.1.11.6)](https://rast.nmpdr.org/seedviewer.cgi?page=FunctionalRole&role=Exodeoxyribonuclease%20VII%20large%20subunit%20(EC%203.1.11.6)&subsystem_name=DNA_repair,_bacterial) |
| DNA Metabolism | DNA repair | [DNA repair, bacterial](https://rast.nmpdr.org/seedviewer.cgi?page=Subsystems&subsystem=DNA_repair,_bacterial&organism=549.408) | [DNA repair protein RecN](https://rast.nmpdr.org/seedviewer.cgi?page=FunctionalRole&role=DNA%20repair%20protein%20RecN&subsystem_name=DNA_repair,_bacterial) |
| DNA Metabolism | DNA repair | [DNA repair, bacterial](https://rast.nmpdr.org/seedviewer.cgi?page=Subsystems&subsystem=DNA_repair,_bacterial&organism=549.408) | [DNA-damage-inducible protein I](https://rast.nmpdr.org/seedviewer.cgi?page=FunctionalRole&role=DNA-damage-inducible%20protein%20I&subsystem_name=DNA_repair,_bacterial) |
| DNA Metabolism | DNA repair | [DNA repair, bacterial](https://rast.nmpdr.org/seedviewer.cgi?page=Subsystems&subsystem=DNA_repair,_bacterial&organism=549.408) | [A/G-specific adenine glycosylase (EC 3.2.2.-)](https://rast.nmpdr.org/seedviewer.cgi?page=FunctionalRole&role=A/G-specific%20adenine%20glycosylase%20(EC%203.2.2.-)&subsystem_name=DNA_repair,_bacterial) |
| DNA Metabolism | DNA repair | [DNA repair, bacterial](https://rast.nmpdr.org/seedviewer.cgi?page=Subsystems&subsystem=DNA_repair,_bacterial&organism=549.408) | [DNA recombination protein RmuC](https://rast.nmpdr.org/seedviewer.cgi?page=FunctionalRole&role=DNA%20recombination%20protein%20RmuC&subsystem_name=DNA_repair,_bacterial) |
| DNA Metabolism | DNA repair | [DNA repair, bacterial](https://rast.nmpdr.org/seedviewer.cgi?page=Subsystems&subsystem=DNA_repair,_bacterial&organism=549.408) | [DNA recombination-dependent growth factor C](https://rast.nmpdr.org/seedviewer.cgi?page=FunctionalRole&role=DNA%20recombination-dependent%20growth%20factor%20C&subsystem_name=DNA_repair,_bacterial) |
| DNA Metabolism | DNA repair | [DNA repair, bacterial](https://rast.nmpdr.org/seedviewer.cgi?page=Subsystems&subsystem=DNA_repair,_bacterial&organism=549.408) | [Methylated-DNA--protein-cysteine methyltransferase (EC 2.1.1.63)](https://rast.nmpdr.org/seedviewer.cgi?page=FunctionalRole&role=Methylated-DNA--protein-cysteine%20methyltransferase%20(EC%202.1.1.63)&subsystem_name=DNA_repair,_bacterial) |
| DNA Metabolism | DNA repair | [DNA repair, bacterial](https://rast.nmpdr.org/seedviewer.cgi?page=Subsystems&subsystem=DNA_repair,_bacterial&organism=549.408) | [G:T/U mismatch-specific uracil/thymine DNA-glycosylase](https://rast.nmpdr.org/seedviewer.cgi?page=FunctionalRole&role=G:T/U%20mismatch-specific%20uracil/thymine%20DNA-glycosylase&subsystem_name=DNA_repair,_bacterial) |
| DNA Metabolism | DNA repair | [DNA repair, bacterial](https://rast.nmpdr.org/seedviewer.cgi?page=Subsystems&subsystem=DNA_repair,_bacterial&organism=549.408) | [Endonuclease IV (EC 3.1.21.2)](https://rast.nmpdr.org/seedviewer.cgi?page=FunctionalRole&role=Endonuclease%20IV%20(EC%203.1.21.2)&subsystem_name=DNA_repair,_bacterial) |
| DNA Metabolism | DNA repair | [DNA repair, bacterial](https://rast.nmpdr.org/seedviewer.cgi?page=Subsystems&subsystem=DNA_repair,_bacterial&organism=549.408) | [RecA protein](https://rast.nmpdr.org/seedviewer.cgi?page=FunctionalRole&role=RecA%20protein&subsystem_name=DNA_repair,_bacterial) |
| DNA Metabolism | DNA repair | [DNA repair, bacterial](https://rast.nmpdr.org/seedviewer.cgi?page=Subsystems&subsystem=DNA_repair,_bacterial&organism=549.408) | [Methyl-directed repair DNA adenine methylase (EC 2.1.1.72)](https://rast.nmpdr.org/seedviewer.cgi?page=FunctionalRole&role=Methyl-directed%20repair%20DNA%20adenine%20methylase%20(EC%202.1.1.72)&subsystem_name=DNA_repair,_bacterial) |
| DNA Metabolism | DNA repair | [DNA repair, bacterial](https://rast.nmpdr.org/seedviewer.cgi?page=Subsystems&subsystem=DNA_repair,_bacterial&organism=549.408) | [Exodeoxyribonuclease III (EC 3.1.11.2)](https://rast.nmpdr.org/seedviewer.cgi?page=FunctionalRole&role=Exodeoxyribonuclease%20III%20(EC%203.1.11.2)&subsystem_name=DNA_repair,_bacterial) |
| DNA Metabolism | DNA repair | [DNA repair, bacterial UmuCD system](https://rast.nmpdr.org/seedviewer.cgi?page=Subsystems&subsystem=DNA_repair,_bacterial_UmuCD_system&organism=549.408) | [SOS-response repressor and protease LexA (EC 3.4.21.88)](https://rast.nmpdr.org/seedviewer.cgi?page=FunctionalRole&role=SOS-response%20repressor%20and%20protease%20LexA%20(EC%203.4.21.88)&subsystem_name=DNA_repair,_bacterial_UmuCD_system) |
| DNA Metabolism | DNA repair | [DNA repair, bacterial UmuCD system](https://rast.nmpdr.org/seedviewer.cgi?page=Subsystems&subsystem=DNA_repair,_bacterial_UmuCD_system&organism=549.408) | [RecA protein](https://rast.nmpdr.org/seedviewer.cgi?page=FunctionalRole&role=RecA%20protein&subsystem_name=DNA_repair,_bacterial_UmuCD_system) |
| DNA Metabolism | DNA repair | [DNA repair, bacterial UmuCD system](https://rast.nmpdr.org/seedviewer.cgi?page=Subsystems&subsystem=DNA_repair,_bacterial_UmuCD_system&organism=549.408) | [Error-prone repair protein UmuD](https://rast.nmpdr.org/seedviewer.cgi?page=FunctionalRole&role=Error-prone%20repair%20protein%20UmuD&subsystem_name=DNA_repair,_bacterial_UmuCD_system) |
| DNA Metabolism | DNA repair | [DNA repair, bacterial UmuCD system](https://rast.nmpdr.org/seedviewer.cgi?page=Subsystems&subsystem=DNA_repair,_bacterial_UmuCD_system&organism=549.408) | [Error-prone, lesion bypass DNA polymerase V (UmuC)](https://rast.nmpdr.org/seedviewer.cgi?page=FunctionalRole&role=Error-prone,%20lesion%20bypass%20DNA%20polymerase%20V%20(UmuC)&subsystem_name=DNA_repair,_bacterial_UmuCD_system) |
| DNA Metabolism | DNA repair | [DNA repair, bacterial RecFOR pathway](https://rast.nmpdr.org/seedviewer.cgi?page=Subsystems&subsystem=DNA_repair,_bacterial_RecFOR_pathway&organism=549.408) | [ATP-dependent DNA helicase RecQ](https://rast.nmpdr.org/seedviewer.cgi?page=FunctionalRole&role=ATP-dependent%20DNA%20helicase%20RecQ&subsystem_name=DNA_repair,_bacterial_RecFOR_pathway) |
| DNA Metabolism | DNA repair | [DNA repair, bacterial RecFOR pathway](https://rast.nmpdr.org/seedviewer.cgi?page=Subsystems&subsystem=DNA_repair,_bacterial_RecFOR_pathway&organism=549.408) | [Single-stranded-DNA-specific exonuclease RecJ (EC 3.1.-.-)](https://rast.nmpdr.org/seedviewer.cgi?page=FunctionalRole&role=Single-stranded-DNA-specific%20exonuclease%20RecJ%20(EC%203.1.-.-)&subsystem_name=DNA_repair,_bacterial_RecFOR_pathway) |
| DNA Metabolism | DNA repair | [DNA repair, bacterial RecFOR pathway](https://rast.nmpdr.org/seedviewer.cgi?page=Subsystems&subsystem=DNA_repair,_bacterial_RecFOR_pathway&organism=549.408) | [Single-stranded DNA-binding protein](https://rast.nmpdr.org/seedviewer.cgi?page=FunctionalRole&role=Single-stranded%20DNA-binding%20protein&subsystem_name=DNA_repair,_bacterial_RecFOR_pathway) |
| DNA Metabolism | DNA repair | [DNA repair, bacterial RecFOR pathway](https://rast.nmpdr.org/seedviewer.cgi?page=Subsystems&subsystem=DNA_repair,_bacterial_RecFOR_pathway&organism=549.408) | [Recombination protein RecR](https://rast.nmpdr.org/seedviewer.cgi?page=FunctionalRole&role=Recombination%20protein%20RecR&subsystem_name=DNA_repair,_bacterial_RecFOR_pathway) |
| DNA Metabolism | DNA repair | [DNA repair, bacterial RecFOR pathway](https://rast.nmpdr.org/seedviewer.cgi?page=Subsystems&subsystem=DNA_repair,_bacterial_RecFOR_pathway&organism=549.408) | [RecA protein](https://rast.nmpdr.org/seedviewer.cgi?page=FunctionalRole&role=RecA%20protein&subsystem_name=DNA_repair,_bacterial_RecFOR_pathway) |
| DNA Metabolism | DNA repair | [DNA repair, bacterial RecFOR pathway](https://rast.nmpdr.org/seedviewer.cgi?page=Subsystems&subsystem=DNA_repair,_bacterial_RecFOR_pathway&organism=549.408) | [DNA recombination and repair protein RecO](https://rast.nmpdr.org/seedviewer.cgi?page=FunctionalRole&role=DNA%20recombination%20and%20repair%20protein%20RecO&subsystem_name=DNA_repair,_bacterial_RecFOR_pathway) |
| DNA Metabolism | DNA repair | [DNA repair, bacterial RecFOR pathway](https://rast.nmpdr.org/seedviewer.cgi?page=Subsystems&subsystem=DNA_repair,_bacterial_RecFOR_pathway&organism=549.408) | [DNA recombination and repair protein RecF](https://rast.nmpdr.org/seedviewer.cgi?page=FunctionalRole&role=DNA%20recombination%20and%20repair%20protein%20RecF&subsystem_name=DNA_repair,_bacterial_RecFOR_pathway) |
| DNA Metabolism | DNA repair | [DNA repair, bacterial RecBCD pathway](https://rast.nmpdr.org/seedviewer.cgi?page=Subsystems&subsystem=DNA_repair,_bacterial_RecBCD_pathway&organism=549.408) | [Exodeoxyribonuclease V beta chain (EC 3.1.11.5)](https://rast.nmpdr.org/seedviewer.cgi?page=FunctionalRole&role=Exodeoxyribonuclease%20V%20beta%20chain%20(EC%203.1.11.5)&subsystem_name=DNA_repair,_bacterial_RecBCD_pathway) |
| DNA Metabolism | DNA repair | [DNA repair, bacterial RecBCD pathway](https://rast.nmpdr.org/seedviewer.cgi?page=Subsystems&subsystem=DNA_repair,_bacterial_RecBCD_pathway&organism=549.408) | [Exodeoxyribonuclease V gamma chain (EC 3.1.11.5)](https://rast.nmpdr.org/seedviewer.cgi?page=FunctionalRole&role=Exodeoxyribonuclease%20V%20gamma%20chain%20(EC%203.1.11.5)&subsystem_name=DNA_repair,_bacterial_RecBCD_pathway) |
| DNA Metabolism | DNA repair | [DNA repair, bacterial RecBCD pathway](https://rast.nmpdr.org/seedviewer.cgi?page=Subsystems&subsystem=DNA_repair,_bacterial_RecBCD_pathway&organism=549.408) | [Protease III precursor (EC 3.4.24.55)](https://rast.nmpdr.org/seedviewer.cgi?page=FunctionalRole&role=Protease%20III%20precursor%20(EC%203.4.24.55)&subsystem_name=DNA_repair,_bacterial_RecBCD_pathway) |
| DNA Metabolism | DNA repair | [DNA repair, bacterial RecBCD pathway](https://rast.nmpdr.org/seedviewer.cgi?page=Subsystems&subsystem=DNA_repair,_bacterial_RecBCD_pathway&organism=549.408) | [Exodeoxyribonuclease V alpha chain (EC 3.1.11.5)](https://rast.nmpdr.org/seedviewer.cgi?page=FunctionalRole&role=Exodeoxyribonuclease%20V%20alpha%20chain%20(EC%203.1.11.5)&subsystem_name=DNA_repair,_bacterial_RecBCD_pathway) |
| DNA Metabolism | DNA repair | [DNA repair, bacterial UvrD and related helicases](https://rast.nmpdr.org/seedviewer.cgi?page=Subsystems&subsystem=DNA_repair,_bacterial_UvrD_and_related_helicases&organism=549.408) | [DNA helicase IV](https://rast.nmpdr.org/seedviewer.cgi?page=FunctionalRole&role=DNA%20helicase%20IV&subsystem_name=DNA_repair,_bacterial_UvrD_and_related_helicases) |
| DNA Metabolism | DNA repair | [DNA repair, bacterial UvrD and related helicases](https://rast.nmpdr.org/seedviewer.cgi?page=Subsystems&subsystem=DNA_repair,_bacterial_UvrD_and_related_helicases&organism=549.408) | [ATP-dependent DNA helicase Rep](https://rast.nmpdr.org/seedviewer.cgi?page=FunctionalRole&role=ATP-dependent%20DNA%20helicase%20Rep&subsystem_name=DNA_repair,_bacterial_UvrD_and_related_helicases) |
| DNA Metabolism | DNA repair | [DNA repair, bacterial UvrD and related helicases](https://rast.nmpdr.org/seedviewer.cgi?page=Subsystems&subsystem=DNA_repair,_bacterial_UvrD_and_related_helicases&organism=549.408) | [ATP-dependent DNA helicase UvrD/PcrA](https://rast.nmpdr.org/seedviewer.cgi?page=FunctionalRole&role=ATP-dependent%20DNA%20helicase%20UvrD/PcrA&subsystem_name=DNA_repair,_bacterial_UvrD_and_related_helicases) |
| DNA Metabolism | DNA repair | [DNA Repair Base Excision](https://rast.nmpdr.org/seedviewer.cgi?page=Subsystems&subsystem=DNA_Repair_Base_Excision&organism=549.408) | [DNA polymerase II (EC 2.7.7.7)](https://rast.nmpdr.org/seedviewer.cgi?page=FunctionalRole&role=DNA%20polymerase%20II%20(EC%202.7.7.7)&subsystem_name=DNA_Repair_Base_Excision) |
| DNA Metabolism | DNA repair | [DNA Repair Base Excision](https://rast.nmpdr.org/seedviewer.cgi?page=Subsystems&subsystem=DNA_Repair_Base_Excision&organism=549.408) | [Uracil-DNA glycosylase, family 1](https://rast.nmpdr.org/seedviewer.cgi?page=FunctionalRole&role=Uracil-DNA%20glycosylase,%20family%201&subsystem_name=DNA_Repair_Base_Excision) |
| DNA Metabolism | DNA repair | [DNA Repair Base Excision](https://rast.nmpdr.org/seedviewer.cgi?page=Subsystems&subsystem=DNA_Repair_Base_Excision&organism=549.408) | [DNA-3-methyladenine glycosylase (EC 3.2.2.20)](https://rast.nmpdr.org/seedviewer.cgi?page=FunctionalRole&role=DNA-3-methyladenine%20glycosylase%20(EC%203.2.2.20)&subsystem_name=DNA_Repair_Base_Excision) |
| DNA Metabolism | DNA repair | [DNA Repair Base Excision](https://rast.nmpdr.org/seedviewer.cgi?page=Subsystems&subsystem=DNA_Repair_Base_Excision&organism=549.408) | [Formamidopyrimidine-DNA glycosylase (EC 3.2.2.23)](https://rast.nmpdr.org/seedviewer.cgi?page=FunctionalRole&role=Formamidopyrimidine-DNA%20glycosylase%20(EC%203.2.2.23)&subsystem_name=DNA_Repair_Base_Excision) |
| DNA Metabolism | DNA repair | [DNA Repair Base Excision](https://rast.nmpdr.org/seedviewer.cgi?page=Subsystems&subsystem=DNA_Repair_Base_Excision&organism=549.408) | [Endonuclease VIII](https://rast.nmpdr.org/seedviewer.cgi?page=FunctionalRole&role=Endonuclease%20VIII&subsystem_name=DNA_Repair_Base_Excision) |
| DNA Metabolism | DNA repair | [DNA Repair Base Excision](https://rast.nmpdr.org/seedviewer.cgi?page=Subsystems&subsystem=DNA_Repair_Base_Excision&organism=549.408) | [Endonuclease III (EC 4.2.99.18)](https://rast.nmpdr.org/seedviewer.cgi?page=FunctionalRole&role=Endonuclease%20III%20(EC%204.2.99.18)&subsystem_name=DNA_Repair_Base_Excision) |
| DNA Metabolism | DNA repair | [DNA Repair Base Excision](https://rast.nmpdr.org/seedviewer.cgi?page=Subsystems&subsystem=DNA_Repair_Base_Excision&organism=549.408) | [Single-stranded-DNA-specific exonuclease RecJ (EC 3.1.-.-)](https://rast.nmpdr.org/seedviewer.cgi?page=FunctionalRole&role=Single-stranded-DNA-specific%20exonuclease%20RecJ%20(EC%203.1.-.-)&subsystem_name=DNA_Repair_Base_Excision) |
| DNA Metabolism | DNA repair | [DNA Repair Base Excision](https://rast.nmpdr.org/seedviewer.cgi?page=Subsystems&subsystem=DNA_Repair_Base_Excision&organism=549.408) | [DNA ligase (EC 6.5.1.2)](https://rast.nmpdr.org/seedviewer.cgi?page=FunctionalRole&role=DNA%20ligase%20(EC%206.5.1.2)&subsystem_name=DNA_Repair_Base_Excision) |
| DNA Metabolism | DNA repair | [DNA Repair Base Excision](https://rast.nmpdr.org/seedviewer.cgi?page=Subsystems&subsystem=DNA_Repair_Base_Excision&organism=549.408) | [DNA polymerase I (EC 2.7.7.7)](https://rast.nmpdr.org/seedviewer.cgi?page=FunctionalRole&role=DNA%20polymerase%20I%20(EC%202.7.7.7)&subsystem_name=DNA_Repair_Base_Excision) |
| DNA Metabolism | DNA repair | [DNA Repair Base Excision](https://rast.nmpdr.org/seedviewer.cgi?page=Subsystems&subsystem=DNA_Repair_Base_Excision&organism=549.408) | [Exodeoxyribonuclease I (EC 3.1.11.1)](https://rast.nmpdr.org/seedviewer.cgi?page=FunctionalRole&role=Exodeoxyribonuclease%20I%20(EC%203.1.11.1)&subsystem_name=DNA_Repair_Base_Excision) |
| DNA Metabolism | DNA Metabolism - no subcategory | [Type I Restriction-Modification](https://rast.nmpdr.org/seedviewer.cgi?page=Subsystems&subsystem=Type_I_Restriction-Modification&organism=549.408) | [Type I restriction-modification system, specificity subunit S (EC 3.1.21.3)](https://rast.nmpdr.org/seedviewer.cgi?page=FunctionalRole&role=Type%20I%20restriction-modification%20system,%20specificity%20subunit%20S%20(EC%203.1.21.3)&subsystem_name=Type_I_Restriction-Modification) |
| DNA Metabolism | DNA Metabolism - no subcategory | [Type I Restriction-Modification](https://rast.nmpdr.org/seedviewer.cgi?page=Subsystems&subsystem=Type_I_Restriction-Modification&organism=549.408) | [Type I restriction-modification system, restriction subunit R (EC 3.1.21.3)](https://rast.nmpdr.org/seedviewer.cgi?page=FunctionalRole&role=Type%20I%20restriction-modification%20system,%20restriction%20subunit%20R%20(EC%203.1.21.3)&subsystem_name=Type_I_Restriction-Modification) |
| DNA Metabolism | DNA Metabolism - no subcategory | [Type I Restriction-Modification](https://rast.nmpdr.org/seedviewer.cgi?page=Subsystems&subsystem=Type_I_Restriction-Modification&organism=549.408) | [Type I restriction-modification system, DNA-methyltransferase subunit M (EC 2.1.1.72)](https://rast.nmpdr.org/seedviewer.cgi?page=FunctionalRole&role=Type%20I%20restriction-modification%20system,%20DNA-methyltransferase%20subunit%20M%20(EC%202.1.1.72)&subsystem_name=Type_I_Restriction-Modification) |
| DNA Metabolism | DNA Metabolism - no subcategory | [Restriction-Modification System](https://rast.nmpdr.org/seedviewer.cgi?page=Subsystems&subsystem=Restriction-Modification_System&organism=549.408) | [Type I restriction-modification system, specificity subunit S (EC 3.1.21.3)](https://rast.nmpdr.org/seedviewer.cgi?page=FunctionalRole&role=Type%20I%20restriction-modification%20system,%20specificity%20subunit%20S%20(EC%203.1.21.3)&subsystem_name=Restriction-Modification_System) |
| DNA Metabolism | DNA Metabolism - no subcategory | [Restriction-Modification System](https://rast.nmpdr.org/seedviewer.cgi?page=Subsystems&subsystem=Restriction-Modification_System&organism=549.408) | [Type I restriction-modification system, restriction subunit R (EC 3.1.21.3)](https://rast.nmpdr.org/seedviewer.cgi?page=FunctionalRole&role=Type%20I%20restriction-modification%20system,%20restriction%20subunit%20R%20(EC%203.1.21.3)&subsystem_name=Restriction-Modification_System) |
| DNA Metabolism | DNA Metabolism - no subcategory | [Restriction-Modification System](https://rast.nmpdr.org/seedviewer.cgi?page=Subsystems&subsystem=Restriction-Modification_System&organism=549.408) | [Type I restriction-modification system, DNA-methyltransferase subunit M (EC 2.1.1.72)](https://rast.nmpdr.org/seedviewer.cgi?page=FunctionalRole&role=Type%20I%20restriction-modification%20system,%20DNA-methyltransferase%20subunit%20M%20(EC%202.1.1.72)&subsystem_name=Restriction-Modification_System) |
| DNA Metabolism | DNA Metabolism - no subcategory | [YcfH](https://rast.nmpdr.org/seedviewer.cgi?page=Subsystems&subsystem=YcfH&organism=549.408) | [Putative deoxyribonuclease YcfH](https://rast.nmpdr.org/seedviewer.cgi?page=FunctionalRole&role=Putative%20deoxyribonuclease%20YcfH&subsystem_name=YcfH) |
| DNA Metabolism | DNA Metabolism - no subcategory | [YcfH](https://rast.nmpdr.org/seedviewer.cgi?page=Subsystems&subsystem=YcfH&organism=549.408) | [Deoxyribonuclease TatD](https://rast.nmpdr.org/seedviewer.cgi?page=FunctionalRole&role=Deoxyribonuclease%20TatD&subsystem_name=YcfH) |
| DNA Metabolism | DNA Metabolism - no subcategory | [YcfH](https://rast.nmpdr.org/seedviewer.cgi?page=Subsystems&subsystem=YcfH&organism=549.408) | [Putative deoxyribonuclease YjjV](https://rast.nmpdr.org/seedviewer.cgi?page=FunctionalRole&role=Putative%20deoxyribonuclease%20YjjV&subsystem_name=YcfH) |
| DNA Metabolism | DNA Metabolism - no subcategory | [DNA structural proteins, bacterial](https://rast.nmpdr.org/seedviewer.cgi?page=Subsystems&subsystem=DNA_structural_proteins,_bacterial&organism=549.408) | [DNA-binding protein HU-alpha](https://rast.nmpdr.org/seedviewer.cgi?page=FunctionalRole&role=DNA-binding%20protein%20HU-alpha&subsystem_name=DNA_structural_proteins,_bacterial) |
| DNA Metabolism | DNA Metabolism - no subcategory | [DNA structural proteins, bacterial](https://rast.nmpdr.org/seedviewer.cgi?page=Subsystems&subsystem=DNA_structural_proteins,_bacterial&organism=549.408) | [Integration host factor beta subunit](https://rast.nmpdr.org/seedviewer.cgi?page=FunctionalRole&role=Integration%20host%20factor%20beta%20subunit&subsystem_name=DNA_structural_proteins,_bacterial) |
| DNA Metabolism | DNA Metabolism - no subcategory | [DNA structural proteins, bacterial](https://rast.nmpdr.org/seedviewer.cgi?page=Subsystems&subsystem=DNA_structural_proteins,_bacterial&organism=549.408) | [Chromosome partition protein MukB](https://rast.nmpdr.org/seedviewer.cgi?page=FunctionalRole&role=Chromosome%20partition%20protein%20MukB&subsystem_name=DNA_structural_proteins,_bacterial) |
| DNA Metabolism | DNA Metabolism - no subcategory | [DNA structural proteins, bacterial](https://rast.nmpdr.org/seedviewer.cgi?page=Subsystems&subsystem=DNA_structural_proteins,_bacterial&organism=549.408) | [DNA-binding protein Fis](https://rast.nmpdr.org/seedviewer.cgi?page=FunctionalRole&role=DNA-binding%20protein%20Fis&subsystem_name=DNA_structural_proteins,_bacterial) |
| DNA Metabolism | DNA Metabolism - no subcategory | [DNA structural proteins, bacterial](https://rast.nmpdr.org/seedviewer.cgi?page=Subsystems&subsystem=DNA_structural_proteins,_bacterial&organism=549.408) | [Chromosome partition protein MukF](https://rast.nmpdr.org/seedviewer.cgi?page=FunctionalRole&role=Chromosome%20partition%20protein%20MukF&subsystem_name=DNA_structural_proteins,_bacterial) |
| DNA Metabolism | DNA Metabolism - no subcategory | [DNA structural proteins, bacterial](https://rast.nmpdr.org/seedviewer.cgi?page=Subsystems&subsystem=DNA_structural_proteins,_bacterial&organism=549.408) | [DNA-binding protein HU-beta](https://rast.nmpdr.org/seedviewer.cgi?page=FunctionalRole&role=DNA-binding%20protein%20HU-beta&subsystem_name=DNA_structural_proteins,_bacterial) |
| DNA Metabolism | DNA Metabolism - no subcategory | [DNA structural proteins, bacterial](https://rast.nmpdr.org/seedviewer.cgi?page=Subsystems&subsystem=DNA_structural_proteins,_bacterial&organism=549.408) | [Integration host factor alpha subunit](https://rast.nmpdr.org/seedviewer.cgi?page=FunctionalRole&role=Integration%20host%20factor%20alpha%20subunit&subsystem_name=DNA_structural_proteins,_bacterial) |
| DNA Metabolism | DNA Metabolism - no subcategory | [DNA structural proteins, bacterial](https://rast.nmpdr.org/seedviewer.cgi?page=Subsystems&subsystem=DNA_structural_proteins,_bacterial&organism=549.408) | [Chromosome partition protein MukE](https://rast.nmpdr.org/seedviewer.cgi?page=FunctionalRole&role=Chromosome%20partition%20protein%20MukE&subsystem_name=DNA_structural_proteins,_bacterial) |
| DNA Metabolism | DNA replication | [DNA topoisomerases, Type I, ATP-independent](https://rast.nmpdr.org/seedviewer.cgi?page=Subsystems&subsystem=DNA_topoisomerases,_Type_I,_ATP-independent&organism=549.408) | [Similar to C-terminal Zn-finger domain of DNA topoisomerase I](https://rast.nmpdr.org/seedviewer.cgi?page=FunctionalRole&role=Similar%20to%20C-terminal%20Zn-finger%20domain%20of%20DNA%20topoisomerase%20I&subsystem_name=DNA_topoisomerases,_Type_I,_ATP-independent) |
| DNA Metabolism | DNA replication | [DNA topoisomerases, Type I, ATP-independent](https://rast.nmpdr.org/seedviewer.cgi?page=Subsystems&subsystem=DNA_topoisomerases,_Type_I,_ATP-independent&organism=549.408) | [DNA topoisomerase III (EC 5.99.1.2)](https://rast.nmpdr.org/seedviewer.cgi?page=FunctionalRole&role=DNA%20topoisomerase%20III%20(EC%205.99.1.2)&subsystem_name=DNA_topoisomerases,_Type_I,_ATP-independent) |
| DNA Metabolism | DNA replication | [DNA topoisomerases, Type I, ATP-independent](https://rast.nmpdr.org/seedviewer.cgi?page=Subsystems&subsystem=DNA_topoisomerases,_Type_I,_ATP-independent&organism=549.408) | [DNA topoisomerase I (EC 5.99.1.2)](https://rast.nmpdr.org/seedviewer.cgi?page=FunctionalRole&role=DNA%20topoisomerase%20I%20(EC%205.99.1.2)&subsystem_name=DNA_topoisomerases,_Type_I,_ATP-independent) |
| DNA Metabolism | DNA replication | [DNA topoisomerases, Type II, ATP-dependent](https://rast.nmpdr.org/seedviewer.cgi?page=Subsystems&subsystem=DNA_topoisomerases,_Type_II,_ATP-dependent&organism=549.408) | [DNA gyrase subunit B (EC 5.99.1.3)](https://rast.nmpdr.org/seedviewer.cgi?page=FunctionalRole&role=DNA%20gyrase%20subunit%20B%20(EC%205.99.1.3)&subsystem_name=DNA_topoisomerases,_Type_II,_ATP-dependent) |
| DNA Metabolism | DNA replication | [DNA topoisomerases, Type II, ATP-dependent](https://rast.nmpdr.org/seedviewer.cgi?page=Subsystems&subsystem=DNA_topoisomerases,_Type_II,_ATP-dependent&organism=549.408) | [DNA gyrase subunit A (EC 5.99.1.3)](https://rast.nmpdr.org/seedviewer.cgi?page=FunctionalRole&role=DNA%20gyrase%20subunit%20A%20(EC%205.99.1.3)&subsystem_name=DNA_topoisomerases,_Type_II,_ATP-dependent) |
| DNA Metabolism | DNA replication | [DNA topoisomerases, Type II, ATP-dependent](https://rast.nmpdr.org/seedviewer.cgi?page=Subsystems&subsystem=DNA_topoisomerases,_Type_II,_ATP-dependent&organism=549.408) | [Topoisomerase IV subunit B (EC 5.99.1.-)](https://rast.nmpdr.org/seedviewer.cgi?page=FunctionalRole&role=Topoisomerase%20IV%20subunit%20B%20(EC%205.99.1.-)&subsystem_name=DNA_topoisomerases,_Type_II,_ATP-dependent) |
| DNA Metabolism | DNA replication | [DNA topoisomerases, Type II, ATP-dependent](https://rast.nmpdr.org/seedviewer.cgi?page=Subsystems&subsystem=DNA_topoisomerases,_Type_II,_ATP-dependent&organism=549.408) | [Topoisomerase IV subunit A (EC 5.99.1.-)](https://rast.nmpdr.org/seedviewer.cgi?page=FunctionalRole&role=Topoisomerase%20IV%20subunit%20A%20(EC%205.99.1.-)&subsystem_name=DNA_topoisomerases,_Type_II,_ATP-dependent) |
| DNA Metabolism | DNA recombination | [RuvABC plus a hypothetical](https://rast.nmpdr.org/seedviewer.cgi?page=Subsystems&subsystem=RuvABC_plus_a_hypothetical&organism=549.408) | [Crossover junction endodeoxyribonuclease RuvC (EC 3.1.22.4)](https://rast.nmpdr.org/seedviewer.cgi?page=FunctionalRole&role=Crossover%20junction%20endodeoxyribonuclease%20RuvC%20(EC%203.1.22.4)&subsystem_name=RuvABC_plus_a_hypothetical) |
| DNA Metabolism | DNA recombination | [RuvABC plus a hypothetical](https://rast.nmpdr.org/seedviewer.cgi?page=Subsystems&subsystem=RuvABC_plus_a_hypothetical&organism=549.408) | [Holliday junction DNA helicase RuvA](https://rast.nmpdr.org/seedviewer.cgi?page=FunctionalRole&role=Holliday%20junction%20DNA%20helicase%20RuvA&subsystem_name=RuvABC_plus_a_hypothetical) |
| DNA Metabolism | DNA recombination | [RuvABC plus a hypothetical](https://rast.nmpdr.org/seedviewer.cgi?page=Subsystems&subsystem=RuvABC_plus_a_hypothetical&organism=549.408) | [Holliday junction DNA helicase RuvB](https://rast.nmpdr.org/seedviewer.cgi?page=FunctionalRole&role=Holliday%20junction%20DNA%20helicase%20RuvB&subsystem_name=RuvABC_plus_a_hypothetical) |
| DNA Metabolism | DNA uptake, competence | [DNA processing cluster](https://rast.nmpdr.org/seedviewer.cgi?page=Subsystems&subsystem=DNA_processing_cluster&organism=549.408) | [Protein involved in catabolism of external DNA](https://rast.nmpdr.org/seedviewer.cgi?page=FunctionalRole&role=Protein%20involved%20in%20catabolism%20of%20external%20DNA&subsystem_name=DNA_processing_cluster) |
| DNA Metabolism | DNA uptake, competence | [DNA processing cluster](https://rast.nmpdr.org/seedviewer.cgi?page=Subsystems&subsystem=DNA_processing_cluster&organism=549.408) | [FIG000557: hypothetical protein co-occurring with RecR](https://rast.nmpdr.org/seedviewer.cgi?page=FunctionalRole&role=FIG000557:%20hypothetical%20protein%20co-occurring%20with%20RecR&subsystem_name=DNA_processing_cluster) |
| DNA Metabolism | DNA uptake, competence | [DNA processing cluster](https://rast.nmpdr.org/seedviewer.cgi?page=Subsystems&subsystem=DNA_processing_cluster&organism=549.408) | [Recombination protein RecR](https://rast.nmpdr.org/seedviewer.cgi?page=FunctionalRole&role=Recombination%20protein%20RecR&subsystem_name=DNA_processing_cluster) |
| DNA Metabolism | DNA uptake, competence | [DNA processing cluster](https://rast.nmpdr.org/seedviewer.cgi?page=Subsystems&subsystem=DNA_processing_cluster&organism=549.408) | [DNA topoisomerase III (EC 5.99.1.2)](https://rast.nmpdr.org/seedviewer.cgi?page=FunctionalRole&role=DNA%20topoisomerase%20III%20(EC%205.99.1.2)&subsystem_name=DNA_processing_cluster) |
| DNA Metabolism | DNA uptake, competence | [DNA processing cluster](https://rast.nmpdr.org/seedviewer.cgi?page=Subsystems&subsystem=DNA_processing_cluster&organism=549.408) | [DNA polymerase III subunits gamma and tau (EC 2.7.7.7)](https://rast.nmpdr.org/seedviewer.cgi?page=FunctionalRole&role=DNA%20polymerase%20III%20subunits%20gamma%20and%20tau%20(EC%202.7.7.7)&subsystem_name=DNA_processing_cluster) |
| Fatty Acids, Lipids, and Isoprenoids | Phospholipids | [Cardiolipin synthesis](https://rast.nmpdr.org/seedviewer.cgi?page=Subsystems&subsystem=Cardiolipin_synthesis&organism=549.408) | [Cardiolipin synthetase (EC 2.7.8.-)](https://rast.nmpdr.org/seedviewer.cgi?page=FunctionalRole&role=Cardiolipin%20synthetase%20(EC%202.7.8.-)&subsystem_name=Cardiolipin_synthesis) |
| Fatty Acids, Lipids, and Isoprenoids | Phospholipids | [Glycerolipid and Glycerophospholipid Metabolism in Bacteria](https://rast.nmpdr.org/seedviewer.cgi?page=Subsystems&subsystem=Glycerolipid_and_Glycerophospholipid_Metabolism_in_Bacteria&organism=549.408) | [Alcohol dehydrogenase (EC 1.1.1.1)](https://rast.nmpdr.org/seedviewer.cgi?page=FunctionalRole&role=Alcohol%20dehydrogenase%20(EC%201.1.1.1)&subsystem_name=Glycerolipid_and_Glycerophospholipid_Metabolism_in_Bacteria) |
| Fatty Acids, Lipids, and Isoprenoids | Phospholipids | [Glycerolipid and Glycerophospholipid Metabolism in Bacteria](https://rast.nmpdr.org/seedviewer.cgi?page=Subsystems&subsystem=Glycerolipid_and_Glycerophospholipid_Metabolism_in_Bacteria&organism=549.408) | [Acyl-phosphate:glycerol-3-phosphate O-acyltransferase PlsY](https://rast.nmpdr.org/seedviewer.cgi?page=FunctionalRole&role=Acyl-phosphate:glycerol-3-phosphate%20O-acyltransferase%20PlsY&subsystem_name=Glycerolipid_and_Glycerophospholipid_Metabolism_in_Bacteria) |
| Fatty Acids, Lipids, and Isoprenoids | Phospholipids | [Glycerolipid and Glycerophospholipid Metabolism in Bacteria](https://rast.nmpdr.org/seedviewer.cgi?page=Subsystems&subsystem=Glycerolipid_and_Glycerophospholipid_Metabolism_in_Bacteria&organism=549.408) | [Acyl carrier protein (ACP1)](https://rast.nmpdr.org/seedviewer.cgi?page=FunctionalRole&role=Acyl%20carrier%20protein%20(ACP1)&subsystem_name=Glycerolipid_and_Glycerophospholipid_Metabolism_in_Bacteria) |
| Fatty Acids, Lipids, and Isoprenoids | Phospholipids | [Glycerolipid and Glycerophospholipid Metabolism in Bacteria](https://rast.nmpdr.org/seedviewer.cgi?page=Subsystems&subsystem=Glycerolipid_and_Glycerophospholipid_Metabolism_in_Bacteria&organism=549.408) | [Phosphatidylglycerophosphatase A (EC 3.1.3.27)](https://rast.nmpdr.org/seedviewer.cgi?page=FunctionalRole&role=Phosphatidylglycerophosphatase%20A%20(EC%203.1.3.27)&subsystem_name=Glycerolipid_and_Glycerophospholipid_Metabolism_in_Bacteria) |
| Fatty Acids, Lipids, and Isoprenoids | Phospholipids | [Glycerolipid and Glycerophospholipid Metabolism in Bacteria](https://rast.nmpdr.org/seedviewer.cgi?page=Subsystems&subsystem=Glycerolipid_and_Glycerophospholipid_Metabolism_in_Bacteria&organism=549.408) | [CDP-diacylglycerol--serine O-phosphatidyltransferase (EC 2.7.8.8)](https://rast.nmpdr.org/seedviewer.cgi?page=FunctionalRole&role=CDP-diacylglycerol--serine%20O-phosphatidyltransferase%20(EC%202.7.8.8)&subsystem_name=Glycerolipid_and_Glycerophospholipid_Metabolism_in_Bacteria) |
| Fatty Acids, Lipids, and Isoprenoids | Phospholipids | [Glycerolipid and Glycerophospholipid Metabolism in Bacteria](https://rast.nmpdr.org/seedviewer.cgi?page=Subsystems&subsystem=Glycerolipid_and_Glycerophospholipid_Metabolism_in_Bacteria&organism=549.408) | [Glycerol-3-phosphate dehydrogenase [NAD(P)+] (EC 1.1.1.94)](https://rast.nmpdr.org/seedviewer.cgi?page=FunctionalRole&role=Glycerol-3-phosphate%20dehydrogenase%20%5bNAD(P)+%5d%20(EC%201.1.1.94)&subsystem_name=Glycerolipid_and_Glycerophospholipid_Metabolism_in_Bacteria) |
| Fatty Acids, Lipids, and Isoprenoids | Phospholipids | [Glycerolipid and Glycerophospholipid Metabolism in Bacteria](https://rast.nmpdr.org/seedviewer.cgi?page=Subsystems&subsystem=Glycerolipid_and_Glycerophospholipid_Metabolism_in_Bacteria&organism=549.408) | [Glycerol kinase (EC 2.7.1.30)](https://rast.nmpdr.org/seedviewer.cgi?page=FunctionalRole&role=Glycerol%20kinase%20(EC%202.7.1.30)&subsystem_name=Glycerolipid_and_Glycerophospholipid_Metabolism_in_Bacteria) |
| Fatty Acids, Lipids, and Isoprenoids | Phospholipids | [Glycerolipid and Glycerophospholipid Metabolism in Bacteria](https://rast.nmpdr.org/seedviewer.cgi?page=Subsystems&subsystem=Glycerolipid_and_Glycerophospholipid_Metabolism_in_Bacteria&organism=549.408) | [Phosphatidylglycerophosphatase B (EC 3.1.3.27)](https://rast.nmpdr.org/seedviewer.cgi?page=FunctionalRole&role=Phosphatidylglycerophosphatase%20B%20(EC%203.1.3.27)&subsystem_name=Glycerolipid_and_Glycerophospholipid_Metabolism_in_Bacteria) |
| Fatty Acids, Lipids, and Isoprenoids | Phospholipids | [Glycerolipid and Glycerophospholipid Metabolism in Bacteria](https://rast.nmpdr.org/seedviewer.cgi?page=Subsystems&subsystem=Glycerolipid_and_Glycerophospholipid_Metabolism_in_Bacteria&organism=549.408) | [Phosphatidylserine decarboxylase (EC 4.1.1.65)](https://rast.nmpdr.org/seedviewer.cgi?page=FunctionalRole&role=Phosphatidylserine%20decarboxylase%20(EC%204.1.1.65)&subsystem_name=Glycerolipid_and_Glycerophospholipid_Metabolism_in_Bacteria) |
| Fatty Acids, Lipids, and Isoprenoids | Phospholipids | [Glycerolipid and Glycerophospholipid Metabolism in Bacteria](https://rast.nmpdr.org/seedviewer.cgi?page=Subsystems&subsystem=Glycerolipid_and_Glycerophospholipid_Metabolism_in_Bacteria&organism=549.408) | [Glycerol-3-phosphate acyltransferase (EC 2.3.1.15)](https://rast.nmpdr.org/seedviewer.cgi?page=FunctionalRole&role=Glycerol-3-phosphate%20acyltransferase%20(EC%202.3.1.15)&subsystem_name=Glycerolipid_and_Glycerophospholipid_Metabolism_in_Bacteria) |
| Fatty Acids, Lipids, and Isoprenoids | Phospholipids | [Glycerolipid and Glycerophospholipid Metabolism in Bacteria](https://rast.nmpdr.org/seedviewer.cgi?page=Subsystems&subsystem=Glycerolipid_and_Glycerophospholipid_Metabolism_in_Bacteria&organism=549.408) | [Aldehyde dehydrogenase (EC 1.2.1.3)](https://rast.nmpdr.org/seedviewer.cgi?page=FunctionalRole&role=Aldehyde%20dehydrogenase%20(EC%201.2.1.3)&subsystem_name=Glycerolipid_and_Glycerophospholipid_Metabolism_in_Bacteria) |
| Fatty Acids, Lipids, and Isoprenoids | Phospholipids | [Glycerolipid and Glycerophospholipid Metabolism in Bacteria](https://rast.nmpdr.org/seedviewer.cgi?page=Subsystems&subsystem=Glycerolipid_and_Glycerophospholipid_Metabolism_in_Bacteria&organism=549.408) | [Aldehyde dehydrogenase B (EC 1.2.1.22)](https://rast.nmpdr.org/seedviewer.cgi?page=FunctionalRole&role=Aldehyde%20dehydrogenase%20B%20(EC%201.2.1.22)&subsystem_name=Glycerolipid_and_Glycerophospholipid_Metabolism_in_Bacteria) |
| Fatty Acids, Lipids, and Isoprenoids | Phospholipids | [Glycerolipid and Glycerophospholipid Metabolism in Bacteria](https://rast.nmpdr.org/seedviewer.cgi?page=Subsystems&subsystem=Glycerolipid_and_Glycerophospholipid_Metabolism_in_Bacteria&organism=549.408) | [Acyl carrier protein (ACP2)](https://rast.nmpdr.org/seedviewer.cgi?page=FunctionalRole&role=Acyl%20carrier%20protein%20(ACP2)&subsystem_name=Glycerolipid_and_Glycerophospholipid_Metabolism_in_Bacteria) |
| Fatty Acids, Lipids, and Isoprenoids | Phospholipids | [Glycerolipid and Glycerophospholipid Metabolism in Bacteria](https://rast.nmpdr.org/seedviewer.cgi?page=Subsystems&subsystem=Glycerolipid_and_Glycerophospholipid_Metabolism_in_Bacteria&organism=549.408) | [1-acyl-sn-glycerol-3-phosphate acyltransferase (EC 2.3.1.51)](https://rast.nmpdr.org/seedviewer.cgi?page=FunctionalRole&role=1-acyl-sn-glycerol-3-phosphate%20acyltransferase%20(EC%202.3.1.51)&subsystem_name=Glycerolipid_and_Glycerophospholipid_Metabolism_in_Bacteria) |
| Fatty Acids, Lipids, and Isoprenoids | Phospholipids | [Glycerolipid and Glycerophospholipid Metabolism in Bacteria](https://rast.nmpdr.org/seedviewer.cgi?page=Subsystems&subsystem=Glycerolipid_and_Glycerophospholipid_Metabolism_in_Bacteria&organism=549.408) | [Diacylglycerol kinase (EC 2.7.1.107)](https://rast.nmpdr.org/seedviewer.cgi?page=FunctionalRole&role=Diacylglycerol%20kinase%20(EC%202.7.1.107)&subsystem_name=Glycerolipid_and_Glycerophospholipid_Metabolism_in_Bacteria) |
| Fatty Acids, Lipids, and Isoprenoids | Phospholipids | [Glycerolipid and Glycerophospholipid Metabolism in Bacteria](https://rast.nmpdr.org/seedviewer.cgi?page=Subsystems&subsystem=Glycerolipid_and_Glycerophospholipid_Metabolism_in_Bacteria&organism=549.408) | [CDP-diacylglycerol--glycerol-3-phosphate 3-phosphatidyltransferase (EC 2.7.8.5)](https://rast.nmpdr.org/seedviewer.cgi?page=FunctionalRole&role=CDP-diacylglycerol--glycerol-3-phosphate%203-phosphatidyltransferase%20(EC%202.7.8.5)&subsystem_name=Glycerolipid_and_Glycerophospholipid_Metabolism_in_Bacteria) |
| Fatty Acids, Lipids, and Isoprenoids | Phospholipids | [Glycerolipid and Glycerophospholipid Metabolism in Bacteria](https://rast.nmpdr.org/seedviewer.cgi?page=Subsystems&subsystem=Glycerolipid_and_Glycerophospholipid_Metabolism_in_Bacteria&organism=549.408) | [Cardiolipin synthetase (EC 2.7.8.-)](https://rast.nmpdr.org/seedviewer.cgi?page=FunctionalRole&role=Cardiolipin%20synthetase%20(EC%202.7.8.-)&subsystem_name=Glycerolipid_and_Glycerophospholipid_Metabolism_in_Bacteria) |
| Fatty Acids, Lipids, and Isoprenoids | Phospholipids | [Glycerolipid and Glycerophospholipid Metabolism in Bacteria](https://rast.nmpdr.org/seedviewer.cgi?page=Subsystems&subsystem=Glycerolipid_and_Glycerophospholipid_Metabolism_in_Bacteria&organism=549.408) | [Phosphatidate cytidylyltransferase (EC 2.7.7.41)](https://rast.nmpdr.org/seedviewer.cgi?page=FunctionalRole&role=Phosphatidate%20cytidylyltransferase%20(EC%202.7.7.41)&subsystem_name=Glycerolipid_and_Glycerophospholipid_Metabolism_in_Bacteria) |
| Fatty Acids, Lipids, and Isoprenoids | Phospholipids | [Glycerolipid and Glycerophospholipid Metabolism in Bacteria](https://rast.nmpdr.org/seedviewer.cgi?page=Subsystems&subsystem=Glycerolipid_and_Glycerophospholipid_Metabolism_in_Bacteria&organism=549.408) | [Aerobic glycerol-3-phosphate dehydrogenase (EC 1.1.5.3)](https://rast.nmpdr.org/seedviewer.cgi?page=FunctionalRole&role=Aerobic%20glycerol-3-phosphate%20dehydrogenase%20(EC%201.1.5.3)&subsystem_name=Glycerolipid_and_Glycerophospholipid_Metabolism_in_Bacteria) |
| Fatty Acids, Lipids, and Isoprenoids | Phospholipids | [Glycerolipid and Glycerophospholipid Metabolism in Bacteria](https://rast.nmpdr.org/seedviewer.cgi?page=Subsystems&subsystem=Glycerolipid_and_Glycerophospholipid_Metabolism_in_Bacteria&organism=549.408) | [Acyl carrier protein](https://rast.nmpdr.org/seedviewer.cgi?page=FunctionalRole&role=Acyl%20carrier%20protein&subsystem_name=Glycerolipid_and_Glycerophospholipid_Metabolism_in_Bacteria) |
| Fatty Acids, Lipids, and Isoprenoids | Phospholipids | [Glycerolipid and Glycerophospholipid Metabolism in Bacteria](https://rast.nmpdr.org/seedviewer.cgi?page=Subsystems&subsystem=Glycerolipid_and_Glycerophospholipid_Metabolism_in_Bacteria&organism=549.408) | [Phosphate:acyl-ACP acyltransferase PlsX](https://rast.nmpdr.org/seedviewer.cgi?page=FunctionalRole&role=Phosphate:acyl-ACP%20acyltransferase%20PlsX&subsystem_name=Glycerolipid_and_Glycerophospholipid_Metabolism_in_Bacteria) |
| Fatty Acids, Lipids, and Isoprenoids | Phospholipids | [Glycerolipid and Glycerophospholipid Metabolism in Bacteria](https://rast.nmpdr.org/seedviewer.cgi?page=Subsystems&subsystem=Glycerolipid_and_Glycerophospholipid_Metabolism_in_Bacteria&organism=549.408) | [CDP-diacylglycerol pyrophosphatase (EC 3.6.1.26)](https://rast.nmpdr.org/seedviewer.cgi?page=FunctionalRole&role=CDP-diacylglycerol%20pyrophosphatase%20(EC%203.6.1.26)&subsystem_name=Glycerolipid_and_Glycerophospholipid_Metabolism_in_Bacteria) |
| Fatty Acids, Lipids, and Isoprenoids | Phospholipids | [Glycerolipid and Glycerophospholipid Metabolism in Bacteria](https://rast.nmpdr.org/seedviewer.cgi?page=Subsystems&subsystem=Glycerolipid_and_Glycerophospholipid_Metabolism_in_Bacteria&organism=549.408) | [Glycerate kinase (EC 2.7.1.31)](https://rast.nmpdr.org/seedviewer.cgi?page=FunctionalRole&role=Glycerate%20kinase%20(EC%202.7.1.31)&subsystem_name=Glycerolipid_and_Glycerophospholipid_Metabolism_in_Bacteria) |
| Fatty Acids, Lipids, and Isoprenoids | Triacylglycerols | [Triacylglycerol metabolism](https://rast.nmpdr.org/seedviewer.cgi?page=Subsystems&subsystem=Triacylglycerol_metabolism&organism=549.408) | [Lysophospholipase L2 (EC 3.1.1.5)](https://rast.nmpdr.org/seedviewer.cgi?page=FunctionalRole&role=Lysophospholipase%20L2%20(EC%203.1.1.5)&subsystem_name=Triacylglycerol_metabolism) |
| Fatty Acids, Lipids, and Isoprenoids | Triacylglycerols | [Triacylglycerol metabolism](https://rast.nmpdr.org/seedviewer.cgi?page=Subsystems&subsystem=Triacylglycerol_metabolism&organism=549.408) | [Phospholipase A1 precursor (EC 3.1.1.32, EC 3.1.1.4)](https://rast.nmpdr.org/seedviewer.cgi?page=FunctionalRole&role=Phospholipase%20A1%20precursor%20(EC%203.1.1.32,%20EC%203.1.1.4)&subsystem_name=Triacylglycerol_metabolism) |
| Fatty Acids, Lipids, and Isoprenoids | Fatty acids | [Fatty Acid Biosynthesis FASII](https://rast.nmpdr.org/seedviewer.cgi?page=Subsystems&subsystem=Fatty_Acid_Biosynthesis_FASII&organism=549.408) | [3-hydroxyacyl-[acyl-carrier-protein] dehydratase, FabA form (EC 4.2.1.59)](https://rast.nmpdr.org/seedviewer.cgi?page=FunctionalRole&role=3-hydroxyacyl-%5bacyl-carrier-protein%5d%20dehydratase,%20FabA%20form%20(EC%204.2.1.59)&subsystem_name=Fatty_Acid_Biosynthesis_FASII) |
| Fatty Acids, Lipids, and Isoprenoids | Fatty acids | [Fatty Acid Biosynthesis FASII](https://rast.nmpdr.org/seedviewer.cgi?page=Subsystems&subsystem=Fatty_Acid_Biosynthesis_FASII&organism=549.408) | [3-oxoacyl-[acyl-carrier protein] reductase (EC 1.1.1.100)](https://rast.nmpdr.org/seedviewer.cgi?page=FunctionalRole&role=3-oxoacyl-%5bacyl-carrier%20protein%5d%20reductase%20(EC%201.1.1.100)&subsystem_name=Fatty_Acid_Biosynthesis_FASII) |
| Fatty Acids, Lipids, and Isoprenoids | Fatty acids | [Fatty Acid Biosynthesis FASII](https://rast.nmpdr.org/seedviewer.cgi?page=Subsystems&subsystem=Fatty_Acid_Biosynthesis_FASII&organism=549.408) | [Acetyl-coenzyme A carboxyl transferase alpha chain (EC 6.4.1.2)](https://rast.nmpdr.org/seedviewer.cgi?page=FunctionalRole&role=Acetyl-coenzyme%20A%20carboxyl%20transferase%20alpha%20chain%20(EC%206.4.1.2)&subsystem_name=Fatty_Acid_Biosynthesis_FASII) |
| Fatty Acids, Lipids, and Isoprenoids | Fatty acids | [Fatty Acid Biosynthesis FASII](https://rast.nmpdr.org/seedviewer.cgi?page=Subsystems&subsystem=Fatty_Acid_Biosynthesis_FASII&organism=549.408) | [3-oxoacyl-[acyl-carrier-protein] synthase, KASI (EC 2.3.1.41)](https://rast.nmpdr.org/seedviewer.cgi?page=FunctionalRole&role=3-oxoacyl-%5bacyl-carrier-protein%5d%20synthase,%20KASI%20(EC%202.3.1.41)&subsystem_name=Fatty_Acid_Biosynthesis_FASII) |
| Fatty Acids, Lipids, and Isoprenoids | Fatty acids | [Fatty Acid Biosynthesis FASII](https://rast.nmpdr.org/seedviewer.cgi?page=Subsystems&subsystem=Fatty_Acid_Biosynthesis_FASII&organism=549.408) | [Enoyl-[acyl-carrier-protein] reductase [NADH] (EC 1.3.1.9)](https://rast.nmpdr.org/seedviewer.cgi?page=FunctionalRole&role=Enoyl-%5bacyl-carrier-protein%5d%20reductase%20%5bNADH%5d%20(EC%201.3.1.9)&subsystem_name=Fatty_Acid_Biosynthesis_FASII) |
| Fatty Acids, Lipids, and Isoprenoids | Fatty acids | [Fatty Acid Biosynthesis FASII](https://rast.nmpdr.org/seedviewer.cgi?page=Subsystems&subsystem=Fatty_Acid_Biosynthesis_FASII&organism=549.408) | [Holo-[acyl-carrier protein] synthase (EC 2.7.8.7)](https://rast.nmpdr.org/seedviewer.cgi?page=FunctionalRole&role=Holo-%5bacyl-carrier%20protein%5d%20synthase%20(EC%202.7.8.7)&subsystem_name=Fatty_Acid_Biosynthesis_FASII) |
| Fatty Acids, Lipids, and Isoprenoids | Fatty acids | [Fatty Acid Biosynthesis FASII](https://rast.nmpdr.org/seedviewer.cgi?page=Subsystems&subsystem=Fatty_Acid_Biosynthesis_FASII&organism=549.408) | [Biotin carboxyl carrier protein of acetyl-CoA carboxylase](https://rast.nmpdr.org/seedviewer.cgi?page=FunctionalRole&role=Biotin%20carboxyl%20carrier%20protein%20of%20acetyl-CoA%20carboxylase&subsystem_name=Fatty_Acid_Biosynthesis_FASII) |
| Fatty Acids, Lipids, and Isoprenoids | Fatty acids | [Fatty Acid Biosynthesis FASII](https://rast.nmpdr.org/seedviewer.cgi?page=Subsystems&subsystem=Fatty_Acid_Biosynthesis_FASII&organism=549.408) | [Biotin carboxylase of acetyl-CoA carboxylase (EC 6.3.4.14)](https://rast.nmpdr.org/seedviewer.cgi?page=FunctionalRole&role=Biotin%20carboxylase%20of%20acetyl-CoA%20carboxylase%20(EC%206.3.4.14)&subsystem_name=Fatty_Acid_Biosynthesis_FASII) |
| Fatty Acids, Lipids, and Isoprenoids | Fatty acids | [Fatty Acid Biosynthesis FASII](https://rast.nmpdr.org/seedviewer.cgi?page=Subsystems&subsystem=Fatty_Acid_Biosynthesis_FASII&organism=549.408) | [Acetyl-coenzyme A carboxyl transferase beta chain (EC 6.4.1.2)](https://rast.nmpdr.org/seedviewer.cgi?page=FunctionalRole&role=Acetyl-coenzyme%20A%20carboxyl%20transferase%20beta%20chain%20(EC%206.4.1.2)&subsystem_name=Fatty_Acid_Biosynthesis_FASII) |
| Fatty Acids, Lipids, and Isoprenoids | Fatty acids | [Fatty Acid Biosynthesis FASII](https://rast.nmpdr.org/seedviewer.cgi?page=Subsystems&subsystem=Fatty_Acid_Biosynthesis_FASII&organism=549.408) | [Acyl carrier protein](https://rast.nmpdr.org/seedviewer.cgi?page=FunctionalRole&role=Acyl%20carrier%20protein&subsystem_name=Fatty_Acid_Biosynthesis_FASII) |
| Fatty Acids, Lipids, and Isoprenoids | Fatty acids | [Fatty Acid Biosynthesis FASII](https://rast.nmpdr.org/seedviewer.cgi?page=Subsystems&subsystem=Fatty_Acid_Biosynthesis_FASII&organism=549.408) | [Acyl carrier protein phosphodiesterase (EC 3.1.4.14)](https://rast.nmpdr.org/seedviewer.cgi?page=FunctionalRole&role=Acyl%20carrier%20protein%20phosphodiesterase%20(EC%203.1.4.14)&subsystem_name=Fatty_Acid_Biosynthesis_FASII) |
| Fatty Acids, Lipids, and Isoprenoids | Fatty acids | [Fatty Acid Biosynthesis FASII](https://rast.nmpdr.org/seedviewer.cgi?page=Subsystems&subsystem=Fatty_Acid_Biosynthesis_FASII&organism=549.408) | [Malonyl CoA-acyl carrier protein transacylase (EC 2.3.1.39)](https://rast.nmpdr.org/seedviewer.cgi?page=FunctionalRole&role=Malonyl%20CoA-acyl%20carrier%20protein%20transacylase%20(EC%202.3.1.39)&subsystem_name=Fatty_Acid_Biosynthesis_FASII) |
| Fatty Acids, Lipids, and Isoprenoids | Fatty acids | [Fatty Acid Biosynthesis FASII](https://rast.nmpdr.org/seedviewer.cgi?page=Subsystems&subsystem=Fatty_Acid_Biosynthesis_FASII&organism=549.408) | [Enoyl-[acyl-carrier-protein] reductase [FMN] (EC 1.3.1.9)](https://rast.nmpdr.org/seedviewer.cgi?page=FunctionalRole&role=Enoyl-%5bacyl-carrier-protein%5d%20reductase%20%5bFMN%5d%20(EC%201.3.1.9)&subsystem_name=Fatty_Acid_Biosynthesis_FASII) |
| Fatty Acids, Lipids, and Isoprenoids | Fatty acids | [Fatty Acid Biosynthesis FASII](https://rast.nmpdr.org/seedviewer.cgi?page=Subsystems&subsystem=Fatty_Acid_Biosynthesis_FASII&organism=549.408) | [3-hydroxyacyl-[acyl-carrier-protein] dehydratase, FabZ form (EC 4.2.1.59)](https://rast.nmpdr.org/seedviewer.cgi?page=FunctionalRole&role=3-hydroxyacyl-%5bacyl-carrier-protein%5d%20dehydratase,%20FabZ%20form%20(EC%204.2.1.59)&subsystem_name=Fatty_Acid_Biosynthesis_FASII) |
| Fatty Acids, Lipids, and Isoprenoids | Fatty acids | [Phospholipid and Fatty acid biosynthesis related cluster](https://rast.nmpdr.org/seedviewer.cgi?page=Subsystems&subsystem=Phospholipid_and_Fatty_acid_biosynthesis_related_cluster&organism=549.408) | [FIG027190: Putative transmembrane protein](https://rast.nmpdr.org/seedviewer.cgi?page=FunctionalRole&role=FIG027190:%20Putative%20transmembrane%20protein&subsystem_name=Phospholipid_and_Fatty_acid_biosynthesis_related_cluster) |
| Fatty Acids, Lipids, and Isoprenoids | Fatty acids | [Phospholipid and Fatty acid biosynthesis related cluster](https://rast.nmpdr.org/seedviewer.cgi?page=Subsystems&subsystem=Phospholipid_and_Fatty_acid_biosynthesis_related_cluster&organism=549.408) | [Acyl carrier protein (ACP1)](https://rast.nmpdr.org/seedviewer.cgi?page=FunctionalRole&role=Acyl%20carrier%20protein%20(ACP1)&subsystem_name=Phospholipid_and_Fatty_acid_biosynthesis_related_cluster) |
| Fatty Acids, Lipids, and Isoprenoids | Fatty acids | [Phospholipid and Fatty acid biosynthesis related cluster](https://rast.nmpdr.org/seedviewer.cgi?page=Subsystems&subsystem=Phospholipid_and_Fatty_acid_biosynthesis_related_cluster&organism=549.408) | [FIG143263: Glycosyl transferase](https://rast.nmpdr.org/seedviewer.cgi?page=FunctionalRole&role=FIG143263:%20Glycosyl%20transferase&subsystem_name=Phospholipid_and_Fatty_acid_biosynthesis_related_cluster) |
| Fatty Acids, Lipids, and Isoprenoids | Fatty acids | [Phospholipid and Fatty acid biosynthesis related cluster](https://rast.nmpdr.org/seedviewer.cgi?page=Subsystems&subsystem=Phospholipid_and_Fatty_acid_biosynthesis_related_cluster&organism=549.408) | [3-hydroxydecanoyl-[ACP] dehydratase (EC 4.2.1.60)](https://rast.nmpdr.org/seedviewer.cgi?page=FunctionalRole&role=3-hydroxydecanoyl-%5bACP%5d%20dehydratase%20(EC%204.2.1.60)&subsystem_name=Phospholipid_and_Fatty_acid_biosynthesis_related_cluster) |
| Fatty Acids, Lipids, and Isoprenoids | Fatty acids | [Phospholipid and Fatty acid biosynthesis related cluster](https://rast.nmpdr.org/seedviewer.cgi?page=Subsystems&subsystem=Phospholipid_and_Fatty_acid_biosynthesis_related_cluster&organism=549.408) | [Lysophospholipid acyltransferase](https://rast.nmpdr.org/seedviewer.cgi?page=FunctionalRole&role=Lysophospholipid%20acyltransferase&subsystem_name=Phospholipid_and_Fatty_acid_biosynthesis_related_cluster) |
| Fatty Acids, Lipids, and Isoprenoids | Fatty acids | [Phospholipid and Fatty acid biosynthesis related cluster](https://rast.nmpdr.org/seedviewer.cgi?page=Subsystems&subsystem=Phospholipid_and_Fatty_acid_biosynthesis_related_cluster&organism=549.408) | [FIG018329: 1-acyl-sn-glycerol-3-phosphate acyltransferase](https://rast.nmpdr.org/seedviewer.cgi?page=FunctionalRole&role=FIG018329:%201-acyl-sn-glycerol-3-phosphate%20acyltransferase&subsystem_name=Phospholipid_and_Fatty_acid_biosynthesis_related_cluster) |
| Fatty Acids, Lipids, and Isoprenoids | Fatty acids | [Phospholipid and Fatty acid biosynthesis related cluster](https://rast.nmpdr.org/seedviewer.cgi?page=Subsystems&subsystem=Phospholipid_and_Fatty_acid_biosynthesis_related_cluster&organism=549.408) | [3-oxoacyl-[ACP] reductase (EC 1.1.1.100)](https://rast.nmpdr.org/seedviewer.cgi?page=FunctionalRole&role=3-oxoacyl-%5bACP%5d%20reductase%20(EC%201.1.1.100)&subsystem_name=Phospholipid_and_Fatty_acid_biosynthesis_related_cluster) |
| Fatty Acids, Lipids, and Isoprenoids | Fatty acids | [Phospholipid and Fatty acid biosynthesis related cluster](https://rast.nmpdr.org/seedviewer.cgi?page=Subsystems&subsystem=Phospholipid_and_Fatty_acid_biosynthesis_related_cluster&organism=549.408) | [Acyl carrier protein (ACP2)](https://rast.nmpdr.org/seedviewer.cgi?page=FunctionalRole&role=Acyl%20carrier%20protein%20(ACP2)&subsystem_name=Phospholipid_and_Fatty_acid_biosynthesis_related_cluster) |
| Fatty Acids, Lipids, and Isoprenoids | Fatty acids | [Phospholipid and Fatty acid biosynthesis related cluster](https://rast.nmpdr.org/seedviewer.cgi?page=Subsystems&subsystem=Phospholipid_and_Fatty_acid_biosynthesis_related_cluster&organism=549.408) | [FIG002571: 4-hydroxybenzoyl-CoA thioesterase domain protein](https://rast.nmpdr.org/seedviewer.cgi?page=FunctionalRole&role=FIG002571:%204-hydroxybenzoyl-CoA%20thioesterase%20domain%20protein&subsystem_name=Phospholipid_and_Fatty_acid_biosynthesis_related_cluster) |
| Fatty Acids, Lipids, and Isoprenoids | Fatty acids | [Phospholipid and Fatty acid biosynthesis related cluster](https://rast.nmpdr.org/seedviewer.cgi?page=Subsystems&subsystem=Phospholipid_and_Fatty_acid_biosynthesis_related_cluster&organism=549.408) | [FIG138576: 3-oxoacyl-[ACP] synthase (EC 2.3.1.41)](https://rast.nmpdr.org/seedviewer.cgi?page=FunctionalRole&role=FIG138576:%203-oxoacyl-%5bACP%5d%20synthase%20(EC%202.3.1.41)&subsystem_name=Phospholipid_and_Fatty_acid_biosynthesis_related_cluster) |
| Fatty Acids, Lipids, and Isoprenoids | Fatty acids | [Phospholipid and Fatty acid biosynthesis related cluster](https://rast.nmpdr.org/seedviewer.cgi?page=Subsystems&subsystem=Phospholipid_and_Fatty_acid_biosynthesis_related_cluster&organism=549.408) | [FIGfam138462: Acyl-CoA synthetase, AMP-(fatty) acid ligase](https://rast.nmpdr.org/seedviewer.cgi?page=FunctionalRole&role=FIGfam138462:%20Acyl-CoA%20synthetase,%20AMP-(fatty)%20acid%20ligase&subsystem_name=Phospholipid_and_Fatty_acid_biosynthesis_related_cluster) |
| Fatty Acids, Lipids, and Isoprenoids | Fatty acids | [Phospholipid and Fatty acid biosynthesis related cluster](https://rast.nmpdr.org/seedviewer.cgi?page=Subsystems&subsystem=Phospholipid_and_Fatty_acid_biosynthesis_related_cluster&organism=549.408) | [(3R)-hydroxymyristoyl-[ACP] dehydratase (EC 4.2.1.-)](https://rast.nmpdr.org/seedviewer.cgi?page=FunctionalRole&role=(3R)-hydroxymyristoyl-%5bACP%5d%20dehydratase%20(EC%204.2.1.-)&subsystem_name=Phospholipid_and_Fatty_acid_biosynthesis_related_cluster) |
| Fatty Acids, Lipids, and Isoprenoids | Fatty acids | [Phospholipid and Fatty acid biosynthesis related cluster](https://rast.nmpdr.org/seedviewer.cgi?page=Subsystems&subsystem=Phospholipid_and_Fatty_acid_biosynthesis_related_cluster&organism=549.408) | [3-oxoacyl-[ACP] synthase](https://rast.nmpdr.org/seedviewer.cgi?page=FunctionalRole&role=3-oxoacyl-%5bACP%5d%20synthase&subsystem_name=Phospholipid_and_Fatty_acid_biosynthesis_related_cluster) |
| Fatty Acids, Lipids, and Isoprenoids | Fatty acids | [Phospholipid and Fatty acid biosynthesis related cluster](https://rast.nmpdr.org/seedviewer.cgi?page=Subsystems&subsystem=Phospholipid_and_Fatty_acid_biosynthesis_related_cluster&organism=549.408) | [FIG035331: hypothetical protein](https://rast.nmpdr.org/seedviewer.cgi?page=FunctionalRole&role=FIG035331:%20hypothetical%20protein&subsystem_name=Phospholipid_and_Fatty_acid_biosynthesis_related_cluster) |
| Fatty Acids, Lipids, and Isoprenoids | Fatty acids | [Phospholipid and Fatty acid biosynthesis related cluster](https://rast.nmpdr.org/seedviewer.cgi?page=Subsystems&subsystem=Phospholipid_and_Fatty_acid_biosynthesis_related_cluster&organism=549.408) | [3-oxoacyl-[ACP] synthase (EC 2.3.1.41) FabV like](https://rast.nmpdr.org/seedviewer.cgi?page=FunctionalRole&role=3-oxoacyl-%5bACP%5d%20synthase%20(EC%202.3.1.41)%20FabV%20like&subsystem_name=Phospholipid_and_Fatty_acid_biosynthesis_related_cluster) |
| Fatty Acids, Lipids, and Isoprenoids | Fatty acids | [Phospholipid and Fatty acid biosynthesis related cluster](https://rast.nmpdr.org/seedviewer.cgi?page=Subsystems&subsystem=Phospholipid_and_Fatty_acid_biosynthesis_related_cluster&organism=549.408) | [FIG021862: membrane protein, exporter](https://rast.nmpdr.org/seedviewer.cgi?page=FunctionalRole&role=FIG021862:%20membrane%20protein,%20exporter&subsystem_name=Phospholipid_and_Fatty_acid_biosynthesis_related_cluster) |
| Fatty Acids, Lipids, and Isoprenoids | Fatty acids | [Phospholipid and Fatty acid biosynthesis related cluster](https://rast.nmpdr.org/seedviewer.cgi?page=Subsystems&subsystem=Phospholipid_and_Fatty_acid_biosynthesis_related_cluster&organism=549.408) | [FIG017861: hypothetical protein](https://rast.nmpdr.org/seedviewer.cgi?page=FunctionalRole&role=FIG017861:%20hypothetical%20protein&subsystem_name=Phospholipid_and_Fatty_acid_biosynthesis_related_cluster) |
| Fatty Acids, Lipids, and Isoprenoids | Fatty acids | [Fatty acid metabolism cluster](https://rast.nmpdr.org/seedviewer.cgi?page=Subsystems&subsystem=Fatty_acid_metabolism_cluster&organism=549.408) | [3-hydroxybutyryl-CoA epimerase (EC 5.1.2.3)](https://rast.nmpdr.org/seedviewer.cgi?page=FunctionalRole&role=3-hydroxybutyryl-CoA%20epimerase%20(EC%205.1.2.3)&subsystem_name=Fatty_acid_metabolism_cluster) |
| Fatty Acids, Lipids, and Isoprenoids | Fatty acids | [Fatty acid metabolism cluster](https://rast.nmpdr.org/seedviewer.cgi?page=Subsystems&subsystem=Fatty_acid_metabolism_cluster&organism=549.408) | [Long-chain-fatty-acid--CoA ligase (EC 6.2.1.3)](https://rast.nmpdr.org/seedviewer.cgi?page=FunctionalRole&role=Long-chain-fatty-acid--CoA%20ligase%20(EC%206.2.1.3)&subsystem_name=Fatty_acid_metabolism_cluster) |
| Fatty Acids, Lipids, and Isoprenoids | Fatty acids | [Fatty acid metabolism cluster](https://rast.nmpdr.org/seedviewer.cgi?page=Subsystems&subsystem=Fatty_acid_metabolism_cluster&organism=549.408) | [Enoyl-CoA hydratase (EC 4.2.1.17)](https://rast.nmpdr.org/seedviewer.cgi?page=FunctionalRole&role=Enoyl-CoA%20hydratase%20(EC%204.2.1.17)&subsystem_name=Fatty_acid_metabolism_cluster) |
| Fatty Acids, Lipids, and Isoprenoids | Fatty acids | [Fatty acid metabolism cluster](https://rast.nmpdr.org/seedviewer.cgi?page=Subsystems&subsystem=Fatty_acid_metabolism_cluster&organism=549.408) | [3-ketoacyl-CoA thiolase (EC 2.3.1.16)](https://rast.nmpdr.org/seedviewer.cgi?page=FunctionalRole&role=3-ketoacyl-CoA%20thiolase%20(EC%202.3.1.16)&subsystem_name=Fatty_acid_metabolism_cluster) |
| Fatty Acids, Lipids, and Isoprenoids | Fatty acids | [Fatty acid metabolism cluster](https://rast.nmpdr.org/seedviewer.cgi?page=Subsystems&subsystem=Fatty_acid_metabolism_cluster&organism=549.408) | [3-hydroxyacyl-CoA dehydrogenase (EC 1.1.1.35)](https://rast.nmpdr.org/seedviewer.cgi?page=FunctionalRole&role=3-hydroxyacyl-CoA%20dehydrogenase%20(EC%201.1.1.35)&subsystem_name=Fatty_acid_metabolism_cluster) |
| Fatty Acids, Lipids, and Isoprenoids | Fatty acids | [Acyl-CoA thioesterase II](https://rast.nmpdr.org/seedviewer.cgi?page=Subsystems&subsystem=Acyl-CoA_thioesterase_II&organism=549.408) | [Acyl-CoA thioesterase II (EC 3.1.2.-)](https://rast.nmpdr.org/seedviewer.cgi?page=FunctionalRole&role=Acyl-CoA%20thioesterase%20II%20(EC%203.1.2.-)&subsystem_name=Acyl-CoA_thioesterase_II) |
| Fatty Acids, Lipids, and Isoprenoids | Fatty Acids, Lipids, and Isoprenoids - no subcategory | [Polyhydroxybutyrate metabolism](https://rast.nmpdr.org/seedviewer.cgi?page=Subsystems&subsystem=Polyhydroxybutyrate_metabolism&organism=549.408) | [3-hydroxybutyryl-CoA epimerase (EC 5.1.2.3)](https://rast.nmpdr.org/seedviewer.cgi?page=FunctionalRole&role=3-hydroxybutyryl-CoA%20epimerase%20(EC%205.1.2.3)&subsystem_name=Polyhydroxybutyrate_metabolism) |
| Fatty Acids, Lipids, and Isoprenoids | Fatty Acids, Lipids, and Isoprenoids - no subcategory | [Polyhydroxybutyrate metabolism](https://rast.nmpdr.org/seedviewer.cgi?page=Subsystems&subsystem=Polyhydroxybutyrate_metabolism&organism=549.408) | [Enoyl-CoA hydratase (EC 4.2.1.17)](https://rast.nmpdr.org/seedviewer.cgi?page=FunctionalRole&role=Enoyl-CoA%20hydratase%20(EC%204.2.1.17)&subsystem_name=Polyhydroxybutyrate_metabolism) |
| Fatty Acids, Lipids, and Isoprenoids | Fatty Acids, Lipids, and Isoprenoids - no subcategory | [Polyhydroxybutyrate metabolism](https://rast.nmpdr.org/seedviewer.cgi?page=Subsystems&subsystem=Polyhydroxybutyrate_metabolism&organism=549.408) | [3-ketoacyl-CoA thiolase (EC 2.3.1.16)](https://rast.nmpdr.org/seedviewer.cgi?page=FunctionalRole&role=3-ketoacyl-CoA%20thiolase%20(EC%202.3.1.16)&subsystem_name=Polyhydroxybutyrate_metabolism) |
| Fatty Acids, Lipids, and Isoprenoids | Fatty Acids, Lipids, and Isoprenoids - no subcategory | [Polyhydroxybutyrate metabolism](https://rast.nmpdr.org/seedviewer.cgi?page=Subsystems&subsystem=Polyhydroxybutyrate_metabolism&organism=549.408) | [3-hydroxyacyl-CoA dehydrogenase (EC 1.1.1.35)](https://rast.nmpdr.org/seedviewer.cgi?page=FunctionalRole&role=3-hydroxyacyl-CoA%20dehydrogenase%20(EC%201.1.1.35)&subsystem_name=Polyhydroxybutyrate_metabolism) |
| Fatty Acids, Lipids, and Isoprenoids | Fatty Acids, Lipids, and Isoprenoids - no subcategory | [Polyhydroxybutyrate metabolism](https://rast.nmpdr.org/seedviewer.cgi?page=Subsystems&subsystem=Polyhydroxybutyrate_metabolism&organism=549.408) | [Acetyl-CoA acetyltransferase (EC 2.3.1.9)](https://rast.nmpdr.org/seedviewer.cgi?page=FunctionalRole&role=Acetyl-CoA%20acetyltransferase%20(EC%202.3.1.9)&subsystem_name=Polyhydroxybutyrate_metabolism) |
| Fatty Acids, Lipids, and Isoprenoids | Fatty Acids, Lipids, and Isoprenoids - no subcategory | [Polyhydroxybutyrate metabolism](https://rast.nmpdr.org/seedviewer.cgi?page=Subsystems&subsystem=Polyhydroxybutyrate_metabolism&organism=549.408) | [D-beta-hydroxybutyrate dehydrogenase (EC 1.1.1.30)](https://rast.nmpdr.org/seedviewer.cgi?page=FunctionalRole&role=D-beta-hydroxybutyrate%20dehydrogenase%20(EC%201.1.1.30)&subsystem_name=Polyhydroxybutyrate_metabolism) |
| Fatty Acids, Lipids, and Isoprenoids | Isoprenoids | [Isoprenoinds for Quinones](https://rast.nmpdr.org/seedviewer.cgi?page=Subsystems&subsystem=Isoprenoinds_for_Quinones&organism=549.408) | [Octaprenyl diphosphate synthase (EC 2.5.1.90)](https://rast.nmpdr.org/seedviewer.cgi?page=FunctionalRole&role=Octaprenyl%20diphosphate%20synthase%20(EC%202.5.1.90)&subsystem_name=Isoprenoinds_for_Quinones) |
| Fatty Acids, Lipids, and Isoprenoids | Isoprenoids | [Isoprenoinds for Quinones](https://rast.nmpdr.org/seedviewer.cgi?page=Subsystems&subsystem=Isoprenoinds_for_Quinones&organism=549.408) | [Dimethylallyltransferase (EC 2.5.1.1)](https://rast.nmpdr.org/seedviewer.cgi?page=FunctionalRole&role=Dimethylallyltransferase%20(EC%202.5.1.1)&subsystem_name=Isoprenoinds_for_Quinones) |
| Fatty Acids, Lipids, and Isoprenoids | Isoprenoids | [Isoprenoinds for Quinones](https://rast.nmpdr.org/seedviewer.cgi?page=Subsystems&subsystem=Isoprenoinds_for_Quinones&organism=549.408) | [Undecaprenyl diphosphate synthase (EC 2.5.1.31)](https://rast.nmpdr.org/seedviewer.cgi?page=FunctionalRole&role=Undecaprenyl%20diphosphate%20synthase%20(EC%202.5.1.31)&subsystem_name=Isoprenoinds_for_Quinones) |
| Fatty Acids, Lipids, and Isoprenoids | Isoprenoids | [Isoprenoinds for Quinones](https://rast.nmpdr.org/seedviewer.cgi?page=Subsystems&subsystem=Isoprenoinds_for_Quinones&organism=549.408) | [(2E,6E)-farnesyl diphosphate synthase (EC 2.5.1.10)](https://rast.nmpdr.org/seedviewer.cgi?page=FunctionalRole&role=(2E,6E)-farnesyl%20diphosphate%20synthase%20(EC%202.5.1.10)&subsystem_name=Isoprenoinds_for_Quinones) |
| Fatty Acids, Lipids, and Isoprenoids | Isoprenoids | [Isoprenoid Biosynthesis](https://rast.nmpdr.org/seedviewer.cgi?page=Subsystems&subsystem=Isoprenoid_Biosynthesis&organism=549.408) | [Dimethylallyltransferase (EC 2.5.1.1)](https://rast.nmpdr.org/seedviewer.cgi?page=FunctionalRole&role=Dimethylallyltransferase%20(EC%202.5.1.1)&subsystem_name=Isoprenoid_Biosynthesis) |
| Fatty Acids, Lipids, and Isoprenoids | Isoprenoids | [Isoprenoid Biosynthesis](https://rast.nmpdr.org/seedviewer.cgi?page=Subsystems&subsystem=Isoprenoid_Biosynthesis&organism=549.408) | [4-hydroxy-3-methylbut-2-enyl diphosphate reductase (EC 1.17.1.2)](https://rast.nmpdr.org/seedviewer.cgi?page=FunctionalRole&role=4-hydroxy-3-methylbut-2-enyl%20diphosphate%20reductase%20(EC%201.17.1.2)&subsystem_name=Isoprenoid_Biosynthesis) |
| Fatty Acids, Lipids, and Isoprenoids | Isoprenoids | [Isoprenoid Biosynthesis](https://rast.nmpdr.org/seedviewer.cgi?page=Subsystems&subsystem=Isoprenoid_Biosynthesis&organism=549.408) | [4-diphosphocytidyl-2-C-methyl-D-erythritol kinase (EC 2.7.1.148)](https://rast.nmpdr.org/seedviewer.cgi?page=FunctionalRole&role=4-diphosphocytidyl-2-C-methyl-D-erythritol%20kinase%20(EC%202.7.1.148)&subsystem_name=Isoprenoid_Biosynthesis) |
| Fatty Acids, Lipids, and Isoprenoids | Isoprenoids | [Isoprenoid Biosynthesis](https://rast.nmpdr.org/seedviewer.cgi?page=Subsystems&subsystem=Isoprenoid_Biosynthesis&organism=549.408) | [2-C-methyl-D-erythritol 2,4-cyclodiphosphate synthase (EC 4.6.1.12)](https://rast.nmpdr.org/seedviewer.cgi?page=FunctionalRole&role=2-C-methyl-D-erythritol%202,4-cyclodiphosphate%20synthase%20(EC%204.6.1.12)&subsystem_name=Isoprenoid_Biosynthesis) |
| Fatty Acids, Lipids, and Isoprenoids | Isoprenoids | [Isoprenoid Biosynthesis](https://rast.nmpdr.org/seedviewer.cgi?page=Subsystems&subsystem=Isoprenoid_Biosynthesis&organism=549.408) | [Acetyl-CoA acetyltransferase (EC 2.3.1.9)](https://rast.nmpdr.org/seedviewer.cgi?page=FunctionalRole&role=Acetyl-CoA%20acetyltransferase%20(EC%202.3.1.9)&subsystem_name=Isoprenoid_Biosynthesis) |
| Fatty Acids, Lipids, and Isoprenoids | Isoprenoids | [Isoprenoid Biosynthesis](https://rast.nmpdr.org/seedviewer.cgi?page=Subsystems&subsystem=Isoprenoid_Biosynthesis&organism=549.408) | [1-deoxy-D-xylulose 5-phosphate synthase (EC 2.2.1.7)](https://rast.nmpdr.org/seedviewer.cgi?page=FunctionalRole&role=1-deoxy-D-xylulose%205-phosphate%20synthase%20(EC%202.2.1.7)&subsystem_name=Isoprenoid_Biosynthesis) |
| Fatty Acids, Lipids, and Isoprenoids | Isoprenoids | [Isoprenoid Biosynthesis](https://rast.nmpdr.org/seedviewer.cgi?page=Subsystems&subsystem=Isoprenoid_Biosynthesis&organism=549.408) | [1-deoxy-D-xylulose 5-phosphate reductoisomerase (EC 1.1.1.267)](https://rast.nmpdr.org/seedviewer.cgi?page=FunctionalRole&role=1-deoxy-D-xylulose%205-phosphate%20reductoisomerase%20(EC%201.1.1.267)&subsystem_name=Isoprenoid_Biosynthesis) |
| Fatty Acids, Lipids, and Isoprenoids | Isoprenoids | [Isoprenoid Biosynthesis](https://rast.nmpdr.org/seedviewer.cgi?page=Subsystems&subsystem=Isoprenoid_Biosynthesis&organism=549.408) | [Undecaprenyl diphosphate synthase (EC 2.5.1.31)](https://rast.nmpdr.org/seedviewer.cgi?page=FunctionalRole&role=Undecaprenyl%20diphosphate%20synthase%20(EC%202.5.1.31)&subsystem_name=Isoprenoid_Biosynthesis) |
| Fatty Acids, Lipids, and Isoprenoids | Isoprenoids | [Isoprenoid Biosynthesis](https://rast.nmpdr.org/seedviewer.cgi?page=Subsystems&subsystem=Isoprenoid_Biosynthesis&organism=549.408) | [1-hydroxy-2-methyl-2-(E)-butenyl 4-diphosphate synthase (EC 1.17.7.1)](https://rast.nmpdr.org/seedviewer.cgi?page=FunctionalRole&role=1-hydroxy-2-methyl-2-(E)-butenyl%204-diphosphate%20synthase%20(EC%201.17.7.1)&subsystem_name=Isoprenoid_Biosynthesis) |
| Fatty Acids, Lipids, and Isoprenoids | Isoprenoids | [Isoprenoid Biosynthesis](https://rast.nmpdr.org/seedviewer.cgi?page=Subsystems&subsystem=Isoprenoid_Biosynthesis&organism=549.408) | [Isopentenyl-diphosphate delta-isomerase (EC 5.3.3.2)](https://rast.nmpdr.org/seedviewer.cgi?page=FunctionalRole&role=Isopentenyl-diphosphate%20delta-isomerase%20(EC%205.3.3.2)&subsystem_name=Isoprenoid_Biosynthesis) |
| Fatty Acids, Lipids, and Isoprenoids | Isoprenoids | [Isoprenoid Biosynthesis](https://rast.nmpdr.org/seedviewer.cgi?page=Subsystems&subsystem=Isoprenoid_Biosynthesis&organism=549.408) | [(2E,6E)-farnesyl diphosphate synthase (EC 2.5.1.10)](https://rast.nmpdr.org/seedviewer.cgi?page=FunctionalRole&role=(2E,6E)-farnesyl%20diphosphate%20synthase%20(EC%202.5.1.10)&subsystem_name=Isoprenoid_Biosynthesis) |
| Fatty Acids, Lipids, and Isoprenoids | Isoprenoids | [Isoprenoid Biosynthesis](https://rast.nmpdr.org/seedviewer.cgi?page=Subsystems&subsystem=Isoprenoid_Biosynthesis&organism=549.408) | [2-C-methyl-D-erythritol 4-phosphate cytidylyltransferase (EC 2.7.7.60)](https://rast.nmpdr.org/seedviewer.cgi?page=FunctionalRole&role=2-C-methyl-D-erythritol%204-phosphate%20cytidylyltransferase%20(EC%202.7.7.60)&subsystem_name=Isoprenoid_Biosynthesis) |
| Fatty Acids, Lipids, and Isoprenoids | Isoprenoids | [Polyprenyl Diphosphate Biosynthesis](https://rast.nmpdr.org/seedviewer.cgi?page=Subsystems&subsystem=Polyprenyl_Diphosphate_Biosynthesis&organism=549.408) | [Octaprenyl diphosphate synthase (EC 2.5.1.90)](https://rast.nmpdr.org/seedviewer.cgi?page=FunctionalRole&role=Octaprenyl%20diphosphate%20synthase%20(EC%202.5.1.90)&subsystem_name=Polyprenyl_Diphosphate_Biosynthesis) |
| Fatty Acids, Lipids, and Isoprenoids | Isoprenoids | [Polyprenyl Diphosphate Biosynthesis](https://rast.nmpdr.org/seedviewer.cgi?page=Subsystems&subsystem=Polyprenyl_Diphosphate_Biosynthesis&organism=549.408) | [Undecaprenyl diphosphate synthase (EC 2.5.1.31)](https://rast.nmpdr.org/seedviewer.cgi?page=FunctionalRole&role=Undecaprenyl%20diphosphate%20synthase%20(EC%202.5.1.31)&subsystem_name=Polyprenyl_Diphosphate_Biosynthesis) |
| Fatty Acids, Lipids, and Isoprenoids | Isoprenoids | [Polyprenyl Diphosphate Biosynthesis](https://rast.nmpdr.org/seedviewer.cgi?page=Subsystems&subsystem=Polyprenyl_Diphosphate_Biosynthesis&organism=549.408) | [(2E,6E)-farnesyl diphosphate synthase (EC 2.5.1.10)](https://rast.nmpdr.org/seedviewer.cgi?page=FunctionalRole&role=(2E,6E)-farnesyl%20diphosphate%20synthase%20(EC%202.5.1.10)&subsystem_name=Polyprenyl_Diphosphate_Biosynthesis) |
| Fatty Acids, Lipids, and Isoprenoids | Isoprenoids | [Nonmevalonate Branch of Isoprenoid Biosynthesis](https://rast.nmpdr.org/seedviewer.cgi?page=Subsystems&subsystem=Nonmevalonate_Branch_of_Isoprenoid_Biosynthesis&organism=549.408) | [1-deoxy-D-xylulose 5-phosphate reductoisomerase (EC 1.1.1.267)](https://rast.nmpdr.org/seedviewer.cgi?page=FunctionalRole&role=1-deoxy-D-xylulose%205-phosphate%20reductoisomerase%20(EC%201.1.1.267)&subsystem_name=Nonmevalonate_Branch_of_Isoprenoid_Biosynthesis) |
| Fatty Acids, Lipids, and Isoprenoids | Isoprenoids | [Nonmevalonate Branch of Isoprenoid Biosynthesis](https://rast.nmpdr.org/seedviewer.cgi?page=Subsystems&subsystem=Nonmevalonate_Branch_of_Isoprenoid_Biosynthesis&organism=549.408) | [4-hydroxy-3-methylbut-2-enyl diphosphate reductase (EC 1.17.1.2)](https://rast.nmpdr.org/seedviewer.cgi?page=FunctionalRole&role=4-hydroxy-3-methylbut-2-enyl%20diphosphate%20reductase%20(EC%201.17.1.2)&subsystem_name=Nonmevalonate_Branch_of_Isoprenoid_Biosynthesis) |
| Fatty Acids, Lipids, and Isoprenoids | Isoprenoids | [Nonmevalonate Branch of Isoprenoid Biosynthesis](https://rast.nmpdr.org/seedviewer.cgi?page=Subsystems&subsystem=Nonmevalonate_Branch_of_Isoprenoid_Biosynthesis&organism=549.408) | [1-hydroxy-2-methyl-2-(E)-butenyl 4-diphosphate synthase (EC 1.17.7.1)](https://rast.nmpdr.org/seedviewer.cgi?page=FunctionalRole&role=1-hydroxy-2-methyl-2-(E)-butenyl%204-diphosphate%20synthase%20(EC%201.17.7.1)&subsystem_name=Nonmevalonate_Branch_of_Isoprenoid_Biosynthesis) |
| Fatty Acids, Lipids, and Isoprenoids | Isoprenoids | [Nonmevalonate Branch of Isoprenoid Biosynthesis](https://rast.nmpdr.org/seedviewer.cgi?page=Subsystems&subsystem=Nonmevalonate_Branch_of_Isoprenoid_Biosynthesis&organism=549.408) | [4-diphosphocytidyl-2-C-methyl-D-erythritol kinase (EC 2.7.1.148)](https://rast.nmpdr.org/seedviewer.cgi?page=FunctionalRole&role=4-diphosphocytidyl-2-C-methyl-D-erythritol%20kinase%20(EC%202.7.1.148)&subsystem_name=Nonmevalonate_Branch_of_Isoprenoid_Biosynthesis) |
| Fatty Acids, Lipids, and Isoprenoids | Isoprenoids | [Nonmevalonate Branch of Isoprenoid Biosynthesis](https://rast.nmpdr.org/seedviewer.cgi?page=Subsystems&subsystem=Nonmevalonate_Branch_of_Isoprenoid_Biosynthesis&organism=549.408) | [2-C-methyl-D-erythritol 2,4-cyclodiphosphate synthase (EC 4.6.1.12)](https://rast.nmpdr.org/seedviewer.cgi?page=FunctionalRole&role=2-C-methyl-D-erythritol%202,4-cyclodiphosphate%20synthase%20(EC%204.6.1.12)&subsystem_name=Nonmevalonate_Branch_of_Isoprenoid_Biosynthesis) |
| Fatty Acids, Lipids, and Isoprenoids | Isoprenoids | [Nonmevalonate Branch of Isoprenoid Biosynthesis](https://rast.nmpdr.org/seedviewer.cgi?page=Subsystems&subsystem=Nonmevalonate_Branch_of_Isoprenoid_Biosynthesis&organism=549.408) | [2-C-methyl-D-erythritol 4-phosphate cytidylyltransferase (EC 2.7.7.60)](https://rast.nmpdr.org/seedviewer.cgi?page=FunctionalRole&role=2-C-methyl-D-erythritol%204-phosphate%20cytidylyltransferase%20(EC%202.7.7.60)&subsystem_name=Nonmevalonate_Branch_of_Isoprenoid_Biosynthesis) |
| Fatty Acids, Lipids, and Isoprenoids | Isoprenoids | [Nonmevalonate Branch of Isoprenoid Biosynthesis](https://rast.nmpdr.org/seedviewer.cgi?page=Subsystems&subsystem=Nonmevalonate_Branch_of_Isoprenoid_Biosynthesis&organism=549.408) | [1-deoxy-D-xylulose 5-phosphate synthase (EC 2.2.1.7)](https://rast.nmpdr.org/seedviewer.cgi?page=FunctionalRole&role=1-deoxy-D-xylulose%205-phosphate%20synthase%20(EC%202.2.1.7)&subsystem_name=Nonmevalonate_Branch_of_Isoprenoid_Biosynthesis) |
| Fatty Acids, Lipids, and Isoprenoids | Isoprenoids | [Isoprenoid Biosynthesis: Interconversions](https://rast.nmpdr.org/seedviewer.cgi?page=Subsystems&subsystem=Isoprenoid_Biosynthesis:_Interconversions&organism=549.408) | [Isopentenyl-diphosphate delta-isomerase (EC 5.3.3.2)](https://rast.nmpdr.org/seedviewer.cgi?page=FunctionalRole&role=Isopentenyl-diphosphate%20delta-isomerase%20(EC%205.3.3.2)&subsystem_name=Isoprenoid_Biosynthesis:_Interconversions) |
| Fatty Acids, Lipids, and Isoprenoids | Isoprenoids | [Isoprenoid Biosynthesis: Interconversions](https://rast.nmpdr.org/seedviewer.cgi?page=Subsystems&subsystem=Isoprenoid_Biosynthesis:_Interconversions&organism=549.408) | [(2E,6E)-farnesyl diphosphate synthase (EC 2.5.1.10)](https://rast.nmpdr.org/seedviewer.cgi?page=FunctionalRole&role=(2E,6E)-farnesyl%20diphosphate%20synthase%20(EC%202.5.1.10)&subsystem_name=Isoprenoid_Biosynthesis:_Interconversions) |
| Nitrogen Metabolism | Nitrogen Metabolism - no subcategory | [Nitrosative stress](https://rast.nmpdr.org/seedviewer.cgi?page=Subsystems&subsystem=Nitrosative_stress&organism=549.408) | [Nitrite-sensitive transcriptional repressor NsrR](https://rast.nmpdr.org/seedviewer.cgi?page=FunctionalRole&role=Nitrite-sensitive%20transcriptional%20repressor%20NsrR&subsystem_name=Nitrosative_stress) |
| Nitrogen Metabolism | Nitrogen Metabolism - no subcategory | [Nitrosative stress](https://rast.nmpdr.org/seedviewer.cgi?page=Subsystems&subsystem=Nitrosative_stress&organism=549.408) | [Anaerobic nitric oxide reductase flavorubredoxin](https://rast.nmpdr.org/seedviewer.cgi?page=FunctionalRole&role=Anaerobic%20nitric%20oxide%20reductase%20flavorubredoxin&subsystem_name=Nitrosative_stress) |
| Nitrogen Metabolism | Nitrogen Metabolism - no subcategory | [Nitrosative stress](https://rast.nmpdr.org/seedviewer.cgi?page=Subsystems&subsystem=Nitrosative_stress&organism=549.408) | [Nitric oxide reductase FlRd-NAD(+) reductase (EC 1.18.1.-)](https://rast.nmpdr.org/seedviewer.cgi?page=FunctionalRole&role=Nitric%20oxide%20reductase%20FlRd-NAD(+)%20reductase%20(EC%201.18.1.-)&subsystem_name=Nitrosative_stress) |
| Nitrogen Metabolism | Nitrogen Metabolism - no subcategory | [Nitrate and nitrite ammonification](https://rast.nmpdr.org/seedviewer.cgi?page=Subsystems&subsystem=Nitrate_and_nitrite_ammonification&organism=549.408) | [Nitrate ABC transporter, nitrate-binding protein](https://rast.nmpdr.org/seedviewer.cgi?page=FunctionalRole&role=Nitrate%20ABC%20transporter,%20nitrate-binding%20protein&subsystem_name=Nitrate_and_nitrite_ammonification) |
| Nitrogen Metabolism | Nitrogen Metabolism - no subcategory | [Nitrate and nitrite ammonification](https://rast.nmpdr.org/seedviewer.cgi?page=Subsystems&subsystem=Nitrate_and_nitrite_ammonification&organism=549.408) | [Respiratory nitrate reductase alpha chain (EC 1.7.99.4)](https://rast.nmpdr.org/seedviewer.cgi?page=FunctionalRole&role=Respiratory%20nitrate%20reductase%20alpha%20chain%20(EC%201.7.99.4)&subsystem_name=Nitrate_and_nitrite_ammonification) |
| Nitrogen Metabolism | Nitrogen Metabolism - no subcategory | [Nitrate and nitrite ammonification](https://rast.nmpdr.org/seedviewer.cgi?page=Subsystems&subsystem=Nitrate_and_nitrite_ammonification&organism=549.408) | [Response regulator NasT](https://rast.nmpdr.org/seedviewer.cgi?page=FunctionalRole&role=Response%20regulator%20NasT&subsystem_name=Nitrate_and_nitrite_ammonification) |
| Nitrogen Metabolism | Nitrogen Metabolism - no subcategory | [Nitrate and nitrite ammonification](https://rast.nmpdr.org/seedviewer.cgi?page=Subsystems&subsystem=Nitrate_and_nitrite_ammonification&organism=549.408) | [Nitrate/nitrite transporter](https://rast.nmpdr.org/seedviewer.cgi?page=FunctionalRole&role=Nitrate/nitrite%20transporter&subsystem_name=Nitrate_and_nitrite_ammonification) |
| Nitrogen Metabolism | Nitrogen Metabolism - no subcategory | [Nitrate and nitrite ammonification](https://rast.nmpdr.org/seedviewer.cgi?page=Subsystems&subsystem=Nitrate_and_nitrite_ammonification&organism=549.408) | [Assimilatory nitrate reductase large subunit (EC:1.7.99.4)](https://rast.nmpdr.org/seedviewer.cgi?page=FunctionalRole&role=Assimilatory%20nitrate%20reductase%20large%20subunit%20(EC:1.7.99.4)&subsystem_name=Nitrate_and_nitrite_ammonification) |
| Nitrogen Metabolism | Nitrogen Metabolism - no subcategory | [Nitrate and nitrite ammonification](https://rast.nmpdr.org/seedviewer.cgi?page=Subsystems&subsystem=Nitrate_and_nitrite_ammonification&organism=549.408) | [Nitrate ABC transporter, ATP-binding protein](https://rast.nmpdr.org/seedviewer.cgi?page=FunctionalRole&role=Nitrate%20ABC%20transporter,%20ATP-binding%20protein&subsystem_name=Nitrate_and_nitrite_ammonification) |
| Nitrogen Metabolism | Nitrogen Metabolism - no subcategory | [Nitrate and nitrite ammonification](https://rast.nmpdr.org/seedviewer.cgi?page=Subsystems&subsystem=Nitrate_and_nitrite_ammonification&organism=549.408) | [Respiratory nitrate reductase gamma chain (EC 1.7.99.4)](https://rast.nmpdr.org/seedviewer.cgi?page=FunctionalRole&role=Respiratory%20nitrate%20reductase%20gamma%20chain%20(EC%201.7.99.4)&subsystem_name=Nitrate_and_nitrite_ammonification) |
| Nitrogen Metabolism | Nitrogen Metabolism - no subcategory | [Nitrate and nitrite ammonification](https://rast.nmpdr.org/seedviewer.cgi?page=Subsystems&subsystem=Nitrate_and_nitrite_ammonification&organism=549.408) | [Respiratory nitrate reductase delta chain (EC 1.7.99.4)](https://rast.nmpdr.org/seedviewer.cgi?page=FunctionalRole&role=Respiratory%20nitrate%20reductase%20delta%20chain%20(EC%201.7.99.4)&subsystem_name=Nitrate_and_nitrite_ammonification) |
| Nitrogen Metabolism | Nitrogen Metabolism - no subcategory | [Nitrate and nitrite ammonification](https://rast.nmpdr.org/seedviewer.cgi?page=Subsystems&subsystem=Nitrate_and_nitrite_ammonification&organism=549.408) | [Respiratory nitrate reductase beta chain (EC 1.7.99.4)](https://rast.nmpdr.org/seedviewer.cgi?page=FunctionalRole&role=Respiratory%20nitrate%20reductase%20beta%20chain%20(EC%201.7.99.4)&subsystem_name=Nitrate_and_nitrite_ammonification) |
| Nitrogen Metabolism | Nitrogen Metabolism - no subcategory | [Nitrate and nitrite ammonification](https://rast.nmpdr.org/seedviewer.cgi?page=Subsystems&subsystem=Nitrate_and_nitrite_ammonification&organism=549.408) | [Nitrate/nitrite sensor protein (EC 2.7.3.-)](https://rast.nmpdr.org/seedviewer.cgi?page=FunctionalRole&role=Nitrate/nitrite%20sensor%20protein%20(EC%202.7.3.-)&subsystem_name=Nitrate_and_nitrite_ammonification) |
| Nitrogen Metabolism | Nitrogen Metabolism - no subcategory | [Nitrate and nitrite ammonification](https://rast.nmpdr.org/seedviewer.cgi?page=Subsystems&subsystem=Nitrate_and_nitrite_ammonification&organism=549.408) | [Nitrate/nitrite response regulator protein](https://rast.nmpdr.org/seedviewer.cgi?page=FunctionalRole&role=Nitrate/nitrite%20response%20regulator%20protein&subsystem_name=Nitrate_and_nitrite_ammonification) |
| Nitrogen Metabolism | Nitrogen Metabolism - no subcategory | [Nitrate and nitrite ammonification](https://rast.nmpdr.org/seedviewer.cgi?page=Subsystems&subsystem=Nitrate_and_nitrite_ammonification&organism=549.408) | [Nitrite reductase [NAD(P)H] large subunit (EC 1.7.1.4)](https://rast.nmpdr.org/seedviewer.cgi?page=FunctionalRole&role=Nitrite%20reductase%20%5bNAD(P)H%5d%20large%20subunit%20(EC%201.7.1.4)&subsystem_name=Nitrate_and_nitrite_ammonification) |
| Nitrogen Metabolism | Nitrogen Metabolism - no subcategory | [Nitrate and nitrite ammonification](https://rast.nmpdr.org/seedviewer.cgi?page=Subsystems&subsystem=Nitrate_and_nitrite_ammonification&organism=549.408) | [Nitrite reductase [NAD(P)H] small subunit (EC 1.7.1.4)](https://rast.nmpdr.org/seedviewer.cgi?page=FunctionalRole&role=Nitrite%20reductase%20%5bNAD(P)H%5d%20small%20subunit%20(EC%201.7.1.4)&subsystem_name=Nitrate_and_nitrite_ammonification) |
| Nitrogen Metabolism | Nitrogen Metabolism - no subcategory | [Ammonia assimilation](https://rast.nmpdr.org/seedviewer.cgi?page=Subsystems&subsystem=Ammonia_assimilation&organism=549.408) | [Nitrogen regulatory protein P-II, glnK](https://rast.nmpdr.org/seedviewer.cgi?page=FunctionalRole&role=Nitrogen%20regulatory%20protein%20P-II,%20glnK&subsystem_name=Ammonia_assimilation) |
| Nitrogen Metabolism | Nitrogen Metabolism - no subcategory | [Ammonia assimilation](https://rast.nmpdr.org/seedviewer.cgi?page=Subsystems&subsystem=Ammonia_assimilation&organism=549.408) | [Nitrogen regulatory protein P-II](https://rast.nmpdr.org/seedviewer.cgi?page=FunctionalRole&role=Nitrogen%20regulatory%20protein%20P-II&subsystem_name=Ammonia_assimilation) |
| Nitrogen Metabolism | Nitrogen Metabolism - no subcategory | [Ammonia assimilation](https://rast.nmpdr.org/seedviewer.cgi?page=Subsystems&subsystem=Ammonia_assimilation&organism=549.408) | [Nitrogen regulation protein NR(I)](https://rast.nmpdr.org/seedviewer.cgi?page=FunctionalRole&role=Nitrogen%20regulation%20protein%20NR(I)&subsystem_name=Ammonia_assimilation) |
| Nitrogen Metabolism | Nitrogen Metabolism - no subcategory | [Ammonia assimilation](https://rast.nmpdr.org/seedviewer.cgi?page=Subsystems&subsystem=Ammonia_assimilation&organism=549.408) | [Glutamate-ammonia-ligase adenylyltransferase (EC 2.7.7.42)](https://rast.nmpdr.org/seedviewer.cgi?page=FunctionalRole&role=Glutamate-ammonia-ligase%20adenylyltransferase%20(EC%202.7.7.42)&subsystem_name=Ammonia_assimilation) |
| Nitrogen Metabolism | Nitrogen Metabolism - no subcategory | [Ammonia assimilation](https://rast.nmpdr.org/seedviewer.cgi?page=Subsystems&subsystem=Ammonia_assimilation&organism=549.408) | [Ammonium transporter](https://rast.nmpdr.org/seedviewer.cgi?page=FunctionalRole&role=Ammonium%20transporter&subsystem_name=Ammonia_assimilation) |
| Nitrogen Metabolism | Nitrogen Metabolism - no subcategory | [Ammonia assimilation](https://rast.nmpdr.org/seedviewer.cgi?page=Subsystems&subsystem=Ammonia_assimilation&organism=549.408) | [Glutamate synthase [NADPH] large chain (EC 1.4.1.13)](https://rast.nmpdr.org/seedviewer.cgi?page=FunctionalRole&role=Glutamate%20synthase%20%5bNADPH%5d%20large%20chain%20(EC%201.4.1.13)&subsystem_name=Ammonia_assimilation) |
| Nitrogen Metabolism | Nitrogen Metabolism - no subcategory | [Ammonia assimilation](https://rast.nmpdr.org/seedviewer.cgi?page=Subsystems&subsystem=Ammonia_assimilation&organism=549.408) | [[Protein-PII] uridylyltransferase (EC 2.7.7.59)](https://rast.nmpdr.org/seedviewer.cgi?page=FunctionalRole&role=%5bProtein-PII%5d%20uridylyltransferase%20(EC%202.7.7.59)&subsystem_name=Ammonia_assimilation) |
| Nitrogen Metabolism | Nitrogen Metabolism - no subcategory | [Ammonia assimilation](https://rast.nmpdr.org/seedviewer.cgi?page=Subsystems&subsystem=Ammonia_assimilation&organism=549.408) | [Glutamine synthetase type I (EC 6.3.1.2)](https://rast.nmpdr.org/seedviewer.cgi?page=FunctionalRole&role=Glutamine%20synthetase%20type%20I%20(EC%206.3.1.2)&subsystem_name=Ammonia_assimilation) |
| Nitrogen Metabolism | Nitrogen Metabolism - no subcategory | [Ammonia assimilation](https://rast.nmpdr.org/seedviewer.cgi?page=Subsystems&subsystem=Ammonia_assimilation&organism=549.408) | [Glutamate synthase [NADPH] small chain (EC 1.4.1.13)](https://rast.nmpdr.org/seedviewer.cgi?page=FunctionalRole&role=Glutamate%20synthase%20%5bNADPH%5d%20small%20chain%20(EC%201.4.1.13)&subsystem_name=Ammonia_assimilation) |
| Nitrogen Metabolism | Denitrification | [Denitrifying reductase gene clusters](https://rast.nmpdr.org/seedviewer.cgi?page=Subsystems&subsystem=Denitrifying_reductase_gene_clusters&organism=549.408) | [Respiratory nitrate reductase alpha chain (EC 1.7.99.4)](https://rast.nmpdr.org/seedviewer.cgi?page=FunctionalRole&role=Respiratory%20nitrate%20reductase%20alpha%20chain%20(EC%201.7.99.4)&subsystem_name=Denitrifying_reductase_gene_clusters) |
| Nitrogen Metabolism | Denitrification | [Denitrifying reductase gene clusters](https://rast.nmpdr.org/seedviewer.cgi?page=Subsystems&subsystem=Denitrifying_reductase_gene_clusters&organism=549.408) | [Respiratory nitrate reductase gamma chain (EC 1.7.99.4)](https://rast.nmpdr.org/seedviewer.cgi?page=FunctionalRole&role=Respiratory%20nitrate%20reductase%20gamma%20chain%20(EC%201.7.99.4)&subsystem_name=Denitrifying_reductase_gene_clusters) |
| Nitrogen Metabolism | Denitrification | [Denitrifying reductase gene clusters](https://rast.nmpdr.org/seedviewer.cgi?page=Subsystems&subsystem=Denitrifying_reductase_gene_clusters&organism=549.408) | [Respiratory nitrate reductase delta chain (EC 1.7.99.4)](https://rast.nmpdr.org/seedviewer.cgi?page=FunctionalRole&role=Respiratory%20nitrate%20reductase%20delta%20chain%20(EC%201.7.99.4)&subsystem_name=Denitrifying_reductase_gene_clusters) |
| Nitrogen Metabolism | Denitrification | [Denitrifying reductase gene clusters](https://rast.nmpdr.org/seedviewer.cgi?page=Subsystems&subsystem=Denitrifying_reductase_gene_clusters&organism=549.408) | [Respiratory nitrate reductase beta chain (EC 1.7.99.4)](https://rast.nmpdr.org/seedviewer.cgi?page=FunctionalRole&role=Respiratory%20nitrate%20reductase%20beta%20chain%20(EC%201.7.99.4)&subsystem_name=Denitrifying_reductase_gene_clusters) |
| Dormancy and Sporulation | Dormancy and Sporulation - no subcategory | [Persister Cells](https://rast.nmpdr.org/seedviewer.cgi?page=Subsystems&subsystem=Persister_Cells&organism=549.408) | [HipA protein](https://rast.nmpdr.org/seedviewer.cgi?page=FunctionalRole&role=HipA%20protein&subsystem_name=Persister_Cells) |
| Dormancy and Sporulation | Dormancy and Sporulation - no subcategory | [Persister Cells](https://rast.nmpdr.org/seedviewer.cgi?page=Subsystems&subsystem=Persister_Cells&organism=549.408) | [Cell division inhibitor](https://rast.nmpdr.org/seedviewer.cgi?page=FunctionalRole&role=Cell%20division%20inhibitor&subsystem_name=Persister_Cells) |
| Dormancy and Sporulation | Dormancy and Sporulation - no subcategory | [Persister Cells](https://rast.nmpdr.org/seedviewer.cgi?page=Subsystems&subsystem=Persister_Cells&organism=549.408) | [HipB protein](https://rast.nmpdr.org/seedviewer.cgi?page=FunctionalRole&role=HipB%20protein&subsystem_name=Persister_Cells) |
| Dormancy and Sporulation | Dormancy and Sporulation - no subcategory | [Sporulation-associated proteins with broader functions](https://rast.nmpdr.org/seedviewer.cgi?page=Subsystems&subsystem=Sporulation-associated_proteins_with_broader_functions&organism=549.408) | [Peptidyl-tRNA hydrolase (EC 3.1.1.29)](https://rast.nmpdr.org/seedviewer.cgi?page=FunctionalRole&role=Peptidyl-tRNA%20hydrolase%20(EC%203.1.1.29)&subsystem_name=Sporulation-associated_proteins_with_broader_functions) |
| Respiration | Electron accepting reactions | [Terminal cytochrome d ubiquinol oxidases](https://rast.nmpdr.org/seedviewer.cgi?page=Subsystems&subsystem=Terminal_cytochrome_d_ubiquinol_oxidases&organism=549.408) | [Cytochrome d ubiquinol oxidase subunit I (EC 1.10.3.-)](https://rast.nmpdr.org/seedviewer.cgi?page=FunctionalRole&role=Cytochrome%20d%20ubiquinol%20oxidase%20subunit%20I%20(EC%201.10.3.-)&subsystem_name=Terminal_cytochrome_d_ubiquinol_oxidases) |
| Respiration | Electron accepting reactions | [Terminal cytochrome d ubiquinol oxidases](https://rast.nmpdr.org/seedviewer.cgi?page=Subsystems&subsystem=Terminal_cytochrome_d_ubiquinol_oxidases&organism=549.408) | [Cytochrome d ubiquinol oxidase subunit II (EC 1.10.3.-)](https://rast.nmpdr.org/seedviewer.cgi?page=FunctionalRole&role=Cytochrome%20d%20ubiquinol%20oxidase%20subunit%20II%20(EC%201.10.3.-)&subsystem_name=Terminal_cytochrome_d_ubiquinol_oxidases) |
| Respiration | Electron accepting reactions | [Terminal cytochrome d ubiquinol oxidases](https://rast.nmpdr.org/seedviewer.cgi?page=Subsystems&subsystem=Terminal_cytochrome_d_ubiquinol_oxidases&organism=549.408) | [Transport ATP-binding protein CydD](https://rast.nmpdr.org/seedviewer.cgi?page=FunctionalRole&role=Transport%20ATP-binding%20protein%20CydD&subsystem_name=Terminal_cytochrome_d_ubiquinol_oxidases) |
| Respiration | Electron accepting reactions | [Terminal cytochrome d ubiquinol oxidases](https://rast.nmpdr.org/seedviewer.cgi?page=Subsystems&subsystem=Terminal_cytochrome_d_ubiquinol_oxidases&organism=549.408) | [putative Cytochrome bd2, subunit I](https://rast.nmpdr.org/seedviewer.cgi?page=FunctionalRole&role=putative%20Cytochrome%20bd2,%20subunit%20I&subsystem_name=Terminal_cytochrome_d_ubiquinol_oxidases) |
| Respiration | Electron accepting reactions | [Terminal cytochrome d ubiquinol oxidases](https://rast.nmpdr.org/seedviewer.cgi?page=Subsystems&subsystem=Terminal_cytochrome_d_ubiquinol_oxidases&organism=549.408) | [putative Cytochrome bd2, subunit II](https://rast.nmpdr.org/seedviewer.cgi?page=FunctionalRole&role=putative%20Cytochrome%20bd2,%20subunit%20II&subsystem_name=Terminal_cytochrome_d_ubiquinol_oxidases) |
| Respiration | Electron accepting reactions | [Terminal cytochrome d ubiquinol oxidases](https://rast.nmpdr.org/seedviewer.cgi?page=Subsystems&subsystem=Terminal_cytochrome_d_ubiquinol_oxidases&organism=549.408) | [Transport ATP-binding protein CydC](https://rast.nmpdr.org/seedviewer.cgi?page=FunctionalRole&role=Transport%20ATP-binding%20protein%20CydC&subsystem_name=Terminal_cytochrome_d_ubiquinol_oxidases) |
| Respiration | Electron accepting reactions | [Terminal cytochrome O ubiquinol oxidase](https://rast.nmpdr.org/seedviewer.cgi?page=Subsystems&subsystem=Terminal_cytochrome_O_ubiquinol_oxidase&organism=549.408) | [Cytochrome O ubiquinol oxidase subunit III (EC 1.10.3.-)](https://rast.nmpdr.org/seedviewer.cgi?page=FunctionalRole&role=Cytochrome%20O%20ubiquinol%20oxidase%20subunit%20III%20(EC%201.10.3.-)&subsystem_name=Terminal_cytochrome_O_ubiquinol_oxidase) |
| Respiration | Electron accepting reactions | [Terminal cytochrome O ubiquinol oxidase](https://rast.nmpdr.org/seedviewer.cgi?page=Subsystems&subsystem=Terminal_cytochrome_O_ubiquinol_oxidase&organism=549.408) | [Cytochrome O ubiquinol oxidase subunit IV (EC 1.10.3.-)](https://rast.nmpdr.org/seedviewer.cgi?page=FunctionalRole&role=Cytochrome%20O%20ubiquinol%20oxidase%20subunit%20IV%20(EC%201.10.3.-)&subsystem_name=Terminal_cytochrome_O_ubiquinol_oxidase) |
| Respiration | Electron accepting reactions | [Terminal cytochrome O ubiquinol oxidase](https://rast.nmpdr.org/seedviewer.cgi?page=Subsystems&subsystem=Terminal_cytochrome_O_ubiquinol_oxidase&organism=549.408) | [Cytochrome O ubiquinol oxidase subunit I (EC 1.10.3.-)](https://rast.nmpdr.org/seedviewer.cgi?page=FunctionalRole&role=Cytochrome%20O%20ubiquinol%20oxidase%20subunit%20I%20(EC%201.10.3.-)&subsystem_name=Terminal_cytochrome_O_ubiquinol_oxidase) |
| Respiration | Electron accepting reactions | [Terminal cytochrome O ubiquinol oxidase](https://rast.nmpdr.org/seedviewer.cgi?page=Subsystems&subsystem=Terminal_cytochrome_O_ubiquinol_oxidase&organism=549.408) | [Cytochrome O ubiquinol oxidase subunit II (EC 1.10.3.-)](https://rast.nmpdr.org/seedviewer.cgi?page=FunctionalRole&role=Cytochrome%20O%20ubiquinol%20oxidase%20subunit%20II%20(EC%201.10.3.-)&subsystem_name=Terminal_cytochrome_O_ubiquinol_oxidase) |
| Respiration | Electron accepting reactions | [Fumarate respiration cluster](https://rast.nmpdr.org/seedviewer.cgi?page=Subsystems&subsystem=Fumarate_respiration_cluster&organism=549.408) | [Fumarate respiration transcriptional regulator DcuR](https://rast.nmpdr.org/seedviewer.cgi?page=FunctionalRole&role=Fumarate%20respiration%20transcriptional%20regulator%20DcuR&subsystem_name=Fumarate_respiration_cluster) |
| Respiration | Electron accepting reactions | [Fumarate respiration cluster](https://rast.nmpdr.org/seedviewer.cgi?page=Subsystems&subsystem=Fumarate_respiration_cluster&organism=549.408) | [Fumarate respiration sensor kinase protein DcuS](https://rast.nmpdr.org/seedviewer.cgi?page=FunctionalRole&role=Fumarate%20respiration%20sensor%20kinase%20protein%20DcuS&subsystem_name=Fumarate_respiration_cluster) |
| Respiration | Electron accepting reactions | [Anaerobic respiratory reductases](https://rast.nmpdr.org/seedviewer.cgi?page=Subsystems&subsystem=Anaerobic_respiratory_reductases&organism=549.408) | [Ferric reductase (1.6.99.14)](https://rast.nmpdr.org/seedviewer.cgi?page=FunctionalRole&role=Ferric%20reductase%20(1.6.99.14)&subsystem_name=Anaerobic_respiratory_reductases) |
| Respiration | Electron accepting reactions | [Anaerobic respiratory reductases](https://rast.nmpdr.org/seedviewer.cgi?page=Subsystems&subsystem=Anaerobic_respiratory_reductases&organism=549.408) | [Formate dehydrogenase -O, gamma subunit (EC 1.2.1.2)](https://rast.nmpdr.org/seedviewer.cgi?page=FunctionalRole&role=Formate%20dehydrogenase%20-O,%20gamma%20subunit%20(EC%201.2.1.2)&subsystem_name=Anaerobic_respiratory_reductases) |
| Respiration | Electron accepting reactions | [Anaerobic respiratory reductases](https://rast.nmpdr.org/seedviewer.cgi?page=Subsystems&subsystem=Anaerobic_respiratory_reductases&organism=549.408) | [Flavodoxin reductases (ferredoxin-NADPH reductases) family 1](https://rast.nmpdr.org/seedviewer.cgi?page=FunctionalRole&role=Flavodoxin%20reductases%20(ferredoxin-NADPH%20reductases)%20family%201&subsystem_name=Anaerobic_respiratory_reductases) |
| Respiration | Electron accepting reactions | [Anaerobic respiratory reductases](https://rast.nmpdr.org/seedviewer.cgi?page=Subsystems&subsystem=Anaerobic_respiratory_reductases&organism=549.408) | [Ferredoxin reductase](https://rast.nmpdr.org/seedviewer.cgi?page=FunctionalRole&role=Ferredoxin%20reductase&subsystem_name=Anaerobic_respiratory_reductases) |
| Respiration | Electron accepting reactions | [Anaerobic respiratory reductases](https://rast.nmpdr.org/seedviewer.cgi?page=Subsystems&subsystem=Anaerobic_respiratory_reductases&organism=549.408) | [Arsenate reductase (EC 1.20.4.1)](https://rast.nmpdr.org/seedviewer.cgi?page=FunctionalRole&role=Arsenate%20reductase%20(EC%201.20.4.1)&subsystem_name=Anaerobic_respiratory_reductases) |
| Respiration | Electron accepting reactions | [Terminal cytochrome oxidases](https://rast.nmpdr.org/seedviewer.cgi?page=Subsystems&subsystem=Terminal_cytochrome_oxidases&organism=549.408) | [Cytochrome d ubiquinol oxidase subunit I (EC 1.10.3.-)](https://rast.nmpdr.org/seedviewer.cgi?page=FunctionalRole&role=Cytochrome%20d%20ubiquinol%20oxidase%20subunit%20I%20(EC%201.10.3.-)&subsystem_name=Terminal_cytochrome_oxidases) |
| Respiration | Electron accepting reactions | [Terminal cytochrome oxidases](https://rast.nmpdr.org/seedviewer.cgi?page=Subsystems&subsystem=Terminal_cytochrome_oxidases&organism=549.408) | [Cytochrome d ubiquinol oxidase subunit II (EC 1.10.3.-)](https://rast.nmpdr.org/seedviewer.cgi?page=FunctionalRole&role=Cytochrome%20d%20ubiquinol%20oxidase%20subunit%20II%20(EC%201.10.3.-)&subsystem_name=Terminal_cytochrome_oxidases) |
| Respiration | Electron accepting reactions | [Terminal cytochrome oxidases](https://rast.nmpdr.org/seedviewer.cgi?page=Subsystems&subsystem=Terminal_cytochrome_oxidases&organism=549.408) | [Cytochrome O ubiquinol oxidase subunit IV (EC 1.10.3.-)](https://rast.nmpdr.org/seedviewer.cgi?page=FunctionalRole&role=Cytochrome%20O%20ubiquinol%20oxidase%20subunit%20IV%20(EC%201.10.3.-)&subsystem_name=Terminal_cytochrome_oxidases) |
| Respiration | Electron accepting reactions | [Terminal cytochrome oxidases](https://rast.nmpdr.org/seedviewer.cgi?page=Subsystems&subsystem=Terminal_cytochrome_oxidases&organism=549.408) | [Cytochrome O ubiquinol oxidase subunit I (EC 1.10.3.-)](https://rast.nmpdr.org/seedviewer.cgi?page=FunctionalRole&role=Cytochrome%20O%20ubiquinol%20oxidase%20subunit%20I%20(EC%201.10.3.-)&subsystem_name=Terminal_cytochrome_oxidases) |
| Respiration | Electron accepting reactions | [Terminal cytochrome oxidases](https://rast.nmpdr.org/seedviewer.cgi?page=Subsystems&subsystem=Terminal_cytochrome_oxidases&organism=549.408) | [Transport ATP-binding protein CydC](https://rast.nmpdr.org/seedviewer.cgi?page=FunctionalRole&role=Transport%20ATP-binding%20protein%20CydC&subsystem_name=Terminal_cytochrome_oxidases) |
| Respiration | Electron accepting reactions | [Terminal cytochrome oxidases](https://rast.nmpdr.org/seedviewer.cgi?page=Subsystems&subsystem=Terminal_cytochrome_oxidases&organism=549.408) | [Cytochrome O ubiquinol oxidase subunit II (EC 1.10.3.-)](https://rast.nmpdr.org/seedviewer.cgi?page=FunctionalRole&role=Cytochrome%20O%20ubiquinol%20oxidase%20subunit%20II%20(EC%201.10.3.-)&subsystem_name=Terminal_cytochrome_oxidases) |
| Respiration | Electron accepting reactions | [Terminal cytochrome oxidases](https://rast.nmpdr.org/seedviewer.cgi?page=Subsystems&subsystem=Terminal_cytochrome_oxidases&organism=549.408) | [Cytochrome O ubiquinol oxidase subunit III (EC 1.10.3.-)](https://rast.nmpdr.org/seedviewer.cgi?page=FunctionalRole&role=Cytochrome%20O%20ubiquinol%20oxidase%20subunit%20III%20(EC%201.10.3.-)&subsystem_name=Terminal_cytochrome_oxidases) |
| Respiration | Electron accepting reactions | [Terminal cytochrome oxidases](https://rast.nmpdr.org/seedviewer.cgi?page=Subsystems&subsystem=Terminal_cytochrome_oxidases&organism=549.408) | [Transport ATP-binding protein CydD](https://rast.nmpdr.org/seedviewer.cgi?page=FunctionalRole&role=Transport%20ATP-binding%20protein%20CydD&subsystem_name=Terminal_cytochrome_oxidases) |
| Respiration | Electron accepting reactions | [Terminal cytochrome oxidases](https://rast.nmpdr.org/seedviewer.cgi?page=Subsystems&subsystem=Terminal_cytochrome_oxidases&organism=549.408) | [putative Cytochrome bd2, subunit I](https://rast.nmpdr.org/seedviewer.cgi?page=FunctionalRole&role=putative%20Cytochrome%20bd2,%20subunit%20I&subsystem_name=Terminal_cytochrome_oxidases) |
| Respiration | Electron accepting reactions | [Terminal cytochrome oxidases](https://rast.nmpdr.org/seedviewer.cgi?page=Subsystems&subsystem=Terminal_cytochrome_oxidases&organism=549.408) | [putative Cytochrome bd2, subunit II](https://rast.nmpdr.org/seedviewer.cgi?page=FunctionalRole&role=putative%20Cytochrome%20bd2,%20subunit%20II&subsystem_name=Terminal_cytochrome_oxidases) |
| Respiration | Electron donating reactions | [Na(+)-translocating NADH-quinone oxidoreductase and rnf-like group of electron transport complexes](https://rast.nmpdr.org/seedviewer.cgi?page=Subsystems&subsystem=Na(+)-translocating_NADH-quinone_oxidoreductase_and_rnf-like_group_of_electron_transport_complexes&organism=549.408) | [Electron transport complex protein RnfE](https://rast.nmpdr.org/seedviewer.cgi?page=FunctionalRole&role=Electron%20transport%20complex%20protein%20RnfE&subsystem_name=Na(+)-translocating_NADH-quinone_oxidoreductase_and_rnf-like_group_of_electron_transport_complexes) |
| Respiration | Electron donating reactions | [Na(+)-translocating NADH-quinone oxidoreductase and rnf-like group of electron transport complexes](https://rast.nmpdr.org/seedviewer.cgi?page=Subsystems&subsystem=Na(+)-translocating_NADH-quinone_oxidoreductase_and_rnf-like_group_of_electron_transport_complexes&organism=549.408) | [Electron transport complex protein RnfA](https://rast.nmpdr.org/seedviewer.cgi?page=FunctionalRole&role=Electron%20transport%20complex%20protein%20RnfA&subsystem_name=Na(+)-translocating_NADH-quinone_oxidoreductase_and_rnf-like_group_of_electron_transport_complexes) |
| Respiration | Electron donating reactions | [Na(+)-translocating NADH-quinone oxidoreductase and rnf-like group of electron transport complexes](https://rast.nmpdr.org/seedviewer.cgi?page=Subsystems&subsystem=Na(+)-translocating_NADH-quinone_oxidoreductase_and_rnf-like_group_of_electron_transport_complexes&organism=549.408) | [Electron transport complex protein RnfG](https://rast.nmpdr.org/seedviewer.cgi?page=FunctionalRole&role=Electron%20transport%20complex%20protein%20RnfG&subsystem_name=Na(+)-translocating_NADH-quinone_oxidoreductase_and_rnf-like_group_of_electron_transport_complexes) |
| Respiration | Electron donating reactions | [Na(+)-translocating NADH-quinone oxidoreductase and rnf-like group of electron transport complexes](https://rast.nmpdr.org/seedviewer.cgi?page=Subsystems&subsystem=Na(+)-translocating_NADH-quinone_oxidoreductase_and_rnf-like_group_of_electron_transport_complexes&organism=549.408) | [Electron transport complex protein RnfD](https://rast.nmpdr.org/seedviewer.cgi?page=FunctionalRole&role=Electron%20transport%20complex%20protein%20RnfD&subsystem_name=Na(+)-translocating_NADH-quinone_oxidoreductase_and_rnf-like_group_of_electron_transport_complexes) |
| Respiration | Electron donating reactions | [Na(+)-translocating NADH-quinone oxidoreductase and rnf-like group of electron transport complexes](https://rast.nmpdr.org/seedviewer.cgi?page=Subsystems&subsystem=Na(+)-translocating_NADH-quinone_oxidoreductase_and_rnf-like_group_of_electron_transport_complexes&organism=549.408) | [Electron transport complex protein RnfB](https://rast.nmpdr.org/seedviewer.cgi?page=FunctionalRole&role=Electron%20transport%20complex%20protein%20RnfB&subsystem_name=Na(+)-translocating_NADH-quinone_oxidoreductase_and_rnf-like_group_of_electron_transport_complexes) |
| Respiration | Electron donating reactions | [Na(+)-translocating NADH-quinone oxidoreductase and rnf-like group of electron transport complexes](https://rast.nmpdr.org/seedviewer.cgi?page=Subsystems&subsystem=Na(+)-translocating_NADH-quinone_oxidoreductase_and_rnf-like_group_of_electron_transport_complexes&organism=549.408) | [Electron transport complex protein RnfC](https://rast.nmpdr.org/seedviewer.cgi?page=FunctionalRole&role=Electron%20transport%20complex%20protein%20RnfC&subsystem_name=Na(+)-translocating_NADH-quinone_oxidoreductase_and_rnf-like_group_of_electron_transport_complexes) |
| Respiration | Electron donating reactions | [Respiratory Complex I](https://rast.nmpdr.org/seedviewer.cgi?page=Subsystems&subsystem=Respiratory_Complex_I&organism=549.408) | [NADH-ubiquinone oxidoreductase chain K (EC 1.6.5.3)](https://rast.nmpdr.org/seedviewer.cgi?page=FunctionalRole&role=NADH-ubiquinone%20oxidoreductase%20chain%20K%20(EC%201.6.5.3)&subsystem_name=Respiratory_Complex_I) |
| Respiration | Electron donating reactions | [Respiratory Complex I](https://rast.nmpdr.org/seedviewer.cgi?page=Subsystems&subsystem=Respiratory_Complex_I&organism=549.408) | [NADH-ubiquinone oxidoreductase chain L (EC 1.6.5.3)](https://rast.nmpdr.org/seedviewer.cgi?page=FunctionalRole&role=NADH-ubiquinone%20oxidoreductase%20chain%20L%20(EC%201.6.5.3)&subsystem_name=Respiratory_Complex_I) |
| Respiration | Electron donating reactions | [Respiratory Complex I](https://rast.nmpdr.org/seedviewer.cgi?page=Subsystems&subsystem=Respiratory_Complex_I&organism=549.408) | [NADH-ubiquinone oxidoreductase chain J (EC 1.6.5.3)](https://rast.nmpdr.org/seedviewer.cgi?page=FunctionalRole&role=NADH-ubiquinone%20oxidoreductase%20chain%20J%20(EC%201.6.5.3)&subsystem_name=Respiratory_Complex_I) |
| Respiration | Electron donating reactions | [Respiratory Complex I](https://rast.nmpdr.org/seedviewer.cgi?page=Subsystems&subsystem=Respiratory_Complex_I&organism=549.408) | [NADH-ubiquinone oxidoreductase chain N (EC 1.6.5.3)](https://rast.nmpdr.org/seedviewer.cgi?page=FunctionalRole&role=NADH-ubiquinone%20oxidoreductase%20chain%20N%20(EC%201.6.5.3)&subsystem_name=Respiratory_Complex_I) |
| Respiration | Electron donating reactions | [Respiratory Complex I](https://rast.nmpdr.org/seedviewer.cgi?page=Subsystems&subsystem=Respiratory_Complex_I&organism=549.408) | [NADH-ubiquinone oxidoreductase chain H (EC 1.6.5.3)](https://rast.nmpdr.org/seedviewer.cgi?page=FunctionalRole&role=NADH-ubiquinone%20oxidoreductase%20chain%20H%20(EC%201.6.5.3)&subsystem_name=Respiratory_Complex_I) |
| Respiration | Electron donating reactions | [Respiratory Complex I](https://rast.nmpdr.org/seedviewer.cgi?page=Subsystems&subsystem=Respiratory_Complex_I&organism=549.408) | [NADH-ubiquinone oxidoreductase chain E (EC 1.6.5.3)](https://rast.nmpdr.org/seedviewer.cgi?page=FunctionalRole&role=NADH-ubiquinone%20oxidoreductase%20chain%20E%20(EC%201.6.5.3)&subsystem_name=Respiratory_Complex_I) |
| Respiration | Electron donating reactions | [Respiratory Complex I](https://rast.nmpdr.org/seedviewer.cgi?page=Subsystems&subsystem=Respiratory_Complex_I&organism=549.408) | [NADH-ubiquinone oxidoreductase chain B (EC 1.6.5.3)](https://rast.nmpdr.org/seedviewer.cgi?page=FunctionalRole&role=NADH-ubiquinone%20oxidoreductase%20chain%20B%20(EC%201.6.5.3)&subsystem_name=Respiratory_Complex_I) |
| Respiration | Electron donating reactions | [Respiratory Complex I](https://rast.nmpdr.org/seedviewer.cgi?page=Subsystems&subsystem=Respiratory_Complex_I&organism=549.408) | [NADH-ubiquinone oxidoreductase chain I (EC 1.6.5.3)](https://rast.nmpdr.org/seedviewer.cgi?page=FunctionalRole&role=NADH-ubiquinone%20oxidoreductase%20chain%20I%20(EC%201.6.5.3)&subsystem_name=Respiratory_Complex_I) |
| Respiration | Electron donating reactions | [Respiratory Complex I](https://rast.nmpdr.org/seedviewer.cgi?page=Subsystems&subsystem=Respiratory_Complex_I&organism=549.408) | [NADH-ubiquinone oxidoreductase chain M (EC 1.6.5.3)](https://rast.nmpdr.org/seedviewer.cgi?page=FunctionalRole&role=NADH-ubiquinone%20oxidoreductase%20chain%20M%20(EC%201.6.5.3)&subsystem_name=Respiratory_Complex_I) |
| Respiration | Electron donating reactions | [Respiratory Complex I](https://rast.nmpdr.org/seedviewer.cgi?page=Subsystems&subsystem=Respiratory_Complex_I&organism=549.408) | [NADH-ubiquinone oxidoreductase chain F (EC 1.6.5.3)](https://rast.nmpdr.org/seedviewer.cgi?page=FunctionalRole&role=NADH-ubiquinone%20oxidoreductase%20chain%20F%20(EC%201.6.5.3)&subsystem_name=Respiratory_Complex_I) |
| Respiration | Electron donating reactions | [Respiratory Complex I](https://rast.nmpdr.org/seedviewer.cgi?page=Subsystems&subsystem=Respiratory_Complex_I&organism=549.408) | [NADH-ubiquinone oxidoreductase chain G (EC 1.6.5.3)](https://rast.nmpdr.org/seedviewer.cgi?page=FunctionalRole&role=NADH-ubiquinone%20oxidoreductase%20chain%20G%20(EC%201.6.5.3)&subsystem_name=Respiratory_Complex_I) |
| Respiration | Electron donating reactions | [Respiratory Complex I](https://rast.nmpdr.org/seedviewer.cgi?page=Subsystems&subsystem=Respiratory_Complex_I&organism=549.408) | [NADH-ubiquinone oxidoreductase chain C (EC 1.6.5.3)](https://rast.nmpdr.org/seedviewer.cgi?page=FunctionalRole&role=NADH-ubiquinone%20oxidoreductase%20chain%20C%20(EC%201.6.5.3)&subsystem_name=Respiratory_Complex_I) |
| Respiration | Electron donating reactions | [Respiratory Complex I](https://rast.nmpdr.org/seedviewer.cgi?page=Subsystems&subsystem=Respiratory_Complex_I&organism=549.408) | [NADH ubiquinone oxidoreductase chain A (EC 1.6.5.3)](https://rast.nmpdr.org/seedviewer.cgi?page=FunctionalRole&role=NADH%20ubiquinone%20oxidoreductase%20chain%20A%20(EC%201.6.5.3)&subsystem_name=Respiratory_Complex_I) |
| Respiration | Electron donating reactions | [Respiratory Complex I](https://rast.nmpdr.org/seedviewer.cgi?page=Subsystems&subsystem=Respiratory_Complex_I&organism=549.408) | [NADH-ubiquinone oxidoreductase chain D (EC 1.6.5.3)](https://rast.nmpdr.org/seedviewer.cgi?page=FunctionalRole&role=NADH-ubiquinone%20oxidoreductase%20chain%20D%20(EC%201.6.5.3)&subsystem_name=Respiratory_Complex_I) |
| Respiration | Electron donating reactions | [Respiratory dehydrogenases 1](https://rast.nmpdr.org/seedviewer.cgi?page=Subsystems&subsystem=Respiratory_dehydrogenases_1&organism=549.408) | [Proline dehydrogenase (EC 1.5.99.8) (Proline oxidase)](https://rast.nmpdr.org/seedviewer.cgi?page=FunctionalRole&role=Proline%20dehydrogenase%20(EC%201.5.99.8)%20(Proline%20oxidase)&subsystem_name=Respiratory_dehydrogenases_1) |
| Respiration | Electron donating reactions | [Respiratory dehydrogenases 1](https://rast.nmpdr.org/seedviewer.cgi?page=Subsystems&subsystem=Respiratory_dehydrogenases_1&organism=549.408) | [Glycerol dehydrogenase (EC 1.1.1.6)](https://rast.nmpdr.org/seedviewer.cgi?page=FunctionalRole&role=Glycerol%20dehydrogenase%20(EC%201.1.1.6)&subsystem_name=Respiratory_dehydrogenases_1) |
| Respiration | Electron donating reactions | [Respiratory dehydrogenases 1](https://rast.nmpdr.org/seedviewer.cgi?page=Subsystems&subsystem=Respiratory_dehydrogenases_1&organism=549.408) | [Glucose dehydrogenase, PQQ-dependent (EC 1.1.5.2)](https://rast.nmpdr.org/seedviewer.cgi?page=FunctionalRole&role=Glucose%20dehydrogenase,%20PQQ-dependent%20(EC%201.1.5.2)&subsystem_name=Respiratory_dehydrogenases_1) |
| Respiration | Electron donating reactions | [Respiratory dehydrogenases 1](https://rast.nmpdr.org/seedviewer.cgi?page=Subsystems&subsystem=Respiratory_dehydrogenases_1&organism=549.408) | [NADH dehydrogenase (EC 1.6.99.3)](https://rast.nmpdr.org/seedviewer.cgi?page=FunctionalRole&role=NADH%20dehydrogenase%20(EC%201.6.99.3)&subsystem_name=Respiratory_dehydrogenases_1) |
| Respiration | Electron donating reactions | [Respiratory dehydrogenases 1](https://rast.nmpdr.org/seedviewer.cgi?page=Subsystems&subsystem=Respiratory_dehydrogenases_1&organism=549.408) | [Aerobic glycerol-3-phosphate dehydrogenase (EC 1.1.5.3)](https://rast.nmpdr.org/seedviewer.cgi?page=FunctionalRole&role=Aerobic%20glycerol-3-phosphate%20dehydrogenase%20(EC%201.1.5.3)&subsystem_name=Respiratory_dehydrogenases_1) |
| Respiration | Electron donating reactions | [Respiratory dehydrogenases 1](https://rast.nmpdr.org/seedviewer.cgi?page=Subsystems&subsystem=Respiratory_dehydrogenases_1&organism=549.408) | [D-amino acid dehydrogenase small subunit (EC 1.4.99.1)](https://rast.nmpdr.org/seedviewer.cgi?page=FunctionalRole&role=D-amino%20acid%20dehydrogenase%20small%20subunit%20(EC%201.4.99.1)&subsystem_name=Respiratory_dehydrogenases_1) |
| Respiration | Electron donating reactions | [Respiratory dehydrogenases 1](https://rast.nmpdr.org/seedviewer.cgi?page=Subsystems&subsystem=Respiratory_dehydrogenases_1&organism=549.408) | [L-lactate dehydrogenase (EC 1.1.2.3)](https://rast.nmpdr.org/seedviewer.cgi?page=FunctionalRole&role=L-lactate%20dehydrogenase%20(EC%201.1.2.3)&subsystem_name=Respiratory_dehydrogenases_1) |
| Respiration | Electron donating reactions | [Respiratory dehydrogenases 1](https://rast.nmpdr.org/seedviewer.cgi?page=Subsystems&subsystem=Respiratory_dehydrogenases_1&organism=549.408) | [D-Lactate dehydrogenase (EC 1.1.2.5)](https://rast.nmpdr.org/seedviewer.cgi?page=FunctionalRole&role=D-Lactate%20dehydrogenase%20(EC%201.1.2.5)&subsystem_name=Respiratory_dehydrogenases_1) |
| Respiration | Electron donating reactions | [Formate dehydrogenase](https://rast.nmpdr.org/seedviewer.cgi?page=Subsystems&subsystem=Formate_dehydrogenase&organism=549.408) | [Formate dehydrogenase O beta subunit (EC 1.2.1.2)](https://rast.nmpdr.org/seedviewer.cgi?page=FunctionalRole&role=Formate%20dehydrogenase%20O%20beta%20subunit%20(EC%201.2.1.2)&subsystem_name=Formate_dehydrogenase) |
| Respiration | Electron donating reactions | [Formate dehydrogenase](https://rast.nmpdr.org/seedviewer.cgi?page=Subsystems&subsystem=Formate_dehydrogenase&organism=549.408) | [Formate dehydrogenase O alpha subunit (EC 1.2.1.2)](https://rast.nmpdr.org/seedviewer.cgi?page=FunctionalRole&role=Formate%20dehydrogenase%20O%20alpha%20subunit%20(EC%201.2.1.2)&subsystem_name=Formate_dehydrogenase) |
| Respiration | Electron donating reactions | [Formate dehydrogenase](https://rast.nmpdr.org/seedviewer.cgi?page=Subsystems&subsystem=Formate_dehydrogenase&organism=549.408) | [Formate dehydrogenase O gamma subunit (EC 1.2.1.2)](https://rast.nmpdr.org/seedviewer.cgi?page=FunctionalRole&role=Formate%20dehydrogenase%20O%20gamma%20subunit%20(EC%201.2.1.2)&subsystem_name=Formate_dehydrogenase) |
| Respiration | Electron donating reactions | [Succinate dehydrogenase](https://rast.nmpdr.org/seedviewer.cgi?page=Subsystems&subsystem=Succinate_dehydrogenase&organism=549.408) | [Succinate dehydrogenase iron-sulfur protein (EC 1.3.99.1)](https://rast.nmpdr.org/seedviewer.cgi?page=FunctionalRole&role=Succinate%20dehydrogenase%20iron-sulfur%20protein%20(EC%201.3.99.1)&subsystem_name=Succinate_dehydrogenase) |
| Respiration | Electron donating reactions | [Succinate dehydrogenase](https://rast.nmpdr.org/seedviewer.cgi?page=Subsystems&subsystem=Succinate_dehydrogenase&organism=549.408) | [Succinate dehydrogenase hydrophobic membrane anchor protein](https://rast.nmpdr.org/seedviewer.cgi?page=FunctionalRole&role=Succinate%20dehydrogenase%20hydrophobic%20membrane%20anchor%20protein&subsystem_name=Succinate_dehydrogenase) |
| Respiration | Electron donating reactions | [Succinate dehydrogenase](https://rast.nmpdr.org/seedviewer.cgi?page=Subsystems&subsystem=Succinate_dehydrogenase&organism=549.408) | [Succinate dehydrogenase cytochrome b-556 subunit](https://rast.nmpdr.org/seedviewer.cgi?page=FunctionalRole&role=Succinate%20dehydrogenase%20cytochrome%20b-556%20subunit&subsystem_name=Succinate_dehydrogenase) |
| Respiration | Electron donating reactions | [Succinate dehydrogenase](https://rast.nmpdr.org/seedviewer.cgi?page=Subsystems&subsystem=Succinate_dehydrogenase&organism=549.408) | [Succinate dehydrogenase flavoprotein subunit (EC 1.3.99.1)](https://rast.nmpdr.org/seedviewer.cgi?page=FunctionalRole&role=Succinate%20dehydrogenase%20flavoprotein%20subunit%20(EC%201.3.99.1)&subsystem_name=Succinate_dehydrogenase) |
| Respiration | Electron donating reactions | [Succinate dehydrogenase](https://rast.nmpdr.org/seedviewer.cgi?page=Subsystems&subsystem=Succinate_dehydrogenase&organism=549.408) | [Fumarate reductase flavoprotein subunit (EC 1.3.99.1)](https://rast.nmpdr.org/seedviewer.cgi?page=FunctionalRole&role=Fumarate%20reductase%20flavoprotein%20subunit%20(EC%201.3.99.1)&subsystem_name=Succinate_dehydrogenase) |
| Respiration | Electron donating reactions | [NADH ubiquinone oxidoreductase](https://rast.nmpdr.org/seedviewer.cgi?page=Subsystems&subsystem=NADH_ubiquinone_oxidoreductase&organism=549.408) | [NADH-ubiquinone oxidoreductase chain K (EC 1.6.5.3)](https://rast.nmpdr.org/seedviewer.cgi?page=FunctionalRole&role=NADH-ubiquinone%20oxidoreductase%20chain%20K%20(EC%201.6.5.3)&subsystem_name=NADH_ubiquinone_oxidoreductase) |
| Respiration | Electron donating reactions | [NADH ubiquinone oxidoreductase](https://rast.nmpdr.org/seedviewer.cgi?page=Subsystems&subsystem=NADH_ubiquinone_oxidoreductase&organism=549.408) | [NADH-ubiquinone oxidoreductase chain L (EC 1.6.5.3)](https://rast.nmpdr.org/seedviewer.cgi?page=FunctionalRole&role=NADH-ubiquinone%20oxidoreductase%20chain%20L%20(EC%201.6.5.3)&subsystem_name=NADH_ubiquinone_oxidoreductase) |
| Respiration | Electron donating reactions | [NADH ubiquinone oxidoreductase](https://rast.nmpdr.org/seedviewer.cgi?page=Subsystems&subsystem=NADH_ubiquinone_oxidoreductase&organism=549.408) | [NADH-ubiquinone oxidoreductase chain J (EC 1.6.5.3)](https://rast.nmpdr.org/seedviewer.cgi?page=FunctionalRole&role=NADH-ubiquinone%20oxidoreductase%20chain%20J%20(EC%201.6.5.3)&subsystem_name=NADH_ubiquinone_oxidoreductase) |
| Respiration | Electron donating reactions | [NADH ubiquinone oxidoreductase](https://rast.nmpdr.org/seedviewer.cgi?page=Subsystems&subsystem=NADH_ubiquinone_oxidoreductase&organism=549.408) | [NADH-ubiquinone oxidoreductase chain N (EC 1.6.5.3)](https://rast.nmpdr.org/seedviewer.cgi?page=FunctionalRole&role=NADH-ubiquinone%20oxidoreductase%20chain%20N%20(EC%201.6.5.3)&subsystem_name=NADH_ubiquinone_oxidoreductase) |
| Respiration | Electron donating reactions | [NADH ubiquinone oxidoreductase](https://rast.nmpdr.org/seedviewer.cgi?page=Subsystems&subsystem=NADH_ubiquinone_oxidoreductase&organism=549.408) | [NADH-ubiquinone oxidoreductase chain H (EC 1.6.5.3)](https://rast.nmpdr.org/seedviewer.cgi?page=FunctionalRole&role=NADH-ubiquinone%20oxidoreductase%20chain%20H%20(EC%201.6.5.3)&subsystem_name=NADH_ubiquinone_oxidoreductase) |
| Respiration | Electron donating reactions | [NADH ubiquinone oxidoreductase](https://rast.nmpdr.org/seedviewer.cgi?page=Subsystems&subsystem=NADH_ubiquinone_oxidoreductase&organism=549.408) | [NADH-ubiquinone oxidoreductase chain E (EC 1.6.5.3)](https://rast.nmpdr.org/seedviewer.cgi?page=FunctionalRole&role=NADH-ubiquinone%20oxidoreductase%20chain%20E%20(EC%201.6.5.3)&subsystem_name=NADH_ubiquinone_oxidoreductase) |
| Respiration | Electron donating reactions | [NADH ubiquinone oxidoreductase](https://rast.nmpdr.org/seedviewer.cgi?page=Subsystems&subsystem=NADH_ubiquinone_oxidoreductase&organism=549.408) | [NADH-ubiquinone oxidoreductase chain B (EC 1.6.5.3)](https://rast.nmpdr.org/seedviewer.cgi?page=FunctionalRole&role=NADH-ubiquinone%20oxidoreductase%20chain%20B%20(EC%201.6.5.3)&subsystem_name=NADH_ubiquinone_oxidoreductase) |
| Respiration | Electron donating reactions | [NADH ubiquinone oxidoreductase](https://rast.nmpdr.org/seedviewer.cgi?page=Subsystems&subsystem=NADH_ubiquinone_oxidoreductase&organism=549.408) | [NADH-ubiquinone oxidoreductase chain I (EC 1.6.5.3)](https://rast.nmpdr.org/seedviewer.cgi?page=FunctionalRole&role=NADH-ubiquinone%20oxidoreductase%20chain%20I%20(EC%201.6.5.3)&subsystem_name=NADH_ubiquinone_oxidoreductase) |
| Respiration | Electron donating reactions | [NADH ubiquinone oxidoreductase](https://rast.nmpdr.org/seedviewer.cgi?page=Subsystems&subsystem=NADH_ubiquinone_oxidoreductase&organism=549.408) | [NADH-ubiquinone oxidoreductase chain M (EC 1.6.5.3)](https://rast.nmpdr.org/seedviewer.cgi?page=FunctionalRole&role=NADH-ubiquinone%20oxidoreductase%20chain%20M%20(EC%201.6.5.3)&subsystem_name=NADH_ubiquinone_oxidoreductase) |
| Respiration | Electron donating reactions | [NADH ubiquinone oxidoreductase](https://rast.nmpdr.org/seedviewer.cgi?page=Subsystems&subsystem=NADH_ubiquinone_oxidoreductase&organism=549.408) | [NADH-ubiquinone oxidoreductase chain F (EC 1.6.5.3)](https://rast.nmpdr.org/seedviewer.cgi?page=FunctionalRole&role=NADH-ubiquinone%20oxidoreductase%20chain%20F%20(EC%201.6.5.3)&subsystem_name=NADH_ubiquinone_oxidoreductase) |
| Respiration | Electron donating reactions | [NADH ubiquinone oxidoreductase](https://rast.nmpdr.org/seedviewer.cgi?page=Subsystems&subsystem=NADH_ubiquinone_oxidoreductase&organism=549.408) | [NADH-ubiquinone oxidoreductase chain G (EC 1.6.5.3)](https://rast.nmpdr.org/seedviewer.cgi?page=FunctionalRole&role=NADH-ubiquinone%20oxidoreductase%20chain%20G%20(EC%201.6.5.3)&subsystem_name=NADH_ubiquinone_oxidoreductase) |
| Respiration | Electron donating reactions | [NADH ubiquinone oxidoreductase](https://rast.nmpdr.org/seedviewer.cgi?page=Subsystems&subsystem=NADH_ubiquinone_oxidoreductase&organism=549.408) | [NADH-ubiquinone oxidoreductase chain C (EC 1.6.5.3)](https://rast.nmpdr.org/seedviewer.cgi?page=FunctionalRole&role=NADH-ubiquinone%20oxidoreductase%20chain%20C%20(EC%201.6.5.3)&subsystem_name=NADH_ubiquinone_oxidoreductase) |
| Respiration | Electron donating reactions | [NADH ubiquinone oxidoreductase](https://rast.nmpdr.org/seedviewer.cgi?page=Subsystems&subsystem=NADH_ubiquinone_oxidoreductase&organism=549.408) | [NADH ubiquinone oxidoreductase chain A (EC 1.6.5.3)](https://rast.nmpdr.org/seedviewer.cgi?page=FunctionalRole&role=NADH%20ubiquinone%20oxidoreductase%20chain%20A%20(EC%201.6.5.3)&subsystem_name=NADH_ubiquinone_oxidoreductase) |
| Respiration | Electron donating reactions | [NADH ubiquinone oxidoreductase](https://rast.nmpdr.org/seedviewer.cgi?page=Subsystems&subsystem=NADH_ubiquinone_oxidoreductase&organism=549.408) | [NADH-ubiquinone oxidoreductase chain D (EC 1.6.5.3)](https://rast.nmpdr.org/seedviewer.cgi?page=FunctionalRole&role=NADH-ubiquinone%20oxidoreductase%20chain%20D%20(EC%201.6.5.3)&subsystem_name=NADH_ubiquinone_oxidoreductase) |
| Respiration | Respiration - no subcategory | [Quinone oxidoreductase family](https://rast.nmpdr.org/seedviewer.cgi?page=Subsystems&subsystem=Quinone_oxidoreductase_family&organism=549.408) | [Quinone oxidoreductase (EC 1.6.5.5)](https://rast.nmpdr.org/seedviewer.cgi?page=FunctionalRole&role=Quinone%20oxidoreductase%20(EC%201.6.5.5)&subsystem_name=Quinone_oxidoreductase_family) |
| Respiration | Respiration - no subcategory | [Quinone oxidoreductase family](https://rast.nmpdr.org/seedviewer.cgi?page=Subsystems&subsystem=Quinone_oxidoreductase_family&organism=549.408) | [Putative oxidoreductase YncB](https://rast.nmpdr.org/seedviewer.cgi?page=FunctionalRole&role=Putative%20oxidoreductase%20YncB&subsystem_name=Quinone_oxidoreductase_family) |
| Respiration | Respiration - no subcategory | [Biogenesis of cytochrome c oxidases](https://rast.nmpdr.org/seedviewer.cgi?page=Subsystems&subsystem=Biogenesis_of_cytochrome_c_oxidases&organism=549.408) | [Ferredoxin--NADP(+) reductase (EC 1.18.1.2)](https://rast.nmpdr.org/seedviewer.cgi?page=FunctionalRole&role=Ferredoxin--NADP(+)%20reductase%20(EC%201.18.1.2)&subsystem_name=Biogenesis_of_cytochrome_c_oxidases) |
| Respiration | Respiration - no subcategory | [Biogenesis of cytochrome c oxidases](https://rast.nmpdr.org/seedviewer.cgi?page=Subsystems&subsystem=Biogenesis_of_cytochrome_c_oxidases&organism=549.408) | [Heme O synthase, protoheme IX farnesyltransferase (EC 2.5.1.-) COX10-CtaB](https://rast.nmpdr.org/seedviewer.cgi?page=FunctionalRole&role=Heme%20O%20synthase,%20protoheme%20IX%20farnesyltransferase%20(EC%202.5.1.-)%20COX10-CtaB&subsystem_name=Biogenesis_of_cytochrome_c_oxidases) |
| Respiration | Respiration - no subcategory | [Formate hydrogenase](https://rast.nmpdr.org/seedviewer.cgi?page=Subsystems&subsystem=Formate_hydrogenase&organism=549.408) | [Formate dehydrogenase chain D (EC 1.2.1.2)](https://rast.nmpdr.org/seedviewer.cgi?page=FunctionalRole&role=Formate%20dehydrogenase%20chain%20D%20(EC%201.2.1.2)&subsystem_name=Formate_hydrogenase) |
| Respiration | Respiration - no subcategory | [Formate hydrogenase](https://rast.nmpdr.org/seedviewer.cgi?page=Subsystems&subsystem=Formate_hydrogenase&organism=549.408) | [formate dehydrogenase formation protein FdhE](https://rast.nmpdr.org/seedviewer.cgi?page=FunctionalRole&role=formate%20dehydrogenase%20formation%20protein%20FdhE&subsystem_name=Formate_hydrogenase) |
| Respiration | Respiration - no subcategory | [Formate hydrogenase](https://rast.nmpdr.org/seedviewer.cgi?page=Subsystems&subsystem=Formate_hydrogenase&organism=549.408) | [Formate dehydrogenase related protein](https://rast.nmpdr.org/seedviewer.cgi?page=FunctionalRole&role=Formate%20dehydrogenase%20related%20protein&subsystem_name=Formate_hydrogenase) |
| Respiration | Respiration - no subcategory | [Formate hydrogenase](https://rast.nmpdr.org/seedviewer.cgi?page=Subsystems&subsystem=Formate_hydrogenase&organism=549.408) | [Formate dehydrogenase O beta subunit (EC 1.2.1.2)](https://rast.nmpdr.org/seedviewer.cgi?page=FunctionalRole&role=Formate%20dehydrogenase%20O%20beta%20subunit%20(EC%201.2.1.2)&subsystem_name=Formate_hydrogenase) |
| Respiration | Respiration - no subcategory | [Formate hydrogenase](https://rast.nmpdr.org/seedviewer.cgi?page=Subsystems&subsystem=Formate_hydrogenase&organism=549.408) | [Formate dehydrogenase O alpha subunit (EC 1.2.1.2)](https://rast.nmpdr.org/seedviewer.cgi?page=FunctionalRole&role=Formate%20dehydrogenase%20O%20alpha%20subunit%20(EC%201.2.1.2)&subsystem_name=Formate_hydrogenase) |
| Respiration | Respiration - no subcategory | [Formate hydrogenase](https://rast.nmpdr.org/seedviewer.cgi?page=Subsystems&subsystem=Formate_hydrogenase&organism=549.408) | [Putative formate dehydrogenase oxidoreductase protein](https://rast.nmpdr.org/seedviewer.cgi?page=FunctionalRole&role=Putative%20formate%20dehydrogenase%20oxidoreductase%20protein&subsystem_name=Formate_hydrogenase) |
| Respiration | Respiration - no subcategory | [Formate hydrogenase](https://rast.nmpdr.org/seedviewer.cgi?page=Subsystems&subsystem=Formate_hydrogenase&organism=549.408) | [Formate dehydrogenase O gamma subunit (EC 1.2.1.2)](https://rast.nmpdr.org/seedviewer.cgi?page=FunctionalRole&role=Formate%20dehydrogenase%20O%20gamma%20subunit%20(EC%201.2.1.2)&subsystem_name=Formate_hydrogenase) |
| Respiration | Respiration - no subcategory | [Formate hydrogenase](https://rast.nmpdr.org/seedviewer.cgi?page=Subsystems&subsystem=Formate_hydrogenase&organism=549.408) | [Formate dehydrogenase -O, gamma subunit (EC 1.2.1.2)](https://rast.nmpdr.org/seedviewer.cgi?page=FunctionalRole&role=Formate%20dehydrogenase%20-O,%20gamma%20subunit%20(EC%201.2.1.2)&subsystem_name=Formate_hydrogenase) |
| Respiration | Respiration - no subcategory | [Biogenesis of c-type cytochromes](https://rast.nmpdr.org/seedviewer.cgi?page=Subsystems&subsystem=Biogenesis_of_c-type_cytochromes&organism=549.408) | [Periplasmic thiol:disulfide oxidoreductase DsbB, required for DsbA reoxidation](https://rast.nmpdr.org/seedviewer.cgi?page=FunctionalRole&role=Periplasmic%20thiol:disulfide%20oxidoreductase%20DsbB,%20required%20for%20DsbA%20reoxidation&subsystem_name=Biogenesis_of_c-type_cytochromes) |
| Respiration | Respiration - no subcategory | [Biogenesis of c-type cytochromes](https://rast.nmpdr.org/seedviewer.cgi?page=Subsystems&subsystem=Biogenesis_of_c-type_cytochromes&organism=549.408) | [Cytochrome c heme lyase subunit CcmH](https://rast.nmpdr.org/seedviewer.cgi?page=FunctionalRole&role=Cytochrome%20c%20heme%20lyase%20subunit%20CcmH&subsystem_name=Biogenesis_of_c-type_cytochromes) |
| Respiration | Respiration - no subcategory | [Biogenesis of c-type cytochromes](https://rast.nmpdr.org/seedviewer.cgi?page=Subsystems&subsystem=Biogenesis_of_c-type_cytochromes&organism=549.408) | [Cytochrome c-type biogenesis protein CcmC, putative heme lyase for CcmE](https://rast.nmpdr.org/seedviewer.cgi?page=FunctionalRole&role=Cytochrome%20c-type%20biogenesis%20protein%20CcmC,%20putative%20heme%20lyase%20for%20CcmE&subsystem_name=Biogenesis_of_c-type_cytochromes) |
| Respiration | Respiration - no subcategory | [Biogenesis of c-type cytochromes](https://rast.nmpdr.org/seedviewer.cgi?page=Subsystems&subsystem=Biogenesis_of_c-type_cytochromes&organism=549.408) | [ABC transporter involved in cytochrome c biogenesis, CcmB subunit](https://rast.nmpdr.org/seedviewer.cgi?page=FunctionalRole&role=ABC%20transporter%20involved%20in%20cytochrome%20c%20biogenesis,%20CcmB%20subunit&subsystem_name=Biogenesis_of_c-type_cytochromes) |
| Respiration | Respiration - no subcategory | [Biogenesis of c-type cytochromes](https://rast.nmpdr.org/seedviewer.cgi?page=Subsystems&subsystem=Biogenesis_of_c-type_cytochromes&organism=549.408) | [Cytochrome c-type biogenesis protein CcmE, heme chaperone](https://rast.nmpdr.org/seedviewer.cgi?page=FunctionalRole&role=Cytochrome%20c-type%20biogenesis%20protein%20CcmE,%20heme%20chaperone&subsystem_name=Biogenesis_of_c-type_cytochromes) |
| Respiration | Respiration - no subcategory | [Biogenesis of c-type cytochromes](https://rast.nmpdr.org/seedviewer.cgi?page=Subsystems&subsystem=Biogenesis_of_c-type_cytochromes&organism=549.408) | [Periplasmic thiol:disulfide interchange protein DsbA](https://rast.nmpdr.org/seedviewer.cgi?page=FunctionalRole&role=Periplasmic%20thiol:disulfide%20interchange%20protein%20DsbA&subsystem_name=Biogenesis_of_c-type_cytochromes) |
| Respiration | Respiration - no subcategory | [Biogenesis of c-type cytochromes](https://rast.nmpdr.org/seedviewer.cgi?page=Subsystems&subsystem=Biogenesis_of_c-type_cytochromes&organism=549.408) | [Cytochrome c heme lyase subunit CcmL](https://rast.nmpdr.org/seedviewer.cgi?page=FunctionalRole&role=Cytochrome%20c%20heme%20lyase%20subunit%20CcmL&subsystem_name=Biogenesis_of_c-type_cytochromes) |
| Respiration | Respiration - no subcategory | [Biogenesis of c-type cytochromes](https://rast.nmpdr.org/seedviewer.cgi?page=Subsystems&subsystem=Biogenesis_of_c-type_cytochromes&organism=549.408) | [ABC transporter involved in cytochrome c biogenesis, ATPase component CcmA](https://rast.nmpdr.org/seedviewer.cgi?page=FunctionalRole&role=ABC%20transporter%20involved%20in%20cytochrome%20c%20biogenesis,%20ATPase%20component%20CcmA&subsystem_name=Biogenesis_of_c-type_cytochromes) |
| Respiration | Respiration - no subcategory | [Biogenesis of c-type cytochromes](https://rast.nmpdr.org/seedviewer.cgi?page=Subsystems&subsystem=Biogenesis_of_c-type_cytochromes&organism=549.408) | [Cytochrome c heme lyase subunit CcmF](https://rast.nmpdr.org/seedviewer.cgi?page=FunctionalRole&role=Cytochrome%20c%20heme%20lyase%20subunit%20CcmF&subsystem_name=Biogenesis_of_c-type_cytochromes) |
| Respiration | Respiration - no subcategory | [Biogenesis of c-type cytochromes](https://rast.nmpdr.org/seedviewer.cgi?page=Subsystems&subsystem=Biogenesis_of_c-type_cytochromes&organism=549.408) | [Cytochrome c-type biogenesis protein CcmG/DsbE, thiol:disulfide oxidoreductase](https://rast.nmpdr.org/seedviewer.cgi?page=FunctionalRole&role=Cytochrome%20c-type%20biogenesis%20protein%20CcmG/DsbE,%20thiol:disulfide%20oxidoreductase&subsystem_name=Biogenesis_of_c-type_cytochromes) |
| Respiration | Respiration - no subcategory | [Biogenesis of c-type cytochromes](https://rast.nmpdr.org/seedviewer.cgi?page=Subsystems&subsystem=Biogenesis_of_c-type_cytochromes&organism=549.408) | [Cytochrome c-type biogenesis protein CcmD, interacts with CcmCE](https://rast.nmpdr.org/seedviewer.cgi?page=FunctionalRole&role=Cytochrome%20c-type%20biogenesis%20protein%20CcmD,%20interacts%20with%20CcmCE&subsystem_name=Biogenesis_of_c-type_cytochromes) |
| Respiration | Respiration - no subcategory | [Biogenesis of c-type cytochromes](https://rast.nmpdr.org/seedviewer.cgi?page=Subsystems&subsystem=Biogenesis_of_c-type_cytochromes&organism=549.408) | [Cytochrome c-type biogenesis protein DsbD, protein-disulfide reductase (EC 1.8.1.8)](https://rast.nmpdr.org/seedviewer.cgi?page=FunctionalRole&role=Cytochrome%20c-type%20biogenesis%20protein%20DsbD,%20protein-disulfide%20reductase%20(EC%201.8.1.8)&subsystem_name=Biogenesis_of_c-type_cytochromes) |
| Respiration | Respiration - no subcategory | [Soluble cytochromes and functionally related electron carriers](https://rast.nmpdr.org/seedviewer.cgi?page=Subsystems&subsystem=Soluble_cytochromes_and_functionally_related_electron_carriers&organism=549.408) | [Cytochrome c4](https://rast.nmpdr.org/seedviewer.cgi?page=FunctionalRole&role=Cytochrome%20c4&subsystem_name=Soluble_cytochromes_and_functionally_related_electron_carriers) |
| Respiration | Respiration - no subcategory | [Soluble cytochromes and functionally related electron carriers](https://rast.nmpdr.org/seedviewer.cgi?page=Subsystems&subsystem=Soluble_cytochromes_and_functionally_related_electron_carriers&organism=549.408) | [Soluble cytochrome b562](https://rast.nmpdr.org/seedviewer.cgi?page=FunctionalRole&role=Soluble%20cytochrome%20b562&subsystem_name=Soluble_cytochromes_and_functionally_related_electron_carriers) |
| Respiration | Respiration - no subcategory | [Soluble cytochromes and functionally related electron carriers](https://rast.nmpdr.org/seedviewer.cgi?page=Subsystems&subsystem=Soluble_cytochromes_and_functionally_related_electron_carriers&organism=549.408) | [Ferredoxin](https://rast.nmpdr.org/seedviewer.cgi?page=FunctionalRole&role=Ferredoxin&subsystem_name=Soluble_cytochromes_and_functionally_related_electron_carriers) |
| Stress Response | Osmotic stress | [Osmotic stress cluster](https://rast.nmpdr.org/seedviewer.cgi?page=Subsystems&subsystem=Osmotic_stress_cluster&organism=549.408) | [Transcriptional regulatory protein YciT](https://rast.nmpdr.org/seedviewer.cgi?page=FunctionalRole&role=Transcriptional%20regulatory%20protein%20YciT&subsystem_name=Osmotic_stress_cluster) |
| Stress Response | Osmotic stress | [Osmotic stress cluster](https://rast.nmpdr.org/seedviewer.cgi?page=Subsystems&subsystem=Osmotic_stress_cluster&organism=549.408) | [Phosphatidylglycerophosphatase B (EC 3.1.3.27)](https://rast.nmpdr.org/seedviewer.cgi?page=FunctionalRole&role=Phosphatidylglycerophosphatase%20B%20(EC%203.1.3.27)&subsystem_name=Osmotic_stress_cluster) |
| Stress Response | Osmotic stress | [Osmotic stress cluster](https://rast.nmpdr.org/seedviewer.cgi?page=Subsystems&subsystem=Osmotic_stress_cluster&organism=549.408) | [Heat shock (predicted periplasmic) protein YciM, precursor](https://rast.nmpdr.org/seedviewer.cgi?page=FunctionalRole&role=Heat%20shock%20(predicted%20periplasmic)%20protein%20YciM,%20precursor&subsystem_name=Osmotic_stress_cluster) |
| Stress Response | Osmotic stress | [Osmotic stress cluster](https://rast.nmpdr.org/seedviewer.cgi?page=Subsystems&subsystem=Osmotic_stress_cluster&organism=549.408) | [Osmotically inducible lipoprotein B precursor](https://rast.nmpdr.org/seedviewer.cgi?page=FunctionalRole&role=Osmotically%20inducible%20lipoprotein%20B%20precursor&subsystem_name=Osmotic_stress_cluster) |
| Stress Response | Osmotic stress | [Osmoregulation](https://rast.nmpdr.org/seedviewer.cgi?page=Subsystems&subsystem=Osmoregulation&organism=549.408) | [Outer membrane protein A precursor](https://rast.nmpdr.org/seedviewer.cgi?page=FunctionalRole&role=Outer%20membrane%20protein%20A%20precursor&subsystem_name=Osmoregulation) |
| Stress Response | Osmotic stress | [Osmoregulation](https://rast.nmpdr.org/seedviewer.cgi?page=Subsystems&subsystem=Osmoregulation&organism=549.408) | [Aquaporin Z](https://rast.nmpdr.org/seedviewer.cgi?page=FunctionalRole&role=Aquaporin%20Z&subsystem_name=Osmoregulation) |
| Stress Response | Osmotic stress | [Osmoregulation](https://rast.nmpdr.org/seedviewer.cgi?page=Subsystems&subsystem=Osmoregulation&organism=549.408) | [Osmotically inducible protein OsmY](https://rast.nmpdr.org/seedviewer.cgi?page=FunctionalRole&role=Osmotically%20inducible%20protein%20OsmY&subsystem_name=Osmoregulation) |
| Stress Response | Osmotic stress | [Osmoregulation](https://rast.nmpdr.org/seedviewer.cgi?page=Subsystems&subsystem=Osmoregulation&organism=549.408) | [Glycerol uptake facilitator protein](https://rast.nmpdr.org/seedviewer.cgi?page=FunctionalRole&role=Glycerol%20uptake%20facilitator%20protein&subsystem_name=Osmoregulation) |
| Stress Response | Osmotic stress | [Osmoprotectant ABC transporter YehZYXW of Enterobacteriales](https://rast.nmpdr.org/seedviewer.cgi?page=Subsystems&subsystem=Osmoprotectant_ABC_transporter_YehZYXW_of_Enterobacteriales&organism=549.408) | [Osmoprotectant ABC transporter permease protein YehY](https://rast.nmpdr.org/seedviewer.cgi?page=FunctionalRole&role=Osmoprotectant%20ABC%20transporter%20permease%20protein%20YehY&subsystem_name=Osmoprotectant_ABC_transporter_YehZYXW_of_Enterobacteriales) |
| Stress Response | Osmotic stress | [Osmoprotectant ABC transporter YehZYXW of Enterobacteriales](https://rast.nmpdr.org/seedviewer.cgi?page=Subsystems&subsystem=Osmoprotectant_ABC_transporter_YehZYXW_of_Enterobacteriales&organism=549.408) | [Osmoprotectant ABC transporter inner membrane protein YehW](https://rast.nmpdr.org/seedviewer.cgi?page=FunctionalRole&role=Osmoprotectant%20ABC%20transporter%20inner%20membrane%20protein%20YehW&subsystem_name=Osmoprotectant_ABC_transporter_YehZYXW_of_Enterobacteriales) |
| Stress Response | Osmotic stress | [Osmoprotectant ABC transporter YehZYXW of Enterobacteriales](https://rast.nmpdr.org/seedviewer.cgi?page=Subsystems&subsystem=Osmoprotectant_ABC_transporter_YehZYXW_of_Enterobacteriales&organism=549.408) | [Osmoprotectant ABC transporter binding protein YehZ](https://rast.nmpdr.org/seedviewer.cgi?page=FunctionalRole&role=Osmoprotectant%20ABC%20transporter%20binding%20protein%20YehZ&subsystem_name=Osmoprotectant_ABC_transporter_YehZYXW_of_Enterobacteriales) |
| Stress Response | Osmotic stress | [Osmoprotectant ABC transporter YehZYXW of Enterobacteriales](https://rast.nmpdr.org/seedviewer.cgi?page=Subsystems&subsystem=Osmoprotectant_ABC_transporter_YehZYXW_of_Enterobacteriales&organism=549.408) | [Osmoprotectant ABC transporter ATP-binding subunit YehX](https://rast.nmpdr.org/seedviewer.cgi?page=FunctionalRole&role=Osmoprotectant%20ABC%20transporter%20ATP-binding%20subunit%20YehX&subsystem_name=Osmoprotectant_ABC_transporter_YehZYXW_of_Enterobacteriales) |
| Stress Response | Osmotic stress | [Synthesis of osmoregulated periplasmic glucans](https://rast.nmpdr.org/seedviewer.cgi?page=Subsystems&subsystem=Synthesis_of_osmoregulated_periplasmic_glucans&organism=549.408) | [Glucans biosynthesis protein C (EC 2.1.-.-)](https://rast.nmpdr.org/seedviewer.cgi?page=FunctionalRole&role=Glucans%20biosynthesis%20protein%20C%20(EC%202.1.-.-)&subsystem_name=Synthesis_of_osmoregulated_periplasmic_glucans) |
| Stress Response | Osmotic stress | [Synthesis of osmoregulated periplasmic glucans](https://rast.nmpdr.org/seedviewer.cgi?page=Subsystems&subsystem=Synthesis_of_osmoregulated_periplasmic_glucans&organism=549.408) | [Cyclic beta-1,2-glucan synthase (EC 2.4.1.-)](https://rast.nmpdr.org/seedviewer.cgi?page=FunctionalRole&role=Cyclic%20beta-1,2-glucan%20synthase%20(EC%202.4.1.-)&subsystem_name=Synthesis_of_osmoregulated_periplasmic_glucans) |
| Stress Response | Osmotic stress | [Synthesis of osmoregulated periplasmic glucans](https://rast.nmpdr.org/seedviewer.cgi?page=Subsystems&subsystem=Synthesis_of_osmoregulated_periplasmic_glucans&organism=549.408) | [Glucans biosynthesis protein D precursor](https://rast.nmpdr.org/seedviewer.cgi?page=FunctionalRole&role=Glucans%20biosynthesis%20protein%20D%20precursor&subsystem_name=Synthesis_of_osmoregulated_periplasmic_glucans) |
| Stress Response | Osmotic stress | [Synthesis of osmoregulated periplasmic glucans](https://rast.nmpdr.org/seedviewer.cgi?page=Subsystems&subsystem=Synthesis_of_osmoregulated_periplasmic_glucans&organism=549.408) | [Phosphoglycerol transferase I (EC 2.7.8.20)](https://rast.nmpdr.org/seedviewer.cgi?page=FunctionalRole&role=Phosphoglycerol%20transferase%20I%20(EC%202.7.8.20)&subsystem_name=Synthesis_of_osmoregulated_periplasmic_glucans) |
| Stress Response | Osmotic stress | [Synthesis of osmoregulated periplasmic glucans](https://rast.nmpdr.org/seedviewer.cgi?page=Subsystems&subsystem=Synthesis_of_osmoregulated_periplasmic_glucans&organism=549.408) | [Glucans biosynthesis glucosyltransferase H (EC 2.4.1.-)](https://rast.nmpdr.org/seedviewer.cgi?page=FunctionalRole&role=Glucans%20biosynthesis%20glucosyltransferase%20H%20(EC%202.4.1.-)&subsystem_name=Synthesis_of_osmoregulated_periplasmic_glucans) |
| Stress Response | Osmotic stress | [Synthesis of osmoregulated periplasmic glucans](https://rast.nmpdr.org/seedviewer.cgi?page=Subsystems&subsystem=Synthesis_of_osmoregulated_periplasmic_glucans&organism=549.408) | [Glucans biosynthesis protein G precursor](https://rast.nmpdr.org/seedviewer.cgi?page=FunctionalRole&role=Glucans%20biosynthesis%20protein%20G%20precursor&subsystem_name=Synthesis_of_osmoregulated_periplasmic_glucans) |
| Stress Response | Osmotic stress | [Choline and Betaine Uptake and Betaine Biosynthesis](https://rast.nmpdr.org/seedviewer.cgi?page=Subsystems&subsystem=Choline_and_Betaine_Uptake_and_Betaine_Biosynthesis&organism=549.408) | [L-proline glycine betaine ABC transport system permease protein ProV (TC 3.A.1.12.1)](https://rast.nmpdr.org/seedviewer.cgi?page=FunctionalRole&role=L-proline%20glycine%20betaine%20ABC%20transport%20system%20permease%20protein%20ProV%20(TC%203.A.1.12.1)&subsystem_name=Choline_and_Betaine_Uptake_and_Betaine_Biosynthesis) |
| Stress Response | Osmotic stress | [Choline and Betaine Uptake and Betaine Biosynthesis](https://rast.nmpdr.org/seedviewer.cgi?page=Subsystems&subsystem=Choline_and_Betaine_Uptake_and_Betaine_Biosynthesis&organism=549.408) | [HTH-type transcriptional regulator BetI](https://rast.nmpdr.org/seedviewer.cgi?page=FunctionalRole&role=HTH-type%20transcriptional%20regulator%20BetI&subsystem_name=Choline_and_Betaine_Uptake_and_Betaine_Biosynthesis) |
| Stress Response | Osmotic stress | [Choline and Betaine Uptake and Betaine Biosynthesis](https://rast.nmpdr.org/seedviewer.cgi?page=Subsystems&subsystem=Choline_and_Betaine_Uptake_and_Betaine_Biosynthesis&organism=549.408) | [L-proline glycine betaine binding ABC transporter protein ProX (TC 3.A.1.12.1)](https://rast.nmpdr.org/seedviewer.cgi?page=FunctionalRole&role=L-proline%20glycine%20betaine%20binding%20ABC%20transporter%20protein%20ProX%20(TC%203.A.1.12.1)&subsystem_name=Choline_and_Betaine_Uptake_and_Betaine_Biosynthesis) |
| Stress Response | Osmotic stress | [Choline and Betaine Uptake and Betaine Biosynthesis](https://rast.nmpdr.org/seedviewer.cgi?page=Subsystems&subsystem=Choline_and_Betaine_Uptake_and_Betaine_Biosynthesis&organism=549.408) | [High-affinity choline uptake protein BetT](https://rast.nmpdr.org/seedviewer.cgi?page=FunctionalRole&role=High-affinity%20choline%20uptake%20protein%20BetT&subsystem_name=Choline_and_Betaine_Uptake_and_Betaine_Biosynthesis) |
| Stress Response | Osmotic stress | [Choline and Betaine Uptake and Betaine Biosynthesis](https://rast.nmpdr.org/seedviewer.cgi?page=Subsystems&subsystem=Choline_and_Betaine_Uptake_and_Betaine_Biosynthesis&organism=549.408) | [Choline dehydrogenase (EC 1.1.99.1)](https://rast.nmpdr.org/seedviewer.cgi?page=FunctionalRole&role=Choline%20dehydrogenase%20(EC%201.1.99.1)&subsystem_name=Choline_and_Betaine_Uptake_and_Betaine_Biosynthesis) |
| Stress Response | Osmotic stress | [Choline and Betaine Uptake and Betaine Biosynthesis](https://rast.nmpdr.org/seedviewer.cgi?page=Subsystems&subsystem=Choline_and_Betaine_Uptake_and_Betaine_Biosynthesis&organism=549.408) | [L-proline glycine betaine ABC transport system permease protein ProW (TC 3.A.1.12.1)](https://rast.nmpdr.org/seedviewer.cgi?page=FunctionalRole&role=L-proline%20glycine%20betaine%20ABC%20transport%20system%20permease%20protein%20ProW%20(TC%203.A.1.12.1)&subsystem_name=Choline_and_Betaine_Uptake_and_Betaine_Biosynthesis) |
| Stress Response | Osmotic stress | [Choline and Betaine Uptake and Betaine Biosynthesis](https://rast.nmpdr.org/seedviewer.cgi?page=Subsystems&subsystem=Choline_and_Betaine_Uptake_and_Betaine_Biosynthesis&organism=549.408) | [Betaine aldehyde dehydrogenase (EC 1.2.1.8)](https://rast.nmpdr.org/seedviewer.cgi?page=FunctionalRole&role=Betaine%20aldehyde%20dehydrogenase%20(EC%201.2.1.8)&subsystem_name=Choline_and_Betaine_Uptake_and_Betaine_Biosynthesis) |
| Stress Response | Oxidative stress | [NADPH:quinone oxidoreductase 2](https://rast.nmpdr.org/seedviewer.cgi?page=Subsystems&subsystem=NADPH:quinone_oxidoreductase_2&organism=549.408) | [Redox-sensing transcriptional regulator QorR](https://rast.nmpdr.org/seedviewer.cgi?page=FunctionalRole&role=Redox-sensing%20transcriptional%20regulator%20QorR&subsystem_name=NADPH:quinone_oxidoreductase_2) |
| Stress Response | Oxidative stress | [NADPH:quinone oxidoreductase 2](https://rast.nmpdr.org/seedviewer.cgi?page=Subsystems&subsystem=NADPH:quinone_oxidoreductase_2&organism=549.408) | [NADPH:quinone oxidoreductase 2](https://rast.nmpdr.org/seedviewer.cgi?page=FunctionalRole&role=NADPH:quinone%20oxidoreductase%202&subsystem_name=NADPH:quinone_oxidoreductase_2) |
| Stress Response | Oxidative stress | [Glutathione: Non-redox reactions](https://rast.nmpdr.org/seedviewer.cgi?page=Subsystems&subsystem=Glutathione:_Non-redox_reactions&organism=549.408) | [Glutathione S-transferase (EC 2.5.1.18)](https://rast.nmpdr.org/seedviewer.cgi?page=FunctionalRole&role=Glutathione%20S-transferase%20(EC%202.5.1.18)&subsystem_name=Glutathione:_Non-redox_reactions) |
| Stress Response | Oxidative stress | [Glutathione: Non-redox reactions](https://rast.nmpdr.org/seedviewer.cgi?page=Subsystems&subsystem=Glutathione:_Non-redox_reactions&organism=549.408) | [Uncharacterized glutathione S-transferase-like protein](https://rast.nmpdr.org/seedviewer.cgi?page=FunctionalRole&role=Uncharacterized%20glutathione%20S-transferase-like%20protein&subsystem_name=Glutathione:_Non-redox_reactions) |
| Stress Response | Oxidative stress | [Glutathione: Non-redox reactions](https://rast.nmpdr.org/seedviewer.cgi?page=Subsystems&subsystem=Glutathione:_Non-redox_reactions&organism=549.408) | [Lactoylglutathione lyase (EC 4.4.1.5)](https://rast.nmpdr.org/seedviewer.cgi?page=FunctionalRole&role=Lactoylglutathione%20lyase%20(EC%204.4.1.5)&subsystem_name=Glutathione:_Non-redox_reactions) |
| Stress Response | Oxidative stress | [Glutathione: Non-redox reactions](https://rast.nmpdr.org/seedviewer.cgi?page=Subsystems&subsystem=Glutathione:_Non-redox_reactions&organism=549.408) | [FIG005121: SAM-dependent methyltransferase (EC 2.1.1.-)](https://rast.nmpdr.org/seedviewer.cgi?page=FunctionalRole&role=FIG005121:%20SAM-dependent%20methyltransferase%20(EC%202.1.1.-)&subsystem_name=Glutathione:_Non-redox_reactions) |
| Stress Response | Oxidative stress | [Glutathione: Non-redox reactions](https://rast.nmpdr.org/seedviewer.cgi?page=Subsystems&subsystem=Glutathione:_Non-redox_reactions&organism=549.408) | [Uncharacterized GST-like protein yibF](https://rast.nmpdr.org/seedviewer.cgi?page=FunctionalRole&role=Uncharacterized%20GST-like%20protein%20yibF&subsystem_name=Glutathione:_Non-redox_reactions) |
| Stress Response | Oxidative stress | [Glutathione: Non-redox reactions](https://rast.nmpdr.org/seedviewer.cgi?page=Subsystems&subsystem=Glutathione:_Non-redox_reactions&organism=549.408) | [Glutathione S-transferase, omega (EC 2.5.1.18)](https://rast.nmpdr.org/seedviewer.cgi?page=FunctionalRole&role=Glutathione%20S-transferase,%20omega%20(EC%202.5.1.18)&subsystem_name=Glutathione:_Non-redox_reactions) |
| Stress Response | Oxidative stress | [Glutathione: Non-redox reactions](https://rast.nmpdr.org/seedviewer.cgi?page=Subsystems&subsystem=Glutathione:_Non-redox_reactions&organism=549.408) | [Hydroxyacylglutathione hydrolase (EC 3.1.2.6)](https://rast.nmpdr.org/seedviewer.cgi?page=FunctionalRole&role=Hydroxyacylglutathione%20hydrolase%20(EC%203.1.2.6)&subsystem_name=Glutathione:_Non-redox_reactions) |
| Stress Response | Oxidative stress | [Glutathione: Non-redox reactions](https://rast.nmpdr.org/seedviewer.cgi?page=Subsystems&subsystem=Glutathione:_Non-redox_reactions&organism=549.408) | [Probable glutathione S-transferase (EC 2.5.1.18), YfcF homolog](https://rast.nmpdr.org/seedviewer.cgi?page=FunctionalRole&role=Probable%20glutathione%20S-transferase%20(EC%202.5.1.18),%20YfcF%20homolog&subsystem_name=Glutathione:_Non-redox_reactions) |
| Stress Response | Oxidative stress | [Redox-dependent regulation of nucleus processes](https://rast.nmpdr.org/seedviewer.cgi?page=Subsystems&subsystem=Redox-dependent_regulation_of_nucleus_processes&organism=549.408) | [Nicotinate phosphoribosyltransferase (EC 2.4.2.11)](https://rast.nmpdr.org/seedviewer.cgi?page=FunctionalRole&role=Nicotinate%20phosphoribosyltransferase%20(EC%202.4.2.11)&subsystem_name=Redox-dependent_regulation_of_nucleus_processes) |
| Stress Response | Oxidative stress | [Redox-dependent regulation of nucleus processes](https://rast.nmpdr.org/seedviewer.cgi?page=Subsystems&subsystem=Redox-dependent_regulation_of_nucleus_processes&organism=549.408) | [Nicotinamidase (EC 3.5.1.19)](https://rast.nmpdr.org/seedviewer.cgi?page=FunctionalRole&role=Nicotinamidase%20(EC%203.5.1.19)&subsystem_name=Redox-dependent_regulation_of_nucleus_processes) |
| Stress Response | Oxidative stress | [Redox-dependent regulation of nucleus processes](https://rast.nmpdr.org/seedviewer.cgi?page=Subsystems&subsystem=Redox-dependent_regulation_of_nucleus_processes&organism=549.408) | [NAD-dependent protein deacetylase of SIR2 family](https://rast.nmpdr.org/seedviewer.cgi?page=FunctionalRole&role=NAD-dependent%20protein%20deacetylase%20of%20SIR2%20family&subsystem_name=Redox-dependent_regulation_of_nucleus_processes) |
| Stress Response | Oxidative stress | [Redox-dependent regulation of nucleus processes](https://rast.nmpdr.org/seedviewer.cgi?page=Subsystems&subsystem=Redox-dependent_regulation_of_nucleus_processes&organism=549.408) | [NAD-dependent glyceraldehyde-3-phosphate dehydrogenase (EC 1.2.1.12)](https://rast.nmpdr.org/seedviewer.cgi?page=FunctionalRole&role=NAD-dependent%20glyceraldehyde-3-phosphate%20dehydrogenase%20(EC%201.2.1.12)&subsystem_name=Redox-dependent_regulation_of_nucleus_processes) |
| Stress Response | Oxidative stress | [Glutathione: Redox cycle](https://rast.nmpdr.org/seedviewer.cgi?page=Subsystems&subsystem=Glutathione:_Redox_cycle&organism=549.408) | [Glutathione reductase (EC 1.8.1.7)](https://rast.nmpdr.org/seedviewer.cgi?page=FunctionalRole&role=Glutathione%20reductase%20(EC%201.8.1.7)&subsystem_name=Glutathione:_Redox_cycle) |
| Stress Response | Oxidative stress | [Glutathione: Redox cycle](https://rast.nmpdr.org/seedviewer.cgi?page=Subsystems&subsystem=Glutathione:_Redox_cycle&organism=549.408) | [Glutaredoxin-like protein NrdH, required for reduction of Ribonucleotide reductase class Ib](https://rast.nmpdr.org/seedviewer.cgi?page=FunctionalRole&role=Glutaredoxin-like%20protein%20NrdH,%20required%20for%20reduction%20of%20Ribonucleotide%20reductase%20class%20Ib&subsystem_name=Glutathione:_Redox_cycle) |
| Stress Response | Oxidative stress | [Glutathione: Redox cycle](https://rast.nmpdr.org/seedviewer.cgi?page=Subsystems&subsystem=Glutathione:_Redox_cycle&organism=549.408) | [Glutaredoxin 3 (Grx3)](https://rast.nmpdr.org/seedviewer.cgi?page=FunctionalRole&role=Glutaredoxin%203%20(Grx3)&subsystem_name=Glutathione:_Redox_cycle) |
| Stress Response | Oxidative stress | [Glutathione: Redox cycle](https://rast.nmpdr.org/seedviewer.cgi?page=Subsystems&subsystem=Glutathione:_Redox_cycle&organism=549.408) | [Glutaredoxin 1](https://rast.nmpdr.org/seedviewer.cgi?page=FunctionalRole&role=Glutaredoxin%201&subsystem_name=Glutathione:_Redox_cycle) |
| Stress Response | Oxidative stress | [Glutathione: Redox cycle](https://rast.nmpdr.org/seedviewer.cgi?page=Subsystems&subsystem=Glutathione:_Redox_cycle&organism=549.408) | [Glutaredoxin 2](https://rast.nmpdr.org/seedviewer.cgi?page=FunctionalRole&role=Glutaredoxin%202&subsystem_name=Glutathione:_Redox_cycle) |
| Stress Response | Oxidative stress | [Glutaredoxins](https://rast.nmpdr.org/seedviewer.cgi?page=Subsystems&subsystem=Glutaredoxins&organism=549.408) | [Flavohemoprotein (Hemoglobin-like protein) (Flavohemoglobin) (Nitric oxide dioxygenase) (EC 1.14.12.17)](https://rast.nmpdr.org/seedviewer.cgi?page=FunctionalRole&role=Flavohemoprotein%20(Hemoglobin-like%20protein)%20(Flavohemoglobin)%20(Nitric%20oxide%20dioxygenase)%20(EC%201.14.12.17)&subsystem_name=Glutaredoxins) |
| Stress Response | Oxidative stress | [Glutaredoxins](https://rast.nmpdr.org/seedviewer.cgi?page=Subsystems&subsystem=Glutaredoxins&organism=549.408) | [Probable monothiol glutaredoxin GrlA](https://rast.nmpdr.org/seedviewer.cgi?page=FunctionalRole&role=Probable%20monothiol%20glutaredoxin%20GrlA&subsystem_name=Glutaredoxins) |
| Stress Response | Oxidative stress | [Glutaredoxins](https://rast.nmpdr.org/seedviewer.cgi?page=Subsystems&subsystem=Glutaredoxins&organism=549.408) | [Glutaredoxin-like protein NrdH, required for reduction of Ribonucleotide reductase class Ib](https://rast.nmpdr.org/seedviewer.cgi?page=FunctionalRole&role=Glutaredoxin-like%20protein%20NrdH,%20required%20for%20reduction%20of%20Ribonucleotide%20reductase%20class%20Ib&subsystem_name=Glutaredoxins) |
| Stress Response | Oxidative stress | [Glutaredoxins](https://rast.nmpdr.org/seedviewer.cgi?page=Subsystems&subsystem=Glutaredoxins&organism=549.408) | [Glutaredoxin 3 (Grx3)](https://rast.nmpdr.org/seedviewer.cgi?page=FunctionalRole&role=Glutaredoxin%203%20(Grx3)&subsystem_name=Glutaredoxins) |
| Stress Response | Oxidative stress | [Glutaredoxins](https://rast.nmpdr.org/seedviewer.cgi?page=Subsystems&subsystem=Glutaredoxins&organism=549.408) | [Glutaredoxin 1](https://rast.nmpdr.org/seedviewer.cgi?page=FunctionalRole&role=Glutaredoxin%201&subsystem_name=Glutaredoxins) |
| Stress Response | Oxidative stress | [Glutaredoxins](https://rast.nmpdr.org/seedviewer.cgi?page=Subsystems&subsystem=Glutaredoxins&organism=549.408) | [Glutaredoxin 2](https://rast.nmpdr.org/seedviewer.cgi?page=FunctionalRole&role=Glutaredoxin%202&subsystem_name=Glutaredoxins) |
| Stress Response | Oxidative stress | [Glutaredoxins](https://rast.nmpdr.org/seedviewer.cgi?page=Subsystems&subsystem=Glutaredoxins&organism=549.408) | [Cell wall endopeptidase, family M23/M37](https://rast.nmpdr.org/seedviewer.cgi?page=FunctionalRole&role=Cell%20wall%20endopeptidase,%20family%20M23/M37&subsystem_name=Glutaredoxins) |
| Stress Response | Oxidative stress | [Glutathione: Biosynthesis and gamma-glutamyl cycle](https://rast.nmpdr.org/seedviewer.cgi?page=Subsystems&subsystem=Glutathione:_Biosynthesis_and_gamma-glutamyl_cycle&organism=549.408) | [Gamma-glutamyltranspeptidase (EC 2.3.2.2)](https://rast.nmpdr.org/seedviewer.cgi?page=FunctionalRole&role=Gamma-glutamyltranspeptidase%20(EC%202.3.2.2)&subsystem_name=Glutathione:_Biosynthesis_and_gamma-glutamyl_cycle) |
| Stress Response | Oxidative stress | [Glutathione: Biosynthesis and gamma-glutamyl cycle](https://rast.nmpdr.org/seedviewer.cgi?page=Subsystems&subsystem=Glutathione:_Biosynthesis_and_gamma-glutamyl_cycle&organism=549.408) | [Glutathione synthetase (EC 6.3.2.3)](https://rast.nmpdr.org/seedviewer.cgi?page=FunctionalRole&role=Glutathione%20synthetase%20(EC%206.3.2.3)&subsystem_name=Glutathione:_Biosynthesis_and_gamma-glutamyl_cycle) |
| Stress Response | Oxidative stress | [Glutathione: Biosynthesis and gamma-glutamyl cycle](https://rast.nmpdr.org/seedviewer.cgi?page=Subsystems&subsystem=Glutathione:_Biosynthesis_and_gamma-glutamyl_cycle&organism=549.408) | [Glutamate--cysteine ligase (EC 6.3.2.2)](https://rast.nmpdr.org/seedviewer.cgi?page=FunctionalRole&role=Glutamate--cysteine%20ligase%20(EC%206.3.2.2)&subsystem_name=Glutathione:_Biosynthesis_and_gamma-glutamyl_cycle) |
| Stress Response | Oxidative stress | [Oxidative stress](https://rast.nmpdr.org/seedviewer.cgi?page=Subsystems&subsystem=Oxidative_stress&organism=549.408) | [Regulatory protein SoxS](https://rast.nmpdr.org/seedviewer.cgi?page=FunctionalRole&role=Regulatory%20protein%20SoxS&subsystem_name=Oxidative_stress) |
| Stress Response | Oxidative stress | [Oxidative stress](https://rast.nmpdr.org/seedviewer.cgi?page=Subsystems&subsystem=Oxidative_stress&organism=549.408) | [Iron-binding ferritin-like antioxidant protein](https://rast.nmpdr.org/seedviewer.cgi?page=FunctionalRole&role=Iron-binding%20ferritin-like%20antioxidant%20protein&subsystem_name=Oxidative_stress) |
| Stress Response | Oxidative stress | [Oxidative stress](https://rast.nmpdr.org/seedviewer.cgi?page=Subsystems&subsystem=Oxidative_stress&organism=549.408) | [Redox-sensitive transcriptional activator SoxR](https://rast.nmpdr.org/seedviewer.cgi?page=FunctionalRole&role=Redox-sensitive%20transcriptional%20activator%20SoxR&subsystem_name=Oxidative_stress) |
| Stress Response | Oxidative stress | [Oxidative stress](https://rast.nmpdr.org/seedviewer.cgi?page=Subsystems&subsystem=Oxidative_stress&organism=549.408) | [Alkyl hydroperoxide reductase subunit C-like protein](https://rast.nmpdr.org/seedviewer.cgi?page=FunctionalRole&role=Alkyl%20hydroperoxide%20reductase%20subunit%20C-like%20protein&subsystem_name=Oxidative_stress) |
| Stress Response | Oxidative stress | [Oxidative stress](https://rast.nmpdr.org/seedviewer.cgi?page=Subsystems&subsystem=Oxidative_stress&organism=549.408) | [Manganese superoxide dismutase (EC 1.15.1.1)](https://rast.nmpdr.org/seedviewer.cgi?page=FunctionalRole&role=Manganese%20superoxide%20dismutase%20(EC%201.15.1.1)&subsystem_name=Oxidative_stress) |
| Stress Response | Oxidative stress | [Oxidative stress](https://rast.nmpdr.org/seedviewer.cgi?page=Subsystems&subsystem=Oxidative_stress&organism=549.408) | [Superoxide dismutase [Cu-Zn] precursor (EC 1.15.1.1)](https://rast.nmpdr.org/seedviewer.cgi?page=FunctionalRole&role=Superoxide%20dismutase%20%5bCu-Zn%5d%20precursor%20(EC%201.15.1.1)&subsystem_name=Oxidative_stress) |
| Stress Response | Oxidative stress | [Oxidative stress](https://rast.nmpdr.org/seedviewer.cgi?page=Subsystems&subsystem=Oxidative_stress&organism=549.408) | [Paraquat-inducible protein B](https://rast.nmpdr.org/seedviewer.cgi?page=FunctionalRole&role=Paraquat-inducible%20protein%20B&subsystem_name=Oxidative_stress) |
| Stress Response | Oxidative stress | [Oxidative stress](https://rast.nmpdr.org/seedviewer.cgi?page=Subsystems&subsystem=Oxidative_stress&organism=549.408) | [Hydrogen peroxide-inducible genes activator](https://rast.nmpdr.org/seedviewer.cgi?page=FunctionalRole&role=Hydrogen%20peroxide-inducible%20genes%20activator&subsystem_name=Oxidative_stress) |
| Stress Response | Oxidative stress | [Oxidative stress](https://rast.nmpdr.org/seedviewer.cgi?page=Subsystems&subsystem=Oxidative_stress&organism=549.408) | [Nitrite-sensitive transcriptional repressor NsrR](https://rast.nmpdr.org/seedviewer.cgi?page=FunctionalRole&role=Nitrite-sensitive%20transcriptional%20repressor%20NsrR&subsystem_name=Oxidative_stress) |
| Stress Response | Oxidative stress | [Oxidative stress](https://rast.nmpdr.org/seedviewer.cgi?page=Subsystems&subsystem=Oxidative_stress&organism=549.408) | [Catalase (EC 1.11.1.6)](https://rast.nmpdr.org/seedviewer.cgi?page=FunctionalRole&role=Catalase%20(EC%201.11.1.6)&subsystem_name=Oxidative_stress) |
| Stress Response | Oxidative stress | [Oxidative stress](https://rast.nmpdr.org/seedviewer.cgi?page=Subsystems&subsystem=Oxidative_stress&organism=549.408) | [Fumarate and nitrate reduction regulatory protein](https://rast.nmpdr.org/seedviewer.cgi?page=FunctionalRole&role=Fumarate%20and%20nitrate%20reduction%20regulatory%20protein&subsystem_name=Oxidative_stress) |
| Stress Response | Oxidative stress | [Oxidative stress](https://rast.nmpdr.org/seedviewer.cgi?page=Subsystems&subsystem=Oxidative_stress&organism=549.408) | [Organic hydroperoxide resistance protein](https://rast.nmpdr.org/seedviewer.cgi?page=FunctionalRole&role=Organic%20hydroperoxide%20resistance%20protein&subsystem_name=Oxidative_stress) |
| Stress Response | Oxidative stress | [Oxidative stress](https://rast.nmpdr.org/seedviewer.cgi?page=Subsystems&subsystem=Oxidative_stress&organism=549.408) | [Organic hydroperoxide resistance transcriptional regulator](https://rast.nmpdr.org/seedviewer.cgi?page=FunctionalRole&role=Organic%20hydroperoxide%20resistance%20transcriptional%20regulator&subsystem_name=Oxidative_stress) |
| Stress Response | Oxidative stress | [Oxidative stress](https://rast.nmpdr.org/seedviewer.cgi?page=Subsystems&subsystem=Oxidative_stress&organism=549.408) | [Zinc uptake regulation protein ZUR](https://rast.nmpdr.org/seedviewer.cgi?page=FunctionalRole&role=Zinc%20uptake%20regulation%20protein%20ZUR&subsystem_name=Oxidative_stress) |
| Stress Response | Oxidative stress | [Oxidative stress](https://rast.nmpdr.org/seedviewer.cgi?page=Subsystems&subsystem=Oxidative_stress&organism=549.408) | [Non-specific DNA-binding protein Dps](https://rast.nmpdr.org/seedviewer.cgi?page=FunctionalRole&role=Non-specific%20DNA-binding%20protein%20Dps&subsystem_name=Oxidative_stress) |
| Stress Response | Oxidative stress | [Oxidative stress](https://rast.nmpdr.org/seedviewer.cgi?page=Subsystems&subsystem=Oxidative_stress&organism=549.408) | [Ferroxidase (EC 1.16.3.1)](https://rast.nmpdr.org/seedviewer.cgi?page=FunctionalRole&role=Ferroxidase%20(EC%201.16.3.1)&subsystem_name=Oxidative_stress) |
| Stress Response | Oxidative stress | [Oxidative stress](https://rast.nmpdr.org/seedviewer.cgi?page=Subsystems&subsystem=Oxidative_stress&organism=549.408) | [Ferric uptake regulation protein FUR](https://rast.nmpdr.org/seedviewer.cgi?page=FunctionalRole&role=Ferric%20uptake%20regulation%20protein%20FUR&subsystem_name=Oxidative_stress) |
| Stress Response | Oxidative stress | [Oxidative stress](https://rast.nmpdr.org/seedviewer.cgi?page=Subsystems&subsystem=Oxidative_stress&organism=549.408) | [Peroxidase (EC 1.11.1.7)](https://rast.nmpdr.org/seedviewer.cgi?page=FunctionalRole&role=Peroxidase%20(EC%201.11.1.7)&subsystem_name=Oxidative_stress) |
| Stress Response | Oxidative stress | [Oxidative stress](https://rast.nmpdr.org/seedviewer.cgi?page=Subsystems&subsystem=Oxidative_stress&organism=549.408) | [Paraquat-inducible protein A](https://rast.nmpdr.org/seedviewer.cgi?page=FunctionalRole&role=Paraquat-inducible%20protein%20A&subsystem_name=Oxidative_stress) |
| Stress Response | Oxidative stress | [Protection from Reactive Oxygen Species](https://rast.nmpdr.org/seedviewer.cgi?page=Subsystems&subsystem=Protection_from_Reactive_Oxygen_Species&organism=549.408) | [Peroxidase (EC 1.11.1.7)](https://rast.nmpdr.org/seedviewer.cgi?page=FunctionalRole&role=Peroxidase%20(EC%201.11.1.7)&subsystem_name=Protection_from_Reactive_Oxygen_Species) |
| Stress Response | Oxidative stress | [Protection from Reactive Oxygen Species](https://rast.nmpdr.org/seedviewer.cgi?page=Subsystems&subsystem=Protection_from_Reactive_Oxygen_Species&organism=549.408) | [Manganese superoxide dismutase (EC 1.15.1.1)](https://rast.nmpdr.org/seedviewer.cgi?page=FunctionalRole&role=Manganese%20superoxide%20dismutase%20(EC%201.15.1.1)&subsystem_name=Protection_from_Reactive_Oxygen_Species) |
| Stress Response | Oxidative stress | [Protection from Reactive Oxygen Species](https://rast.nmpdr.org/seedviewer.cgi?page=Subsystems&subsystem=Protection_from_Reactive_Oxygen_Species&organism=549.408) | [Superoxide dismutase [Cu-Zn] precursor (EC 1.15.1.1)](https://rast.nmpdr.org/seedviewer.cgi?page=FunctionalRole&role=Superoxide%20dismutase%20%5bCu-Zn%5d%20precursor%20(EC%201.15.1.1)&subsystem_name=Protection_from_Reactive_Oxygen_Species) |
| Stress Response | Oxidative stress | [Protection from Reactive Oxygen Species](https://rast.nmpdr.org/seedviewer.cgi?page=Subsystems&subsystem=Protection_from_Reactive_Oxygen_Species&organism=549.408) | [Catalase (EC 1.11.1.6)](https://rast.nmpdr.org/seedviewer.cgi?page=FunctionalRole&role=Catalase%20(EC%201.11.1.6)&subsystem_name=Protection_from_Reactive_Oxygen_Species) |
| Stress Response | Oxidative stress | [Glutathionylspermidine and Trypanothione](https://rast.nmpdr.org/seedviewer.cgi?page=Subsystems&subsystem=Glutathionylspermidine_and_Trypanothione&organism=549.408) | [Similarity with glutathionylspermidine synthase (EC 6.3.1.8), group 1](https://rast.nmpdr.org/seedviewer.cgi?page=FunctionalRole&role=Similarity%20with%20glutathionylspermidine%20synthase%20(EC%206.3.1.8),%20group%201&subsystem_name=Glutathionylspermidine_and_Trypanothione) |
| Stress Response | Cold shock | [Cold shock, CspA family of proteins](https://rast.nmpdr.org/seedviewer.cgi?page=Subsystems&subsystem=Cold_shock,_CspA_family_of_proteins&organism=549.408) | [Cold shock protein CspG](https://rast.nmpdr.org/seedviewer.cgi?page=FunctionalRole&role=Cold%20shock%20protein%20CspG&subsystem_name=Cold_shock,_CspA_family_of_proteins) |
| Stress Response | Cold shock | [Cold shock, CspA family of proteins](https://rast.nmpdr.org/seedviewer.cgi?page=Subsystems&subsystem=Cold_shock,_CspA_family_of_proteins&organism=549.408) | [Cold shock protein CspD](https://rast.nmpdr.org/seedviewer.cgi?page=FunctionalRole&role=Cold%20shock%20protein%20CspD&subsystem_name=Cold_shock,_CspA_family_of_proteins) |
| Stress Response | Cold shock | [Cold shock, CspA family of proteins](https://rast.nmpdr.org/seedviewer.cgi?page=Subsystems&subsystem=Cold_shock,_CspA_family_of_proteins&organism=549.408) | [Cold shock protein CspA](https://rast.nmpdr.org/seedviewer.cgi?page=FunctionalRole&role=Cold%20shock%20protein%20CspA&subsystem_name=Cold_shock,_CspA_family_of_proteins) |
| Stress Response | Cold shock | [Cold shock, CspA family of proteins](https://rast.nmpdr.org/seedviewer.cgi?page=Subsystems&subsystem=Cold_shock,_CspA_family_of_proteins&organism=549.408) | [Cold shock protein CspE](https://rast.nmpdr.org/seedviewer.cgi?page=FunctionalRole&role=Cold%20shock%20protein%20CspE&subsystem_name=Cold_shock,_CspA_family_of_proteins) |
| Stress Response | Heat shock | [Heat shock dnaK gene cluster extended](https://rast.nmpdr.org/seedviewer.cgi?page=Subsystems&subsystem=Heat_shock_dnaK_gene_cluster_extended&organism=549.408) | [FIG001341: Probable Fe(2+)-trafficking protein YggX](https://rast.nmpdr.org/seedviewer.cgi?page=FunctionalRole&role=FIG001341:%20Probable%20Fe(2+)-trafficking%20protein%20YggX&subsystem_name=Heat_shock_dnaK_gene_cluster_extended) |
| Stress Response | Heat shock | [Heat shock dnaK gene cluster extended](https://rast.nmpdr.org/seedviewer.cgi?page=Subsystems&subsystem=Heat_shock_dnaK_gene_cluster_extended&organism=549.408) | [Chaperone protein DnaK](https://rast.nmpdr.org/seedviewer.cgi?page=FunctionalRole&role=Chaperone%20protein%20DnaK&subsystem_name=Heat_shock_dnaK_gene_cluster_extended) |
| Stress Response | Heat shock | [Heat shock dnaK gene cluster extended](https://rast.nmpdr.org/seedviewer.cgi?page=Subsystems&subsystem=Heat_shock_dnaK_gene_cluster_extended&organism=549.408) | [Glutathione synthetase (EC 6.3.2.3)](https://rast.nmpdr.org/seedviewer.cgi?page=FunctionalRole&role=Glutathione%20synthetase%20(EC%206.3.2.3)&subsystem_name=Heat_shock_dnaK_gene_cluster_extended) |
| Stress Response | Heat shock | [Heat shock dnaK gene cluster extended](https://rast.nmpdr.org/seedviewer.cgi?page=Subsystems&subsystem=Heat_shock_dnaK_gene_cluster_extended&organism=549.408) | [Chaperone protein DnaJ](https://rast.nmpdr.org/seedviewer.cgi?page=FunctionalRole&role=Chaperone%20protein%20DnaJ&subsystem_name=Heat_shock_dnaK_gene_cluster_extended) |
| Stress Response | Heat shock | [Heat shock dnaK gene cluster extended](https://rast.nmpdr.org/seedviewer.cgi?page=Subsystems&subsystem=Heat_shock_dnaK_gene_cluster_extended&organism=549.408) | [Ribosomal RNA small subunit methyltransferase E (EC 2.1.1.-)](https://rast.nmpdr.org/seedviewer.cgi?page=FunctionalRole&role=Ribosomal%20RNA%20small%20subunit%20methyltransferase%20E%20(EC%202.1.1.-)&subsystem_name=Heat_shock_dnaK_gene_cluster_extended) |
| Stress Response | Heat shock | [Heat shock dnaK gene cluster extended](https://rast.nmpdr.org/seedviewer.cgi?page=Subsystems&subsystem=Heat_shock_dnaK_gene_cluster_extended&organism=549.408) | [tmRNA-binding protein SmpB](https://rast.nmpdr.org/seedviewer.cgi?page=FunctionalRole&role=tmRNA-binding%20protein%20SmpB&subsystem_name=Heat_shock_dnaK_gene_cluster_extended) |
| Stress Response | Heat shock | [Heat shock dnaK gene cluster extended](https://rast.nmpdr.org/seedviewer.cgi?page=Subsystems&subsystem=Heat_shock_dnaK_gene_cluster_extended&organism=549.408) | [Radical SAM family enzyme, similar to coproporphyrinogen III oxidase, oxygen-independent, clustered with nucleoside-triphosphatase RdgB](https://rast.nmpdr.org/seedviewer.cgi?page=FunctionalRole&role=Radical%20SAM%20family%20enzyme,%20similar%20to%20coproporphyrinogen%20III%20oxidase,%20oxygen-independent,%20clustered%20with%20nucleoside-triphosphatase%20RdgB&subsystem_name=Heat_shock_dnaK_gene_cluster_extended) |
| Stress Response | Heat shock | [Heat shock dnaK gene cluster extended](https://rast.nmpdr.org/seedviewer.cgi?page=Subsystems&subsystem=Heat_shock_dnaK_gene_cluster_extended&organism=549.408) | [Heat shock protein GrpE](https://rast.nmpdr.org/seedviewer.cgi?page=FunctionalRole&role=Heat%20shock%20protein%20GrpE&subsystem_name=Heat_shock_dnaK_gene_cluster_extended) |
| Stress Response | Heat shock | [Heat shock dnaK gene cluster extended](https://rast.nmpdr.org/seedviewer.cgi?page=Subsystems&subsystem=Heat_shock_dnaK_gene_cluster_extended&organism=549.408) | [RNA polymerase sigma factor RpoH](https://rast.nmpdr.org/seedviewer.cgi?page=FunctionalRole&role=RNA%20polymerase%20sigma%20factor%20RpoH&subsystem_name=Heat_shock_dnaK_gene_cluster_extended) |
| Stress Response | Heat shock | [Heat shock dnaK gene cluster extended](https://rast.nmpdr.org/seedviewer.cgi?page=Subsystems&subsystem=Heat_shock_dnaK_gene_cluster_extended&organism=549.408) | [Translation elongation factor LepA](https://rast.nmpdr.org/seedviewer.cgi?page=FunctionalRole&role=Translation%20elongation%20factor%20LepA&subsystem_name=Heat_shock_dnaK_gene_cluster_extended) |
| Stress Response | Heat shock | [Heat shock dnaK gene cluster extended](https://rast.nmpdr.org/seedviewer.cgi?page=Subsystems&subsystem=Heat_shock_dnaK_gene_cluster_extended&organism=549.408) | [Ribosome-associated heat shock protein implicated in the recycling of the 50S subunit (S4 paralog)](https://rast.nmpdr.org/seedviewer.cgi?page=FunctionalRole&role=Ribosome-associated%20heat%20shock%20protein%20implicated%20in%20the%20recycling%20of%20the%2050S%20subunit%20(S4%20paralog)&subsystem_name=Heat_shock_dnaK_gene_cluster_extended) |
| Stress Response | Heat shock | [Heat shock dnaK gene cluster extended](https://rast.nmpdr.org/seedviewer.cgi?page=Subsystems&subsystem=Heat_shock_dnaK_gene_cluster_extended&organism=549.408) | [Nucleoside 5-triphosphatase RdgB (dHAPTP, dITP, XTP-specific) (EC 3.6.1.15)](https://rast.nmpdr.org/seedviewer.cgi?page=FunctionalRole&role=Nucleoside%205-triphosphatase%20RdgB%20(dHAPTP,%20dITP,%20XTP-specific)%20(EC%203.6.1.15)&subsystem_name=Heat_shock_dnaK_gene_cluster_extended) |
| Stress Response | Heat shock | [Heat shock dnaK gene cluster extended](https://rast.nmpdr.org/seedviewer.cgi?page=Subsystems&subsystem=Heat_shock_dnaK_gene_cluster_extended&organism=549.408) | [Ribosomal protein L11 methyltransferase (EC 2.1.1.-)](https://rast.nmpdr.org/seedviewer.cgi?page=FunctionalRole&role=Ribosomal%20protein%20L11%20methyltransferase%20(EC%202.1.1.-)&subsystem_name=Heat_shock_dnaK_gene_cluster_extended) |
| Stress Response | Heat shock | [Heat shock dnaK gene cluster extended](https://rast.nmpdr.org/seedviewer.cgi?page=Subsystems&subsystem=Heat_shock_dnaK_gene_cluster_extended&organism=549.408) | [Ribonuclease PH (EC 2.7.7.56)](https://rast.nmpdr.org/seedviewer.cgi?page=FunctionalRole&role=Ribonuclease%20PH%20(EC%202.7.7.56)&subsystem_name=Heat_shock_dnaK_gene_cluster_extended) |
| Stress Response | Heat shock | [Heat shock dnaK gene cluster extended](https://rast.nmpdr.org/seedviewer.cgi?page=Subsystems&subsystem=Heat_shock_dnaK_gene_cluster_extended&organism=549.408) | [rRNA small subunit methyltransferase I](https://rast.nmpdr.org/seedviewer.cgi?page=FunctionalRole&role=rRNA%20small%20subunit%20methyltransferase%20I&subsystem_name=Heat_shock_dnaK_gene_cluster_extended) |
| Stress Response | Detoxification | [Uptake of selenate and selenite](https://rast.nmpdr.org/seedviewer.cgi?page=Subsystems&subsystem=Uptake_of_selenate_and_selenite&organism=549.408) | [TsgA protein homolog](https://rast.nmpdr.org/seedviewer.cgi?page=FunctionalRole&role=TsgA%20protein%20homolog&subsystem_name=Uptake_of_selenate_and_selenite) |
| Stress Response | Detoxification | [Uptake of selenate and selenite](https://rast.nmpdr.org/seedviewer.cgi?page=Subsystems&subsystem=Uptake_of_selenate_and_selenite&organism=549.408) | [Sulfate and thiosulfate import ATP-binding protein CysA (EC 3.6.3.25)](https://rast.nmpdr.org/seedviewer.cgi?page=FunctionalRole&role=Sulfate%20and%20thiosulfate%20import%20ATP-binding%20protein%20CysA%20(EC%203.6.3.25)&subsystem_name=Uptake_of_selenate_and_selenite) |
| Stress Response | Detoxification | [Uptake of selenate and selenite](https://rast.nmpdr.org/seedviewer.cgi?page=Subsystems&subsystem=Uptake_of_selenate_and_selenite&organism=549.408) | [DedA protein](https://rast.nmpdr.org/seedviewer.cgi?page=FunctionalRole&role=DedA%20protein&subsystem_name=Uptake_of_selenate_and_selenite) |
| Stress Response | Detoxification | [Tellurite resistance: Chromosomal determinants](https://rast.nmpdr.org/seedviewer.cgi?page=Subsystems&subsystem=Tellurite_resistance:_Chromosomal_determinants&organism=549.408) | [Tellurite resistance protein TehB](https://rast.nmpdr.org/seedviewer.cgi?page=FunctionalRole&role=Tellurite%20resistance%20protein%20TehB&subsystem_name=Tellurite_resistance:_Chromosomal_determinants) |
| Stress Response | Detoxification | [Glutathione-dependent pathway of formaldehyde detoxification](https://rast.nmpdr.org/seedviewer.cgi?page=Subsystems&subsystem=Glutathione-dependent_pathway_of_formaldehyde_detoxification&organism=549.408) | [S-formylglutathione hydrolase (EC 3.1.2.12)](https://rast.nmpdr.org/seedviewer.cgi?page=FunctionalRole&role=S-formylglutathione%20hydrolase%20(EC%203.1.2.12)&subsystem_name=Glutathione-dependent_pathway_of_formaldehyde_detoxification) |
| Stress Response | Detoxification | [Glutathione-dependent pathway of formaldehyde detoxification](https://rast.nmpdr.org/seedviewer.cgi?page=Subsystems&subsystem=Glutathione-dependent_pathway_of_formaldehyde_detoxification&organism=549.408) | [S-(hydroxymethyl)glutathione dehydrogenase (EC 1.1.1.284)](https://rast.nmpdr.org/seedviewer.cgi?page=FunctionalRole&role=S-(hydroxymethyl)glutathione%20dehydrogenase%20(EC%201.1.1.284)&subsystem_name=Glutathione-dependent_pathway_of_formaldehyde_detoxification) |
| Stress Response | Stress Response - no subcategory | [Flavohaemoglobin](https://rast.nmpdr.org/seedviewer.cgi?page=Subsystems&subsystem=Flavohaemoglobin&organism=549.408) | [Flavohemoprotein (Hemoglobin-like protein) (Flavohemoglobin) (Nitric oxide dioxygenase) (EC 1.14.12.17)](https://rast.nmpdr.org/seedviewer.cgi?page=FunctionalRole&role=Flavohemoprotein%20(Hemoglobin-like%20protein)%20(Flavohemoglobin)%20(Nitric%20oxide%20dioxygenase)%20(EC%201.14.12.17)&subsystem_name=Flavohaemoglobin) |
| Stress Response | Stress Response - no subcategory | [Universal stress protein family](https://rast.nmpdr.org/seedviewer.cgi?page=Subsystems&subsystem=Universal_stress_protein_family&organism=549.408) | [Universal stress protein E](https://rast.nmpdr.org/seedviewer.cgi?page=FunctionalRole&role=Universal%20stress%20protein%20E&subsystem_name=Universal_stress_protein_family) |
| Stress Response | Stress Response - no subcategory | [Universal stress protein family](https://rast.nmpdr.org/seedviewer.cgi?page=Subsystems&subsystem=Universal_stress_protein_family&organism=549.408) | [Universal stress protein B](https://rast.nmpdr.org/seedviewer.cgi?page=FunctionalRole&role=Universal%20stress%20protein%20B&subsystem_name=Universal_stress_protein_family) |
| Stress Response | Stress Response - no subcategory | [Universal stress protein family](https://rast.nmpdr.org/seedviewer.cgi?page=Subsystems&subsystem=Universal_stress_protein_family&organism=549.408) | [Universal stress protein A](https://rast.nmpdr.org/seedviewer.cgi?page=FunctionalRole&role=Universal%20stress%20protein%20A&subsystem_name=Universal_stress_protein_family) |
| Stress Response | Stress Response - no subcategory | [Phage shock protein (psp) operon](https://rast.nmpdr.org/seedviewer.cgi?page=Subsystems&subsystem=Phage_shock_protein_(psp)_operon&organism=549.408) | [Phage shock protein D](https://rast.nmpdr.org/seedviewer.cgi?page=FunctionalRole&role=Phage%20shock%20protein%20D&subsystem_name=Phage_shock_protein_(psp)_operon) |
| Stress Response | Stress Response - no subcategory | [Phage shock protein (psp) operon](https://rast.nmpdr.org/seedviewer.cgi?page=Subsystems&subsystem=Phage_shock_protein_(psp)_operon&organism=549.408) | [Psp operon transcriptional activator](https://rast.nmpdr.org/seedviewer.cgi?page=FunctionalRole&role=Psp%20operon%20transcriptional%20activator&subsystem_name=Phage_shock_protein_(psp)_operon) |
| Stress Response | Stress Response - no subcategory | [Phage shock protein (psp) operon](https://rast.nmpdr.org/seedviewer.cgi?page=Subsystems&subsystem=Phage_shock_protein_(psp)_operon&organism=549.408) | [Phage shock protein B](https://rast.nmpdr.org/seedviewer.cgi?page=FunctionalRole&role=Phage%20shock%20protein%20B&subsystem_name=Phage_shock_protein_(psp)_operon) |
| Stress Response | Stress Response - no subcategory | [Phage shock protein (psp) operon](https://rast.nmpdr.org/seedviewer.cgi?page=Subsystems&subsystem=Phage_shock_protein_(psp)_operon&organism=549.408) | [Phage shock protein C](https://rast.nmpdr.org/seedviewer.cgi?page=FunctionalRole&role=Phage%20shock%20protein%20C&subsystem_name=Phage_shock_protein_(psp)_operon) |
| Stress Response | Stress Response - no subcategory | [Phage shock protein (psp) operon](https://rast.nmpdr.org/seedviewer.cgi?page=Subsystems&subsystem=Phage_shock_protein_(psp)_operon&organism=549.408) | [Phage shock protein A](https://rast.nmpdr.org/seedviewer.cgi?page=FunctionalRole&role=Phage%20shock%20protein%20A&subsystem_name=Phage_shock_protein_(psp)_operon) |
| Stress Response | Stress Response - no subcategory | [Sugar-phosphate stress regulation](https://rast.nmpdr.org/seedviewer.cgi?page=Subsystems&subsystem=Sugar-phosphate_stress_regulation&organism=549.408) | [SgrR, sugar-phosphate stress, transcriptional activator of SgrS small RNA](https://rast.nmpdr.org/seedviewer.cgi?page=FunctionalRole&role=SgrR,%20sugar-phosphate%20stress,%20transcriptional%20activator%20of%20SgrS%20small%20RNA&subsystem_name=Sugar-phosphate_stress_regulation) |
| Stress Response | Stress Response - no subcategory | [Hfl operon](https://rast.nmpdr.org/seedviewer.cgi?page=Subsystems&subsystem=Hfl_operon&organism=549.408) | [Putative inner membrane protein YjeT (clustered with HflC)](https://rast.nmpdr.org/seedviewer.cgi?page=FunctionalRole&role=Putative%20inner%20membrane%20protein%20YjeT%20(clustered%20with%20HflC)&subsystem_name=Hfl_operon) |
| Stress Response | Stress Response - no subcategory | [Hfl operon](https://rast.nmpdr.org/seedviewer.cgi?page=Subsystems&subsystem=Hfl_operon&organism=549.408) | [GTP-binding protein HflX](https://rast.nmpdr.org/seedviewer.cgi?page=FunctionalRole&role=GTP-binding%20protein%20HflX&subsystem_name=Hfl_operon) |
| Stress Response | Stress Response - no subcategory | [Hfl operon](https://rast.nmpdr.org/seedviewer.cgi?page=Subsystems&subsystem=Hfl_operon&organism=549.408) | [HflC protein](https://rast.nmpdr.org/seedviewer.cgi?page=FunctionalRole&role=HflC%20protein&subsystem_name=Hfl_operon) |
| Stress Response | Stress Response - no subcategory | [Hfl operon](https://rast.nmpdr.org/seedviewer.cgi?page=Subsystems&subsystem=Hfl_operon&organism=549.408) | [RNA-binding protein Hfq](https://rast.nmpdr.org/seedviewer.cgi?page=FunctionalRole&role=RNA-binding%20protein%20Hfq&subsystem_name=Hfl_operon) |
| Stress Response | Stress Response - no subcategory | [Hfl operon](https://rast.nmpdr.org/seedviewer.cgi?page=Subsystems&subsystem=Hfl_operon&organism=549.408) | [HflK protein](https://rast.nmpdr.org/seedviewer.cgi?page=FunctionalRole&role=HflK%20protein&subsystem_name=Hfl_operon) |
| Stress Response | Stress Response - no subcategory | [Carbon Starvation](https://rast.nmpdr.org/seedviewer.cgi?page=Subsystems&subsystem=Carbon_Starvation&organism=549.408) | [Carbon storage regulator](https://rast.nmpdr.org/seedviewer.cgi?page=FunctionalRole&role=Carbon%20storage%20regulator&subsystem_name=Carbon_Starvation) |
| Stress Response | Stress Response - no subcategory | [Carbon Starvation](https://rast.nmpdr.org/seedviewer.cgi?page=Subsystems&subsystem=Carbon_Starvation&organism=549.408) | [Starvation sensing protein RspA](https://rast.nmpdr.org/seedviewer.cgi?page=FunctionalRole&role=Starvation%20sensing%20protein%20RspA&subsystem_name=Carbon_Starvation) |
| Stress Response | Stress Response - no subcategory | [Carbon Starvation](https://rast.nmpdr.org/seedviewer.cgi?page=Subsystems&subsystem=Carbon_Starvation&organism=549.408) | [Starvation lipoprotein Slp paralog](https://rast.nmpdr.org/seedviewer.cgi?page=FunctionalRole&role=Starvation%20lipoprotein%20Slp%20paralog&subsystem_name=Carbon_Starvation) |
| Stress Response | Stress Response - no subcategory | [Carbon Starvation](https://rast.nmpdr.org/seedviewer.cgi?page=Subsystems&subsystem=Carbon_Starvation&organism=549.408) | [Carbon starvation protein A paralog](https://rast.nmpdr.org/seedviewer.cgi?page=FunctionalRole&role=Carbon%20starvation%20protein%20A%20paralog&subsystem_name=Carbon_Starvation) |
| Stress Response | Stress Response - no subcategory | [Carbon Starvation](https://rast.nmpdr.org/seedviewer.cgi?page=Subsystems&subsystem=Carbon_Starvation&organism=549.408) | [Stringent starvation protein A](https://rast.nmpdr.org/seedviewer.cgi?page=FunctionalRole&role=Stringent%20starvation%20protein%20A&subsystem_name=Carbon_Starvation) |
| Stress Response | Stress Response - no subcategory | [Carbon Starvation](https://rast.nmpdr.org/seedviewer.cgi?page=Subsystems&subsystem=Carbon_Starvation&organism=549.408) | [Starvation sensing protein RspB](https://rast.nmpdr.org/seedviewer.cgi?page=FunctionalRole&role=Starvation%20sensing%20protein%20RspB&subsystem_name=Carbon_Starvation) |
| Stress Response | Stress Response - no subcategory | [Carbon Starvation](https://rast.nmpdr.org/seedviewer.cgi?page=Subsystems&subsystem=Carbon_Starvation&organism=549.408) | [Stringent starvation protein B](https://rast.nmpdr.org/seedviewer.cgi?page=FunctionalRole&role=Stringent%20starvation%20protein%20B&subsystem_name=Carbon_Starvation) |
| Stress Response | Stress Response - no subcategory | [Bacterial hemoglobins](https://rast.nmpdr.org/seedviewer.cgi?page=Subsystems&subsystem=Bacterial_hemoglobins&organism=549.408) | [diguanylate cyclase/phosphodiesterase (GGDEF & EAL domains) with PAS/PAC sensor(s)](https://rast.nmpdr.org/seedviewer.cgi?page=FunctionalRole&role=diguanylate%20cyclase/phosphodiesterase%20(GGDEF%20&%20EAL%20domains)%20with%20PAS/PAC%20sensor(s)&subsystem_name=Bacterial_hemoglobins) |
| Stress Response | Stress Response - no subcategory | [Bacterial hemoglobins](https://rast.nmpdr.org/seedviewer.cgi?page=Subsystems&subsystem=Bacterial_hemoglobins&organism=549.408) | [Flavohemoprotein (Hemoglobin-like protein) (Flavohemoglobin) (Nitric oxide dioxygenase) (EC 1.14.12.17)](https://rast.nmpdr.org/seedviewer.cgi?page=FunctionalRole&role=Flavohemoprotein%20(Hemoglobin-like%20protein)%20(Flavohemoglobin)%20(Nitric%20oxide%20dioxygenase)%20(EC%201.14.12.17)&subsystem_name=Bacterial_hemoglobins) |
| Stress Response | Stress Response - no subcategory | [Commensurate regulon activation](https://rast.nmpdr.org/seedviewer.cgi?page=Subsystems&subsystem=Commensurate_regulon_activation&organism=549.408) | [Regulatory protein SoxS](https://rast.nmpdr.org/seedviewer.cgi?page=FunctionalRole&role=Regulatory%20protein%20SoxS&subsystem_name=Commensurate_regulon_activation) |
| Stress Response | Stress Response - no subcategory | [Commensurate regulon activation](https://rast.nmpdr.org/seedviewer.cgi?page=Subsystems&subsystem=Commensurate_regulon_activation&organism=549.408) | [Right origin-binding protein](https://rast.nmpdr.org/seedviewer.cgi?page=FunctionalRole&role=Right%20origin-binding%20protein&subsystem_name=Commensurate_regulon_activation) |
| Stress Response | Periplasmic Stress | [Periplasmic Stress Response](https://rast.nmpdr.org/seedviewer.cgi?page=Subsystems&subsystem=Periplasmic_Stress_Response&organism=549.408) | [Survival protein SurA precursor (Peptidyl-prolyl cis-trans isomerase SurA) (EC 5.2.1.8)](https://rast.nmpdr.org/seedviewer.cgi?page=FunctionalRole&role=Survival%20protein%20SurA%20precursor%20(Peptidyl-prolyl%20cis-trans%20isomerase%20SurA)%20(EC%205.2.1.8)&subsystem_name=Periplasmic_Stress_Response) |
| Stress Response | Periplasmic Stress | [Periplasmic Stress Response](https://rast.nmpdr.org/seedviewer.cgi?page=Subsystems&subsystem=Periplasmic_Stress_Response&organism=549.408) | [Sigma factor RpoE negative regulatory protein RseA](https://rast.nmpdr.org/seedviewer.cgi?page=FunctionalRole&role=Sigma%20factor%20RpoE%20negative%20regulatory%20protein%20RseA&subsystem_name=Periplasmic_Stress_Response) |
| Stress Response | Periplasmic Stress | [Periplasmic Stress Response](https://rast.nmpdr.org/seedviewer.cgi?page=Subsystems&subsystem=Periplasmic_Stress_Response&organism=549.408) | [Outer membrane stress sensor protease DegS](https://rast.nmpdr.org/seedviewer.cgi?page=FunctionalRole&role=Outer%20membrane%20stress%20sensor%20protease%20DegS&subsystem_name=Periplasmic_Stress_Response) |
| Stress Response | Periplasmic Stress | [Periplasmic Stress Response](https://rast.nmpdr.org/seedviewer.cgi?page=Subsystems&subsystem=Periplasmic_Stress_Response&organism=549.408) | [Outer membrane protein H precursor](https://rast.nmpdr.org/seedviewer.cgi?page=FunctionalRole&role=Outer%20membrane%20protein%20H%20precursor&subsystem_name=Periplasmic_Stress_Response) |
| Stress Response | Periplasmic Stress | [Periplasmic Stress Response](https://rast.nmpdr.org/seedviewer.cgi?page=Subsystems&subsystem=Periplasmic_Stress_Response&organism=549.408) | [Sigma factor RpoE negative regulatory protein RseB precursor](https://rast.nmpdr.org/seedviewer.cgi?page=FunctionalRole&role=Sigma%20factor%20RpoE%20negative%20regulatory%20protein%20RseB%20precursor&subsystem_name=Periplasmic_Stress_Response) |
| Stress Response | Periplasmic Stress | [Periplasmic Stress Response](https://rast.nmpdr.org/seedviewer.cgi?page=Subsystems&subsystem=Periplasmic_Stress_Response&organism=549.408) | [Outer membrane stress sensor protease DegQ, serine protease](https://rast.nmpdr.org/seedviewer.cgi?page=FunctionalRole&role=Outer%20membrane%20stress%20sensor%20protease%20DegQ,%20serine%20protease&subsystem_name=Periplasmic_Stress_Response) |
| Stress Response | Periplasmic Stress | [Periplasmic Stress Response](https://rast.nmpdr.org/seedviewer.cgi?page=Subsystems&subsystem=Periplasmic_Stress_Response&organism=549.408) | [HtrA protease/chaperone protein](https://rast.nmpdr.org/seedviewer.cgi?page=FunctionalRole&role=HtrA%20protease/chaperone%20protein&subsystem_name=Periplasmic_Stress_Response) |
| Metabolism of Aromatic Compounds | Peripheral pathways for catabolism of aromatic compounds | [Salicylate ester degradation](https://rast.nmpdr.org/seedviewer.cgi?page=Subsystems&subsystem=Salicylate_ester_degradation&organism=549.408) | [Salicylate hydroxylase (EC 1.14.13.1)](https://rast.nmpdr.org/seedviewer.cgi?page=FunctionalRole&role=Salicylate%20hydroxylase%20(EC%201.14.13.1)&subsystem_name=Salicylate_ester_degradation) |
| Metabolism of Aromatic Compounds | Peripheral pathways for catabolism of aromatic compounds | [Quinate degradation](https://rast.nmpdr.org/seedviewer.cgi?page=Subsystems&subsystem=Quinate_degradation&organism=549.408) | [Quinate/shikimate dehydrogenase [Pyrroloquinoline-quinone] (EC 1.1.99.25)](https://rast.nmpdr.org/seedviewer.cgi?page=FunctionalRole&role=Quinate/shikimate%20dehydrogenase%20%5bPyrroloquinoline-quinone%5d%20(EC%201.1.99.25)&subsystem_name=Quinate_degradation) |
| Metabolism of Aromatic Compounds | Peripheral pathways for catabolism of aromatic compounds | [Quinate degradation](https://rast.nmpdr.org/seedviewer.cgi?page=Subsystems&subsystem=Quinate_degradation&organism=549.408) | [3-dehydroquinate dehydratase II (EC 4.2.1.10)](https://rast.nmpdr.org/seedviewer.cgi?page=FunctionalRole&role=3-dehydroquinate%20dehydratase%20II%20(EC%204.2.1.10)&subsystem_name=Quinate_degradation) |
| Metabolism of Aromatic Compounds | Peripheral pathways for catabolism of aromatic compounds | [Benzoate degradation](https://rast.nmpdr.org/seedviewer.cgi?page=Subsystems&subsystem=Benzoate_degradation&organism=549.408) | [Benzoate transport protein](https://rast.nmpdr.org/seedviewer.cgi?page=FunctionalRole&role=Benzoate%20transport%20protein&subsystem_name=Benzoate_degradation) |
| Metabolism of Aromatic Compounds | Peripheral pathways for catabolism of aromatic compounds | [p-Hydroxybenzoate degradation](https://rast.nmpdr.org/seedviewer.cgi?page=Subsystems&subsystem=p-Hydroxybenzoate_degradation&organism=549.408) | [4-hydroxybenzoate transporter](https://rast.nmpdr.org/seedviewer.cgi?page=FunctionalRole&role=4-hydroxybenzoate%20transporter&subsystem_name=p-Hydroxybenzoate_degradation) |
| Metabolism of Aromatic Compounds | Metabolism of central aromatic intermediates | [Salicylate and gentisate catabolism](https://rast.nmpdr.org/seedviewer.cgi?page=Subsystems&subsystem=Salicylate_and_gentisate_catabolism&organism=549.408) | [4-hydroxybenzoate transporter](https://rast.nmpdr.org/seedviewer.cgi?page=FunctionalRole&role=4-hydroxybenzoate%20transporter&subsystem_name=Salicylate_and_gentisate_catabolism) |
| Metabolism of Aromatic Compounds | Metabolism of central aromatic intermediates | [Salicylate and gentisate catabolism](https://rast.nmpdr.org/seedviewer.cgi?page=Subsystems&subsystem=Salicylate_and_gentisate_catabolism&organism=549.408) | [Salicylate hydroxylase (EC 1.14.13.1)](https://rast.nmpdr.org/seedviewer.cgi?page=FunctionalRole&role=Salicylate%20hydroxylase%20(EC%201.14.13.1)&subsystem_name=Salicylate_and_gentisate_catabolism) |
| Amino Acids and Derivatives | Glutamine, glutamate, aspartate, asparagine; ammonia assimilation | [Glutamine, Glutamate, Aspartate and Asparagine Biosynthesis](https://rast.nmpdr.org/seedviewer.cgi?page=Subsystems&subsystem=Glutamine,_Glutamate,_Aspartate_and_Asparagine_Biosynthesis&organism=549.408) | [L-asparaginase (EC 3.5.1.1)](https://rast.nmpdr.org/seedviewer.cgi?page=FunctionalRole&role=L-asparaginase%20(EC%203.5.1.1)&subsystem_name=Glutamine,_Glutamate,_Aspartate_and_Asparagine_Biosynthesis) |
| Amino Acids and Derivatives | Glutamine, glutamate, aspartate, asparagine; ammonia assimilation | [Glutamine, Glutamate, Aspartate and Asparagine Biosynthesis](https://rast.nmpdr.org/seedviewer.cgi?page=Subsystems&subsystem=Glutamine,_Glutamate,_Aspartate_and_Asparagine_Biosynthesis&organism=549.408) | [Aspartate aminotransferase (EC 2.6.1.1)](https://rast.nmpdr.org/seedviewer.cgi?page=FunctionalRole&role=Aspartate%20aminotransferase%20(EC%202.6.1.1)&subsystem_name=Glutamine,_Glutamate,_Aspartate_and_Asparagine_Biosynthesis) |
| Amino Acids and Derivatives | Glutamine, glutamate, aspartate, asparagine; ammonia assimilation | [Glutamine, Glutamate, Aspartate and Asparagine Biosynthesis](https://rast.nmpdr.org/seedviewer.cgi?page=Subsystems&subsystem=Glutamine,_Glutamate,_Aspartate_and_Asparagine_Biosynthesis&organism=549.408) | [NAD-specific glutamate dehydrogenase (EC 1.4.1.2)](https://rast.nmpdr.org/seedviewer.cgi?page=FunctionalRole&role=NAD-specific%20glutamate%20dehydrogenase%20(EC%201.4.1.2)&subsystem_name=Glutamine,_Glutamate,_Aspartate_and_Asparagine_Biosynthesis) |
| Amino Acids and Derivatives | Glutamine, glutamate, aspartate, asparagine; ammonia assimilation | [Glutamine, Glutamate, Aspartate and Asparagine Biosynthesis](https://rast.nmpdr.org/seedviewer.cgi?page=Subsystems&subsystem=Glutamine,_Glutamate,_Aspartate_and_Asparagine_Biosynthesis&organism=549.408) | [Glutamate racemase (EC 5.1.1.3)](https://rast.nmpdr.org/seedviewer.cgi?page=FunctionalRole&role=Glutamate%20racemase%20(EC%205.1.1.3)&subsystem_name=Glutamine,_Glutamate,_Aspartate_and_Asparagine_Biosynthesis) |
| Amino Acids and Derivatives | Glutamine, glutamate, aspartate, asparagine; ammonia assimilation | [Glutamine, Glutamate, Aspartate and Asparagine Biosynthesis](https://rast.nmpdr.org/seedviewer.cgi?page=Subsystems&subsystem=Glutamine,_Glutamate,_Aspartate_and_Asparagine_Biosynthesis&organism=549.408) | [Asparagine synthetase [glutamine-hydrolyzing] (EC 6.3.5.4)](https://rast.nmpdr.org/seedviewer.cgi?page=FunctionalRole&role=Asparagine%20synthetase%20%5bglutamine-hydrolyzing%5d%20(EC%206.3.5.4)&subsystem_name=Glutamine,_Glutamate,_Aspartate_and_Asparagine_Biosynthesis) |
| Amino Acids and Derivatives | Glutamine, glutamate, aspartate, asparagine; ammonia assimilation | [Glutamine, Glutamate, Aspartate and Asparagine Biosynthesis](https://rast.nmpdr.org/seedviewer.cgi?page=Subsystems&subsystem=Glutamine,_Glutamate,_Aspartate_and_Asparagine_Biosynthesis&organism=549.408) | [Glutaminase (EC 3.5.1.2)](https://rast.nmpdr.org/seedviewer.cgi?page=FunctionalRole&role=Glutaminase%20(EC%203.5.1.2)&subsystem_name=Glutamine,_Glutamate,_Aspartate_and_Asparagine_Biosynthesis) |
| Amino Acids and Derivatives | Glutamine, glutamate, aspartate, asparagine; ammonia assimilation | [Glutamine, Glutamate, Aspartate and Asparagine Biosynthesis](https://rast.nmpdr.org/seedviewer.cgi?page=Subsystems&subsystem=Glutamine,_Glutamate,_Aspartate_and_Asparagine_Biosynthesis&organism=549.408) | [Glutamate synthase [NADPH] large chain (EC 1.4.1.13)](https://rast.nmpdr.org/seedviewer.cgi?page=FunctionalRole&role=Glutamate%20synthase%20%5bNADPH%5d%20large%20chain%20(EC%201.4.1.13)&subsystem_name=Glutamine,_Glutamate,_Aspartate_and_Asparagine_Biosynthesis) |
| Amino Acids and Derivatives | Glutamine, glutamate, aspartate, asparagine; ammonia assimilation | [Glutamine, Glutamate, Aspartate and Asparagine Biosynthesis](https://rast.nmpdr.org/seedviewer.cgi?page=Subsystems&subsystem=Glutamine,_Glutamate,_Aspartate_and_Asparagine_Biosynthesis&organism=549.408) | [Glutamine synthetase type I (EC 6.3.1.2)](https://rast.nmpdr.org/seedviewer.cgi?page=FunctionalRole&role=Glutamine%20synthetase%20type%20I%20(EC%206.3.1.2)&subsystem_name=Glutamine,_Glutamate,_Aspartate_and_Asparagine_Biosynthesis) |
| Amino Acids and Derivatives | Glutamine, glutamate, aspartate, asparagine; ammonia assimilation | [Glutamine, Glutamate, Aspartate and Asparagine Biosynthesis](https://rast.nmpdr.org/seedviewer.cgi?page=Subsystems&subsystem=Glutamine,_Glutamate,_Aspartate_and_Asparagine_Biosynthesis&organism=549.408) | [Glutamate synthase [NADPH] small chain (EC 1.4.1.13)](https://rast.nmpdr.org/seedviewer.cgi?page=FunctionalRole&role=Glutamate%20synthase%20%5bNADPH%5d%20small%20chain%20(EC%201.4.1.13)&subsystem_name=Glutamine,_Glutamate,_Aspartate_and_Asparagine_Biosynthesis) |
| Amino Acids and Derivatives | Glutamine, glutamate, aspartate, asparagine; ammonia assimilation | [Glutamine, Glutamate, Aspartate and Asparagine Biosynthesis](https://rast.nmpdr.org/seedviewer.cgi?page=Subsystems&subsystem=Glutamine,_Glutamate,_Aspartate_and_Asparagine_Biosynthesis&organism=549.408) | [NADP-specific glutamate dehydrogenase (EC 1.4.1.4)](https://rast.nmpdr.org/seedviewer.cgi?page=FunctionalRole&role=NADP-specific%20glutamate%20dehydrogenase%20(EC%201.4.1.4)&subsystem_name=Glutamine,_Glutamate,_Aspartate_and_Asparagine_Biosynthesis) |
| Amino Acids and Derivatives | Glutamine, glutamate, aspartate, asparagine; ammonia assimilation | [Glutamine, Glutamate, Aspartate and Asparagine Biosynthesis](https://rast.nmpdr.org/seedviewer.cgi?page=Subsystems&subsystem=Glutamine,_Glutamate,_Aspartate_and_Asparagine_Biosynthesis&organism=549.408) | [Leucine-responsive regulatory protein, regulator for leucine (or lrp) regulon and high-affinity branched-chain amino acid transport system](https://rast.nmpdr.org/seedviewer.cgi?page=FunctionalRole&role=Leucine-responsive%20regulatory%20protein,%20regulator%20for%20leucine%20(or%20lrp)%20regulon%20and%20high-affinity%20branched-chain%20amino%20acid%20transport%20system&subsystem_name=Glutamine,_Glutamate,_Aspartate_and_Asparagine_Biosynthesis) |
| Amino Acids and Derivatives | Glutamine, glutamate, aspartate, asparagine; ammonia assimilation | [Glutamine, Glutamate, Aspartate and Asparagine Biosynthesis](https://rast.nmpdr.org/seedviewer.cgi?page=Subsystems&subsystem=Glutamine,_Glutamate,_Aspartate_and_Asparagine_Biosynthesis&organism=549.408) | [Aspartate ammonia-lyase (EC 4.3.1.1)](https://rast.nmpdr.org/seedviewer.cgi?page=FunctionalRole&role=Aspartate%20ammonia-lyase%20(EC%204.3.1.1)&subsystem_name=Glutamine,_Glutamate,_Aspartate_and_Asparagine_Biosynthesis) |
| Amino Acids and Derivatives | Glutamine, glutamate, aspartate, asparagine; ammonia assimilation | [Glutamine, Glutamate, Aspartate and Asparagine Biosynthesis](https://rast.nmpdr.org/seedviewer.cgi?page=Subsystems&subsystem=Glutamine,_Glutamate,_Aspartate_and_Asparagine_Biosynthesis&organism=549.408) | [Aspartate racemase (EC 5.1.1.13)](https://rast.nmpdr.org/seedviewer.cgi?page=FunctionalRole&role=Aspartate%20racemase%20(EC%205.1.1.13)&subsystem_name=Glutamine,_Glutamate,_Aspartate_and_Asparagine_Biosynthesis) |
| Amino Acids and Derivatives | Glutamine, glutamate, aspartate, asparagine; ammonia assimilation | [Glutamate dehydrogenases](https://rast.nmpdr.org/seedviewer.cgi?page=Subsystems&subsystem=Glutamate_dehydrogenases&organism=549.408) | [NADP-specific glutamate dehydrogenase (EC 1.4.1.4)](https://rast.nmpdr.org/seedviewer.cgi?page=FunctionalRole&role=NADP-specific%20glutamate%20dehydrogenase%20(EC%201.4.1.4)&subsystem_name=Glutamate_dehydrogenases) |
| Amino Acids and Derivatives | Glutamine, glutamate, aspartate, asparagine; ammonia assimilation | [Glutamate dehydrogenases](https://rast.nmpdr.org/seedviewer.cgi?page=Subsystems&subsystem=Glutamate_dehydrogenases&organism=549.408) | [NAD-specific glutamate dehydrogenase (EC 1.4.1.2)](https://rast.nmpdr.org/seedviewer.cgi?page=FunctionalRole&role=NAD-specific%20glutamate%20dehydrogenase%20(EC%201.4.1.2)&subsystem_name=Glutamate_dehydrogenases) |
| Amino Acids and Derivatives | Glutamine, glutamate, aspartate, asparagine; ammonia assimilation | [Glutamine synthetases](https://rast.nmpdr.org/seedviewer.cgi?page=Subsystems&subsystem=Glutamine_synthetases&organism=549.408) | [Glutamine synthetase type I (EC 6.3.1.2)](https://rast.nmpdr.org/seedviewer.cgi?page=FunctionalRole&role=Glutamine%20synthetase%20type%20I%20(EC%206.3.1.2)&subsystem_name=Glutamine_synthetases) |
| Amino Acids and Derivatives | Histidine Metabolism | [Histidine Degradation](https://rast.nmpdr.org/seedviewer.cgi?page=Subsystems&subsystem=Histidine_Degradation&organism=549.408) | [Conserved hypothetical protein (perhaps related to histidine degradation)](https://rast.nmpdr.org/seedviewer.cgi?page=FunctionalRole&role=Conserved%20hypothetical%20protein%20(perhaps%20related%20to%20histidine%20degradation)&subsystem_name=Histidine_Degradation) |
| Amino Acids and Derivatives | Histidine Metabolism | [Histidine Degradation](https://rast.nmpdr.org/seedviewer.cgi?page=Subsystems&subsystem=Histidine_Degradation&organism=549.408) | [N-formylglutamate deformylase (EC 3.5.1.68)](https://rast.nmpdr.org/seedviewer.cgi?page=FunctionalRole&role=N-formylglutamate%20deformylase%20(EC%203.5.1.68)&subsystem_name=Histidine_Degradation) |
| Amino Acids and Derivatives | Histidine Metabolism | [Histidine Degradation](https://rast.nmpdr.org/seedviewer.cgi?page=Subsystems&subsystem=Histidine_Degradation&organism=549.408) | [Imidazolonepropionase (EC 3.5.2.7)](https://rast.nmpdr.org/seedviewer.cgi?page=FunctionalRole&role=Imidazolonepropionase%20(EC%203.5.2.7)&subsystem_name=Histidine_Degradation) |
| Amino Acids and Derivatives | Histidine Metabolism | [Histidine Degradation](https://rast.nmpdr.org/seedviewer.cgi?page=Subsystems&subsystem=Histidine_Degradation&organism=549.408) | [Urocanate hydratase (EC 4.2.1.49)](https://rast.nmpdr.org/seedviewer.cgi?page=FunctionalRole&role=Urocanate%20hydratase%20(EC%204.2.1.49)&subsystem_name=Histidine_Degradation) |
| Amino Acids and Derivatives | Histidine Metabolism | [Histidine Degradation](https://rast.nmpdr.org/seedviewer.cgi?page=Subsystems&subsystem=Histidine_Degradation&organism=549.408) | [Histidine utilization repressor](https://rast.nmpdr.org/seedviewer.cgi?page=FunctionalRole&role=Histidine%20utilization%20repressor&subsystem_name=Histidine_Degradation) |
| Amino Acids and Derivatives | Histidine Metabolism | [Histidine Degradation](https://rast.nmpdr.org/seedviewer.cgi?page=Subsystems&subsystem=Histidine_Degradation&organism=549.408) | [Histidine ammonia-lyase (EC 4.3.1.3)](https://rast.nmpdr.org/seedviewer.cgi?page=FunctionalRole&role=Histidine%20ammonia-lyase%20(EC%204.3.1.3)&subsystem_name=Histidine_Degradation) |
| Amino Acids and Derivatives | Histidine Metabolism | [Histidine Degradation](https://rast.nmpdr.org/seedviewer.cgi?page=Subsystems&subsystem=Histidine_Degradation&organism=549.408) | [Formiminoglutamic iminohydrolase (EC 3.5.3.13)](https://rast.nmpdr.org/seedviewer.cgi?page=FunctionalRole&role=Formiminoglutamic%20iminohydrolase%20(EC%203.5.3.13)&subsystem_name=Histidine_Degradation) |
| Amino Acids and Derivatives | Histidine Metabolism | [Histidine Biosynthesis](https://rast.nmpdr.org/seedviewer.cgi?page=Subsystems&subsystem=Histidine_Biosynthesis&organism=549.408) | [Phosphoribosylformimino-5-aminoimidazole carboxamide ribotide isomerase (EC 5.3.1.16)](https://rast.nmpdr.org/seedviewer.cgi?page=FunctionalRole&role=Phosphoribosylformimino-5-aminoimidazole%20carboxamide%20ribotide%20isomerase%20(EC%205.3.1.16)&subsystem_name=Histidine_Biosynthesis) |
| Amino Acids and Derivatives | Histidine Metabolism | [Histidine Biosynthesis](https://rast.nmpdr.org/seedviewer.cgi?page=Subsystems&subsystem=Histidine_Biosynthesis&organism=549.408) | [Phosphoribosyl-ATP pyrophosphatase (EC 3.6.1.31)](https://rast.nmpdr.org/seedviewer.cgi?page=FunctionalRole&role=Phosphoribosyl-ATP%20pyrophosphatase%20(EC%203.6.1.31)&subsystem_name=Histidine_Biosynthesis) |
| Amino Acids and Derivatives | Histidine Metabolism | [Histidine Biosynthesis](https://rast.nmpdr.org/seedviewer.cgi?page=Subsystems&subsystem=Histidine_Biosynthesis&organism=549.408) | [Imidazole glycerol phosphate synthase amidotransferase subunit (EC 2.4.2.-)](https://rast.nmpdr.org/seedviewer.cgi?page=FunctionalRole&role=Imidazole%20glycerol%20phosphate%20synthase%20amidotransferase%20subunit%20(EC%202.4.2.-)&subsystem_name=Histidine_Biosynthesis) |
| Amino Acids and Derivatives | Histidine Metabolism | [Histidine Biosynthesis](https://rast.nmpdr.org/seedviewer.cgi?page=Subsystems&subsystem=Histidine_Biosynthesis&organism=549.408) | [Histidinol-phosphate aminotransferase (EC 2.6.1.9)](https://rast.nmpdr.org/seedviewer.cgi?page=FunctionalRole&role=Histidinol-phosphate%20aminotransferase%20(EC%202.6.1.9)&subsystem_name=Histidine_Biosynthesis) |
| Amino Acids and Derivatives | Histidine Metabolism | [Histidine Biosynthesis](https://rast.nmpdr.org/seedviewer.cgi?page=Subsystems&subsystem=Histidine_Biosynthesis&organism=549.408) | [Imidazoleglycerol-phosphate dehydratase (EC 4.2.1.19)](https://rast.nmpdr.org/seedviewer.cgi?page=FunctionalRole&role=Imidazoleglycerol-phosphate%20dehydratase%20(EC%204.2.1.19)&subsystem_name=Histidine_Biosynthesis) |
| Amino Acids and Derivatives | Histidine Metabolism | [Histidine Biosynthesis](https://rast.nmpdr.org/seedviewer.cgi?page=Subsystems&subsystem=Histidine_Biosynthesis&organism=549.408) | [Imidazole glycerol phosphate synthase cyclase subunit (EC 4.1.3.-)](https://rast.nmpdr.org/seedviewer.cgi?page=FunctionalRole&role=Imidazole%20glycerol%20phosphate%20synthase%20cyclase%20subunit%20(EC%204.1.3.-)&subsystem_name=Histidine_Biosynthesis) |
| Amino Acids and Derivatives | Histidine Metabolism | [Histidine Biosynthesis](https://rast.nmpdr.org/seedviewer.cgi?page=Subsystems&subsystem=Histidine_Biosynthesis&organism=549.408) | [Phosphoribosyl-AMP cyclohydrolase (EC 3.5.4.19)](https://rast.nmpdr.org/seedviewer.cgi?page=FunctionalRole&role=Phosphoribosyl-AMP%20cyclohydrolase%20(EC%203.5.4.19)&subsystem_name=Histidine_Biosynthesis) |
| Amino Acids and Derivatives | Histidine Metabolism | [Histidine Biosynthesis](https://rast.nmpdr.org/seedviewer.cgi?page=Subsystems&subsystem=Histidine_Biosynthesis&organism=549.408) | [ATP phosphoribosyltransferase (EC 2.4.2.17)](https://rast.nmpdr.org/seedviewer.cgi?page=FunctionalRole&role=ATP%20phosphoribosyltransferase%20(EC%202.4.2.17)&subsystem_name=Histidine_Biosynthesis) |
| Amino Acids and Derivatives | Histidine Metabolism | [Histidine Biosynthesis](https://rast.nmpdr.org/seedviewer.cgi?page=Subsystems&subsystem=Histidine_Biosynthesis&organism=549.408) | [Histidinol dehydrogenase (EC 1.1.1.23)](https://rast.nmpdr.org/seedviewer.cgi?page=FunctionalRole&role=Histidinol%20dehydrogenase%20(EC%201.1.1.23)&subsystem_name=Histidine_Biosynthesis) |
| Amino Acids and Derivatives | Histidine Metabolism | [Histidine Biosynthesis](https://rast.nmpdr.org/seedviewer.cgi?page=Subsystems&subsystem=Histidine_Biosynthesis&organism=549.408) | [Histidinol-phosphatase (EC 3.1.3.15)](https://rast.nmpdr.org/seedviewer.cgi?page=FunctionalRole&role=Histidinol-phosphatase%20(EC%203.1.3.15)&subsystem_name=Histidine_Biosynthesis) |
| Amino Acids and Derivatives | Arginine; urea cycle, polyamines | [Putrescine utilization pathways](https://rast.nmpdr.org/seedviewer.cgi?page=Subsystems&subsystem=Putrescine_utilization_pathways&organism=549.408) | [Putrescine importer](https://rast.nmpdr.org/seedviewer.cgi?page=FunctionalRole&role=Putrescine%20importer&subsystem_name=Putrescine_utilization_pathways) |
| Amino Acids and Derivatives | Arginine; urea cycle, polyamines | [Putrescine utilization pathways](https://rast.nmpdr.org/seedviewer.cgi?page=Subsystems&subsystem=Putrescine_utilization_pathways&organism=549.408) | [Gamma-glutamyl-putrescine synthetase (EC 6.3.1.11)](https://rast.nmpdr.org/seedviewer.cgi?page=FunctionalRole&role=Gamma-glutamyl-putrescine%20synthetase%20(EC%206.3.1.11)&subsystem_name=Putrescine_utilization_pathways) |
| Amino Acids and Derivatives | Arginine; urea cycle, polyamines | [Putrescine utilization pathways](https://rast.nmpdr.org/seedviewer.cgi?page=Subsystems&subsystem=Putrescine_utilization_pathways&organism=549.408) | [Gamma-glutamyl-putrescine oxidase (EC1.4.3.-)](https://rast.nmpdr.org/seedviewer.cgi?page=FunctionalRole&role=Gamma-glutamyl-putrescine%20oxidase%20(EC1.4.3.-)&subsystem_name=Putrescine_utilization_pathways) |
| Amino Acids and Derivatives | Arginine; urea cycle, polyamines | [Putrescine utilization pathways](https://rast.nmpdr.org/seedviewer.cgi?page=Subsystems&subsystem=Putrescine_utilization_pathways&organism=549.408) | [Gamma-aminobutyrate:alpha-ketoglutarate aminotransferase (EC 2.6.1.19)](https://rast.nmpdr.org/seedviewer.cgi?page=FunctionalRole&role=Gamma-aminobutyrate:alpha-ketoglutarate%20aminotransferase%20(EC%202.6.1.19)&subsystem_name=Putrescine_utilization_pathways) |
| Amino Acids and Derivatives | Arginine; urea cycle, polyamines | [Putrescine utilization pathways](https://rast.nmpdr.org/seedviewer.cgi?page=Subsystems&subsystem=Putrescine_utilization_pathways&organism=549.408) | [Putrescine utilization regulator](https://rast.nmpdr.org/seedviewer.cgi?page=FunctionalRole&role=Putrescine%20utilization%20regulator&subsystem_name=Putrescine_utilization_pathways) |
| Amino Acids and Derivatives | Arginine; urea cycle, polyamines | [Putrescine utilization pathways](https://rast.nmpdr.org/seedviewer.cgi?page=Subsystems&subsystem=Putrescine_utilization_pathways&organism=549.408) | [Gamma-glutamyl-GABA hydrolase (EC 3.5.1.94)](https://rast.nmpdr.org/seedviewer.cgi?page=FunctionalRole&role=Gamma-glutamyl-GABA%20hydrolase%20(EC%203.5.1.94)&subsystem_name=Putrescine_utilization_pathways) |
| Amino Acids and Derivatives | Arginine; urea cycle, polyamines | [Arginine Deiminase Pathway](https://rast.nmpdr.org/seedviewer.cgi?page=Subsystems&subsystem=Arginine_Deiminase_Pathway&organism=549.408) | [Arginine/ornithine antiporter ArcD](https://rast.nmpdr.org/seedviewer.cgi?page=FunctionalRole&role=Arginine/ornithine%20antiporter%20ArcD&subsystem_name=Arginine_Deiminase_Pathway) |
| Amino Acids and Derivatives | Arginine; urea cycle, polyamines | [Arginine Deiminase Pathway](https://rast.nmpdr.org/seedviewer.cgi?page=Subsystems&subsystem=Arginine_Deiminase_Pathway&organism=549.408) | [Arginine pathway regulatory protein ArgR, repressor of arg regulon](https://rast.nmpdr.org/seedviewer.cgi?page=FunctionalRole&role=Arginine%20pathway%20regulatory%20protein%20ArgR,%20repressor%20of%20arg%20regulon&subsystem_name=Arginine_Deiminase_Pathway) |
| Amino Acids and Derivatives | Arginine; urea cycle, polyamines | [Arginine Deiminase Pathway](https://rast.nmpdr.org/seedviewer.cgi?page=Subsystems&subsystem=Arginine_Deiminase_Pathway&organism=549.408) | [Ornithine carbamoyltransferase (EC 2.1.3.3)](https://rast.nmpdr.org/seedviewer.cgi?page=FunctionalRole&role=Ornithine%20carbamoyltransferase%20(EC%202.1.3.3)&subsystem_name=Arginine_Deiminase_Pathway) |
| Amino Acids and Derivatives | Arginine; urea cycle, polyamines | [Polyamine Metabolism](https://rast.nmpdr.org/seedviewer.cgi?page=Subsystems&subsystem=Polyamine_Metabolism&organism=549.408) | [Arginine/ornithine antiporter ArcD](https://rast.nmpdr.org/seedviewer.cgi?page=FunctionalRole&role=Arginine/ornithine%20antiporter%20ArcD&subsystem_name=Polyamine_Metabolism) |
| Amino Acids and Derivatives | Arginine; urea cycle, polyamines | [Polyamine Metabolism](https://rast.nmpdr.org/seedviewer.cgi?page=Subsystems&subsystem=Polyamine_Metabolism&organism=549.408) | [Spermidine synthase (EC 2.5.1.16)](https://rast.nmpdr.org/seedviewer.cgi?page=FunctionalRole&role=Spermidine%20synthase%20(EC%202.5.1.16)&subsystem_name=Polyamine_Metabolism) |
| Amino Acids and Derivatives | Arginine; urea cycle, polyamines | [Polyamine Metabolism](https://rast.nmpdr.org/seedviewer.cgi?page=Subsystems&subsystem=Polyamine_Metabolism&organism=549.408) | [Putrescine transport ATP-binding protein PotG (TC 3.A.1.11.2)](https://rast.nmpdr.org/seedviewer.cgi?page=FunctionalRole&role=Putrescine%20transport%20ATP-binding%20protein%20PotG%20(TC%203.A.1.11.2)&subsystem_name=Polyamine_Metabolism) |
| Amino Acids and Derivatives | Arginine; urea cycle, polyamines | [Polyamine Metabolism](https://rast.nmpdr.org/seedviewer.cgi?page=Subsystems&subsystem=Polyamine_Metabolism&organism=549.408) | [S-adenosylmethionine decarboxylase proenzyme (EC 4.1.1.50), prokaryotic class 1A](https://rast.nmpdr.org/seedviewer.cgi?page=FunctionalRole&role=S-adenosylmethionine%20decarboxylase%20proenzyme%20(EC%204.1.1.50),%20prokaryotic%20class%201A&subsystem_name=Polyamine_Metabolism) |
| Amino Acids and Derivatives | Arginine; urea cycle, polyamines | [Polyamine Metabolism](https://rast.nmpdr.org/seedviewer.cgi?page=Subsystems&subsystem=Polyamine_Metabolism&organism=549.408) | [Biosynthetic arginine decarboxylase (EC 4.1.1.19)](https://rast.nmpdr.org/seedviewer.cgi?page=FunctionalRole&role=Biosynthetic%20arginine%20decarboxylase%20(EC%204.1.1.19)&subsystem_name=Polyamine_Metabolism) |
| Amino Acids and Derivatives | Arginine; urea cycle, polyamines | [Polyamine Metabolism](https://rast.nmpdr.org/seedviewer.cgi?page=Subsystems&subsystem=Polyamine_Metabolism&organism=549.408) | [Spermidine N1-acetyltransferase (EC 2.3.1.57)](https://rast.nmpdr.org/seedviewer.cgi?page=FunctionalRole&role=Spermidine%20N1-acetyltransferase%20(EC%202.3.1.57)&subsystem_name=Polyamine_Metabolism) |
| Amino Acids and Derivatives | Arginine; urea cycle, polyamines | [Polyamine Metabolism](https://rast.nmpdr.org/seedviewer.cgi?page=Subsystems&subsystem=Polyamine_Metabolism&organism=549.408) | [Agmatinase (EC 3.5.3.11)](https://rast.nmpdr.org/seedviewer.cgi?page=FunctionalRole&role=Agmatinase%20(EC%203.5.3.11)&subsystem_name=Polyamine_Metabolism) |
| Amino Acids and Derivatives | Arginine; urea cycle, polyamines | [Polyamine Metabolism](https://rast.nmpdr.org/seedviewer.cgi?page=Subsystems&subsystem=Polyamine_Metabolism&organism=549.408) | [Putrescine transport system permease protein PotH (TC 3.A.1.11.2)](https://rast.nmpdr.org/seedviewer.cgi?page=FunctionalRole&role=Putrescine%20transport%20system%20permease%20protein%20PotH%20(TC%203.A.1.11.2)&subsystem_name=Polyamine_Metabolism) |
| Amino Acids and Derivatives | Arginine; urea cycle, polyamines | [Polyamine Metabolism](https://rast.nmpdr.org/seedviewer.cgi?page=Subsystems&subsystem=Polyamine_Metabolism&organism=549.408) | [Putrescine transport ATP-binding protein PotA (TC 3.A.1.11.1)](https://rast.nmpdr.org/seedviewer.cgi?page=FunctionalRole&role=Putrescine%20transport%20ATP-binding%20protein%20PotA%20(TC%203.A.1.11.1)&subsystem_name=Polyamine_Metabolism) |
| Amino Acids and Derivatives | Arginine; urea cycle, polyamines | [Polyamine Metabolism](https://rast.nmpdr.org/seedviewer.cgi?page=Subsystems&subsystem=Polyamine_Metabolism&organism=549.408) | [Putrescine transport system permease protein PotI (TC 3.A.1.11.2)](https://rast.nmpdr.org/seedviewer.cgi?page=FunctionalRole&role=Putrescine%20transport%20system%20permease%20protein%20PotI%20(TC%203.A.1.11.2)&subsystem_name=Polyamine_Metabolism) |
| Amino Acids and Derivatives | Arginine; urea cycle, polyamines | [Polyamine Metabolism](https://rast.nmpdr.org/seedviewer.cgi?page=Subsystems&subsystem=Polyamine_Metabolism&organism=549.408) | [Predicted chaperone lipoprotein YacC, potentially involved in protein secretion](https://rast.nmpdr.org/seedviewer.cgi?page=FunctionalRole&role=Predicted%20chaperone%20lipoprotein%20YacC,%20potentially%20involved%20in%20protein%20secretion&subsystem_name=Polyamine_Metabolism) |
| Amino Acids and Derivatives | Arginine; urea cycle, polyamines | [Polyamine Metabolism](https://rast.nmpdr.org/seedviewer.cgi?page=Subsystems&subsystem=Polyamine_Metabolism&organism=549.408) | [DNA-binding response regulator, LuxR family, near polyamine transporter](https://rast.nmpdr.org/seedviewer.cgi?page=FunctionalRole&role=DNA-binding%20response%20regulator,%20LuxR%20family,%20near%20polyamine%20transporter&subsystem_name=Polyamine_Metabolism) |
| Amino Acids and Derivatives | Arginine; urea cycle, polyamines | [Polyamine Metabolism](https://rast.nmpdr.org/seedviewer.cgi?page=Subsystems&subsystem=Polyamine_Metabolism&organism=549.408) | [Ornithine decarboxylase (EC 4.1.1.17)](https://rast.nmpdr.org/seedviewer.cgi?page=FunctionalRole&role=Ornithine%20decarboxylase%20(EC%204.1.1.17)&subsystem_name=Polyamine_Metabolism) |
| Amino Acids and Derivatives | Arginine; urea cycle, polyamines | [Polyamine Metabolism](https://rast.nmpdr.org/seedviewer.cgi?page=Subsystems&subsystem=Polyamine_Metabolism&organism=549.408) | [Putrescine ABC transporter putrescine-binding protein PotF (TC 3.A.1.11.2)](https://rast.nmpdr.org/seedviewer.cgi?page=FunctionalRole&role=Putrescine%20ABC%20transporter%20putrescine-binding%20protein%20PotF%20(TC%203.A.1.11.2)&subsystem_name=Polyamine_Metabolism) |
| Amino Acids and Derivatives | Arginine; urea cycle, polyamines | [Polyamine Metabolism](https://rast.nmpdr.org/seedviewer.cgi?page=Subsystems&subsystem=Polyamine_Metabolism&organism=549.408) | [5'-methylthioadenosine nucleosidase (EC 3.2.2.16)](https://rast.nmpdr.org/seedviewer.cgi?page=FunctionalRole&role=5) |
| Amino Acids and Derivatives | Arginine; urea cycle, polyamines | [Arginine and Ornithine Degradation](https://rast.nmpdr.org/seedviewer.cgi?page=Subsystems&subsystem=Arginine_and_Ornithine_Degradation&organism=549.408) | [Arginine/ornithine antiporter ArcD](https://rast.nmpdr.org/seedviewer.cgi?page=FunctionalRole&role=Arginine/ornithine%20antiporter%20ArcD&subsystem_name=Arginine_and_Ornithine_Degradation) |
| Amino Acids and Derivatives | Arginine; urea cycle, polyamines | [Arginine and Ornithine Degradation](https://rast.nmpdr.org/seedviewer.cgi?page=Subsystems&subsystem=Arginine_and_Ornithine_Degradation&organism=549.408) | [Arginine ABC transporter, permease protein ArtQ](https://rast.nmpdr.org/seedviewer.cgi?page=FunctionalRole&role=Arginine%20ABC%20transporter,%20permease%20protein%20ArtQ&subsystem_name=Arginine_and_Ornithine_Degradation) |
| Amino Acids and Derivatives | Arginine; urea cycle, polyamines | [Arginine and Ornithine Degradation](https://rast.nmpdr.org/seedviewer.cgi?page=Subsystems&subsystem=Arginine_and_Ornithine_Degradation&organism=549.408) | [Histidine ABC transporter, histidine-binding periplasmic protein precursor HisJ (TC 3.A.1.3.1)](https://rast.nmpdr.org/seedviewer.cgi?page=FunctionalRole&role=Histidine%20ABC%20transporter,%20histidine-binding%20periplasmic%20protein%20precursor%20HisJ%20(TC%203.A.1.3.1)&subsystem_name=Arginine_and_Ornithine_Degradation) |
| Amino Acids and Derivatives | Arginine; urea cycle, polyamines | [Arginine and Ornithine Degradation](https://rast.nmpdr.org/seedviewer.cgi?page=Subsystems&subsystem=Arginine_and_Ornithine_Degradation&organism=549.408) | [Succinylornithine transaminase (EC 2.6.1.81)](https://rast.nmpdr.org/seedviewer.cgi?page=FunctionalRole&role=Succinylornithine%20transaminase%20(EC%202.6.1.81)&subsystem_name=Arginine_and_Ornithine_Degradation) |
| Amino Acids and Derivatives | Arginine; urea cycle, polyamines | [Arginine and Ornithine Degradation](https://rast.nmpdr.org/seedviewer.cgi?page=Subsystems&subsystem=Arginine_and_Ornithine_Degradation&organism=549.408) | [Succinylglutamic semialdehyde dehydrogenase (EC 1.2.1.71)](https://rast.nmpdr.org/seedviewer.cgi?page=FunctionalRole&role=Succinylglutamic%20semialdehyde%20dehydrogenase%20(EC%201.2.1.71)&subsystem_name=Arginine_and_Ornithine_Degradation) |
| Amino Acids and Derivatives | Arginine; urea cycle, polyamines | [Arginine and Ornithine Degradation](https://rast.nmpdr.org/seedviewer.cgi?page=Subsystems&subsystem=Arginine_and_Ornithine_Degradation&organism=549.408) | [Ornithine decarboxylase (EC 4.1.1.17)](https://rast.nmpdr.org/seedviewer.cgi?page=FunctionalRole&role=Ornithine%20decarboxylase%20(EC%204.1.1.17)&subsystem_name=Arginine_and_Ornithine_Degradation) |
| Amino Acids and Derivatives | Arginine; urea cycle, polyamines | [Arginine and Ornithine Degradation](https://rast.nmpdr.org/seedviewer.cgi?page=Subsystems&subsystem=Arginine_and_Ornithine_Degradation&organism=549.408) | [Succinylarginine dihydrolase (EC 3.5.3.23)](https://rast.nmpdr.org/seedviewer.cgi?page=FunctionalRole&role=Succinylarginine%20dihydrolase%20(EC%203.5.3.23)&subsystem_name=Arginine_and_Ornithine_Degradation) |
| Amino Acids and Derivatives | Arginine; urea cycle, polyamines | [Arginine and Ornithine Degradation](https://rast.nmpdr.org/seedviewer.cgi?page=Subsystems&subsystem=Arginine_and_Ornithine_Degradation&organism=549.408) | [NADP-specific glutamate dehydrogenase (EC 1.4.1.4)](https://rast.nmpdr.org/seedviewer.cgi?page=FunctionalRole&role=NADP-specific%20glutamate%20dehydrogenase%20(EC%201.4.1.4)&subsystem_name=Arginine_and_Ornithine_Degradation) |
| Amino Acids and Derivatives | Arginine; urea cycle, polyamines | [Arginine and Ornithine Degradation](https://rast.nmpdr.org/seedviewer.cgi?page=Subsystems&subsystem=Arginine_and_Ornithine_Degradation&organism=549.408) | [Histidine ABC transporter, permease protein HisQ (TC 3.A.1.3.1)](https://rast.nmpdr.org/seedviewer.cgi?page=FunctionalRole&role=Histidine%20ABC%20transporter,%20permease%20protein%20HisQ%20(TC%203.A.1.3.1)&subsystem_name=Arginine_and_Ornithine_Degradation) |
| Amino Acids and Derivatives | Arginine; urea cycle, polyamines | [Arginine and Ornithine Degradation](https://rast.nmpdr.org/seedviewer.cgi?page=Subsystems&subsystem=Arginine_and_Ornithine_Degradation&organism=549.408) | [Arginine ABC transporter, periplasmic arginine-binding protein ArtJ](https://rast.nmpdr.org/seedviewer.cgi?page=FunctionalRole&role=Arginine%20ABC%20transporter,%20periplasmic%20arginine-binding%20protein%20ArtJ&subsystem_name=Arginine_and_Ornithine_Degradation) |
| Amino Acids and Derivatives | Arginine; urea cycle, polyamines | [Arginine and Ornithine Degradation](https://rast.nmpdr.org/seedviewer.cgi?page=Subsystems&subsystem=Arginine_and_Ornithine_Degradation&organism=549.408) | [Arginine ABC transporter, permease protein ArtM](https://rast.nmpdr.org/seedviewer.cgi?page=FunctionalRole&role=Arginine%20ABC%20transporter,%20permease%20protein%20ArtM&subsystem_name=Arginine_and_Ornithine_Degradation) |
| Amino Acids and Derivatives | Arginine; urea cycle, polyamines | [Arginine and Ornithine Degradation](https://rast.nmpdr.org/seedviewer.cgi?page=Subsystems&subsystem=Arginine_and_Ornithine_Degradation&organism=549.408) | [Biosynthetic arginine decarboxylase (EC 4.1.1.19)](https://rast.nmpdr.org/seedviewer.cgi?page=FunctionalRole&role=Biosynthetic%20arginine%20decarboxylase%20(EC%204.1.1.19)&subsystem_name=Arginine_and_Ornithine_Degradation) |
| Amino Acids and Derivatives | Arginine; urea cycle, polyamines | [Arginine and Ornithine Degradation](https://rast.nmpdr.org/seedviewer.cgi?page=Subsystems&subsystem=Arginine_and_Ornithine_Degradation&organism=549.408) | [Agmatinase (EC 3.5.3.11)](https://rast.nmpdr.org/seedviewer.cgi?page=FunctionalRole&role=Agmatinase%20(EC%203.5.3.11)&subsystem_name=Arginine_and_Ornithine_Degradation) |
| Amino Acids and Derivatives | Arginine; urea cycle, polyamines | [Arginine and Ornithine Degradation](https://rast.nmpdr.org/seedviewer.cgi?page=Subsystems&subsystem=Arginine_and_Ornithine_Degradation&organism=549.408) | [Arginine ABC transporter, ATP-binding protein ArtP](https://rast.nmpdr.org/seedviewer.cgi?page=FunctionalRole&role=Arginine%20ABC%20transporter,%20ATP-binding%20protein%20ArtP&subsystem_name=Arginine_and_Ornithine_Degradation) |
| Amino Acids and Derivatives | Arginine; urea cycle, polyamines | [Arginine and Ornithine Degradation](https://rast.nmpdr.org/seedviewer.cgi?page=Subsystems&subsystem=Arginine_and_Ornithine_Degradation&organism=549.408) | [Arginine ABC transporter, periplasmic arginine-binding protein ArtI](https://rast.nmpdr.org/seedviewer.cgi?page=FunctionalRole&role=Arginine%20ABC%20transporter,%20periplasmic%20arginine-binding%20protein%20ArtI&subsystem_name=Arginine_and_Ornithine_Degradation) |
| Amino Acids and Derivatives | Arginine; urea cycle, polyamines | [Arginine and Ornithine Degradation](https://rast.nmpdr.org/seedviewer.cgi?page=Subsystems&subsystem=Arginine_and_Ornithine_Degradation&organism=549.408) | [Arginine N-succinyltransferase (EC 2.3.1.109)](https://rast.nmpdr.org/seedviewer.cgi?page=FunctionalRole&role=Arginine%20N-succinyltransferase%20(EC%202.3.1.109)&subsystem_name=Arginine_and_Ornithine_Degradation) |
| Amino Acids and Derivatives | Arginine; urea cycle, polyamines | [Arginine and Ornithine Degradation](https://rast.nmpdr.org/seedviewer.cgi?page=Subsystems&subsystem=Arginine_and_Ornithine_Degradation&organism=549.408) | [Histidine ABC transporter, ATP-binding protein HisP (TC 3.A.1.3.1)](https://rast.nmpdr.org/seedviewer.cgi?page=FunctionalRole&role=Histidine%20ABC%20transporter,%20ATP-binding%20protein%20HisP%20(TC%203.A.1.3.1)&subsystem_name=Arginine_and_Ornithine_Degradation) |
| Amino Acids and Derivatives | Arginine; urea cycle, polyamines | [Arginine and Ornithine Degradation](https://rast.nmpdr.org/seedviewer.cgi?page=Subsystems&subsystem=Arginine_and_Ornithine_Degradation&organism=549.408) | [Histidine ABC transporter, permease protein HisM (TC 3.A.1.3.1)](https://rast.nmpdr.org/seedviewer.cgi?page=FunctionalRole&role=Histidine%20ABC%20transporter,%20permease%20protein%20HisM%20(TC%203.A.1.3.1)&subsystem_name=Arginine_and_Ornithine_Degradation) |
| Amino Acids and Derivatives | Arginine; urea cycle, polyamines | [Arginine and Ornithine Degradation](https://rast.nmpdr.org/seedviewer.cgi?page=Subsystems&subsystem=Arginine_and_Ornithine_Degradation&organism=549.408) | [Arginine pathway regulatory protein ArgR, repressor of arg regulon](https://rast.nmpdr.org/seedviewer.cgi?page=FunctionalRole&role=Arginine%20pathway%20regulatory%20protein%20ArgR,%20repressor%20of%20arg%20regulon&subsystem_name=Arginine_and_Ornithine_Degradation) |
| Amino Acids and Derivatives | Arginine; urea cycle, polyamines | [Arginine and Ornithine Degradation](https://rast.nmpdr.org/seedviewer.cgi?page=Subsystems&subsystem=Arginine_and_Ornithine_Degradation&organism=549.408) | [Succinylglutamate desuccinylase (EC 3.5.1.96)](https://rast.nmpdr.org/seedviewer.cgi?page=FunctionalRole&role=Succinylglutamate%20desuccinylase%20(EC%203.5.1.96)&subsystem_name=Arginine_and_Ornithine_Degradation) |
| Amino Acids and Derivatives | Arginine; urea cycle, polyamines | [Arginine and Ornithine Degradation](https://rast.nmpdr.org/seedviewer.cgi?page=Subsystems&subsystem=Arginine_and_Ornithine_Degradation&organism=549.408) | [Ornithine carbamoyltransferase (EC 2.1.3.3)](https://rast.nmpdr.org/seedviewer.cgi?page=FunctionalRole&role=Ornithine%20carbamoyltransferase%20(EC%202.1.3.3)&subsystem_name=Arginine_and_Ornithine_Degradation) |
| Amino Acids and Derivatives | Arginine; urea cycle, polyamines | [Arginine and Ornithine Degradation](https://rast.nmpdr.org/seedviewer.cgi?page=Subsystems&subsystem=Arginine_and_Ornithine_Degradation&organism=549.408) | [Lysine-arginine-ornithine-binding periplasmic protein precursor (TC 3.A.1.3.1)](https://rast.nmpdr.org/seedviewer.cgi?page=FunctionalRole&role=Lysine-arginine-ornithine-binding%20periplasmic%20protein%20precursor%20(TC%203.A.1.3.1)&subsystem_name=Arginine_and_Ornithine_Degradation) |
| Amino Acids and Derivatives | Arginine; urea cycle, polyamines | [Arginine Biosynthesis extended](https://rast.nmpdr.org/seedviewer.cgi?page=Subsystems&subsystem=Arginine_Biosynthesis_extended&organism=549.408) | [N-succinyl-L,L-diaminopimelate desuccinylase (EC 3.5.1.18)](https://rast.nmpdr.org/seedviewer.cgi?page=FunctionalRole&role=N-succinyl-L,L-diaminopimelate%20desuccinylase%20(EC%203.5.1.18)&subsystem_name=Arginine_Biosynthesis_extended) |
| Amino Acids and Derivatives | Arginine; urea cycle, polyamines | [Arginine Biosynthesis extended](https://rast.nmpdr.org/seedviewer.cgi?page=Subsystems&subsystem=Arginine_Biosynthesis_extended&organism=549.408) | [Acetylornithine deacetylase (EC 3.5.1.16)](https://rast.nmpdr.org/seedviewer.cgi?page=FunctionalRole&role=Acetylornithine%20deacetylase%20(EC%203.5.1.16)&subsystem_name=Arginine_Biosynthesis_extended) |
| Amino Acids and Derivatives | Arginine; urea cycle, polyamines | [Arginine Biosynthesis extended](https://rast.nmpdr.org/seedviewer.cgi?page=Subsystems&subsystem=Arginine_Biosynthesis_extended&organism=549.408) | [Acetylglutamate kinase (EC 2.7.2.8)](https://rast.nmpdr.org/seedviewer.cgi?page=FunctionalRole&role=Acetylglutamate%20kinase%20(EC%202.7.2.8)&subsystem_name=Arginine_Biosynthesis_extended) |
| Amino Acids and Derivatives | Arginine; urea cycle, polyamines | [Arginine Biosynthesis extended](https://rast.nmpdr.org/seedviewer.cgi?page=Subsystems&subsystem=Arginine_Biosynthesis_extended&organism=549.408) | [Arginine pathway regulatory protein ArgR, repressor of arg regulon](https://rast.nmpdr.org/seedviewer.cgi?page=FunctionalRole&role=Arginine%20pathway%20regulatory%20protein%20ArgR,%20repressor%20of%20arg%20regulon&subsystem_name=Arginine_Biosynthesis_extended) |
| Amino Acids and Derivatives | Arginine; urea cycle, polyamines | [Arginine Biosynthesis extended](https://rast.nmpdr.org/seedviewer.cgi?page=Subsystems&subsystem=Arginine_Biosynthesis_extended&organism=549.408) | [Argininosuccinate lyase (EC 4.3.2.1)](https://rast.nmpdr.org/seedviewer.cgi?page=FunctionalRole&role=Argininosuccinate%20lyase%20(EC%204.3.2.1)&subsystem_name=Arginine_Biosynthesis_extended) |
| Amino Acids and Derivatives | Arginine; urea cycle, polyamines | [Arginine Biosynthesis extended](https://rast.nmpdr.org/seedviewer.cgi?page=Subsystems&subsystem=Arginine_Biosynthesis_extended&organism=549.408) | [N-acetylglutamate synthase (EC 2.3.1.1)](https://rast.nmpdr.org/seedviewer.cgi?page=FunctionalRole&role=N-acetylglutamate%20synthase%20(EC%202.3.1.1)&subsystem_name=Arginine_Biosynthesis_extended) |
| Amino Acids and Derivatives | Arginine; urea cycle, polyamines | [Arginine Biosynthesis extended](https://rast.nmpdr.org/seedviewer.cgi?page=Subsystems&subsystem=Arginine_Biosynthesis_extended&organism=549.408) | [Argininosuccinate synthase (EC 6.3.4.5)](https://rast.nmpdr.org/seedviewer.cgi?page=FunctionalRole&role=Argininosuccinate%20synthase%20(EC%206.3.4.5)&subsystem_name=Arginine_Biosynthesis_extended) |
| Amino Acids and Derivatives | Arginine; urea cycle, polyamines | [Arginine Biosynthesis extended](https://rast.nmpdr.org/seedviewer.cgi?page=Subsystems&subsystem=Arginine_Biosynthesis_extended&organism=549.408) | [N-acetyl-gamma-glutamyl-phosphate reductase (EC 1.2.1.38)](https://rast.nmpdr.org/seedviewer.cgi?page=FunctionalRole&role=N-acetyl-gamma-glutamyl-phosphate%20reductase%20(EC%201.2.1.38)&subsystem_name=Arginine_Biosynthesis_extended) |
| Amino Acids and Derivatives | Arginine; urea cycle, polyamines | [Arginine Biosynthesis extended](https://rast.nmpdr.org/seedviewer.cgi?page=Subsystems&subsystem=Arginine_Biosynthesis_extended&organism=549.408) | [Ornithine carbamoyltransferase (EC 2.1.3.3)](https://rast.nmpdr.org/seedviewer.cgi?page=FunctionalRole&role=Ornithine%20carbamoyltransferase%20(EC%202.1.3.3)&subsystem_name=Arginine_Biosynthesis_extended) |
| Amino Acids and Derivatives | Arginine; urea cycle, polyamines | [Arginine Biosynthesis extended](https://rast.nmpdr.org/seedviewer.cgi?page=Subsystems&subsystem=Arginine_Biosynthesis_extended&organism=549.408) | [Acetylornithine aminotransferase (EC 2.6.1.11)](https://rast.nmpdr.org/seedviewer.cgi?page=FunctionalRole&role=Acetylornithine%20aminotransferase%20(EC%202.6.1.11)&subsystem_name=Arginine_Biosynthesis_extended) |
| Amino Acids and Derivatives | Arginine; urea cycle, polyamines | [Arginine Biosynthesis -- gjo](https://rast.nmpdr.org/seedviewer.cgi?page=Subsystems&subsystem=Arginine_Biosynthesis_--_gjo&organism=549.408) | [N-succinyl-L,L-diaminopimelate desuccinylase (EC 3.5.1.18)](https://rast.nmpdr.org/seedviewer.cgi?page=FunctionalRole&role=N-succinyl-L,L-diaminopimelate%20desuccinylase%20(EC%203.5.1.18)&subsystem_name=Arginine_Biosynthesis_--_gjo) |
| Amino Acids and Derivatives | Arginine; urea cycle, polyamines | [Arginine Biosynthesis -- gjo](https://rast.nmpdr.org/seedviewer.cgi?page=Subsystems&subsystem=Arginine_Biosynthesis_--_gjo&organism=549.408) | [Acetylornithine deacetylase (EC 3.5.1.16)](https://rast.nmpdr.org/seedviewer.cgi?page=FunctionalRole&role=Acetylornithine%20deacetylase%20(EC%203.5.1.16)&subsystem_name=Arginine_Biosynthesis_--_gjo) |
| Amino Acids and Derivatives | Arginine; urea cycle, polyamines | [Arginine Biosynthesis -- gjo](https://rast.nmpdr.org/seedviewer.cgi?page=Subsystems&subsystem=Arginine_Biosynthesis_--_gjo&organism=549.408) | [Acetylglutamate kinase (EC 2.7.2.8)](https://rast.nmpdr.org/seedviewer.cgi?page=FunctionalRole&role=Acetylglutamate%20kinase%20(EC%202.7.2.8)&subsystem_name=Arginine_Biosynthesis_--_gjo) |
| Amino Acids and Derivatives | Arginine; urea cycle, polyamines | [Arginine Biosynthesis -- gjo](https://rast.nmpdr.org/seedviewer.cgi?page=Subsystems&subsystem=Arginine_Biosynthesis_--_gjo&organism=549.408) | [Arginine pathway regulatory protein ArgR, repressor of arg regulon](https://rast.nmpdr.org/seedviewer.cgi?page=FunctionalRole&role=Arginine%20pathway%20regulatory%20protein%20ArgR,%20repressor%20of%20arg%20regulon&subsystem_name=Arginine_Biosynthesis_--_gjo) |
| Amino Acids and Derivatives | Arginine; urea cycle, polyamines | [Arginine Biosynthesis -- gjo](https://rast.nmpdr.org/seedviewer.cgi?page=Subsystems&subsystem=Arginine_Biosynthesis_--_gjo&organism=549.408) | [Argininosuccinate lyase (EC 4.3.2.1)](https://rast.nmpdr.org/seedviewer.cgi?page=FunctionalRole&role=Argininosuccinate%20lyase%20(EC%204.3.2.1)&subsystem_name=Arginine_Biosynthesis_--_gjo) |
| Amino Acids and Derivatives | Arginine; urea cycle, polyamines | [Arginine Biosynthesis -- gjo](https://rast.nmpdr.org/seedviewer.cgi?page=Subsystems&subsystem=Arginine_Biosynthesis_--_gjo&organism=549.408) | [N-acetylglutamate synthase (EC 2.3.1.1)](https://rast.nmpdr.org/seedviewer.cgi?page=FunctionalRole&role=N-acetylglutamate%20synthase%20(EC%202.3.1.1)&subsystem_name=Arginine_Biosynthesis_--_gjo) |
| Amino Acids and Derivatives | Arginine; urea cycle, polyamines | [Arginine Biosynthesis -- gjo](https://rast.nmpdr.org/seedviewer.cgi?page=Subsystems&subsystem=Arginine_Biosynthesis_--_gjo&organism=549.408) | [Argininosuccinate synthase (EC 6.3.4.5)](https://rast.nmpdr.org/seedviewer.cgi?page=FunctionalRole&role=Argininosuccinate%20synthase%20(EC%206.3.4.5)&subsystem_name=Arginine_Biosynthesis_--_gjo) |
| Amino Acids and Derivatives | Arginine; urea cycle, polyamines | [Arginine Biosynthesis -- gjo](https://rast.nmpdr.org/seedviewer.cgi?page=Subsystems&subsystem=Arginine_Biosynthesis_--_gjo&organism=549.408) | [N-acetyl-gamma-glutamyl-phosphate reductase (EC 1.2.1.38)](https://rast.nmpdr.org/seedviewer.cgi?page=FunctionalRole&role=N-acetyl-gamma-glutamyl-phosphate%20reductase%20(EC%201.2.1.38)&subsystem_name=Arginine_Biosynthesis_--_gjo) |
| Amino Acids and Derivatives | Arginine; urea cycle, polyamines | [Arginine Biosynthesis -- gjo](https://rast.nmpdr.org/seedviewer.cgi?page=Subsystems&subsystem=Arginine_Biosynthesis_--_gjo&organism=549.408) | [Ornithine carbamoyltransferase (EC 2.1.3.3)](https://rast.nmpdr.org/seedviewer.cgi?page=FunctionalRole&role=Ornithine%20carbamoyltransferase%20(EC%202.1.3.3)&subsystem_name=Arginine_Biosynthesis_--_gjo) |
| Amino Acids and Derivatives | Arginine; urea cycle, polyamines | [Arginine Biosynthesis -- gjo](https://rast.nmpdr.org/seedviewer.cgi?page=Subsystems&subsystem=Arginine_Biosynthesis_--_gjo&organism=549.408) | [Acetylornithine aminotransferase (EC 2.6.1.11)](https://rast.nmpdr.org/seedviewer.cgi?page=FunctionalRole&role=Acetylornithine%20aminotransferase%20(EC%202.6.1.11)&subsystem_name=Arginine_Biosynthesis_--_gjo) |
| Amino Acids and Derivatives | Arginine; urea cycle, polyamines | [Urea decomposition](https://rast.nmpdr.org/seedviewer.cgi?page=Subsystems&subsystem=Urea_decomposition&organism=549.408) | [Urea carboxylase-related ABC transporter, permease protein](https://rast.nmpdr.org/seedviewer.cgi?page=FunctionalRole&role=Urea%20carboxylase-related%20ABC%20transporter,%20permease%20protein&subsystem_name=Urea_decomposition) |
| Amino Acids and Derivatives | Arginine; urea cycle, polyamines | [Urea decomposition](https://rast.nmpdr.org/seedviewer.cgi?page=Subsystems&subsystem=Urea_decomposition&organism=549.408) | [Urea carboxylase-related ABC transporter, ATPase protein](https://rast.nmpdr.org/seedviewer.cgi?page=FunctionalRole&role=Urea%20carboxylase-related%20ABC%20transporter,%20ATPase%20protein&subsystem_name=Urea_decomposition) |
| Amino Acids and Derivatives | Arginine; urea cycle, polyamines | [Urea decomposition](https://rast.nmpdr.org/seedviewer.cgi?page=Subsystems&subsystem=Urea_decomposition&organism=549.408) | [Urea ABC transporter, permease protein UrtC](https://rast.nmpdr.org/seedviewer.cgi?page=FunctionalRole&role=Urea%20ABC%20transporter,%20permease%20protein%20UrtC&subsystem_name=Urea_decomposition) |
| Amino Acids and Derivatives | Arginine; urea cycle, polyamines | [Urea decomposition](https://rast.nmpdr.org/seedviewer.cgi?page=Subsystems&subsystem=Urea_decomposition&organism=549.408) | [Allophanate hydrolase (EC 3.5.1.54)](https://rast.nmpdr.org/seedviewer.cgi?page=FunctionalRole&role=Allophanate%20hydrolase%20(EC%203.5.1.54)&subsystem_name=Urea_decomposition) |
| Amino Acids and Derivatives | Arginine; urea cycle, polyamines | [Urea decomposition](https://rast.nmpdr.org/seedviewer.cgi?page=Subsystems&subsystem=Urea_decomposition&organism=549.408) | [Urea carboxylase-related aminomethyltransferase (EC 2.1.2.10)](https://rast.nmpdr.org/seedviewer.cgi?page=FunctionalRole&role=Urea%20carboxylase-related%20aminomethyltransferase%20(EC%202.1.2.10)&subsystem_name=Urea_decomposition) |
| Amino Acids and Derivatives | Arginine; urea cycle, polyamines | [Urea decomposition](https://rast.nmpdr.org/seedviewer.cgi?page=Subsystems&subsystem=Urea_decomposition&organism=549.408) | [Urea carboxylase-related ABC transporter, periplasmic substrate-binding protein](https://rast.nmpdr.org/seedviewer.cgi?page=FunctionalRole&role=Urea%20carboxylase-related%20ABC%20transporter,%20periplasmic%20substrate-binding%20protein&subsystem_name=Urea_decomposition) |
| Amino Acids and Derivatives | Arginine; urea cycle, polyamines | [Urea decomposition](https://rast.nmpdr.org/seedviewer.cgi?page=Subsystems&subsystem=Urea_decomposition&organism=549.408) | [Urea ABC transporter, ATPase protein UrtE](https://rast.nmpdr.org/seedviewer.cgi?page=FunctionalRole&role=Urea%20ABC%20transporter,%20ATPase%20protein%20UrtE&subsystem_name=Urea_decomposition) |
| Amino Acids and Derivatives | Arginine; urea cycle, polyamines | [Urea decomposition](https://rast.nmpdr.org/seedviewer.cgi?page=Subsystems&subsystem=Urea_decomposition&organism=549.408) | [Urea ABC transporter, ATPase protein UrtD](https://rast.nmpdr.org/seedviewer.cgi?page=FunctionalRole&role=Urea%20ABC%20transporter,%20ATPase%20protein%20UrtD&subsystem_name=Urea_decomposition) |
| Amino Acids and Derivatives | Arginine; urea cycle, polyamines | [Urea decomposition](https://rast.nmpdr.org/seedviewer.cgi?page=Subsystems&subsystem=Urea_decomposition&organism=549.408) | [Urea carboxylase (EC 6.3.4.6)](https://rast.nmpdr.org/seedviewer.cgi?page=FunctionalRole&role=Urea%20carboxylase%20(EC%206.3.4.6)&subsystem_name=Urea_decomposition) |
| Amino Acids and Derivatives | Arginine; urea cycle, polyamines | [Urea decomposition](https://rast.nmpdr.org/seedviewer.cgi?page=Subsystems&subsystem=Urea_decomposition&organism=549.408) | [Urea ABC transporter, permease protein UrtB](https://rast.nmpdr.org/seedviewer.cgi?page=FunctionalRole&role=Urea%20ABC%20transporter,%20permease%20protein%20UrtB&subsystem_name=Urea_decomposition) |
| Amino Acids and Derivatives | Arginine; urea cycle, polyamines | [Urea carboxylase and Allophanate hydrolase cluster](https://rast.nmpdr.org/seedviewer.cgi?page=Subsystems&subsystem=Urea_carboxylase_and_Allophanate_hydrolase_cluster&organism=549.408) | [Allophanate hydrolase 2 subunit 1 (EC 3.5.1.54)](https://rast.nmpdr.org/seedviewer.cgi?page=FunctionalRole&role=Allophanate%20hydrolase%202%20subunit%201%20(EC%203.5.1.54)&subsystem_name=Urea_carboxylase_and_Allophanate_hydrolase_cluster) |
| Amino Acids and Derivatives | Arginine; urea cycle, polyamines | [Urea carboxylase and Allophanate hydrolase cluster](https://rast.nmpdr.org/seedviewer.cgi?page=Subsystems&subsystem=Urea_carboxylase_and_Allophanate_hydrolase_cluster&organism=549.408) | [Lactam utilization protein LamB](https://rast.nmpdr.org/seedviewer.cgi?page=FunctionalRole&role=Lactam%20utilization%20protein%20LamB&subsystem_name=Urea_carboxylase_and_Allophanate_hydrolase_cluster) |
| Amino Acids and Derivatives | Arginine; urea cycle, polyamines | [Urea carboxylase and Allophanate hydrolase cluster](https://rast.nmpdr.org/seedviewer.cgi?page=Subsystems&subsystem=Urea_carboxylase_and_Allophanate_hydrolase_cluster&organism=549.408) | [Allophanate hydrolase (EC 3.5.1.54)](https://rast.nmpdr.org/seedviewer.cgi?page=FunctionalRole&role=Allophanate%20hydrolase%20(EC%203.5.1.54)&subsystem_name=Urea_carboxylase_and_Allophanate_hydrolase_cluster) |
| Amino Acids and Derivatives | Arginine; urea cycle, polyamines | [Urea carboxylase and Allophanate hydrolase cluster](https://rast.nmpdr.org/seedviewer.cgi?page=Subsystems&subsystem=Urea_carboxylase_and_Allophanate_hydrolase_cluster&organism=549.408) | [Allophanate hydrolase 2 subunit 2 (EC 3.5.1.54)](https://rast.nmpdr.org/seedviewer.cgi?page=FunctionalRole&role=Allophanate%20hydrolase%202%20subunit%202%20(EC%203.5.1.54)&subsystem_name=Urea_carboxylase_and_Allophanate_hydrolase_cluster) |
| Amino Acids and Derivatives | Arginine; urea cycle, polyamines | [Urea carboxylase and Allophanate hydrolase cluster](https://rast.nmpdr.org/seedviewer.cgi?page=Subsystems&subsystem=Urea_carboxylase_and_Allophanate_hydrolase_cluster&organism=549.408) | [Urea carboxylase (EC 6.3.4.6)](https://rast.nmpdr.org/seedviewer.cgi?page=FunctionalRole&role=Urea%20carboxylase%20(EC%206.3.4.6)&subsystem_name=Urea_carboxylase_and_Allophanate_hydrolase_cluster) |
| Amino Acids and Derivatives | Lysine, threonine, methionine, and cysteine | [S-methylmethionine](https://rast.nmpdr.org/seedviewer.cgi?page=Subsystems&subsystem=S-methylmethionine&organism=549.408) | [Homocysteine S-methyltransferase (EC 2.1.1.10)](https://rast.nmpdr.org/seedviewer.cgi?page=FunctionalRole&role=Homocysteine%20S-methyltransferase%20(EC%202.1.1.10)&subsystem_name=S-methylmethionine) |
| Amino Acids and Derivatives | Lysine, threonine, methionine, and cysteine | [S-methylmethionine](https://rast.nmpdr.org/seedviewer.cgi?page=Subsystems&subsystem=S-methylmethionine&organism=549.408) | [S-methylmethionine permease](https://rast.nmpdr.org/seedviewer.cgi?page=FunctionalRole&role=S-methylmethionine%20permease&subsystem_name=S-methylmethionine) |
| Amino Acids and Derivatives | Lysine, threonine, methionine, and cysteine | [Methionine Biosynthesis](https://rast.nmpdr.org/seedviewer.cgi?page=Subsystems&subsystem=Methionine_Biosynthesis&organism=549.408) | [Methionine ABC transporter ATP-binding protein](https://rast.nmpdr.org/seedviewer.cgi?page=FunctionalRole&role=Methionine%20ABC%20transporter%20ATP-binding%20protein&subsystem_name=Methionine_Biosynthesis) |
| Amino Acids and Derivatives | Lysine, threonine, methionine, and cysteine | [Methionine Biosynthesis](https://rast.nmpdr.org/seedviewer.cgi?page=Subsystems&subsystem=Methionine_Biosynthesis&organism=549.408) | [S-adenosylmethionine synthetase (EC 2.5.1.6)](https://rast.nmpdr.org/seedviewer.cgi?page=FunctionalRole&role=S-adenosylmethionine%20synthetase%20(EC%202.5.1.6)&subsystem_name=Methionine_Biosynthesis) |
| Amino Acids and Derivatives | Lysine, threonine, methionine, and cysteine | [Methionine Biosynthesis](https://rast.nmpdr.org/seedviewer.cgi?page=Subsystems&subsystem=Methionine_Biosynthesis&organism=549.408) | [Serine acetyltransferase (EC 2.3.1.30)](https://rast.nmpdr.org/seedviewer.cgi?page=FunctionalRole&role=Serine%20acetyltransferase%20(EC%202.3.1.30)&subsystem_name=Methionine_Biosynthesis) |
| Amino Acids and Derivatives | Lysine, threonine, methionine, and cysteine | [Methionine Biosynthesis](https://rast.nmpdr.org/seedviewer.cgi?page=Subsystems&subsystem=Methionine_Biosynthesis&organism=549.408) | [Homoserine kinase (EC 2.7.1.39)](https://rast.nmpdr.org/seedviewer.cgi?page=FunctionalRole&role=Homoserine%20kinase%20(EC%202.7.1.39)&subsystem_name=Methionine_Biosynthesis) |
| Amino Acids and Derivatives | Lysine, threonine, methionine, and cysteine | [Methionine Biosynthesis](https://rast.nmpdr.org/seedviewer.cgi?page=Subsystems&subsystem=Methionine_Biosynthesis&organism=549.408) | [Homoserine O-succinyltransferase (EC 2.3.1.46)](https://rast.nmpdr.org/seedviewer.cgi?page=FunctionalRole&role=Homoserine%20O-succinyltransferase%20(EC%202.3.1.46)&subsystem_name=Methionine_Biosynthesis) |
| Amino Acids and Derivatives | Lysine, threonine, methionine, and cysteine | [Methionine Biosynthesis](https://rast.nmpdr.org/seedviewer.cgi?page=Subsystems&subsystem=Methionine_Biosynthesis&organism=549.408) | [5-methyltetrahydropteroyltriglutamate--homocysteine methyltransferase (EC 2.1.1.14)](https://rast.nmpdr.org/seedviewer.cgi?page=FunctionalRole&role=5-methyltetrahydropteroyltriglutamate--homocysteine%20methyltransferase%20(EC%202.1.1.14)&subsystem_name=Methionine_Biosynthesis) |
| Amino Acids and Derivatives | Lysine, threonine, methionine, and cysteine | [Methionine Biosynthesis](https://rast.nmpdr.org/seedviewer.cgi?page=Subsystems&subsystem=Methionine_Biosynthesis&organism=549.408) | [5-methyltetrahydrofolate--homocysteine methyltransferase (EC 2.1.1.13)](https://rast.nmpdr.org/seedviewer.cgi?page=FunctionalRole&role=5-methyltetrahydrofolate--homocysteine%20methyltransferase%20(EC%202.1.1.13)&subsystem_name=Methionine_Biosynthesis) |
| Amino Acids and Derivatives | Lysine, threonine, methionine, and cysteine | [Methionine Biosynthesis](https://rast.nmpdr.org/seedviewer.cgi?page=Subsystems&subsystem=Methionine_Biosynthesis&organism=549.408) | [Cystathionine beta-lyase, type II (EC 4.4.1.8)](https://rast.nmpdr.org/seedviewer.cgi?page=FunctionalRole&role=Cystathionine%20beta-lyase,%20type%20II%20(EC%204.4.1.8)&subsystem_name=Methionine_Biosynthesis) |
| Amino Acids and Derivatives | Lysine, threonine, methionine, and cysteine | [Methionine Biosynthesis](https://rast.nmpdr.org/seedviewer.cgi?page=Subsystems&subsystem=Methionine_Biosynthesis&organism=549.408) | [Cysteine synthase (EC 2.5.1.47)](https://rast.nmpdr.org/seedviewer.cgi?page=FunctionalRole&role=Cysteine%20synthase%20(EC%202.5.1.47)&subsystem_name=Methionine_Biosynthesis) |
| Amino Acids and Derivatives | Lysine, threonine, methionine, and cysteine | [Methionine Biosynthesis](https://rast.nmpdr.org/seedviewer.cgi?page=Subsystems&subsystem=Methionine_Biosynthesis&organism=549.408) | [S-ribosylhomocysteine lyase (EC 4.4.1.21)](https://rast.nmpdr.org/seedviewer.cgi?page=FunctionalRole&role=S-ribosylhomocysteine%20lyase%20(EC%204.4.1.21)&subsystem_name=Methionine_Biosynthesis) |
| Amino Acids and Derivatives | Lysine, threonine, methionine, and cysteine | [Methionine Biosynthesis](https://rast.nmpdr.org/seedviewer.cgi?page=Subsystems&subsystem=Methionine_Biosynthesis&organism=549.408) | [5,10-methylenetetrahydrofolate reductase (EC 1.5.1.20)](https://rast.nmpdr.org/seedviewer.cgi?page=FunctionalRole&role=5,10-methylenetetrahydrofolate%20reductase%20(EC%201.5.1.20)&subsystem_name=Methionine_Biosynthesis) |
| Amino Acids and Derivatives | Lysine, threonine, methionine, and cysteine | [Methionine Biosynthesis](https://rast.nmpdr.org/seedviewer.cgi?page=Subsystems&subsystem=Methionine_Biosynthesis&organism=549.408) | [Homocysteine S-methyltransferase (EC 2.1.1.10)](https://rast.nmpdr.org/seedviewer.cgi?page=FunctionalRole&role=Homocysteine%20S-methyltransferase%20(EC%202.1.1.10)&subsystem_name=Methionine_Biosynthesis) |
| Amino Acids and Derivatives | Lysine, threonine, methionine, and cysteine | [Methionine Biosynthesis](https://rast.nmpdr.org/seedviewer.cgi?page=Subsystems&subsystem=Methionine_Biosynthesis&organism=549.408) | [Homoserine dehydrogenase (EC 1.1.1.3)](https://rast.nmpdr.org/seedviewer.cgi?page=FunctionalRole&role=Homoserine%20dehydrogenase%20(EC%201.1.1.3)&subsystem_name=Methionine_Biosynthesis) |
| Amino Acids and Derivatives | Lysine, threonine, methionine, and cysteine | [Methionine Biosynthesis](https://rast.nmpdr.org/seedviewer.cgi?page=Subsystems&subsystem=Methionine_Biosynthesis&organism=549.408) | [Methionine ABC transporter substrate-binding protein](https://rast.nmpdr.org/seedviewer.cgi?page=FunctionalRole&role=Methionine%20ABC%20transporter%20substrate-binding%20protein&subsystem_name=Methionine_Biosynthesis) |
| Amino Acids and Derivatives | Lysine, threonine, methionine, and cysteine | [Methionine Biosynthesis](https://rast.nmpdr.org/seedviewer.cgi?page=Subsystems&subsystem=Methionine_Biosynthesis&organism=549.408) | [Cystathionine gamma-synthase (EC 2.5.1.48)](https://rast.nmpdr.org/seedviewer.cgi?page=FunctionalRole&role=Cystathionine%20gamma-synthase%20(EC%202.5.1.48)&subsystem_name=Methionine_Biosynthesis) |
| Amino Acids and Derivatives | Lysine, threonine, methionine, and cysteine | [Methionine Biosynthesis](https://rast.nmpdr.org/seedviewer.cgi?page=Subsystems&subsystem=Methionine_Biosynthesis&organism=549.408) | [Cystathionine beta-lyase (EC 4.4.1.8)](https://rast.nmpdr.org/seedviewer.cgi?page=FunctionalRole&role=Cystathionine%20beta-lyase%20(EC%204.4.1.8)&subsystem_name=Methionine_Biosynthesis) |
| Amino Acids and Derivatives | Lysine, threonine, methionine, and cysteine | [Methionine Biosynthesis](https://rast.nmpdr.org/seedviewer.cgi?page=Subsystems&subsystem=Methionine_Biosynthesis&organism=549.408) | [S-adenosylhomocysteine nucleosidase (EC 3.2.2.9)](https://rast.nmpdr.org/seedviewer.cgi?page=FunctionalRole&role=S-adenosylhomocysteine%20nucleosidase%20(EC%203.2.2.9)&subsystem_name=Methionine_Biosynthesis) |
| Amino Acids and Derivatives | Lysine, threonine, methionine, and cysteine | [Methionine Biosynthesis](https://rast.nmpdr.org/seedviewer.cgi?page=Subsystems&subsystem=Methionine_Biosynthesis&organism=549.408) | [Methionine ABC transporter permease protein](https://rast.nmpdr.org/seedviewer.cgi?page=FunctionalRole&role=Methionine%20ABC%20transporter%20permease%20protein&subsystem_name=Methionine_Biosynthesis) |
| Amino Acids and Derivatives | Lysine, threonine, methionine, and cysteine | [Methionine Biosynthesis](https://rast.nmpdr.org/seedviewer.cgi?page=Subsystems&subsystem=Methionine_Biosynthesis&organism=549.408) | [Transcriptional activator MetR](https://rast.nmpdr.org/seedviewer.cgi?page=FunctionalRole&role=Transcriptional%20activator%20MetR&subsystem_name=Methionine_Biosynthesis) |
| Amino Acids and Derivatives | Lysine, threonine, methionine, and cysteine | [Methionine Biosynthesis](https://rast.nmpdr.org/seedviewer.cgi?page=Subsystems&subsystem=Methionine_Biosynthesis&organism=549.408) | [Methionine repressor MetJ](https://rast.nmpdr.org/seedviewer.cgi?page=FunctionalRole&role=Methionine%20repressor%20MetJ&subsystem_name=Methionine_Biosynthesis) |
| Amino Acids and Derivatives | Lysine, threonine, methionine, and cysteine | [Threonine degradation](https://rast.nmpdr.org/seedviewer.cgi?page=Subsystems&subsystem=Threonine_degradation&organism=549.408) | [Threonine dehydrogenase and related Zn-dependent dehydrogenases](https://rast.nmpdr.org/seedviewer.cgi?page=FunctionalRole&role=Threonine%20dehydrogenase%20and%20related%20Zn-dependent%20dehydrogenases&subsystem_name=Threonine_degradation) |
| Amino Acids and Derivatives | Lysine, threonine, methionine, and cysteine | [Threonine degradation](https://rast.nmpdr.org/seedviewer.cgi?page=Subsystems&subsystem=Threonine_degradation&organism=549.408) | [Transcriptional regulator GabR of GABA utilization (GntR family with aminotransferase-like domain)](https://rast.nmpdr.org/seedviewer.cgi?page=FunctionalRole&role=Transcriptional%20regulator%20GabR%20of%20GABA%20utilization%20(GntR%20family%20with%20aminotransferase-like%20domain)&subsystem_name=Threonine_degradation) |
| Amino Acids and Derivatives | Lysine, threonine, methionine, and cysteine | [Threonine degradation](https://rast.nmpdr.org/seedviewer.cgi?page=Subsystems&subsystem=Threonine_degradation&organism=549.408) | [low-specificity D-threonine aldolase](https://rast.nmpdr.org/seedviewer.cgi?page=FunctionalRole&role=low-specificity%20D-threonine%20aldolase&subsystem_name=Threonine_degradation) |
| Amino Acids and Derivatives | Lysine, threonine, methionine, and cysteine | [Threonine degradation](https://rast.nmpdr.org/seedviewer.cgi?page=Subsystems&subsystem=Threonine_degradation&organism=549.408) | [Threonine dehydratase, catabolic (EC 4.3.1.19)](https://rast.nmpdr.org/seedviewer.cgi?page=FunctionalRole&role=Threonine%20dehydratase,%20catabolic%20(EC%204.3.1.19)&subsystem_name=Threonine_degradation) |
| Amino Acids and Derivatives | Lysine, threonine, methionine, and cysteine | [Threonine degradation](https://rast.nmpdr.org/seedviewer.cgi?page=Subsystems&subsystem=Threonine_degradation&organism=549.408) | [L-threonine 3-dehydrogenase (EC 1.1.1.103)](https://rast.nmpdr.org/seedviewer.cgi?page=FunctionalRole&role=L-threonine%203-dehydrogenase%20(EC%201.1.1.103)&subsystem_name=Threonine_degradation) |
| Amino Acids and Derivatives | Lysine, threonine, methionine, and cysteine | [Lysine Biosynthesis DAP Pathway, GJO scratch](https://rast.nmpdr.org/seedviewer.cgi?page=Subsystems&subsystem=Lysine_Biosynthesis_DAP_Pathway,_GJO_scratch&organism=549.408) | [N-succinyl-L,L-diaminopimelate desuccinylase (EC 3.5.1.18)](https://rast.nmpdr.org/seedviewer.cgi?page=FunctionalRole&role=N-succinyl-L,L-diaminopimelate%20desuccinylase%20(EC%203.5.1.18)&subsystem_name=Lysine_Biosynthesis_DAP_Pathway,_GJO_scratch) |
| Amino Acids and Derivatives | Lysine, threonine, methionine, and cysteine | [Lysine Biosynthesis DAP Pathway, GJO scratch](https://rast.nmpdr.org/seedviewer.cgi?page=Subsystems&subsystem=Lysine_Biosynthesis_DAP_Pathway,_GJO_scratch&organism=549.408) | [N-acetyl-L,L-diaminopimelate deacetylase (EC 3.5.1.47)](https://rast.nmpdr.org/seedviewer.cgi?page=FunctionalRole&role=N-acetyl-L,L-diaminopimelate%20deacetylase%20(EC%203.5.1.47)&subsystem_name=Lysine_Biosynthesis_DAP_Pathway,_GJO_scratch) |
| Amino Acids and Derivatives | Lysine, threonine, methionine, and cysteine | [Lysine Biosynthesis DAP Pathway, GJO scratch](https://rast.nmpdr.org/seedviewer.cgi?page=Subsystems&subsystem=Lysine_Biosynthesis_DAP_Pathway,_GJO_scratch&organism=549.408) | [2,3,4,5-tetrahydropyridine-2,6-dicarboxylate N-succinyltransferase (EC 2.3.1.117)](https://rast.nmpdr.org/seedviewer.cgi?page=FunctionalRole&role=2,3,4,5-tetrahydropyridine-2,6-dicarboxylate%20N-succinyltransferase%20(EC%202.3.1.117)&subsystem_name=Lysine_Biosynthesis_DAP_Pathway,_GJO_scratch) |
| Amino Acids and Derivatives | Lysine, threonine, methionine, and cysteine | [Lysine Biosynthesis DAP Pathway, GJO scratch](https://rast.nmpdr.org/seedviewer.cgi?page=Subsystems&subsystem=Lysine_Biosynthesis_DAP_Pathway,_GJO_scratch&organism=549.408) | [Diaminopimelate epimerase (EC 5.1.1.7)](https://rast.nmpdr.org/seedviewer.cgi?page=FunctionalRole&role=Diaminopimelate%20epimerase%20(EC%205.1.1.7)&subsystem_name=Lysine_Biosynthesis_DAP_Pathway,_GJO_scratch) |
| Amino Acids and Derivatives | Lysine, threonine, methionine, and cysteine | [Lysine Biosynthesis DAP Pathway, GJO scratch](https://rast.nmpdr.org/seedviewer.cgi?page=Subsystems&subsystem=Lysine_Biosynthesis_DAP_Pathway,_GJO_scratch&organism=549.408) | [Aspartate-semialdehyde dehydrogenase (EC 1.2.1.11)](https://rast.nmpdr.org/seedviewer.cgi?page=FunctionalRole&role=Aspartate-semialdehyde%20dehydrogenase%20(EC%201.2.1.11)&subsystem_name=Lysine_Biosynthesis_DAP_Pathway,_GJO_scratch) |
| Amino Acids and Derivatives | Lysine, threonine, methionine, and cysteine | [Lysine Biosynthesis DAP Pathway, GJO scratch](https://rast.nmpdr.org/seedviewer.cgi?page=Subsystems&subsystem=Lysine_Biosynthesis_DAP_Pathway,_GJO_scratch&organism=549.408) | [4-hydroxy-tetrahydrodipicolinate synthase (EC 4.3.3.7)](https://rast.nmpdr.org/seedviewer.cgi?page=FunctionalRole&role=4-hydroxy-tetrahydrodipicolinate%20synthase%20(EC%204.3.3.7)&subsystem_name=Lysine_Biosynthesis_DAP_Pathway,_GJO_scratch) |
| Amino Acids and Derivatives | Lysine, threonine, methionine, and cysteine | [Lysine Biosynthesis DAP Pathway, GJO scratch](https://rast.nmpdr.org/seedviewer.cgi?page=Subsystems&subsystem=Lysine_Biosynthesis_DAP_Pathway,_GJO_scratch&organism=549.408) | [Diaminopimelate decarboxylase (EC 4.1.1.20)](https://rast.nmpdr.org/seedviewer.cgi?page=FunctionalRole&role=Diaminopimelate%20decarboxylase%20(EC%204.1.1.20)&subsystem_name=Lysine_Biosynthesis_DAP_Pathway,_GJO_scratch) |
| Amino Acids and Derivatives | Lysine, threonine, methionine, and cysteine | [Lysine Biosynthesis DAP Pathway, GJO scratch](https://rast.nmpdr.org/seedviewer.cgi?page=Subsystems&subsystem=Lysine_Biosynthesis_DAP_Pathway,_GJO_scratch&organism=549.408) | [4-hydroxy-tetrahydrodipicolinate reductase (EC 1.17.1.8)](https://rast.nmpdr.org/seedviewer.cgi?page=FunctionalRole&role=4-hydroxy-tetrahydrodipicolinate%20reductase%20(EC%201.17.1.8)&subsystem_name=Lysine_Biosynthesis_DAP_Pathway,_GJO_scratch) |
| Amino Acids and Derivatives | Lysine, threonine, methionine, and cysteine | [Lysine Biosynthesis DAP Pathway, GJO scratch](https://rast.nmpdr.org/seedviewer.cgi?page=Subsystems&subsystem=Lysine_Biosynthesis_DAP_Pathway,_GJO_scratch&organism=549.408) | [N-succinyl-L,L-diaminopimelate aminotransferase (EC 2.6.1.17)](https://rast.nmpdr.org/seedviewer.cgi?page=FunctionalRole&role=N-succinyl-L,L-diaminopimelate%20aminotransferase%20(EC%202.6.1.17)&subsystem_name=Lysine_Biosynthesis_DAP_Pathway,_GJO_scratch) |
| Amino Acids and Derivatives | Lysine, threonine, methionine, and cysteine | [Lysine Biosynthesis DAP Pathway, GJO scratch](https://rast.nmpdr.org/seedviewer.cgi?page=Subsystems&subsystem=Lysine_Biosynthesis_DAP_Pathway,_GJO_scratch&organism=549.408) | [Aspartokinase (EC 2.7.2.4)](https://rast.nmpdr.org/seedviewer.cgi?page=FunctionalRole&role=Aspartokinase%20(EC%202.7.2.4)&subsystem_name=Lysine_Biosynthesis_DAP_Pathway,_GJO_scratch) |
| Amino Acids and Derivatives | Lysine, threonine, methionine, and cysteine | [Methionine Degradation](https://rast.nmpdr.org/seedviewer.cgi?page=Subsystems&subsystem=Methionine_Degradation&organism=549.408) | [Methionine ABC transporter substrate-binding protein](https://rast.nmpdr.org/seedviewer.cgi?page=FunctionalRole&role=Methionine%20ABC%20transporter%20substrate-binding%20protein&subsystem_name=Methionine_Degradation) |
| Amino Acids and Derivatives | Lysine, threonine, methionine, and cysteine | [Methionine Degradation](https://rast.nmpdr.org/seedviewer.cgi?page=Subsystems&subsystem=Methionine_Degradation&organism=549.408) | [Methionine ABC transporter ATP-binding protein](https://rast.nmpdr.org/seedviewer.cgi?page=FunctionalRole&role=Methionine%20ABC%20transporter%20ATP-binding%20protein&subsystem_name=Methionine_Degradation) |
| Amino Acids and Derivatives | Lysine, threonine, methionine, and cysteine | [Methionine Degradation](https://rast.nmpdr.org/seedviewer.cgi?page=Subsystems&subsystem=Methionine_Degradation&organism=549.408) | [S-adenosylmethionine synthetase (EC 2.5.1.6)](https://rast.nmpdr.org/seedviewer.cgi?page=FunctionalRole&role=S-adenosylmethionine%20synthetase%20(EC%202.5.1.6)&subsystem_name=Methionine_Degradation) |
| Amino Acids and Derivatives | Lysine, threonine, methionine, and cysteine | [Methionine Degradation](https://rast.nmpdr.org/seedviewer.cgi?page=Subsystems&subsystem=Methionine_Degradation&organism=549.408) | [Pyruvate dehydrogenase E1 component (EC 1.2.4.1)](https://rast.nmpdr.org/seedviewer.cgi?page=FunctionalRole&role=Pyruvate%20dehydrogenase%20E1%20component%20(EC%201.2.4.1)&subsystem_name=Methionine_Degradation) |
| Amino Acids and Derivatives | Lysine, threonine, methionine, and cysteine | [Methionine Degradation](https://rast.nmpdr.org/seedviewer.cgi?page=Subsystems&subsystem=Methionine_Degradation&organism=549.408) | [S-adenosylhomocysteine nucleosidase (EC 3.2.2.9)](https://rast.nmpdr.org/seedviewer.cgi?page=FunctionalRole&role=S-adenosylhomocysteine%20nucleosidase%20(EC%203.2.2.9)&subsystem_name=Methionine_Degradation) |
| Amino Acids and Derivatives | Lysine, threonine, methionine, and cysteine | [Methionine Degradation](https://rast.nmpdr.org/seedviewer.cgi?page=Subsystems&subsystem=Methionine_Degradation&organism=549.408) | [Methionine ABC transporter permease protein](https://rast.nmpdr.org/seedviewer.cgi?page=FunctionalRole&role=Methionine%20ABC%20transporter%20permease%20protein&subsystem_name=Methionine_Degradation) |
| Amino Acids and Derivatives | Lysine, threonine, methionine, and cysteine | [Methionine Degradation](https://rast.nmpdr.org/seedviewer.cgi?page=Subsystems&subsystem=Methionine_Degradation&organism=549.408) | [S-ribosylhomocysteine lyase (EC 4.4.1.21)](https://rast.nmpdr.org/seedviewer.cgi?page=FunctionalRole&role=S-ribosylhomocysteine%20lyase%20(EC%204.4.1.21)&subsystem_name=Methionine_Degradation) |
| Amino Acids and Derivatives | Lysine, threonine, methionine, and cysteine | [Methionine Degradation](https://rast.nmpdr.org/seedviewer.cgi?page=Subsystems&subsystem=Methionine_Degradation&organism=549.408) | [2-Oxobutyrate oxidase, putative](https://rast.nmpdr.org/seedviewer.cgi?page=FunctionalRole&role=2-Oxobutyrate%20oxidase,%20putative&subsystem_name=Methionine_Degradation) |
| Amino Acids and Derivatives | Lysine, threonine, methionine, and cysteine | [Threonine and Homoserine Biosynthesis](https://rast.nmpdr.org/seedviewer.cgi?page=Subsystems&subsystem=Threonine_and_Homoserine_Biosynthesis&organism=549.408) | [Homoserine dehydrogenase (EC 1.1.1.3)](https://rast.nmpdr.org/seedviewer.cgi?page=FunctionalRole&role=Homoserine%20dehydrogenase%20(EC%201.1.1.3)&subsystem_name=Threonine_and_Homoserine_Biosynthesis) |
| Amino Acids and Derivatives | Lysine, threonine, methionine, and cysteine | [Threonine and Homoserine Biosynthesis](https://rast.nmpdr.org/seedviewer.cgi?page=Subsystems&subsystem=Threonine_and_Homoserine_Biosynthesis&organism=549.408) | [Aspartate-semialdehyde dehydrogenase (EC 1.2.1.11)](https://rast.nmpdr.org/seedviewer.cgi?page=FunctionalRole&role=Aspartate-semialdehyde%20dehydrogenase%20(EC%201.2.1.11)&subsystem_name=Threonine_and_Homoserine_Biosynthesis) |
| Amino Acids and Derivatives | Lysine, threonine, methionine, and cysteine | [Threonine and Homoserine Biosynthesis](https://rast.nmpdr.org/seedviewer.cgi?page=Subsystems&subsystem=Threonine_and_Homoserine_Biosynthesis&organism=549.408) | [Aspartate aminotransferase (EC 2.6.1.1)](https://rast.nmpdr.org/seedviewer.cgi?page=FunctionalRole&role=Aspartate%20aminotransferase%20(EC%202.6.1.1)&subsystem_name=Threonine_and_Homoserine_Biosynthesis) |
| Amino Acids and Derivatives | Lysine, threonine, methionine, and cysteine | [Threonine and Homoserine Biosynthesis](https://rast.nmpdr.org/seedviewer.cgi?page=Subsystems&subsystem=Threonine_and_Homoserine_Biosynthesis&organism=549.408) | [Threonine synthase (EC 4.2.3.1)](https://rast.nmpdr.org/seedviewer.cgi?page=FunctionalRole&role=Threonine%20synthase%20(EC%204.2.3.1)&subsystem_name=Threonine_and_Homoserine_Biosynthesis) |
| Amino Acids and Derivatives | Lysine, threonine, methionine, and cysteine | [Threonine and Homoserine Biosynthesis](https://rast.nmpdr.org/seedviewer.cgi?page=Subsystems&subsystem=Threonine_and_Homoserine_Biosynthesis&organism=549.408) | [Homoserine kinase (EC 2.7.1.39)](https://rast.nmpdr.org/seedviewer.cgi?page=FunctionalRole&role=Homoserine%20kinase%20(EC%202.7.1.39)&subsystem_name=Threonine_and_Homoserine_Biosynthesis) |
| Amino Acids and Derivatives | Lysine, threonine, methionine, and cysteine | [Threonine and Homoserine Biosynthesis](https://rast.nmpdr.org/seedviewer.cgi?page=Subsystems&subsystem=Threonine_and_Homoserine_Biosynthesis&organism=549.408) | [Aspartokinase (EC 2.7.2.4)](https://rast.nmpdr.org/seedviewer.cgi?page=FunctionalRole&role=Aspartokinase%20(EC%202.7.2.4)&subsystem_name=Threonine_and_Homoserine_Biosynthesis) |
| Amino Acids and Derivatives | Lysine, threonine, methionine, and cysteine | [Methionine Salvage](https://rast.nmpdr.org/seedviewer.cgi?page=Subsystems&subsystem=Methionine_Salvage&organism=549.408) | [5-methylthioribose kinase (EC 2.7.1.100)](https://rast.nmpdr.org/seedviewer.cgi?page=FunctionalRole&role=5-methylthioribose%20kinase%20(EC%202.7.1.100)&subsystem_name=Methionine_Salvage) |
| Amino Acids and Derivatives | Lysine, threonine, methionine, and cysteine | [Methionine Salvage](https://rast.nmpdr.org/seedviewer.cgi?page=Subsystems&subsystem=Methionine_Salvage&organism=549.408) | [Methionine aminotransferase, PLP-dependent](https://rast.nmpdr.org/seedviewer.cgi?page=FunctionalRole&role=Methionine%20aminotransferase,%20PLP-dependent&subsystem_name=Methionine_Salvage) |
| Amino Acids and Derivatives | Lysine, threonine, methionine, and cysteine | [Methionine Salvage](https://rast.nmpdr.org/seedviewer.cgi?page=Subsystems&subsystem=Methionine_Salvage&organism=549.408) | [Methylthioribose-1-phosphate isomerase (EC 5.3.1.23)](https://rast.nmpdr.org/seedviewer.cgi?page=FunctionalRole&role=Methylthioribose-1-phosphate%20isomerase%20(EC%205.3.1.23)&subsystem_name=Methionine_Salvage) |
| Amino Acids and Derivatives | Lysine, threonine, methionine, and cysteine | [Methionine Salvage](https://rast.nmpdr.org/seedviewer.cgi?page=Subsystems&subsystem=Methionine_Salvage&organism=549.408) | [Methylthioribulose-1-phosphate dehydratase (EC 4.2.1.109)](https://rast.nmpdr.org/seedviewer.cgi?page=FunctionalRole&role=Methylthioribulose-1-phosphate%20dehydratase%20(EC%204.2.1.109)&subsystem_name=Methionine_Salvage) |
| Amino Acids and Derivatives | Lysine, threonine, methionine, and cysteine | [Methionine Salvage](https://rast.nmpdr.org/seedviewer.cgi?page=Subsystems&subsystem=Methionine_Salvage&organism=549.408) | [2,3-diketo-5-methylthiopentyl-1-phosphate enolase-phosphatase (EC 3.1.3.77)](https://rast.nmpdr.org/seedviewer.cgi?page=FunctionalRole&role=2,3-diketo-5-methylthiopentyl-1-phosphate%20enolase-phosphatase%20(EC%203.1.3.77)&subsystem_name=Methionine_Salvage) |
| Amino Acids and Derivatives | Lysine, threonine, methionine, and cysteine | [Methionine Salvage](https://rast.nmpdr.org/seedviewer.cgi?page=Subsystems&subsystem=Methionine_Salvage&organism=549.408) | [1,2-dihydroxy-3-keto-5-methylthiopentene dioxygenase (EC 1.13.11.54)](https://rast.nmpdr.org/seedviewer.cgi?page=FunctionalRole&role=1,2-dihydroxy-3-keto-5-methylthiopentene%20dioxygenase%20(EC%201.13.11.54)&subsystem_name=Methionine_Salvage) |
| Amino Acids and Derivatives | Lysine, threonine, methionine, and cysteine | [Methionine Salvage](https://rast.nmpdr.org/seedviewer.cgi?page=Subsystems&subsystem=Methionine_Salvage&organism=549.408) | [5'-methylthioadenosine nucleosidase (EC 3.2.2.16)](https://rast.nmpdr.org/seedviewer.cgi?page=FunctionalRole&role=5) |
| Amino Acids and Derivatives | Lysine, threonine, methionine, and cysteine | [Cysteine Biosynthesis](https://rast.nmpdr.org/seedviewer.cgi?page=Subsystems&subsystem=Cysteine_Biosynthesis&organism=549.408) | [Cys regulon transcriptional activator CysB](https://rast.nmpdr.org/seedviewer.cgi?page=FunctionalRole&role=Cys%20regulon%20transcriptional%20activator%20CysB&subsystem_name=Cysteine_Biosynthesis) |
| Amino Acids and Derivatives | Lysine, threonine, methionine, and cysteine | [Cysteine Biosynthesis](https://rast.nmpdr.org/seedviewer.cgi?page=Subsystems&subsystem=Cysteine_Biosynthesis&organism=549.408) | [Adenylylsulfate kinase (EC 2.7.1.25)](https://rast.nmpdr.org/seedviewer.cgi?page=FunctionalRole&role=Adenylylsulfate%20kinase%20(EC%202.7.1.25)&subsystem_name=Cysteine_Biosynthesis) |
| Amino Acids and Derivatives | Lysine, threonine, methionine, and cysteine | [Cysteine Biosynthesis](https://rast.nmpdr.org/seedviewer.cgi?page=Subsystems&subsystem=Cysteine_Biosynthesis&organism=549.408) | [Sulfate and thiosulfate binding protein CysP](https://rast.nmpdr.org/seedviewer.cgi?page=FunctionalRole&role=Sulfate%20and%20thiosulfate%20binding%20protein%20CysP&subsystem_name=Cysteine_Biosynthesis) |
| Amino Acids and Derivatives | Lysine, threonine, methionine, and cysteine | [Cysteine Biosynthesis](https://rast.nmpdr.org/seedviewer.cgi?page=Subsystems&subsystem=Cysteine_Biosynthesis&organism=549.408) | [Serine acetyltransferase (EC 2.3.1.30)](https://rast.nmpdr.org/seedviewer.cgi?page=FunctionalRole&role=Serine%20acetyltransferase%20(EC%202.3.1.30)&subsystem_name=Cysteine_Biosynthesis) |
| Amino Acids and Derivatives | Lysine, threonine, methionine, and cysteine | [Cysteine Biosynthesis](https://rast.nmpdr.org/seedviewer.cgi?page=Subsystems&subsystem=Cysteine_Biosynthesis&organism=549.408) | [Sulfate transport system permease protein CysW](https://rast.nmpdr.org/seedviewer.cgi?page=FunctionalRole&role=Sulfate%20transport%20system%20permease%20protein%20CysW&subsystem_name=Cysteine_Biosynthesis) |
| Amino Acids and Derivatives | Lysine, threonine, methionine, and cysteine | [Cysteine Biosynthesis](https://rast.nmpdr.org/seedviewer.cgi?page=Subsystems&subsystem=Cysteine_Biosynthesis&organism=549.408) | [Sulfate transport system permease protein CysT](https://rast.nmpdr.org/seedviewer.cgi?page=FunctionalRole&role=Sulfate%20transport%20system%20permease%20protein%20CysT&subsystem_name=Cysteine_Biosynthesis) |
| Amino Acids and Derivatives | Lysine, threonine, methionine, and cysteine | [Cysteine Biosynthesis](https://rast.nmpdr.org/seedviewer.cgi?page=Subsystems&subsystem=Cysteine_Biosynthesis&organism=549.408) | [Cysteine synthase (EC 2.5.1.47)](https://rast.nmpdr.org/seedviewer.cgi?page=FunctionalRole&role=Cysteine%20synthase%20(EC%202.5.1.47)&subsystem_name=Cysteine_Biosynthesis) |
| Amino Acids and Derivatives | Lysine, threonine, methionine, and cysteine | [Cysteine Biosynthesis](https://rast.nmpdr.org/seedviewer.cgi?page=Subsystems&subsystem=Cysteine_Biosynthesis&organism=549.408) | [Phosphoadenylyl-sulfate reductase [thioredoxin] (EC 1.8.4.8)](https://rast.nmpdr.org/seedviewer.cgi?page=FunctionalRole&role=Phosphoadenylyl-sulfate%20reductase%20%5bthioredoxin%5d%20(EC%201.8.4.8)&subsystem_name=Cysteine_Biosynthesis) |
| Amino Acids and Derivatives | Lysine, threonine, methionine, and cysteine | [Cysteine Biosynthesis](https://rast.nmpdr.org/seedviewer.cgi?page=Subsystems&subsystem=Cysteine_Biosynthesis&organism=549.408) | [Sulfate adenylyltransferase subunit 1 (EC 2.7.7.4)](https://rast.nmpdr.org/seedviewer.cgi?page=FunctionalRole&role=Sulfate%20adenylyltransferase%20subunit%201%20(EC%202.7.7.4)&subsystem_name=Cysteine_Biosynthesis) |
| Amino Acids and Derivatives | Lysine, threonine, methionine, and cysteine | [Cysteine Biosynthesis](https://rast.nmpdr.org/seedviewer.cgi?page=Subsystems&subsystem=Cysteine_Biosynthesis&organism=549.408) | [Sulfate adenylyltransferase subunit 2 (EC 2.7.7.4)](https://rast.nmpdr.org/seedviewer.cgi?page=FunctionalRole&role=Sulfate%20adenylyltransferase%20subunit%202%20(EC%202.7.7.4)&subsystem_name=Cysteine_Biosynthesis) |
| Amino Acids and Derivatives | Lysine, threonine, methionine, and cysteine | [Cysteine Biosynthesis](https://rast.nmpdr.org/seedviewer.cgi?page=Subsystems&subsystem=Cysteine_Biosynthesis&organism=549.408) | [Sulfate permease](https://rast.nmpdr.org/seedviewer.cgi?page=FunctionalRole&role=Sulfate%20permease&subsystem_name=Cysteine_Biosynthesis) |
| Amino Acids and Derivatives | Lysine, threonine, methionine, and cysteine | [Cysteine Biosynthesis](https://rast.nmpdr.org/seedviewer.cgi?page=Subsystems&subsystem=Cysteine_Biosynthesis&organism=549.408) | [Sulfite reductase [NADPH] hemoprotein beta-component (EC 1.8.1.2)](https://rast.nmpdr.org/seedviewer.cgi?page=FunctionalRole&role=Sulfite%20reductase%20%5bNADPH%5d%20hemoprotein%20beta-component%20(EC%201.8.1.2)&subsystem_name=Cysteine_Biosynthesis) |
| Amino Acids and Derivatives | Lysine, threonine, methionine, and cysteine | [Cysteine Biosynthesis](https://rast.nmpdr.org/seedviewer.cgi?page=Subsystems&subsystem=Cysteine_Biosynthesis&organism=549.408) | [Sulfate transporter, CysZ-type](https://rast.nmpdr.org/seedviewer.cgi?page=FunctionalRole&role=Sulfate%20transporter,%20CysZ-type&subsystem_name=Cysteine_Biosynthesis) |
| Amino Acids and Derivatives | Lysine, threonine, methionine, and cysteine | [Cysteine Biosynthesis](https://rast.nmpdr.org/seedviewer.cgi?page=Subsystems&subsystem=Cysteine_Biosynthesis&organism=549.408) | [Sulfate and thiosulfate import ATP-binding protein CysA (EC 3.6.3.25)](https://rast.nmpdr.org/seedviewer.cgi?page=FunctionalRole&role=Sulfate%20and%20thiosulfate%20import%20ATP-binding%20protein%20CysA%20(EC%203.6.3.25)&subsystem_name=Cysteine_Biosynthesis) |
| Amino Acids and Derivatives | Lysine, threonine, methionine, and cysteine | [Cysteine Biosynthesis](https://rast.nmpdr.org/seedviewer.cgi?page=Subsystems&subsystem=Cysteine_Biosynthesis&organism=549.408) | [Sulfite reductase [NADPH] flavoprotein alpha-component (EC 1.8.1.2)](https://rast.nmpdr.org/seedviewer.cgi?page=FunctionalRole&role=Sulfite%20reductase%20%5bNADPH%5d%20flavoprotein%20alpha-component%20(EC%201.8.1.2)&subsystem_name=Cysteine_Biosynthesis) |
| Amino Acids and Derivatives | Lysine, threonine, methionine, and cysteine | [Lysine Biosynthesis DAP Pathway](https://rast.nmpdr.org/seedviewer.cgi?page=Subsystems&subsystem=Lysine_Biosynthesis_DAP_Pathway&organism=549.408) | [N-succinyl-L,L-diaminopimelate desuccinylase (EC 3.5.1.18)](https://rast.nmpdr.org/seedviewer.cgi?page=FunctionalRole&role=N-succinyl-L,L-diaminopimelate%20desuccinylase%20(EC%203.5.1.18)&subsystem_name=Lysine_Biosynthesis_DAP_Pathway) |
| Amino Acids and Derivatives | Lysine, threonine, methionine, and cysteine | [Lysine Biosynthesis DAP Pathway](https://rast.nmpdr.org/seedviewer.cgi?page=Subsystems&subsystem=Lysine_Biosynthesis_DAP_Pathway&organism=549.408) | [N-acetyl-L,L-diaminopimelate deacetylase (EC 3.5.1.47)](https://rast.nmpdr.org/seedviewer.cgi?page=FunctionalRole&role=N-acetyl-L,L-diaminopimelate%20deacetylase%20(EC%203.5.1.47)&subsystem_name=Lysine_Biosynthesis_DAP_Pathway) |
| Amino Acids and Derivatives | Lysine, threonine, methionine, and cysteine | [Lysine Biosynthesis DAP Pathway](https://rast.nmpdr.org/seedviewer.cgi?page=Subsystems&subsystem=Lysine_Biosynthesis_DAP_Pathway&organism=549.408) | [2,3,4,5-tetrahydropyridine-2,6-dicarboxylate N-succinyltransferase (EC 2.3.1.117)](https://rast.nmpdr.org/seedviewer.cgi?page=FunctionalRole&role=2,3,4,5-tetrahydropyridine-2,6-dicarboxylate%20N-succinyltransferase%20(EC%202.3.1.117)&subsystem_name=Lysine_Biosynthesis_DAP_Pathway) |
| Amino Acids and Derivatives | Lysine, threonine, methionine, and cysteine | [Lysine Biosynthesis DAP Pathway](https://rast.nmpdr.org/seedviewer.cgi?page=Subsystems&subsystem=Lysine_Biosynthesis_DAP_Pathway&organism=549.408) | [Diaminopimelate epimerase (EC 5.1.1.7)](https://rast.nmpdr.org/seedviewer.cgi?page=FunctionalRole&role=Diaminopimelate%20epimerase%20(EC%205.1.1.7)&subsystem_name=Lysine_Biosynthesis_DAP_Pathway) |
| Amino Acids and Derivatives | Lysine, threonine, methionine, and cysteine | [Lysine Biosynthesis DAP Pathway](https://rast.nmpdr.org/seedviewer.cgi?page=Subsystems&subsystem=Lysine_Biosynthesis_DAP_Pathway&organism=549.408) | [Aspartate-semialdehyde dehydrogenase (EC 1.2.1.11)](https://rast.nmpdr.org/seedviewer.cgi?page=FunctionalRole&role=Aspartate-semialdehyde%20dehydrogenase%20(EC%201.2.1.11)&subsystem_name=Lysine_Biosynthesis_DAP_Pathway) |
| Amino Acids and Derivatives | Lysine, threonine, methionine, and cysteine | [Lysine Biosynthesis DAP Pathway](https://rast.nmpdr.org/seedviewer.cgi?page=Subsystems&subsystem=Lysine_Biosynthesis_DAP_Pathway&organism=549.408) | [4-hydroxy-tetrahydrodipicolinate synthase (EC 4.3.3.7)](https://rast.nmpdr.org/seedviewer.cgi?page=FunctionalRole&role=4-hydroxy-tetrahydrodipicolinate%20synthase%20(EC%204.3.3.7)&subsystem_name=Lysine_Biosynthesis_DAP_Pathway) |
| Amino Acids and Derivatives | Lysine, threonine, methionine, and cysteine | [Lysine Biosynthesis DAP Pathway](https://rast.nmpdr.org/seedviewer.cgi?page=Subsystems&subsystem=Lysine_Biosynthesis_DAP_Pathway&organism=549.408) | [Diaminopimelate decarboxylase (EC 4.1.1.20)](https://rast.nmpdr.org/seedviewer.cgi?page=FunctionalRole&role=Diaminopimelate%20decarboxylase%20(EC%204.1.1.20)&subsystem_name=Lysine_Biosynthesis_DAP_Pathway) |
| Amino Acids and Derivatives | Lysine, threonine, methionine, and cysteine | [Lysine Biosynthesis DAP Pathway](https://rast.nmpdr.org/seedviewer.cgi?page=Subsystems&subsystem=Lysine_Biosynthesis_DAP_Pathway&organism=549.408) | [4-hydroxy-tetrahydrodipicolinate reductase (EC 1.17.1.8)](https://rast.nmpdr.org/seedviewer.cgi?page=FunctionalRole&role=4-hydroxy-tetrahydrodipicolinate%20reductase%20(EC%201.17.1.8)&subsystem_name=Lysine_Biosynthesis_DAP_Pathway) |
| Amino Acids and Derivatives | Lysine, threonine, methionine, and cysteine | [Lysine Biosynthesis DAP Pathway](https://rast.nmpdr.org/seedviewer.cgi?page=Subsystems&subsystem=Lysine_Biosynthesis_DAP_Pathway&organism=549.408) | [N-succinyl-L,L-diaminopimelate aminotransferase (EC 2.6.1.17)](https://rast.nmpdr.org/seedviewer.cgi?page=FunctionalRole&role=N-succinyl-L,L-diaminopimelate%20aminotransferase%20(EC%202.6.1.17)&subsystem_name=Lysine_Biosynthesis_DAP_Pathway) |
| Amino Acids and Derivatives | Lysine, threonine, methionine, and cysteine | [Lysine Biosynthesis DAP Pathway](https://rast.nmpdr.org/seedviewer.cgi?page=Subsystems&subsystem=Lysine_Biosynthesis_DAP_Pathway&organism=549.408) | [Aspartokinase (EC 2.7.2.4)](https://rast.nmpdr.org/seedviewer.cgi?page=FunctionalRole&role=Aspartokinase%20(EC%202.7.2.4)&subsystem_name=Lysine_Biosynthesis_DAP_Pathway) |
| Amino Acids and Derivatives | Lysine, threonine, methionine, and cysteine | [Lysine degradation](https://rast.nmpdr.org/seedviewer.cgi?page=Subsystems&subsystem=Lysine_degradation&organism=549.408) | [L-lysine permease](https://rast.nmpdr.org/seedviewer.cgi?page=FunctionalRole&role=L-lysine%20permease&subsystem_name=Lysine_degradation) |
| Amino Acids and Derivatives | Lysine, threonine, methionine, and cysteine | [Lysine degradation](https://rast.nmpdr.org/seedviewer.cgi?page=Subsystems&subsystem=Lysine_degradation&organism=549.408) | [Lysine 2,3-aminomutase (EC 5.4.3.2)](https://rast.nmpdr.org/seedviewer.cgi?page=FunctionalRole&role=Lysine%202,3-aminomutase%20(EC%205.4.3.2)&subsystem_name=Lysine_degradation) |
| Amino Acids and Derivatives | Lysine, threonine, methionine, and cysteine | [Lysine degradation](https://rast.nmpdr.org/seedviewer.cgi?page=Subsystems&subsystem=Lysine_degradation&organism=549.408) | [Lysine decarboxylase 2, constitutive (EC 4.1.1.18)](https://rast.nmpdr.org/seedviewer.cgi?page=FunctionalRole&role=Lysine%20decarboxylase%202,%20constitutive%20(EC%204.1.1.18)&subsystem_name=Lysine_degradation) |
| Amino Acids and Derivatives | Amino Acids and Derivatives - no subcategory | [Creatine and Creatinine Degradation](https://rast.nmpdr.org/seedviewer.cgi?page=Subsystems&subsystem=Creatine_and_Creatinine_Degradation&organism=549.408) | [N-methylhydantoinase B (EC 3.5.2.14)](https://rast.nmpdr.org/seedviewer.cgi?page=FunctionalRole&role=N-methylhydantoinase%20B%20(EC%203.5.2.14)&subsystem_name=Creatine_and_Creatinine_Degradation) |
| Amino Acids and Derivatives | Amino Acids and Derivatives - no subcategory | [Creatine and Creatinine Degradation](https://rast.nmpdr.org/seedviewer.cgi?page=Subsystems&subsystem=Creatine_and_Creatinine_Degradation&organism=549.408) | [Cytosine deaminase (EC 3.5.4.1)](https://rast.nmpdr.org/seedviewer.cgi?page=FunctionalRole&role=Cytosine%20deaminase%20(EC%203.5.4.1)&subsystem_name=Creatine_and_Creatinine_Degradation) |
| Amino Acids and Derivatives | Amino Acids and Derivatives - no subcategory | [Creatine and Creatinine Degradation](https://rast.nmpdr.org/seedviewer.cgi?page=Subsystems&subsystem=Creatine_and_Creatinine_Degradation&organism=549.408) | [N-methylhydantoinase A (EC 3.5.2.14)](https://rast.nmpdr.org/seedviewer.cgi?page=FunctionalRole&role=N-methylhydantoinase%20A%20(EC%203.5.2.14)&subsystem_name=Creatine_and_Creatinine_Degradation) |
| Amino Acids and Derivatives | Branched-chain amino acids | [Branched-Chain Amino Acid Biosynthesis](https://rast.nmpdr.org/seedviewer.cgi?page=Subsystems&subsystem=Branched-Chain_Amino_Acid_Biosynthesis&organism=549.408) | [3-isopropylmalate dehydratase small subunit (EC 4.2.1.33)](https://rast.nmpdr.org/seedviewer.cgi?page=FunctionalRole&role=3-isopropylmalate%20dehydratase%20small%20subunit%20(EC%204.2.1.33)&subsystem_name=Branched-Chain_Amino_Acid_Biosynthesis) |
| Amino Acids and Derivatives | Branched-chain amino acids | [Branched-Chain Amino Acid Biosynthesis](https://rast.nmpdr.org/seedviewer.cgi?page=Subsystems&subsystem=Branched-Chain_Amino_Acid_Biosynthesis&organism=549.408) | [2-isopropylmalate synthase (EC 2.3.3.13)](https://rast.nmpdr.org/seedviewer.cgi?page=FunctionalRole&role=2-isopropylmalate%20synthase%20(EC%202.3.3.13)&subsystem_name=Branched-Chain_Amino_Acid_Biosynthesis) |
| Amino Acids and Derivatives | Branched-chain amino acids | [Branched-Chain Amino Acid Biosynthesis](https://rast.nmpdr.org/seedviewer.cgi?page=Subsystems&subsystem=Branched-Chain_Amino_Acid_Biosynthesis&organism=549.408) | [Branched-chain amino acid aminotransferase (EC 2.6.1.42)](https://rast.nmpdr.org/seedviewer.cgi?page=FunctionalRole&role=Branched-chain%20amino%20acid%20aminotransferase%20(EC%202.6.1.42)&subsystem_name=Branched-Chain_Amino_Acid_Biosynthesis) |
| Amino Acids and Derivatives | Branched-chain amino acids | [Branched-Chain Amino Acid Biosynthesis](https://rast.nmpdr.org/seedviewer.cgi?page=Subsystems&subsystem=Branched-Chain_Amino_Acid_Biosynthesis&organism=549.408) | [3-isopropylmalate dehydrogenase (EC 1.1.1.85)](https://rast.nmpdr.org/seedviewer.cgi?page=FunctionalRole&role=3-isopropylmalate%20dehydrogenase%20(EC%201.1.1.85)&subsystem_name=Branched-Chain_Amino_Acid_Biosynthesis) |
| Amino Acids and Derivatives | Branched-chain amino acids | [Branched-Chain Amino Acid Biosynthesis](https://rast.nmpdr.org/seedviewer.cgi?page=Subsystems&subsystem=Branched-Chain_Amino_Acid_Biosynthesis&organism=549.408) | [Valine--pyruvate aminotransferase (EC 2.6.1.66)](https://rast.nmpdr.org/seedviewer.cgi?page=FunctionalRole&role=Valine--pyruvate%20aminotransferase%20(EC%202.6.1.66)&subsystem_name=Branched-Chain_Amino_Acid_Biosynthesis) |
| Amino Acids and Derivatives | Branched-chain amino acids | [Branched-Chain Amino Acid Biosynthesis](https://rast.nmpdr.org/seedviewer.cgi?page=Subsystems&subsystem=Branched-Chain_Amino_Acid_Biosynthesis&organism=549.408) | [Threonine dehydratase, catabolic (EC 4.3.1.19)](https://rast.nmpdr.org/seedviewer.cgi?page=FunctionalRole&role=Threonine%20dehydratase,%20catabolic%20(EC%204.3.1.19)&subsystem_name=Branched-Chain_Amino_Acid_Biosynthesis) |
| Amino Acids and Derivatives | Branched-chain amino acids | [Branched-Chain Amino Acid Biosynthesis](https://rast.nmpdr.org/seedviewer.cgi?page=Subsystems&subsystem=Branched-Chain_Amino_Acid_Biosynthesis&organism=549.408) | [3-isopropylmalate dehydratase large subunit (EC 4.2.1.33)](https://rast.nmpdr.org/seedviewer.cgi?page=FunctionalRole&role=3-isopropylmalate%20dehydratase%20large%20subunit%20(EC%204.2.1.33)&subsystem_name=Branched-Chain_Amino_Acid_Biosynthesis) |
| Amino Acids and Derivatives | Branched-chain amino acids | [Branched-Chain Amino Acid Biosynthesis](https://rast.nmpdr.org/seedviewer.cgi?page=Subsystems&subsystem=Branched-Chain_Amino_Acid_Biosynthesis&organism=549.408) | [Ketol-acid reductoisomerase (EC 1.1.1.86)](https://rast.nmpdr.org/seedviewer.cgi?page=FunctionalRole&role=Ketol-acid%20reductoisomerase%20(EC%201.1.1.86)&subsystem_name=Branched-Chain_Amino_Acid_Biosynthesis) |
| Amino Acids and Derivatives | Branched-chain amino acids | [Branched-Chain Amino Acid Biosynthesis](https://rast.nmpdr.org/seedviewer.cgi?page=Subsystems&subsystem=Branched-Chain_Amino_Acid_Biosynthesis&organism=549.408) | [Threonine dehydratase biosynthetic (EC 4.3.1.19)](https://rast.nmpdr.org/seedviewer.cgi?page=FunctionalRole&role=Threonine%20dehydratase%20biosynthetic%20(EC%204.3.1.19)&subsystem_name=Branched-Chain_Amino_Acid_Biosynthesis) |
| Amino Acids and Derivatives | Branched-chain amino acids | [Branched-Chain Amino Acid Biosynthesis](https://rast.nmpdr.org/seedviewer.cgi?page=Subsystems&subsystem=Branched-Chain_Amino_Acid_Biosynthesis&organism=549.408) | [Acetolactate synthase large subunit (EC 2.2.1.6)](https://rast.nmpdr.org/seedviewer.cgi?page=FunctionalRole&role=Acetolactate%20synthase%20large%20subunit%20(EC%202.2.1.6)&subsystem_name=Branched-Chain_Amino_Acid_Biosynthesis) |
| Amino Acids and Derivatives | Branched-chain amino acids | [Branched-Chain Amino Acid Biosynthesis](https://rast.nmpdr.org/seedviewer.cgi?page=Subsystems&subsystem=Branched-Chain_Amino_Acid_Biosynthesis&organism=549.408) | [Dihydroxy-acid dehydratase (EC 4.2.1.9)](https://rast.nmpdr.org/seedviewer.cgi?page=FunctionalRole&role=Dihydroxy-acid%20dehydratase%20(EC%204.2.1.9)&subsystem_name=Branched-Chain_Amino_Acid_Biosynthesis) |
| Amino Acids and Derivatives | Branched-chain amino acids | [Branched-Chain Amino Acid Biosynthesis](https://rast.nmpdr.org/seedviewer.cgi?page=Subsystems&subsystem=Branched-Chain_Amino_Acid_Biosynthesis&organism=549.408) | [Leucine-responsive regulatory protein, regulator for leucine (or lrp) regulon and high-affinity branched-chain amino acid transport system](https://rast.nmpdr.org/seedviewer.cgi?page=FunctionalRole&role=Leucine-responsive%20regulatory%20protein,%20regulator%20for%20leucine%20(or%20lrp)%20regulon%20and%20high-affinity%20branched-chain%20amino%20acid%20transport%20system&subsystem_name=Branched-Chain_Amino_Acid_Biosynthesis) |
| Amino Acids and Derivatives | Branched-chain amino acids | [Branched-Chain Amino Acid Biosynthesis](https://rast.nmpdr.org/seedviewer.cgi?page=Subsystems&subsystem=Branched-Chain_Amino_Acid_Biosynthesis&organism=549.408) | [Acetolactate synthase small subunit (EC 2.2.1.6)](https://rast.nmpdr.org/seedviewer.cgi?page=FunctionalRole&role=Acetolactate%20synthase%20small%20subunit%20(EC%202.2.1.6)&subsystem_name=Branched-Chain_Amino_Acid_Biosynthesis) |
| Amino Acids and Derivatives | Branched-chain amino acids | [Leucine Biosynthesis](https://rast.nmpdr.org/seedviewer.cgi?page=Subsystems&subsystem=Leucine_Biosynthesis&organism=549.408) | [3-isopropylmalate dehydratase small subunit (EC 4.2.1.33)](https://rast.nmpdr.org/seedviewer.cgi?page=FunctionalRole&role=3-isopropylmalate%20dehydratase%20small%20subunit%20(EC%204.2.1.33)&subsystem_name=Leucine_Biosynthesis) |
| Amino Acids and Derivatives | Branched-chain amino acids | [Leucine Biosynthesis](https://rast.nmpdr.org/seedviewer.cgi?page=Subsystems&subsystem=Leucine_Biosynthesis&organism=549.408) | [3-isopropylmalate dehydratase large subunit (EC 4.2.1.33)](https://rast.nmpdr.org/seedviewer.cgi?page=FunctionalRole&role=3-isopropylmalate%20dehydratase%20large%20subunit%20(EC%204.2.1.33)&subsystem_name=Leucine_Biosynthesis) |
| Amino Acids and Derivatives | Branched-chain amino acids | [Leucine Biosynthesis](https://rast.nmpdr.org/seedviewer.cgi?page=Subsystems&subsystem=Leucine_Biosynthesis&organism=549.408) | [2-isopropylmalate synthase (EC 2.3.3.13)](https://rast.nmpdr.org/seedviewer.cgi?page=FunctionalRole&role=2-isopropylmalate%20synthase%20(EC%202.3.3.13)&subsystem_name=Leucine_Biosynthesis) |
| Amino Acids and Derivatives | Branched-chain amino acids | [Leucine Biosynthesis](https://rast.nmpdr.org/seedviewer.cgi?page=Subsystems&subsystem=Leucine_Biosynthesis&organism=549.408) | [Branched-chain amino acid aminotransferase (EC 2.6.1.42)](https://rast.nmpdr.org/seedviewer.cgi?page=FunctionalRole&role=Branched-chain%20amino%20acid%20aminotransferase%20(EC%202.6.1.42)&subsystem_name=Leucine_Biosynthesis) |
| Amino Acids and Derivatives | Branched-chain amino acids | [Leucine Biosynthesis](https://rast.nmpdr.org/seedviewer.cgi?page=Subsystems&subsystem=Leucine_Biosynthesis&organism=549.408) | [3-isopropylmalate dehydrogenase (EC 1.1.1.85)](https://rast.nmpdr.org/seedviewer.cgi?page=FunctionalRole&role=3-isopropylmalate%20dehydrogenase%20(EC%201.1.1.85)&subsystem_name=Leucine_Biosynthesis) |
| Amino Acids and Derivatives | Polyamines | [DAP (1,3-diaminopropane) production](https://rast.nmpdr.org/seedviewer.cgi?page=Subsystems&subsystem=DAP_(1,3-diaminopropane)_production&organism=549.408) | [Diaminobutyrate--2-oxoglutarate aminotransferase (EC 2.6.1.76)](https://rast.nmpdr.org/seedviewer.cgi?page=FunctionalRole&role=Diaminobutyrate--2-oxoglutarate%20aminotransferase%20(EC%202.6.1.76)&subsystem_name=DAP_(1,3-diaminopropane)_production) |
| Amino Acids and Derivatives | Polyamines | [DAP (1,3-diaminopropane) production](https://rast.nmpdr.org/seedviewer.cgi?page=Subsystems&subsystem=DAP_(1,3-diaminopropane)_production&organism=549.408) | [Siderophore biosynthesis L-2,4-diaminobutyrate decarboxylase](https://rast.nmpdr.org/seedviewer.cgi?page=FunctionalRole&role=Siderophore%20biosynthesis%20L-2,4-diaminobutyrate%20decarboxylase&subsystem_name=DAP_(1,3-diaminopropane)_production) |
| Amino Acids and Derivatives | Polyamines | [DAP (1,3-diaminopropane) production](https://rast.nmpdr.org/seedviewer.cgi?page=Subsystems&subsystem=DAP_(1,3-diaminopropane)_production&organism=549.408) | [L-2,4-diaminobutyrate decarboxylase (EC 4.1.1.86)](https://rast.nmpdr.org/seedviewer.cgi?page=FunctionalRole&role=L-2,4-diaminobutyrate%20decarboxylase%20(EC%204.1.1.86)&subsystem_name=DAP_(1,3-diaminopropane)_production) |
| Amino Acids and Derivatives | Aromatic amino acids and derivatives | [Common Pathway For Synthesis of Aromatic Compounds (DAHP synthase to chorismate)](https://rast.nmpdr.org/seedviewer.cgi?page=Subsystems&subsystem=Common_Pathway_For_Synthesis_of_Aromatic_Compounds_(DAHP_synthase_to_chorismate)&organism=549.408) | [5-Enolpyruvylshikimate-3-phosphate synthase (EC 2.5.1.19)](https://rast.nmpdr.org/seedviewer.cgi?page=FunctionalRole&role=5-Enolpyruvylshikimate-3-phosphate%20synthase%20(EC%202.5.1.19)&subsystem_name=Common_Pathway_For_Synthesis_of_Aromatic_Compounds_(DAHP_synthase_to_chorismate)) |
| Amino Acids and Derivatives | Aromatic amino acids and derivatives | [Common Pathway For Synthesis of Aromatic Compounds (DAHP synthase to chorismate)](https://rast.nmpdr.org/seedviewer.cgi?page=Subsystems&subsystem=Common_Pathway_For_Synthesis_of_Aromatic_Compounds_(DAHP_synthase_to_chorismate)&organism=549.408) | [3-dehydroquinate dehydratase II (EC 4.2.1.10)](https://rast.nmpdr.org/seedviewer.cgi?page=FunctionalRole&role=3-dehydroquinate%20dehydratase%20II%20(EC%204.2.1.10)&subsystem_name=Common_Pathway_For_Synthesis_of_Aromatic_Compounds_(DAHP_synthase_to_chorismate)) |
| Amino Acids and Derivatives | Aromatic amino acids and derivatives | [Common Pathway For Synthesis of Aromatic Compounds (DAHP synthase to chorismate)](https://rast.nmpdr.org/seedviewer.cgi?page=Subsystems&subsystem=Common_Pathway_For_Synthesis_of_Aromatic_Compounds_(DAHP_synthase_to_chorismate)&organism=549.408) | [2-keto-3-deoxy-D-arabino-heptulosonate-7-phosphate synthase I alpha (EC 2.5.1.54)](https://rast.nmpdr.org/seedviewer.cgi?page=FunctionalRole&role=2-keto-3-deoxy-D-arabino-heptulosonate-7-phosphate%20synthase%20I%20alpha%20(EC%202.5.1.54)&subsystem_name=Common_Pathway_For_Synthesis_of_Aromatic_Compounds_(DAHP_synthase_to_chorismate)) |
| Amino Acids and Derivatives | Aromatic amino acids and derivatives | [Common Pathway For Synthesis of Aromatic Compounds (DAHP synthase to chorismate)](https://rast.nmpdr.org/seedviewer.cgi?page=Subsystems&subsystem=Common_Pathway_For_Synthesis_of_Aromatic_Compounds_(DAHP_synthase_to_chorismate)&organism=549.408) | [Transcriptional repressor protein TyrR](https://rast.nmpdr.org/seedviewer.cgi?page=FunctionalRole&role=Transcriptional%20repressor%20protein%20TyrR&subsystem_name=Common_Pathway_For_Synthesis_of_Aromatic_Compounds_(DAHP_synthase_to_chorismate)) |
| Amino Acids and Derivatives | Aromatic amino acids and derivatives | [Common Pathway For Synthesis of Aromatic Compounds (DAHP synthase to chorismate)](https://rast.nmpdr.org/seedviewer.cgi?page=Subsystems&subsystem=Common_Pathway_For_Synthesis_of_Aromatic_Compounds_(DAHP_synthase_to_chorismate)&organism=549.408) | [3-dehydroquinate synthase (EC 4.2.3.4)](https://rast.nmpdr.org/seedviewer.cgi?page=FunctionalRole&role=3-dehydroquinate%20synthase%20(EC%204.2.3.4)&subsystem_name=Common_Pathway_For_Synthesis_of_Aromatic_Compounds_(DAHP_synthase_to_chorismate)) |
| Amino Acids and Derivatives | Aromatic amino acids and derivatives | [Common Pathway For Synthesis of Aromatic Compounds (DAHP synthase to chorismate)](https://rast.nmpdr.org/seedviewer.cgi?page=Subsystems&subsystem=Common_Pathway_For_Synthesis_of_Aromatic_Compounds_(DAHP_synthase_to_chorismate)&organism=549.408) | [Chorismate synthase (EC 4.2.3.5)](https://rast.nmpdr.org/seedviewer.cgi?page=FunctionalRole&role=Chorismate%20synthase%20(EC%204.2.3.5)&subsystem_name=Common_Pathway_For_Synthesis_of_Aromatic_Compounds_(DAHP_synthase_to_chorismate)) |
| Amino Acids and Derivatives | Aromatic amino acids and derivatives | [Common Pathway For Synthesis of Aromatic Compounds (DAHP synthase to chorismate)](https://rast.nmpdr.org/seedviewer.cgi?page=Subsystems&subsystem=Common_Pathway_For_Synthesis_of_Aromatic_Compounds_(DAHP_synthase_to_chorismate)&organism=549.408) | [Shikimate kinase I (EC 2.7.1.71)](https://rast.nmpdr.org/seedviewer.cgi?page=FunctionalRole&role=Shikimate%20kinase%20I%20(EC%202.7.1.71)&subsystem_name=Common_Pathway_For_Synthesis_of_Aromatic_Compounds_(DAHP_synthase_to_chorismate)) |
| Amino Acids and Derivatives | Aromatic amino acids and derivatives | [Common Pathway For Synthesis of Aromatic Compounds (DAHP synthase to chorismate)](https://rast.nmpdr.org/seedviewer.cgi?page=Subsystems&subsystem=Common_Pathway_For_Synthesis_of_Aromatic_Compounds_(DAHP_synthase_to_chorismate)&organism=549.408) | [Shikimate 5-dehydrogenase I alpha (EC 1.1.1.25)](https://rast.nmpdr.org/seedviewer.cgi?page=FunctionalRole&role=Shikimate%205-dehydrogenase%20I%20alpha%20(EC%201.1.1.25)&subsystem_name=Common_Pathway_For_Synthesis_of_Aromatic_Compounds_(DAHP_synthase_to_chorismate)) |
| Amino Acids and Derivatives | Aromatic amino acids and derivatives | [Common Pathway For Synthesis of Aromatic Compounds (DAHP synthase to chorismate)](https://rast.nmpdr.org/seedviewer.cgi?page=Subsystems&subsystem=Common_Pathway_For_Synthesis_of_Aromatic_Compounds_(DAHP_synthase_to_chorismate)&organism=549.408) | [Transcriptional repressor protein TrpR](https://rast.nmpdr.org/seedviewer.cgi?page=FunctionalRole&role=Transcriptional%20repressor%20protein%20TrpR&subsystem_name=Common_Pathway_For_Synthesis_of_Aromatic_Compounds_(DAHP_synthase_to_chorismate)) |
| Amino Acids and Derivatives | Aromatic amino acids and derivatives | [Common Pathway For Synthesis of Aromatic Compounds (DAHP synthase to chorismate)](https://rast.nmpdr.org/seedviewer.cgi?page=Subsystems&subsystem=Common_Pathway_For_Synthesis_of_Aromatic_Compounds_(DAHP_synthase_to_chorismate)&organism=549.408) | [Shikimate kinase III (EC 2.7.1.71)](https://rast.nmpdr.org/seedviewer.cgi?page=FunctionalRole&role=Shikimate%20kinase%20III%20(EC%202.7.1.71)&subsystem_name=Common_Pathway_For_Synthesis_of_Aromatic_Compounds_(DAHP_synthase_to_chorismate)) |
| Amino Acids and Derivatives | Aromatic amino acids and derivatives | [Chorismate Synthesis](https://rast.nmpdr.org/seedviewer.cgi?page=Subsystems&subsystem=Chorismate_Synthesis&organism=549.408) | [5-Enolpyruvylshikimate-3-phosphate synthase (EC 2.5.1.19)](https://rast.nmpdr.org/seedviewer.cgi?page=FunctionalRole&role=5-Enolpyruvylshikimate-3-phosphate%20synthase%20(EC%202.5.1.19)&subsystem_name=Chorismate_Synthesis) |
| Amino Acids and Derivatives | Aromatic amino acids and derivatives | [Chorismate Synthesis](https://rast.nmpdr.org/seedviewer.cgi?page=Subsystems&subsystem=Chorismate_Synthesis&organism=549.408) | [Chorismate mutase I (EC 5.4.99.5)](https://rast.nmpdr.org/seedviewer.cgi?page=FunctionalRole&role=Chorismate%20mutase%20I%20(EC%205.4.99.5)&subsystem_name=Chorismate_Synthesis) |
| Amino Acids and Derivatives | Aromatic amino acids and derivatives | [Chorismate Synthesis](https://rast.nmpdr.org/seedviewer.cgi?page=Subsystems&subsystem=Chorismate_Synthesis&organism=549.408) | [3-dehydroquinate dehydratase II (EC 4.2.1.10)](https://rast.nmpdr.org/seedviewer.cgi?page=FunctionalRole&role=3-dehydroquinate%20dehydratase%20II%20(EC%204.2.1.10)&subsystem_name=Chorismate_Synthesis) |
| Amino Acids and Derivatives | Aromatic amino acids and derivatives | [Chorismate Synthesis](https://rast.nmpdr.org/seedviewer.cgi?page=Subsystems&subsystem=Chorismate_Synthesis&organism=549.408) | [2-keto-3-deoxy-D-arabino-heptulosonate-7-phosphate synthase I alpha (EC 2.5.1.54)](https://rast.nmpdr.org/seedviewer.cgi?page=FunctionalRole&role=2-keto-3-deoxy-D-arabino-heptulosonate-7-phosphate%20synthase%20I%20alpha%20(EC%202.5.1.54)&subsystem_name=Chorismate_Synthesis) |
| Amino Acids and Derivatives | Aromatic amino acids and derivatives | [Chorismate Synthesis](https://rast.nmpdr.org/seedviewer.cgi?page=Subsystems&subsystem=Chorismate_Synthesis&organism=549.408) | [Prephenate dehydratase (EC 4.2.1.51)](https://rast.nmpdr.org/seedviewer.cgi?page=FunctionalRole&role=Prephenate%20dehydratase%20(EC%204.2.1.51)&subsystem_name=Chorismate_Synthesis) |
| Amino Acids and Derivatives | Aromatic amino acids and derivatives | [Chorismate Synthesis](https://rast.nmpdr.org/seedviewer.cgi?page=Subsystems&subsystem=Chorismate_Synthesis&organism=549.408) | [3-dehydroquinate synthase (EC 4.2.3.4)](https://rast.nmpdr.org/seedviewer.cgi?page=FunctionalRole&role=3-dehydroquinate%20synthase%20(EC%204.2.3.4)&subsystem_name=Chorismate_Synthesis) |
| Amino Acids and Derivatives | Aromatic amino acids and derivatives | [Chorismate Synthesis](https://rast.nmpdr.org/seedviewer.cgi?page=Subsystems&subsystem=Chorismate_Synthesis&organism=549.408) | [Chorismate synthase (EC 4.2.3.5)](https://rast.nmpdr.org/seedviewer.cgi?page=FunctionalRole&role=Chorismate%20synthase%20(EC%204.2.3.5)&subsystem_name=Chorismate_Synthesis) |
| Amino Acids and Derivatives | Aromatic amino acids and derivatives | [Chorismate Synthesis](https://rast.nmpdr.org/seedviewer.cgi?page=Subsystems&subsystem=Chorismate_Synthesis&organism=549.408) | [Shikimate kinase I (EC 2.7.1.71)](https://rast.nmpdr.org/seedviewer.cgi?page=FunctionalRole&role=Shikimate%20kinase%20I%20(EC%202.7.1.71)&subsystem_name=Chorismate_Synthesis) |
| Amino Acids and Derivatives | Aromatic amino acids and derivatives | [Chorismate Synthesis](https://rast.nmpdr.org/seedviewer.cgi?page=Subsystems&subsystem=Chorismate_Synthesis&organism=549.408) | [Quinate/shikimate dehydrogenase [Pyrroloquinoline-quinone] (EC 1.1.99.25)](https://rast.nmpdr.org/seedviewer.cgi?page=FunctionalRole&role=Quinate/shikimate%20dehydrogenase%20%5bPyrroloquinoline-quinone%5d%20(EC%201.1.99.25)&subsystem_name=Chorismate_Synthesis) |
| Amino Acids and Derivatives | Aromatic amino acids and derivatives | [Chorismate Synthesis](https://rast.nmpdr.org/seedviewer.cgi?page=Subsystems&subsystem=Chorismate_Synthesis&organism=549.408) | [Shikimate 5-dehydrogenase I alpha (EC 1.1.1.25)](https://rast.nmpdr.org/seedviewer.cgi?page=FunctionalRole&role=Shikimate%205-dehydrogenase%20I%20alpha%20(EC%201.1.1.25)&subsystem_name=Chorismate_Synthesis) |
| Amino Acids and Derivatives | Aromatic amino acids and derivatives | [Chorismate Synthesis](https://rast.nmpdr.org/seedviewer.cgi?page=Subsystems&subsystem=Chorismate_Synthesis&organism=549.408) | [Periplasmic chorismate mutase I precursor (EC 5.4.99.5)](https://rast.nmpdr.org/seedviewer.cgi?page=FunctionalRole&role=Periplasmic%20chorismate%20mutase%20I%20precursor%20(EC%205.4.99.5)&subsystem_name=Chorismate_Synthesis) |
| Amino Acids and Derivatives | Aromatic amino acids and derivatives | [Chorismate Synthesis](https://rast.nmpdr.org/seedviewer.cgi?page=Subsystems&subsystem=Chorismate_Synthesis&organism=549.408) | [Shikimate kinase III (EC 2.7.1.71)](https://rast.nmpdr.org/seedviewer.cgi?page=FunctionalRole&role=Shikimate%20kinase%20III%20(EC%202.7.1.71)&subsystem_name=Chorismate_Synthesis) |
| Amino Acids and Derivatives | Aromatic amino acids and derivatives | [Chorismate Synthesis](https://rast.nmpdr.org/seedviewer.cgi?page=Subsystems&subsystem=Chorismate_Synthesis&organism=549.408) | [Cyclohexadienyl dehydrogenase (EC 1.3.1.12)(EC 1.3.1.43)](https://rast.nmpdr.org/seedviewer.cgi?page=FunctionalRole&role=Cyclohexadienyl%20dehydrogenase%20(EC%201.3.1.12)(EC%201.3.1.43)&subsystem_name=Chorismate_Synthesis) |
| Amino Acids and Derivatives | Aromatic amino acids and derivatives | [Chorismate: Intermediate for synthesis of Tryptophan, PAPA antibiotics, PABA, 3-hydroxyanthranilate and more.](https://rast.nmpdr.org/seedviewer.cgi?page=Subsystems&subsystem=Chorismate:_Intermediate_for_synthesis_of_Tryptophan,_PAPA_antibiotics,_PABA,_3-hydroxyanthranilate_and_more.&organism=549.408) | [Phosphoribosylformimino-5-aminoimidazole carboxamide ribotide isomerase (EC 5.3.1.16)](https://rast.nmpdr.org/seedviewer.cgi?page=FunctionalRole&role=Phosphoribosylformimino-5-aminoimidazole%20carboxamide%20ribotide%20isomerase%20(EC%205.3.1.16)&subsystem_name=Chorismate:_Intermediate_for_synthesis_of_Tryptophan,_PAPA_antibiotics,_PABA,_3-hydroxyanthranilate_and_more.) |
| Amino Acids and Derivatives | Aromatic amino acids and derivatives | [Chorismate: Intermediate for synthesis of Tryptophan, PAPA antibiotics, PABA, 3-hydroxyanthranilate and more.](https://rast.nmpdr.org/seedviewer.cgi?page=Subsystems&subsystem=Chorismate:_Intermediate_for_synthesis_of_Tryptophan,_PAPA_antibiotics,_PABA,_3-hydroxyanthranilate_and_more.&organism=549.408) | [Anthranilate synthase, amidotransferase component (EC 4.1.3.27)](https://rast.nmpdr.org/seedviewer.cgi?page=FunctionalRole&role=Anthranilate%20synthase,%20amidotransferase%20component%20(EC%204.1.3.27)&subsystem_name=Chorismate:_Intermediate_for_synthesis_of_Tryptophan,_PAPA_antibiotics,_PABA,_3-hydroxyanthranilate_and_more.) |
| Amino Acids and Derivatives | Aromatic amino acids and derivatives | [Chorismate: Intermediate for synthesis of Tryptophan, PAPA antibiotics, PABA, 3-hydroxyanthranilate and more.](https://rast.nmpdr.org/seedviewer.cgi?page=Subsystems&subsystem=Chorismate:_Intermediate_for_synthesis_of_Tryptophan,_PAPA_antibiotics,_PABA,_3-hydroxyanthranilate_and_more.&organism=549.408) | [Aminodeoxychorismate lyase (EC 4.1.3.38)](https://rast.nmpdr.org/seedviewer.cgi?page=FunctionalRole&role=Aminodeoxychorismate%20lyase%20(EC%204.1.3.38)&subsystem_name=Chorismate:_Intermediate_for_synthesis_of_Tryptophan,_PAPA_antibiotics,_PABA,_3-hydroxyanthranilate_and_more.) |
| Amino Acids and Derivatives | Aromatic amino acids and derivatives | [Chorismate: Intermediate for synthesis of Tryptophan, PAPA antibiotics, PABA, 3-hydroxyanthranilate and more.](https://rast.nmpdr.org/seedviewer.cgi?page=Subsystems&subsystem=Chorismate:_Intermediate_for_synthesis_of_Tryptophan,_PAPA_antibiotics,_PABA,_3-hydroxyanthranilate_and_more.&organism=549.408) | [Tryptophan synthase alpha chain (EC 4.2.1.20)](https://rast.nmpdr.org/seedviewer.cgi?page=FunctionalRole&role=Tryptophan%20synthase%20alpha%20chain%20(EC%204.2.1.20)&subsystem_name=Chorismate:_Intermediate_for_synthesis_of_Tryptophan,_PAPA_antibiotics,_PABA,_3-hydroxyanthranilate_and_more.) |
| Amino Acids and Derivatives | Aromatic amino acids and derivatives | [Chorismate: Intermediate for synthesis of Tryptophan, PAPA antibiotics, PABA, 3-hydroxyanthranilate and more.](https://rast.nmpdr.org/seedviewer.cgi?page=Subsystems&subsystem=Chorismate:_Intermediate_for_synthesis_of_Tryptophan,_PAPA_antibiotics,_PABA,_3-hydroxyanthranilate_and_more.&organism=549.408) | [Anthranilate phosphoribosyltransferase (EC 2.4.2.18)](https://rast.nmpdr.org/seedviewer.cgi?page=FunctionalRole&role=Anthranilate%20phosphoribosyltransferase%20(EC%202.4.2.18)&subsystem_name=Chorismate:_Intermediate_for_synthesis_of_Tryptophan,_PAPA_antibiotics,_PABA,_3-hydroxyanthranilate_and_more.) |
| Amino Acids and Derivatives | Aromatic amino acids and derivatives | [Chorismate: Intermediate for synthesis of Tryptophan, PAPA antibiotics, PABA, 3-hydroxyanthranilate and more.](https://rast.nmpdr.org/seedviewer.cgi?page=Subsystems&subsystem=Chorismate:_Intermediate_for_synthesis_of_Tryptophan,_PAPA_antibiotics,_PABA,_3-hydroxyanthranilate_and_more.&organism=549.408) | [Tryptophan synthase beta chain (EC 4.2.1.20)](https://rast.nmpdr.org/seedviewer.cgi?page=FunctionalRole&role=Tryptophan%20synthase%20beta%20chain%20(EC%204.2.1.20)&subsystem_name=Chorismate:_Intermediate_for_synthesis_of_Tryptophan,_PAPA_antibiotics,_PABA,_3-hydroxyanthranilate_and_more.) |
| Amino Acids and Derivatives | Aromatic amino acids and derivatives | [Chorismate: Intermediate for synthesis of Tryptophan, PAPA antibiotics, PABA, 3-hydroxyanthranilate and more.](https://rast.nmpdr.org/seedviewer.cgi?page=Subsystems&subsystem=Chorismate:_Intermediate_for_synthesis_of_Tryptophan,_PAPA_antibiotics,_PABA,_3-hydroxyanthranilate_and_more.&organism=549.408) | [Indole-3-glycerol phosphate synthase (EC 4.1.1.48)](https://rast.nmpdr.org/seedviewer.cgi?page=FunctionalRole&role=Indole-3-glycerol%20phosphate%20synthase%20(EC%204.1.1.48)&subsystem_name=Chorismate:_Intermediate_for_synthesis_of_Tryptophan,_PAPA_antibiotics,_PABA,_3-hydroxyanthranilate_and_more.) |
| Amino Acids and Derivatives | Aromatic amino acids and derivatives | [Chorismate: Intermediate for synthesis of Tryptophan, PAPA antibiotics, PABA, 3-hydroxyanthranilate and more.](https://rast.nmpdr.org/seedviewer.cgi?page=Subsystems&subsystem=Chorismate:_Intermediate_for_synthesis_of_Tryptophan,_PAPA_antibiotics,_PABA,_3-hydroxyanthranilate_and_more.&organism=549.408) | [Anthranilate synthase, aminase component (EC 4.1.3.27)](https://rast.nmpdr.org/seedviewer.cgi?page=FunctionalRole&role=Anthranilate%20synthase,%20aminase%20component%20(EC%204.1.3.27)&subsystem_name=Chorismate:_Intermediate_for_synthesis_of_Tryptophan,_PAPA_antibiotics,_PABA,_3-hydroxyanthranilate_and_more.) |
| Amino Acids and Derivatives | Aromatic amino acids and derivatives | [Chorismate: Intermediate for synthesis of Tryptophan, PAPA antibiotics, PABA, 3-hydroxyanthranilate and more.](https://rast.nmpdr.org/seedviewer.cgi?page=Subsystems&subsystem=Chorismate:_Intermediate_for_synthesis_of_Tryptophan,_PAPA_antibiotics,_PABA,_3-hydroxyanthranilate_and_more.&organism=549.408) | [Phosphoribosylanthranilate isomerase (EC 5.3.1.24)](https://rast.nmpdr.org/seedviewer.cgi?page=FunctionalRole&role=Phosphoribosylanthranilate%20isomerase%20(EC%205.3.1.24)&subsystem_name=Chorismate:_Intermediate_for_synthesis_of_Tryptophan,_PAPA_antibiotics,_PABA,_3-hydroxyanthranilate_and_more.) |
| Amino Acids and Derivatives | Aromatic amino acids and derivatives | [Chorismate: Intermediate for synthesis of Tryptophan, PAPA antibiotics, PABA, 3-hydroxyanthranilate and more.](https://rast.nmpdr.org/seedviewer.cgi?page=Subsystems&subsystem=Chorismate:_Intermediate_for_synthesis_of_Tryptophan,_PAPA_antibiotics,_PABA,_3-hydroxyanthranilate_and_more.&organism=549.408) | [Para-aminobenzoate synthase, aminase component (EC 2.6.1.85)](https://rast.nmpdr.org/seedviewer.cgi?page=FunctionalRole&role=Para-aminobenzoate%20synthase,%20aminase%20component%20(EC%202.6.1.85)&subsystem_name=Chorismate:_Intermediate_for_synthesis_of_Tryptophan,_PAPA_antibiotics,_PABA,_3-hydroxyanthranilate_and_more.) |
| Amino Acids and Derivatives | Aromatic amino acids and derivatives | [Chorismate: Intermediate for synthesis of Tryptophan, PAPA antibiotics, PABA, 3-hydroxyanthranilate and more.](https://rast.nmpdr.org/seedviewer.cgi?page=Subsystems&subsystem=Chorismate:_Intermediate_for_synthesis_of_Tryptophan,_PAPA_antibiotics,_PABA,_3-hydroxyanthranilate_and_more.&organism=549.408) | [Tryptophan synthase beta chain like (EC 4.2.1.20)](https://rast.nmpdr.org/seedviewer.cgi?page=FunctionalRole&role=Tryptophan%20synthase%20beta%20chain%20like%20(EC%204.2.1.20)&subsystem_name=Chorismate:_Intermediate_for_synthesis_of_Tryptophan,_PAPA_antibiotics,_PABA,_3-hydroxyanthranilate_and_more.) |
| Amino Acids and Derivatives | Aromatic amino acids and derivatives | [Chorismate: Intermediate for synthesis of Tryptophan, PAPA antibiotics, PABA, 3-hydroxyanthranilate and more.](https://rast.nmpdr.org/seedviewer.cgi?page=Subsystems&subsystem=Chorismate:_Intermediate_for_synthesis_of_Tryptophan,_PAPA_antibiotics,_PABA,_3-hydroxyanthranilate_and_more.&organism=549.408) | [Isochorismatase (EC 3.3.2.1)](https://rast.nmpdr.org/seedviewer.cgi?page=FunctionalRole&role=Isochorismatase%20(EC%203.3.2.1)&subsystem_name=Chorismate:_Intermediate_for_synthesis_of_Tryptophan,_PAPA_antibiotics,_PABA,_3-hydroxyanthranilate_and_more.) |
| Amino Acids and Derivatives | Aromatic amino acids and derivatives | [Chorismate: Intermediate for synthesis of Tryptophan, PAPA antibiotics, PABA, 3-hydroxyanthranilate and more.](https://rast.nmpdr.org/seedviewer.cgi?page=Subsystems&subsystem=Chorismate:_Intermediate_for_synthesis_of_Tryptophan,_PAPA_antibiotics,_PABA,_3-hydroxyanthranilate_and_more.&organism=549.408) | [Para-aminobenzoate synthase, amidotransferase component (EC 2.6.1.85)](https://rast.nmpdr.org/seedviewer.cgi?page=FunctionalRole&role=Para-aminobenzoate%20synthase,%20amidotransferase%20component%20(EC%202.6.1.85)&subsystem_name=Chorismate:_Intermediate_for_synthesis_of_Tryptophan,_PAPA_antibiotics,_PABA,_3-hydroxyanthranilate_and_more.) |
| Amino Acids and Derivatives | Aromatic amino acids and derivatives | [Phenylalanine and Tyrosine Branches from Chorismate](https://rast.nmpdr.org/seedviewer.cgi?page=Subsystems&subsystem=Phenylalanine_and_Tyrosine_Branches_from_Chorismate&organism=549.408) | [Periplasmic chorismate mutase I precursor (EC 5.4.99.5)](https://rast.nmpdr.org/seedviewer.cgi?page=FunctionalRole&role=Periplasmic%20chorismate%20mutase%20I%20precursor%20(EC%205.4.99.5)&subsystem_name=Phenylalanine_and_Tyrosine_Branches_from_Chorismate) |
| Amino Acids and Derivatives | Aromatic amino acids and derivatives | [Phenylalanine and Tyrosine Branches from Chorismate](https://rast.nmpdr.org/seedviewer.cgi?page=Subsystems&subsystem=Phenylalanine_and_Tyrosine_Branches_from_Chorismate&organism=549.408) | [Chorismate mutase I (EC 5.4.99.5)](https://rast.nmpdr.org/seedviewer.cgi?page=FunctionalRole&role=Chorismate%20mutase%20I%20(EC%205.4.99.5)&subsystem_name=Phenylalanine_and_Tyrosine_Branches_from_Chorismate) |
| Amino Acids and Derivatives | Aromatic amino acids and derivatives | [Phenylalanine and Tyrosine Branches from Chorismate](https://rast.nmpdr.org/seedviewer.cgi?page=Subsystems&subsystem=Phenylalanine_and_Tyrosine_Branches_from_Chorismate&organism=549.408) | [Cyclohexadienyl dehydratase (EC 4.2.1.51)(EC 4.2.1.91)](https://rast.nmpdr.org/seedviewer.cgi?page=FunctionalRole&role=Cyclohexadienyl%20dehydratase%20(EC%204.2.1.51)(EC%204.2.1.91)&subsystem_name=Phenylalanine_and_Tyrosine_Branches_from_Chorismate) |
| Amino Acids and Derivatives | Aromatic amino acids and derivatives | [Phenylalanine and Tyrosine Branches from Chorismate](https://rast.nmpdr.org/seedviewer.cgi?page=Subsystems&subsystem=Phenylalanine_and_Tyrosine_Branches_from_Chorismate&organism=549.408) | [Prephenate dehydratase (EC 4.2.1.51)](https://rast.nmpdr.org/seedviewer.cgi?page=FunctionalRole&role=Prephenate%20dehydratase%20(EC%204.2.1.51)&subsystem_name=Phenylalanine_and_Tyrosine_Branches_from_Chorismate) |
| Amino Acids and Derivatives | Aromatic amino acids and derivatives | [Phenylalanine and Tyrosine Branches from Chorismate](https://rast.nmpdr.org/seedviewer.cgi?page=Subsystems&subsystem=Phenylalanine_and_Tyrosine_Branches_from_Chorismate&organism=549.408) | [Transcriptional repressor protein TyrR](https://rast.nmpdr.org/seedviewer.cgi?page=FunctionalRole&role=Transcriptional%20repressor%20protein%20TyrR&subsystem_name=Phenylalanine_and_Tyrosine_Branches_from_Chorismate) |
| Amino Acids and Derivatives | Aromatic amino acids and derivatives | [Phenylalanine and Tyrosine Branches from Chorismate](https://rast.nmpdr.org/seedviewer.cgi?page=Subsystems&subsystem=Phenylalanine_and_Tyrosine_Branches_from_Chorismate&organism=549.408) | [Biosynthetic Aromatic amino acid aminotransferase alpha (EC 2.6.1.57)](https://rast.nmpdr.org/seedviewer.cgi?page=FunctionalRole&role=Biosynthetic%20Aromatic%20amino%20acid%20aminotransferase%20alpha%20(EC%202.6.1.57)&subsystem_name=Phenylalanine_and_Tyrosine_Branches_from_Chorismate) |
| Amino Acids and Derivatives | Aromatic amino acids and derivatives | [Phenylalanine and Tyrosine Branches from Chorismate](https://rast.nmpdr.org/seedviewer.cgi?page=Subsystems&subsystem=Phenylalanine_and_Tyrosine_Branches_from_Chorismate&organism=549.408) | [Cyclohexadienyl dehydrogenase (EC 1.3.1.12)(EC 1.3.1.43)](https://rast.nmpdr.org/seedviewer.cgi?page=FunctionalRole&role=Cyclohexadienyl%20dehydrogenase%20(EC%201.3.1.12)(EC%201.3.1.43)&subsystem_name=Phenylalanine_and_Tyrosine_Branches_from_Chorismate) |
| Amino Acids and Derivatives | Aromatic amino acids and derivatives | [Tryptophan synthesis](https://rast.nmpdr.org/seedviewer.cgi?page=Subsystems&subsystem=Tryptophan_synthesis&organism=549.408) | [Anthranilate synthase, amidotransferase component (EC 4.1.3.27)](https://rast.nmpdr.org/seedviewer.cgi?page=FunctionalRole&role=Anthranilate%20synthase,%20amidotransferase%20component%20(EC%204.1.3.27)&subsystem_name=Tryptophan_synthesis) |
| Amino Acids and Derivatives | Aromatic amino acids and derivatives | [Tryptophan synthesis](https://rast.nmpdr.org/seedviewer.cgi?page=Subsystems&subsystem=Tryptophan_synthesis&organism=549.408) | [Aminodeoxychorismate lyase (EC 4.1.3.38)](https://rast.nmpdr.org/seedviewer.cgi?page=FunctionalRole&role=Aminodeoxychorismate%20lyase%20(EC%204.1.3.38)&subsystem_name=Tryptophan_synthesis) |
| Amino Acids and Derivatives | Aromatic amino acids and derivatives | [Tryptophan synthesis](https://rast.nmpdr.org/seedviewer.cgi?page=Subsystems&subsystem=Tryptophan_synthesis&organism=549.408) | [Tryptophan synthase alpha chain (EC 4.2.1.20)](https://rast.nmpdr.org/seedviewer.cgi?page=FunctionalRole&role=Tryptophan%20synthase%20alpha%20chain%20(EC%204.2.1.20)&subsystem_name=Tryptophan_synthesis) |
| Amino Acids and Derivatives | Aromatic amino acids and derivatives | [Tryptophan synthesis](https://rast.nmpdr.org/seedviewer.cgi?page=Subsystems&subsystem=Tryptophan_synthesis&organism=549.408) | [Anthranilate phosphoribosyltransferase (EC 2.4.2.18)](https://rast.nmpdr.org/seedviewer.cgi?page=FunctionalRole&role=Anthranilate%20phosphoribosyltransferase%20(EC%202.4.2.18)&subsystem_name=Tryptophan_synthesis) |
| Amino Acids and Derivatives | Aromatic amino acids and derivatives | [Tryptophan synthesis](https://rast.nmpdr.org/seedviewer.cgi?page=Subsystems&subsystem=Tryptophan_synthesis&organism=549.408) | [Tryptophan synthase beta chain (EC 4.2.1.20)](https://rast.nmpdr.org/seedviewer.cgi?page=FunctionalRole&role=Tryptophan%20synthase%20beta%20chain%20(EC%204.2.1.20)&subsystem_name=Tryptophan_synthesis) |
| Amino Acids and Derivatives | Aromatic amino acids and derivatives | [Tryptophan synthesis](https://rast.nmpdr.org/seedviewer.cgi?page=Subsystems&subsystem=Tryptophan_synthesis&organism=549.408) | [Indole-3-glycerol phosphate synthase (EC 4.1.1.48)](https://rast.nmpdr.org/seedviewer.cgi?page=FunctionalRole&role=Indole-3-glycerol%20phosphate%20synthase%20(EC%204.1.1.48)&subsystem_name=Tryptophan_synthesis) |
| Amino Acids and Derivatives | Aromatic amino acids and derivatives | [Tryptophan synthesis](https://rast.nmpdr.org/seedviewer.cgi?page=Subsystems&subsystem=Tryptophan_synthesis&organism=549.408) | [Anthranilate synthase, aminase component (EC 4.1.3.27)](https://rast.nmpdr.org/seedviewer.cgi?page=FunctionalRole&role=Anthranilate%20synthase,%20aminase%20component%20(EC%204.1.3.27)&subsystem_name=Tryptophan_synthesis) |
| Amino Acids and Derivatives | Aromatic amino acids and derivatives | [Tryptophan synthesis](https://rast.nmpdr.org/seedviewer.cgi?page=Subsystems&subsystem=Tryptophan_synthesis&organism=549.408) | [Phosphoribosylanthranilate isomerase (EC 5.3.1.24)](https://rast.nmpdr.org/seedviewer.cgi?page=FunctionalRole&role=Phosphoribosylanthranilate%20isomerase%20(EC%205.3.1.24)&subsystem_name=Tryptophan_synthesis) |
| Amino Acids and Derivatives | Aromatic amino acids and derivatives | [Tryptophan synthesis](https://rast.nmpdr.org/seedviewer.cgi?page=Subsystems&subsystem=Tryptophan_synthesis&organism=549.408) | [Para-aminobenzoate synthase, aminase component (EC 2.6.1.85)](https://rast.nmpdr.org/seedviewer.cgi?page=FunctionalRole&role=Para-aminobenzoate%20synthase,%20aminase%20component%20(EC%202.6.1.85)&subsystem_name=Tryptophan_synthesis) |
| Amino Acids and Derivatives | Aromatic amino acids and derivatives | [Tryptophan synthesis](https://rast.nmpdr.org/seedviewer.cgi?page=Subsystems&subsystem=Tryptophan_synthesis&organism=549.408) | [Transcriptional repressor protein TrpR](https://rast.nmpdr.org/seedviewer.cgi?page=FunctionalRole&role=Transcriptional%20repressor%20protein%20TrpR&subsystem_name=Tryptophan_synthesis) |
| Amino Acids and Derivatives | Aromatic amino acids and derivatives | [Tryptophan synthesis](https://rast.nmpdr.org/seedviewer.cgi?page=Subsystems&subsystem=Tryptophan_synthesis&organism=549.408) | [Para-aminobenzoate synthase, amidotransferase component (EC 2.6.1.85)](https://rast.nmpdr.org/seedviewer.cgi?page=FunctionalRole&role=Para-aminobenzoate%20synthase,%20amidotransferase%20component%20(EC%202.6.1.85)&subsystem_name=Tryptophan_synthesis) |
| Amino Acids and Derivatives | Proline and 4-hydroxyproline | [Proline Synthesis](https://rast.nmpdr.org/seedviewer.cgi?page=Subsystems&subsystem=Proline_Synthesis&organism=549.408) | [Pyrroline-5-carboxylate reductase (EC 1.5.1.2)](https://rast.nmpdr.org/seedviewer.cgi?page=FunctionalRole&role=Pyrroline-5-carboxylate%20reductase%20(EC%201.5.1.2)&subsystem_name=Proline_Synthesis) |
| Amino Acids and Derivatives | Proline and 4-hydroxyproline | [Proline Synthesis](https://rast.nmpdr.org/seedviewer.cgi?page=Subsystems&subsystem=Proline_Synthesis&organism=549.408) | [Gamma-glutamyl phosphate reductase (EC 1.2.1.41)](https://rast.nmpdr.org/seedviewer.cgi?page=FunctionalRole&role=Gamma-glutamyl%20phosphate%20reductase%20(EC%201.2.1.41)&subsystem_name=Proline_Synthesis) |
| Amino Acids and Derivatives | Proline and 4-hydroxyproline | [Proline Synthesis](https://rast.nmpdr.org/seedviewer.cgi?page=Subsystems&subsystem=Proline_Synthesis&organism=549.408) | [NADP-specific glutamate dehydrogenase (EC 1.4.1.4)](https://rast.nmpdr.org/seedviewer.cgi?page=FunctionalRole&role=NADP-specific%20glutamate%20dehydrogenase%20(EC%201.4.1.4)&subsystem_name=Proline_Synthesis) |
| Amino Acids and Derivatives | Proline and 4-hydroxyproline | [Proline Synthesis](https://rast.nmpdr.org/seedviewer.cgi?page=Subsystems&subsystem=Proline_Synthesis&organism=549.408) | [Glutamate 5-kinase (EC 2.7.2.11)](https://rast.nmpdr.org/seedviewer.cgi?page=FunctionalRole&role=Glutamate%205-kinase%20(EC%202.7.2.11)&subsystem_name=Proline_Synthesis) |
| Amino Acids and Derivatives | Proline and 4-hydroxyproline | [A Hypothetical Protein Related to Proline Metabolism](https://rast.nmpdr.org/seedviewer.cgi?page=Subsystems&subsystem=A_Hypothetical_Protein_Related_to_Proline_Metabolism&organism=549.408) | [Hypothetical protein YggS, proline synthase co-transcribed bacterial homolog PROSC](https://rast.nmpdr.org/seedviewer.cgi?page=FunctionalRole&role=Hypothetical%20protein%20YggS,%20proline%20synthase%20co-transcribed%20bacterial%20homolog%20PROSC&subsystem_name=A_Hypothetical_Protein_Related_to_Proline_Metabolism) |
| Amino Acids and Derivatives | Proline and 4-hydroxyproline | [A Hypothetical Protein Related to Proline Metabolism](https://rast.nmpdr.org/seedviewer.cgi?page=Subsystems&subsystem=A_Hypothetical_Protein_Related_to_Proline_Metabolism&organism=549.408) | [Pyrroline-5-carboxylate reductase (EC 1.5.1.2)](https://rast.nmpdr.org/seedviewer.cgi?page=FunctionalRole&role=Pyrroline-5-carboxylate%20reductase%20(EC%201.5.1.2)&subsystem_name=A_Hypothetical_Protein_Related_to_Proline_Metabolism) |
| Amino Acids and Derivatives | Proline and 4-hydroxyproline | [Proline, 4-hydroxyproline uptake and utilization](https://rast.nmpdr.org/seedviewer.cgi?page=Subsystems&subsystem=Proline,_4-hydroxyproline_uptake_and_utilization&organism=549.408) | [Proline/sodium symporter PutP (TC 2.A.21.2.1)](https://rast.nmpdr.org/seedviewer.cgi?page=FunctionalRole&role=Proline/sodium%20symporter%20PutP%20(TC%202.A.21.2.1)&subsystem_name=Proline,_4-hydroxyproline_uptake_and_utilization) |
| Amino Acids and Derivatives | Proline and 4-hydroxyproline | [Proline, 4-hydroxyproline uptake and utilization](https://rast.nmpdr.org/seedviewer.cgi?page=Subsystems&subsystem=Proline,_4-hydroxyproline_uptake_and_utilization&organism=549.408) | [Proline dehydrogenase (EC 1.5.99.8) (Proline oxidase)](https://rast.nmpdr.org/seedviewer.cgi?page=FunctionalRole&role=Proline%20dehydrogenase%20(EC%201.5.99.8)%20(Proline%20oxidase)&subsystem_name=Proline,_4-hydroxyproline_uptake_and_utilization) |
| Amino Acids and Derivatives | Proline and 4-hydroxyproline | [Proline, 4-hydroxyproline uptake and utilization](https://rast.nmpdr.org/seedviewer.cgi?page=Subsystems&subsystem=Proline,_4-hydroxyproline_uptake_and_utilization&organism=549.408) | [ProQ: influences osmotic activation of compatible solute ProP](https://rast.nmpdr.org/seedviewer.cgi?page=FunctionalRole&role=ProQ:%20influences%20osmotic%20activation%20of%20compatible%20solute%20ProP&subsystem_name=Proline,_4-hydroxyproline_uptake_and_utilization) |
| Amino Acids and Derivatives | Proline and 4-hydroxyproline | [Proline, 4-hydroxyproline uptake and utilization](https://rast.nmpdr.org/seedviewer.cgi?page=Subsystems&subsystem=Proline,_4-hydroxyproline_uptake_and_utilization&organism=549.408) | [L-Proline/Glycine betaine transporter ProP](https://rast.nmpdr.org/seedviewer.cgi?page=FunctionalRole&role=L-Proline/Glycine%20betaine%20transporter%20ProP&subsystem_name=Proline,_4-hydroxyproline_uptake_and_utilization) |
| Amino Acids and Derivatives | Proline and 4-hydroxyproline | [Proline, 4-hydroxyproline uptake and utilization](https://rast.nmpdr.org/seedviewer.cgi?page=Subsystems&subsystem=Proline,_4-hydroxyproline_uptake_and_utilization&organism=549.408) | [Transcriptional repressor of PutA and PutP](https://rast.nmpdr.org/seedviewer.cgi?page=FunctionalRole&role=Transcriptional%20repressor%20of%20PutA%20and%20PutP&subsystem_name=Proline,_4-hydroxyproline_uptake_and_utilization) |
| Amino Acids and Derivatives | Proline and 4-hydroxyproline | [Proline, 4-hydroxyproline uptake and utilization](https://rast.nmpdr.org/seedviewer.cgi?page=Subsystems&subsystem=Proline,_4-hydroxyproline_uptake_and_utilization&organism=549.408) | [Proline-specific permease proY](https://rast.nmpdr.org/seedviewer.cgi?page=FunctionalRole&role=Proline-specific%20permease%20proY&subsystem_name=Proline,_4-hydroxyproline_uptake_and_utilization) |
| Amino Acids and Derivatives | Alanine, serine, and glycine | [Glycine Biosynthesis](https://rast.nmpdr.org/seedviewer.cgi?page=Subsystems&subsystem=Glycine_Biosynthesis&organism=549.408) | [2-amino-3-ketobutyrate coenzyme A ligase (EC 2.3.1.29)](https://rast.nmpdr.org/seedviewer.cgi?page=FunctionalRole&role=2-amino-3-ketobutyrate%20coenzyme%20A%20ligase%20(EC%202.3.1.29)&subsystem_name=Glycine_Biosynthesis) |
| Amino Acids and Derivatives | Alanine, serine, and glycine | [Glycine Biosynthesis](https://rast.nmpdr.org/seedviewer.cgi?page=Subsystems&subsystem=Glycine_Biosynthesis&organism=549.408) | [Serine hydroxymethyltransferase (EC 2.1.2.1)](https://rast.nmpdr.org/seedviewer.cgi?page=FunctionalRole&role=Serine%20hydroxymethyltransferase%20(EC%202.1.2.1)&subsystem_name=Glycine_Biosynthesis) |
| Amino Acids and Derivatives | Alanine, serine, and glycine | [Glycine Biosynthesis](https://rast.nmpdr.org/seedviewer.cgi?page=Subsystems&subsystem=Glycine_Biosynthesis&organism=549.408) | [L-threonine 3-dehydrogenase (EC 1.1.1.103)](https://rast.nmpdr.org/seedviewer.cgi?page=FunctionalRole&role=L-threonine%203-dehydrogenase%20(EC%201.1.1.103)&subsystem_name=Glycine_Biosynthesis) |
| Amino Acids and Derivatives | Alanine, serine, and glycine | [Alanine biosynthesis](https://rast.nmpdr.org/seedviewer.cgi?page=Subsystems&subsystem=Alanine_biosynthesis&organism=549.408) | [Branched-chain amino acid aminotransferase (EC 2.6.1.42)](https://rast.nmpdr.org/seedviewer.cgi?page=FunctionalRole&role=Branched-chain%20amino%20acid%20aminotransferase%20(EC%202.6.1.42)&subsystem_name=Alanine_biosynthesis) |
| Amino Acids and Derivatives | Alanine, serine, and glycine | [Alanine biosynthesis](https://rast.nmpdr.org/seedviewer.cgi?page=Subsystems&subsystem=Alanine_biosynthesis&organism=549.408) | [Valine--pyruvate aminotransferase (EC 2.6.1.66)](https://rast.nmpdr.org/seedviewer.cgi?page=FunctionalRole&role=Valine--pyruvate%20aminotransferase%20(EC%202.6.1.66)&subsystem_name=Alanine_biosynthesis) |
| Amino Acids and Derivatives | Alanine, serine, and glycine | [Alanine biosynthesis](https://rast.nmpdr.org/seedviewer.cgi?page=Subsystems&subsystem=Alanine_biosynthesis&organism=549.408) | [Iron-sulfur cluster regulator IscR](https://rast.nmpdr.org/seedviewer.cgi?page=FunctionalRole&role=Iron-sulfur%20cluster%20regulator%20IscR&subsystem_name=Alanine_biosynthesis) |
| Amino Acids and Derivatives | Alanine, serine, and glycine | [Alanine biosynthesis](https://rast.nmpdr.org/seedviewer.cgi?page=Subsystems&subsystem=Alanine_biosynthesis&organism=549.408) | [Cysteine desulfurase CsdA-CsdE (EC 2.8.1.7), main protein CsdA](https://rast.nmpdr.org/seedviewer.cgi?page=FunctionalRole&role=Cysteine%20desulfurase%20CsdA-CsdE%20(EC%202.8.1.7),%20main%20protein%20CsdA&subsystem_name=Alanine_biosynthesis) |
| Amino Acids and Derivatives | Alanine, serine, and glycine | [Alanine biosynthesis](https://rast.nmpdr.org/seedviewer.cgi?page=Subsystems&subsystem=Alanine_biosynthesis&organism=549.408) | [Alanine racemase, catabolic (EC 5.1.1.1)](https://rast.nmpdr.org/seedviewer.cgi?page=FunctionalRole&role=Alanine%20racemase,%20catabolic%20(EC%205.1.1.1)&subsystem_name=Alanine_biosynthesis) |
| Amino Acids and Derivatives | Alanine, serine, and glycine | [Alanine biosynthesis](https://rast.nmpdr.org/seedviewer.cgi?page=Subsystems&subsystem=Alanine_biosynthesis&organism=549.408) | [HTH-type transcriptional regulator IlvY](https://rast.nmpdr.org/seedviewer.cgi?page=FunctionalRole&role=HTH-type%20transcriptional%20regulator%20IlvY&subsystem_name=Alanine_biosynthesis) |
| Amino Acids and Derivatives | Alanine, serine, and glycine | [Alanine biosynthesis](https://rast.nmpdr.org/seedviewer.cgi?page=Subsystems&subsystem=Alanine_biosynthesis&organism=549.408) | [Cysteine desulfurase (EC 2.8.1.7), IscS subfamily](https://rast.nmpdr.org/seedviewer.cgi?page=FunctionalRole&role=Cysteine%20desulfurase%20(EC%202.8.1.7),%20IscS%20subfamily&subsystem_name=Alanine_biosynthesis) |
| Amino Acids and Derivatives | Alanine, serine, and glycine | [Alanine biosynthesis](https://rast.nmpdr.org/seedviewer.cgi?page=Subsystems&subsystem=Alanine_biosynthesis&organism=549.408) | [Cysteine desulfurase (EC 2.8.1.7), SufS subfamily](https://rast.nmpdr.org/seedviewer.cgi?page=FunctionalRole&role=Cysteine%20desulfurase%20(EC%202.8.1.7),%20SufS%20subfamily&subsystem_name=Alanine_biosynthesis) |
| Amino Acids and Derivatives | Alanine, serine, and glycine | [Serine Biosynthesis](https://rast.nmpdr.org/seedviewer.cgi?page=Subsystems&subsystem=Serine_Biosynthesis&organism=549.408) | [D-3-phosphoglycerate dehydrogenase (EC 1.1.1.95)](https://rast.nmpdr.org/seedviewer.cgi?page=FunctionalRole&role=D-3-phosphoglycerate%20dehydrogenase%20(EC%201.1.1.95)&subsystem_name=Serine_Biosynthesis) |
| Amino Acids and Derivatives | Alanine, serine, and glycine | [Serine Biosynthesis](https://rast.nmpdr.org/seedviewer.cgi?page=Subsystems&subsystem=Serine_Biosynthesis&organism=549.408) | [Phosphoserine phosphatase (EC 3.1.3.3)](https://rast.nmpdr.org/seedviewer.cgi?page=FunctionalRole&role=Phosphoserine%20phosphatase%20(EC%203.1.3.3)&subsystem_name=Serine_Biosynthesis) |
| Amino Acids and Derivatives | Alanine, serine, and glycine | [Serine Biosynthesis](https://rast.nmpdr.org/seedviewer.cgi?page=Subsystems&subsystem=Serine_Biosynthesis&organism=549.408) | [Serine hydroxymethyltransferase (EC 2.1.2.1)](https://rast.nmpdr.org/seedviewer.cgi?page=FunctionalRole&role=Serine%20hydroxymethyltransferase%20(EC%202.1.2.1)&subsystem_name=Serine_Biosynthesis) |
| Amino Acids and Derivatives | Alanine, serine, and glycine | [Serine Biosynthesis](https://rast.nmpdr.org/seedviewer.cgi?page=Subsystems&subsystem=Serine_Biosynthesis&organism=549.408) | [Phosphoserine aminotransferase (EC 2.6.1.52)](https://rast.nmpdr.org/seedviewer.cgi?page=FunctionalRole&role=Phosphoserine%20aminotransferase%20(EC%202.6.1.52)&subsystem_name=Serine_Biosynthesis) |
| Amino Acids and Derivatives | Alanine, serine, and glycine | [Glycine cleavage system](https://rast.nmpdr.org/seedviewer.cgi?page=Subsystems&subsystem=Glycine_cleavage_system&organism=549.408) | [Glycine cleavage system H protein](https://rast.nmpdr.org/seedviewer.cgi?page=FunctionalRole&role=Glycine%20cleavage%20system%20H%20protein&subsystem_name=Glycine_cleavage_system) |
| Amino Acids and Derivatives | Alanine, serine, and glycine | [Glycine cleavage system](https://rast.nmpdr.org/seedviewer.cgi?page=Subsystems&subsystem=Glycine_cleavage_system&organism=549.408) | [Glycine dehydrogenase [decarboxylating] (glycine cleavage system P protein) (EC 1.4.4.2)](https://rast.nmpdr.org/seedviewer.cgi?page=FunctionalRole&role=Glycine%20dehydrogenase%20%5bdecarboxylating%5d%20(glycine%20cleavage%20system%20P%20protein)%20(EC%201.4.4.2)&subsystem_name=Glycine_cleavage_system) |
| Amino Acids and Derivatives | Alanine, serine, and glycine | [Glycine cleavage system](https://rast.nmpdr.org/seedviewer.cgi?page=Subsystems&subsystem=Glycine_cleavage_system&organism=549.408) | [Glycine cleavage system transcriptional activator GcvA](https://rast.nmpdr.org/seedviewer.cgi?page=FunctionalRole&role=Glycine%20cleavage%20system%20transcriptional%20activator%20GcvA&subsystem_name=Glycine_cleavage_system) |
| Amino Acids and Derivatives | Alanine, serine, and glycine | [Glycine cleavage system](https://rast.nmpdr.org/seedviewer.cgi?page=Subsystems&subsystem=Glycine_cleavage_system&organism=549.408) | [Glycine cleavage system transcriptional antiactivator GcvR](https://rast.nmpdr.org/seedviewer.cgi?page=FunctionalRole&role=Glycine%20cleavage%20system%20transcriptional%20antiactivator%20GcvR&subsystem_name=Glycine_cleavage_system) |
| Amino Acids and Derivatives | Alanine, serine, and glycine | [Glycine cleavage system](https://rast.nmpdr.org/seedviewer.cgi?page=Subsystems&subsystem=Glycine_cleavage_system&organism=549.408) | [Dihydrolipoamide dehydrogenase (EC 1.8.1.4)](https://rast.nmpdr.org/seedviewer.cgi?page=FunctionalRole&role=Dihydrolipoamide%20dehydrogenase%20(EC%201.8.1.4)&subsystem_name=Glycine_cleavage_system) |
| Amino Acids and Derivatives | Alanine, serine, and glycine | [Glycine cleavage system](https://rast.nmpdr.org/seedviewer.cgi?page=Subsystems&subsystem=Glycine_cleavage_system&organism=549.408) | [Aminomethyltransferase (glycine cleavage system T protein) (EC 2.1.2.10)](https://rast.nmpdr.org/seedviewer.cgi?page=FunctionalRole&role=Aminomethyltransferase%20(glycine%20cleavage%20system%20T%20protein)%20(EC%202.1.2.10)&subsystem_name=Glycine_cleavage_system) |
| Amino Acids and Derivatives | Alanine, serine, and glycine | [Glycine and Serine Utilization](https://rast.nmpdr.org/seedviewer.cgi?page=Subsystems&subsystem=Glycine_and_Serine_Utilization&organism=549.408) | [D-serine/D-alanine/glycine transporter](https://rast.nmpdr.org/seedviewer.cgi?page=FunctionalRole&role=D-serine/D-alanine/glycine%20transporter&subsystem_name=Glycine_and_Serine_Utilization) |
| Amino Acids and Derivatives | Alanine, serine, and glycine | [Glycine and Serine Utilization](https://rast.nmpdr.org/seedviewer.cgi?page=Subsystems&subsystem=Glycine_and_Serine_Utilization&organism=549.408) | [D-3-phosphoglycerate dehydrogenase (EC 1.1.1.95)](https://rast.nmpdr.org/seedviewer.cgi?page=FunctionalRole&role=D-3-phosphoglycerate%20dehydrogenase%20(EC%201.1.1.95)&subsystem_name=Glycine_and_Serine_Utilization) |
| Amino Acids and Derivatives | Alanine, serine, and glycine | [Glycine and Serine Utilization](https://rast.nmpdr.org/seedviewer.cgi?page=Subsystems&subsystem=Glycine_and_Serine_Utilization&organism=549.408) | [Phosphoserine phosphatase (EC 3.1.3.3)](https://rast.nmpdr.org/seedviewer.cgi?page=FunctionalRole&role=Phosphoserine%20phosphatase%20(EC%203.1.3.3)&subsystem_name=Glycine_and_Serine_Utilization) |
| Amino Acids and Derivatives | Alanine, serine, and glycine | [Glycine and Serine Utilization](https://rast.nmpdr.org/seedviewer.cgi?page=Subsystems&subsystem=Glycine_and_Serine_Utilization&organism=549.408) | [Serine hydroxymethyltransferase (EC 2.1.2.1)](https://rast.nmpdr.org/seedviewer.cgi?page=FunctionalRole&role=Serine%20hydroxymethyltransferase%20(EC%202.1.2.1)&subsystem_name=Glycine_and_Serine_Utilization) |
| Amino Acids and Derivatives | Alanine, serine, and glycine | [Glycine and Serine Utilization](https://rast.nmpdr.org/seedviewer.cgi?page=Subsystems&subsystem=Glycine_and_Serine_Utilization&organism=549.408) | [Threonine dehydratase, catabolic (EC 4.3.1.19)](https://rast.nmpdr.org/seedviewer.cgi?page=FunctionalRole&role=Threonine%20dehydratase,%20catabolic%20(EC%204.3.1.19)&subsystem_name=Glycine_and_Serine_Utilization) |
| Amino Acids and Derivatives | Alanine, serine, and glycine | [Glycine and Serine Utilization](https://rast.nmpdr.org/seedviewer.cgi?page=Subsystems&subsystem=Glycine_and_Serine_Utilization&organism=549.408) | [Seryl-tRNA synthetase (EC 6.1.1.11)](https://rast.nmpdr.org/seedviewer.cgi?page=FunctionalRole&role=Seryl-tRNA%20synthetase%20(EC%206.1.1.11)&subsystem_name=Glycine_and_Serine_Utilization) |
| Amino Acids and Derivatives | Alanine, serine, and glycine | [Glycine and Serine Utilization](https://rast.nmpdr.org/seedviewer.cgi?page=Subsystems&subsystem=Glycine_and_Serine_Utilization&organism=549.408) | [Serine transporter](https://rast.nmpdr.org/seedviewer.cgi?page=FunctionalRole&role=Serine%20transporter&subsystem_name=Glycine_and_Serine_Utilization) |
| Amino Acids and Derivatives | Alanine, serine, and glycine | [Glycine and Serine Utilization](https://rast.nmpdr.org/seedviewer.cgi?page=Subsystems&subsystem=Glycine_and_Serine_Utilization&organism=549.408) | [Phosphoserine aminotransferase (EC 2.6.1.52)](https://rast.nmpdr.org/seedviewer.cgi?page=FunctionalRole&role=Phosphoserine%20aminotransferase%20(EC%202.6.1.52)&subsystem_name=Glycine_and_Serine_Utilization) |
| Amino Acids and Derivatives | Alanine, serine, and glycine | [Glycine and Serine Utilization](https://rast.nmpdr.org/seedviewer.cgi?page=Subsystems&subsystem=Glycine_and_Serine_Utilization&organism=549.408) | [Glycine cleavage system H protein](https://rast.nmpdr.org/seedviewer.cgi?page=FunctionalRole&role=Glycine%20cleavage%20system%20H%20protein&subsystem_name=Glycine_and_Serine_Utilization) |
| Amino Acids and Derivatives | Alanine, serine, and glycine | [Glycine and Serine Utilization](https://rast.nmpdr.org/seedviewer.cgi?page=Subsystems&subsystem=Glycine_and_Serine_Utilization&organism=549.408) | [Glycine dehydrogenase [decarboxylating] (glycine cleavage system P protein) (EC 1.4.4.2)](https://rast.nmpdr.org/seedviewer.cgi?page=FunctionalRole&role=Glycine%20dehydrogenase%20%5bdecarboxylating%5d%20(glycine%20cleavage%20system%20P%20protein)%20(EC%201.4.4.2)&subsystem_name=Glycine_and_Serine_Utilization) |
| Amino Acids and Derivatives | Alanine, serine, and glycine | [Glycine and Serine Utilization](https://rast.nmpdr.org/seedviewer.cgi?page=Subsystems&subsystem=Glycine_and_Serine_Utilization&organism=549.408) | [2-amino-3-ketobutyrate coenzyme A ligase (EC 2.3.1.29)](https://rast.nmpdr.org/seedviewer.cgi?page=FunctionalRole&role=2-amino-3-ketobutyrate%20coenzyme%20A%20ligase%20(EC%202.3.1.29)&subsystem_name=Glycine_and_Serine_Utilization) |
| Amino Acids and Derivatives | Alanine, serine, and glycine | [Glycine and Serine Utilization](https://rast.nmpdr.org/seedviewer.cgi?page=Subsystems&subsystem=Glycine_and_Serine_Utilization&organism=549.408) | [L-serine dehydratase (EC 4.3.1.17)](https://rast.nmpdr.org/seedviewer.cgi?page=FunctionalRole&role=L-serine%20dehydratase%20(EC%204.3.1.17)&subsystem_name=Glycine_and_Serine_Utilization) |
| Amino Acids and Derivatives | Alanine, serine, and glycine | [Glycine and Serine Utilization](https://rast.nmpdr.org/seedviewer.cgi?page=Subsystems&subsystem=Glycine_and_Serine_Utilization&organism=549.408) | [Aminomethyltransferase (glycine cleavage system T protein) (EC 2.1.2.10)](https://rast.nmpdr.org/seedviewer.cgi?page=FunctionalRole&role=Aminomethyltransferase%20(glycine%20cleavage%20system%20T%20protein)%20(EC%202.1.2.10)&subsystem_name=Glycine_and_Serine_Utilization) |
| Amino Acids and Derivatives | Alanine, serine, and glycine | [Glycine and Serine Utilization](https://rast.nmpdr.org/seedviewer.cgi?page=Subsystems&subsystem=Glycine_and_Serine_Utilization&organism=549.408) | [Glycerate kinase (EC 2.7.1.31)](https://rast.nmpdr.org/seedviewer.cgi?page=FunctionalRole&role=Glycerate%20kinase%20(EC%202.7.1.31)&subsystem_name=Glycine_and_Serine_Utilization) |
| Sulfur Metabolism | Inorganic sulfur assimilation | [Sulfite reduction-associated complex DsrMKJOP and co-clustering genes](https://rast.nmpdr.org/seedviewer.cgi?page=Subsystems&subsystem=Sulfite_reduction-associated_complex_DsrMKJOP_and_co-clustering_genes&organism=549.408) | [tRNA 5-methylaminomethyl-2-thiouridine synthase TusC](https://rast.nmpdr.org/seedviewer.cgi?page=FunctionalRole&role=tRNA%205-methylaminomethyl-2-thiouridine%20synthase%20TusC&subsystem_name=Sulfite_reduction-associated_complex_DsrMKJOP_and_co-clustering_genes) |
| Sulfur Metabolism | Inorganic sulfur assimilation | [Sulfite reduction-associated complex DsrMKJOP and co-clustering genes](https://rast.nmpdr.org/seedviewer.cgi?page=Subsystems&subsystem=Sulfite_reduction-associated_complex_DsrMKJOP_and_co-clustering_genes&organism=549.408) | [tRNA 5-methylaminomethyl-2-thiouridine synthase TusB](https://rast.nmpdr.org/seedviewer.cgi?page=FunctionalRole&role=tRNA%205-methylaminomethyl-2-thiouridine%20synthase%20TusB&subsystem_name=Sulfite_reduction-associated_complex_DsrMKJOP_and_co-clustering_genes) |
| Sulfur Metabolism | Inorganic sulfur assimilation | [Sulfite reduction-associated complex DsrMKJOP and co-clustering genes](https://rast.nmpdr.org/seedviewer.cgi?page=Subsystems&subsystem=Sulfite_reduction-associated_complex_DsrMKJOP_and_co-clustering_genes&organism=549.408) | [tRNA 2-thiouridine synthesizing protein E (EC 2.8.1.-)](https://rast.nmpdr.org/seedviewer.cgi?page=FunctionalRole&role=tRNA%202-thiouridine%20synthesizing%20protein%20E%20(EC%202.8.1.-)&subsystem_name=Sulfite_reduction-associated_complex_DsrMKJOP_and_co-clustering_genes) |
| Sulfur Metabolism | Inorganic sulfur assimilation | [Sulfite reduction-associated complex DsrMKJOP and co-clustering genes](https://rast.nmpdr.org/seedviewer.cgi?page=Subsystems&subsystem=Sulfite_reduction-associated_complex_DsrMKJOP_and_co-clustering_genes&organism=549.408) | [tRNA 5-methylaminomethyl-2-thiouridine synthase TusD](https://rast.nmpdr.org/seedviewer.cgi?page=FunctionalRole&role=tRNA%205-methylaminomethyl-2-thiouridine%20synthase%20TusD&subsystem_name=Sulfite_reduction-associated_complex_DsrMKJOP_and_co-clustering_genes) |
| Sulfur Metabolism | Inorganic sulfur assimilation | [Inorganic Sulfur Assimilation](https://rast.nmpdr.org/seedviewer.cgi?page=Subsystems&subsystem=Inorganic_Sulfur_Assimilation&organism=549.408) | [4Fe-4S ferredoxin, iron-sulfur binding](https://rast.nmpdr.org/seedviewer.cgi?page=FunctionalRole&role=4Fe-4S%20ferredoxin,%20iron-sulfur%20binding&subsystem_name=Inorganic_Sulfur_Assimilation) |
| Sulfur Metabolism | Inorganic sulfur assimilation | [Inorganic Sulfur Assimilation](https://rast.nmpdr.org/seedviewer.cgi?page=Subsystems&subsystem=Inorganic_Sulfur_Assimilation&organism=549.408) | [Ferredoxin--NADP(+) reductase (EC 1.18.1.2)](https://rast.nmpdr.org/seedviewer.cgi?page=FunctionalRole&role=Ferredoxin--NADP(+)%20reductase%20(EC%201.18.1.2)&subsystem_name=Inorganic_Sulfur_Assimilation) |
| Sulfur Metabolism | Inorganic sulfur assimilation | [Inorganic Sulfur Assimilation](https://rast.nmpdr.org/seedviewer.cgi?page=Subsystems&subsystem=Inorganic_Sulfur_Assimilation&organism=549.408) | [Adenylylsulfate kinase (EC 2.7.1.25)](https://rast.nmpdr.org/seedviewer.cgi?page=FunctionalRole&role=Adenylylsulfate%20kinase%20(EC%202.7.1.25)&subsystem_name=Inorganic_Sulfur_Assimilation) |
| Sulfur Metabolism | Inorganic sulfur assimilation | [Inorganic Sulfur Assimilation](https://rast.nmpdr.org/seedviewer.cgi?page=Subsystems&subsystem=Inorganic_Sulfur_Assimilation&organism=549.408) | [Sulfate and thiosulfate binding protein CysP](https://rast.nmpdr.org/seedviewer.cgi?page=FunctionalRole&role=Sulfate%20and%20thiosulfate%20binding%20protein%20CysP&subsystem_name=Inorganic_Sulfur_Assimilation) |
| Sulfur Metabolism | Inorganic sulfur assimilation | [Inorganic Sulfur Assimilation](https://rast.nmpdr.org/seedviewer.cgi?page=Subsystems&subsystem=Inorganic_Sulfur_Assimilation&organism=549.408) | [3'(2'),5'-bisphosphate nucleotidase (EC 3.1.3.7)](https://rast.nmpdr.org/seedviewer.cgi?page=FunctionalRole&role=3) |
| Sulfur Metabolism | Inorganic sulfur assimilation | [Inorganic Sulfur Assimilation](https://rast.nmpdr.org/seedviewer.cgi?page=Subsystems&subsystem=Inorganic_Sulfur_Assimilation&organism=549.408) | [Sulfate transport system permease protein CysW](https://rast.nmpdr.org/seedviewer.cgi?page=FunctionalRole&role=Sulfate%20transport%20system%20permease%20protein%20CysW&subsystem_name=Inorganic_Sulfur_Assimilation) |
| Sulfur Metabolism | Inorganic sulfur assimilation | [Inorganic Sulfur Assimilation](https://rast.nmpdr.org/seedviewer.cgi?page=Subsystems&subsystem=Inorganic_Sulfur_Assimilation&organism=549.408) | [Sulfate transport system permease protein CysT](https://rast.nmpdr.org/seedviewer.cgi?page=FunctionalRole&role=Sulfate%20transport%20system%20permease%20protein%20CysT&subsystem_name=Inorganic_Sulfur_Assimilation) |
| Sulfur Metabolism | Inorganic sulfur assimilation | [Inorganic Sulfur Assimilation](https://rast.nmpdr.org/seedviewer.cgi?page=Subsystems&subsystem=Inorganic_Sulfur_Assimilation&organism=549.408) | [Phosphoadenylyl-sulfate reductase [thioredoxin] (EC 1.8.4.8)](https://rast.nmpdr.org/seedviewer.cgi?page=FunctionalRole&role=Phosphoadenylyl-sulfate%20reductase%20%5bthioredoxin%5d%20(EC%201.8.4.8)&subsystem_name=Inorganic_Sulfur_Assimilation) |
| Sulfur Metabolism | Inorganic sulfur assimilation | [Inorganic Sulfur Assimilation](https://rast.nmpdr.org/seedviewer.cgi?page=Subsystems&subsystem=Inorganic_Sulfur_Assimilation&organism=549.408) | [Sulfate adenylyltransferase subunit 1 (EC 2.7.7.4)](https://rast.nmpdr.org/seedviewer.cgi?page=FunctionalRole&role=Sulfate%20adenylyltransferase%20subunit%201%20(EC%202.7.7.4)&subsystem_name=Inorganic_Sulfur_Assimilation) |
| Sulfur Metabolism | Inorganic sulfur assimilation | [Inorganic Sulfur Assimilation](https://rast.nmpdr.org/seedviewer.cgi?page=Subsystems&subsystem=Inorganic_Sulfur_Assimilation&organism=549.408) | [Sulfate adenylyltransferase subunit 2 (EC 2.7.7.4)](https://rast.nmpdr.org/seedviewer.cgi?page=FunctionalRole&role=Sulfate%20adenylyltransferase%20subunit%202%20(EC%202.7.7.4)&subsystem_name=Inorganic_Sulfur_Assimilation) |
| Sulfur Metabolism | Inorganic sulfur assimilation | [Inorganic Sulfur Assimilation](https://rast.nmpdr.org/seedviewer.cgi?page=Subsystems&subsystem=Inorganic_Sulfur_Assimilation&organism=549.408) | [Sulfite reductase [NADPH] hemoprotein beta-component (EC 1.8.1.2)](https://rast.nmpdr.org/seedviewer.cgi?page=FunctionalRole&role=Sulfite%20reductase%20%5bNADPH%5d%20hemoprotein%20beta-component%20(EC%201.8.1.2)&subsystem_name=Inorganic_Sulfur_Assimilation) |
| Sulfur Metabolism | Inorganic sulfur assimilation | [Inorganic Sulfur Assimilation](https://rast.nmpdr.org/seedviewer.cgi?page=Subsystems&subsystem=Inorganic_Sulfur_Assimilation&organism=549.408) | [Sulfate transporter, CysZ-type](https://rast.nmpdr.org/seedviewer.cgi?page=FunctionalRole&role=Sulfate%20transporter,%20CysZ-type&subsystem_name=Inorganic_Sulfur_Assimilation) |
| Sulfur Metabolism | Inorganic sulfur assimilation | [Inorganic Sulfur Assimilation](https://rast.nmpdr.org/seedviewer.cgi?page=Subsystems&subsystem=Inorganic_Sulfur_Assimilation&organism=549.408) | [Sulfate and thiosulfate import ATP-binding protein CysA (EC 3.6.3.25)](https://rast.nmpdr.org/seedviewer.cgi?page=FunctionalRole&role=Sulfate%20and%20thiosulfate%20import%20ATP-binding%20protein%20CysA%20(EC%203.6.3.25)&subsystem_name=Inorganic_Sulfur_Assimilation) |
| Sulfur Metabolism | Inorganic sulfur assimilation | [Inorganic Sulfur Assimilation](https://rast.nmpdr.org/seedviewer.cgi?page=Subsystems&subsystem=Inorganic_Sulfur_Assimilation&organism=549.408) | [Sulfite reductase [NADPH] flavoprotein alpha-component (EC 1.8.1.2)](https://rast.nmpdr.org/seedviewer.cgi?page=FunctionalRole&role=Sulfite%20reductase%20%5bNADPH%5d%20flavoprotein%20alpha-component%20(EC%201.8.1.2)&subsystem_name=Inorganic_Sulfur_Assimilation) |
| Sulfur Metabolism | Inorganic sulfur assimilation | [Inorganic Sulfur Assimilation](https://rast.nmpdr.org/seedviewer.cgi?page=Subsystems&subsystem=Inorganic_Sulfur_Assimilation&organism=549.408) | [Sulfate-binding protein Sbp](https://rast.nmpdr.org/seedviewer.cgi?page=FunctionalRole&role=Sulfate-binding%20protein%20Sbp&subsystem_name=Inorganic_Sulfur_Assimilation) |
| Sulfur Metabolism | Inorganic sulfur assimilation | [Inorganic Sulfur Assimilation](https://rast.nmpdr.org/seedviewer.cgi?page=Subsystems&subsystem=Inorganic_Sulfur_Assimilation&organism=549.408) | [Ferredoxin](https://rast.nmpdr.org/seedviewer.cgi?page=FunctionalRole&role=Ferredoxin&subsystem_name=Inorganic_Sulfur_Assimilation) |
| Sulfur Metabolism | Sulfur Metabolism - no subcategory | [Thioredoxin-disulfide reductase](https://rast.nmpdr.org/seedviewer.cgi?page=Subsystems&subsystem=Thioredoxin-disulfide_reductase&organism=549.408) | [Thiol peroxidase, Tpx-type (EC 1.11.1.15)](https://rast.nmpdr.org/seedviewer.cgi?page=FunctionalRole&role=Thiol%20peroxidase,%20Tpx-type%20(EC%201.11.1.15)&subsystem_name=Thioredoxin-disulfide_reductase) |
| Sulfur Metabolism | Sulfur Metabolism - no subcategory | [Thioredoxin-disulfide reductase](https://rast.nmpdr.org/seedviewer.cgi?page=Subsystems&subsystem=Thioredoxin-disulfide_reductase&organism=549.408) | [Hydrogen peroxide-inducible genes activator](https://rast.nmpdr.org/seedviewer.cgi?page=FunctionalRole&role=Hydrogen%20peroxide-inducible%20genes%20activator&subsystem_name=Thioredoxin-disulfide_reductase) |
| Sulfur Metabolism | Sulfur Metabolism - no subcategory | [Thioredoxin-disulfide reductase](https://rast.nmpdr.org/seedviewer.cgi?page=Subsystems&subsystem=Thioredoxin-disulfide_reductase&organism=549.408) | [Alkyl hydroperoxide reductase protein F (EC 1.6.4.-)](https://rast.nmpdr.org/seedviewer.cgi?page=FunctionalRole&role=Alkyl%20hydroperoxide%20reductase%20protein%20F%20(EC%201.6.4.-)&subsystem_name=Thioredoxin-disulfide_reductase) |
| Sulfur Metabolism | Sulfur Metabolism - no subcategory | [Thioredoxin-disulfide reductase](https://rast.nmpdr.org/seedviewer.cgi?page=Subsystems&subsystem=Thioredoxin-disulfide_reductase&organism=549.408) | [Thiol peroxidase, Bcp-type (EC 1.11.1.15)](https://rast.nmpdr.org/seedviewer.cgi?page=FunctionalRole&role=Thiol%20peroxidase,%20Bcp-type%20(EC%201.11.1.15)&subsystem_name=Thioredoxin-disulfide_reductase) |
| Sulfur Metabolism | Sulfur Metabolism - no subcategory | [Thioredoxin-disulfide reductase](https://rast.nmpdr.org/seedviewer.cgi?page=Subsystems&subsystem=Thioredoxin-disulfide_reductase&organism=549.408) | [Alkyl hydroperoxide reductase subunit C-like protein](https://rast.nmpdr.org/seedviewer.cgi?page=FunctionalRole&role=Alkyl%20hydroperoxide%20reductase%20subunit%20C-like%20protein&subsystem_name=Thioredoxin-disulfide_reductase) |
| Sulfur Metabolism | Sulfur Metabolism - no subcategory | [Thioredoxin-disulfide reductase](https://rast.nmpdr.org/seedviewer.cgi?page=Subsystems&subsystem=Thioredoxin-disulfide_reductase&organism=549.408) | [Alkyl hydroperoxide reductase protein C (EC 1.6.4.-)](https://rast.nmpdr.org/seedviewer.cgi?page=FunctionalRole&role=Alkyl%20hydroperoxide%20reductase%20protein%20C%20(EC%201.6.4.-)&subsystem_name=Thioredoxin-disulfide_reductase) |
| Sulfur Metabolism | Sulfur Metabolism - no subcategory | [Thioredoxin-disulfide reductase](https://rast.nmpdr.org/seedviewer.cgi?page=Subsystems&subsystem=Thioredoxin-disulfide_reductase&organism=549.408) | [Thioredoxin reductase (EC 1.8.1.9)](https://rast.nmpdr.org/seedviewer.cgi?page=FunctionalRole&role=Thioredoxin%20reductase%20(EC%201.8.1.9)&subsystem_name=Thioredoxin-disulfide_reductase) |
| Sulfur Metabolism | Sulfur Metabolism - no subcategory | [Galactosylceramide and Sulfatide metabolism](https://rast.nmpdr.org/seedviewer.cgi?page=Subsystems&subsystem=Galactosylceramide_and_Sulfatide_metabolism&organism=549.408) | [Beta-galactosidase (EC 3.2.1.23)](https://rast.nmpdr.org/seedviewer.cgi?page=FunctionalRole&role=Beta-galactosidase%20(EC%203.2.1.23)&subsystem_name=Galactosylceramide_and_Sulfatide_metabolism) |
| Sulfur Metabolism | Sulfur Metabolism - no subcategory | [Galactosylceramide and Sulfatide metabolism](https://rast.nmpdr.org/seedviewer.cgi?page=Subsystems&subsystem=Galactosylceramide_and_Sulfatide_metabolism&organism=549.408) | [Arylsulfatase (EC 3.1.6.1)](https://rast.nmpdr.org/seedviewer.cgi?page=FunctionalRole&role=Arylsulfatase%20(EC%203.1.6.1)&subsystem_name=Galactosylceramide_and_Sulfatide_metabolism) |
| Sulfur Metabolism | Organic sulfur assimilation | [L-Cystine Uptake and Metabolism](https://rast.nmpdr.org/seedviewer.cgi?page=Subsystems&subsystem=L-Cystine_Uptake_and_Metabolism&organism=549.408) | [Cystine ABC transporter, permease protein](https://rast.nmpdr.org/seedviewer.cgi?page=FunctionalRole&role=Cystine%20ABC%20transporter,%20permease%20protein&subsystem_name=L-Cystine_Uptake_and_Metabolism) |
| Sulfur Metabolism | Organic sulfur assimilation | [L-Cystine Uptake and Metabolism](https://rast.nmpdr.org/seedviewer.cgi?page=Subsystems&subsystem=L-Cystine_Uptake_and_Metabolism&organism=549.408) | [Cystine ABC transporter, ATP-binding protein](https://rast.nmpdr.org/seedviewer.cgi?page=FunctionalRole&role=Cystine%20ABC%20transporter,%20ATP-binding%20protein&subsystem_name=L-Cystine_Uptake_and_Metabolism) |
| Sulfur Metabolism | Organic sulfur assimilation | [L-Cystine Uptake and Metabolism](https://rast.nmpdr.org/seedviewer.cgi?page=Subsystems&subsystem=L-Cystine_Uptake_and_Metabolism&organism=549.408) | [Cystine ABC transporter, periplasmic cystine-binding protein FliY](https://rast.nmpdr.org/seedviewer.cgi?page=FunctionalRole&role=Cystine%20ABC%20transporter,%20periplasmic%20cystine-binding%20protein%20FliY&subsystem_name=L-Cystine_Uptake_and_Metabolism) |
| Sulfur Metabolism | Organic sulfur assimilation | [L-Cystine Uptake and Metabolism](https://rast.nmpdr.org/seedviewer.cgi?page=Subsystems&subsystem=L-Cystine_Uptake_and_Metabolism&organism=549.408) | [Cystathionine beta-lyase (EC 4.4.1.8)](https://rast.nmpdr.org/seedviewer.cgi?page=FunctionalRole&role=Cystathionine%20beta-lyase%20(EC%204.4.1.8)&subsystem_name=L-Cystine_Uptake_and_Metabolism) |
| Sulfur Metabolism | Organic sulfur assimilation | [L-Cystine Uptake and Metabolism](https://rast.nmpdr.org/seedviewer.cgi?page=Subsystems&subsystem=L-Cystine_Uptake_and_Metabolism&organism=549.408) | [L-cystine uptake protein TcyP](https://rast.nmpdr.org/seedviewer.cgi?page=FunctionalRole&role=L-cystine%20uptake%20protein%20TcyP&subsystem_name=L-Cystine_Uptake_and_Metabolism) |
| Sulfur Metabolism | Organic sulfur assimilation | [L-Cystine Uptake and Metabolism](https://rast.nmpdr.org/seedviewer.cgi?page=Subsystems&subsystem=L-Cystine_Uptake_and_Metabolism&organism=549.408) | [Bacterial luciferase family protein YtmO, in cluster with L-cystine ABC transporter](https://rast.nmpdr.org/seedviewer.cgi?page=FunctionalRole&role=Bacterial%20luciferase%20family%20protein%20YtmO,%20in%20cluster%20with%20L-cystine%20ABC%20transporter&subsystem_name=L-Cystine_Uptake_and_Metabolism) |
| Sulfur Metabolism | Organic sulfur assimilation | [L-Cystine Uptake and Metabolism](https://rast.nmpdr.org/seedviewer.cgi?page=Subsystems&subsystem=L-Cystine_Uptake_and_Metabolism&organism=549.408) | [Cystathionine gamma-synthase (EC 2.5.1.48)](https://rast.nmpdr.org/seedviewer.cgi?page=FunctionalRole&role=Cystathionine%20gamma-synthase%20(EC%202.5.1.48)&subsystem_name=L-Cystine_Uptake_and_Metabolism) |
| Sulfur Metabolism | Organic sulfur assimilation | [Taurine Utilization](https://rast.nmpdr.org/seedviewer.cgi?page=Subsystems&subsystem=Taurine_Utilization&organism=549.408) | [Taurine transport ATP-binding protein TauB](https://rast.nmpdr.org/seedviewer.cgi?page=FunctionalRole&role=Taurine%20transport%20ATP-binding%20protein%20TauB&subsystem_name=Taurine_Utilization) |
| Sulfur Metabolism | Organic sulfur assimilation | [Taurine Utilization](https://rast.nmpdr.org/seedviewer.cgi?page=Subsystems&subsystem=Taurine_Utilization&organism=549.408) | [Taurine transport system permease protein TauC](https://rast.nmpdr.org/seedviewer.cgi?page=FunctionalRole&role=Taurine%20transport%20system%20permease%20protein%20TauC&subsystem_name=Taurine_Utilization) |
| Sulfur Metabolism | Organic sulfur assimilation | [Taurine Utilization](https://rast.nmpdr.org/seedviewer.cgi?page=Subsystems&subsystem=Taurine_Utilization&organism=549.408) | [Taurine-binding periplasmic protein TauA](https://rast.nmpdr.org/seedviewer.cgi?page=FunctionalRole&role=Taurine-binding%20periplasmic%20protein%20TauA&subsystem_name=Taurine_Utilization) |
| Sulfur Metabolism | Organic sulfur assimilation | [Alkanesulfonate assimilation](https://rast.nmpdr.org/seedviewer.cgi?page=Subsystems&subsystem=Alkanesulfonate_assimilation&organism=549.408) | [Alkanesulfonates transport system permease protein](https://rast.nmpdr.org/seedviewer.cgi?page=FunctionalRole&role=Alkanesulfonates%20transport%20system%20permease%20protein&subsystem_name=Alkanesulfonate_assimilation) |
| Sulfur Metabolism | Organic sulfur assimilation | [Alkanesulfonate assimilation](https://rast.nmpdr.org/seedviewer.cgi?page=Subsystems&subsystem=Alkanesulfonate_assimilation&organism=549.408) | [Alkanesulfonates ABC transporter ATP-binding protein](https://rast.nmpdr.org/seedviewer.cgi?page=FunctionalRole&role=Alkanesulfonates%20ABC%20transporter%20ATP-binding%20protein&subsystem_name=Alkanesulfonate_assimilation) |
| Sulfur Metabolism | Organic sulfur assimilation | [Alkanesulfonate assimilation](https://rast.nmpdr.org/seedviewer.cgi?page=Subsystems&subsystem=Alkanesulfonate_assimilation&organism=549.408) | [sulfonate monooxygenase](https://rast.nmpdr.org/seedviewer.cgi?page=FunctionalRole&role=sulfonate%20monooxygenase&subsystem_name=Alkanesulfonate_assimilation) |
| Sulfur Metabolism | Organic sulfur assimilation | [Alkanesulfonate assimilation](https://rast.nmpdr.org/seedviewer.cgi?page=Subsystems&subsystem=Alkanesulfonate_assimilation&organism=549.408) | [Arylsulfatase (EC 3.1.6.1)](https://rast.nmpdr.org/seedviewer.cgi?page=FunctionalRole&role=Arylsulfatase%20(EC%203.1.6.1)&subsystem_name=Alkanesulfonate_assimilation) |
| Sulfur Metabolism | Organic sulfur assimilation | [Alkanesulfonate assimilation](https://rast.nmpdr.org/seedviewer.cgi?page=Subsystems&subsystem=Alkanesulfonate_assimilation&organism=549.408) | [Putative alkylsulfatase (EC 1.14.11.17)](https://rast.nmpdr.org/seedviewer.cgi?page=FunctionalRole&role=Putative%20alkylsulfatase%20(EC%201.14.11.17)&subsystem_name=Alkanesulfonate_assimilation) |
| Sulfur Metabolism | Organic sulfur assimilation | [Alkanesulfonate assimilation](https://rast.nmpdr.org/seedviewer.cgi?page=Subsystems&subsystem=Alkanesulfonate_assimilation&organism=549.408) | [ABC-type nitrate/sulfonate/bicarbonate transport system, ATPase component](https://rast.nmpdr.org/seedviewer.cgi?page=FunctionalRole&role=ABC-type%20nitrate/sulfonate/bicarbonate%20transport%20system,%20ATPase%20component&subsystem_name=Alkanesulfonate_assimilation) |
| Sulfur Metabolism | Organic sulfur assimilation | [Alkanesulfonate assimilation](https://rast.nmpdr.org/seedviewer.cgi?page=Subsystems&subsystem=Alkanesulfonate_assimilation&organism=549.408) | [Alkanesulfonates-binding protein](https://rast.nmpdr.org/seedviewer.cgi?page=FunctionalRole&role=Alkanesulfonates-binding%20protein&subsystem_name=Alkanesulfonate_assimilation) |
| Sulfur Metabolism | Organic sulfur assimilation | [Alkanesulfonate assimilation](https://rast.nmpdr.org/seedviewer.cgi?page=Subsystems&subsystem=Alkanesulfonate_assimilation&organism=549.408) | [FMN reductase (EC 1.5.1.29)](https://rast.nmpdr.org/seedviewer.cgi?page=FunctionalRole&role=FMN%20reductase%20(EC%201.5.1.29)&subsystem_name=Alkanesulfonate_assimilation) |
| Sulfur Metabolism | Organic sulfur assimilation | [Alkanesulfonate assimilation](https://rast.nmpdr.org/seedviewer.cgi?page=Subsystems&subsystem=Alkanesulfonate_assimilation&organism=549.408) | [Alkanesulfonate monooxygenase (EC 1.14.14.5)](https://rast.nmpdr.org/seedviewer.cgi?page=FunctionalRole&role=Alkanesulfonate%20monooxygenase%20(EC%201.14.14.5)&subsystem_name=Alkanesulfonate_assimilation) |
| Sulfur Metabolism | Organic sulfur assimilation | [Alkanesulfonates Utilization](https://rast.nmpdr.org/seedviewer.cgi?page=Subsystems&subsystem=Alkanesulfonates_Utilization&organism=549.408) | [Alkanesulfonates-binding protein](https://rast.nmpdr.org/seedviewer.cgi?page=FunctionalRole&role=Alkanesulfonates-binding%20protein&subsystem_name=Alkanesulfonates_Utilization) |
| Sulfur Metabolism | Organic sulfur assimilation | [Alkanesulfonates Utilization](https://rast.nmpdr.org/seedviewer.cgi?page=Subsystems&subsystem=Alkanesulfonates_Utilization&organism=549.408) | [Alkanesulfonates transport system permease protein](https://rast.nmpdr.org/seedviewer.cgi?page=FunctionalRole&role=Alkanesulfonates%20transport%20system%20permease%20protein&subsystem_name=Alkanesulfonates_Utilization) |
| Sulfur Metabolism | Organic sulfur assimilation | [Alkanesulfonates Utilization](https://rast.nmpdr.org/seedviewer.cgi?page=Subsystems&subsystem=Alkanesulfonates_Utilization&organism=549.408) | [FMN reductase (EC 1.5.1.29)](https://rast.nmpdr.org/seedviewer.cgi?page=FunctionalRole&role=FMN%20reductase%20(EC%201.5.1.29)&subsystem_name=Alkanesulfonates_Utilization) |
| Sulfur Metabolism | Organic sulfur assimilation | [Alkanesulfonates Utilization](https://rast.nmpdr.org/seedviewer.cgi?page=Subsystems&subsystem=Alkanesulfonates_Utilization&organism=549.408) | [Alkanesulfonates ABC transporter ATP-binding protein](https://rast.nmpdr.org/seedviewer.cgi?page=FunctionalRole&role=Alkanesulfonates%20ABC%20transporter%20ATP-binding%20protein&subsystem_name=Alkanesulfonates_Utilization) |
| Sulfur Metabolism | Organic sulfur assimilation | [Alkanesulfonates Utilization](https://rast.nmpdr.org/seedviewer.cgi?page=Subsystems&subsystem=Alkanesulfonates_Utilization&organism=549.408) | [Alkanesulfonate monooxygenase (EC 1.14.14.5)](https://rast.nmpdr.org/seedviewer.cgi?page=FunctionalRole&role=Alkanesulfonate%20monooxygenase%20(EC%201.14.14.5)&subsystem_name=Alkanesulfonates_Utilization) |
| Phosphorus Metabolism | Phosphorus Metabolism - no subcategory | [High affinity phosphate transporter and control of PHO regulon](https://rast.nmpdr.org/seedviewer.cgi?page=Subsystems&subsystem=High_affinity_phosphate_transporter_and_control_of_PHO_regulon&organism=549.408) | [Phosphate regulon transcriptional regulatory protein PhoB (SphR)](https://rast.nmpdr.org/seedviewer.cgi?page=FunctionalRole&role=Phosphate%20regulon%20transcriptional%20regulatory%20protein%20PhoB%20(SphR)&subsystem_name=High_affinity_phosphate_transporter_and_control_of_PHO_regulon) |
| Phosphorus Metabolism | Phosphorus Metabolism - no subcategory | [High affinity phosphate transporter and control of PHO regulon](https://rast.nmpdr.org/seedviewer.cgi?page=Subsystems&subsystem=High_affinity_phosphate_transporter_and_control_of_PHO_regulon&organism=549.408) | [Phosphate transport system permease protein PstC (TC 3.A.1.7.1)](https://rast.nmpdr.org/seedviewer.cgi?page=FunctionalRole&role=Phosphate%20transport%20system%20permease%20protein%20PstC%20(TC%203.A.1.7.1)&subsystem_name=High_affinity_phosphate_transporter_and_control_of_PHO_regulon) |
| Phosphorus Metabolism | Phosphorus Metabolism - no subcategory | [High affinity phosphate transporter and control of PHO regulon](https://rast.nmpdr.org/seedviewer.cgi?page=Subsystems&subsystem=High_affinity_phosphate_transporter_and_control_of_PHO_regulon&organism=549.408) | [Phosphate transport ATP-binding protein PstB (TC 3.A.1.7.1)](https://rast.nmpdr.org/seedviewer.cgi?page=FunctionalRole&role=Phosphate%20transport%20ATP-binding%20protein%20PstB%20(TC%203.A.1.7.1)&subsystem_name=High_affinity_phosphate_transporter_and_control_of_PHO_regulon) |
| Phosphorus Metabolism | Phosphorus Metabolism - no subcategory | [High affinity phosphate transporter and control of PHO regulon](https://rast.nmpdr.org/seedviewer.cgi?page=Subsystems&subsystem=High_affinity_phosphate_transporter_and_control_of_PHO_regulon&organism=549.408) | [Phosphate transport system permease protein PstA (TC 3.A.1.7.1)](https://rast.nmpdr.org/seedviewer.cgi?page=FunctionalRole&role=Phosphate%20transport%20system%20permease%20protein%20PstA%20(TC%203.A.1.7.1)&subsystem_name=High_affinity_phosphate_transporter_and_control_of_PHO_regulon) |
| Phosphorus Metabolism | Phosphorus Metabolism - no subcategory | [High affinity phosphate transporter and control of PHO regulon](https://rast.nmpdr.org/seedviewer.cgi?page=Subsystems&subsystem=High_affinity_phosphate_transporter_and_control_of_PHO_regulon&organism=549.408) | [Phosphate transport system regulatory protein PhoU](https://rast.nmpdr.org/seedviewer.cgi?page=FunctionalRole&role=Phosphate%20transport%20system%20regulatory%20protein%20PhoU&subsystem_name=High_affinity_phosphate_transporter_and_control_of_PHO_regulon) |
| Phosphorus Metabolism | Phosphorus Metabolism - no subcategory | [High affinity phosphate transporter and control of PHO regulon](https://rast.nmpdr.org/seedviewer.cgi?page=Subsystems&subsystem=High_affinity_phosphate_transporter_and_control_of_PHO_regulon&organism=549.408) | [Phosphate ABC transporter, periplasmic phosphate-binding protein PstS (TC 3.A.1.7.1)](https://rast.nmpdr.org/seedviewer.cgi?page=FunctionalRole&role=Phosphate%20ABC%20transporter,%20periplasmic%20phosphate-binding%20protein%20PstS%20(TC%203.A.1.7.1)&subsystem_name=High_affinity_phosphate_transporter_and_control_of_PHO_regulon) |
| Phosphorus Metabolism | Phosphorus Metabolism - no subcategory | [High affinity phosphate transporter and control of PHO regulon](https://rast.nmpdr.org/seedviewer.cgi?page=Subsystems&subsystem=High_affinity_phosphate_transporter_and_control_of_PHO_regulon&organism=549.408) | [Polyphosphate kinase (EC 2.7.4.1)](https://rast.nmpdr.org/seedviewer.cgi?page=FunctionalRole&role=Polyphosphate%20kinase%20(EC%202.7.4.1)&subsystem_name=High_affinity_phosphate_transporter_and_control_of_PHO_regulon) |
| Phosphorus Metabolism | Phosphorus Metabolism - no subcategory | [High affinity phosphate transporter and control of PHO regulon](https://rast.nmpdr.org/seedviewer.cgi?page=Subsystems&subsystem=High_affinity_phosphate_transporter_and_control_of_PHO_regulon&organism=549.408) | [Phosphate regulon sensor protein PhoR (SphS) (EC 2.7.13.3)](https://rast.nmpdr.org/seedviewer.cgi?page=FunctionalRole&role=Phosphate%20regulon%20sensor%20protein%20PhoR%20(SphS)%20(EC%202.7.13.3)&subsystem_name=High_affinity_phosphate_transporter_and_control_of_PHO_regulon) |
| Phosphorus Metabolism | Phosphorus Metabolism - no subcategory | [Phosphate metabolism](https://rast.nmpdr.org/seedviewer.cgi?page=Subsystems&subsystem=Phosphate_metabolism&organism=549.408) | [NAD(P) transhydrogenase subunit beta (EC 1.6.1.2)](https://rast.nmpdr.org/seedviewer.cgi?page=FunctionalRole&role=NAD(P)%20transhydrogenase%20subunit%20beta%20(EC%201.6.1.2)&subsystem_name=Phosphate_metabolism) |
| Phosphorus Metabolism | Phosphorus Metabolism - no subcategory | [Phosphate metabolism](https://rast.nmpdr.org/seedviewer.cgi?page=Subsystems&subsystem=Phosphate_metabolism&organism=549.408) | [Phosphate transport system permease protein PstC (TC 3.A.1.7.1)](https://rast.nmpdr.org/seedviewer.cgi?page=FunctionalRole&role=Phosphate%20transport%20system%20permease%20protein%20PstC%20(TC%203.A.1.7.1)&subsystem_name=Phosphate_metabolism) |
| Phosphorus Metabolism | Phosphorus Metabolism - no subcategory | [Phosphate metabolism](https://rast.nmpdr.org/seedviewer.cgi?page=Subsystems&subsystem=Phosphate_metabolism&organism=549.408) | [Phosphate transport system permease protein PstA (TC 3.A.1.7.1)](https://rast.nmpdr.org/seedviewer.cgi?page=FunctionalRole&role=Phosphate%20transport%20system%20permease%20protein%20PstA%20(TC%203.A.1.7.1)&subsystem_name=Phosphate_metabolism) |
| Phosphorus Metabolism | Phosphorus Metabolism - no subcategory | [Phosphate metabolism](https://rast.nmpdr.org/seedviewer.cgi?page=Subsystems&subsystem=Phosphate_metabolism&organism=549.408) | [Exopolyphosphatase (EC 3.6.1.11)](https://rast.nmpdr.org/seedviewer.cgi?page=FunctionalRole&role=Exopolyphosphatase%20(EC%203.6.1.11)&subsystem_name=Phosphate_metabolism) |
| Phosphorus Metabolism | Phosphorus Metabolism - no subcategory | [Phosphate metabolism](https://rast.nmpdr.org/seedviewer.cgi?page=Subsystems&subsystem=Phosphate_metabolism&organism=549.408) | [Phosphate transport system regulatory protein PhoU](https://rast.nmpdr.org/seedviewer.cgi?page=FunctionalRole&role=Phosphate%20transport%20system%20regulatory%20protein%20PhoU&subsystem_name=Phosphate_metabolism) |
| Phosphorus Metabolism | Phosphorus Metabolism - no subcategory | [Phosphate metabolism](https://rast.nmpdr.org/seedviewer.cgi?page=Subsystems&subsystem=Phosphate_metabolism&organism=549.408) | [NAD(P) transhydrogenase alpha subunit (EC 1.6.1.2)](https://rast.nmpdr.org/seedviewer.cgi?page=FunctionalRole&role=NAD(P)%20transhydrogenase%20alpha%20subunit%20(EC%201.6.1.2)&subsystem_name=Phosphate_metabolism) |
| Phosphorus Metabolism | Phosphorus Metabolism - no subcategory | [Phosphate metabolism](https://rast.nmpdr.org/seedviewer.cgi?page=Subsystems&subsystem=Phosphate_metabolism&organism=549.408) | [Polyphosphate kinase (EC 2.7.4.1)](https://rast.nmpdr.org/seedviewer.cgi?page=FunctionalRole&role=Polyphosphate%20kinase%20(EC%202.7.4.1)&subsystem_name=Phosphate_metabolism) |
| Phosphorus Metabolism | Phosphorus Metabolism - no subcategory | [Phosphate metabolism](https://rast.nmpdr.org/seedviewer.cgi?page=Subsystems&subsystem=Phosphate_metabolism&organism=549.408) | [Low-affinity inorganic phosphate transporter](https://rast.nmpdr.org/seedviewer.cgi?page=FunctionalRole&role=Low-affinity%20inorganic%20phosphate%20transporter&subsystem_name=Phosphate_metabolism) |
| Phosphorus Metabolism | Phosphorus Metabolism - no subcategory | [Phosphate metabolism](https://rast.nmpdr.org/seedviewer.cgi?page=Subsystems&subsystem=Phosphate_metabolism&organism=549.408) | [Phosphate starvation-inducible protein PhoH, predicted ATPase](https://rast.nmpdr.org/seedviewer.cgi?page=FunctionalRole&role=Phosphate%20starvation-inducible%20protein%20PhoH,%20predicted%20ATPase&subsystem_name=Phosphate_metabolism) |
| Phosphorus Metabolism | Phosphorus Metabolism - no subcategory | [Phosphate metabolism](https://rast.nmpdr.org/seedviewer.cgi?page=Subsystems&subsystem=Phosphate_metabolism&organism=549.408) | [Sodium-dependent phosphate transporter](https://rast.nmpdr.org/seedviewer.cgi?page=FunctionalRole&role=Sodium-dependent%20phosphate%20transporter&subsystem_name=Phosphate_metabolism) |
| Phosphorus Metabolism | Phosphorus Metabolism - no subcategory | [Phosphate metabolism](https://rast.nmpdr.org/seedviewer.cgi?page=Subsystems&subsystem=Phosphate_metabolism&organism=549.408) | [Phosphate regulon sensor protein PhoR (SphS) (EC 2.7.13.3)](https://rast.nmpdr.org/seedviewer.cgi?page=FunctionalRole&role=Phosphate%20regulon%20sensor%20protein%20PhoR%20(SphS)%20(EC%202.7.13.3)&subsystem_name=Phosphate_metabolism) |
| Phosphorus Metabolism | Phosphorus Metabolism - no subcategory | [Phosphate metabolism](https://rast.nmpdr.org/seedviewer.cgi?page=Subsystems&subsystem=Phosphate_metabolism&organism=549.408) | [Guanosine-5'-triphosphate,3'-diphosphate pyrophosphatase (EC 3.6.1.40)](https://rast.nmpdr.org/seedviewer.cgi?page=FunctionalRole&role=Guanosine-5) |
| Phosphorus Metabolism | Phosphorus Metabolism - no subcategory | [Phosphate metabolism](https://rast.nmpdr.org/seedviewer.cgi?page=Subsystems&subsystem=Phosphate_metabolism&organism=549.408) | [Inorganic pyrophosphatase (EC 3.6.1.1)](https://rast.nmpdr.org/seedviewer.cgi?page=FunctionalRole&role=Inorganic%20pyrophosphatase%20(EC%203.6.1.1)&subsystem_name=Phosphate_metabolism) |
| Phosphorus Metabolism | Phosphorus Metabolism - no subcategory | [Phosphate metabolism](https://rast.nmpdr.org/seedviewer.cgi?page=Subsystems&subsystem=Phosphate_metabolism&organism=549.408) | [Phosphate regulon transcriptional regulatory protein PhoB (SphR)](https://rast.nmpdr.org/seedviewer.cgi?page=FunctionalRole&role=Phosphate%20regulon%20transcriptional%20regulatory%20protein%20PhoB%20(SphR)&subsystem_name=Phosphate_metabolism) |
| Phosphorus Metabolism | Phosphorus Metabolism - no subcategory | [Phosphate metabolism](https://rast.nmpdr.org/seedviewer.cgi?page=Subsystems&subsystem=Phosphate_metabolism&organism=549.408) | [Phosphate transport ATP-binding protein PstB (TC 3.A.1.7.1)](https://rast.nmpdr.org/seedviewer.cgi?page=FunctionalRole&role=Phosphate%20transport%20ATP-binding%20protein%20PstB%20(TC%203.A.1.7.1)&subsystem_name=Phosphate_metabolism) |
| Phosphorus Metabolism | Phosphorus Metabolism - no subcategory | [Phosphate metabolism](https://rast.nmpdr.org/seedviewer.cgi?page=Subsystems&subsystem=Phosphate_metabolism&organism=549.408) | [Phosphate ABC transporter, periplasmic phosphate-binding protein PstS (TC 3.A.1.7.1)](https://rast.nmpdr.org/seedviewer.cgi?page=FunctionalRole&role=Phosphate%20ABC%20transporter,%20periplasmic%20phosphate-binding%20protein%20PstS%20(TC%203.A.1.7.1)&subsystem_name=Phosphate_metabolism) |
| Phosphorus Metabolism | Phosphorus Metabolism - no subcategory | [Phosphate metabolism](https://rast.nmpdr.org/seedviewer.cgi?page=Subsystems&subsystem=Phosphate_metabolism&organism=549.408) | [Soluble pyridine nucleotide transhydrogenase (EC 1.6.1.1)](https://rast.nmpdr.org/seedviewer.cgi?page=FunctionalRole&role=Soluble%20pyridine%20nucleotide%20transhydrogenase%20(EC%201.6.1.1)&subsystem_name=Phosphate_metabolism) |
| Phosphorus Metabolism | Phosphorus Metabolism - no subcategory | [Polyphosphate](https://rast.nmpdr.org/seedviewer.cgi?page=Subsystems&subsystem=Polyphosphate&organism=549.408) | [Guanosine-5'-triphosphate,3'-diphosphate pyrophosphatase (EC 3.6.1.40)](https://rast.nmpdr.org/seedviewer.cgi?page=FunctionalRole&role=Guanosine-5) |
| Phosphorus Metabolism | Phosphorus Metabolism - no subcategory | [Polyphosphate](https://rast.nmpdr.org/seedviewer.cgi?page=Subsystems&subsystem=Polyphosphate&organism=549.408) | [Polyphosphate kinase (EC 2.7.4.1)](https://rast.nmpdr.org/seedviewer.cgi?page=FunctionalRole&role=Polyphosphate%20kinase%20(EC%202.7.4.1)&subsystem_name=Polyphosphate) |
| Phosphorus Metabolism | Phosphorus Metabolism - no subcategory | [Polyphosphate](https://rast.nmpdr.org/seedviewer.cgi?page=Subsystems&subsystem=Polyphosphate&organism=549.408) | [Exopolyphosphatase (EC 3.6.1.11)](https://rast.nmpdr.org/seedviewer.cgi?page=FunctionalRole&role=Exopolyphosphatase%20(EC%203.6.1.11)&subsystem_name=Polyphosphate) |
| Phosphorus Metabolism | Phosphorus Metabolism - no subcategory | [Alkylphosphonate utilization](https://rast.nmpdr.org/seedviewer.cgi?page=Subsystems&subsystem=Alkylphosphonate_utilization&organism=549.408) | [PhnO protein](https://rast.nmpdr.org/seedviewer.cgi?page=FunctionalRole&role=PhnO%20protein&subsystem_name=Alkylphosphonate_utilization) |
| Phosphorus Metabolism | Phosphorus Metabolism - no subcategory | [Alkylphosphonate utilization](https://rast.nmpdr.org/seedviewer.cgi?page=Subsystems&subsystem=Alkylphosphonate_utilization&organism=549.408) | [Transcriptional regulator PhnF](https://rast.nmpdr.org/seedviewer.cgi?page=FunctionalRole&role=Transcriptional%20regulator%20PhnF&subsystem_name=Alkylphosphonate_utilization) |
| Phosphorus Metabolism | Phosphorus Metabolism - no subcategory | [Alkylphosphonate utilization](https://rast.nmpdr.org/seedviewer.cgi?page=Subsystems&subsystem=Alkylphosphonate_utilization&organism=549.408) | [Alkylphosphonate utilization operon protein PhnA](https://rast.nmpdr.org/seedviewer.cgi?page=FunctionalRole&role=Alkylphosphonate%20utilization%20operon%20protein%20PhnA&subsystem_name=Alkylphosphonate_utilization) |
| Phosphorus Metabolism | Phosphorus Metabolism - no subcategory | [Alkylphosphonate utilization](https://rast.nmpdr.org/seedviewer.cgi?page=Subsystems&subsystem=Alkylphosphonate_utilization&organism=549.408) | [PhnG protein](https://rast.nmpdr.org/seedviewer.cgi?page=FunctionalRole&role=PhnG%20protein&subsystem_name=Alkylphosphonate_utilization) |
| Phosphorus Metabolism | Phosphorus Metabolism - no subcategory | [Alkylphosphonate utilization](https://rast.nmpdr.org/seedviewer.cgi?page=Subsystems&subsystem=Alkylphosphonate_utilization&organism=549.408) | [PhnI protein](https://rast.nmpdr.org/seedviewer.cgi?page=FunctionalRole&role=PhnI%20protein&subsystem_name=Alkylphosphonate_utilization) |
| Phosphorus Metabolism | Phosphorus Metabolism - no subcategory | [Alkylphosphonate utilization](https://rast.nmpdr.org/seedviewer.cgi?page=Subsystems&subsystem=Alkylphosphonate_utilization&organism=549.408) | [Phosphonates transport ATP-binding protein PhnK](https://rast.nmpdr.org/seedviewer.cgi?page=FunctionalRole&role=Phosphonates%20transport%20ATP-binding%20protein%20PhnK&subsystem_name=Alkylphosphonate_utilization) |
| Phosphorus Metabolism | Phosphorus Metabolism - no subcategory | [Alkylphosphonate utilization](https://rast.nmpdr.org/seedviewer.cgi?page=Subsystems&subsystem=Alkylphosphonate_utilization&organism=549.408) | [ATP-binding protein PhnN](https://rast.nmpdr.org/seedviewer.cgi?page=FunctionalRole&role=ATP-binding%20protein%20PhnN&subsystem_name=Alkylphosphonate_utilization) |
| Phosphorus Metabolism | Phosphorus Metabolism - no subcategory | [Alkylphosphonate utilization](https://rast.nmpdr.org/seedviewer.cgi?page=Subsystems&subsystem=Alkylphosphonate_utilization&organism=549.408) | [PhnH protein](https://rast.nmpdr.org/seedviewer.cgi?page=FunctionalRole&role=PhnH%20protein&subsystem_name=Alkylphosphonate_utilization) |
| Phosphorus Metabolism | Phosphorus Metabolism - no subcategory | [Alkylphosphonate utilization](https://rast.nmpdr.org/seedviewer.cgi?page=Subsystems&subsystem=Alkylphosphonate_utilization&organism=549.408) | [Protein RcsF](https://rast.nmpdr.org/seedviewer.cgi?page=FunctionalRole&role=Protein%20RcsF&subsystem_name=Alkylphosphonate_utilization) |
| Phosphorus Metabolism | Phosphorus Metabolism - no subcategory | [Alkylphosphonate utilization](https://rast.nmpdr.org/seedviewer.cgi?page=Subsystems&subsystem=Alkylphosphonate_utilization&organism=549.408) | [Phosphonates transport ATP-binding protein PhnL](https://rast.nmpdr.org/seedviewer.cgi?page=FunctionalRole&role=Phosphonates%20transport%20ATP-binding%20protein%20PhnL&subsystem_name=Alkylphosphonate_utilization) |
| Phosphorus Metabolism | Phosphorus Metabolism - no subcategory | [Alkylphosphonate utilization](https://rast.nmpdr.org/seedviewer.cgi?page=Subsystems&subsystem=Alkylphosphonate_utilization&organism=549.408) | [Metal-dependent hydrolase involved in phosphonate metabolism](https://rast.nmpdr.org/seedviewer.cgi?page=FunctionalRole&role=Metal-dependent%20hydrolase%20involved%20in%20phosphonate%20metabolism&subsystem_name=Alkylphosphonate_utilization) |
| Phosphorus Metabolism | Phosphorus Metabolism - no subcategory | [Alkylphosphonate utilization](https://rast.nmpdr.org/seedviewer.cgi?page=Subsystems&subsystem=Alkylphosphonate_utilization&organism=549.408) | [PhnB protein](https://rast.nmpdr.org/seedviewer.cgi?page=FunctionalRole&role=PhnB%20protein&subsystem_name=Alkylphosphonate_utilization) |
| Phosphorus Metabolism | Phosphorus Metabolism - no subcategory | [Alkylphosphonate utilization](https://rast.nmpdr.org/seedviewer.cgi?page=Subsystems&subsystem=Alkylphosphonate_utilization&organism=549.408) | [PhnJ protein](https://rast.nmpdr.org/seedviewer.cgi?page=FunctionalRole&role=PhnJ%20protein&subsystem_name=Alkylphosphonate_utilization) |
| Carbohydrates | Central carbohydrate metabolism | [Methylglyoxal Metabolism](https://rast.nmpdr.org/seedviewer.cgi?page=Subsystems&subsystem=Methylglyoxal_Metabolism&organism=549.408) | [Methylglyoxal synthase (EC 4.2.3.3)](https://rast.nmpdr.org/seedviewer.cgi?page=FunctionalRole&role=Methylglyoxal%20synthase%20(EC%204.2.3.3)&subsystem_name=Methylglyoxal_Metabolism) |
| Carbohydrates | Central carbohydrate metabolism | [Methylglyoxal Metabolism](https://rast.nmpdr.org/seedviewer.cgi?page=Subsystems&subsystem=Methylglyoxal_Metabolism&organism=549.408) | [Lactoylglutathione lyase (EC 4.4.1.5)](https://rast.nmpdr.org/seedviewer.cgi?page=FunctionalRole&role=Lactoylglutathione%20lyase%20(EC%204.4.1.5)&subsystem_name=Methylglyoxal_Metabolism) |
| Carbohydrates | Central carbohydrate metabolism | [Methylglyoxal Metabolism](https://rast.nmpdr.org/seedviewer.cgi?page=Subsystems&subsystem=Methylglyoxal_Metabolism&organism=549.408) | [Aldehyde dehydrogenase B (EC 1.2.1.22)](https://rast.nmpdr.org/seedviewer.cgi?page=FunctionalRole&role=Aldehyde%20dehydrogenase%20B%20(EC%201.2.1.22)&subsystem_name=Methylglyoxal_Metabolism) |
| Carbohydrates | Central carbohydrate metabolism | [Methylglyoxal Metabolism](https://rast.nmpdr.org/seedviewer.cgi?page=Subsystems&subsystem=Methylglyoxal_Metabolism&organism=549.408) | [Aldehyde dehydrogenase (EC 1.2.1.3)](https://rast.nmpdr.org/seedviewer.cgi?page=FunctionalRole&role=Aldehyde%20dehydrogenase%20(EC%201.2.1.3)&subsystem_name=Methylglyoxal_Metabolism) |
| Carbohydrates | Central carbohydrate metabolism | [Methylglyoxal Metabolism](https://rast.nmpdr.org/seedviewer.cgi?page=Subsystems&subsystem=Methylglyoxal_Metabolism&organism=549.408) | [Hydroxyacylglutathione hydrolase (EC 3.1.2.6)](https://rast.nmpdr.org/seedviewer.cgi?page=FunctionalRole&role=Hydroxyacylglutathione%20hydrolase%20(EC%203.1.2.6)&subsystem_name=Methylglyoxal_Metabolism) |
| Carbohydrates | Central carbohydrate metabolism | [Methylglyoxal Metabolism](https://rast.nmpdr.org/seedviewer.cgi?page=Subsystems&subsystem=Methylglyoxal_Metabolism&organism=549.408) | [Aldehyde dehydrogenase A (EC 1.2.1.22)](https://rast.nmpdr.org/seedviewer.cgi?page=FunctionalRole&role=Aldehyde%20dehydrogenase%20A%20(EC%201.2.1.22)&subsystem_name=Methylglyoxal_Metabolism) |
| Carbohydrates | Central carbohydrate metabolism | [Pyruvate metabolism II: acetyl-CoA, acetogenesis from pyruvate](https://rast.nmpdr.org/seedviewer.cgi?page=Subsystems&subsystem=Pyruvate_metabolism_II:_acetyl-CoA,_acetogenesis_from_pyruvate&organism=549.408) | [Pyruvate decarboxylase (EC 4.1.1.1)](https://rast.nmpdr.org/seedviewer.cgi?page=FunctionalRole&role=Pyruvate%20decarboxylase%20(EC%204.1.1.1)&subsystem_name=Pyruvate_metabolism_II:_acetyl-CoA,_acetogenesis_from_pyruvate) |
| Carbohydrates | Central carbohydrate metabolism | [Pyruvate metabolism II: acetyl-CoA, acetogenesis from pyruvate](https://rast.nmpdr.org/seedviewer.cgi?page=Subsystems&subsystem=Pyruvate_metabolism_II:_acetyl-CoA,_acetogenesis_from_pyruvate&organism=549.408) | [Acylphosphate phosphohydrolase (EC 3.6.1.7), putative](https://rast.nmpdr.org/seedviewer.cgi?page=FunctionalRole&role=Acylphosphate%20phosphohydrolase%20(EC%203.6.1.7),%20putative&subsystem_name=Pyruvate_metabolism_II:_acetyl-CoA,_acetogenesis_from_pyruvate) |
| Carbohydrates | Central carbohydrate metabolism | [Pyruvate metabolism II: acetyl-CoA, acetogenesis from pyruvate](https://rast.nmpdr.org/seedviewer.cgi?page=Subsystems&subsystem=Pyruvate_metabolism_II:_acetyl-CoA,_acetogenesis_from_pyruvate&organism=549.408) | [Putative membrane protein, clustering with ActP](https://rast.nmpdr.org/seedviewer.cgi?page=FunctionalRole&role=Putative%20membrane%20protein,%20clustering%20with%20ActP&subsystem_name=Pyruvate_metabolism_II:_acetyl-CoA,_acetogenesis_from_pyruvate) |
| Carbohydrates | Central carbohydrate metabolism | [Pyruvate metabolism II: acetyl-CoA, acetogenesis from pyruvate](https://rast.nmpdr.org/seedviewer.cgi?page=Subsystems&subsystem=Pyruvate_metabolism_II:_acetyl-CoA,_acetogenesis_from_pyruvate&organism=549.408) | [Pyruvate oxidase (EC 1.2.3.3)](https://rast.nmpdr.org/seedviewer.cgi?page=FunctionalRole&role=Pyruvate%20oxidase%20(EC%201.2.3.3)&subsystem_name=Pyruvate_metabolism_II:_acetyl-CoA,_acetogenesis_from_pyruvate) |
| Carbohydrates | Central carbohydrate metabolism | [Pyruvate metabolism II: acetyl-CoA, acetogenesis from pyruvate](https://rast.nmpdr.org/seedviewer.cgi?page=Subsystems&subsystem=Pyruvate_metabolism_II:_acetyl-CoA,_acetogenesis_from_pyruvate&organism=549.408) | [Aldehyde dehydrogenase (EC 1.2.1.3)](https://rast.nmpdr.org/seedviewer.cgi?page=FunctionalRole&role=Aldehyde%20dehydrogenase%20(EC%201.2.1.3)&subsystem_name=Pyruvate_metabolism_II:_acetyl-CoA,_acetogenesis_from_pyruvate) |
| Carbohydrates | Central carbohydrate metabolism | [Pyruvate metabolism II: acetyl-CoA, acetogenesis from pyruvate](https://rast.nmpdr.org/seedviewer.cgi?page=Subsystems&subsystem=Pyruvate_metabolism_II:_acetyl-CoA,_acetogenesis_from_pyruvate&organism=549.408) | [Acetate kinase (EC 2.7.2.1)](https://rast.nmpdr.org/seedviewer.cgi?page=FunctionalRole&role=Acetate%20kinase%20(EC%202.7.2.1)&subsystem_name=Pyruvate_metabolism_II:_acetyl-CoA,_acetogenesis_from_pyruvate) |
| Carbohydrates | Central carbohydrate metabolism | [Pyruvate metabolism II: acetyl-CoA, acetogenesis from pyruvate](https://rast.nmpdr.org/seedviewer.cgi?page=Subsystems&subsystem=Pyruvate_metabolism_II:_acetyl-CoA,_acetogenesis_from_pyruvate&organism=549.408) | [Acetaldehyde dehydrogenase (EC 1.2.1.10)](https://rast.nmpdr.org/seedviewer.cgi?page=FunctionalRole&role=Acetaldehyde%20dehydrogenase%20(EC%201.2.1.10)&subsystem_name=Pyruvate_metabolism_II:_acetyl-CoA,_acetogenesis_from_pyruvate) |
| Carbohydrates | Central carbohydrate metabolism | [Pyruvate metabolism II: acetyl-CoA, acetogenesis from pyruvate](https://rast.nmpdr.org/seedviewer.cgi?page=Subsystems&subsystem=Pyruvate_metabolism_II:_acetyl-CoA,_acetogenesis_from_pyruvate&organism=549.408) | [Acetate permease ActP (cation/acetate symporter)](https://rast.nmpdr.org/seedviewer.cgi?page=FunctionalRole&role=Acetate%20permease%20ActP%20(cation/acetate%20symporter)&subsystem_name=Pyruvate_metabolism_II:_acetyl-CoA,_acetogenesis_from_pyruvate) |
| Carbohydrates | Central carbohydrate metabolism | [Pyruvate metabolism II: acetyl-CoA, acetogenesis from pyruvate](https://rast.nmpdr.org/seedviewer.cgi?page=Subsystems&subsystem=Pyruvate_metabolism_II:_acetyl-CoA,_acetogenesis_from_pyruvate&organism=549.408) | [Pyruvate dehydrogenase E1 component (EC 1.2.4.1)](https://rast.nmpdr.org/seedviewer.cgi?page=FunctionalRole&role=Pyruvate%20dehydrogenase%20E1%20component%20(EC%201.2.4.1)&subsystem_name=Pyruvate_metabolism_II:_acetyl-CoA,_acetogenesis_from_pyruvate) |
| Carbohydrates | Central carbohydrate metabolism | [Pyruvate metabolism II: acetyl-CoA, acetogenesis from pyruvate](https://rast.nmpdr.org/seedviewer.cgi?page=Subsystems&subsystem=Pyruvate_metabolism_II:_acetyl-CoA,_acetogenesis_from_pyruvate&organism=549.408) | [Pyruvate oxidase [ubiquinone, cytochrome] (EC 1.2.2.2)](https://rast.nmpdr.org/seedviewer.cgi?page=FunctionalRole&role=Pyruvate%20oxidase%20%5bubiquinone,%20cytochrome%5d%20(EC%201.2.2.2)&subsystem_name=Pyruvate_metabolism_II:_acetyl-CoA,_acetogenesis_from_pyruvate) |
| Carbohydrates | Central carbohydrate metabolism | [Pyruvate metabolism II: acetyl-CoA, acetogenesis from pyruvate](https://rast.nmpdr.org/seedviewer.cgi?page=Subsystems&subsystem=Pyruvate_metabolism_II:_acetyl-CoA,_acetogenesis_from_pyruvate&organism=549.408) | [Acetyl-coenzyme A synthetase (EC 6.2.1.1)](https://rast.nmpdr.org/seedviewer.cgi?page=FunctionalRole&role=Acetyl-coenzyme%20A%20synthetase%20(EC%206.2.1.1)&subsystem_name=Pyruvate_metabolism_II:_acetyl-CoA,_acetogenesis_from_pyruvate) |
| Carbohydrates | Central carbohydrate metabolism | [Pyruvate metabolism II: acetyl-CoA, acetogenesis from pyruvate](https://rast.nmpdr.org/seedviewer.cgi?page=Subsystems&subsystem=Pyruvate_metabolism_II:_acetyl-CoA,_acetogenesis_from_pyruvate&organism=549.408) | [Protein acetyltransferase](https://rast.nmpdr.org/seedviewer.cgi?page=FunctionalRole&role=Protein%20acetyltransferase&subsystem_name=Pyruvate_metabolism_II:_acetyl-CoA,_acetogenesis_from_pyruvate) |
| Carbohydrates | Central carbohydrate metabolism | [Pyruvate metabolism II: acetyl-CoA, acetogenesis from pyruvate](https://rast.nmpdr.org/seedviewer.cgi?page=Subsystems&subsystem=Pyruvate_metabolism_II:_acetyl-CoA,_acetogenesis_from_pyruvate&organism=549.408) | [Dihydrolipoamide dehydrogenase of pyruvate dehydrogenase complex (EC 1.8.1.4)](https://rast.nmpdr.org/seedviewer.cgi?page=FunctionalRole&role=Dihydrolipoamide%20dehydrogenase%20of%20pyruvate%20dehydrogenase%20complex%20(EC%201.8.1.4)&subsystem_name=Pyruvate_metabolism_II:_acetyl-CoA,_acetogenesis_from_pyruvate) |
| Carbohydrates | Central carbohydrate metabolism | [Pyruvate metabolism II: acetyl-CoA, acetogenesis from pyruvate](https://rast.nmpdr.org/seedviewer.cgi?page=Subsystems&subsystem=Pyruvate_metabolism_II:_acetyl-CoA,_acetogenesis_from_pyruvate&organism=549.408) | [NAD-dependent protein deacetylase of SIR2 family](https://rast.nmpdr.org/seedviewer.cgi?page=FunctionalRole&role=NAD-dependent%20protein%20deacetylase%20of%20SIR2%20family&subsystem_name=Pyruvate_metabolism_II:_acetyl-CoA,_acetogenesis_from_pyruvate) |
| Carbohydrates | Central carbohydrate metabolism | [Pyruvate metabolism II: acetyl-CoA, acetogenesis from pyruvate](https://rast.nmpdr.org/seedviewer.cgi?page=Subsystems&subsystem=Pyruvate_metabolism_II:_acetyl-CoA,_acetogenesis_from_pyruvate&organism=549.408) | [Dihydrolipoamide acetyltransferase component of pyruvate dehydrogenase complex (EC 2.3.1.12)](https://rast.nmpdr.org/seedviewer.cgi?page=FunctionalRole&role=Dihydrolipoamide%20acetyltransferase%20component%20of%20pyruvate%20dehydrogenase%20complex%20(EC%202.3.1.12)&subsystem_name=Pyruvate_metabolism_II:_acetyl-CoA,_acetogenesis_from_pyruvate) |
| Carbohydrates | Central carbohydrate metabolism | [Pyruvate metabolism II: acetyl-CoA, acetogenesis from pyruvate](https://rast.nmpdr.org/seedviewer.cgi?page=Subsystems&subsystem=Pyruvate_metabolism_II:_acetyl-CoA,_acetogenesis_from_pyruvate&organism=549.408) | [Phosphate acetyltransferase (EC 2.3.1.8)](https://rast.nmpdr.org/seedviewer.cgi?page=FunctionalRole&role=Phosphate%20acetyltransferase%20(EC%202.3.1.8)&subsystem_name=Pyruvate_metabolism_II:_acetyl-CoA,_acetogenesis_from_pyruvate) |
| Carbohydrates | Central carbohydrate metabolism | [Pyruvate Alanine Serine Interconversions](https://rast.nmpdr.org/seedviewer.cgi?page=Subsystems&subsystem=Pyruvate_Alanine_Serine_Interconversions&organism=549.408) | [D-serine/D-alanine/glycine transporter](https://rast.nmpdr.org/seedviewer.cgi?page=FunctionalRole&role=D-serine/D-alanine/glycine%20transporter&subsystem_name=Pyruvate_Alanine_Serine_Interconversions) |
| Carbohydrates | Central carbohydrate metabolism | [Pyruvate Alanine Serine Interconversions](https://rast.nmpdr.org/seedviewer.cgi?page=Subsystems&subsystem=Pyruvate_Alanine_Serine_Interconversions&organism=549.408) | [Branched-chain amino acid aminotransferase (EC 2.6.1.42)](https://rast.nmpdr.org/seedviewer.cgi?page=FunctionalRole&role=Branched-chain%20amino%20acid%20aminotransferase%20(EC%202.6.1.42)&subsystem_name=Pyruvate_Alanine_Serine_Interconversions) |
| Carbohydrates | Central carbohydrate metabolism | [Pyruvate Alanine Serine Interconversions](https://rast.nmpdr.org/seedviewer.cgi?page=Subsystems&subsystem=Pyruvate_Alanine_Serine_Interconversions&organism=549.408) | [Valine--pyruvate aminotransferase (EC 2.6.1.66)](https://rast.nmpdr.org/seedviewer.cgi?page=FunctionalRole&role=Valine--pyruvate%20aminotransferase%20(EC%202.6.1.66)&subsystem_name=Pyruvate_Alanine_Serine_Interconversions) |
| Carbohydrates | Central carbohydrate metabolism | [Pyruvate Alanine Serine Interconversions](https://rast.nmpdr.org/seedviewer.cgi?page=Subsystems&subsystem=Pyruvate_Alanine_Serine_Interconversions&organism=549.408) | [D-amino acid dehydrogenase small subunit (EC 1.4.99.1)](https://rast.nmpdr.org/seedviewer.cgi?page=FunctionalRole&role=D-amino%20acid%20dehydrogenase%20small%20subunit%20(EC%201.4.99.1)&subsystem_name=Pyruvate_Alanine_Serine_Interconversions) |
| Carbohydrates | Central carbohydrate metabolism | [Pyruvate Alanine Serine Interconversions](https://rast.nmpdr.org/seedviewer.cgi?page=Subsystems&subsystem=Pyruvate_Alanine_Serine_Interconversions&organism=549.408) | [Serine transporter](https://rast.nmpdr.org/seedviewer.cgi?page=FunctionalRole&role=Serine%20transporter&subsystem_name=Pyruvate_Alanine_Serine_Interconversions) |
| Carbohydrates | Central carbohydrate metabolism | [Pyruvate Alanine Serine Interconversions](https://rast.nmpdr.org/seedviewer.cgi?page=Subsystems&subsystem=Pyruvate_Alanine_Serine_Interconversions&organism=549.408) | [L-alanine:glyoxylate aminotransferase (EC 2.6.1.44)](https://rast.nmpdr.org/seedviewer.cgi?page=FunctionalRole&role=L-alanine:glyoxylate%20aminotransferase%20(EC%202.6.1.44)&subsystem_name=Pyruvate_Alanine_Serine_Interconversions) |
| Carbohydrates | Central carbohydrate metabolism | [Pyruvate Alanine Serine Interconversions](https://rast.nmpdr.org/seedviewer.cgi?page=Subsystems&subsystem=Pyruvate_Alanine_Serine_Interconversions&organism=549.408) | [Alanine racemase, catabolic (EC 5.1.1.1)](https://rast.nmpdr.org/seedviewer.cgi?page=FunctionalRole&role=Alanine%20racemase,%20catabolic%20(EC%205.1.1.1)&subsystem_name=Pyruvate_Alanine_Serine_Interconversions) |
| Carbohydrates | Central carbohydrate metabolism | [Pyruvate Alanine Serine Interconversions](https://rast.nmpdr.org/seedviewer.cgi?page=Subsystems&subsystem=Pyruvate_Alanine_Serine_Interconversions&organism=549.408) | [L-serine dehydratase (EC 4.3.1.17)](https://rast.nmpdr.org/seedviewer.cgi?page=FunctionalRole&role=L-serine%20dehydratase%20(EC%204.3.1.17)&subsystem_name=Pyruvate_Alanine_Serine_Interconversions) |
| Carbohydrates | Central carbohydrate metabolism | [Glyoxylate bypass](https://rast.nmpdr.org/seedviewer.cgi?page=Subsystems&subsystem=Glyoxylate_bypass&organism=549.408) | [Malate dehydrogenase (EC 1.1.1.37)](https://rast.nmpdr.org/seedviewer.cgi?page=FunctionalRole&role=Malate%20dehydrogenase%20(EC%201.1.1.37)&subsystem_name=Glyoxylate_bypass) |
| Carbohydrates | Central carbohydrate metabolism | [Glyoxylate bypass](https://rast.nmpdr.org/seedviewer.cgi?page=Subsystems&subsystem=Glyoxylate_bypass&organism=549.408) | [Citrate synthase (si) (EC 2.3.3.1)](https://rast.nmpdr.org/seedviewer.cgi?page=FunctionalRole&role=Citrate%20synthase%20(si)%20(EC%202.3.3.1)&subsystem_name=Glyoxylate_bypass) |
| Carbohydrates | Central carbohydrate metabolism | [Glyoxylate bypass](https://rast.nmpdr.org/seedviewer.cgi?page=Subsystems&subsystem=Glyoxylate_bypass&organism=549.408) | [Aconitate hydratase 2 (EC 4.2.1.3)](https://rast.nmpdr.org/seedviewer.cgi?page=FunctionalRole&role=Aconitate%20hydratase%202%20(EC%204.2.1.3)&subsystem_name=Glyoxylate_bypass) |
| Carbohydrates | Central carbohydrate metabolism | [Glyoxylate bypass](https://rast.nmpdr.org/seedviewer.cgi?page=Subsystems&subsystem=Glyoxylate_bypass&organism=549.408) | [Isocitrate lyase (EC 4.1.3.1)](https://rast.nmpdr.org/seedviewer.cgi?page=FunctionalRole&role=Isocitrate%20lyase%20(EC%204.1.3.1)&subsystem_name=Glyoxylate_bypass) |
| Carbohydrates | Central carbohydrate metabolism | [Glyoxylate bypass](https://rast.nmpdr.org/seedviewer.cgi?page=Subsystems&subsystem=Glyoxylate_bypass&organism=549.408) | [Malate synthase (EC 2.3.3.9)](https://rast.nmpdr.org/seedviewer.cgi?page=FunctionalRole&role=Malate%20synthase%20(EC%202.3.3.9)&subsystem_name=Glyoxylate_bypass) |
| Carbohydrates | Central carbohydrate metabolism | [Glyoxylate bypass](https://rast.nmpdr.org/seedviewer.cgi?page=Subsystems&subsystem=Glyoxylate_bypass&organism=549.408) | [Isocitrate dehydrogenase phosphatase (EC 2.7.11.5)/kinase (EC 3.1.3.-)](https://rast.nmpdr.org/seedviewer.cgi?page=FunctionalRole&role=Isocitrate%20dehydrogenase%20phosphatase%20(EC%202.7.11.5)/kinase%20(EC%203.1.3.-)&subsystem_name=Glyoxylate_bypass) |
| Carbohydrates | Central carbohydrate metabolism | [Glyoxylate bypass](https://rast.nmpdr.org/seedviewer.cgi?page=Subsystems&subsystem=Glyoxylate_bypass&organism=549.408) | [Aconitate hydratase (EC 4.2.1.3)](https://rast.nmpdr.org/seedviewer.cgi?page=FunctionalRole&role=Aconitate%20hydratase%20(EC%204.2.1.3)&subsystem_name=Glyoxylate_bypass) |
| Carbohydrates | Central carbohydrate metabolism | [Dihydroxyacetone kinases](https://rast.nmpdr.org/seedviewer.cgi?page=Subsystems&subsystem=Dihydroxyacetone_kinases&organism=549.408) | [Dihydroxyacetone kinase, ATP-dependent (EC 2.7.1.29)](https://rast.nmpdr.org/seedviewer.cgi?page=FunctionalRole&role=Dihydroxyacetone%20kinase,%20ATP-dependent%20(EC%202.7.1.29)&subsystem_name=Dihydroxyacetone_kinases) |
| Carbohydrates | Central carbohydrate metabolism | [Glycolysis and Gluconeogenesis](https://rast.nmpdr.org/seedviewer.cgi?page=Subsystems&subsystem=Glycolysis_and_Gluconeogenesis&organism=549.408) | [6-phosphofructokinase (EC 2.7.1.11)](https://rast.nmpdr.org/seedviewer.cgi?page=FunctionalRole&role=6-phosphofructokinase%20(EC%202.7.1.11)&subsystem_name=Glycolysis_and_Gluconeogenesis) |
| Carbohydrates | Central carbohydrate metabolism | [Glycolysis and Gluconeogenesis](https://rast.nmpdr.org/seedviewer.cgi?page=Subsystems&subsystem=Glycolysis_and_Gluconeogenesis&organism=549.408) | [Triosephosphate isomerase (EC 5.3.1.1)](https://rast.nmpdr.org/seedviewer.cgi?page=FunctionalRole&role=Triosephosphate%20isomerase%20(EC%205.3.1.1)&subsystem_name=Glycolysis_and_Gluconeogenesis) |
| Carbohydrates | Central carbohydrate metabolism | [Glycolysis and Gluconeogenesis](https://rast.nmpdr.org/seedviewer.cgi?page=Subsystems&subsystem=Glycolysis_and_Gluconeogenesis&organism=549.408) | [Fructose-1,6-bisphosphatase, GlpX type (EC 3.1.3.11)](https://rast.nmpdr.org/seedviewer.cgi?page=FunctionalRole&role=Fructose-1,6-bisphosphatase,%20GlpX%20type%20(EC%203.1.3.11)&subsystem_name=Glycolysis_and_Gluconeogenesis) |
| Carbohydrates | Central carbohydrate metabolism | [Glycolysis and Gluconeogenesis](https://rast.nmpdr.org/seedviewer.cgi?page=Subsystems&subsystem=Glycolysis_and_Gluconeogenesis&organism=549.408) | [Fructose-bisphosphate aldolase class II (EC 4.1.2.13)](https://rast.nmpdr.org/seedviewer.cgi?page=FunctionalRole&role=Fructose-bisphosphate%20aldolase%20class%20II%20(EC%204.1.2.13)&subsystem_name=Glycolysis_and_Gluconeogenesis) |
| Carbohydrates | Central carbohydrate metabolism | [Glycolysis and Gluconeogenesis](https://rast.nmpdr.org/seedviewer.cgi?page=Subsystems&subsystem=Glycolysis_and_Gluconeogenesis&organism=549.408) | [Fructose-1,6-bisphosphatase, type I (EC 3.1.3.11)](https://rast.nmpdr.org/seedviewer.cgi?page=FunctionalRole&role=Fructose-1,6-bisphosphatase,%20type%20I%20(EC%203.1.3.11)&subsystem_name=Glycolysis_and_Gluconeogenesis) |
| Carbohydrates | Central carbohydrate metabolism | [Glycolysis and Gluconeogenesis](https://rast.nmpdr.org/seedviewer.cgi?page=Subsystems&subsystem=Glycolysis_and_Gluconeogenesis&organism=549.408) | [Glucose-6-phosphate isomerase (EC 5.3.1.9)](https://rast.nmpdr.org/seedviewer.cgi?page=FunctionalRole&role=Glucose-6-phosphate%20isomerase%20(EC%205.3.1.9)&subsystem_name=Glycolysis_and_Gluconeogenesis) |
| Carbohydrates | Central carbohydrate metabolism | [Glycolysis and Gluconeogenesis](https://rast.nmpdr.org/seedviewer.cgi?page=Subsystems&subsystem=Glycolysis_and_Gluconeogenesis&organism=549.408) | [Phosphoglycerate mutase (EC 5.4.2.1)](https://rast.nmpdr.org/seedviewer.cgi?page=FunctionalRole&role=Phosphoglycerate%20mutase%20(EC%205.4.2.1)&subsystem_name=Glycolysis_and_Gluconeogenesis) |
| Carbohydrates | Central carbohydrate metabolism | [Glycolysis and Gluconeogenesis](https://rast.nmpdr.org/seedviewer.cgi?page=Subsystems&subsystem=Glycolysis_and_Gluconeogenesis&organism=549.408) | [Glucokinase (EC 2.7.1.2)](https://rast.nmpdr.org/seedviewer.cgi?page=FunctionalRole&role=Glucokinase%20(EC%202.7.1.2)&subsystem_name=Glycolysis_and_Gluconeogenesis) |
| Carbohydrates | Central carbohydrate metabolism | [Glycolysis and Gluconeogenesis](https://rast.nmpdr.org/seedviewer.cgi?page=Subsystems&subsystem=Glycolysis_and_Gluconeogenesis&organism=549.408) | [Pyruvate kinase (EC 2.7.1.40)](https://rast.nmpdr.org/seedviewer.cgi?page=FunctionalRole&role=Pyruvate%20kinase%20(EC%202.7.1.40)&subsystem_name=Glycolysis_and_Gluconeogenesis) |
| Carbohydrates | Central carbohydrate metabolism | [Glycolysis and Gluconeogenesis](https://rast.nmpdr.org/seedviewer.cgi?page=Subsystems&subsystem=Glycolysis_and_Gluconeogenesis&organism=549.408) | [Phosphoenolpyruvate synthase (EC 2.7.9.2)](https://rast.nmpdr.org/seedviewer.cgi?page=FunctionalRole&role=Phosphoenolpyruvate%20synthase%20(EC%202.7.9.2)&subsystem_name=Glycolysis_and_Gluconeogenesis) |
| Carbohydrates | Central carbohydrate metabolism | [Glycolysis and Gluconeogenesis](https://rast.nmpdr.org/seedviewer.cgi?page=Subsystems&subsystem=Glycolysis_and_Gluconeogenesis&organism=549.408) | [Phosphoglycerate kinase (EC 2.7.2.3)](https://rast.nmpdr.org/seedviewer.cgi?page=FunctionalRole&role=Phosphoglycerate%20kinase%20(EC%202.7.2.3)&subsystem_name=Glycolysis_and_Gluconeogenesis) |
| Carbohydrates | Central carbohydrate metabolism | [Glycolysis and Gluconeogenesis](https://rast.nmpdr.org/seedviewer.cgi?page=Subsystems&subsystem=Glycolysis_and_Gluconeogenesis&organism=549.408) | [Enolase (EC 4.2.1.11)](https://rast.nmpdr.org/seedviewer.cgi?page=FunctionalRole&role=Enolase%20(EC%204.2.1.11)&subsystem_name=Glycolysis_and_Gluconeogenesis) |
| Carbohydrates | Central carbohydrate metabolism | [Glycolysis and Gluconeogenesis](https://rast.nmpdr.org/seedviewer.cgi?page=Subsystems&subsystem=Glycolysis_and_Gluconeogenesis&organism=549.408) | [NAD-dependent glyceraldehyde-3-phosphate dehydrogenase (EC 1.2.1.12)](https://rast.nmpdr.org/seedviewer.cgi?page=FunctionalRole&role=NAD-dependent%20glyceraldehyde-3-phosphate%20dehydrogenase%20(EC%201.2.1.12)&subsystem_name=Glycolysis_and_Gluconeogenesis) |
| Carbohydrates | Central carbohydrate metabolism | [Entner-Doudoroff Pathway](https://rast.nmpdr.org/seedviewer.cgi?page=Subsystems&subsystem=Entner-Doudoroff_Pathway&organism=549.408) | [Glucose dehydrogenase, PQQ-dependent (EC 1.1.5.2)](https://rast.nmpdr.org/seedviewer.cgi?page=FunctionalRole&role=Glucose%20dehydrogenase,%20PQQ-dependent%20(EC%201.1.5.2)&subsystem_name=Entner-Doudoroff_Pathway) |
| Carbohydrates | Central carbohydrate metabolism | [Entner-Doudoroff Pathway](https://rast.nmpdr.org/seedviewer.cgi?page=Subsystems&subsystem=Entner-Doudoroff_Pathway&organism=549.408) | [2-dehydro-3-deoxygluconate kinase (EC 2.7.1.45)](https://rast.nmpdr.org/seedviewer.cgi?page=FunctionalRole&role=2-dehydro-3-deoxygluconate%20kinase%20(EC%202.7.1.45)&subsystem_name=Entner-Doudoroff_Pathway) |
| Carbohydrates | Central carbohydrate metabolism | [Entner-Doudoroff Pathway](https://rast.nmpdr.org/seedviewer.cgi?page=Subsystems&subsystem=Entner-Doudoroff_Pathway&organism=549.408) | [Glucose-6-phosphate 1-dehydrogenase (EC 1.1.1.49)](https://rast.nmpdr.org/seedviewer.cgi?page=FunctionalRole&role=Glucose-6-phosphate%201-dehydrogenase%20(EC%201.1.1.49)&subsystem_name=Entner-Doudoroff_Pathway) |
| Carbohydrates | Central carbohydrate metabolism | [Entner-Doudoroff Pathway](https://rast.nmpdr.org/seedviewer.cgi?page=Subsystems&subsystem=Entner-Doudoroff_Pathway&organism=549.408) | [2-dehydro-3-deoxyphosphogluconate aldolase (EC 4.1.2.14)](https://rast.nmpdr.org/seedviewer.cgi?page=FunctionalRole&role=2-dehydro-3-deoxyphosphogluconate%20aldolase%20(EC%204.1.2.14)&subsystem_name=Entner-Doudoroff_Pathway) |
| Carbohydrates | Central carbohydrate metabolism | [Entner-Doudoroff Pathway](https://rast.nmpdr.org/seedviewer.cgi?page=Subsystems&subsystem=Entner-Doudoroff_Pathway&organism=549.408) | [Glucose 1-dehydrogenase (EC 1.1.1.47)](https://rast.nmpdr.org/seedviewer.cgi?page=FunctionalRole&role=Glucose%201-dehydrogenase%20(EC%201.1.1.47)&subsystem_name=Entner-Doudoroff_Pathway) |
| Carbohydrates | Central carbohydrate metabolism | [Entner-Doudoroff Pathway](https://rast.nmpdr.org/seedviewer.cgi?page=Subsystems&subsystem=Entner-Doudoroff_Pathway&organism=549.408) | [6-phosphogluconolactonase (EC 3.1.1.31)](https://rast.nmpdr.org/seedviewer.cgi?page=FunctionalRole&role=6-phosphogluconolactonase%20(EC%203.1.1.31)&subsystem_name=Entner-Doudoroff_Pathway) |
| Carbohydrates | Central carbohydrate metabolism | [Entner-Doudoroff Pathway](https://rast.nmpdr.org/seedviewer.cgi?page=Subsystems&subsystem=Entner-Doudoroff_Pathway&organism=549.408) | [Gluconokinase (EC 2.7.1.12)](https://rast.nmpdr.org/seedviewer.cgi?page=FunctionalRole&role=Gluconokinase%20(EC%202.7.1.12)&subsystem_name=Entner-Doudoroff_Pathway) |
| Carbohydrates | Central carbohydrate metabolism | [Entner-Doudoroff Pathway](https://rast.nmpdr.org/seedviewer.cgi?page=Subsystems&subsystem=Entner-Doudoroff_Pathway&organism=549.408) | [Phosphoglycerate mutase (EC 5.4.2.1)](https://rast.nmpdr.org/seedviewer.cgi?page=FunctionalRole&role=Phosphoglycerate%20mutase%20(EC%205.4.2.1)&subsystem_name=Entner-Doudoroff_Pathway) |
| Carbohydrates | Central carbohydrate metabolism | [Entner-Doudoroff Pathway](https://rast.nmpdr.org/seedviewer.cgi?page=Subsystems&subsystem=Entner-Doudoroff_Pathway&organism=549.408) | [Glucokinase (EC 2.7.1.2)](https://rast.nmpdr.org/seedviewer.cgi?page=FunctionalRole&role=Glucokinase%20(EC%202.7.1.2)&subsystem_name=Entner-Doudoroff_Pathway) |
| Carbohydrates | Central carbohydrate metabolism | [Entner-Doudoroff Pathway](https://rast.nmpdr.org/seedviewer.cgi?page=Subsystems&subsystem=Entner-Doudoroff_Pathway&organism=549.408) | [Pyruvate kinase (EC 2.7.1.40)](https://rast.nmpdr.org/seedviewer.cgi?page=FunctionalRole&role=Pyruvate%20kinase%20(EC%202.7.1.40)&subsystem_name=Entner-Doudoroff_Pathway) |
| Carbohydrates | Central carbohydrate metabolism | [Entner-Doudoroff Pathway](https://rast.nmpdr.org/seedviewer.cgi?page=Subsystems&subsystem=Entner-Doudoroff_Pathway&organism=549.408) | [Phosphoglycerate kinase (EC 2.7.2.3)](https://rast.nmpdr.org/seedviewer.cgi?page=FunctionalRole&role=Phosphoglycerate%20kinase%20(EC%202.7.2.3)&subsystem_name=Entner-Doudoroff_Pathway) |
| Carbohydrates | Central carbohydrate metabolism | [Entner-Doudoroff Pathway](https://rast.nmpdr.org/seedviewer.cgi?page=Subsystems&subsystem=Entner-Doudoroff_Pathway&organism=549.408) | [NAD-dependent glyceraldehyde-3-phosphate dehydrogenase (EC 1.2.1.12)](https://rast.nmpdr.org/seedviewer.cgi?page=FunctionalRole&role=NAD-dependent%20glyceraldehyde-3-phosphate%20dehydrogenase%20(EC%201.2.1.12)&subsystem_name=Entner-Doudoroff_Pathway) |
| Carbohydrates | Central carbohydrate metabolism | [Entner-Doudoroff Pathway](https://rast.nmpdr.org/seedviewer.cgi?page=Subsystems&subsystem=Entner-Doudoroff_Pathway&organism=549.408) | [Enolase (EC 4.2.1.11)](https://rast.nmpdr.org/seedviewer.cgi?page=FunctionalRole&role=Enolase%20(EC%204.2.1.11)&subsystem_name=Entner-Doudoroff_Pathway) |
| Carbohydrates | Central carbohydrate metabolism | [Dehydrogenase complexes](https://rast.nmpdr.org/seedviewer.cgi?page=Subsystems&subsystem=Dehydrogenase_complexes&organism=549.408) | [Dihydrolipoamide succinyltransferase component (E2) of 2-oxoglutarate dehydrogenase complex (EC 2.3.1.61)](https://rast.nmpdr.org/seedviewer.cgi?page=FunctionalRole&role=Dihydrolipoamide%20succinyltransferase%20component%20(E2)%20of%202-oxoglutarate%20dehydrogenase%20complex%20(EC%202.3.1.61)&subsystem_name=Dehydrogenase_complexes) |
| Carbohydrates | Central carbohydrate metabolism | [Dehydrogenase complexes](https://rast.nmpdr.org/seedviewer.cgi?page=Subsystems&subsystem=Dehydrogenase_complexes&organism=549.408) | [Pyruvate dehydrogenase E1 component (EC 1.2.4.1)](https://rast.nmpdr.org/seedviewer.cgi?page=FunctionalRole&role=Pyruvate%20dehydrogenase%20E1%20component%20(EC%201.2.4.1)&subsystem_name=Dehydrogenase_complexes) |
| Carbohydrates | Central carbohydrate metabolism | [Dehydrogenase complexes](https://rast.nmpdr.org/seedviewer.cgi?page=Subsystems&subsystem=Dehydrogenase_complexes&organism=549.408) | [Dihydrolipoamide acetyltransferase component of pyruvate dehydrogenase complex (EC 2.3.1.12)](https://rast.nmpdr.org/seedviewer.cgi?page=FunctionalRole&role=Dihydrolipoamide%20acetyltransferase%20component%20of%20pyruvate%20dehydrogenase%20complex%20(EC%202.3.1.12)&subsystem_name=Dehydrogenase_complexes) |
| Carbohydrates | Central carbohydrate metabolism | [Dehydrogenase complexes](https://rast.nmpdr.org/seedviewer.cgi?page=Subsystems&subsystem=Dehydrogenase_complexes&organism=549.408) | [2-oxoglutarate dehydrogenase E1 component (EC 1.2.4.2)](https://rast.nmpdr.org/seedviewer.cgi?page=FunctionalRole&role=2-oxoglutarate%20dehydrogenase%20E1%20component%20(EC%201.2.4.2)&subsystem_name=Dehydrogenase_complexes) |
| Carbohydrates | Central carbohydrate metabolism | [Dehydrogenase complexes](https://rast.nmpdr.org/seedviewer.cgi?page=Subsystems&subsystem=Dehydrogenase_complexes&organism=549.408) | [Cytosol aminopeptidase PepA (EC 3.4.11.1)](https://rast.nmpdr.org/seedviewer.cgi?page=FunctionalRole&role=Cytosol%20aminopeptidase%20PepA%20(EC%203.4.11.1)&subsystem_name=Dehydrogenase_complexes) |
| Carbohydrates | Central carbohydrate metabolism | [TCA Cycle](https://rast.nmpdr.org/seedviewer.cgi?page=Subsystems&subsystem=TCA_Cycle&organism=549.408) | [Malate dehydrogenase (EC 1.1.1.37)](https://rast.nmpdr.org/seedviewer.cgi?page=FunctionalRole&role=Malate%20dehydrogenase%20(EC%201.1.1.37)&subsystem_name=TCA_Cycle) |
| Carbohydrates | Central carbohydrate metabolism | [TCA Cycle](https://rast.nmpdr.org/seedviewer.cgi?page=Subsystems&subsystem=TCA_Cycle&organism=549.408) | [Succinate dehydrogenase flavoprotein subunit (EC 1.3.99.1)](https://rast.nmpdr.org/seedviewer.cgi?page=FunctionalRole&role=Succinate%20dehydrogenase%20flavoprotein%20subunit%20(EC%201.3.99.1)&subsystem_name=TCA_Cycle) |
| Carbohydrates | Central carbohydrate metabolism | [TCA Cycle](https://rast.nmpdr.org/seedviewer.cgi?page=Subsystems&subsystem=TCA_Cycle&organism=549.408) | [Aconitate hydratase (EC 4.2.1.3)](https://rast.nmpdr.org/seedviewer.cgi?page=FunctionalRole&role=Aconitate%20hydratase%20(EC%204.2.1.3)&subsystem_name=TCA_Cycle) |
| Carbohydrates | Central carbohydrate metabolism | [TCA Cycle](https://rast.nmpdr.org/seedviewer.cgi?page=Subsystems&subsystem=TCA_Cycle&organism=549.408) | [Succinate dehydrogenase iron-sulfur protein (EC 1.3.99.1)](https://rast.nmpdr.org/seedviewer.cgi?page=FunctionalRole&role=Succinate%20dehydrogenase%20iron-sulfur%20protein%20(EC%201.3.99.1)&subsystem_name=TCA_Cycle) |
| Carbohydrates | Central carbohydrate metabolism | [TCA Cycle](https://rast.nmpdr.org/seedviewer.cgi?page=Subsystems&subsystem=TCA_Cycle&organism=549.408) | [Aconitate hydratase 2 (EC 4.2.1.3)](https://rast.nmpdr.org/seedviewer.cgi?page=FunctionalRole&role=Aconitate%20hydratase%202%20(EC%204.2.1.3)&subsystem_name=TCA_Cycle) |
| Carbohydrates | Central carbohydrate metabolism | [TCA Cycle](https://rast.nmpdr.org/seedviewer.cgi?page=Subsystems&subsystem=TCA_Cycle&organism=549.408) | [Citrate synthase (si) (EC 2.3.3.1)](https://rast.nmpdr.org/seedviewer.cgi?page=FunctionalRole&role=Citrate%20synthase%20(si)%20(EC%202.3.3.1)&subsystem_name=TCA_Cycle) |
| Carbohydrates | Central carbohydrate metabolism | [TCA Cycle](https://rast.nmpdr.org/seedviewer.cgi?page=Subsystems&subsystem=TCA_Cycle&organism=549.408) | [Transcriptional regulator of succinyl CoA synthetase operon](https://rast.nmpdr.org/seedviewer.cgi?page=FunctionalRole&role=Transcriptional%20regulator%20of%20succinyl%20CoA%20synthetase%20operon&subsystem_name=TCA_Cycle) |
| Carbohydrates | Central carbohydrate metabolism | [TCA Cycle](https://rast.nmpdr.org/seedviewer.cgi?page=Subsystems&subsystem=TCA_Cycle&organism=549.408) | [Malate:quinone oxidoreductase (EC 1.1.5.4)](https://rast.nmpdr.org/seedviewer.cgi?page=FunctionalRole&role=Malate:quinone%20oxidoreductase%20(EC%201.1.5.4)&subsystem_name=TCA_Cycle) |
| Carbohydrates | Central carbohydrate metabolism | [TCA Cycle](https://rast.nmpdr.org/seedviewer.cgi?page=Subsystems&subsystem=TCA_Cycle&organism=549.408) | [Dihydrolipoamide dehydrogenase (EC 1.8.1.4)](https://rast.nmpdr.org/seedviewer.cgi?page=FunctionalRole&role=Dihydrolipoamide%20dehydrogenase%20(EC%201.8.1.4)&subsystem_name=TCA_Cycle) |
| Carbohydrates | Central carbohydrate metabolism | [TCA Cycle](https://rast.nmpdr.org/seedviewer.cgi?page=Subsystems&subsystem=TCA_Cycle&organism=549.408) | [Fumarate hydratase class II (EC 4.2.1.2)](https://rast.nmpdr.org/seedviewer.cgi?page=FunctionalRole&role=Fumarate%20hydratase%20class%20II%20(EC%204.2.1.2)&subsystem_name=TCA_Cycle) |
| Carbohydrates | Central carbohydrate metabolism | [TCA Cycle](https://rast.nmpdr.org/seedviewer.cgi?page=Subsystems&subsystem=TCA_Cycle&organism=549.408) | [Isocitrate dehydrogenase [NADP] (EC 1.1.1.42)](https://rast.nmpdr.org/seedviewer.cgi?page=FunctionalRole&role=Isocitrate%20dehydrogenase%20%5bNADP%5d%20(EC%201.1.1.42)&subsystem_name=TCA_Cycle) |
| Carbohydrates | Central carbohydrate metabolism | [TCA Cycle](https://rast.nmpdr.org/seedviewer.cgi?page=Subsystems&subsystem=TCA_Cycle&organism=549.408) | [Dihydrolipoamide dehydrogenase of pyruvate dehydrogenase complex (EC 1.8.1.4)](https://rast.nmpdr.org/seedviewer.cgi?page=FunctionalRole&role=Dihydrolipoamide%20dehydrogenase%20of%20pyruvate%20dehydrogenase%20complex%20(EC%201.8.1.4)&subsystem_name=TCA_Cycle) |
| Carbohydrates | Central carbohydrate metabolism | [TCA Cycle](https://rast.nmpdr.org/seedviewer.cgi?page=Subsystems&subsystem=TCA_Cycle&organism=549.408) | [Succinyl-CoA ligase [ADP-forming] alpha chain (EC 6.2.1.5)](https://rast.nmpdr.org/seedviewer.cgi?page=FunctionalRole&role=Succinyl-CoA%20ligase%20%5bADP-forming%5d%20alpha%20chain%20(EC%206.2.1.5)&subsystem_name=TCA_Cycle) |
| Carbohydrates | Central carbohydrate metabolism | [TCA Cycle](https://rast.nmpdr.org/seedviewer.cgi?page=Subsystems&subsystem=TCA_Cycle&organism=549.408) | [Dihydrolipoamide succinyltransferase component (E2) of 2-oxoglutarate dehydrogenase complex (EC 2.3.1.61)](https://rast.nmpdr.org/seedviewer.cgi?page=FunctionalRole&role=Dihydrolipoamide%20succinyltransferase%20component%20(E2)%20of%202-oxoglutarate%20dehydrogenase%20complex%20(EC%202.3.1.61)&subsystem_name=TCA_Cycle) |
| Carbohydrates | Central carbohydrate metabolism | [TCA Cycle](https://rast.nmpdr.org/seedviewer.cgi?page=Subsystems&subsystem=TCA_Cycle&organism=549.408) | [Fumarate hydratase class I, aerobic (EC 4.2.1.2)](https://rast.nmpdr.org/seedviewer.cgi?page=FunctionalRole&role=Fumarate%20hydratase%20class%20I,%20aerobic%20(EC%204.2.1.2)&subsystem_name=TCA_Cycle) |
| Carbohydrates | Central carbohydrate metabolism | [TCA Cycle](https://rast.nmpdr.org/seedviewer.cgi?page=Subsystems&subsystem=TCA_Cycle&organism=549.408) | [2-oxoglutarate dehydrogenase E1 component (EC 1.2.4.2)](https://rast.nmpdr.org/seedviewer.cgi?page=FunctionalRole&role=2-oxoglutarate%20dehydrogenase%20E1%20component%20(EC%201.2.4.2)&subsystem_name=TCA_Cycle) |
| Carbohydrates | Central carbohydrate metabolism | [TCA Cycle](https://rast.nmpdr.org/seedviewer.cgi?page=Subsystems&subsystem=TCA_Cycle&organism=549.408) | [Succinyl-CoA ligase [ADP-forming] beta chain (EC 6.2.1.5)](https://rast.nmpdr.org/seedviewer.cgi?page=FunctionalRole&role=Succinyl-CoA%20ligase%20%5bADP-forming%5d%20beta%20chain%20(EC%206.2.1.5)&subsystem_name=TCA_Cycle) |
| Carbohydrates | Central carbohydrate metabolism | [Pyruvate metabolism I: anaplerotic reactions, PEP](https://rast.nmpdr.org/seedviewer.cgi?page=Subsystems&subsystem=Pyruvate_metabolism_I:_anaplerotic_reactions,_PEP&organism=549.408) | [D-malic enzyme (EC 1.1.1.83)](https://rast.nmpdr.org/seedviewer.cgi?page=FunctionalRole&role=D-malic%20enzyme%20(EC%201.1.1.83)&subsystem_name=Pyruvate_metabolism_I:_anaplerotic_reactions,_PEP) |
| Carbohydrates | Central carbohydrate metabolism | [Pyruvate metabolism I: anaplerotic reactions, PEP](https://rast.nmpdr.org/seedviewer.cgi?page=Subsystems&subsystem=Pyruvate_metabolism_I:_anaplerotic_reactions,_PEP&organism=549.408) | [Phosphoenolpyruvate carboxykinase [ATP] (EC 4.1.1.49)](https://rast.nmpdr.org/seedviewer.cgi?page=FunctionalRole&role=Phosphoenolpyruvate%20carboxykinase%20%5bATP%5d%20(EC%204.1.1.49)&subsystem_name=Pyruvate_metabolism_I:_anaplerotic_reactions,_PEP) |
| Carbohydrates | Central carbohydrate metabolism | [Pyruvate metabolism I: anaplerotic reactions, PEP](https://rast.nmpdr.org/seedviewer.cgi?page=Subsystems&subsystem=Pyruvate_metabolism_I:_anaplerotic_reactions,_PEP&organism=549.408) | [NAD-dependent malic enzyme (EC 1.1.1.38)](https://rast.nmpdr.org/seedviewer.cgi?page=FunctionalRole&role=NAD-dependent%20malic%20enzyme%20(EC%201.1.1.38)&subsystem_name=Pyruvate_metabolism_I:_anaplerotic_reactions,_PEP) |
| Carbohydrates | Central carbohydrate metabolism | [Pyruvate metabolism I: anaplerotic reactions, PEP](https://rast.nmpdr.org/seedviewer.cgi?page=Subsystems&subsystem=Pyruvate_metabolism_I:_anaplerotic_reactions,_PEP&organism=549.408) | [Phosphoenolpyruvate carboxylase (EC 4.1.1.31)](https://rast.nmpdr.org/seedviewer.cgi?page=FunctionalRole&role=Phosphoenolpyruvate%20carboxylase%20(EC%204.1.1.31)&subsystem_name=Pyruvate_metabolism_I:_anaplerotic_reactions,_PEP) |
| Carbohydrates | Central carbohydrate metabolism | [Pyruvate metabolism I: anaplerotic reactions, PEP](https://rast.nmpdr.org/seedviewer.cgi?page=Subsystems&subsystem=Pyruvate_metabolism_I:_anaplerotic_reactions,_PEP&organism=549.408) | [Pyruvate kinase (EC 2.7.1.40)](https://rast.nmpdr.org/seedviewer.cgi?page=FunctionalRole&role=Pyruvate%20kinase%20(EC%202.7.1.40)&subsystem_name=Pyruvate_metabolism_I:_anaplerotic_reactions,_PEP) |
| Carbohydrates | Central carbohydrate metabolism | [Pyruvate metabolism I: anaplerotic reactions, PEP](https://rast.nmpdr.org/seedviewer.cgi?page=Subsystems&subsystem=Pyruvate_metabolism_I:_anaplerotic_reactions,_PEP&organism=549.408) | [Phosphoenolpyruvate synthase (EC 2.7.9.2)](https://rast.nmpdr.org/seedviewer.cgi?page=FunctionalRole&role=Phosphoenolpyruvate%20synthase%20(EC%202.7.9.2)&subsystem_name=Pyruvate_metabolism_I:_anaplerotic_reactions,_PEP) |
| Carbohydrates | Central carbohydrate metabolism | [Pentose phosphate pathway](https://rast.nmpdr.org/seedviewer.cgi?page=Subsystems&subsystem=Pentose_phosphate_pathway&organism=549.408) | [Transketolase (EC 2.2.1.1)](https://rast.nmpdr.org/seedviewer.cgi?page=FunctionalRole&role=Transketolase%20(EC%202.2.1.1)&subsystem_name=Pentose_phosphate_pathway) |
| Carbohydrates | Central carbohydrate metabolism | [Pentose phosphate pathway](https://rast.nmpdr.org/seedviewer.cgi?page=Subsystems&subsystem=Pentose_phosphate_pathway&organism=549.408) | [Transketolase, N-terminal section (EC 2.2.1.1)](https://rast.nmpdr.org/seedviewer.cgi?page=FunctionalRole&role=Transketolase,%20N-terminal%20section%20(EC%202.2.1.1)&subsystem_name=Pentose_phosphate_pathway) |
| Carbohydrates | Central carbohydrate metabolism | [Pentose phosphate pathway](https://rast.nmpdr.org/seedviewer.cgi?page=Subsystems&subsystem=Pentose_phosphate_pathway&organism=549.408) | [Ribose-phosphate pyrophosphokinase (EC 2.7.6.1)](https://rast.nmpdr.org/seedviewer.cgi?page=FunctionalRole&role=Ribose-phosphate%20pyrophosphokinase%20(EC%202.7.6.1)&subsystem_name=Pentose_phosphate_pathway) |
| Carbohydrates | Central carbohydrate metabolism | [Pentose phosphate pathway](https://rast.nmpdr.org/seedviewer.cgi?page=Subsystems&subsystem=Pentose_phosphate_pathway&organism=549.408) | [Ribose 5-phosphate isomerase A (EC 5.3.1.6)](https://rast.nmpdr.org/seedviewer.cgi?page=FunctionalRole&role=Ribose%205-phosphate%20isomerase%20A%20(EC%205.3.1.6)&subsystem_name=Pentose_phosphate_pathway) |
| Carbohydrates | Central carbohydrate metabolism | [Pentose phosphate pathway](https://rast.nmpdr.org/seedviewer.cgi?page=Subsystems&subsystem=Pentose_phosphate_pathway&organism=549.408) | [Transketolase, C-terminal section (EC 2.2.1.1)](https://rast.nmpdr.org/seedviewer.cgi?page=FunctionalRole&role=Transketolase,%20C-terminal%20section%20(EC%202.2.1.1)&subsystem_name=Pentose_phosphate_pathway) |
| Carbohydrates | Central carbohydrate metabolism | [Pentose phosphate pathway](https://rast.nmpdr.org/seedviewer.cgi?page=Subsystems&subsystem=Pentose_phosphate_pathway&organism=549.408) | [Ribulose-phosphate 3-epimerase (EC 5.1.3.1)](https://rast.nmpdr.org/seedviewer.cgi?page=FunctionalRole&role=Ribulose-phosphate%203-epimerase%20(EC%205.1.3.1)&subsystem_name=Pentose_phosphate_pathway) |
| Carbohydrates | Central carbohydrate metabolism | [Pentose phosphate pathway](https://rast.nmpdr.org/seedviewer.cgi?page=Subsystems&subsystem=Pentose_phosphate_pathway&organism=549.408) | [Glucose-6-phosphate 1-dehydrogenase (EC 1.1.1.49)](https://rast.nmpdr.org/seedviewer.cgi?page=FunctionalRole&role=Glucose-6-phosphate%201-dehydrogenase%20(EC%201.1.1.49)&subsystem_name=Pentose_phosphate_pathway) |
| Carbohydrates | Central carbohydrate metabolism | [Pentose phosphate pathway](https://rast.nmpdr.org/seedviewer.cgi?page=Subsystems&subsystem=Pentose_phosphate_pathway&organism=549.408) | [6-phosphogluconate dehydrogenase, decarboxylating (EC 1.1.1.44)](https://rast.nmpdr.org/seedviewer.cgi?page=FunctionalRole&role=6-phosphogluconate%20dehydrogenase,%20decarboxylating%20(EC%201.1.1.44)&subsystem_name=Pentose_phosphate_pathway) |
| Carbohydrates | Central carbohydrate metabolism | [Pentose phosphate pathway](https://rast.nmpdr.org/seedviewer.cgi?page=Subsystems&subsystem=Pentose_phosphate_pathway&organism=549.408) | [6-phosphogluconolactonase (EC 3.1.1.31)](https://rast.nmpdr.org/seedviewer.cgi?page=FunctionalRole&role=6-phosphogluconolactonase%20(EC%203.1.1.31)&subsystem_name=Pentose_phosphate_pathway) |
| Carbohydrates | Central carbohydrate metabolism | [Pentose phosphate pathway](https://rast.nmpdr.org/seedviewer.cgi?page=Subsystems&subsystem=Pentose_phosphate_pathway&organism=549.408) | [Xylulose-5-phosphate phosphoketolase (EC 4.1.2.9)](https://rast.nmpdr.org/seedviewer.cgi?page=FunctionalRole&role=Xylulose-5-phosphate%20phosphoketolase%20(EC%204.1.2.9)&subsystem_name=Pentose_phosphate_pathway) |
| Carbohydrates | Central carbohydrate metabolism | [Pentose phosphate pathway](https://rast.nmpdr.org/seedviewer.cgi?page=Subsystems&subsystem=Pentose_phosphate_pathway&organism=549.408) | [Fructose-6-phosphate phosphoketolase (EC 4.1.2.22)](https://rast.nmpdr.org/seedviewer.cgi?page=FunctionalRole&role=Fructose-6-phosphate%20phosphoketolase%20(EC%204.1.2.22)&subsystem_name=Pentose_phosphate_pathway) |
| Carbohydrates | Central carbohydrate metabolism | [Pentose phosphate pathway](https://rast.nmpdr.org/seedviewer.cgi?page=Subsystems&subsystem=Pentose_phosphate_pathway&organism=549.408) | [Transaldolase (EC 2.2.1.2)](https://rast.nmpdr.org/seedviewer.cgi?page=FunctionalRole&role=Transaldolase%20(EC%202.2.1.2)&subsystem_name=Pentose_phosphate_pathway) |
| Carbohydrates | Central carbohydrate metabolism | [Glycolate, glyoxylate interconversions](https://rast.nmpdr.org/seedviewer.cgi?page=Subsystems&subsystem=Glycolate,_glyoxylate_interconversions&organism=549.408) | [Phosphoglycolate phosphatase (EC 3.1.3.18)](https://rast.nmpdr.org/seedviewer.cgi?page=FunctionalRole&role=Phosphoglycolate%20phosphatase%20(EC%203.1.3.18)&subsystem_name=Glycolate,_glyoxylate_interconversions) |
| Carbohydrates | Central carbohydrate metabolism | [Glycolate, glyoxylate interconversions](https://rast.nmpdr.org/seedviewer.cgi?page=Subsystems&subsystem=Glycolate,_glyoxylate_interconversions&organism=549.408) | [Glycolate dehydrogenase (EC 1.1.99.14), subunit GlcD](https://rast.nmpdr.org/seedviewer.cgi?page=FunctionalRole&role=Glycolate%20dehydrogenase%20(EC%201.1.99.14),%20subunit%20GlcD&subsystem_name=Glycolate,_glyoxylate_interconversions) |
| Carbohydrates | Aminosugars | [Chitin and N-acetylglucosamine utilization](https://rast.nmpdr.org/seedviewer.cgi?page=Subsystems&subsystem=Chitin_and_N-acetylglucosamine_utilization&organism=549.408) | [PTS system, N-acetylglucosamine-specific IIA component (EC 2.7.1.69)](https://rast.nmpdr.org/seedviewer.cgi?page=FunctionalRole&role=PTS%20system,%20N-acetylglucosamine-specific%20IIA%20component%20(EC%202.7.1.69)&subsystem_name=Chitin_and_N-acetylglucosamine_utilization) |
| Carbohydrates | Aminosugars | [Chitin and N-acetylglucosamine utilization](https://rast.nmpdr.org/seedviewer.cgi?page=Subsystems&subsystem=Chitin_and_N-acetylglucosamine_utilization&organism=549.408) | [Glucosamine-6-phosphate deaminase (EC 3.5.99.6)](https://rast.nmpdr.org/seedviewer.cgi?page=FunctionalRole&role=Glucosamine-6-phosphate%20deaminase%20(EC%203.5.99.6)&subsystem_name=Chitin_and_N-acetylglucosamine_utilization) |
| Carbohydrates | Aminosugars | [Chitin and N-acetylglucosamine utilization](https://rast.nmpdr.org/seedviewer.cgi?page=Subsystems&subsystem=Chitin_and_N-acetylglucosamine_utilization&organism=549.408) | [PTS system, N-acetylglucosamine-specific IIB component (EC 2.7.1.69)](https://rast.nmpdr.org/seedviewer.cgi?page=FunctionalRole&role=PTS%20system,%20N-acetylglucosamine-specific%20IIB%20component%20(EC%202.7.1.69)&subsystem_name=Chitin_and_N-acetylglucosamine_utilization) |
| Carbohydrates | Aminosugars | [Chitin and N-acetylglucosamine utilization](https://rast.nmpdr.org/seedviewer.cgi?page=Subsystems&subsystem=Chitin_and_N-acetylglucosamine_utilization&organism=549.408) | [N-acetylglucosamine-6P-responsive transcriptional repressor NagC, ROK family](https://rast.nmpdr.org/seedviewer.cgi?page=FunctionalRole&role=N-acetylglucosamine-6P-responsive%20transcriptional%20repressor%20NagC,%20ROK%20family&subsystem_name=Chitin_and_N-acetylglucosamine_utilization) |
| Carbohydrates | Aminosugars | [Chitin and N-acetylglucosamine utilization](https://rast.nmpdr.org/seedviewer.cgi?page=Subsystems&subsystem=Chitin_and_N-acetylglucosamine_utilization&organism=549.408) | [Beta-hexosaminidase (EC 3.2.1.52)](https://rast.nmpdr.org/seedviewer.cgi?page=FunctionalRole&role=Beta-hexosaminidase%20(EC%203.2.1.52)&subsystem_name=Chitin_and_N-acetylglucosamine_utilization) |
| Carbohydrates | Aminosugars | [Chitin and N-acetylglucosamine utilization](https://rast.nmpdr.org/seedviewer.cgi?page=Subsystems&subsystem=Chitin_and_N-acetylglucosamine_utilization&organism=549.408) | [PTS system, N-acetylglucosamine-specific IIC component (EC 2.7.1.69)](https://rast.nmpdr.org/seedviewer.cgi?page=FunctionalRole&role=PTS%20system,%20N-acetylglucosamine-specific%20IIC%20component%20(EC%202.7.1.69)&subsystem_name=Chitin_and_N-acetylglucosamine_utilization) |
| Carbohydrates | Aminosugars | [Chitin and N-acetylglucosamine utilization](https://rast.nmpdr.org/seedviewer.cgi?page=Subsystems&subsystem=Chitin_and_N-acetylglucosamine_utilization&organism=549.408) | [Phosphatase NagD predicted to act in N-acetylglucosamine utilization subsystem](https://rast.nmpdr.org/seedviewer.cgi?page=FunctionalRole&role=Phosphatase%20NagD%20predicted%20to%20act%20in%20N-acetylglucosamine%20utilization%20subsystem&subsystem_name=Chitin_and_N-acetylglucosamine_utilization) |
| Carbohydrates | Aminosugars | [Chitin and N-acetylglucosamine utilization](https://rast.nmpdr.org/seedviewer.cgi?page=Subsystems&subsystem=Chitin_and_N-acetylglucosamine_utilization&organism=549.408) | [N-acetylglucosamine-regulated outer membrane porin](https://rast.nmpdr.org/seedviewer.cgi?page=FunctionalRole&role=N-acetylglucosamine-regulated%20outer%20membrane%20porin&subsystem_name=Chitin_and_N-acetylglucosamine_utilization) |
| Carbohydrates | Aminosugars | [Chitin and N-acetylglucosamine utilization](https://rast.nmpdr.org/seedviewer.cgi?page=Subsystems&subsystem=Chitin_and_N-acetylglucosamine_utilization&organism=549.408) | [N-acetylglucosamine-6-phosphate deacetylase (EC 3.5.1.25)](https://rast.nmpdr.org/seedviewer.cgi?page=FunctionalRole&role=N-acetylglucosamine-6-phosphate%20deacetylase%20(EC%203.5.1.25)&subsystem_name=Chitin_and_N-acetylglucosamine_utilization) |
| Carbohydrates | Di- and oligosaccharides | [Trehalose Biosynthesis](https://rast.nmpdr.org/seedviewer.cgi?page=Subsystems&subsystem=Trehalose_Biosynthesis&organism=549.408) | [Malto-oligosyltrehalose synthase (EC 5.4.99.15)](https://rast.nmpdr.org/seedviewer.cgi?page=FunctionalRole&role=Malto-oligosyltrehalose%20synthase%20(EC%205.4.99.15)&subsystem_name=Trehalose_Biosynthesis) |
| Carbohydrates | Di- and oligosaccharides | [Trehalose Biosynthesis](https://rast.nmpdr.org/seedviewer.cgi?page=Subsystems&subsystem=Trehalose_Biosynthesis&organism=549.408) | [1,4-alpha-glucan (glycogen) branching enzyme, GH-13-type (EC 2.4.1.18)](https://rast.nmpdr.org/seedviewer.cgi?page=FunctionalRole&role=1,4-alpha-glucan%20(glycogen)%20branching%20enzyme,%20GH-13-type%20(EC%202.4.1.18)&subsystem_name=Trehalose_Biosynthesis) |
| Carbohydrates | Di- and oligosaccharides | [Trehalose Biosynthesis](https://rast.nmpdr.org/seedviewer.cgi?page=Subsystems&subsystem=Trehalose_Biosynthesis&organism=549.408) | [Trehalose-6-phosphate phosphatase (EC 3.1.3.12)](https://rast.nmpdr.org/seedviewer.cgi?page=FunctionalRole&role=Trehalose-6-phosphate%20phosphatase%20(EC%203.1.3.12)&subsystem_name=Trehalose_Biosynthesis) |
| Carbohydrates | Di- and oligosaccharides | [Trehalose Biosynthesis](https://rast.nmpdr.org/seedviewer.cgi?page=Subsystems&subsystem=Trehalose_Biosynthesis&organism=549.408) | [Malto-oligosyltrehalose trehalohydrolase (EC 3.2.1.141)](https://rast.nmpdr.org/seedviewer.cgi?page=FunctionalRole&role=Malto-oligosyltrehalose%20trehalohydrolase%20(EC%203.2.1.141)&subsystem_name=Trehalose_Biosynthesis) |
| Carbohydrates | Di- and oligosaccharides | [Trehalose Biosynthesis](https://rast.nmpdr.org/seedviewer.cgi?page=Subsystems&subsystem=Trehalose_Biosynthesis&organism=549.408) | [Glycogen debranching enzyme (EC 3.2.1.-)](https://rast.nmpdr.org/seedviewer.cgi?page=FunctionalRole&role=Glycogen%20debranching%20enzyme%20(EC%203.2.1.-)&subsystem_name=Trehalose_Biosynthesis) |
| Carbohydrates | Di- and oligosaccharides | [Trehalose Biosynthesis](https://rast.nmpdr.org/seedviewer.cgi?page=Subsystems&subsystem=Trehalose_Biosynthesis&organism=549.408) | [Alpha,alpha-trehalose-phosphate synthase [UDP-forming] (EC 2.4.1.15)](https://rast.nmpdr.org/seedviewer.cgi?page=FunctionalRole&role=Alpha,alpha-trehalose-phosphate%20synthase%20%5bUDP-forming%5d%20(EC%202.4.1.15)&subsystem_name=Trehalose_Biosynthesis) |
| Carbohydrates | Di- and oligosaccharides | [Trehalose Biosynthesis](https://rast.nmpdr.org/seedviewer.cgi?page=Subsystems&subsystem=Trehalose_Biosynthesis&organism=549.408) | [Glucoamylase (EC 3.2.1.3)](https://rast.nmpdr.org/seedviewer.cgi?page=FunctionalRole&role=Glucoamylase%20(EC%203.2.1.3)&subsystem_name=Trehalose_Biosynthesis) |
| Carbohydrates | Di- and oligosaccharides | [Sucrose utilization](https://rast.nmpdr.org/seedviewer.cgi?page=Subsystems&subsystem=Sucrose_utilization&organism=549.408) | [Sucrose operon repressor ScrR, LacI family](https://rast.nmpdr.org/seedviewer.cgi?page=FunctionalRole&role=Sucrose%20operon%20repressor%20ScrR,%20LacI%20family&subsystem_name=Sucrose_utilization) |
| Carbohydrates | Di- and oligosaccharides | [Sucrose utilization](https://rast.nmpdr.org/seedviewer.cgi?page=Subsystems&subsystem=Sucrose_utilization&organism=549.408) | [PTS system, sucrose-specific IIC component (EC 2.7.1.69)](https://rast.nmpdr.org/seedviewer.cgi?page=FunctionalRole&role=PTS%20system,%20sucrose-specific%20IIC%20component%20(EC%202.7.1.69)&subsystem_name=Sucrose_utilization) |
| Carbohydrates | Di- and oligosaccharides | [Sucrose utilization](https://rast.nmpdr.org/seedviewer.cgi?page=Subsystems&subsystem=Sucrose_utilization&organism=549.408) | [PTS system, sucrose-specific IIB component (EC 2.7.1.69)](https://rast.nmpdr.org/seedviewer.cgi?page=FunctionalRole&role=PTS%20system,%20sucrose-specific%20IIB%20component%20(EC%202.7.1.69)&subsystem_name=Sucrose_utilization) |
| Carbohydrates | Di- and oligosaccharides | [Sucrose utilization](https://rast.nmpdr.org/seedviewer.cgi?page=Subsystems&subsystem=Sucrose_utilization&organism=549.408) | [Fructokinase (EC 2.7.1.4)](https://rast.nmpdr.org/seedviewer.cgi?page=FunctionalRole&role=Fructokinase%20(EC%202.7.1.4)&subsystem_name=Sucrose_utilization) |
| Carbohydrates | Di- and oligosaccharides | [Maltose and Maltodextrin Utilization](https://rast.nmpdr.org/seedviewer.cgi?page=Subsystems&subsystem=Maltose_and_Maltodextrin_Utilization&organism=549.408) | [Maltose operon periplasmic protein MalM](https://rast.nmpdr.org/seedviewer.cgi?page=FunctionalRole&role=Maltose%20operon%20periplasmic%20protein%20MalM&subsystem_name=Maltose_and_Maltodextrin_Utilization) |
| Carbohydrates | Di- and oligosaccharides | [Maltose and Maltodextrin Utilization](https://rast.nmpdr.org/seedviewer.cgi?page=Subsystems&subsystem=Maltose_and_Maltodextrin_Utilization&organism=549.408) | [4-alpha-glucanotransferase (amylomaltase) (EC 2.4.1.25)](https://rast.nmpdr.org/seedviewer.cgi?page=FunctionalRole&role=4-alpha-glucanotransferase%20(amylomaltase)%20(EC%202.4.1.25)&subsystem_name=Maltose_and_Maltodextrin_Utilization) |
| Carbohydrates | Di- and oligosaccharides | [Maltose and Maltodextrin Utilization](https://rast.nmpdr.org/seedviewer.cgi?page=Subsystems&subsystem=Maltose_and_Maltodextrin_Utilization&organism=549.408) | [Maltose regulon modulator](https://rast.nmpdr.org/seedviewer.cgi?page=FunctionalRole&role=Maltose%20regulon%20modulator&subsystem_name=Maltose_and_Maltodextrin_Utilization) |
| Carbohydrates | Di- and oligosaccharides | [Maltose and Maltodextrin Utilization](https://rast.nmpdr.org/seedviewer.cgi?page=Subsystems&subsystem=Maltose_and_Maltodextrin_Utilization&organism=549.408) | [Glycogen phosphorylase (EC 2.4.1.1)](https://rast.nmpdr.org/seedviewer.cgi?page=FunctionalRole&role=Glycogen%20phosphorylase%20(EC%202.4.1.1)&subsystem_name=Maltose_and_Maltodextrin_Utilization) |
| Carbohydrates | Di- and oligosaccharides | [Maltose and Maltodextrin Utilization](https://rast.nmpdr.org/seedviewer.cgi?page=Subsystems&subsystem=Maltose_and_Maltodextrin_Utilization&organism=549.408) | [Aldose 1-epimerase (EC 5.1.3.3)](https://rast.nmpdr.org/seedviewer.cgi?page=FunctionalRole&role=Aldose%201-epimerase%20(EC%205.1.3.3)&subsystem_name=Maltose_and_Maltodextrin_Utilization) |
| Carbohydrates | Di- and oligosaccharides | [Maltose and Maltodextrin Utilization](https://rast.nmpdr.org/seedviewer.cgi?page=Subsystems&subsystem=Maltose_and_Maltodextrin_Utilization&organism=549.408) | [Cytoplasmic alpha-amylase (EC 3.2.1.1)](https://rast.nmpdr.org/seedviewer.cgi?page=FunctionalRole&role=Cytoplasmic%20alpha-amylase%20(EC%203.2.1.1)&subsystem_name=Maltose_and_Maltodextrin_Utilization) |
| Carbohydrates | Di- and oligosaccharides | [Maltose and Maltodextrin Utilization](https://rast.nmpdr.org/seedviewer.cgi?page=Subsystems&subsystem=Maltose_and_Maltodextrin_Utilization&organism=549.408) | [Maltose regulon regulatory protein MalI (repressor for malXY)](https://rast.nmpdr.org/seedviewer.cgi?page=FunctionalRole&role=Maltose%20regulon%20regulatory%20protein%20MalI%20(repressor%20for%20malXY)&subsystem_name=Maltose_and_Maltodextrin_Utilization) |
| Carbohydrates | Di- and oligosaccharides | [Maltose and Maltodextrin Utilization](https://rast.nmpdr.org/seedviewer.cgi?page=Subsystems&subsystem=Maltose_and_Maltodextrin_Utilization&organism=549.408) | [PTS system, maltose and glucose-specific IIC component (EC 2.7.1.69)](https://rast.nmpdr.org/seedviewer.cgi?page=FunctionalRole&role=PTS%20system,%20maltose%20and%20glucose-specific%20IIC%20component%20(EC%202.7.1.69)&subsystem_name=Maltose_and_Maltodextrin_Utilization) |
| Carbohydrates | Di- and oligosaccharides | [Maltose and Maltodextrin Utilization](https://rast.nmpdr.org/seedviewer.cgi?page=Subsystems&subsystem=Maltose_and_Maltodextrin_Utilization&organism=549.408) | [Maltose/maltodextrin transport ATP-binding protein MalK (EC 3.6.3.19)](https://rast.nmpdr.org/seedviewer.cgi?page=FunctionalRole&role=Maltose/maltodextrin%20transport%20ATP-binding%20protein%20MalK%20(EC%203.6.3.19)&subsystem_name=Maltose_and_Maltodextrin_Utilization) |
| Carbohydrates | Di- and oligosaccharides | [Maltose and Maltodextrin Utilization](https://rast.nmpdr.org/seedviewer.cgi?page=Subsystems&subsystem=Maltose_and_Maltodextrin_Utilization&organism=549.408) | [PTS system, maltose and glucose-specific IIB component (EC 2.7.1.69)](https://rast.nmpdr.org/seedviewer.cgi?page=FunctionalRole&role=PTS%20system,%20maltose%20and%20glucose-specific%20IIB%20component%20(EC%202.7.1.69)&subsystem_name=Maltose_and_Maltodextrin_Utilization) |
| Carbohydrates | Di- and oligosaccharides | [Maltose and Maltodextrin Utilization](https://rast.nmpdr.org/seedviewer.cgi?page=Subsystems&subsystem=Maltose_and_Maltodextrin_Utilization&organism=549.408) | [Glucoamylase (EC 3.2.1.3)](https://rast.nmpdr.org/seedviewer.cgi?page=FunctionalRole&role=Glucoamylase%20(EC%203.2.1.3)&subsystem_name=Maltose_and_Maltodextrin_Utilization) |
| Carbohydrates | Di- and oligosaccharides | [Maltose and Maltodextrin Utilization](https://rast.nmpdr.org/seedviewer.cgi?page=Subsystems&subsystem=Maltose_and_Maltodextrin_Utilization&organism=549.408) | [Malto-oligosyltrehalose synthase (EC 5.4.99.15)](https://rast.nmpdr.org/seedviewer.cgi?page=FunctionalRole&role=Malto-oligosyltrehalose%20synthase%20(EC%205.4.99.15)&subsystem_name=Maltose_and_Maltodextrin_Utilization) |
| Carbohydrates | Di- and oligosaccharides | [Maltose and Maltodextrin Utilization](https://rast.nmpdr.org/seedviewer.cgi?page=Subsystems&subsystem=Maltose_and_Maltodextrin_Utilization&organism=549.408) | [Maltose/maltodextrin ABC transporter, permease protein MalF](https://rast.nmpdr.org/seedviewer.cgi?page=FunctionalRole&role=Maltose/maltodextrin%20ABC%20transporter,%20permease%20protein%20MalF&subsystem_name=Maltose_and_Maltodextrin_Utilization) |
| Carbohydrates | Di- and oligosaccharides | [Maltose and Maltodextrin Utilization](https://rast.nmpdr.org/seedviewer.cgi?page=Subsystems&subsystem=Maltose_and_Maltodextrin_Utilization&organism=549.408) | [Alpha-glucosidase (EC 3.2.1.20)](https://rast.nmpdr.org/seedviewer.cgi?page=FunctionalRole&role=Alpha-glucosidase%20(EC%203.2.1.20)&subsystem_name=Maltose_and_Maltodextrin_Utilization) |
| Carbohydrates | Di- and oligosaccharides | [Maltose and Maltodextrin Utilization](https://rast.nmpdr.org/seedviewer.cgi?page=Subsystems&subsystem=Maltose_and_Maltodextrin_Utilization&organism=549.408) | [Maltose/maltodextrin ABC transporter, substrate binding periplasmic protein MalE](https://rast.nmpdr.org/seedviewer.cgi?page=FunctionalRole&role=Maltose/maltodextrin%20ABC%20transporter,%20substrate%20binding%20periplasmic%20protein%20MalE&subsystem_name=Maltose_and_Maltodextrin_Utilization) |
| Carbohydrates | Di- and oligosaccharides | [Maltose and Maltodextrin Utilization](https://rast.nmpdr.org/seedviewer.cgi?page=Subsystems&subsystem=Maltose_and_Maltodextrin_Utilization&organism=549.408) | [Maltodextrin phosphorylase (EC 2.4.1.1)](https://rast.nmpdr.org/seedviewer.cgi?page=FunctionalRole&role=Maltodextrin%20phosphorylase%20(EC%202.4.1.1)&subsystem_name=Maltose_and_Maltodextrin_Utilization) |
| Carbohydrates | Di- and oligosaccharides | [Maltose and Maltodextrin Utilization](https://rast.nmpdr.org/seedviewer.cgi?page=Subsystems&subsystem=Maltose_and_Maltodextrin_Utilization&organism=549.408) | [Mlc, transcriptional repressor of MalT (the transcriptional activator of maltose regulon) and manXYZ operon](https://rast.nmpdr.org/seedviewer.cgi?page=FunctionalRole&role=Mlc,%20transcriptional%20repressor%20of%20MalT%20(the%20transcriptional%20activator%20of%20maltose%20regulon)%20and%20manXYZ%20operon&subsystem_name=Maltose_and_Maltodextrin_Utilization) |
| Carbohydrates | Di- and oligosaccharides | [Maltose and Maltodextrin Utilization](https://rast.nmpdr.org/seedviewer.cgi?page=Subsystems&subsystem=Maltose_and_Maltodextrin_Utilization&organism=549.408) | [Transcriptional activator of maltose regulon, MalT](https://rast.nmpdr.org/seedviewer.cgi?page=FunctionalRole&role=Transcriptional%20activator%20of%20maltose%20regulon,%20MalT&subsystem_name=Maltose_and_Maltodextrin_Utilization) |
| Carbohydrates | Di- and oligosaccharides | [Maltose and Maltodextrin Utilization](https://rast.nmpdr.org/seedviewer.cgi?page=Subsystems&subsystem=Maltose_and_Maltodextrin_Utilization&organism=549.408) | [Putative sucrose phosphorylase (EC 2.4.1.7)](https://rast.nmpdr.org/seedviewer.cgi?page=FunctionalRole&role=Putative%20sucrose%20phosphorylase%20(EC%202.4.1.7)&subsystem_name=Maltose_and_Maltodextrin_Utilization) |
| Carbohydrates | Di- and oligosaccharides | [Maltose and Maltodextrin Utilization](https://rast.nmpdr.org/seedviewer.cgi?page=Subsystems&subsystem=Maltose_and_Maltodextrin_Utilization&organism=549.408) | [Multiple sugar ABC transporter, ATP-binding protein](https://rast.nmpdr.org/seedviewer.cgi?page=FunctionalRole&role=Multiple%20sugar%20ABC%20transporter,%20ATP-binding%20protein&subsystem_name=Maltose_and_Maltodextrin_Utilization) |
| Carbohydrates | Di- and oligosaccharides | [Maltose and Maltodextrin Utilization](https://rast.nmpdr.org/seedviewer.cgi?page=Subsystems&subsystem=Maltose_and_Maltodextrin_Utilization&organism=549.408) | [Maltoporin (maltose/maltodextrin high-affinity receptor, phage lambda receptor protein)](https://rast.nmpdr.org/seedviewer.cgi?page=FunctionalRole&role=Maltoporin%20(maltose/maltodextrin%20high-affinity%20receptor,%20phage%20lambda%20receptor%20protein)&subsystem_name=Maltose_and_Maltodextrin_Utilization) |
| Carbohydrates | Di- and oligosaccharides | [Maltose and Maltodextrin Utilization](https://rast.nmpdr.org/seedviewer.cgi?page=Subsystems&subsystem=Maltose_and_Maltodextrin_Utilization&organism=549.408) | [Maltose/maltodextrin ABC transporter, permease protein MalG](https://rast.nmpdr.org/seedviewer.cgi?page=FunctionalRole&role=Maltose/maltodextrin%20ABC%20transporter,%20permease%20protein%20MalG&subsystem_name=Maltose_and_Maltodextrin_Utilization) |
| Carbohydrates | Di- and oligosaccharides | [Beta-Glucoside Metabolism](https://rast.nmpdr.org/seedviewer.cgi?page=Subsystems&subsystem=Beta-Glucoside_Metabolism&organism=549.408) | [AscBF operon repressor](https://rast.nmpdr.org/seedviewer.cgi?page=FunctionalRole&role=AscBF%20operon%20repressor&subsystem_name=Beta-Glucoside_Metabolism) |
| Carbohydrates | Di- and oligosaccharides | [Beta-Glucoside Metabolism](https://rast.nmpdr.org/seedviewer.cgi?page=Subsystems&subsystem=Beta-Glucoside_Metabolism&organism=549.408) | [Transcriptional antiterminator of lichenan operon, BglG family](https://rast.nmpdr.org/seedviewer.cgi?page=FunctionalRole&role=Transcriptional%20antiterminator%20of%20lichenan%20operon,%20BglG%20family&subsystem_name=Beta-Glucoside_Metabolism) |
| Carbohydrates | Di- and oligosaccharides | [Beta-Glucoside Metabolism](https://rast.nmpdr.org/seedviewer.cgi?page=Subsystems&subsystem=Beta-Glucoside_Metabolism&organism=549.408) | [Periplasmic beta-glucosidase (EC 3.2.1.21)](https://rast.nmpdr.org/seedviewer.cgi?page=FunctionalRole&role=Periplasmic%20beta-glucosidase%20(EC%203.2.1.21)&subsystem_name=Beta-Glucoside_Metabolism) |
| Carbohydrates | Di- and oligosaccharides | [Beta-Glucoside Metabolism](https://rast.nmpdr.org/seedviewer.cgi?page=Subsystems&subsystem=Beta-Glucoside_Metabolism&organism=549.408) | [PTS system, cellobiose-specific IIA component (EC 2.7.1.69)](https://rast.nmpdr.org/seedviewer.cgi?page=FunctionalRole&role=PTS%20system,%20cellobiose-specific%20IIA%20component%20(EC%202.7.1.69)&subsystem_name=Beta-Glucoside_Metabolism) |
| Carbohydrates | Di- and oligosaccharides | [Beta-Glucoside Metabolism](https://rast.nmpdr.org/seedviewer.cgi?page=Subsystems&subsystem=Beta-Glucoside_Metabolism&organism=549.408) | [PTS system, beta-glucoside-specific IIB component (EC 2.7.1.69)](https://rast.nmpdr.org/seedviewer.cgi?page=FunctionalRole&role=PTS%20system,%20beta-glucoside-specific%20IIB%20component%20(EC%202.7.1.69)&subsystem_name=Beta-Glucoside_Metabolism) |
| Carbohydrates | Di- and oligosaccharides | [Beta-Glucoside Metabolism](https://rast.nmpdr.org/seedviewer.cgi?page=Subsystems&subsystem=Beta-Glucoside_Metabolism&organism=549.408) | [Beta-glucosidase (EC 3.2.1.21)](https://rast.nmpdr.org/seedviewer.cgi?page=FunctionalRole&role=Beta-glucosidase%20(EC%203.2.1.21)&subsystem_name=Beta-Glucoside_Metabolism) |
| Carbohydrates | Di- and oligosaccharides | [Beta-Glucoside Metabolism](https://rast.nmpdr.org/seedviewer.cgi?page=Subsystems&subsystem=Beta-Glucoside_Metabolism&organism=549.408) | [PTS system, cellobiose-specific IIB component (EC 2.7.1.69)](https://rast.nmpdr.org/seedviewer.cgi?page=FunctionalRole&role=PTS%20system,%20cellobiose-specific%20IIB%20component%20(EC%202.7.1.69)&subsystem_name=Beta-Glucoside_Metabolism) |
| Carbohydrates | Di- and oligosaccharides | [Beta-Glucoside Metabolism](https://rast.nmpdr.org/seedviewer.cgi?page=Subsystems&subsystem=Beta-Glucoside_Metabolism&organism=549.408) | [6-phospho-beta-glucosidase (EC 3.2.1.86)](https://rast.nmpdr.org/seedviewer.cgi?page=FunctionalRole&role=6-phospho-beta-glucosidase%20(EC%203.2.1.86)&subsystem_name=Beta-Glucoside_Metabolism) |
| Carbohydrates | Di- and oligosaccharides | [Beta-Glucoside Metabolism](https://rast.nmpdr.org/seedviewer.cgi?page=Subsystems&subsystem=Beta-Glucoside_Metabolism&organism=549.408) | [PTS system, arbutin-, cellobiose-, and salicin-specific IIBC component (EC 2.7.1.69)](https://rast.nmpdr.org/seedviewer.cgi?page=FunctionalRole&role=PTS%20system,%20arbutin-,%20cellobiose-,%20and%20salicin-specific%20IIBC%20component%20(EC%202.7.1.69)&subsystem_name=Beta-Glucoside_Metabolism) |
| Carbohydrates | Di- and oligosaccharides | [Beta-Glucoside Metabolism](https://rast.nmpdr.org/seedviewer.cgi?page=Subsystems&subsystem=Beta-Glucoside_Metabolism&organism=549.408) | [PTS system, cellobiose-specific IIC component (EC 2.7.1.69)](https://rast.nmpdr.org/seedviewer.cgi?page=FunctionalRole&role=PTS%20system,%20cellobiose-specific%20IIC%20component%20(EC%202.7.1.69)&subsystem_name=Beta-Glucoside_Metabolism) |
| Carbohydrates | Di- and oligosaccharides | [Beta-Glucoside Metabolism](https://rast.nmpdr.org/seedviewer.cgi?page=Subsystems&subsystem=Beta-Glucoside_Metabolism&organism=549.408) | [Beta-glucoside bgl operon antiterminator, BglG family](https://rast.nmpdr.org/seedviewer.cgi?page=FunctionalRole&role=Beta-glucoside%20bgl%20operon%20antiterminator,%20BglG%20family&subsystem_name=Beta-Glucoside_Metabolism) |
| Carbohydrates | Di- and oligosaccharides | [Beta-Glucoside Metabolism](https://rast.nmpdr.org/seedviewer.cgi?page=Subsystems&subsystem=Beta-Glucoside_Metabolism&organism=549.408) | [6-phospho-beta-glucosidase ascB (EC 3.2.1.86)](https://rast.nmpdr.org/seedviewer.cgi?page=FunctionalRole&role=6-phospho-beta-glucosidase%20ascB%20(EC%203.2.1.86)&subsystem_name=Beta-Glucoside_Metabolism) |
| Carbohydrates | Di- and oligosaccharides | [Lactose and Galactose Uptake and Utilization](https://rast.nmpdr.org/seedviewer.cgi?page=Subsystems&subsystem=Lactose_and_Galactose_Uptake_and_Utilization&organism=549.408) | [UDP-glucose 4-epimerase (EC 5.1.3.2)](https://rast.nmpdr.org/seedviewer.cgi?page=FunctionalRole&role=UDP-glucose%204-epimerase%20(EC%205.1.3.2)&subsystem_name=Lactose_and_Galactose_Uptake_and_Utilization) |
| Carbohydrates | Di- and oligosaccharides | [Lactose and Galactose Uptake and Utilization](https://rast.nmpdr.org/seedviewer.cgi?page=Subsystems&subsystem=Lactose_and_Galactose_Uptake_and_Utilization&organism=549.408) | [Transcriptional repressor of the lac operon](https://rast.nmpdr.org/seedviewer.cgi?page=FunctionalRole&role=Transcriptional%20repressor%20of%20the%20lac%20operon&subsystem_name=Lactose_and_Galactose_Uptake_and_Utilization) |
| Carbohydrates | Di- and oligosaccharides | [Lactose and Galactose Uptake and Utilization](https://rast.nmpdr.org/seedviewer.cgi?page=Subsystems&subsystem=Lactose_and_Galactose_Uptake_and_Utilization&organism=549.408) | [Galactose/methyl galactoside ABC transport system, permease protein MglC (TC 3.A.1.2.3)](https://rast.nmpdr.org/seedviewer.cgi?page=FunctionalRole&role=Galactose/methyl%20galactoside%20ABC%20transport%20system,%20permease%20protein%20MglC%20(TC%203.A.1.2.3)&subsystem_name=Lactose_and_Galactose_Uptake_and_Utilization) |
| Carbohydrates | Di- and oligosaccharides | [Lactose and Galactose Uptake and Utilization](https://rast.nmpdr.org/seedviewer.cgi?page=Subsystems&subsystem=Lactose_and_Galactose_Uptake_and_Utilization&organism=549.408) | [Arabinogalactan endo-1,4-beta-galactosidase (EC 3.2.1.89)](https://rast.nmpdr.org/seedviewer.cgi?page=FunctionalRole&role=Arabinogalactan%20endo-1,4-beta-galactosidase%20(EC%203.2.1.89)&subsystem_name=Lactose_and_Galactose_Uptake_and_Utilization) |
| Carbohydrates | Di- and oligosaccharides | [Lactose and Galactose Uptake and Utilization](https://rast.nmpdr.org/seedviewer.cgi?page=Subsystems&subsystem=Lactose_and_Galactose_Uptake_and_Utilization&organism=549.408) | [Lactose permease](https://rast.nmpdr.org/seedviewer.cgi?page=FunctionalRole&role=Lactose%20permease&subsystem_name=Lactose_and_Galactose_Uptake_and_Utilization) |
| Carbohydrates | Di- and oligosaccharides | [Lactose and Galactose Uptake and Utilization](https://rast.nmpdr.org/seedviewer.cgi?page=Subsystems&subsystem=Lactose_and_Galactose_Uptake_and_Utilization&organism=549.408) | [Galactokinase (EC 2.7.1.6)](https://rast.nmpdr.org/seedviewer.cgi?page=FunctionalRole&role=Galactokinase%20(EC%202.7.1.6)&subsystem_name=Lactose_and_Galactose_Uptake_and_Utilization) |
| Carbohydrates | Di- and oligosaccharides | [Lactose and Galactose Uptake and Utilization](https://rast.nmpdr.org/seedviewer.cgi?page=Subsystems&subsystem=Lactose_and_Galactose_Uptake_and_Utilization&organism=549.408) | [Aldose 1-epimerase (EC 5.1.3.3)](https://rast.nmpdr.org/seedviewer.cgi?page=FunctionalRole&role=Aldose%201-epimerase%20(EC%205.1.3.3)&subsystem_name=Lactose_and_Galactose_Uptake_and_Utilization) |
| Carbohydrates | Di- and oligosaccharides | [Lactose and Galactose Uptake and Utilization](https://rast.nmpdr.org/seedviewer.cgi?page=Subsystems&subsystem=Lactose_and_Galactose_Uptake_and_Utilization&organism=549.408) | [Galactose/methyl galactoside ABC transport system, D-galactose-binding periplasmic protein MglB (TC 3.A.1.2.3)](https://rast.nmpdr.org/seedviewer.cgi?page=FunctionalRole&role=Galactose/methyl%20galactoside%20ABC%20transport%20system,%20D-galactose-binding%20periplasmic%20protein%20MglB%20(TC%203.A.1.2.3)&subsystem_name=Lactose_and_Galactose_Uptake_and_Utilization) |
| Carbohydrates | Di- and oligosaccharides | [Lactose and Galactose Uptake and Utilization](https://rast.nmpdr.org/seedviewer.cgi?page=Subsystems&subsystem=Lactose_and_Galactose_Uptake_and_Utilization&organism=549.408) | [Galactose operon repressor, GalR-LacI family of transcriptional regulators](https://rast.nmpdr.org/seedviewer.cgi?page=FunctionalRole&role=Galactose%20operon%20repressor,%20GalR-LacI%20family%20of%20transcriptional%20regulators&subsystem_name=Lactose_and_Galactose_Uptake_and_Utilization) |
| Carbohydrates | Di- and oligosaccharides | [Lactose and Galactose Uptake and Utilization](https://rast.nmpdr.org/seedviewer.cgi?page=Subsystems&subsystem=Lactose_and_Galactose_Uptake_and_Utilization&organism=549.408) | [Beta-galactosidase (EC 3.2.1.23)](https://rast.nmpdr.org/seedviewer.cgi?page=FunctionalRole&role=Beta-galactosidase%20(EC%203.2.1.23)&subsystem_name=Lactose_and_Galactose_Uptake_and_Utilization) |
| Carbohydrates | Di- and oligosaccharides | [Lactose and Galactose Uptake and Utilization](https://rast.nmpdr.org/seedviewer.cgi?page=Subsystems&subsystem=Lactose_and_Galactose_Uptake_and_Utilization&organism=549.408) | [Galactose/methyl galactoside ABC transport system, ATP-binding protein MglA (EC 3.6.3.17)](https://rast.nmpdr.org/seedviewer.cgi?page=FunctionalRole&role=Galactose/methyl%20galactoside%20ABC%20transport%20system,%20ATP-binding%20protein%20MglA%20(EC%203.6.3.17)&subsystem_name=Lactose_and_Galactose_Uptake_and_Utilization) |
| Carbohydrates | Di- and oligosaccharides | [Lactose and Galactose Uptake and Utilization](https://rast.nmpdr.org/seedviewer.cgi?page=Subsystems&subsystem=Lactose_and_Galactose_Uptake_and_Utilization&organism=549.408) | [Mgl repressor and galactose ultrainduction factor GalS, HTH-type transcriptional regulator](https://rast.nmpdr.org/seedviewer.cgi?page=FunctionalRole&role=Mgl%20repressor%20and%20galactose%20ultrainduction%20factor%20GalS,%20HTH-type%20transcriptional%20regulator&subsystem_name=Lactose_and_Galactose_Uptake_and_Utilization) |
| Carbohydrates | Di- and oligosaccharides | [Lactose and Galactose Uptake and Utilization](https://rast.nmpdr.org/seedviewer.cgi?page=Subsystems&subsystem=Lactose_and_Galactose_Uptake_and_Utilization&organism=549.408) | [Galactose-1-phosphate uridylyltransferase (EC 2.7.7.10)](https://rast.nmpdr.org/seedviewer.cgi?page=FunctionalRole&role=Galactose-1-phosphate%20uridylyltransferase%20(EC%202.7.7.10)&subsystem_name=Lactose_and_Galactose_Uptake_and_Utilization) |
| Carbohydrates | Di- and oligosaccharides | [Lactose and Galactose Uptake and Utilization](https://rast.nmpdr.org/seedviewer.cgi?page=Subsystems&subsystem=Lactose_and_Galactose_Uptake_and_Utilization&organism=549.408) | [Lactose phosphotransferase system repressor](https://rast.nmpdr.org/seedviewer.cgi?page=FunctionalRole&role=Lactose%20phosphotransferase%20system%20repressor&subsystem_name=Lactose_and_Galactose_Uptake_and_Utilization) |
| Carbohydrates | Di- and oligosaccharides | [Lactose utilization](https://rast.nmpdr.org/seedviewer.cgi?page=Subsystems&subsystem=Lactose_utilization&organism=549.408) | [Transcriptional repressor of the lac operon](https://rast.nmpdr.org/seedviewer.cgi?page=FunctionalRole&role=Transcriptional%20repressor%20of%20the%20lac%20operon&subsystem_name=Lactose_utilization) |
| Carbohydrates | Di- and oligosaccharides | [Lactose utilization](https://rast.nmpdr.org/seedviewer.cgi?page=Subsystems&subsystem=Lactose_utilization&organism=549.408) | [Beta-galactosidase (EC 3.2.1.23)](https://rast.nmpdr.org/seedviewer.cgi?page=FunctionalRole&role=Beta-galactosidase%20(EC%203.2.1.23)&subsystem_name=Lactose_utilization) |
| Carbohydrates | Di- and oligosaccharides | [Lactose utilization](https://rast.nmpdr.org/seedviewer.cgi?page=Subsystems&subsystem=Lactose_utilization&organism=549.408) | [Lactose permease](https://rast.nmpdr.org/seedviewer.cgi?page=FunctionalRole&role=Lactose%20permease&subsystem_name=Lactose_utilization) |
| Carbohydrates | One-carbon Metabolism | [Serine-glyoxylate cycle](https://rast.nmpdr.org/seedviewer.cgi?page=Subsystems&subsystem=Serine-glyoxylate_cycle&organism=549.408) | [Malate dehydrogenase (EC 1.1.1.37)](https://rast.nmpdr.org/seedviewer.cgi?page=FunctionalRole&role=Malate%20dehydrogenase%20(EC%201.1.1.37)&subsystem_name=Serine-glyoxylate_cycle) |
| Carbohydrates | One-carbon Metabolism | [Serine-glyoxylate cycle](https://rast.nmpdr.org/seedviewer.cgi?page=Subsystems&subsystem=Serine-glyoxylate_cycle&organism=549.408) | [Serine--pyruvate aminotransferase (EC 2.6.1.51)](https://rast.nmpdr.org/seedviewer.cgi?page=FunctionalRole&role=Serine--pyruvate%20aminotransferase%20(EC%202.6.1.51)&subsystem_name=Serine-glyoxylate_cycle) |
| Carbohydrates | One-carbon Metabolism | [Serine-glyoxylate cycle](https://rast.nmpdr.org/seedviewer.cgi?page=Subsystems&subsystem=Serine-glyoxylate_cycle&organism=549.408) | [Serine hydroxymethyltransferase (EC 2.1.2.1)](https://rast.nmpdr.org/seedviewer.cgi?page=FunctionalRole&role=Serine%20hydroxymethyltransferase%20(EC%202.1.2.1)&subsystem_name=Serine-glyoxylate_cycle) |
| Carbohydrates | One-carbon Metabolism | [Serine-glyoxylate cycle](https://rast.nmpdr.org/seedviewer.cgi?page=Subsystems&subsystem=Serine-glyoxylate_cycle&organism=549.408) | [Succinate dehydrogenase flavoprotein subunit (EC 1.3.99.1)](https://rast.nmpdr.org/seedviewer.cgi?page=FunctionalRole&role=Succinate%20dehydrogenase%20flavoprotein%20subunit%20(EC%201.3.99.1)&subsystem_name=Serine-glyoxylate_cycle) |
| Carbohydrates | One-carbon Metabolism | [Serine-glyoxylate cycle](https://rast.nmpdr.org/seedviewer.cgi?page=Subsystems&subsystem=Serine-glyoxylate_cycle&organism=549.408) | [Aconitate hydratase (EC 4.2.1.3)](https://rast.nmpdr.org/seedviewer.cgi?page=FunctionalRole&role=Aconitate%20hydratase%20(EC%204.2.1.3)&subsystem_name=Serine-glyoxylate_cycle) |
| Carbohydrates | One-carbon Metabolism | [Serine-glyoxylate cycle](https://rast.nmpdr.org/seedviewer.cgi?page=Subsystems&subsystem=Serine-glyoxylate_cycle&organism=549.408) | [Succinate dehydrogenase iron-sulfur protein (EC 1.3.99.1)](https://rast.nmpdr.org/seedviewer.cgi?page=FunctionalRole&role=Succinate%20dehydrogenase%20iron-sulfur%20protein%20(EC%201.3.99.1)&subsystem_name=Serine-glyoxylate_cycle) |
| Carbohydrates | One-carbon Metabolism | [Serine-glyoxylate cycle](https://rast.nmpdr.org/seedviewer.cgi?page=Subsystems&subsystem=Serine-glyoxylate_cycle&organism=549.408) | [Citrate synthase (si) (EC 2.3.3.1)](https://rast.nmpdr.org/seedviewer.cgi?page=FunctionalRole&role=Citrate%20synthase%20(si)%20(EC%202.3.3.1)&subsystem_name=Serine-glyoxylate_cycle) |
| Carbohydrates | One-carbon Metabolism | [Serine-glyoxylate cycle](https://rast.nmpdr.org/seedviewer.cgi?page=Subsystems&subsystem=Serine-glyoxylate_cycle&organism=549.408) | [5,10-methylenetetrahydrofolate reductase (EC 1.5.1.20)](https://rast.nmpdr.org/seedviewer.cgi?page=FunctionalRole&role=5,10-methylenetetrahydrofolate%20reductase%20(EC%201.5.1.20)&subsystem_name=Serine-glyoxylate_cycle) |
| Carbohydrates | One-carbon Metabolism | [Serine-glyoxylate cycle](https://rast.nmpdr.org/seedviewer.cgi?page=Subsystems&subsystem=Serine-glyoxylate_cycle&organism=549.408) | [Methenyltetrahydrofolate cyclohydrolase (EC 3.5.4.9)](https://rast.nmpdr.org/seedviewer.cgi?page=FunctionalRole&role=Methenyltetrahydrofolate%20cyclohydrolase%20(EC%203.5.4.9)&subsystem_name=Serine-glyoxylate_cycle) |
| Carbohydrates | One-carbon Metabolism | [Serine-glyoxylate cycle](https://rast.nmpdr.org/seedviewer.cgi?page=Subsystems&subsystem=Serine-glyoxylate_cycle&organism=549.408) | [Enolase (EC 4.2.1.11)](https://rast.nmpdr.org/seedviewer.cgi?page=FunctionalRole&role=Enolase%20(EC%204.2.1.11)&subsystem_name=Serine-glyoxylate_cycle) |
| Carbohydrates | One-carbon Metabolism | [Serine-glyoxylate cycle](https://rast.nmpdr.org/seedviewer.cgi?page=Subsystems&subsystem=Serine-glyoxylate_cycle&organism=549.408) | [5-formyltetrahydrofolate cyclo-ligase (EC 6.3.3.2)](https://rast.nmpdr.org/seedviewer.cgi?page=FunctionalRole&role=5-formyltetrahydrofolate%20cyclo-ligase%20(EC%206.3.3.2)&subsystem_name=Serine-glyoxylate_cycle) |
| Carbohydrates | One-carbon Metabolism | [Serine-glyoxylate cycle](https://rast.nmpdr.org/seedviewer.cgi?page=Subsystems&subsystem=Serine-glyoxylate_cycle&organism=549.408) | [Phosphoenolpyruvate carboxykinase [ATP] (EC 4.1.1.49)](https://rast.nmpdr.org/seedviewer.cgi?page=FunctionalRole&role=Phosphoenolpyruvate%20carboxykinase%20%5bATP%5d%20(EC%204.1.1.49)&subsystem_name=Serine-glyoxylate_cycle) |
| Carbohydrates | One-carbon Metabolism | [Serine-glyoxylate cycle](https://rast.nmpdr.org/seedviewer.cgi?page=Subsystems&subsystem=Serine-glyoxylate_cycle&organism=549.408) | [Methylenetetrahydrofolate dehydrogenase (NADP+) (EC 1.5.1.5)](https://rast.nmpdr.org/seedviewer.cgi?page=FunctionalRole&role=Methylenetetrahydrofolate%20dehydrogenase%20(NADP+)%20(EC%201.5.1.5)&subsystem_name=Serine-glyoxylate_cycle) |
| Carbohydrates | One-carbon Metabolism | [Serine-glyoxylate cycle](https://rast.nmpdr.org/seedviewer.cgi?page=Subsystems&subsystem=Serine-glyoxylate_cycle&organism=549.408) | [Acetyl-CoA acetyltransferase (EC 2.3.1.9)](https://rast.nmpdr.org/seedviewer.cgi?page=FunctionalRole&role=Acetyl-CoA%20acetyltransferase%20(EC%202.3.1.9)&subsystem_name=Serine-glyoxylate_cycle) |
| Carbohydrates | One-carbon Metabolism | [Serine-glyoxylate cycle](https://rast.nmpdr.org/seedviewer.cgi?page=Subsystems&subsystem=Serine-glyoxylate_cycle&organism=549.408) | [Succinyl-CoA ligase [ADP-forming] alpha chain (EC 6.2.1.5)](https://rast.nmpdr.org/seedviewer.cgi?page=FunctionalRole&role=Succinyl-CoA%20ligase%20%5bADP-forming%5d%20alpha%20chain%20(EC%206.2.1.5)&subsystem_name=Serine-glyoxylate_cycle) |
| Carbohydrates | One-carbon Metabolism | [Serine-glyoxylate cycle](https://rast.nmpdr.org/seedviewer.cgi?page=Subsystems&subsystem=Serine-glyoxylate_cycle&organism=549.408) | [3-ketoacyl-CoA thiolase (EC 2.3.1.16)](https://rast.nmpdr.org/seedviewer.cgi?page=FunctionalRole&role=3-ketoacyl-CoA%20thiolase%20(EC%202.3.1.16)&subsystem_name=Serine-glyoxylate_cycle) |
| Carbohydrates | One-carbon Metabolism | [Serine-glyoxylate cycle](https://rast.nmpdr.org/seedviewer.cgi?page=Subsystems&subsystem=Serine-glyoxylate_cycle&organism=549.408) | [Isocitrate lyase (EC 4.1.3.1)](https://rast.nmpdr.org/seedviewer.cgi?page=FunctionalRole&role=Isocitrate%20lyase%20(EC%204.1.3.1)&subsystem_name=Serine-glyoxylate_cycle) |
| Carbohydrates | One-carbon Metabolism | [Serine-glyoxylate cycle](https://rast.nmpdr.org/seedviewer.cgi?page=Subsystems&subsystem=Serine-glyoxylate_cycle&organism=549.408) | [Fumarate hydratase class I, aerobic (EC 4.2.1.2)](https://rast.nmpdr.org/seedviewer.cgi?page=FunctionalRole&role=Fumarate%20hydratase%20class%20I,%20aerobic%20(EC%204.2.1.2)&subsystem_name=Serine-glyoxylate_cycle) |
| Carbohydrates | One-carbon Metabolism | [Serine-glyoxylate cycle](https://rast.nmpdr.org/seedviewer.cgi?page=Subsystems&subsystem=Serine-glyoxylate_cycle&organism=549.408) | [low-specificity D-threonine aldolase](https://rast.nmpdr.org/seedviewer.cgi?page=FunctionalRole&role=low-specificity%20D-threonine%20aldolase&subsystem_name=Serine-glyoxylate_cycle) |
| Carbohydrates | One-carbon Metabolism | [Serine-glyoxylate cycle](https://rast.nmpdr.org/seedviewer.cgi?page=Subsystems&subsystem=Serine-glyoxylate_cycle&organism=549.408) | [Succinyl-CoA ligase [ADP-forming] beta chain (EC 6.2.1.5)](https://rast.nmpdr.org/seedviewer.cgi?page=FunctionalRole&role=Succinyl-CoA%20ligase%20%5bADP-forming%5d%20beta%20chain%20(EC%206.2.1.5)&subsystem_name=Serine-glyoxylate_cycle) |
| Carbohydrates | One-carbon Metabolism | [Serine-glyoxylate cycle](https://rast.nmpdr.org/seedviewer.cgi?page=Subsystems&subsystem=Serine-glyoxylate_cycle&organism=549.408) | [Glycerate kinase (EC 2.7.1.31)](https://rast.nmpdr.org/seedviewer.cgi?page=FunctionalRole&role=Glycerate%20kinase%20(EC%202.7.1.31)&subsystem_name=Serine-glyoxylate_cycle) |
| Carbohydrates | One-carbon Metabolism | [One-carbon metabolism by tetrahydropterines](https://rast.nmpdr.org/seedviewer.cgi?page=Subsystems&subsystem=One-carbon_metabolism_by_tetrahydropterines&organism=549.408) | [Formyltetrahydrofolate deformylase (EC 3.5.1.10)](https://rast.nmpdr.org/seedviewer.cgi?page=FunctionalRole&role=Formyltetrahydrofolate%20deformylase%20(EC%203.5.1.10)&subsystem_name=One-carbon_metabolism_by_tetrahydropterines) |
| Carbohydrates | One-carbon Metabolism | [One-carbon metabolism by tetrahydropterines](https://rast.nmpdr.org/seedviewer.cgi?page=Subsystems&subsystem=One-carbon_metabolism_by_tetrahydropterines&organism=549.408) | [5-formyltetrahydrofolate cyclo-ligase (EC 6.3.3.2)](https://rast.nmpdr.org/seedviewer.cgi?page=FunctionalRole&role=5-formyltetrahydrofolate%20cyclo-ligase%20(EC%206.3.3.2)&subsystem_name=One-carbon_metabolism_by_tetrahydropterines) |
| Carbohydrates | One-carbon Metabolism | [One-carbon metabolism by tetrahydropterines](https://rast.nmpdr.org/seedviewer.cgi?page=Subsystems&subsystem=One-carbon_metabolism_by_tetrahydropterines&organism=549.408) | [Methylenetetrahydrofolate dehydrogenase (NADP+) (EC 1.5.1.5)](https://rast.nmpdr.org/seedviewer.cgi?page=FunctionalRole&role=Methylenetetrahydrofolate%20dehydrogenase%20(NADP+)%20(EC%201.5.1.5)&subsystem_name=One-carbon_metabolism_by_tetrahydropterines) |
| Carbohydrates | One-carbon Metabolism | [One-carbon metabolism by tetrahydropterines](https://rast.nmpdr.org/seedviewer.cgi?page=Subsystems&subsystem=One-carbon_metabolism_by_tetrahydropterines&organism=549.408) | [5,10-methylenetetrahydrofolate reductase (EC 1.5.1.20)](https://rast.nmpdr.org/seedviewer.cgi?page=FunctionalRole&role=5,10-methylenetetrahydrofolate%20reductase%20(EC%201.5.1.20)&subsystem_name=One-carbon_metabolism_by_tetrahydropterines) |
| Carbohydrates | One-carbon Metabolism | [One-carbon metabolism by tetrahydropterines](https://rast.nmpdr.org/seedviewer.cgi?page=Subsystems&subsystem=One-carbon_metabolism_by_tetrahydropterines&organism=549.408) | [Methenyltetrahydrofolate cyclohydrolase (EC 3.5.4.9)](https://rast.nmpdr.org/seedviewer.cgi?page=FunctionalRole&role=Methenyltetrahydrofolate%20cyclohydrolase%20(EC%203.5.4.9)&subsystem_name=One-carbon_metabolism_by_tetrahydropterines) |
| Carbohydrates | Organic acids | [Glycerate metabolism](https://rast.nmpdr.org/seedviewer.cgi?page=Subsystems&subsystem=Glycerate_metabolism&organism=549.408) | [Hydroxypyruvate isomerase (EC 5.3.1.22)](https://rast.nmpdr.org/seedviewer.cgi?page=FunctionalRole&role=Hydroxypyruvate%20isomerase%20(EC%205.3.1.22)&subsystem_name=Glycerate_metabolism) |
| Carbohydrates | Organic acids | [Glycerate metabolism](https://rast.nmpdr.org/seedviewer.cgi?page=Subsystems&subsystem=Glycerate_metabolism&organism=549.408) | [2-dehydro-3-deoxyglucarate aldolase (EC 4.1.2.20)](https://rast.nmpdr.org/seedviewer.cgi?page=FunctionalRole&role=2-dehydro-3-deoxyglucarate%20aldolase%20(EC%204.1.2.20)&subsystem_name=Glycerate_metabolism) |
| Carbohydrates | Organic acids | [Glycerate metabolism](https://rast.nmpdr.org/seedviewer.cgi?page=Subsystems&subsystem=Glycerate_metabolism&organism=549.408) | [2-hydroxy-3-oxopropionate reductase (EC 1.1.1.60)](https://rast.nmpdr.org/seedviewer.cgi?page=FunctionalRole&role=2-hydroxy-3-oxopropionate%20reductase%20(EC%201.1.1.60)&subsystem_name=Glycerate_metabolism) |
| Carbohydrates | Organic acids | [Glycerate metabolism](https://rast.nmpdr.org/seedviewer.cgi?page=Subsystems&subsystem=Glycerate_metabolism&organism=549.408) | [Pyruvate kinase (EC 2.7.1.40)](https://rast.nmpdr.org/seedviewer.cgi?page=FunctionalRole&role=Pyruvate%20kinase%20(EC%202.7.1.40)&subsystem_name=Glycerate_metabolism) |
| Carbohydrates | Organic acids | [Glycerate metabolism](https://rast.nmpdr.org/seedviewer.cgi?page=Subsystems&subsystem=Glycerate_metabolism&organism=549.408) | [Tartrate decarboxylase (EC 4.1.1.73)](https://rast.nmpdr.org/seedviewer.cgi?page=FunctionalRole&role=Tartrate%20decarboxylase%20(EC%204.1.1.73)&subsystem_name=Glycerate_metabolism) |
| Carbohydrates | Organic acids | [Glycerate metabolism](https://rast.nmpdr.org/seedviewer.cgi?page=Subsystems&subsystem=Glycerate_metabolism&organism=549.408) | [Glycerate kinase (EC 2.7.1.31)](https://rast.nmpdr.org/seedviewer.cgi?page=FunctionalRole&role=Glycerate%20kinase%20(EC%202.7.1.31)&subsystem_name=Glycerate_metabolism) |
| Carbohydrates | Organic acids | [Lactate utilization](https://rast.nmpdr.org/seedviewer.cgi?page=Subsystems&subsystem=Lactate_utilization&organism=549.408) | [L-lactate permease](https://rast.nmpdr.org/seedviewer.cgi?page=FunctionalRole&role=L-lactate%20permease&subsystem_name=Lactate_utilization) |
| Carbohydrates | Organic acids | [Lactate utilization](https://rast.nmpdr.org/seedviewer.cgi?page=Subsystems&subsystem=Lactate_utilization&organism=549.408) | [Lactate-responsive regulator LldR in Enterobacteria, GntR family](https://rast.nmpdr.org/seedviewer.cgi?page=FunctionalRole&role=Lactate-responsive%20regulator%20LldR%20in%20Enterobacteria,%20GntR%20family&subsystem_name=Lactate_utilization) |
| Carbohydrates | Organic acids | [Lactate utilization](https://rast.nmpdr.org/seedviewer.cgi?page=Subsystems&subsystem=Lactate_utilization&organism=549.408) | [L-lactate dehydrogenase (EC 1.1.2.3)](https://rast.nmpdr.org/seedviewer.cgi?page=FunctionalRole&role=L-lactate%20dehydrogenase%20(EC%201.1.2.3)&subsystem_name=Lactate_utilization) |
| Carbohydrates | Organic acids | [Lactate utilization](https://rast.nmpdr.org/seedviewer.cgi?page=Subsystems&subsystem=Lactate_utilization&organism=549.408) | [D-Lactate dehydrogenase (EC 1.1.2.5)](https://rast.nmpdr.org/seedviewer.cgi?page=FunctionalRole&role=D-Lactate%20dehydrogenase%20(EC%201.1.2.5)&subsystem_name=Lactate_utilization) |
| Carbohydrates | Organic acids | [Malonate decarboxylase](https://rast.nmpdr.org/seedviewer.cgi?page=Subsystems&subsystem=Malonate_decarboxylase&organism=549.408) | [Triphosphoribosyl-dephospho-CoA synthetase (EC 2.7.8.25)](https://rast.nmpdr.org/seedviewer.cgi?page=FunctionalRole&role=Triphosphoribosyl-dephospho-CoA%20synthetase%20(EC%202.7.8.25)&subsystem_name=Malonate_decarboxylase) |
| Carbohydrates | Organic acids | [Malonate decarboxylase](https://rast.nmpdr.org/seedviewer.cgi?page=Subsystems&subsystem=Malonate_decarboxylase&organism=549.408) | [Phosphoribosyl-dephospho-CoA transferase (EC 2.7.7.-)](https://rast.nmpdr.org/seedviewer.cgi?page=FunctionalRole&role=Phosphoribosyl-dephospho-CoA%20transferase%20(EC%202.7.7.-)&subsystem_name=Malonate_decarboxylase) |
| Carbohydrates | Organic acids | [Malonate decarboxylase](https://rast.nmpdr.org/seedviewer.cgi?page=Subsystems&subsystem=Malonate_decarboxylase&organism=549.408) | [Malonate decarboxylase delta subunit](https://rast.nmpdr.org/seedviewer.cgi?page=FunctionalRole&role=Malonate%20decarboxylase%20delta%20subunit&subsystem_name=Malonate_decarboxylase) |
| Carbohydrates | Organic acids | [Malonate decarboxylase](https://rast.nmpdr.org/seedviewer.cgi?page=Subsystems&subsystem=Malonate_decarboxylase&organism=549.408) | [Malonate decarboxylase beta subunit](https://rast.nmpdr.org/seedviewer.cgi?page=FunctionalRole&role=Malonate%20decarboxylase%20beta%20subunit&subsystem_name=Malonate_decarboxylase) |
| Carbohydrates | Organic acids | [Malonate decarboxylase](https://rast.nmpdr.org/seedviewer.cgi?page=Subsystems&subsystem=Malonate_decarboxylase&organism=549.408) | [Malonyl CoA acyl carrier protein transacylase (EC 2.3.1.39)](https://rast.nmpdr.org/seedviewer.cgi?page=FunctionalRole&role=Malonyl%20CoA%20acyl%20carrier%20protein%20transacylase%20(EC%202.3.1.39)&subsystem_name=Malonate_decarboxylase) |
| Carbohydrates | Organic acids | [Malonate decarboxylase](https://rast.nmpdr.org/seedviewer.cgi?page=Subsystems&subsystem=Malonate_decarboxylase&organism=549.408) | [Malonate decarboxylase alpha subunit](https://rast.nmpdr.org/seedviewer.cgi?page=FunctionalRole&role=Malonate%20decarboxylase%20alpha%20subunit&subsystem_name=Malonate_decarboxylase) |
| Carbohydrates | Organic acids | [Malonate decarboxylase](https://rast.nmpdr.org/seedviewer.cgi?page=Subsystems&subsystem=Malonate_decarboxylase&organism=549.408) | [Malonate utilization transcriptional regulator](https://rast.nmpdr.org/seedviewer.cgi?page=FunctionalRole&role=Malonate%20utilization%20transcriptional%20regulator&subsystem_name=Malonate_decarboxylase) |
| Carbohydrates | Organic acids | [Malonate decarboxylase](https://rast.nmpdr.org/seedviewer.cgi?page=Subsystems&subsystem=Malonate_decarboxylase&organism=549.408) | [Malonate decarboxylase gamma subunit](https://rast.nmpdr.org/seedviewer.cgi?page=FunctionalRole&role=Malonate%20decarboxylase%20gamma%20subunit&subsystem_name=Malonate_decarboxylase) |
| Carbohydrates | Organic acids | [Alpha-acetolactate operon](https://rast.nmpdr.org/seedviewer.cgi?page=Subsystems&subsystem=Alpha-acetolactate_operon&organism=549.408) | [Alpha-acetolactate decarboxylase (EC 4.1.1.5)](https://rast.nmpdr.org/seedviewer.cgi?page=FunctionalRole&role=Alpha-acetolactate%20decarboxylase%20(EC%204.1.1.5)&subsystem_name=Alpha-acetolactate_operon) |
| Carbohydrates | Organic acids | [Alpha-acetolactate operon](https://rast.nmpdr.org/seedviewer.cgi?page=Subsystems&subsystem=Alpha-acetolactate_operon&organism=549.408) | [Acetolactate synthase, catabolic (EC 2.2.1.6)](https://rast.nmpdr.org/seedviewer.cgi?page=FunctionalRole&role=Acetolactate%20synthase,%20catabolic%20(EC%202.2.1.6)&subsystem_name=Alpha-acetolactate_operon) |
| Carbohydrates | Fermentation | [Butanol Biosynthesis](https://rast.nmpdr.org/seedviewer.cgi?page=Subsystems&subsystem=Butanol_Biosynthesis&organism=549.408) | [Alcohol dehydrogenase (EC 1.1.1.1)](https://rast.nmpdr.org/seedviewer.cgi?page=FunctionalRole&role=Alcohol%20dehydrogenase%20(EC%201.1.1.1)&subsystem_name=Butanol_Biosynthesis) |
| Carbohydrates | Fermentation | [Butanol Biosynthesis](https://rast.nmpdr.org/seedviewer.cgi?page=Subsystems&subsystem=Butanol_Biosynthesis&organism=549.408) | [Enoyl-CoA hydratase (EC 4.2.1.17)](https://rast.nmpdr.org/seedviewer.cgi?page=FunctionalRole&role=Enoyl-CoA%20hydratase%20(EC%204.2.1.17)&subsystem_name=Butanol_Biosynthesis) |
| Carbohydrates | Fermentation | [Butanol Biosynthesis](https://rast.nmpdr.org/seedviewer.cgi?page=Subsystems&subsystem=Butanol_Biosynthesis&organism=549.408) | [Pyruvate formate-lyase (EC 2.3.1.54)](https://rast.nmpdr.org/seedviewer.cgi?page=FunctionalRole&role=Pyruvate%20formate-lyase%20(EC%202.3.1.54)&subsystem_name=Butanol_Biosynthesis) |
| Carbohydrates | Fermentation | [Butanol Biosynthesis](https://rast.nmpdr.org/seedviewer.cgi?page=Subsystems&subsystem=Butanol_Biosynthesis&organism=549.408) | [Acetaldehyde dehydrogenase (EC 1.2.1.10)](https://rast.nmpdr.org/seedviewer.cgi?page=FunctionalRole&role=Acetaldehyde%20dehydrogenase%20(EC%201.2.1.10)&subsystem_name=Butanol_Biosynthesis) |
| Carbohydrates | Fermentation | [Butanol Biosynthesis](https://rast.nmpdr.org/seedviewer.cgi?page=Subsystems&subsystem=Butanol_Biosynthesis&organism=549.408) | [Acetyl-CoA acetyltransferase (EC 2.3.1.9)](https://rast.nmpdr.org/seedviewer.cgi?page=FunctionalRole&role=Acetyl-CoA%20acetyltransferase%20(EC%202.3.1.9)&subsystem_name=Butanol_Biosynthesis) |
| Carbohydrates | Fermentation | [Fermentations: Mixed acid](https://rast.nmpdr.org/seedviewer.cgi?page=Subsystems&subsystem=Fermentations:_Mixed_acid&organism=549.408) | [D-lactate dehydrogenase (EC 1.1.1.28)](https://rast.nmpdr.org/seedviewer.cgi?page=FunctionalRole&role=D-lactate%20dehydrogenase%20(EC%201.1.1.28)&subsystem_name=Fermentations:_Mixed_acid) |
| Carbohydrates | Fermentation | [Fermentations: Mixed acid](https://rast.nmpdr.org/seedviewer.cgi?page=Subsystems&subsystem=Fermentations:_Mixed_acid&organism=549.408) | [Alcohol dehydrogenase (EC 1.1.1.1)](https://rast.nmpdr.org/seedviewer.cgi?page=FunctionalRole&role=Alcohol%20dehydrogenase%20(EC%201.1.1.1)&subsystem_name=Fermentations:_Mixed_acid) |
| Carbohydrates | Fermentation | [Fermentations: Mixed acid](https://rast.nmpdr.org/seedviewer.cgi?page=Subsystems&subsystem=Fermentations:_Mixed_acid&organism=549.408) | [Acetaldehyde dehydrogenase (EC 1.2.1.10)](https://rast.nmpdr.org/seedviewer.cgi?page=FunctionalRole&role=Acetaldehyde%20dehydrogenase%20(EC%201.2.1.10)&subsystem_name=Fermentations:_Mixed_acid) |
| Carbohydrates | Fermentation | [Fermentations: Mixed acid](https://rast.nmpdr.org/seedviewer.cgi?page=Subsystems&subsystem=Fermentations:_Mixed_acid&organism=549.408) | [Formate efflux transporter (TC 2.A.44 family)](https://rast.nmpdr.org/seedviewer.cgi?page=FunctionalRole&role=Formate%20efflux%20transporter%20(TC%202.A.44%20family)&subsystem_name=Fermentations:_Mixed_acid) |
| Carbohydrates | Fermentation | [Fermentations: Mixed acid](https://rast.nmpdr.org/seedviewer.cgi?page=Subsystems&subsystem=Fermentations:_Mixed_acid&organism=549.408) | [Pyruvate formate-lyase activating enzyme (EC 1.97.1.4)](https://rast.nmpdr.org/seedviewer.cgi?page=FunctionalRole&role=Pyruvate%20formate-lyase%20activating%20enzyme%20(EC%201.97.1.4)&subsystem_name=Fermentations:_Mixed_acid) |
| Carbohydrates | Fermentation | [Fermentations: Mixed acid](https://rast.nmpdr.org/seedviewer.cgi?page=Subsystems&subsystem=Fermentations:_Mixed_acid&organism=549.408) | [Sugar/maltose fermentation stimulation protein homolog](https://rast.nmpdr.org/seedviewer.cgi?page=FunctionalRole&role=Sugar/maltose%20fermentation%20stimulation%20protein%20homolog&subsystem_name=Fermentations:_Mixed_acid) |
| Carbohydrates | Fermentation | [Fermentations: Mixed acid](https://rast.nmpdr.org/seedviewer.cgi?page=Subsystems&subsystem=Fermentations:_Mixed_acid&organism=549.408) | [Pyruvate formate-lyase (EC 2.3.1.54)](https://rast.nmpdr.org/seedviewer.cgi?page=FunctionalRole&role=Pyruvate%20formate-lyase%20(EC%202.3.1.54)&subsystem_name=Fermentations:_Mixed_acid) |
| Carbohydrates | Fermentation | [Fermentations: Mixed acid](https://rast.nmpdr.org/seedviewer.cgi?page=Subsystems&subsystem=Fermentations:_Mixed_acid&organism=549.408) | [Phosphoenolpyruvate carboxylase (EC 4.1.1.31)](https://rast.nmpdr.org/seedviewer.cgi?page=FunctionalRole&role=Phosphoenolpyruvate%20carboxylase%20(EC%204.1.1.31)&subsystem_name=Fermentations:_Mixed_acid) |
| Carbohydrates | Fermentation | [Fermentations: Mixed acid](https://rast.nmpdr.org/seedviewer.cgi?page=Subsystems&subsystem=Fermentations:_Mixed_acid&organism=549.408) | [Acetate kinase (EC 2.7.2.1)](https://rast.nmpdr.org/seedviewer.cgi?page=FunctionalRole&role=Acetate%20kinase%20(EC%202.7.2.1)&subsystem_name=Fermentations:_Mixed_acid) |
| Carbohydrates | Fermentation | [Fermentations: Mixed acid](https://rast.nmpdr.org/seedviewer.cgi?page=Subsystems&subsystem=Fermentations:_Mixed_acid&organism=549.408) | [Phosphate acetyltransferase (EC 2.3.1.8)](https://rast.nmpdr.org/seedviewer.cgi?page=FunctionalRole&role=Phosphate%20acetyltransferase%20(EC%202.3.1.8)&subsystem_name=Fermentations:_Mixed_acid) |
| Carbohydrates | Fermentation | [Acetolactate synthase subunits](https://rast.nmpdr.org/seedviewer.cgi?page=Subsystems&subsystem=Acetolactate_synthase_subunits&organism=549.408) | [Acetolactate synthase large subunit (EC 2.2.1.6)](https://rast.nmpdr.org/seedviewer.cgi?page=FunctionalRole&role=Acetolactate%20synthase%20large%20subunit%20(EC%202.2.1.6)&subsystem_name=Acetolactate_synthase_subunits) |
| Carbohydrates | Fermentation | [Acetolactate synthase subunits](https://rast.nmpdr.org/seedviewer.cgi?page=Subsystems&subsystem=Acetolactate_synthase_subunits&organism=549.408) | [Acetolactate synthase small subunit (EC 2.2.1.6)](https://rast.nmpdr.org/seedviewer.cgi?page=FunctionalRole&role=Acetolactate%20synthase%20small%20subunit%20(EC%202.2.1.6)&subsystem_name=Acetolactate_synthase_subunits) |
| Carbohydrates | Fermentation | [Fermentations: Lactate](https://rast.nmpdr.org/seedviewer.cgi?page=Subsystems&subsystem=Fermentations:_Lactate&organism=549.408) | [D-lactate dehydrogenase (EC 1.1.1.28)](https://rast.nmpdr.org/seedviewer.cgi?page=FunctionalRole&role=D-lactate%20dehydrogenase%20(EC%201.1.1.28)&subsystem_name=Fermentations:_Lactate) |
| Carbohydrates | Fermentation | [Fermentations: Lactate](https://rast.nmpdr.org/seedviewer.cgi?page=Subsystems&subsystem=Fermentations:_Lactate&organism=549.408) | [Acetaldehyde dehydrogenase (EC 1.2.1.10)](https://rast.nmpdr.org/seedviewer.cgi?page=FunctionalRole&role=Acetaldehyde%20dehydrogenase%20(EC%201.2.1.10)&subsystem_name=Fermentations:_Lactate) |
| Carbohydrates | Fermentation | [Fermentations: Lactate](https://rast.nmpdr.org/seedviewer.cgi?page=Subsystems&subsystem=Fermentations:_Lactate&organism=549.408) | [Xylulose-5-phosphate phosphoketolase (EC 4.1.2.9)](https://rast.nmpdr.org/seedviewer.cgi?page=FunctionalRole&role=Xylulose-5-phosphate%20phosphoketolase%20(EC%204.1.2.9)&subsystem_name=Fermentations:_Lactate) |
| Carbohydrates | Fermentation | [Fermentations: Lactate](https://rast.nmpdr.org/seedviewer.cgi?page=Subsystems&subsystem=Fermentations:_Lactate&organism=549.408) | [Fructose-6-phosphate phosphoketolase (EC 4.1.2.22)](https://rast.nmpdr.org/seedviewer.cgi?page=FunctionalRole&role=Fructose-6-phosphate%20phosphoketolase%20(EC%204.1.2.22)&subsystem_name=Fermentations:_Lactate) |
| Carbohydrates | Fermentation | [Fermentations: Lactate](https://rast.nmpdr.org/seedviewer.cgi?page=Subsystems&subsystem=Fermentations:_Lactate&organism=549.408) | [Phosphate acetyltransferase (EC 2.3.1.8)](https://rast.nmpdr.org/seedviewer.cgi?page=FunctionalRole&role=Phosphate%20acetyltransferase%20(EC%202.3.1.8)&subsystem_name=Fermentations:_Lactate) |
| Carbohydrates | Fermentation | [Fermentations: Lactate](https://rast.nmpdr.org/seedviewer.cgi?page=Subsystems&subsystem=Fermentations:_Lactate&organism=549.408) | [Acetate kinase (EC 2.7.2.1)](https://rast.nmpdr.org/seedviewer.cgi?page=FunctionalRole&role=Acetate%20kinase%20(EC%202.7.2.1)&subsystem_name=Fermentations:_Lactate) |
| Carbohydrates | Fermentation | [Acetyl-CoA fermentation to Butyrate](https://rast.nmpdr.org/seedviewer.cgi?page=Subsystems&subsystem=Acetyl-CoA_fermentation_to_Butyrate&organism=549.408) | [3-hydroxybutyryl-CoA epimerase (EC 5.1.2.3)](https://rast.nmpdr.org/seedviewer.cgi?page=FunctionalRole&role=3-hydroxybutyryl-CoA%20epimerase%20(EC%205.1.2.3)&subsystem_name=Acetyl-CoA_fermentation_to_Butyrate) |
| Carbohydrates | Fermentation | [Acetyl-CoA fermentation to Butyrate](https://rast.nmpdr.org/seedviewer.cgi?page=Subsystems&subsystem=Acetyl-CoA_fermentation_to_Butyrate&organism=549.408) | [Enoyl-CoA hydratase (EC 4.2.1.17)](https://rast.nmpdr.org/seedviewer.cgi?page=FunctionalRole&role=Enoyl-CoA%20hydratase%20(EC%204.2.1.17)&subsystem_name=Acetyl-CoA_fermentation_to_Butyrate) |
| Carbohydrates | Fermentation | [Acetyl-CoA fermentation to Butyrate](https://rast.nmpdr.org/seedviewer.cgi?page=Subsystems&subsystem=Acetyl-CoA_fermentation_to_Butyrate&organism=549.408) | [3-hydroxyacyl-CoA dehydrogenase (EC 1.1.1.35)](https://rast.nmpdr.org/seedviewer.cgi?page=FunctionalRole&role=3-hydroxyacyl-CoA%20dehydrogenase%20(EC%201.1.1.35)&subsystem_name=Acetyl-CoA_fermentation_to_Butyrate) |
| Carbohydrates | Fermentation | [Acetyl-CoA fermentation to Butyrate](https://rast.nmpdr.org/seedviewer.cgi?page=Subsystems&subsystem=Acetyl-CoA_fermentation_to_Butyrate&organism=549.408) | [Acetyl-CoA acetyltransferase (EC 2.3.1.9)](https://rast.nmpdr.org/seedviewer.cgi?page=FunctionalRole&role=Acetyl-CoA%20acetyltransferase%20(EC%202.3.1.9)&subsystem_name=Acetyl-CoA_fermentation_to_Butyrate) |
| Carbohydrates | Fermentation | [Acetoin, butanediol metabolism](https://rast.nmpdr.org/seedviewer.cgi?page=Subsystems&subsystem=Acetoin,_butanediol_metabolism&organism=549.408) | [Transcriptional activator of acetoin dehydrogenase operon AcoR](https://rast.nmpdr.org/seedviewer.cgi?page=FunctionalRole&role=Transcriptional%20activator%20of%20acetoin%20dehydrogenase%20operon%20AcoR&subsystem_name=Acetoin,_butanediol_metabolism) |
| Carbohydrates | Fermentation | [Acetoin, butanediol metabolism](https://rast.nmpdr.org/seedviewer.cgi?page=Subsystems&subsystem=Acetoin,_butanediol_metabolism&organism=549.408) | [Alpha-acetolactate decarboxylase (EC 4.1.1.5)](https://rast.nmpdr.org/seedviewer.cgi?page=FunctionalRole&role=Alpha-acetolactate%20decarboxylase%20(EC%204.1.1.5)&subsystem_name=Acetoin,_butanediol_metabolism) |
| Carbohydrates | Fermentation | [Acetoin, butanediol metabolism](https://rast.nmpdr.org/seedviewer.cgi?page=Subsystems&subsystem=Acetoin,_butanediol_metabolism&organism=549.408) | [Acetolactate synthase large subunit (EC 2.2.1.6)](https://rast.nmpdr.org/seedviewer.cgi?page=FunctionalRole&role=Acetolactate%20synthase%20large%20subunit%20(EC%202.2.1.6)&subsystem_name=Acetoin,_butanediol_metabolism) |
| Carbohydrates | Fermentation | [Acetoin, butanediol metabolism](https://rast.nmpdr.org/seedviewer.cgi?page=Subsystems&subsystem=Acetoin,_butanediol_metabolism&organism=549.408) | [Acetolactate synthase small subunit (EC 2.2.1.6)](https://rast.nmpdr.org/seedviewer.cgi?page=FunctionalRole&role=Acetolactate%20synthase%20small%20subunit%20(EC%202.2.1.6)&subsystem_name=Acetoin,_butanediol_metabolism) |
| Carbohydrates | Fermentation | [Acetoin, butanediol metabolism](https://rast.nmpdr.org/seedviewer.cgi?page=Subsystems&subsystem=Acetoin,_butanediol_metabolism&organism=549.408) | [Acetolactate synthase, catabolic (EC 2.2.1.6)](https://rast.nmpdr.org/seedviewer.cgi?page=FunctionalRole&role=Acetolactate%20synthase,%20catabolic%20(EC%202.2.1.6)&subsystem_name=Acetoin,_butanediol_metabolism) |
| Carbohydrates | Fermentation | [Acetoin, butanediol metabolism](https://rast.nmpdr.org/seedviewer.cgi?page=Subsystems&subsystem=Acetoin,_butanediol_metabolism&organism=549.408) | [2,3-butanediol dehydrogenase, S-alcohol forming, (R)-acetoin-specific (EC 1.1.1.4)](https://rast.nmpdr.org/seedviewer.cgi?page=FunctionalRole&role=2,3-butanediol%20dehydrogenase,%20S-alcohol%20forming,%20(R)-acetoin-specific%20(EC%201.1.1.4)&subsystem_name=Acetoin,_butanediol_metabolism) |
| Carbohydrates | Sugar alcohols | [Glycerol and Glycerol-3-phosphate Uptake and Utilization](https://rast.nmpdr.org/seedviewer.cgi?page=Subsystems&subsystem=Glycerol_and_Glycerol-3-phosphate_Uptake_and_Utilization&organism=549.408) | [Glycerol-3-phosphate ABC transporter, permease protein UgpE (TC 3.A.1.1.3)](https://rast.nmpdr.org/seedviewer.cgi?page=FunctionalRole&role=Glycerol-3-phosphate%20ABC%20transporter,%20permease%20protein%20UgpE%20(TC%203.A.1.1.3)&subsystem_name=Glycerol_and_Glycerol-3-phosphate_Uptake_and_Utilization) |
| Carbohydrates | Sugar alcohols | [Glycerol and Glycerol-3-phosphate Uptake and Utilization](https://rast.nmpdr.org/seedviewer.cgi?page=Subsystems&subsystem=Glycerol_and_Glycerol-3-phosphate_Uptake_and_Utilization&organism=549.408) | [Glycerol-3-phosphate dehydrogenase [NAD(P)+] (EC 1.1.1.94)](https://rast.nmpdr.org/seedviewer.cgi?page=FunctionalRole&role=Glycerol-3-phosphate%20dehydrogenase%20%5bNAD(P)+%5d%20(EC%201.1.1.94)&subsystem_name=Glycerol_and_Glycerol-3-phosphate_Uptake_and_Utilization) |
| Carbohydrates | Sugar alcohols | [Glycerol and Glycerol-3-phosphate Uptake and Utilization](https://rast.nmpdr.org/seedviewer.cgi?page=Subsystems&subsystem=Glycerol_and_Glycerol-3-phosphate_Uptake_and_Utilization&organism=549.408) | [Glycerol-3-phosphate ABC transporter, permease protein UgpA (TC 3.A.1.1.3)](https://rast.nmpdr.org/seedviewer.cgi?page=FunctionalRole&role=Glycerol-3-phosphate%20ABC%20transporter,%20permease%20protein%20UgpA%20(TC%203.A.1.1.3)&subsystem_name=Glycerol_and_Glycerol-3-phosphate_Uptake_and_Utilization) |
| Carbohydrates | Sugar alcohols | [Glycerol and Glycerol-3-phosphate Uptake and Utilization](https://rast.nmpdr.org/seedviewer.cgi?page=Subsystems&subsystem=Glycerol_and_Glycerol-3-phosphate_Uptake_and_Utilization&organism=549.408) | [Glycerol kinase (EC 2.7.1.30)](https://rast.nmpdr.org/seedviewer.cgi?page=FunctionalRole&role=Glycerol%20kinase%20(EC%202.7.1.30)&subsystem_name=Glycerol_and_Glycerol-3-phosphate_Uptake_and_Utilization) |
| Carbohydrates | Sugar alcohols | [Glycerol and Glycerol-3-phosphate Uptake and Utilization](https://rast.nmpdr.org/seedviewer.cgi?page=Subsystems&subsystem=Glycerol_and_Glycerol-3-phosphate_Uptake_and_Utilization&organism=549.408) | [GlpG protein (membrane protein of glp regulon)](https://rast.nmpdr.org/seedviewer.cgi?page=FunctionalRole&role=GlpG%20protein%20(membrane%20protein%20of%20glp%20regulon)&subsystem_name=Glycerol_and_Glycerol-3-phosphate_Uptake_and_Utilization) |
| Carbohydrates | Sugar alcohols | [Glycerol and Glycerol-3-phosphate Uptake and Utilization](https://rast.nmpdr.org/seedviewer.cgi?page=Subsystems&subsystem=Glycerol_and_Glycerol-3-phosphate_Uptake_and_Utilization&organism=549.408) | [Glycerol-3-phosphate ABC transporter, periplasmic glycerol-3-phosphate-binding protein (TC 3.A.1.1.3)](https://rast.nmpdr.org/seedviewer.cgi?page=FunctionalRole&role=Glycerol-3-phosphate%20ABC%20transporter,%20periplasmic%20glycerol-3-phosphate-binding%20protein%20(TC%203.A.1.1.3)&subsystem_name=Glycerol_and_Glycerol-3-phosphate_Uptake_and_Utilization) |
| Carbohydrates | Sugar alcohols | [Glycerol and Glycerol-3-phosphate Uptake and Utilization](https://rast.nmpdr.org/seedviewer.cgi?page=Subsystems&subsystem=Glycerol_and_Glycerol-3-phosphate_Uptake_and_Utilization&organism=549.408) | [Thiosulfate sulfurtransferase GlpE (EC 2.8.1.1)](https://rast.nmpdr.org/seedviewer.cgi?page=FunctionalRole&role=Thiosulfate%20sulfurtransferase%20GlpE%20(EC%202.8.1.1)&subsystem_name=Glycerol_and_Glycerol-3-phosphate_Uptake_and_Utilization) |
| Carbohydrates | Sugar alcohols | [Glycerol and Glycerol-3-phosphate Uptake and Utilization](https://rast.nmpdr.org/seedviewer.cgi?page=Subsystems&subsystem=Glycerol_and_Glycerol-3-phosphate_Uptake_and_Utilization&organism=549.408) | [Glycerophosphoryl diester phosphodiesterase (EC 3.1.4.46)](https://rast.nmpdr.org/seedviewer.cgi?page=FunctionalRole&role=Glycerophosphoryl%20diester%20phosphodiesterase%20(EC%203.1.4.46)&subsystem_name=Glycerol_and_Glycerol-3-phosphate_Uptake_and_Utilization) |
| Carbohydrates | Sugar alcohols | [Glycerol and Glycerol-3-phosphate Uptake and Utilization](https://rast.nmpdr.org/seedviewer.cgi?page=Subsystems&subsystem=Glycerol_and_Glycerol-3-phosphate_Uptake_and_Utilization&organism=549.408) | [Glycerol-3-phosphate transporter](https://rast.nmpdr.org/seedviewer.cgi?page=FunctionalRole&role=Glycerol-3-phosphate%20transporter&subsystem_name=Glycerol_and_Glycerol-3-phosphate_Uptake_and_Utilization) |
| Carbohydrates | Sugar alcohols | [Glycerol and Glycerol-3-phosphate Uptake and Utilization](https://rast.nmpdr.org/seedviewer.cgi?page=Subsystems&subsystem=Glycerol_and_Glycerol-3-phosphate_Uptake_and_Utilization&organism=549.408) | [Aerobic glycerol-3-phosphate dehydrogenase (EC 1.1.5.3)](https://rast.nmpdr.org/seedviewer.cgi?page=FunctionalRole&role=Aerobic%20glycerol-3-phosphate%20dehydrogenase%20(EC%201.1.5.3)&subsystem_name=Glycerol_and_Glycerol-3-phosphate_Uptake_and_Utilization) |
| Carbohydrates | Sugar alcohols | [Glycerol and Glycerol-3-phosphate Uptake and Utilization](https://rast.nmpdr.org/seedviewer.cgi?page=Subsystems&subsystem=Glycerol_and_Glycerol-3-phosphate_Uptake_and_Utilization&organism=549.408) | [Glycerol uptake facilitator protein](https://rast.nmpdr.org/seedviewer.cgi?page=FunctionalRole&role=Glycerol%20uptake%20facilitator%20protein&subsystem_name=Glycerol_and_Glycerol-3-phosphate_Uptake_and_Utilization) |
| Carbohydrates | Sugar alcohols | [Glycerol and Glycerol-3-phosphate Uptake and Utilization](https://rast.nmpdr.org/seedviewer.cgi?page=Subsystems&subsystem=Glycerol_and_Glycerol-3-phosphate_Uptake_and_Utilization&organism=549.408) | [Glycerol-3-phosphate ABC transporter, ATP-binding protein UgpC (TC 3.A.1.1.3)](https://rast.nmpdr.org/seedviewer.cgi?page=FunctionalRole&role=Glycerol-3-phosphate%20ABC%20transporter,%20ATP-binding%20protein%20UgpC%20(TC%203.A.1.1.3)&subsystem_name=Glycerol_and_Glycerol-3-phosphate_Uptake_and_Utilization) |
| Carbohydrates | Sugar alcohols | [Mannitol Utilization](https://rast.nmpdr.org/seedviewer.cgi?page=Subsystems&subsystem=Mannitol_Utilization&organism=549.408) | [Phosphoenolpyruvate-protein phosphotransferase of PTS system (EC 2.7.3.9)](https://rast.nmpdr.org/seedviewer.cgi?page=FunctionalRole&role=Phosphoenolpyruvate-protein%20phosphotransferase%20of%20PTS%20system%20(EC%202.7.3.9)&subsystem_name=Mannitol_Utilization) |
| Carbohydrates | Sugar alcohols | [Mannitol Utilization](https://rast.nmpdr.org/seedviewer.cgi?page=Subsystems&subsystem=Mannitol_Utilization&organism=549.408) | [PTS system, mannitol-specific IIC component (EC 2.7.1.69)](https://rast.nmpdr.org/seedviewer.cgi?page=FunctionalRole&role=PTS%20system,%20mannitol-specific%20IIC%20component%20(EC%202.7.1.69)&subsystem_name=Mannitol_Utilization) |
| Carbohydrates | Sugar alcohols | [Mannitol Utilization](https://rast.nmpdr.org/seedviewer.cgi?page=Subsystems&subsystem=Mannitol_Utilization&organism=549.408) | [Mannitol-1-phosphate 5-dehydrogenase (EC 1.1.1.17)](https://rast.nmpdr.org/seedviewer.cgi?page=FunctionalRole&role=Mannitol-1-phosphate%205-dehydrogenase%20(EC%201.1.1.17)&subsystem_name=Mannitol_Utilization) |
| Carbohydrates | Sugar alcohols | [Mannitol Utilization](https://rast.nmpdr.org/seedviewer.cgi?page=Subsystems&subsystem=Mannitol_Utilization&organism=549.408) | [Transcriptional regulator of mannitol utilization, DeoR family protein](https://rast.nmpdr.org/seedviewer.cgi?page=FunctionalRole&role=Transcriptional%20regulator%20of%20mannitol%20utilization,%20DeoR%20family%20protein&subsystem_name=Mannitol_Utilization) |
| Carbohydrates | Sugar alcohols | [Mannitol Utilization](https://rast.nmpdr.org/seedviewer.cgi?page=Subsystems&subsystem=Mannitol_Utilization&organism=549.408) | [PTS system, mannitol-specific IIB component (EC 2.7.1.69)](https://rast.nmpdr.org/seedviewer.cgi?page=FunctionalRole&role=PTS%20system,%20mannitol-specific%20IIB%20component%20(EC%202.7.1.69)&subsystem_name=Mannitol_Utilization) |
| Carbohydrates | Sugar alcohols | [Mannitol Utilization](https://rast.nmpdr.org/seedviewer.cgi?page=Subsystems&subsystem=Mannitol_Utilization&organism=549.408) | [Mannitol operon repressor](https://rast.nmpdr.org/seedviewer.cgi?page=FunctionalRole&role=Mannitol%20operon%20repressor&subsystem_name=Mannitol_Utilization) |
| Carbohydrates | Sugar alcohols | [Mannitol Utilization](https://rast.nmpdr.org/seedviewer.cgi?page=Subsystems&subsystem=Mannitol_Utilization&organism=549.408) | [Fructokinase (EC 2.7.1.4)](https://rast.nmpdr.org/seedviewer.cgi?page=FunctionalRole&role=Fructokinase%20(EC%202.7.1.4)&subsystem_name=Mannitol_Utilization) |
| Carbohydrates | Sugar alcohols | [Inositol catabolism](https://rast.nmpdr.org/seedviewer.cgi?page=Subsystems&subsystem=Inositol_catabolism&organism=549.408) | [Inositol transport system sugar-binding protein](https://rast.nmpdr.org/seedviewer.cgi?page=FunctionalRole&role=Inositol%20transport%20system%20sugar-binding%20protein&subsystem_name=Inositol_catabolism) |
| Carbohydrates | Sugar alcohols | [Inositol catabolism](https://rast.nmpdr.org/seedviewer.cgi?page=Subsystems&subsystem=Inositol_catabolism&organism=549.408) | [Inositol transport system ATP-binding protein](https://rast.nmpdr.org/seedviewer.cgi?page=FunctionalRole&role=Inositol%20transport%20system%20ATP-binding%20protein&subsystem_name=Inositol_catabolism) |
| Carbohydrates | Sugar alcohols | [Inositol catabolism](https://rast.nmpdr.org/seedviewer.cgi?page=Subsystems&subsystem=Inositol_catabolism&organism=549.408) | [Major myo-inositol transporter IolT](https://rast.nmpdr.org/seedviewer.cgi?page=FunctionalRole&role=Major%20myo-inositol%20transporter%20IolT&subsystem_name=Inositol_catabolism) |
| Carbohydrates | Sugar alcohols | [Inositol catabolism](https://rast.nmpdr.org/seedviewer.cgi?page=Subsystems&subsystem=Inositol_catabolism&organism=549.408) | [Myo-inositol 2-dehydrogenase 2 (EC 1.1.1.18)](https://rast.nmpdr.org/seedviewer.cgi?page=FunctionalRole&role=Myo-inositol%202-dehydrogenase%202%20(EC%201.1.1.18)&subsystem_name=Inositol_catabolism) |
| Carbohydrates | Sugar alcohols | [Inositol catabolism](https://rast.nmpdr.org/seedviewer.cgi?page=Subsystems&subsystem=Inositol_catabolism&organism=549.408) | [5-deoxy-glucuronate isomerase (EC 5.3.1.-)](https://rast.nmpdr.org/seedviewer.cgi?page=FunctionalRole&role=5-deoxy-glucuronate%20isomerase%20(EC%205.3.1.-)&subsystem_name=Inositol_catabolism) |
| Carbohydrates | Sugar alcohols | [Inositol catabolism](https://rast.nmpdr.org/seedviewer.cgi?page=Subsystems&subsystem=Inositol_catabolism&organism=549.408) | [Epi-inositol hydrolase (EC 3.7.1.-)](https://rast.nmpdr.org/seedviewer.cgi?page=FunctionalRole&role=Epi-inositol%20hydrolase%20(EC%203.7.1.-)&subsystem_name=Inositol_catabolism) |
| Carbohydrates | Sugar alcohols | [Inositol catabolism](https://rast.nmpdr.org/seedviewer.cgi?page=Subsystems&subsystem=Inositol_catabolism&organism=549.408) | [Inositol transport system permease protein](https://rast.nmpdr.org/seedviewer.cgi?page=FunctionalRole&role=Inositol%20transport%20system%20permease%20protein&subsystem_name=Inositol_catabolism) |
| Carbohydrates | Sugar alcohols | [Inositol catabolism](https://rast.nmpdr.org/seedviewer.cgi?page=Subsystems&subsystem=Inositol_catabolism&organism=549.408) | [Myo-inositol 2-dehydrogenase 1 (EC 1.1.1.18)](https://rast.nmpdr.org/seedviewer.cgi?page=FunctionalRole&role=Myo-inositol%202-dehydrogenase%201%20(EC%201.1.1.18)&subsystem_name=Inositol_catabolism) |
| Carbohydrates | Sugar alcohols | [Inositol catabolism](https://rast.nmpdr.org/seedviewer.cgi?page=Subsystems&subsystem=Inositol_catabolism&organism=549.408) | [Predicted transcriptional regulator of the myo-inositol catabolic operon](https://rast.nmpdr.org/seedviewer.cgi?page=FunctionalRole&role=Predicted%20transcriptional%20regulator%20of%20the%20myo-inositol%20catabolic%20operon&subsystem_name=Inositol_catabolism) |
| Carbohydrates | Sugar alcohols | [Inositol catabolism](https://rast.nmpdr.org/seedviewer.cgi?page=Subsystems&subsystem=Inositol_catabolism&organism=549.408) | [Glyceraldehyde-3-phosphate ketol-isomerase (EC 5.3.1.1)](https://rast.nmpdr.org/seedviewer.cgi?page=FunctionalRole&role=Glyceraldehyde-3-phosphate%20ketol-isomerase%20(EC%205.3.1.1)&subsystem_name=Inositol_catabolism) |
| Carbohydrates | Sugar alcohols | [Inositol catabolism](https://rast.nmpdr.org/seedviewer.cgi?page=Subsystems&subsystem=Inositol_catabolism&organism=549.408) | [uncharacterized domain](https://rast.nmpdr.org/seedviewer.cgi?page=FunctionalRole&role=uncharacterized%20domain&subsystem_name=Inositol_catabolism) |
| Carbohydrates | Sugar alcohols | [Inositol catabolism](https://rast.nmpdr.org/seedviewer.cgi?page=Subsystems&subsystem=Inositol_catabolism&organism=549.408) | [Inosose isomerase (EC 5.3.99.-)](https://rast.nmpdr.org/seedviewer.cgi?page=FunctionalRole&role=Inosose%20isomerase%20(EC%205.3.99.-)&subsystem_name=Inositol_catabolism) |
| Carbohydrates | Sugar alcohols | [Inositol catabolism](https://rast.nmpdr.org/seedviewer.cgi?page=Subsystems&subsystem=Inositol_catabolism&organism=549.408) | [Inosose dehydratase (EC 4.2.1.44)](https://rast.nmpdr.org/seedviewer.cgi?page=FunctionalRole&role=Inosose%20dehydratase%20(EC%204.2.1.44)&subsystem_name=Inositol_catabolism) |
| Carbohydrates | Sugar alcohols | [Inositol catabolism](https://rast.nmpdr.org/seedviewer.cgi?page=Subsystems&subsystem=Inositol_catabolism&organism=549.408) | [Methylmalonate-semialdehyde dehydrogenase [inositol] (EC 1.2.1.27)](https://rast.nmpdr.org/seedviewer.cgi?page=FunctionalRole&role=Methylmalonate-semialdehyde%20dehydrogenase%20%5binositol%5d%20(EC%201.2.1.27)&subsystem_name=Inositol_catabolism) |
| Carbohydrates | Sugar alcohols | [Inositol catabolism](https://rast.nmpdr.org/seedviewer.cgi?page=Subsystems&subsystem=Inositol_catabolism&organism=549.408) | [Myo-inositol 2-dehydrogenase (EC 1.1.1.18)](https://rast.nmpdr.org/seedviewer.cgi?page=FunctionalRole&role=Myo-inositol%202-dehydrogenase%20(EC%201.1.1.18)&subsystem_name=Inositol_catabolism) |
| Carbohydrates | Sugar alcohols | [Inositol catabolism](https://rast.nmpdr.org/seedviewer.cgi?page=Subsystems&subsystem=Inositol_catabolism&organism=549.408) | [5-keto-2-deoxygluconokinase (EC 2.7.1.92)](https://rast.nmpdr.org/seedviewer.cgi?page=FunctionalRole&role=5-keto-2-deoxygluconokinase%20(EC%202.7.1.92)&subsystem_name=Inositol_catabolism) |
| Carbohydrates | Carbohydrates - no subcategory | [Carbon storage regulator](https://rast.nmpdr.org/seedviewer.cgi?page=Subsystems&subsystem=Carbon_storage_regulator&organism=549.408) | [Carbon storage regulator](https://rast.nmpdr.org/seedviewer.cgi?page=FunctionalRole&role=Carbon%20storage%20regulator&subsystem_name=Carbon_storage_regulator) |
| Carbohydrates | Polysaccharides | [Glycogen metabolism](https://rast.nmpdr.org/seedviewer.cgi?page=Subsystems&subsystem=Glycogen_metabolism&organism=549.408) | [Maltodextrin phosphorylase (EC 2.4.1.1)](https://rast.nmpdr.org/seedviewer.cgi?page=FunctionalRole&role=Maltodextrin%20phosphorylase%20(EC%202.4.1.1)&subsystem_name=Glycogen_metabolism) |
| Carbohydrates | Polysaccharides | [Glycogen metabolism](https://rast.nmpdr.org/seedviewer.cgi?page=Subsystems&subsystem=Glycogen_metabolism&organism=549.408) | [Glucose-1-phosphate adenylyltransferase (EC 2.7.7.27)](https://rast.nmpdr.org/seedviewer.cgi?page=FunctionalRole&role=Glucose-1-phosphate%20adenylyltransferase%20(EC%202.7.7.27)&subsystem_name=Glycogen_metabolism) |
| Carbohydrates | Polysaccharides | [Glycogen metabolism](https://rast.nmpdr.org/seedviewer.cgi?page=Subsystems&subsystem=Glycogen_metabolism&organism=549.408) | [4-alpha-glucanotransferase (amylomaltase) (EC 2.4.1.25)](https://rast.nmpdr.org/seedviewer.cgi?page=FunctionalRole&role=4-alpha-glucanotransferase%20(amylomaltase)%20(EC%202.4.1.25)&subsystem_name=Glycogen_metabolism) |
| Carbohydrates | Polysaccharides | [Glycogen metabolism](https://rast.nmpdr.org/seedviewer.cgi?page=Subsystems&subsystem=Glycogen_metabolism&organism=549.408) | [1,4-alpha-glucan (glycogen) branching enzyme, GH-13-type (EC 2.4.1.18)](https://rast.nmpdr.org/seedviewer.cgi?page=FunctionalRole&role=1,4-alpha-glucan%20(glycogen)%20branching%20enzyme,%20GH-13-type%20(EC%202.4.1.18)&subsystem_name=Glycogen_metabolism) |
| Carbohydrates | Polysaccharides | [Glycogen metabolism](https://rast.nmpdr.org/seedviewer.cgi?page=Subsystems&subsystem=Glycogen_metabolism&organism=549.408) | [Glycogen phosphorylase (EC 2.4.1.1)](https://rast.nmpdr.org/seedviewer.cgi?page=FunctionalRole&role=Glycogen%20phosphorylase%20(EC%202.4.1.1)&subsystem_name=Glycogen_metabolism) |
| Carbohydrates | Polysaccharides | [Glycogen metabolism](https://rast.nmpdr.org/seedviewer.cgi?page=Subsystems&subsystem=Glycogen_metabolism&organism=549.408) | [Glycogen debranching enzyme (EC 3.2.1.-)](https://rast.nmpdr.org/seedviewer.cgi?page=FunctionalRole&role=Glycogen%20debranching%20enzyme%20(EC%203.2.1.-)&subsystem_name=Glycogen_metabolism) |
| Carbohydrates | Polysaccharides | [Glycogen metabolism](https://rast.nmpdr.org/seedviewer.cgi?page=Subsystems&subsystem=Glycogen_metabolism&organism=549.408) | [Glycogen synthase, ADP-glucose transglucosylase (EC 2.4.1.21)](https://rast.nmpdr.org/seedviewer.cgi?page=FunctionalRole&role=Glycogen%20synthase,%20ADP-glucose%20transglucosylase%20(EC%202.4.1.21)&subsystem_name=Glycogen_metabolism) |
| Carbohydrates | Polysaccharides | [Alpha-Amylase locus in Streptocococcus](https://rast.nmpdr.org/seedviewer.cgi?page=Subsystems&subsystem=Alpha-Amylase_locus_in_Streptocococcus&organism=549.408) | [Maltose/maltodextrin ABC transporter, substrate binding periplasmic protein MalE](https://rast.nmpdr.org/seedviewer.cgi?page=FunctionalRole&role=Maltose/maltodextrin%20ABC%20transporter,%20substrate%20binding%20periplasmic%20protein%20MalE&subsystem_name=Alpha-Amylase_locus_in_Streptocococcus) |
| Carbohydrates | Monosaccharides | [Mannose Metabolism](https://rast.nmpdr.org/seedviewer.cgi?page=Subsystems&subsystem=Mannose_Metabolism&organism=549.408) | [Phosphomannomutase (EC 5.4.2.8)](https://rast.nmpdr.org/seedviewer.cgi?page=FunctionalRole&role=Phosphomannomutase%20(EC%205.4.2.8)&subsystem_name=Mannose_Metabolism) |
| Carbohydrates | Monosaccharides | [Mannose Metabolism](https://rast.nmpdr.org/seedviewer.cgi?page=Subsystems&subsystem=Mannose_Metabolism&organism=549.408) | [PTS system, mannose-specific IIB component (EC 2.7.1.69)](https://rast.nmpdr.org/seedviewer.cgi?page=FunctionalRole&role=PTS%20system,%20mannose-specific%20IIB%20component%20(EC%202.7.1.69)&subsystem_name=Mannose_Metabolism) |
| Carbohydrates | Monosaccharides | [Mannose Metabolism](https://rast.nmpdr.org/seedviewer.cgi?page=Subsystems&subsystem=Mannose_Metabolism&organism=549.408) | [Mannose-6-phosphate isomerase (EC 5.3.1.8)](https://rast.nmpdr.org/seedviewer.cgi?page=FunctionalRole&role=Mannose-6-phosphate%20isomerase%20(EC%205.3.1.8)&subsystem_name=Mannose_Metabolism) |
| Carbohydrates | Monosaccharides | [Mannose Metabolism](https://rast.nmpdr.org/seedviewer.cgi?page=Subsystems&subsystem=Mannose_Metabolism&organism=549.408) | [Mannose-1-phosphate guanylyltransferase (GDP) (EC 2.7.7.22)](https://rast.nmpdr.org/seedviewer.cgi?page=FunctionalRole&role=Mannose-1-phosphate%20guanylyltransferase%20(GDP)%20(EC%202.7.7.22)&subsystem_name=Mannose_Metabolism) |
| Carbohydrates | Monosaccharides | [2-Ketogluconate Utilization](https://rast.nmpdr.org/seedviewer.cgi?page=Subsystems&subsystem=2-Ketogluconate_Utilization&organism=549.408) | [Epimerase KguE](https://rast.nmpdr.org/seedviewer.cgi?page=FunctionalRole&role=Epimerase%20KguE&subsystem_name=2-Ketogluconate_Utilization) |
| Carbohydrates | Monosaccharides | [2-Ketogluconate Utilization](https://rast.nmpdr.org/seedviewer.cgi?page=Subsystems&subsystem=2-Ketogluconate_Utilization&organism=549.408) | [2-ketogluconate transporter](https://rast.nmpdr.org/seedviewer.cgi?page=FunctionalRole&role=2-ketogluconate%20transporter&subsystem_name=2-Ketogluconate_Utilization) |
| Carbohydrates | Monosaccharides | [2-Ketogluconate Utilization](https://rast.nmpdr.org/seedviewer.cgi?page=Subsystems&subsystem=2-Ketogluconate_Utilization&organism=549.408) | [2-ketogluconate utilization repressor PtxS](https://rast.nmpdr.org/seedviewer.cgi?page=FunctionalRole&role=2-ketogluconate%20utilization%20repressor%20PtxS&subsystem_name=2-Ketogluconate_Utilization) |
| Carbohydrates | Monosaccharides | [2-Ketogluconate Utilization](https://rast.nmpdr.org/seedviewer.cgi?page=Subsystems&subsystem=2-Ketogluconate_Utilization&organism=549.408) | [2-ketogluconate kinase (EC 2.7.1.13)](https://rast.nmpdr.org/seedviewer.cgi?page=FunctionalRole&role=2-ketogluconate%20kinase%20(EC%202.7.1.13)&subsystem_name=2-Ketogluconate_Utilization) |
| Carbohydrates | Monosaccharides | [Hexose Phosphate Uptake System](https://rast.nmpdr.org/seedviewer.cgi?page=Subsystems&subsystem=Hexose_Phosphate_Uptake_System&organism=549.408) | [Sensor histidine protein kinase UhpB, glucose-6-phosphate specific (EC 2.7.13.3)](https://rast.nmpdr.org/seedviewer.cgi?page=FunctionalRole&role=Sensor%20histidine%20protein%20kinase%20UhpB,%20glucose-6-phosphate%20specific%20(EC%202.7.13.3)&subsystem_name=Hexose_Phosphate_Uptake_System) |
| Carbohydrates | Monosaccharides | [Hexose Phosphate Uptake System](https://rast.nmpdr.org/seedviewer.cgi?page=Subsystems&subsystem=Hexose_Phosphate_Uptake_System&organism=549.408) | [Transcriptional regulatory protein UhpA](https://rast.nmpdr.org/seedviewer.cgi?page=FunctionalRole&role=Transcriptional%20regulatory%20protein%20UhpA&subsystem_name=Hexose_Phosphate_Uptake_System) |
| Carbohydrates | Monosaccharides | [Hexose Phosphate Uptake System](https://rast.nmpdr.org/seedviewer.cgi?page=Subsystems&subsystem=Hexose_Phosphate_Uptake_System&organism=549.408) | [Homolog of fucose/glucose/galactose permeases](https://rast.nmpdr.org/seedviewer.cgi?page=FunctionalRole&role=Homolog%20of%20fucose/glucose/galactose%20permeases&subsystem_name=Hexose_Phosphate_Uptake_System) |
| Carbohydrates | Monosaccharides | [Hexose Phosphate Uptake System](https://rast.nmpdr.org/seedviewer.cgi?page=Subsystems&subsystem=Hexose_Phosphate_Uptake_System&organism=549.408) | [Hexose phosphate uptake regulatory protein UhpC](https://rast.nmpdr.org/seedviewer.cgi?page=FunctionalRole&role=Hexose%20phosphate%20uptake%20regulatory%20protein%20UhpC&subsystem_name=Hexose_Phosphate_Uptake_System) |
| Carbohydrates | Monosaccharides | [Hexose Phosphate Uptake System](https://rast.nmpdr.org/seedviewer.cgi?page=Subsystems&subsystem=Hexose_Phosphate_Uptake_System&organism=549.408) | [Hexose phosphate transport protein UhpT](https://rast.nmpdr.org/seedviewer.cgi?page=FunctionalRole&role=Hexose%20phosphate%20transport%20protein%20UhpT&subsystem_name=Hexose_Phosphate_Uptake_System) |
| Carbohydrates | Monosaccharides | [Deoxyribose and Deoxynucleoside Catabolism](https://rast.nmpdr.org/seedviewer.cgi?page=Subsystems&subsystem=Deoxyribose_and_Deoxynucleoside_Catabolism&organism=549.408) | [Purine nucleoside phosphorylase (EC 2.4.2.1)](https://rast.nmpdr.org/seedviewer.cgi?page=FunctionalRole&role=Purine%20nucleoside%20phosphorylase%20(EC%202.4.2.1)&subsystem_name=Deoxyribose_and_Deoxynucleoside_Catabolism) |
| Carbohydrates | Monosaccharides | [Deoxyribose and Deoxynucleoside Catabolism](https://rast.nmpdr.org/seedviewer.cgi?page=Subsystems&subsystem=Deoxyribose_and_Deoxynucleoside_Catabolism&organism=549.408) | [Ribokinase (EC 2.7.1.15)](https://rast.nmpdr.org/seedviewer.cgi?page=FunctionalRole&role=Ribokinase%20(EC%202.7.1.15)&subsystem_name=Deoxyribose_and_Deoxynucleoside_Catabolism) |
| Carbohydrates | Monosaccharides | [Deoxyribose and Deoxynucleoside Catabolism](https://rast.nmpdr.org/seedviewer.cgi?page=Subsystems&subsystem=Deoxyribose_and_Deoxynucleoside_Catabolism&organism=549.408) | [Phosphopentomutase (EC 5.4.2.7)](https://rast.nmpdr.org/seedviewer.cgi?page=FunctionalRole&role=Phosphopentomutase%20(EC%205.4.2.7)&subsystem_name=Deoxyribose_and_Deoxynucleoside_Catabolism) |
| Carbohydrates | Monosaccharides | [Deoxyribose and Deoxynucleoside Catabolism](https://rast.nmpdr.org/seedviewer.cgi?page=Subsystems&subsystem=Deoxyribose_and_Deoxynucleoside_Catabolism&organism=549.408) | [Deoxyribose operon repressor, DeoR family](https://rast.nmpdr.org/seedviewer.cgi?page=FunctionalRole&role=Deoxyribose%20operon%20repressor,%20DeoR%20family&subsystem_name=Deoxyribose_and_Deoxynucleoside_Catabolism) |
| Carbohydrates | Monosaccharides | [Deoxyribose and Deoxynucleoside Catabolism](https://rast.nmpdr.org/seedviewer.cgi?page=Subsystems&subsystem=Deoxyribose_and_Deoxynucleoside_Catabolism&organism=549.408) | [Thymidine phosphorylase (EC 2.4.2.4)](https://rast.nmpdr.org/seedviewer.cgi?page=FunctionalRole&role=Thymidine%20phosphorylase%20(EC%202.4.2.4)&subsystem_name=Deoxyribose_and_Deoxynucleoside_Catabolism) |
| Carbohydrates | Monosaccharides | [Deoxyribose and Deoxynucleoside Catabolism](https://rast.nmpdr.org/seedviewer.cgi?page=Subsystems&subsystem=Deoxyribose_and_Deoxynucleoside_Catabolism&organism=549.408) | [Putative deoxyribonuclease YjjV](https://rast.nmpdr.org/seedviewer.cgi?page=FunctionalRole&role=Putative%20deoxyribonuclease%20YjjV&subsystem_name=Deoxyribose_and_Deoxynucleoside_Catabolism) |
| Carbohydrates | Monosaccharides | [Deoxyribose and Deoxynucleoside Catabolism](https://rast.nmpdr.org/seedviewer.cgi?page=Subsystems&subsystem=Deoxyribose_and_Deoxynucleoside_Catabolism&organism=549.408) | [Deoxyribose-phosphate aldolase (EC 4.1.2.4)](https://rast.nmpdr.org/seedviewer.cgi?page=FunctionalRole&role=Deoxyribose-phosphate%20aldolase%20(EC%204.1.2.4)&subsystem_name=Deoxyribose_and_Deoxynucleoside_Catabolism) |
| Carbohydrates | Monosaccharides | [D-gluconate and ketogluconates metabolism](https://rast.nmpdr.org/seedviewer.cgi?page=Subsystems&subsystem=D-gluconate_and_ketogluconates_metabolism&organism=549.408) | [Gluconate utilization system Gnt-I transcriptional repressor](https://rast.nmpdr.org/seedviewer.cgi?page=FunctionalRole&role=Gluconate%20utilization%20system%20Gnt-I%20transcriptional%20repressor&subsystem_name=D-gluconate_and_ketogluconates_metabolism) |
| Carbohydrates | Monosaccharides | [D-gluconate and ketogluconates metabolism](https://rast.nmpdr.org/seedviewer.cgi?page=Subsystems&subsystem=D-gluconate_and_ketogluconates_metabolism&organism=549.408) | [2-ketogluconate kinase (EC 2.7.1.13)](https://rast.nmpdr.org/seedviewer.cgi?page=FunctionalRole&role=2-ketogluconate%20kinase%20(EC%202.7.1.13)&subsystem_name=D-gluconate_and_ketogluconates_metabolism) |
| Carbohydrates | Monosaccharides | [D-gluconate and ketogluconates metabolism](https://rast.nmpdr.org/seedviewer.cgi?page=Subsystems&subsystem=D-gluconate_and_ketogluconates_metabolism&organism=549.408) | [Glucose dehydrogenase, PQQ-dependent (EC 1.1.5.2)](https://rast.nmpdr.org/seedviewer.cgi?page=FunctionalRole&role=Glucose%20dehydrogenase,%20PQQ-dependent%20(EC%201.1.5.2)&subsystem_name=D-gluconate_and_ketogluconates_metabolism) |
| Carbohydrates | Monosaccharides | [D-gluconate and ketogluconates metabolism](https://rast.nmpdr.org/seedviewer.cgi?page=Subsystems&subsystem=D-gluconate_and_ketogluconates_metabolism&organism=549.408) | [L-idonate 5-dehydrogenase (EC 1.1.1.264)](https://rast.nmpdr.org/seedviewer.cgi?page=FunctionalRole&role=L-idonate%205-dehydrogenase%20(EC%201.1.1.264)&subsystem_name=D-gluconate_and_ketogluconates_metabolism) |
| Carbohydrates | Monosaccharides | [D-gluconate and ketogluconates metabolism](https://rast.nmpdr.org/seedviewer.cgi?page=Subsystems&subsystem=D-gluconate_and_ketogluconates_metabolism&organism=549.408) | [2-ketoaldonate reductase, broad specificity (EC 1.1.1.215) (EC 1.1.1.-)](https://rast.nmpdr.org/seedviewer.cgi?page=FunctionalRole&role=2-ketoaldonate%20reductase,%20broad%20specificity%20(EC%201.1.1.215)%20(EC%201.1.1.-)&subsystem_name=D-gluconate_and_ketogluconates_metabolism) |
| Carbohydrates | Monosaccharides | [D-gluconate and ketogluconates metabolism](https://rast.nmpdr.org/seedviewer.cgi?page=Subsystems&subsystem=D-gluconate_and_ketogluconates_metabolism&organism=549.408) | [6-phosphogluconate dehydrogenase, decarboxylating (EC 1.1.1.44)](https://rast.nmpdr.org/seedviewer.cgi?page=FunctionalRole&role=6-phosphogluconate%20dehydrogenase,%20decarboxylating%20(EC%201.1.1.44)&subsystem_name=D-gluconate_and_ketogluconates_metabolism) |
| Carbohydrates | Monosaccharides | [D-gluconate and ketogluconates metabolism](https://rast.nmpdr.org/seedviewer.cgi?page=Subsystems&subsystem=D-gluconate_and_ketogluconates_metabolism&organism=549.408) | [Gluconate 2-dehydrogenase (EC 1.1.99.3), membrane-bound, gamma subunit](https://rast.nmpdr.org/seedviewer.cgi?page=FunctionalRole&role=Gluconate%202-dehydrogenase%20(EC%201.1.99.3),%20membrane-bound,%20gamma%20subunit&subsystem_name=D-gluconate_and_ketogluconates_metabolism) |
| Carbohydrates | Monosaccharides | [D-gluconate and ketogluconates metabolism](https://rast.nmpdr.org/seedviewer.cgi?page=Subsystems&subsystem=D-gluconate_and_ketogluconates_metabolism&organism=549.408) | [Gluconate 2-dehydrogenase (EC 1.1.99.3), membrane-bound, flavoprotein](https://rast.nmpdr.org/seedviewer.cgi?page=FunctionalRole&role=Gluconate%202-dehydrogenase%20(EC%201.1.99.3),%20membrane-bound,%20flavoprotein&subsystem_name=D-gluconate_and_ketogluconates_metabolism) |
| Carbohydrates | Monosaccharides | [D-gluconate and ketogluconates metabolism](https://rast.nmpdr.org/seedviewer.cgi?page=Subsystems&subsystem=D-gluconate_and_ketogluconates_metabolism&organism=549.408) | [Gluconate permease](https://rast.nmpdr.org/seedviewer.cgi?page=FunctionalRole&role=Gluconate%20permease&subsystem_name=D-gluconate_and_ketogluconates_metabolism) |
| Carbohydrates | Monosaccharides | [D-gluconate and ketogluconates metabolism](https://rast.nmpdr.org/seedviewer.cgi?page=Subsystems&subsystem=D-gluconate_and_ketogluconates_metabolism&organism=549.408) | [High-affinity gluconate transporter GntT](https://rast.nmpdr.org/seedviewer.cgi?page=FunctionalRole&role=High-affinity%20gluconate%20transporter%20GntT&subsystem_name=D-gluconate_and_ketogluconates_metabolism) |
| Carbohydrates | Monosaccharides | [D-gluconate and ketogluconates metabolism](https://rast.nmpdr.org/seedviewer.cgi?page=Subsystems&subsystem=D-gluconate_and_ketogluconates_metabolism&organism=549.408) | [2-dehydro-3-deoxygluconate kinase (EC 2.7.1.45)](https://rast.nmpdr.org/seedviewer.cgi?page=FunctionalRole&role=2-dehydro-3-deoxygluconate%20kinase%20(EC%202.7.1.45)&subsystem_name=D-gluconate_and_ketogluconates_metabolism) |
| Carbohydrates | Monosaccharides | [D-gluconate and ketogluconates metabolism](https://rast.nmpdr.org/seedviewer.cgi?page=Subsystems&subsystem=D-gluconate_and_ketogluconates_metabolism&organism=549.408) | [5-keto-D-gluconate 5-reductase (EC 1.1.1.69)](https://rast.nmpdr.org/seedviewer.cgi?page=FunctionalRole&role=5-keto-D-gluconate%205-reductase%20(EC%201.1.1.69)&subsystem_name=D-gluconate_and_ketogluconates_metabolism) |
| Carbohydrates | Monosaccharides | [D-gluconate and ketogluconates metabolism](https://rast.nmpdr.org/seedviewer.cgi?page=Subsystems&subsystem=D-gluconate_and_ketogluconates_metabolism&organism=549.408) | [Low-affinity gluconate/H+ symporter GntU](https://rast.nmpdr.org/seedviewer.cgi?page=FunctionalRole&role=Low-affinity%20gluconate/H+%20symporter%20GntU&subsystem_name=D-gluconate_and_ketogluconates_metabolism) |
| Carbohydrates | Monosaccharides | [D-gluconate and ketogluconates metabolism](https://rast.nmpdr.org/seedviewer.cgi?page=Subsystems&subsystem=D-gluconate_and_ketogluconates_metabolism&organism=549.408) | [Glucose 1-dehydrogenase (EC 1.1.1.47)](https://rast.nmpdr.org/seedviewer.cgi?page=FunctionalRole&role=Glucose%201-dehydrogenase%20(EC%201.1.1.47)&subsystem_name=D-gluconate_and_ketogluconates_metabolism) |
| Carbohydrates | Monosaccharides | [D-gluconate and ketogluconates metabolism](https://rast.nmpdr.org/seedviewer.cgi?page=Subsystems&subsystem=D-gluconate_and_ketogluconates_metabolism&organism=549.408) | [Gluconate 2-dehydrogenase (EC 1.1.99.3), membrane-bound, cytochrome c](https://rast.nmpdr.org/seedviewer.cgi?page=FunctionalRole&role=Gluconate%202-dehydrogenase%20(EC%201.1.99.3),%20membrane-bound,%20cytochrome%20c&subsystem_name=D-gluconate_and_ketogluconates_metabolism) |
| Carbohydrates | Monosaccharides | [D-gluconate and ketogluconates metabolism](https://rast.nmpdr.org/seedviewer.cgi?page=Subsystems&subsystem=D-gluconate_and_ketogluconates_metabolism&organism=549.408) | [2,5-diketo-D-gluconate reductase A (EC 1.1.1.274)](https://rast.nmpdr.org/seedviewer.cgi?page=FunctionalRole&role=2,5-diketo-D-gluconate%20reductase%20A%20(EC%201.1.1.274)&subsystem_name=D-gluconate_and_ketogluconates_metabolism) |
| Carbohydrates | Monosaccharides | [D-gluconate and ketogluconates metabolism](https://rast.nmpdr.org/seedviewer.cgi?page=Subsystems&subsystem=D-gluconate_and_ketogluconates_metabolism&organism=549.408) | [Gluconokinase (EC 2.7.1.12)](https://rast.nmpdr.org/seedviewer.cgi?page=FunctionalRole&role=Gluconokinase%20(EC%202.7.1.12)&subsystem_name=D-gluconate_and_ketogluconates_metabolism) |
| Carbohydrates | Monosaccharides | [Fructose utilization](https://rast.nmpdr.org/seedviewer.cgi?page=Subsystems&subsystem=Fructose_utilization&organism=549.408) | [Phosphoenolpyruvate-protein phosphotransferase of PTS system (EC 2.7.3.9)](https://rast.nmpdr.org/seedviewer.cgi?page=FunctionalRole&role=Phosphoenolpyruvate-protein%20phosphotransferase%20of%20PTS%20system%20(EC%202.7.3.9)&subsystem_name=Fructose_utilization) |
| Carbohydrates | Monosaccharides | [Fructose utilization](https://rast.nmpdr.org/seedviewer.cgi?page=Subsystems&subsystem=Fructose_utilization&organism=549.408) | [PTS system, fructose-specific IIC component (EC 2.7.1.69)](https://rast.nmpdr.org/seedviewer.cgi?page=FunctionalRole&role=PTS%20system,%20fructose-specific%20IIC%20component%20(EC%202.7.1.69)&subsystem_name=Fructose_utilization) |
| Carbohydrates | Monosaccharides | [Fructose utilization](https://rast.nmpdr.org/seedviewer.cgi?page=Subsystems&subsystem=Fructose_utilization&organism=549.408) | [Fructose repressor FruR, LacI family](https://rast.nmpdr.org/seedviewer.cgi?page=FunctionalRole&role=Fructose%20repressor%20FruR,%20LacI%20family&subsystem_name=Fructose_utilization) |
| Carbohydrates | Monosaccharides | [Fructose utilization](https://rast.nmpdr.org/seedviewer.cgi?page=Subsystems&subsystem=Fructose_utilization&organism=549.408) | [PTS system, fructose-specific IIB component (EC 2.7.1.69)](https://rast.nmpdr.org/seedviewer.cgi?page=FunctionalRole&role=PTS%20system,%20fructose-specific%20IIB%20component%20(EC%202.7.1.69)&subsystem_name=Fructose_utilization) |
| Carbohydrates | Monosaccharides | [Fructose utilization](https://rast.nmpdr.org/seedviewer.cgi?page=Subsystems&subsystem=Fructose_utilization&organism=549.408) | [Transaldolase (EC 2.2.1.2)](https://rast.nmpdr.org/seedviewer.cgi?page=FunctionalRole&role=Transaldolase%20(EC%202.2.1.2)&subsystem_name=Fructose_utilization) |
| Carbohydrates | Monosaccharides | [Fructose utilization](https://rast.nmpdr.org/seedviewer.cgi?page=Subsystems&subsystem=Fructose_utilization&organism=549.408) | [1-phosphofructokinase (EC 2.7.1.56)](https://rast.nmpdr.org/seedviewer.cgi?page=FunctionalRole&role=1-phosphofructokinase%20(EC%202.7.1.56)&subsystem_name=Fructose_utilization) |
| Carbohydrates | Monosaccharides | [Fructose utilization](https://rast.nmpdr.org/seedviewer.cgi?page=Subsystems&subsystem=Fructose_utilization&organism=549.408) | [PTS system, fructose-specific IIA component (EC 2.7.1.69)](https://rast.nmpdr.org/seedviewer.cgi?page=FunctionalRole&role=PTS%20system,%20fructose-specific%20IIA%20component%20(EC%202.7.1.69)&subsystem_name=Fructose_utilization) |
| Carbohydrates | Monosaccharides | [Fructose utilization](https://rast.nmpdr.org/seedviewer.cgi?page=Subsystems&subsystem=Fructose_utilization&organism=549.408) | [Fructose-specific phosphocarrier protein HPr (EC 2.7.1.69)](https://rast.nmpdr.org/seedviewer.cgi?page=FunctionalRole&role=Fructose-specific%20phosphocarrier%20protein%20HPr%20(EC%202.7.1.69)&subsystem_name=Fructose_utilization) |
| Carbohydrates | Monosaccharides | [Fructose utilization](https://rast.nmpdr.org/seedviewer.cgi?page=Subsystems&subsystem=Fructose_utilization&organism=549.408) | [Fructokinase (EC 2.7.1.4)](https://rast.nmpdr.org/seedviewer.cgi?page=FunctionalRole&role=Fructokinase%20(EC%202.7.1.4)&subsystem_name=Fructose_utilization) |
| Carbohydrates | Monosaccharides | [D-galactarate, D-glucarate and D-glycerate catabolism - gjo](https://rast.nmpdr.org/seedviewer.cgi?page=Subsystems&subsystem=D-galactarate,_D-glucarate_and_D-glycerate_catabolism_-_gjo&organism=549.408) | [D-galactarate dehydratase (EC 4.2.1.42)](https://rast.nmpdr.org/seedviewer.cgi?page=FunctionalRole&role=D-galactarate%20dehydratase%20(EC%204.2.1.42)&subsystem_name=D-galactarate,_D-glucarate_and_D-glycerate_catabolism_-_gjo) |
| Carbohydrates | Monosaccharides | [D-galactarate, D-glucarate and D-glycerate catabolism - gjo](https://rast.nmpdr.org/seedviewer.cgi?page=Subsystems&subsystem=D-galactarate,_D-glucarate_and_D-glycerate_catabolism_-_gjo&organism=549.408) | [2-dehydro-3-deoxyglucarate aldolase (EC 4.1.2.20)](https://rast.nmpdr.org/seedviewer.cgi?page=FunctionalRole&role=2-dehydro-3-deoxyglucarate%20aldolase%20(EC%204.1.2.20)&subsystem_name=D-galactarate,_D-glucarate_and_D-glycerate_catabolism_-_gjo) |
| Carbohydrates | Monosaccharides | [D-galactarate, D-glucarate and D-glycerate catabolism - gjo](https://rast.nmpdr.org/seedviewer.cgi?page=Subsystems&subsystem=D-galactarate,_D-glucarate_and_D-glycerate_catabolism_-_gjo&organism=549.408) | [D-glucarate permease](https://rast.nmpdr.org/seedviewer.cgi?page=FunctionalRole&role=D-glucarate%20permease&subsystem_name=D-galactarate,_D-glucarate_and_D-glycerate_catabolism_-_gjo) |
| Carbohydrates | Monosaccharides | [D-galactarate, D-glucarate and D-glycerate catabolism - gjo](https://rast.nmpdr.org/seedviewer.cgi?page=Subsystems&subsystem=D-galactarate,_D-glucarate_and_D-glycerate_catabolism_-_gjo&organism=549.408) | [2-hydroxy-3-oxopropionate reductase (EC 1.1.1.60)](https://rast.nmpdr.org/seedviewer.cgi?page=FunctionalRole&role=2-hydroxy-3-oxopropionate%20reductase%20(EC%201.1.1.60)&subsystem_name=D-galactarate,_D-glucarate_and_D-glycerate_catabolism_-_gjo) |
| Carbohydrates | Monosaccharides | [D-galactarate, D-glucarate and D-glycerate catabolism - gjo](https://rast.nmpdr.org/seedviewer.cgi?page=Subsystems&subsystem=D-galactarate,_D-glucarate_and_D-glycerate_catabolism_-_gjo&organism=549.408) | [Glucarate dehydratase (EC 4.2.1.40)](https://rast.nmpdr.org/seedviewer.cgi?page=FunctionalRole&role=Glucarate%20dehydratase%20(EC%204.2.1.40)&subsystem_name=D-galactarate,_D-glucarate_and_D-glycerate_catabolism_-_gjo) |
| Carbohydrates | Monosaccharides | [D-galactarate, D-glucarate and D-glycerate catabolism - gjo](https://rast.nmpdr.org/seedviewer.cgi?page=Subsystems&subsystem=D-galactarate,_D-glucarate_and_D-glycerate_catabolism_-_gjo&organism=549.408) | [Sugar diacid utilization regulator SdaR](https://rast.nmpdr.org/seedviewer.cgi?page=FunctionalRole&role=Sugar%20diacid%20utilization%20regulator%20SdaR&subsystem_name=D-galactarate,_D-glucarate_and_D-glycerate_catabolism_-_gjo) |
| Carbohydrates | Monosaccharides | [D-galactarate, D-glucarate and D-glycerate catabolism - gjo](https://rast.nmpdr.org/seedviewer.cgi?page=Subsystems&subsystem=D-galactarate,_D-glucarate_and_D-glycerate_catabolism_-_gjo&organism=549.408) | [Glycerate kinase (EC 2.7.1.31)](https://rast.nmpdr.org/seedviewer.cgi?page=FunctionalRole&role=Glycerate%20kinase%20(EC%202.7.1.31)&subsystem_name=D-galactarate,_D-glucarate_and_D-glycerate_catabolism_-_gjo) |
| Carbohydrates | Monosaccharides | [D-Galacturonate and D-Glucuronate Utilization](https://rast.nmpdr.org/seedviewer.cgi?page=Subsystems&subsystem=D-Galacturonate_and_D-Glucuronate_Utilization&organism=549.408) | [Fructuronate transporter GntP](https://rast.nmpdr.org/seedviewer.cgi?page=FunctionalRole&role=Fructuronate%20transporter%20GntP&subsystem_name=D-Galacturonate_and_D-Glucuronate_Utilization) |
| Carbohydrates | Monosaccharides | [D-Galacturonate and D-Glucuronate Utilization](https://rast.nmpdr.org/seedviewer.cgi?page=Subsystems&subsystem=D-Galacturonate_and_D-Glucuronate_Utilization&organism=549.408) | [Mannonate dehydratase (EC 4.2.1.8)](https://rast.nmpdr.org/seedviewer.cgi?page=FunctionalRole&role=Mannonate%20dehydratase%20(EC%204.2.1.8)&subsystem_name=D-Galacturonate_and_D-Glucuronate_Utilization) |
| Carbohydrates | Monosaccharides | [D-Galacturonate and D-Glucuronate Utilization](https://rast.nmpdr.org/seedviewer.cgi?page=Subsystems&subsystem=D-Galacturonate_and_D-Glucuronate_Utilization&organism=549.408) | [Rhamnogalacturonides degradation protein RhiN](https://rast.nmpdr.org/seedviewer.cgi?page=FunctionalRole&role=Rhamnogalacturonides%20degradation%20protein%20RhiN&subsystem_name=D-Galacturonate_and_D-Glucuronate_Utilization) |
| Carbohydrates | Monosaccharides | [D-Galacturonate and D-Glucuronate Utilization](https://rast.nmpdr.org/seedviewer.cgi?page=Subsystems&subsystem=D-Galacturonate_and_D-Glucuronate_Utilization&organism=549.408) | [2-dehydro-3-deoxyphosphogluconate aldolase (EC 4.1.2.14)](https://rast.nmpdr.org/seedviewer.cgi?page=FunctionalRole&role=2-dehydro-3-deoxyphosphogluconate%20aldolase%20(EC%204.1.2.14)&subsystem_name=D-Galacturonate_and_D-Glucuronate_Utilization) |
| Carbohydrates | Monosaccharides | [D-Galacturonate and D-Glucuronate Utilization](https://rast.nmpdr.org/seedviewer.cgi?page=Subsystems&subsystem=D-Galacturonate_and_D-Glucuronate_Utilization&organism=549.408) | [D-galactarate dehydratase (EC 4.2.1.42)](https://rast.nmpdr.org/seedviewer.cgi?page=FunctionalRole&role=D-galactarate%20dehydratase%20(EC%204.2.1.42)&subsystem_name=D-Galacturonate_and_D-Glucuronate_Utilization) |
| Carbohydrates | Monosaccharides | [D-Galacturonate and D-Glucuronate Utilization](https://rast.nmpdr.org/seedviewer.cgi?page=Subsystems&subsystem=D-Galacturonate_and_D-Glucuronate_Utilization&organism=549.408) | [Altronate oxidoreductase (EC 1.1.1.58)](https://rast.nmpdr.org/seedviewer.cgi?page=FunctionalRole&role=Altronate%20oxidoreductase%20(EC%201.1.1.58)&subsystem_name=D-Galacturonate_and_D-Glucuronate_Utilization) |
| Carbohydrates | Monosaccharides | [D-Galacturonate and D-Glucuronate Utilization](https://rast.nmpdr.org/seedviewer.cgi?page=Subsystems&subsystem=D-Galacturonate_and_D-Glucuronate_Utilization&organism=549.408) | [Polygalacturonase (EC 3.2.1.15)](https://rast.nmpdr.org/seedviewer.cgi?page=FunctionalRole&role=Polygalacturonase%20(EC%203.2.1.15)&subsystem_name=D-Galacturonate_and_D-Glucuronate_Utilization) |
| Carbohydrates | Monosaccharides | [D-Galacturonate and D-Glucuronate Utilization](https://rast.nmpdr.org/seedviewer.cgi?page=Subsystems&subsystem=D-Galacturonate_and_D-Glucuronate_Utilization&organism=549.408) | [2-keto-3-deoxygluconate permease (KDG permease)](https://rast.nmpdr.org/seedviewer.cgi?page=FunctionalRole&role=2-keto-3-deoxygluconate%20permease%20(KDG%20permease)&subsystem_name=D-Galacturonate_and_D-Glucuronate_Utilization) |
| Carbohydrates | Monosaccharides | [D-Galacturonate and D-Glucuronate Utilization](https://rast.nmpdr.org/seedviewer.cgi?page=Subsystems&subsystem=D-Galacturonate_and_D-Glucuronate_Utilization&organism=549.408) | [Hexuronate transporter](https://rast.nmpdr.org/seedviewer.cgi?page=FunctionalRole&role=Hexuronate%20transporter&subsystem_name=D-Galacturonate_and_D-Glucuronate_Utilization) |
| Carbohydrates | Monosaccharides | [D-Galacturonate and D-Glucuronate Utilization](https://rast.nmpdr.org/seedviewer.cgi?page=Subsystems&subsystem=D-Galacturonate_and_D-Glucuronate_Utilization&organism=549.408) | [Transcriptional regulator KdgR, KDG operon repressor](https://rast.nmpdr.org/seedviewer.cgi?page=FunctionalRole&role=Transcriptional%20regulator%20KdgR,%20KDG%20operon%20repressor&subsystem_name=D-Galacturonate_and_D-Glucuronate_Utilization) |
| Carbohydrates | Monosaccharides | [D-Galacturonate and D-Glucuronate Utilization](https://rast.nmpdr.org/seedviewer.cgi?page=Subsystems&subsystem=D-Galacturonate_and_D-Glucuronate_Utilization&organism=549.408) | [2-dehydro-3-deoxygluconate kinase (EC 2.7.1.45)](https://rast.nmpdr.org/seedviewer.cgi?page=FunctionalRole&role=2-dehydro-3-deoxygluconate%20kinase%20(EC%202.7.1.45)&subsystem_name=D-Galacturonate_and_D-Glucuronate_Utilization) |
| Carbohydrates | Monosaccharides | [D-Galacturonate and D-Glucuronate Utilization](https://rast.nmpdr.org/seedviewer.cgi?page=Subsystems&subsystem=D-Galacturonate_and_D-Glucuronate_Utilization&organism=549.408) | [Pectin degradation protein KdgF](https://rast.nmpdr.org/seedviewer.cgi?page=FunctionalRole&role=Pectin%20degradation%20protein%20KdgF&subsystem_name=D-Galacturonate_and_D-Glucuronate_Utilization) |
| Carbohydrates | Monosaccharides | [D-Galacturonate and D-Glucuronate Utilization](https://rast.nmpdr.org/seedviewer.cgi?page=Subsystems&subsystem=D-Galacturonate_and_D-Glucuronate_Utilization&organism=549.408) | [4-deoxy-L-threo-5-hexosulose-uronate ketol-isomerase (EC 5.3.1.17)](https://rast.nmpdr.org/seedviewer.cgi?page=FunctionalRole&role=4-deoxy-L-threo-5-hexosulose-uronate%20ketol-isomerase%20(EC%205.3.1.17)&subsystem_name=D-Galacturonate_and_D-Glucuronate_Utilization) |
| Carbohydrates | Monosaccharides | [D-Galacturonate and D-Glucuronate Utilization](https://rast.nmpdr.org/seedviewer.cgi?page=Subsystems&subsystem=D-Galacturonate_and_D-Glucuronate_Utilization&organism=549.408) | [Alpha-glucosidase (EC 3.2.1.20)](https://rast.nmpdr.org/seedviewer.cgi?page=FunctionalRole&role=Alpha-glucosidase%20(EC%203.2.1.20)&subsystem_name=D-Galacturonate_and_D-Glucuronate_Utilization) |
| Carbohydrates | Monosaccharides | [D-Galacturonate and D-Glucuronate Utilization](https://rast.nmpdr.org/seedviewer.cgi?page=Subsystems&subsystem=D-Galacturonate_and_D-Glucuronate_Utilization&organism=549.408) | [2-deoxy-D-gluconate 3-dehydrogenase (EC 1.1.1.125)](https://rast.nmpdr.org/seedviewer.cgi?page=FunctionalRole&role=2-deoxy-D-gluconate%203-dehydrogenase%20(EC%201.1.1.125)&subsystem_name=D-Galacturonate_and_D-Glucuronate_Utilization) |
| Carbohydrates | Monosaccharides | [D-Galacturonate and D-Glucuronate Utilization](https://rast.nmpdr.org/seedviewer.cgi?page=Subsystems&subsystem=D-Galacturonate_and_D-Glucuronate_Utilization&organism=549.408) | [D-mannonate oxidoreductase (EC 1.1.1.57)](https://rast.nmpdr.org/seedviewer.cgi?page=FunctionalRole&role=D-mannonate%20oxidoreductase%20(EC%201.1.1.57)&subsystem_name=D-Galacturonate_and_D-Glucuronate_Utilization) |
| Carbohydrates | Monosaccharides | [D-Galacturonate and D-Glucuronate Utilization](https://rast.nmpdr.org/seedviewer.cgi?page=Subsystems&subsystem=D-Galacturonate_and_D-Glucuronate_Utilization&organism=549.408) | [Uronate isomerase (EC 5.3.1.12)](https://rast.nmpdr.org/seedviewer.cgi?page=FunctionalRole&role=Uronate%20isomerase%20(EC%205.3.1.12)&subsystem_name=D-Galacturonate_and_D-Glucuronate_Utilization) |
| Carbohydrates | Monosaccharides | [D-Galacturonate and D-Glucuronate Utilization](https://rast.nmpdr.org/seedviewer.cgi?page=Subsystems&subsystem=D-Galacturonate_and_D-Glucuronate_Utilization&organism=549.408) | [Hexuronate utilization operon transcriptional repressor ExuR](https://rast.nmpdr.org/seedviewer.cgi?page=FunctionalRole&role=Hexuronate%20utilization%20operon%20transcriptional%20repressor%20ExuR&subsystem_name=D-Galacturonate_and_D-Glucuronate_Utilization) |
| Carbohydrates | Monosaccharides | [L-rhamnose utilization](https://rast.nmpdr.org/seedviewer.cgi?page=Subsystems&subsystem=L-rhamnose_utilization&organism=549.408) | [Rhamnulose-1-phosphate aldolase (EC 4.1.2.19)](https://rast.nmpdr.org/seedviewer.cgi?page=FunctionalRole&role=Rhamnulose-1-phosphate%20aldolase%20(EC%204.1.2.19)&subsystem_name=L-rhamnose_utilization) |
| Carbohydrates | Monosaccharides | [L-rhamnose utilization](https://rast.nmpdr.org/seedviewer.cgi?page=Subsystems&subsystem=L-rhamnose_utilization&organism=549.408) | [Lactaldehyde reductase (EC 1.1.1.77)](https://rast.nmpdr.org/seedviewer.cgi?page=FunctionalRole&role=Lactaldehyde%20reductase%20(EC%201.1.1.77)&subsystem_name=L-rhamnose_utilization) |
| Carbohydrates | Monosaccharides | [L-rhamnose utilization](https://rast.nmpdr.org/seedviewer.cgi?page=Subsystems&subsystem=L-rhamnose_utilization&organism=549.408) | [L-rhamnose operon regulatory protein RhaS](https://rast.nmpdr.org/seedviewer.cgi?page=FunctionalRole&role=L-rhamnose%20operon%20regulatory%20protein%20RhaS&subsystem_name=L-rhamnose_utilization) |
| Carbohydrates | Monosaccharides | [L-rhamnose utilization](https://rast.nmpdr.org/seedviewer.cgi?page=Subsystems&subsystem=L-rhamnose_utilization&organism=549.408) | [L-rhamnose isomerase (EC 5.3.1.14)](https://rast.nmpdr.org/seedviewer.cgi?page=FunctionalRole&role=L-rhamnose%20isomerase%20(EC%205.3.1.14)&subsystem_name=L-rhamnose_utilization) |
| Carbohydrates | Monosaccharides | [L-rhamnose utilization](https://rast.nmpdr.org/seedviewer.cgi?page=Subsystems&subsystem=L-rhamnose_utilization&organism=549.408) | [Rhamnogalacturonides degradation protein RhiN](https://rast.nmpdr.org/seedviewer.cgi?page=FunctionalRole&role=Rhamnogalacturonides%20degradation%20protein%20RhiN&subsystem_name=L-rhamnose_utilization) |
| Carbohydrates | Monosaccharides | [L-rhamnose utilization](https://rast.nmpdr.org/seedviewer.cgi?page=Subsystems&subsystem=L-rhamnose_utilization&organism=549.408) | [L-rhamnose operon transcriptional activator RhaR](https://rast.nmpdr.org/seedviewer.cgi?page=FunctionalRole&role=L-rhamnose%20operon%20transcriptional%20activator%20RhaR&subsystem_name=L-rhamnose_utilization) |
| Carbohydrates | Monosaccharides | [L-rhamnose utilization](https://rast.nmpdr.org/seedviewer.cgi?page=Subsystems&subsystem=L-rhamnose_utilization&organism=549.408) | [Rhamnogalacturonide transporter RhiT](https://rast.nmpdr.org/seedviewer.cgi?page=FunctionalRole&role=Rhamnogalacturonide%20transporter%20RhiT&subsystem_name=L-rhamnose_utilization) |
| Carbohydrates | Monosaccharides | [L-rhamnose utilization](https://rast.nmpdr.org/seedviewer.cgi?page=Subsystems&subsystem=L-rhamnose_utilization&organism=549.408) | [Rhamnulokinase (EC 2.7.1.5)](https://rast.nmpdr.org/seedviewer.cgi?page=FunctionalRole&role=Rhamnulokinase%20(EC%202.7.1.5)&subsystem_name=L-rhamnose_utilization) |
| Carbohydrates | Monosaccharides | [L-rhamnose utilization](https://rast.nmpdr.org/seedviewer.cgi?page=Subsystems&subsystem=L-rhamnose_utilization&organism=549.408) | [L-rhamnose mutarotase](https://rast.nmpdr.org/seedviewer.cgi?page=FunctionalRole&role=L-rhamnose%20mutarotase&subsystem_name=L-rhamnose_utilization) |
| Carbohydrates | Monosaccharides | [L-rhamnose utilization](https://rast.nmpdr.org/seedviewer.cgi?page=Subsystems&subsystem=L-rhamnose_utilization&organism=549.408) | [L-rhamnose-proton symporter](https://rast.nmpdr.org/seedviewer.cgi?page=FunctionalRole&role=L-rhamnose-proton%20symporter&subsystem_name=L-rhamnose_utilization) |
| Carbohydrates | Monosaccharides | [L-rhamnose utilization](https://rast.nmpdr.org/seedviewer.cgi?page=Subsystems&subsystem=L-rhamnose_utilization&organism=549.408) | [Aldehyde dehydrogenase A (EC 1.2.1.22)](https://rast.nmpdr.org/seedviewer.cgi?page=FunctionalRole&role=Aldehyde%20dehydrogenase%20A%20(EC%201.2.1.22)&subsystem_name=L-rhamnose_utilization) |
| Carbohydrates | Monosaccharides | [L-rhamnose utilization](https://rast.nmpdr.org/seedviewer.cgi?page=Subsystems&subsystem=L-rhamnose_utilization&organism=549.408) | [L-lactate dehydrogenase (EC 1.1.2.3)](https://rast.nmpdr.org/seedviewer.cgi?page=FunctionalRole&role=L-lactate%20dehydrogenase%20(EC%201.1.2.3)&subsystem_name=L-rhamnose_utilization) |
| Carbohydrates | Monosaccharides | [D-ribose utilization](https://rast.nmpdr.org/seedviewer.cgi?page=Subsystems&subsystem=D-ribose_utilization&organism=549.408) | [Ribose ABC transport system, periplasmic ribose-binding protein RbsB (TC 3.A.1.2.1)](https://rast.nmpdr.org/seedviewer.cgi?page=FunctionalRole&role=Ribose%20ABC%20transport%20system,%20periplasmic%20ribose-binding%20protein%20RbsB%20(TC%203.A.1.2.1)&subsystem_name=D-ribose_utilization) |
| Carbohydrates | Monosaccharides | [D-ribose utilization](https://rast.nmpdr.org/seedviewer.cgi?page=Subsystems&subsystem=D-ribose_utilization&organism=549.408) | [Ribose ABC transport system, ATP-binding protein RbsA (TC 3.A.1.2.1)](https://rast.nmpdr.org/seedviewer.cgi?page=FunctionalRole&role=Ribose%20ABC%20transport%20system,%20ATP-binding%20protein%20RbsA%20(TC%203.A.1.2.1)&subsystem_name=D-ribose_utilization) |
| Carbohydrates | Monosaccharides | [D-ribose utilization](https://rast.nmpdr.org/seedviewer.cgi?page=Subsystems&subsystem=D-ribose_utilization&organism=549.408) | [Ribose operon repressor](https://rast.nmpdr.org/seedviewer.cgi?page=FunctionalRole&role=Ribose%20operon%20repressor&subsystem_name=D-ribose_utilization) |
| Carbohydrates | Monosaccharides | [D-ribose utilization](https://rast.nmpdr.org/seedviewer.cgi?page=Subsystems&subsystem=D-ribose_utilization&organism=549.408) | [Ribose ABC transport system, permease protein RbsC (TC 3.A.1.2.1)](https://rast.nmpdr.org/seedviewer.cgi?page=FunctionalRole&role=Ribose%20ABC%20transport%20system,%20permease%20protein%20RbsC%20(TC%203.A.1.2.1)&subsystem_name=D-ribose_utilization) |
| Carbohydrates | Monosaccharides | [D-ribose utilization](https://rast.nmpdr.org/seedviewer.cgi?page=Subsystems&subsystem=D-ribose_utilization&organism=549.408) | [Ribokinase (EC 2.7.1.15)](https://rast.nmpdr.org/seedviewer.cgi?page=FunctionalRole&role=Ribokinase%20(EC%202.7.1.15)&subsystem_name=D-ribose_utilization) |
| Carbohydrates | Monosaccharides | [D-ribose utilization](https://rast.nmpdr.org/seedviewer.cgi?page=Subsystems&subsystem=D-ribose_utilization&organism=549.408) | [Ribose 5-phosphate isomerase A (EC 5.3.1.6)](https://rast.nmpdr.org/seedviewer.cgi?page=FunctionalRole&role=Ribose%205-phosphate%20isomerase%20A%20(EC%205.3.1.6)&subsystem_name=D-ribose_utilization) |
| Carbohydrates | Monosaccharides | [D-ribose utilization](https://rast.nmpdr.org/seedviewer.cgi?page=Subsystems&subsystem=D-ribose_utilization&organism=549.408) | [Ribose ABC transport system, high affinity permease RbsD (TC 3.A.1.2.1)](https://rast.nmpdr.org/seedviewer.cgi?page=FunctionalRole&role=Ribose%20ABC%20transport%20system,%20high%20affinity%20permease%20RbsD%20(TC%203.A.1.2.1)&subsystem_name=D-ribose_utilization) |
| Carbohydrates | Monosaccharides | [Xylose utilization](https://rast.nmpdr.org/seedviewer.cgi?page=Subsystems&subsystem=Xylose_utilization&organism=549.408) | [Xylose activator XylR (AraC family)](https://rast.nmpdr.org/seedviewer.cgi?page=FunctionalRole&role=Xylose%20activator%20XylR%20(AraC%20family)&subsystem_name=Xylose_utilization) |
| Carbohydrates | Monosaccharides | [Xylose utilization](https://rast.nmpdr.org/seedviewer.cgi?page=Subsystems&subsystem=Xylose_utilization&organism=549.408) | [Xylose ABC transporter, periplasmic xylose-binding protein XylF](https://rast.nmpdr.org/seedviewer.cgi?page=FunctionalRole&role=Xylose%20ABC%20transporter,%20periplasmic%20xylose-binding%20protein%20XylF&subsystem_name=Xylose_utilization) |
| Carbohydrates | Monosaccharides | [Xylose utilization](https://rast.nmpdr.org/seedviewer.cgi?page=Subsystems&subsystem=Xylose_utilization&organism=549.408) | [Xylose ABC transporter, permease protein XylH](https://rast.nmpdr.org/seedviewer.cgi?page=FunctionalRole&role=Xylose%20ABC%20transporter,%20permease%20protein%20XylH&subsystem_name=Xylose_utilization) |
| Carbohydrates | Monosaccharides | [Xylose utilization](https://rast.nmpdr.org/seedviewer.cgi?page=Subsystems&subsystem=Xylose_utilization&organism=549.408) | [Xylanase](https://rast.nmpdr.org/seedviewer.cgi?page=FunctionalRole&role=Xylanase&subsystem_name=Xylose_utilization) |
| Carbohydrates | Monosaccharides | [Xylose utilization](https://rast.nmpdr.org/seedviewer.cgi?page=Subsystems&subsystem=Xylose_utilization&organism=549.408) | [Xylose isomerase (EC 5.3.1.5)](https://rast.nmpdr.org/seedviewer.cgi?page=FunctionalRole&role=Xylose%20isomerase%20(EC%205.3.1.5)&subsystem_name=Xylose_utilization) |
| Carbohydrates | Monosaccharides | [Xylose utilization](https://rast.nmpdr.org/seedviewer.cgi?page=Subsystems&subsystem=Xylose_utilization&organism=549.408) | [D-xylose transport ATP-binding protein XylG](https://rast.nmpdr.org/seedviewer.cgi?page=FunctionalRole&role=D-xylose%20transport%20ATP-binding%20protein%20XylG&subsystem_name=Xylose_utilization) |
| Carbohydrates | Monosaccharides | [Xylose utilization](https://rast.nmpdr.org/seedviewer.cgi?page=Subsystems&subsystem=Xylose_utilization&organism=549.408) | [Xylulose kinase (EC 2.7.1.17)](https://rast.nmpdr.org/seedviewer.cgi?page=FunctionalRole&role=Xylulose%20kinase%20(EC%202.7.1.17)&subsystem_name=Xylose_utilization) |
| Carbohydrates | Monosaccharides | [Xylose utilization](https://rast.nmpdr.org/seedviewer.cgi?page=Subsystems&subsystem=Xylose_utilization&organism=549.408) | [Xyloside transporter XynT](https://rast.nmpdr.org/seedviewer.cgi?page=FunctionalRole&role=Xyloside%20transporter%20XynT&subsystem_name=Xylose_utilization) |
| Carbohydrates | Monosaccharides | [Xylose utilization](https://rast.nmpdr.org/seedviewer.cgi?page=Subsystems&subsystem=Xylose_utilization&organism=549.408) | [Beta-xylosidase (EC 3.2.1.37)](https://rast.nmpdr.org/seedviewer.cgi?page=FunctionalRole&role=Beta-xylosidase%20(EC%203.2.1.37)&subsystem_name=Xylose_utilization) |
| Carbohydrates | Monosaccharides | [D-galactarate, D-glucarate and D-glycerate catabolism](https://rast.nmpdr.org/seedviewer.cgi?page=Subsystems&subsystem=D-galactarate,_D-glucarate_and_D-glycerate_catabolism&organism=549.408) | [D-galactarate dehydratase (EC 4.2.1.42)](https://rast.nmpdr.org/seedviewer.cgi?page=FunctionalRole&role=D-galactarate%20dehydratase%20(EC%204.2.1.42)&subsystem_name=D-galactarate,_D-glucarate_and_D-glycerate_catabolism) |
| Carbohydrates | Monosaccharides | [D-galactarate, D-glucarate and D-glycerate catabolism](https://rast.nmpdr.org/seedviewer.cgi?page=Subsystems&subsystem=D-galactarate,_D-glucarate_and_D-glycerate_catabolism&organism=549.408) | [2-dehydro-3-deoxyglucarate aldolase (EC 4.1.2.20)](https://rast.nmpdr.org/seedviewer.cgi?page=FunctionalRole&role=2-dehydro-3-deoxyglucarate%20aldolase%20(EC%204.1.2.20)&subsystem_name=D-galactarate,_D-glucarate_and_D-glycerate_catabolism) |
| Carbohydrates | Monosaccharides | [D-galactarate, D-glucarate and D-glycerate catabolism](https://rast.nmpdr.org/seedviewer.cgi?page=Subsystems&subsystem=D-galactarate,_D-glucarate_and_D-glycerate_catabolism&organism=549.408) | [D-glucarate permease](https://rast.nmpdr.org/seedviewer.cgi?page=FunctionalRole&role=D-glucarate%20permease&subsystem_name=D-galactarate,_D-glucarate_and_D-glycerate_catabolism) |
| Carbohydrates | Monosaccharides | [D-galactarate, D-glucarate and D-glycerate catabolism](https://rast.nmpdr.org/seedviewer.cgi?page=Subsystems&subsystem=D-galactarate,_D-glucarate_and_D-glycerate_catabolism&organism=549.408) | [2-hydroxy-3-oxopropionate reductase (EC 1.1.1.60)](https://rast.nmpdr.org/seedviewer.cgi?page=FunctionalRole&role=2-hydroxy-3-oxopropionate%20reductase%20(EC%201.1.1.60)&subsystem_name=D-galactarate,_D-glucarate_and_D-glycerate_catabolism) |
| Carbohydrates | Monosaccharides | [D-galactarate, D-glucarate and D-glycerate catabolism](https://rast.nmpdr.org/seedviewer.cgi?page=Subsystems&subsystem=D-galactarate,_D-glucarate_and_D-glycerate_catabolism&organism=549.408) | [Glucarate dehydratase (EC 4.2.1.40)](https://rast.nmpdr.org/seedviewer.cgi?page=FunctionalRole&role=Glucarate%20dehydratase%20(EC%204.2.1.40)&subsystem_name=D-galactarate,_D-glucarate_and_D-glycerate_catabolism) |
| Carbohydrates | Monosaccharides | [D-galactarate, D-glucarate and D-glycerate catabolism](https://rast.nmpdr.org/seedviewer.cgi?page=Subsystems&subsystem=D-galactarate,_D-glucarate_and_D-glycerate_catabolism&organism=549.408) | [Sugar diacid utilization regulator SdaR](https://rast.nmpdr.org/seedviewer.cgi?page=FunctionalRole&role=Sugar%20diacid%20utilization%20regulator%20SdaR&subsystem_name=D-galactarate,_D-glucarate_and_D-glycerate_catabolism) |
| Carbohydrates | Monosaccharides | [D-galactarate, D-glucarate and D-glycerate catabolism](https://rast.nmpdr.org/seedviewer.cgi?page=Subsystems&subsystem=D-galactarate,_D-glucarate_and_D-glycerate_catabolism&organism=549.408) | [Glycerate kinase (EC 2.7.1.31)](https://rast.nmpdr.org/seedviewer.cgi?page=FunctionalRole&role=Glycerate%20kinase%20(EC%202.7.1.31)&subsystem_name=D-galactarate,_D-glucarate_and_D-glycerate_catabolism) |
| Carbohydrates | Monosaccharides | [L-Arabinose utilization](https://rast.nmpdr.org/seedviewer.cgi?page=Subsystems&subsystem=L-Arabinose_utilization&organism=549.408) | [Arabinose operon regulatory protein](https://rast.nmpdr.org/seedviewer.cgi?page=FunctionalRole&role=Arabinose%20operon%20regulatory%20protein&subsystem_name=L-Arabinose_utilization) |
| Carbohydrates | Monosaccharides |  | [L-arabinose transport ATP-binding protein AraG (TC 3.A.1.2.2)](https://rast.nmpdr.org/seedviewer.cgi?page=FunctionalRole&role=L-arabinose%20transport%20ATP-binding%20protein%20AraG%20(TC%203.A.1.2.2)&subsystem_name=L-Arabinose_utilization) |
| Carbohydrates | Monosaccharides |  | [L-arabinose isomerase (EC 5.3.1.4)](https://rast.nmpdr.org/seedviewer.cgi?page=FunctionalRole&role=L-arabinose%20isomerase%20(EC%205.3.1.4)&subsystem_name=L-Arabinose_utilization) |
| Carbohydrates | Monosaccharides |  | [Arabinose-proton symporter](https://rast.nmpdr.org/seedviewer.cgi?page=FunctionalRole&role=Arabinose-proton%20symporter&subsystem_name=L-Arabinose_utilization) |
| Carbohydrates | Monosaccharides |  | [Alpha-L-arabinofuranosidase II precursor (EC 3.2.1.55)](https://rast.nmpdr.org/seedviewer.cgi?page=FunctionalRole&role=Alpha-L-arabinofuranosidase%20II%20precursor%20(EC%203.2.1.55)&subsystem_name=L-Arabinose_utilization) |
| Carbohydrates | Monosaccharides |  | [L-arabinose-binding periplasmic protein precursor AraF (TC 3.A.1.2.2)](https://rast.nmpdr.org/seedviewer.cgi?page=FunctionalRole&role=L-arabinose-binding%20periplasmic%20protein%20precursor%20AraF%20(TC%203.A.1.2.2)&subsystem_name=L-Arabinose_utilization) |
| Carbohydrates | Monosaccharides |  | [L-ribulose-5-phosphate 4-epimerase (EC 5.1.3.4)](https://rast.nmpdr.org/seedviewer.cgi?page=FunctionalRole&role=L-ribulose-5-phosphate%204-epimerase%20(EC%205.1.3.4)&subsystem_name=L-Arabinose_utilization) |
| Carbohydrates | Monosaccharides |  | [L-arabinolactonase (EC 3.1.1.15)](https://rast.nmpdr.org/seedviewer.cgi?page=FunctionalRole&role=L-arabinolactonase%20(EC%203.1.1.15)&subsystem_name=L-Arabinose_utilization) |
| Carbohydrates | Monosaccharides |  | [Ribulokinase (EC 2.7.1.16)](https://rast.nmpdr.org/seedviewer.cgi?page=FunctionalRole&role=Ribulokinase%20(EC%202.7.1.16)&subsystem_name=L-Arabinose_utilization) |
| Carbohydrates | Monosaccharides |  | [L-arabinose transport system permease protein (TC 3.A.1.2.2)](https://rast.nmpdr.org/seedviewer.cgi?page=FunctionalRole&role=L-arabinose%20transport%20system%20permease%20protein%20(TC%203.A.1.2.2)&subsystem_name=L-Arabinose_utilization) |
